# Enrichment of Food With Tannin Extracts Promotes Healthy Changes in the Human Gut Microbiota

**DOI:** 10.3389/fmicb.2021.625782

**Published:** 2021-03-16

**Authors:** Silvia Molino, Alberto Lerma-Aguilera, Nuria Jiménez-Hernández, María José Gosalbes, José Ángel Rufián-Henares, M. Pilar Francino

**Affiliations:** ^1^Departamento de Nutrición y Bromatología, Instituto de Nutrición y Tecnología de los Alimentos, Centro de Investigación Biomédica, Universidad de Granada, Granada, Spain; ^2^Area de Genòmica i Salut, Fundació per al Foment de la Investigació Sanitària i Biomèdica de la Comunitat Valenciana (FISABIO-Salut Pública), València, Spain; ^3^CIBER en Epidemiología y Salud Pública, Madrid, Spain; ^4^Instituto de Investigación Biosanitaria ibs.Granada, Granada, Spain

**Keywords:** tannins, quebracho, chestnut, tara, *in vitro* digestion-fermentation, gut microbiota, short chain fatty acids

## Abstract

Food and food bioactive components are major drivers of modulation of the human gut microbiota. Tannin extracts consist of a mix of bioactive compounds, which are already exploited in the food industry for their chemical and sensorial properties. The aim of our study was to explore the viability of associations between tannin wood extracts of different origin and food as gut microbiota modulators. 16S rRNA amplicon next-generation sequencing (NGS) was used to test the effects on the gut microbiota of tannin extracts from quebracho, chestnut, and tara associated with commercial food products with different composition in macronutrients. The different tannin-enriched and non-enriched foods were submitted to *in vitro* digestion and fermentation by the gut microbiota of healthy subjects. The profile of the short chain fatty acids (SCFAs) produced by the microbiota was also investigated. The presence of tannin extracts in food promoted an increase of the relative abundance of the genus *Akkermansia*, recognized as a marker of a healthy gut, and of various members of the Lachnospiraceae and Ruminococcaceae families, involved in SCFA production. The enrichment of foods with tannin extracts had a booster effect on the production of SCFAs, without altering the profile given by the foods alone. These preliminary results suggest a positive modulation of the gut microbiota with potential benefits for human health through the enrichment of foods with tannin extracts.

## Introduction

In the last decades, the human microbiota has been the focus of great attention. Considering the genome of the entire microbial ecosystem, we can count a 100-fold higher number of genes compared to the human genome (Gilbert et al., 2018). The microbiome is involved in the modulation of human health, playing a role in several pathologies (i.e., metabolic diseases, inflammation, and neurological disorders; Ding et al., 2019). The human gut microbiota composition and function can be influenced by several factors. Among others, food and dietary components play a critical role in influencing the microbial ecosystem in the gastrointestinal tract (Laitinen and Gueimonde, 2019; Cao et al., 2020). Diet is an essential factor in determining human health status and changes in macronutrients or in diet composition can rapidly modulate the composition and the metabolic functions of the intestinal microbiota (David et al., 2014). Thus, different types of foods have been investigated to understand the different response that they induce in the gut microbiota (Aguirre et al., 2016; Wu et al., 2019).

Tannin wood extracts comprehend a large group of botanical compounds with different properties, molecular weight, and structure, which can be obtained in huge amount by natural extraction from the wood and bark of different plants. Condensed tannins and hydrolysable tannins (including gallotannins and ellagitannins) are the two main categories (Serrano et al., 2009). These bioactive compounds are polyphenolic secondary metabolites, which plants use as a defense system against different aggressions, exerting a strong antimicrobial activity against pathogenic bacteria. In view of this, tannins have been investigated for their potential as alternatives to antibiotics, in particular for livestock animals (Díaz Carrasco et al., 2018; Farha et al., 2020). On the other hand, these compounds have also been widely studied *in vitro* and *in vivo* for bioactive effects similar to those of dietary fiber (Sieniawska, 2015).

The interaction of tannins with food has been well-described in relation with several sensorial aspects, such as astringency and bitter taste (Brossaud et al., 2001; Silva et al., 2017; Li et al., 2018; Pérez-Burillo et al., 2018). The structure of tannins gives them a distinctive tendency to bind to proteins and carbohydrates (Molino et al., 2019). For instance, both tannin molecular weight and degree of galloylation enhance their affinity for proteins, probably because tannin size determines the number of interaction sites. Nevertheless, larger tannin structures can cause steric hindrance and impede access to binding sites.

As mentioned, another interesting property of tannins is related to their fiber-like behavior since they are not broken down during gastrointestinal digestion and, hence, they are not absorbed at the small intestine. Therefore, they reach the large intestine where they can be used as substrate by gut microbes, which could result in potential prebiotic properties (Molino et al., 2018).

In light of this, we wanted to investigate if the association between different tannin extracts and a particular source of food could have modulatory effects on gut microbiota composition. We studied also the production of short chain fatty acids (SCFAs), which are good indicators of the effect of a foodstuff on the intestinal microbiota.

## Materials and Methods

### Reagents

The reagents used for the *in vitro* digestion and fermentation were: potassium di-hydrogen phosphate, potassium chloride, magnesium chloride hexahydrate, sodium chloride, calcium chloride dihydrate, sodium mono-hydrogen carbonate, ammonium carbonate, and hydrochloric acid, all purchased from Sigma-Aldrich (Germany). The enzymes (salivary alpha-amylase and porcine pepsin) and bile acids (porcine bile extract) were obtained from Sigma-Aldrich, and porcine pancreatin was from Alfa Aesar (United Kingdom). The fermentation reagents (sodium di-hydrogen phosphate, sodium sulphide, tryptone, cysteine, and resazurin) were obtained from Sigma-Aldrich (Germany).

Short chain fatty acids standards (acetic acid, propionic acid, and butyric acid) were purchased from Sigma-Aldrich (Germany).

### Plant Material

Three natural tannin extracts were chosen, which are representatives for the three main categories of tannins: quebracho wood extract (QUE; rich in a profisetinidin condensed tannin), chestnut wood extract (CHE; principally characterized by the presence of hydrolysable ellagitannins), and tara pods extract (TE; mainly represented by hydrolysable gallotannins). The tannin content in the extracts, determined by the International Organization of Vine and Wine (OIV) method (Aurand, 2017), was 80% for QUE, 72% for CHE, and 92% for TE. All the extracts were purchased from Silvateam Spa (San Michele di Mondoví, Italia), as powder. The extraction methods were all food grade, characterized by a natural hot water extraction.

### Sample Preparation

Eight different food items, representing three types of products with different compositions in macronutrients were tested as potential tannin carriers: cereal-based foods [breakfast cereals (C), breakfast cereals with sugar (CS), and bread (B)], meat [meat (M) and meat with 30% fat (MF)], and dairy products [milk (L), low fat yogurt (Y), and full fat Greek yogurt (YG)]. All commercial products were bought in local supermarkets (Granada, Spain). The food samples were ground using an Ultraturrax (model T25, IKA, Spain) at 13,000 rpm. Then, the products were supplemented or not with 0.6% w/w of different tannin extracts (QUE, CHE, and TE). This amount of tannin extract added to food was decided based on our previous studies (data not shown). Since tannins can exert antimicrobial activity, it was critical to find a concentration effective for our purposes but not high enough as to affect negatively the gut microbes. On the other hand, we should use an amount of extracts that does not alter the flavor or the structure of the food matrix.

**Figure 1:**
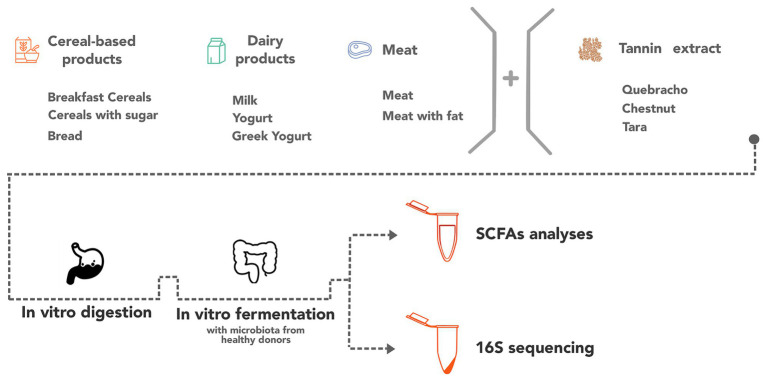
Representation of the study design.

The samples were aliquoted and stored at −80°C until the *in vitro* digestion and fermentation processes. Figure 1 summarizes the study design.

### *In vitro* Digestion-Fermentation

Samples were *in vitro* digested and fermented following the method described by Pérez-Burillo et al. (2018). Briefly, three steps mimic those of gastrointestinal human digestion. The oral phase was carried out by adding 5 ml of simulated salivary fluid with α-amylase (75 U/ml) and 25 μl of CaCl_2_ (0.3 M) to 5 g of food item alone or enriched with a tannin wood extract. The samples were then incubated for 2 min at 37°C. For the gastric phase, 10 ml of simulated gastric fluid with pepsin (2000 U/ml) and 5 μl of CaCl_2_ (0.3 M) were added and the pH was lowered to 3.0 by adding 1 N HCl. The mix was then incubated at 37°C for 2 h. Finally, 20 ml of simulated intestinal fluid with pancreatin (13.37 mg/ml), bile salts (10 mM), and 40 μl of CaCl_2_ (0.3 M) were added to the tube and the pH was raised to 7.0 with 1 N NaOH. The intestinal phase was followed by an incubation of the samples at 37°C for 2 h. A subsequent immersion of the tubes in iced water stopped the enzymatic reactions. The supernatants (potentially absorbed solution) were separated from the solid residue trough a centrifugation at 5000 rpm for 10 min at 4°C. To simulate the fraction that is not readily absorbed after digestion, 10% of the supernatant was added to the solid residue.

The fermentation was performed by adding 500 mg of the digested wet-solid residues deriving from digestion to 7.5 ml of fermentation final solution (peptone water + resazurine) and 2 ml of inoculum (32% feces w/v in phosphate buffer 100 mM, pH 7.0). We used an oligotrophic fermentation medium so that the food/tannin combination to be tested would be the only source of energy and nutrients for the microbiota. The inoculum was obtained by mixing the feces of six selected healthy volunteers, adult individuals (men or women) with a body mass index within the range 18.5–25 (University of Granada Ethics Committee approval no 1080/CEIH/2020). The selected people were not consuming any antibiotic or drug, and were following similar dietary patterns rich in vegetables, fruits, olive oil, and fish, typical of the Mediterranean diet.

In the tubes, an anaerobic atmosphere was produced by bubbling nitrogen through the mix, followed by an incubation at 37°C for 20 h, under oscillation. Immediately afterward, the samples were immersed in ice, to stop microbial activity, and centrifuged at 5,000 rpm for 10 min. The supernatant was collected as a soluble fraction potentially absorbed after fermentation and stored at −80°C, until the SCFA analyses. The solid residue, representing the non-absorbed fraction after fermentation, was also stored in order to perform 16S rRNA amplicon sequencing analyses (Figure 1).

### DNA Extraction

Genomic DNA was extracted from the solid residues deriving from the fermentation process using the MagNA Pure LC JE379 platform (Roche) and DNA Isolation Kit III (Bacteria, Fungi; REF 03264785001), following the manufacturer’s instructions, with a previous lysis with lysozyme at a final concentration of 0.1 mg/ml. DNA integrity was determined by agarose gel electrophoresis (0.8% w/v agarose in Tris-acetate-EDTA buffer) and DNA samples were quantified using a Qubit 3·0 Fluorometer (Invitrogen). All DNA samples were stored at −80°C until further processing.

### High-Throughput Amplicon Sequencing

Total DNA (12 ng) was used as template for the amplification of the V3-V4 hypervariable region of the 16S rRNA gene. PCR primers were used as described by Klindworth et al. (2013), using the forward primer (5'-TCGT CGGC AGCG TCAG ATGT GTAT AAGA GACA GCCT ACGG GNGG CWGCA-G3') and reverse primer (5'-GTCT CGTG GGCT CGGA GATG TGTA TAAG AGAC AGGA CTAC HVGG GTAT CTAA TCC3'). We followed for the library construction the Illumina protocol for the small subunit ribosomal RNA gene (16S rRNA) Metagenomic Sequencing Library Preparation (Cod 15044223 RevA). Primers were fitted with adapter sequences added to the gene-specific sequences to make them compatible with the Illumina Nextera XT Index kit. Then, the amplicons were sequenced in an Illumina MiSeq sequencer according to the manufacturer’s instructions in a 2 × 300 cycles paired-end run (MiSeq Reagent kit v3). The data for the present study were deposited in the European Nucleotide Archive (ENA) at EMBL-EBI under accession number PRJEB41013.^1^

### Bioinformatic Analyses

The sequence processing, assembly, amplicon sequence variants (ASVs) generation and annotation were performed in the DADA2 (v1.8.0) package from R (v3.6.0; Callahan et al., 2016). The filter and trimming parameters used were the following: maxN = 0, maxEE = c(2,5), truncQ = 0, trimLeft = c(17,21), truncLen = c(270,220), and rm.phix = TRUE. The merging process of the forward and reverse reads required a minimum overlap of 15 nucleotides and a maximum mismatch of 1. The reads were aligned using Bowtie2 against the human genome (GRCh38.p11) and matches were subsequently discarded (Langmead and Salzberg, 2012). The ASVs were generated by clustering sequences with 100% similarity. Taxonomic annotation was assigned by comparison to the SILVA 132 reference database using DADA2 v. 1.12 (Quast et al., 2013). Annotation was assigned at species level for 100% similarity matches or at the deepest possible taxonomic level in other cases.

### SCFA Analysis

Short chain fatty acids production was assessed by analyzing acetic, propionic, and butyric acids, according to the procedure described by Molino et al. (2018). After the fermentation process, 1 ml of supernatant from the fermentation was centrifuged to remove solid particles, filtered through a 0.22 μm nylon filter, and finally transferred to a vial for UV-HPLC analysis. The sample did not require any pre-treatment before injection. The results were expressed as mmol of SCFAs per ml of fermented soluble fraction.

### Statistical Analysis

Amplicon sequence variants with less than 10 counts in total were discarded. The ASV count table was normalized by total-sum scaling (TSS). Alpha and beta diversity measures were computed using various packages in the R platform. The Shannon diversity index, Chao1, and ACE richness estimators and Bray-Curtis dissimilarity index were obtained with the Vegan library (v2.5-2; Oksanen et al., 2012). Phylogeny-based measures such as Faith’s phylogenetic diversity (PD) and the weighted UniFrac distance were computed using the picante (v1.8.2; Kembel et al., 2010) and GuniFrac packages (v1.1; Chen et al., 2012), respectively, after sequence alignment with msa (v1.4.3; Bodenhofer et al., 2015) and UPGMA tree-buiding with phangorn (v2.5.5; Schliep, 2011). In addition, principal component analysis (PCA), principal coordinate analysis (PCoA), and heatmaps were generated with in-house R scripts. Wilcoxon signed-rank tests with false discovery rate (FDR) adjustment for multiple comparisons were employed to evaluate differences in richness, diversity, and relative abundance of taxa among samples. The linear discriminant analysis (LDA) effect size (LEfSe) algorithm was applied to identify taxonomical biomarkers from different tannins (Segata et al., 2011). It combines Kruskal-Wallis and pairwise Wilcoxon rank-sum tests for statistical significance assessment and feature selection. Default parameters were used for significance (*p* < 0.05) and linear discriminant analysis threshold (<2.0).

Results of SCFA production are expressed as mean values of triplicates (*n* = 3) ± SD. One-way ANOVA with Bonferroni post-test correction was performed with the SPSS software (version 23, SPSS, Chicago, IL, United States) to determine significant differences among mean values on all the measured parameters.

## Results

To assess the bioactivity of tannin wood extracts of different origin and chemical composition, we chose three extracts representative of different classes of tannins: condensed tannins (QUE), ellagitannins (CHE), and gallotannins (TE). We evaluated the effect on the gut microbiota by adding the extracts to eight different sources of food grouped in three food types: cereal-based foods [breakfast cereals (C), breakfast cereals with sugar (CS), and bread (B)], meat [meat (M) and meat with 30% fat (MF)], and dairy products [milk (L), low fat yogurt (Y), and full fat Greek yogurt (YG)]. All the samples were subjected to an *in vitro* digestion-fermentation process designed to mimic natural digestion in the human oral, gastric, and intestinal chambers. The bioactivity was measured as the capacity to modify the gut microbiota in terms of taxonomic composition and SCFA production.

### Comparisons of Microbiota Among Different Samples

**Figure 2:**
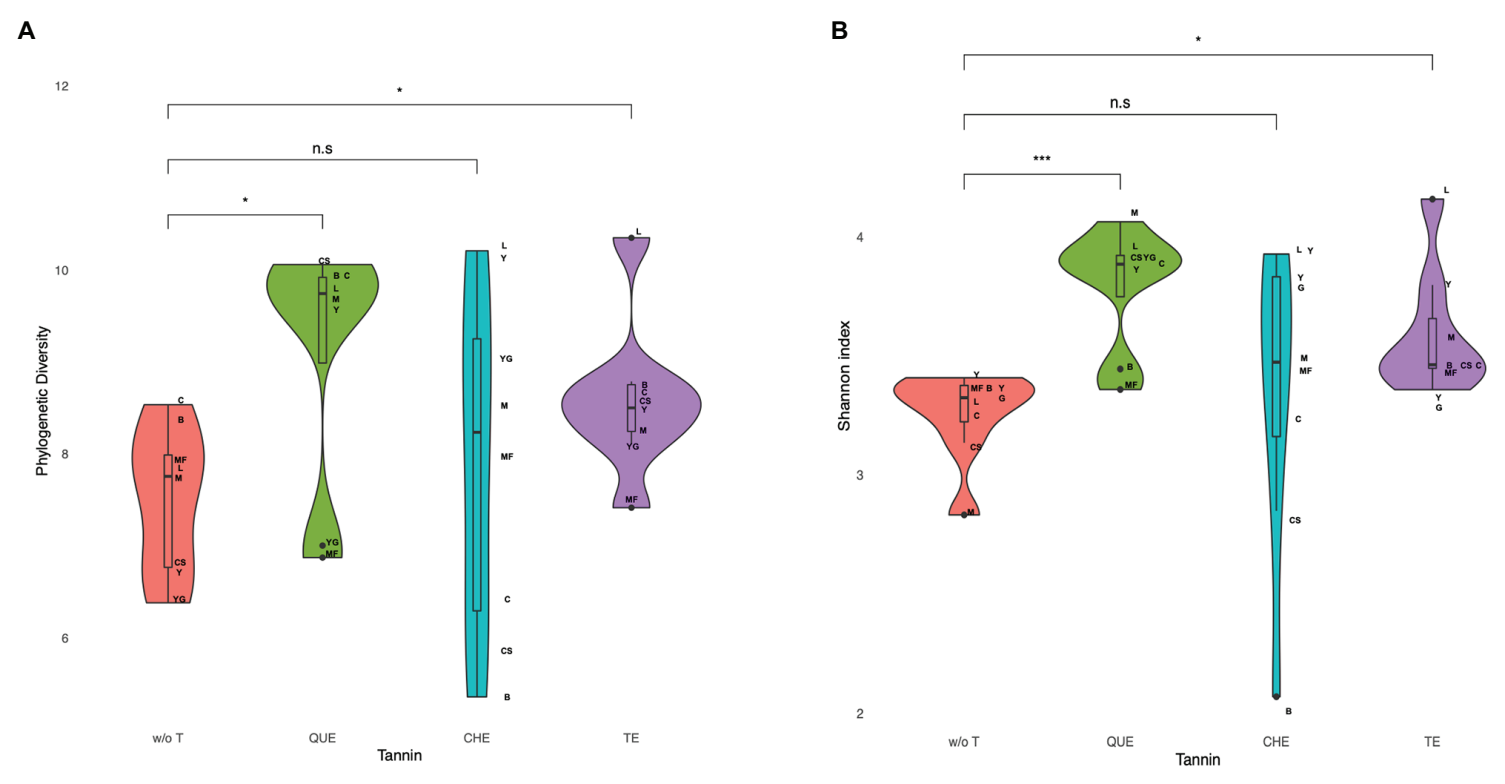
Microbiota diversity measured as **(A)** phylogenetic diversity and **(B)** Shannon index in fermentations of food matrices with and without tannin extracts. Diversity increases for quebracho wood extract (QUE) and tara pods extract (TE) when all food matrices are considered together (*p* = 0.016, adjusted *p* = 0.023, and *p* = 0.008, adjusted *p* = 0.023, respectively). w/o T, without tannins; QUE, quebracho tannins extract; CHE, chestnut tannins extract; TE, tara tannins extract.

The addition of tannin to the different food matrices determined a general trend of increase of the sample richness and diversity, as evaluated by different estimators and indexes: the Chao1 and ACE richness estimators, Faith’s phylogenetic diversity (PD) and the Shannon diversity index that takes into account both richness and evenness (Supplementary Table S1). As an exception, microbiota richness and diversity rather decreased in cereal-based foods supplemented with CHE. Figure 2 shows the increase in diversity detected when all food matrices are considered together for QUE (PD: adjusted *p* = 0.035; Shannon: adjusted *p* = 0.023) and TE (PD: adjusted *p* = 0.035; Shannon: adjusted *p* = 0.023), and the larger variability observed with the addition of CHE.

**Figure 3:**
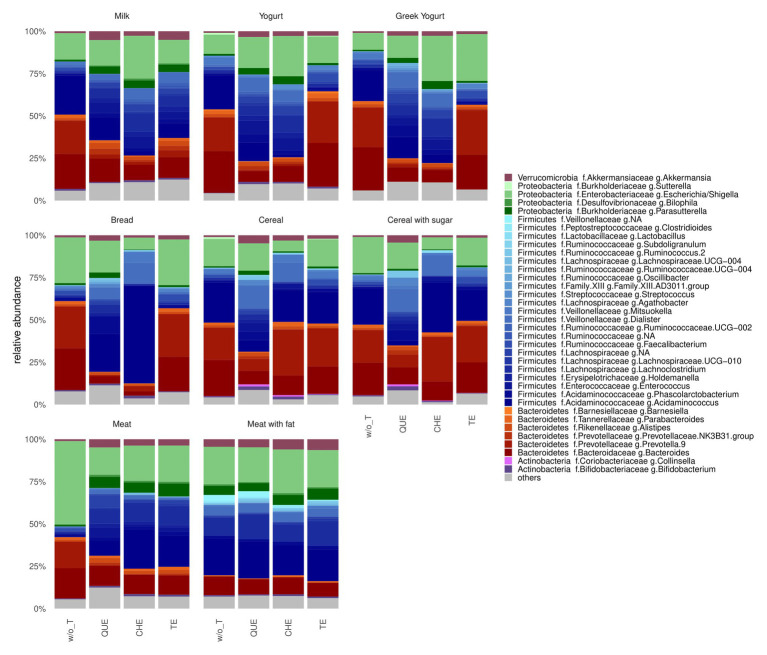
Barplot of gut microbial community structure at genus level. w/o T, without tannins; QUE, quebracho tannins extract; CHE, chestnut tannins extract; TE, tara tannins extract. Relative abundance obtained by total-sum scaling (TSS) from genus-level abundance table. “Others” include genera with relative abundance lower than 1% for all conditions.

At the phylum level, the gut microbiota after *in vitro* fermentation of most samples was similar and dominated by Firmicutes and Bacteroidetes, followed by Proteobacteria, Verrucomicrobia and Actinobacteria, with some exceptions (Figure 3; Supplementary Table S1). Indeed, for meat (M and MF) enriched with tannin extracts, the relative abundance of Proteobacteria was higher (*p* = 0.00084, adjusted *p* = 0.0051), while that of Bacteroidetes was lower (*p* = 0.027, adjusted *p* = 0.053) compared to meat samples not enriched with tannins.

**Figure 4:**
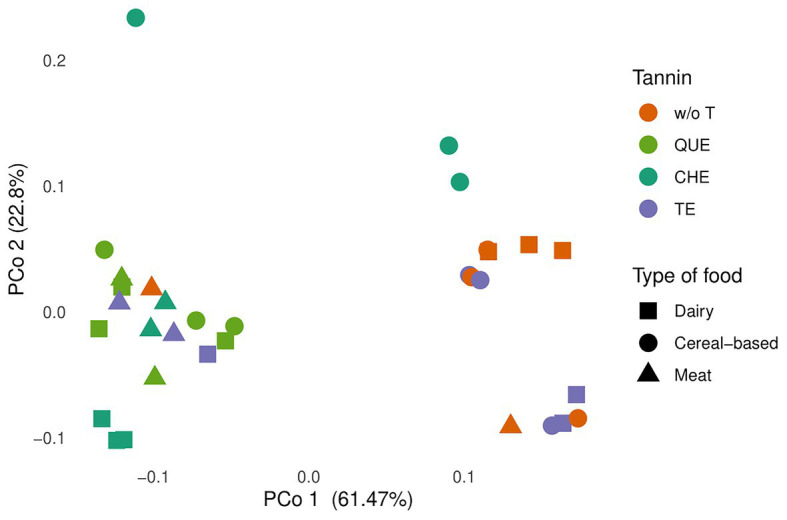
Principal coordinate analysis (PCoA) plot of total variation based on weighted UniFrac distances among microbial communities in all profiled samples, evaluated at genus level. The food sources were grouped in three food types: cereal-based foods (breakfast cereals, breakfast cereals with sugar, and bread), meat (meat and meat with 30% fat), and dairy products (milk, low fat yogurt, and full-fat Greek yogurt). w/o T, without tannins, QUE, quebracho tannins extract; CHE, chestnut tannins extract; TE, tara tannins extract.

Principal coordinate analyses based on the Bray-Curtis dissimilarity index and the weighted UniFrac distance were performed to establish whether samples separate into clusters. As seen in Figure 4, PCo1 and PCo2 accounted for 61.47 and 22.8%, respectively, of the total variation based on weighted UniFrac distances. All samples containing QUE and CHE clustered away from non-enriched foods, as did the milk and meat samples enriched with TE. The PCoA based on the Bray-Curtis index provided very similar results (Supplementary Figure S1). Thus, the results indicate that in most cases, the microbiota communities resulting from foods enriched with these tannin extracts are different from those of the food matrices alone.

More in depth, the obtained pattern of sample clustering does not reflect exclusively the tannin or the associated food matrix, but is rather influenced by both. Among the evaluated tannin extracts, QUE clustered most homogeneously, with all samples present in a single cluster independently of the matrix to which QUE was associated. In contrast, for CHE and TE, microbial composition depended on the food matrix. In the case of CHE, although all samples separated well from non-enriched foods, dairy and meat samples clustered with the QUE samples, whereas cereal-based foods did not. In the case of TE, the yogurt and cereal-based samples did not separate from non-enriched foods, whereas the milk and meat samples were located in the cluster containing all the QUE samples and the dairy and meat CHE samples. This suggests that TE only has an effect on the gut microbiota when added to meat or milk.

**Figure 5:**
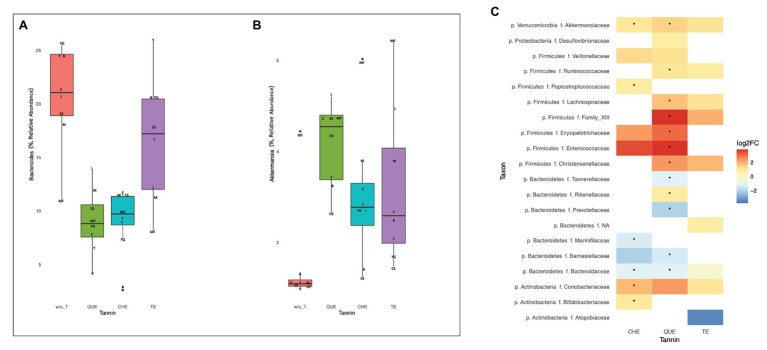
Changes in microbiota composition with tannin addition in all foods. **(A)** Decrease of *Bacteroides* (QUE: *p* = 0.0078, adjusted *p* = 0.048; CHE: *p* = 0.0078, adjusted *p* = 0.11; TE: *p* = 0.023, adjusted *p* = 0.14) and **(B)** increase of *Akkermansia* (QUE: *p* = 0.0078, adjusted *p* = 0.048; CHE: *p* = 0.0078, adjusted *p* = 0.11; TE: *p* = 0.0078, adjusted *p* = 0.079) with every tannin tested. Initials indicate the different food matrices (C, breakfast cereals; CS, breakfast cereals with sugar; B, bread; M, meat; MF, meat with 30% fat; L, milk; Y, low fat yogurt; and YG, full-fat Greek yogurt). **(C)** Fold-changes in the relative abundance of bacterial families with each tannin. All changes significant at nominal level by Wilcoxon signed-rank tests are shown (*p* < 0.05). ^*^Indicates statistically significant differences (*p* < 0.05) after correction for multiple testing.

To identify the bacteria most generally affected by the addition of each of the tannins, we applied Wilcoxon signed-rank tests to compare bacterial relative abundances in all samples enriched with a given tannin vs. all samples containing no tannins. These comparisons identified a large number of genera for which abundance after tannin addition differed from that of non-enriched foods at nominal significance level, but only differences in QUE were still significant after adjusting for multiple comparisons (Supplementary Figure S2). Remarkably, with every tannin tested *Bacteroides* decreased (QUE: *p* = 0.008, adjusted *p* = 0.048; CHE: *p* = 0.008, adjusted *p* = 0.11; TE: *p* = 0.023, adjusted *p* = 0.14; Figure 5A) while *Akkermansia* increased (QUE: *p* = 0.008, adjusted *p* = 0.048; CHE: *p* = 0.008, adjusted *p* = 0.11; TE: *p* = 0.008, adjusted *p* = 0.079; Figure 5B). Wilcoxon signed-rank tests at family level (Figure 5C) confirmed that Bacteroidaceae decreased (QUE: *p* = 0.008, adjusted *p* = 0.031; CHE: *p* = 0.008, adjusted *p* = 0.049; TE *p* = 0.023, adjusted *p* = 0.103) and Akkermansiaceae increased (CHE: *p* = 0.008, adjusted *p* = 0.048; QUE: *p* = 0.008, adjusted *p* = 0.031; TE *p* = 0.008, adjusted *p* = 0.068) with each of the tannins, although, in this case, only the differences with QUE and CHE were significant after adjustment for multiple comparisons. In addition, several abundance changes were significant only with QUE, including decreases of the Bacteroidales families Prevotellaceae (adjusted *p* = 0.031), Barnesiellaceae (adjusted *p* = 0.031), and Tannerellaceae (adjusted *p* = 0.031), as well as increases of the Rickenellaceae (Bacteroidales; adjusted *p* = 0.047) and of several Clostridiales families [Ruminococcaceae (adjusted *p* = 0.031), Lachnospiraceae (adjusted *p* = 0.031), Christensenellaceae (adjusted *p* = 0.047), and Family XIII (adjusted *p* = 0.031)]. On the other hand, Peptostreptococcaceae (Clostridiales; adjusted *p* = 0.049) and the actinobacterial families Coriobacteriaceae (adjusted *p* = 0.048) and Bifidobacteriaceae (adjusted *p* = 0.048) augmented significantly only with CHE.

**Figure 6:**
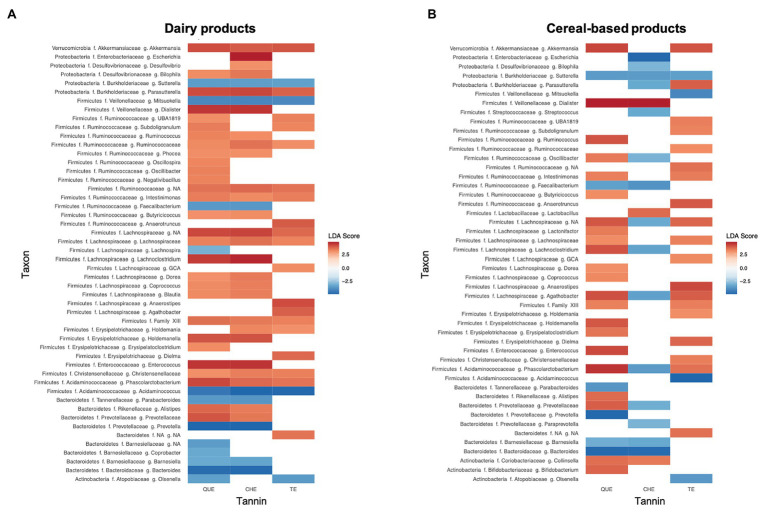
Genera responsible for the main differences in gut microbiota composition due to the addition of tannin extracts to **(A)** dairy products and **(B)** cereal-based products, detected by Linear Discriminant Analysis (LDA) Effect Size (LEfSe). Differences are represented as LDA score (>3) by color gradient. All represented biomarkers are significant at *p* < 0.05. NA taxon refers to unclassified ASVs at f. (family) or g. (genus) level.

We also applied LEfSe analyses to identify the specific biomarkers that best characterize the microbiota changes generated by the addition of tannins within each of the three food groups [cereal-based foods (C, CS, and B), meat (M and MF), and dairy products (L, Y, and YG)]. The only group where significant differences were not detected between tannin-enriched and non-enriched matrices was that of meats. Figure 6 depicts the over- and underrepresented genera (LDA score >3 and *p* < 0.05) in dairy sources and cereal-based foods enriched with the different tannin extracts, QUE, CHE, and TE. In most cases, genera increased rather than decreased with the addition of tannins, except in the case of CHE added to cereal-based foods, where several members of the Proteobacteria, Bacteroidetes, and Firmicutes were found to decrease, including various genera of the Ruminococcaceae and Lachnospiraceae families. *Sutterella* was the only genus that decreased in both food matrices with all of the tannins tested. No genus was overrepresented in both food matrices with all of the tannins, but *Akkermansia*, *Intestinimonas*, *Phascolarctobacterium*, and various unassigned Lachnospiraceae and Clostridiales Family XIII genera did increase in all cases except when cereal-based foods were enriched with CHE. It is interesting to note that several of the genera that decreased when CHE was added to carbohydrates actually increased when CHE was added to dairy (*Escherichia*, *Bilophila*, or *Lachnoclostridium*).

Finally, the differences generated by the addition of each tannin extract to each individual food matrix are represented as fold changes in Supplementary Figure S3, although further repetitions of each fermentation would be needed to assess their significance.

### Production of SCFAs

**Figure 7:**
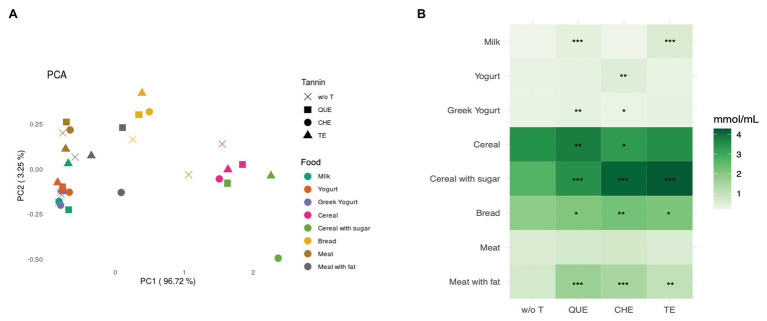
Short chain fatty acids (SCFAs) released after *in vitro* fermentation of the food matrices with and without tannins. **(A)** Principal component analysis (PCA) based on Euclidean distance. **(B)** Sum of the concentration of SCFAs (in mmol/ml) per each sample, represented by color gradient as shown next to the heatmap. w/o T, without tannins; QUE, quebracho tannins extract; CHE, chestnut tannins extract; TE, tara tannins extract. ^*^Indicates statistically significant differences by ANOVA and Bonferroni *post-hoc* test: ^*^p < 0.05, ^**^p < 0.01, ^***^p < 0.001.

Short chain fatty acids are metabolites produced by gut microbial fermentation of carbohydrates, dietary fibers, proteins, and resistant starch. We focused on the release of the three principal subtypes: acetate, propionate, and butyrate. The PCA in Figure 7A depicts the similarity of the samples based on the relative abundance of each SCFA, showing that SCFA production is similar in fermentations of the same food group independently of the presence of the tannin extracts.

As expected, cereal-based foods released the highest amount of SCFAs (Figure 7B), producing, in decreasing order, acetic acid, propionic acid, and butyric acid. In foods with a higher protein content, the ratio between acetic and propionic acid decreased, or even reversed, as in the case of M (Table 1). In most cases, the addition of tannins did not alter the relative production of the different SCFAs, but resulted in a booster effect (Table 1). Looking at the production of total SCFAs (Figure 7B), QUE, CHE, and TE nearly always resulted in a significant increase, particularly when they were combined with CS (QUE: adjusted *p* < 0.001, CHE: adjusted *p* < 0.001, and TE: adjusted *p* < 0.001) and MF (QUE: adjusted *p* < 0.001, CHE: adjusted *p* < 0.001, and TE: adjusted *p* = 0.002). On the contrary, when added to M, none of the three tannin extracts produced a statistically significant increase of total SCFAs. However, the addition of QUE, CHE, or TE to M induced a significant increase in the production of acetic acid compared to M without tannins, suggesting that they were exerting a booster effect on specific acetate-producing bacteria (Table 1).

**Table 1:** Short chain fatty acids produced after the fermentation of the food source enriched or not with tannin extracts.

		Acetic acid	Propionic acid	Butyric acid
Milk	w/o T	0.117 ± 0.006	0.055 ± 0.000	0.047 ± 0.001
	QUE	0.269 ± 0.020^∗^	0.063 ± 0.021	0.077 ± 0.000^∗^
	CHE	0.120 ± 0.001	0.060 ± 0.001^∗^	0.042 ± 0.004
	TE	0.174 ± 0.004^∗^	0.302 ± 0.002^∗^	0.107 ± 0.024^∗^
Yogurt	w/o T	0.124 ± 0.009	0.115 ± 0.000	0.081 ± 0.008
	QUE	0.138 ± 0.003	0.149 ± 0.000^∗^	0.091 ± 0.001
	CHE	0.244 ± 0.013^∗^	0.158 ± 0.006^∗^	0.098 ± 0.001^∗^
	TE	0.067 ± 0.019^∗^	0.151 ± 0.001^∗^	0.092 ± 0.003
Greek yogurt	w/o T	0.139 ± 0.008	0.104 ± 0.001	0.088 ± 0.002
	QUE	0.152 ± 0.020	0.133 ± 0.010^∗^	0.092 ± 0.000
	CHE	0.151 ± 0.002	0.047 ± 0.002^∗^	0.087 ± 0.000
	TE	0.133 ± 0.006	0.133 ± 0.010^∗^	0.090 ± 0.001
Cereals	w/o T	2.232 ± 0.033	1.181 ± 0.010	0.059 ± 0.000
	QUE	2.545 ± 0.027^∗^	1.173 ± 0.009	0.068 ± 0.003
	CHE	2.259 ± 0.010	0.985 ± 0.038^∗^	0.042 ± 0.005^∗^
	TE	2.359 ± 0.009^∗^	1.076 ± 0.011^∗^	0.064 ± 0.001
Cereals with sugar	w/o T	1.832 ± 0.002	0.853 ± 0.006	0.016 ± 0.001
	QUE	2.379 ± 0.023^∗^	1.002 ± 0.029^∗^	0.075 ± 0.003^∗^
	CHE	3.214 ± 0.018^∗^	0.865 ± 0.008	0.047 ± 0.001^∗^
	TE	2.957 ± 0.028^∗^	1.259 ± 0.004^∗^	0.054 ± 0.003^∗^
Bread	w/o T	1.006 ± 0.005	0.754 ± 0.012	0.093 ± 0.010
	QUE	1.043 ± 0.022	0.912 ± 0.022^∗^	0.122 ± 0.002^∗^
	CHE	1.177 ± 0.025^∗^	0.980 ± 0.013^∗^	0.112 ± 0.003
	TE	1.041 ± 0.046	1.043 ± 0.001^∗^	0.060 ± 0.002^∗^
Meat	w/o T	0.039 ± 0.001	0.434 ± 0.026	0.104 ± 0.005
	QUE	0.068 ± 0.000^∗^	0.510 ± 0.014^∗^	0.115 ± 0.002
	CHE	0.134 ± 0.003^∗^	0.488 ± 0.033	0.088 ± 0.000
	TE	0.110 ± 0.003^∗^	0.367 ± 0.038^∗^	0.093 ± 0.005
Meat with 30% fat	w/o T	0.255 ± 0.004	0.372 ± 0.019	0.069 ± 0.000
	QUE	0.842 ± 0.015^∗^	0.765 ± 0.029^∗^	0.074 ± 0.001
	CHE	0.951 ± 0.025^∗^	0.419 ± 0.017^∗^	0.075 ± 0.002
	TE	0.473 ± 0.002^∗^	0.460 ± 0.021^∗^	0.089 ± 0.001^∗^

The results are expressed as mmol/ml and data are represented as means ± SD (*n* = 3). w/o T, without tannins; QUE, quebracho tannins extract; CHE, chestnut tannins extract; TE, tara tannins extract.

∗ Indicates statistically significant differences (*p* < 0.05) by ANOVA and Bonferroni *post-hoc* test between foods enriched with tannins and not.

Regarding differences among the three tannins, we detected several cases where only one or two of the extracts were effective at boosting overall SCFA production when added to specific foods (Figure 7B). Notably, within dairy foods, QUE was able to boost SCFA production in M and YG, whereas CHE and TE were only able to do so in Y and M, respectively. The measurements of specific SCFAs (Table 1) indicate that CHE and TE were able to boost the production of all three SCFAs in Y and M, respectively, whereas the other tannin extracts were not. QUE, on the other hand, had a significant booster effect on acetic and butyric acids in M, and on propionic acid in YG. This differential effect on specific SCFAs was, however, not sufficient to alter the overall profile of SCFA production in these foods, as observed in Figure 7A.

## Discussion

### Effects on Microbiota Composition

The gut microbiota has a determinant role in maintaining human health. Specific foods and products containing prebiotics interact directly or indirectly with the microbiota, driving its composition and function (Laitinen and Gueimonde, 2019). In this context, tannins can modulate gut microbial composition and function, selectively inhibiting pathogens and promoting the growth of beneficial bacteria (Ozdal et al., 2016). Tannins are known also for their capacity to interact with proteins and carbohydrates, among other compounds. So, we were interested in investigating if the association of tannin extracts from different sources with different types of food matrices could determine a different response when interacting with the microbiota of healthy people. In particular, we decided to study tannins extracted from wood and bark of different plants, which are now finding new relevant applications in the food sector (Molino et al., 2019).

For most food matrices and tannins tested, tannin enrichment resulted in a change in the composition of the microbiota, compared with the fermentations employing the foods alone (Figure 4). Besides the increase in overall richness and diversity, the increase of the genus *Akkermansia* was one of the most general results in our study (Figure 5B). These are mucin-degrading bacteria, living in the mucus layer, recognized as markers of a healthy gut. In fact, several studies have highlighted their anti-inflammatory properties, and the ability to increase insulin sensitivity, and boost gut barrier function (Masumoto et al., 2016; Cires et al., 2017; Rinninella et al., 2019). These properties are also related to their production of propionate and butyrate (Venegas et al., 2019). Furthermore, some authors have proposed *Akkermansia* as a key player in the breakdown of phenolic compounds in the intestine (Li et al., 2015), suggesting a likely reason for the observed increase of this genus with the addition of tannins.

The addition of tannins (in particular QUE and CHE) also determined a decrease of *Bacteroides* and other genera and families of the order Bacteroidales (Figure 5A). *Bacteroides* is known to be implicated in proteolytic fermentation in the gut, so that the binding of proteins by tannins may be responsible for the decrease of these bacteria by rendering protein molecules unavailable for digestion (Smith and MacFarlane, 1998). Inhibitory effects of tannins on proteolytic bacteria and proteolytic enzyme activity have been proposed, likely due to coating of the protein surface leading to interference with the interaction of enzyme and substrate (McManus et al., 1981; Patra et al., 2012).

The interactions between tannins and food macromolecules may be different depending on the food matrices. Binding mechanisms between tannins and proteins or carbohydrates occur in a specific and selective way, mediated by hydrogen bonds and hydrophobic interactions (McManus et al., 1985; de Freitas and Nuno, 2012). Some factors related to these macromolecules may influence such interactions: size, charge, side chains, and conformation (Molino et al., 2019). Therefore, we expected that some effects of tannins would be dependent on the food matrix to which they were added.

In fact, we detected increases of the families Lachnospiraceae and Ruminococcaceae in most of the tannin-food combinations analyzed, except for the case of cereal-based foods supplemented with CHE (Figure 6). Several authors have reported an increase of the abundance of bacteria belonging to the Lachnospiraceae and Ruminococcaceae families when tannins were added to animal feed (Choy et al., 2014; Díaz Carrasco et al., 2018). Lachnospiraceae and Ruminococcaceae belong to the order Clostridiales, which encompasses mostly beneficial bacteria, including members that have been associated with the modulation of physiologic, metabolic, and immune processes in the gut and with prevention of inflammatory bowel disease. Furthermore, numerous genera of these families have been attributed the capacity to produce SCFAs (Lopetuso et al., 2013; Louis and Flint, 2017). Díaz Carrasco et al. (2018) observed the same trend of increment of the aforementioned Clostridiales families in chicken supplemented with a mix of QUE and CHE. In particular, they detected an increment of the genus *Faecalibacterium*, among others. In our analyses, *Faecalibacterium* rather decreased with addition of QUE and CHE to cereal-based and dairy products (Figure 6). However, addition of TE did determine a large increase of *Faecalibacterium* in C, CS, Y, and YG (Supplementary Figure S2), although *Faecalibacterium* increases did not reach significance when all cereal-based or dairy foods were considered together. This finding suggests that it will be interesting to analyze these individual food matrices supplemented with TE in further experiments to assess the significance of the *Faecalibacterium* increases independently in each of them.

Besides not showing an increase in Lachnospiraceae and Ruminococcaceae (Figure 6), cereal-based foods supplemented with CHE clustered far from the other samples (Figure 4) and, unlike other tannin-food combinations, showed a decrease in microbiota richness and diversity (Supplementary Table S1). These results could be related to a specific interaction between the hydrolysable ellagitannins contained in the chestnut wood extract and the fiber present in the evaluated food matrices. The inhibitory effects of ellagitannins on the activity of carbohydrate digestive enzymes, reported in the systematic review of Prpa et al. (2020), could also contribute to explain these differences.

### Effects on SCFA Production

Tannins can act locally at intestinal level (especially non-absorbable tannins of high molecular weight), reaching the colonic gut microbiota. Herein, these compounds could be used by microorganisms, resulting in metabolites with different bioavailability, activity, or functional effect compared to the parent molecule (Serrano et al., 2009).

Tannins have been proposed as prebiotic substrates for gut microbes as these molecules favor SCFA production, have growth-promoting effects for beneficial bacteria, and/or could activate their metabolic functions. In our previous studies, we demonstrated a great production of SCFAs following *in vitro* digestion and fermentation of QUE and CHE (Molino et al., 2018). Now, our aim was to investigate whether the association of these extracts to various food sources could result in a booster effect or in an altered production of different SCFAs from that observed with the food sources alone.

It is well-known that carbohydrates exert a considerable prebiotic effect because polysaccharides act as a nutrient for the gut microbiota (Aguirre et al., 2016). Their metabolization by the microbiota results in the production, in decreasing order of proportion, of acetate, propionate, and butyrate. Generally, proteins are not a good source of prebiotics and they alter the profile of SCFA production. Increased fermentation of amino acids results in an elevated production of propionate, together with branched SCFAs, and some potentially harmful molecules such as amines or hydrogen disulfide (Diether and Willing, 2019). Here, we show that the combination of tannin extracts (QUE, CHE, and TE) with the food matrices resulted in most cases in an increment of total SCFA production (Figure 7B). The increased SCFA production in presence of tannins may be potentially related to the synthesis of these compounds using the tannins or their metabolized products as substrates, or to the increased relative abundance and/or activity of gut microbiota species that ferment carbohydrates. Indeed, the increment in the relative abundances of both potential tannin metabolizers such as *Akkermansia* and carbohydrate fermenters/SCFA producers such as the Ruminococcaceae and Lachnospiraceae families suggests that both processes may be contributing to the boost in SCFA production.

The only case where none of the tannins led to any significant booster effect on total SCFA production was when combined with M. The strong protein-binding capacity is one of the distinguishing properties of tannins. This may have affected the potential interaction of the newly formed tannin-protein complexes with the microbiota, probably making the proteins less digestible and the tannins less available to exert their prebiotic action. The presence of a higher percentage of fat in MF may have reduced the formation of complexes between meat proteins and tannins, thus allowing greater interaction between tannins and the microbiota, resulting in a greater production of SCFAs.

Finally, our analyses revealed some differences in the modulation of SCFA production among the three tannin extracts (Figure 7B; Table 1). QUE (a condensed tannin), CHE (an ellagitannin), and TE (a gallotannin) have different chemical structures and it is well-known that the interaction between macromolecules and the different classes of tannins could differ (McManus et al., 1985; Baxter et al., 1997; Carn et al., 2012). However, although in some foods different tannins favored the synthesis of different SCFAs, this differential effect did not change the relative proportions of the SCFAs sufficiently to alter the expected profile favored by each food matrix. As illustrated in Figure 7A, the separation of the different samples according to SCFA production profile depended on the food sources (dairy products, meat, and cereal-based foods). So, the factor driving the clustering of SCFA profiles was the different nutritional composition of the food sources, not the tannin enrichment. It is possible that longer fermentation times might be needed to obtain significant differences in the effect of different tannins on the overall profile of SCFA production.

Our study may suffer some limitations related to the *in vitro* digestion and fermentation system. Indeed, some bacteria could need longer times for growth, so that the length of the fermentation experiment would not be sufficient to observe an increment of their relative abundance and/or their metabolic activity reflected in the production of SCFAs. An *in vivo* study could be more reliable because factors such as the interaction between the tannins and the gastrointestinal system could come into play. Furthermore, a long-term interventional study could allow investigating gradual changes in the composition of the intestinal microbiota and its functions. On the other hand, an *in vivo* study could introduce many confounding variables resulting in a disadvantage for the study of the interaction of tannins with specific food matrices.

In conclusion, the enrichment of foods with different composition (dairy products, cereal-based foods, and meat) with tannin extracts (QUE, CHE, and TE) evidenced the potential to influence host physiology through the modulation of the composition and the functionality of symbiotic bacteria in the gut. Our findings are in line with the considerations of Cires et al. (2017), which associated the consumption of condensed tannins with a shift to a healthier environment in the colonic ecosystem that could play a key role in the digestion and absorption of nutrients, but also in protecting the gastrointestinal system against pathogens. These preliminary results will pave the ground for the development of new functional foods within the framework of the European Commission research project Stance4Health. Indeed, these extracts, which are produced in a sustainable way, could represent new and cost-effective natural supplements to be exploited for their prebiotic effect, acting by boosting the production of SCFAs, which are crucial in the maintenance of gut and immune homeostasis. Depending on the matrix to which they were added, the new products could determine a different modulation of the composition and activity of the gut microbiota. In particular, QUE extracts generate the most homogeneous response across foods, whereas the effects of CHE and TE are more highly dependent on the food matrix. These new findings on the advantageous effects of tannins, and on the versatility provided by their combination with different foods, make of these compounds a promising new tool for the promotion of a healthy gut environment, with potential long-term benefits for metabolism and immunity.

## Data Availability Statement

The sequence data are available in the European Nucleotide Archive (ENA) under accession number PRJEB14013 (https://www.ebi.ac.uk/ena/browser/view/PRJEB41013).

## Author Contributions

SM, JAR-H, and MPF designed the research. SM, AL-A, and NJ-H conducted the experiments. SM and AL-A analyzed data and performed statistical analyses. AL-A conducted the bioinformatic analyses. SM and MPF wrote the manuscript. MJG, AL-A, and JAR-H provided significant advice and critically edited the manuscript. JAR-H obtained funding and coordinated the Stance4Health project. All authors contributed to the article and approved the submitted version.

### Conflict of Interest

The authors declare that the research was conducted in the absence of any commercial or financial relationships that could be construed as a potential conflict of interest.

## Footnotes

^1^https://www.ebi.ac.uk/ena/browser/view/PRJEB41013
