# Supplementary material for: Enrichment of Food With Tannin Extracts Promotes Healthy Changes in the Human Gut Microbiota
Source: Front Microbiol. 2021 Mar 16;12:625782. doi: 10.3389/fmicb.2021.625782 (PMC8008114; doi:10.3389/fmicb.2021.625782)
Supplement: Supplementary file 1 [file Data_Sheet_1.pdf]

Supplementary Material

Enrichment of food with tannin extracts promotes healthy changes in the human gut microbiota

Silvia Molino[1†], Alberto Lerma-Aguilera[2†], Nuria Jiménez-Hernández[2,3], María José Gosalbes[2,3*], José Ángel Rufián Henares[1,4*], M. Pilar Francino[2,3*]

[1]Departamento de Nutrición y Bromatología, Instituto de Nutrición y Tecnología de los Alimentos, Centro de Investigación Biomédica, Universidad de Granada, Granada, Spain

[2]Area de Genòmica i Salut, Fundació per al Foment de la Investigació Sanitária i Biomèdica de la Comunitat Valenciana (FISABIO-Salut Pública), València, Spain

[3] CIBER en Epidemiología y Salud Pública, Madrid, 28029, Spain

[4] Instituto de Investigación Biosanitaria ibs,Granada, Granada, Spain

[†] Equal contribution

*** Correspondence:**
M. Pilar Francino
mpfrancino@gmail.com

María José Gosalbes
Maria.Jose.Gosalbes@uv.es

José Ángel Rufián Henares
jarufian@ugr.es

1. Supplementary Table

Table S1. Indexes of α diversity and relative abundance of phyla.

		Diversity indexes						Phyla composition					
Food	Tannin	Phylogenetic diversity	Shannon	Chao1	SE.Chao1	ACE	SE.ACE	Actinobacteria	Bacterioidetes	Firmicutes	Proteobacteria	Verrucomicrobia	other
Milk	w/o T	7.79	3.29	219.00	4.13	218.25	7.03	1.11	43.87	31.33	16.77	1.14	5.79
	QUE	9.79	3.96	287.50	5.41	286.86	8.10	0.66	24.64	39.25	19.99	5.21	10.25
	CHE	10.20	3.91	302.80	4.53	303.45	8.46	1.18	14.60	39.79	30.91	2.69	10.83
	TE	10.35	4.16	305.00	4.54	304.23	8.12	0.98	23.57	38.92	19.06	5.08	12.39
Yogurt	w/o T	6.73	3.41	172.00	1.82	171.63	5.79	0.24	49.33	31.71	13.48	0.95	4.30
	QUE	9.65	3.85	269.00	4.31	267.24	7.68	1.33	12.20	51.24	22.11	3.42	9.69
	CHE	10.08	3.93	297.21	7.43	292.75	8.13	0.95	14.54	43.07	28.53	2.84	10.07
	TE	8.46	3.80	261.00	4.13	259.78	7.70	1.08	56.36	15.40	17.39	2.68	7.09
Greek yogurt	w/o T	6.38	3.35	139.00	0.12	139.17	4.94	0.00	52.78	29.43	10.83	1.03	5.93
	QUE	7.00	3.90	159.00	0.10	159.22	5.15	0.00	13.89	56.27	16.11	2.65	11.08
	CHE	8.97	3.80	227.25	0.74	227.39	6.58	0.00	11.41	43.72	31.47	2.70	10.70
	TE	8.10	3.36	216.88	1.25	218.35	6.91	0.00	50.05	12.93	28.80	1.71	6.51
Cereals	w/o T	8.53	3.25	311.17	17.56	293.07	8.37	0.78	43.36	32.49	18.07	1.08	4.21
	QUE	9.91	3.87	307.79	8.35	303.48	8.49	3.31	19.29	45.37	18.64	4.78	8.60
	CHE	6.41	3.26	167.00	4.31	166.10	6.28	2.48	43.25	40.85	7.21	3.14	3.07
	TE	8.74	3.45	271.77	10.02	264.40	7.87	0.68	41.58	32.56	17.52	2.07	5.58
Cereals with Sugar	w/o T	6.78	3.13	165.67	1.15	166.13	5.93	0.75	41.65	29.64	22.16	1.04	4.76
	QUE	10.05	3.91	325.53	7.25	328.39	8.95	3.49	23.03	44.16	16.56	4.35	8.40
	CHE	5.95	2.85	146.50	3.92	146.89	5.73	0.98	40.29	48.53	7.59	1.24	1.36
	TE	8.54	3.46	261.93	6.85	259.30	7.74	0.36	42.57	31.64	17.49	1.47	6.47
Bread	w/o T	8.28	3.38	230.40	1.62	231.95	7.47	0.84	52.53	9.43	28.17	1.30	7.73
	QUE	9.92	3.44	290.81	8.71	286.05	8.13	1.00	6.93	55.44	22.02	3.24	11.36
	CHE	5.36	2.07	108.14	0.49	108.58	4.94	1.61	7.25	78.84	7.27	1.42	3.61
	TE	8.79	3.46	263.22	3.69	264.45	7.93	0.52	49.10	12.44	28.19	2.49	7.26
Meat	w/o T	7.72	2.83	212.33	2.42	212.42	7.08	0.54	36.19	6.35	50.40	1.10	5.42
	QUE	9.69	4.06	281.77	2.68	282.23	7.65	1.02	17.80	39.98	23.99	4.84	12.36
	CHE	8.56	3.50	258.13	3.36	258.58	7.60	1.28	15.00	44.87	27.81	3.78	7.26
	TE	8.29	3.61	231.33	2.55	231.10	6.98	1.16	16.46	41.55	30.08	3.73	7.03
Meat with 30% fat	w/o T	7.89	3.37	214.06	0.28	214.52	7.13	0.85	11.71	47.49	28.44	4.44	7.07
	QUE	6.87	3.36	181.60	1.19	181.73	6.08	0.67	9.45	51.54	25.93	4.74	7.67
	CHE	7.90	3.45	231.88	3.19	233.51	7.24	1.02	11.20	41.57	32.78	6.03	7.41
	TE	7.41	3.43	203.50	3.92	203.21	6.43	0.97	9.07	48.05	29.35	6.41	6.13

2. Supplementary Figures

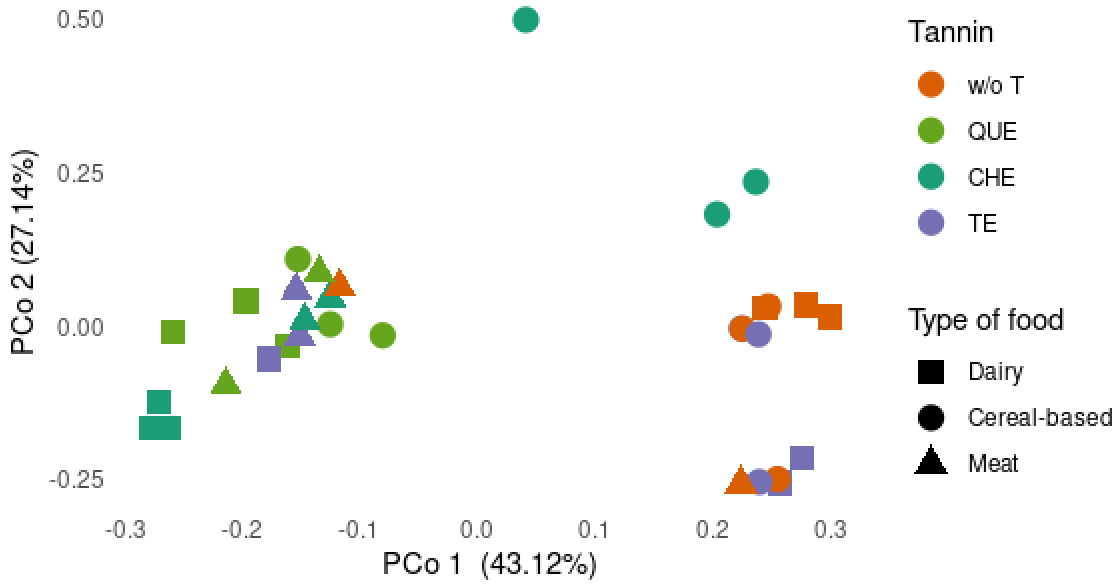

Figure S1 Principal coordinate analysis (PCoA) plot with Bray-Curtis dissimilarity of microbial genus abundance among all profiled samples. The food sources were grouped in three food types: cereal-based foods (breakfast cereals, breakfast cereals with sugar, bread), meat (meat and meat with 30% fat) and dairy products (milk, low fat yogurt and full-fat Greek yogurt). w/o T: without tannins, QUE: quebracho tannins extract, CHE: chestnut tannins extract, TE: tara tannins extract.

Figure S2 Comparisons of relative abundances of bacteria at genus level in all foods with and without tannin addition (S2A: QUE, S2B: CHE, S2C: TE). Adjusted and unadjusted p values by Wilcoxon signed-rank tests are shown. (3 pdf files uploaded separately)

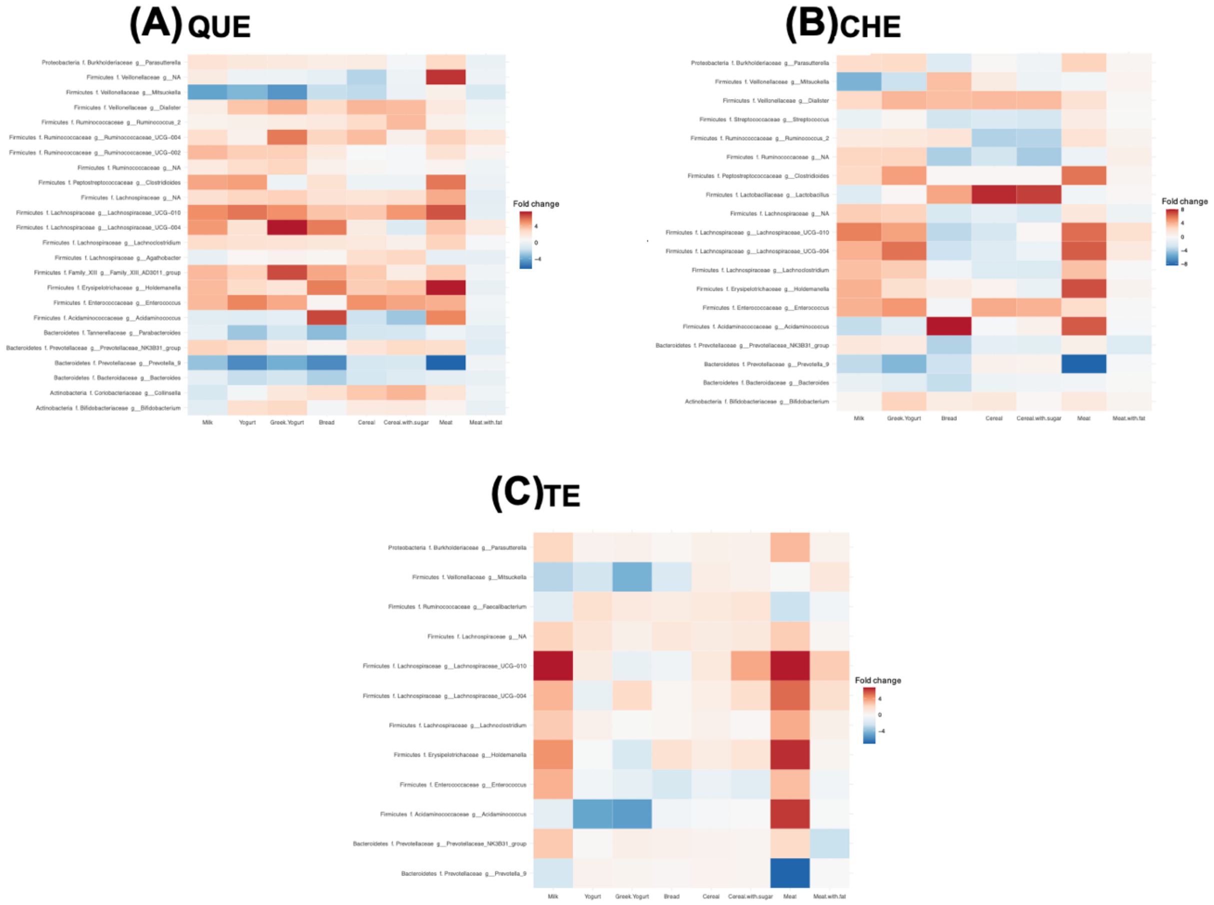

Figure S3 Heatmap of relative abundance fold changes of genera induced by the addition of tannins extract (A: QUE, B: CHE, C: TE) to the food sources. The graphs report the genera with an abundance > 1% with a fold change > 2.5. QUE: quebracho tannins extract, CHE: chestnut tannins extract, TE: tara tannins extract.
