# Supplementary material for: Enrichment of Food With Tannin Extracts Promotes Healthy Changes in the Human Gut Microbiota
Source: Front Microbiol. 2021 Mar 16;12:625782. doi: 10.3389/fmicb.2021.625782 (PMC8008114; doi:10.3389/fmicb.2021.625782)
Supplement: Supplementary file 3 [file Data_Sheet_3.pdf]

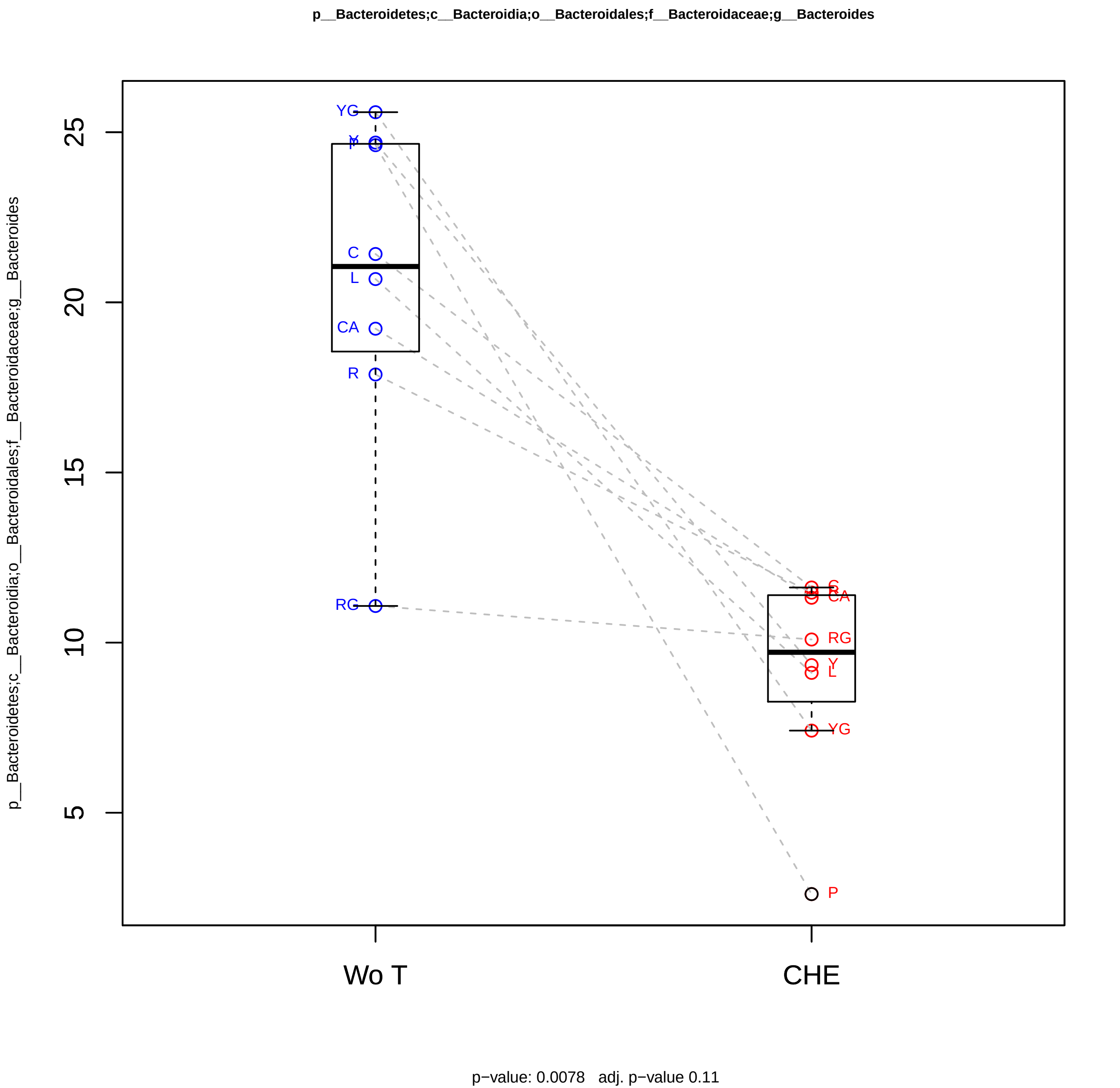
p__Bacteroidetes;c__Bacteroidia;o__Bacteroidales;f__Bacteroidaceae;g__Bacteroides
p__Bacteroidetes;c__Bacteroidia;o__Bacteroidales;f__Bacteroidaceae;g__Bacteroides
25
20
15
10
5
YG
Y
P
C
L
CA
R
RG
C
CA
RG
Y
L
YG
P
Wo T
CHE
p−value: 0.0078 adj. p−value 0.11

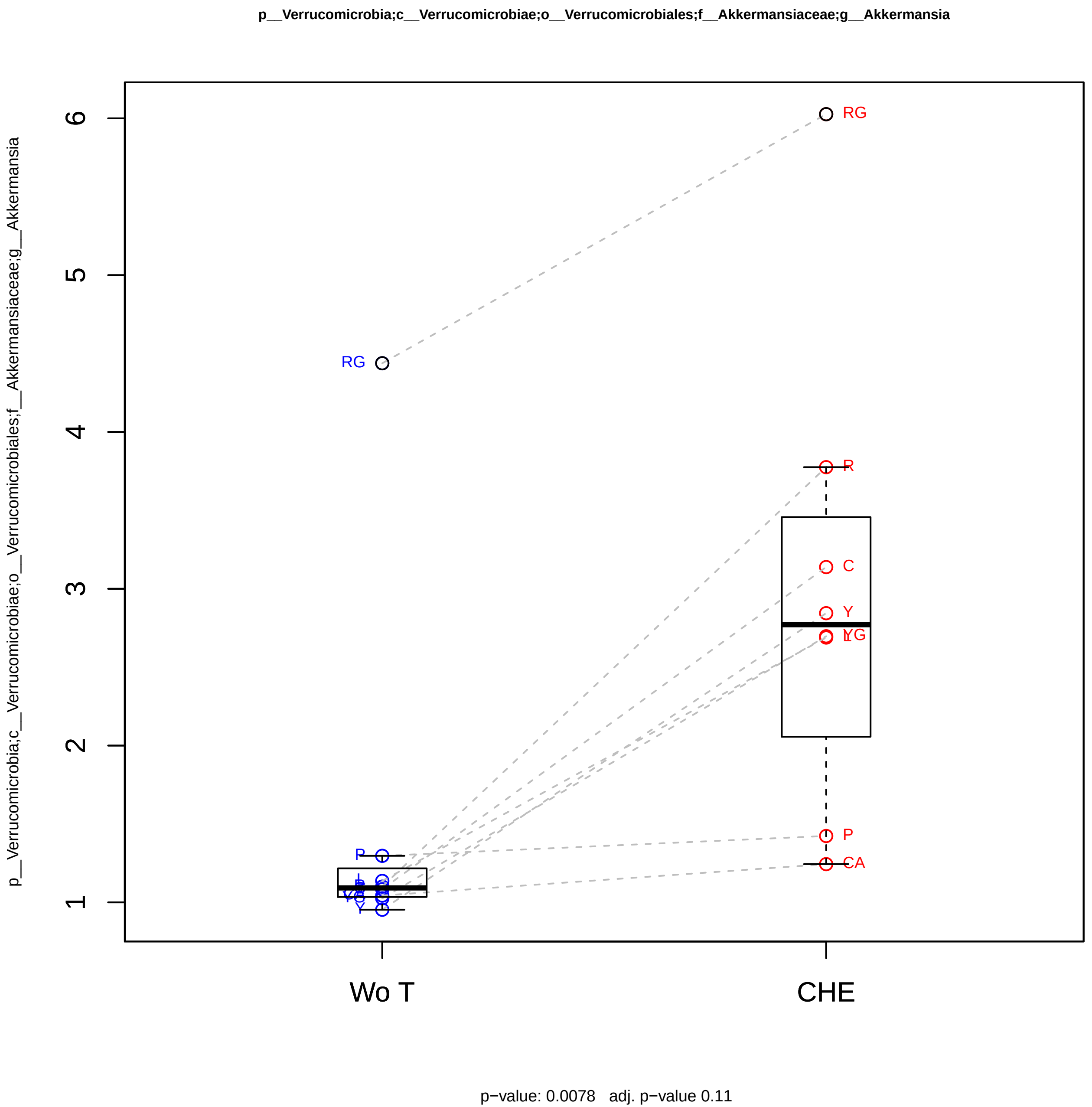
p__Verrucomicrobia;c__Verrucomicrobiae;o__Verrucomicrobiales;f__Akkermansiaceae;g__Akkermansia
p__Verrucomicrobia;c__Verrucomicrobiae;o__Verrucomicrobiales;f__Akkermansiaceae;g__Akkermansia
1
2
3
4
5
6
Wo T
CHE
RG
P
RG
R
C
Y
LG
P
CA
p−value: 0.0078 adj. p−value 0.11

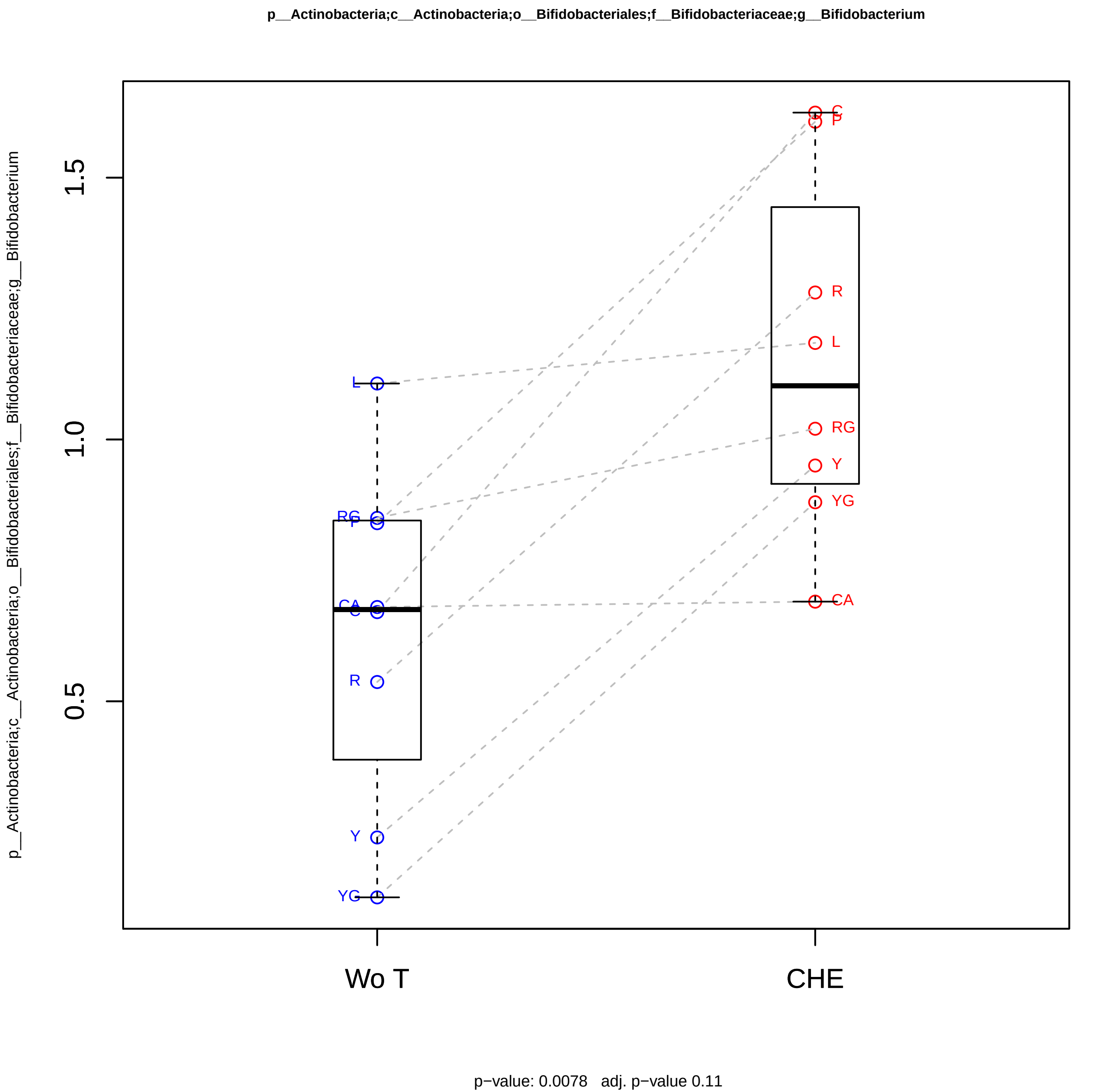
p__Actinobacteria;c__Actinobacteria;o__Bifidobacteriales;f__Bifidobacteriaceae;g__Bifidobacterium
p__Actinobacteria;c__Actinobacteria;o__Bifidobacteriales;f__Bifidobacteriaceae;g__Bifidobacterium
1.5
1.0
0.5
Wo T
CHE
L
RG
F
CA
C
R
Y
YG
C
P
R
L
RG
Y
YG
CA
p−value: 0.0078 adj. p−value 0.11

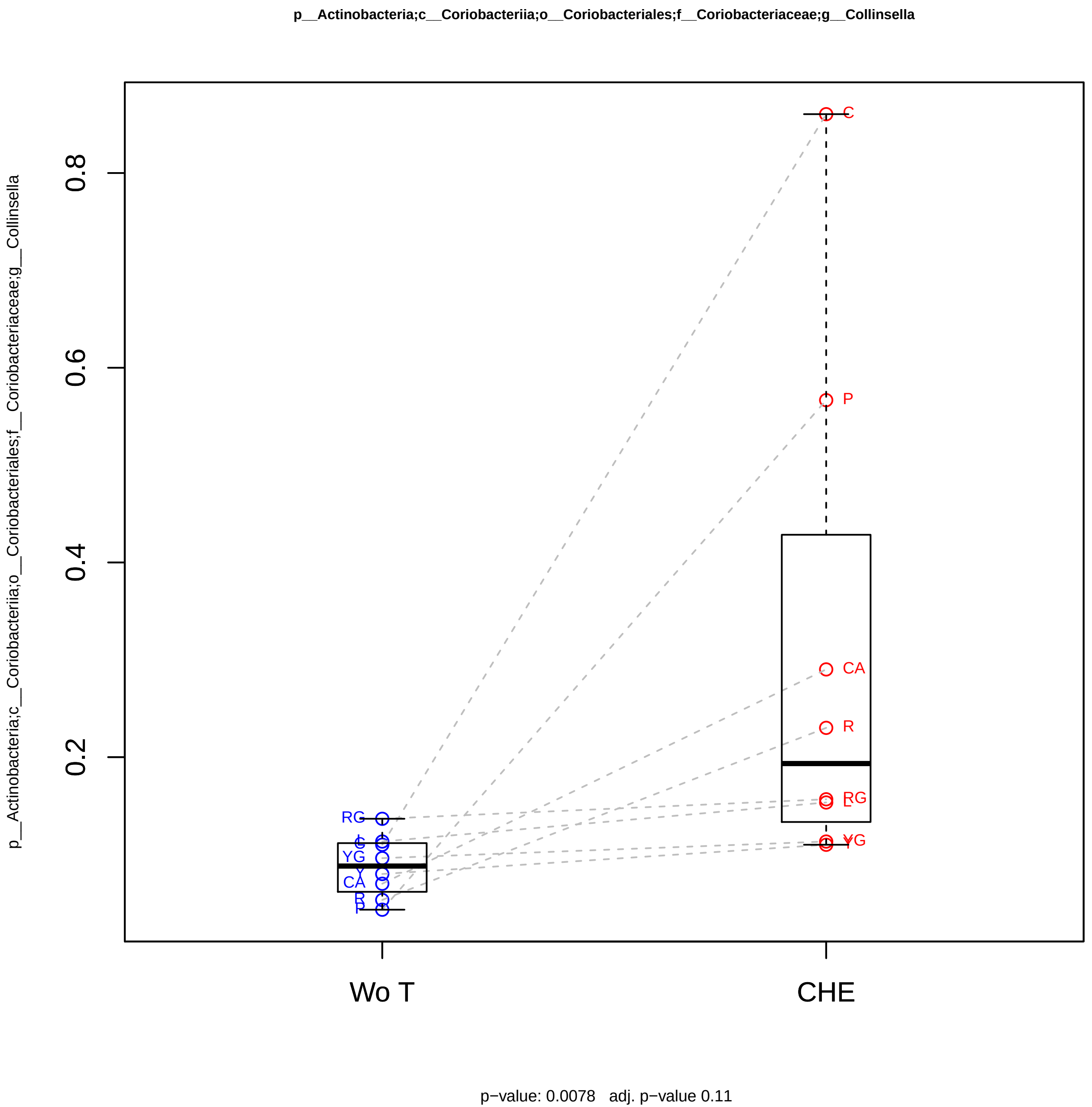
p__Actinobacteria;c__Coriobacteriia;o__Coriobacteriales;f__Coriobacteriaceae;g__Collinsella
p__Actinobacteria;c__Coriobacteriia;o__Coriobacteriales;f__Coriobacteriaceae;g__Collinsella
0.2
0.4
0.6
0.8
Wo T
CHE
RG
L
C
YG
Y
CA
R
P
C
P
CA
R
RG
L
YG
Y
p−value: 0.0078 adj. p−value 0.11

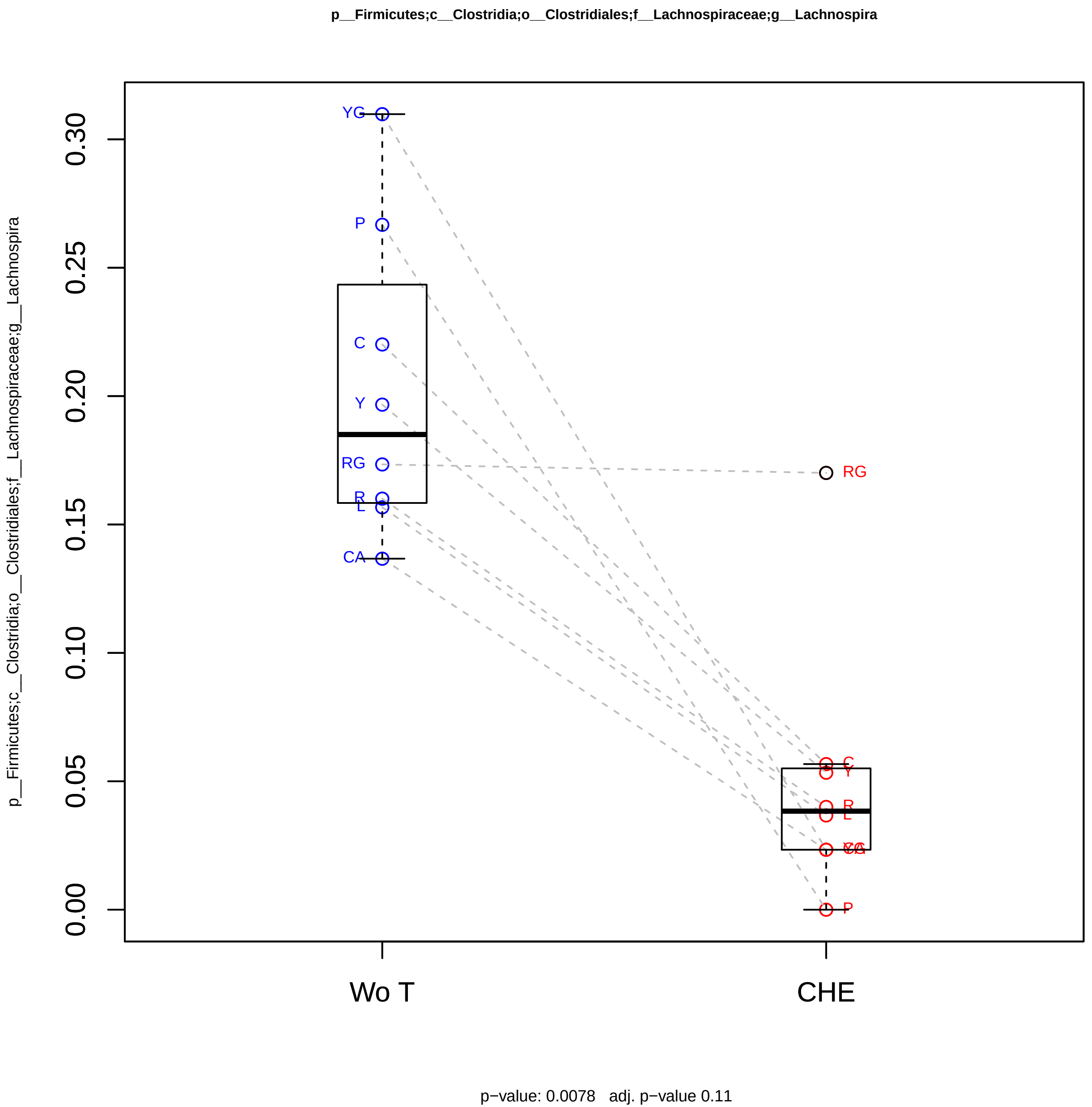

p__Firmicutes;c__Clostridia;o__Clostridiales;f__Lachnospiraceae;g__Lachnospira
p__Firmicutes;c__Clostridia;o__Clostridiales;f__Lachnospiraceae;g__Lachnospira
0.00
0.05
0.10
0.15
0.20
0.25
0.30
YG
P
C
Y
RG
R
L
CA
RG
C
Y
R
L
CA
P
Wo T
CHE
p−value: 0.0078 adj. p−value 0.11

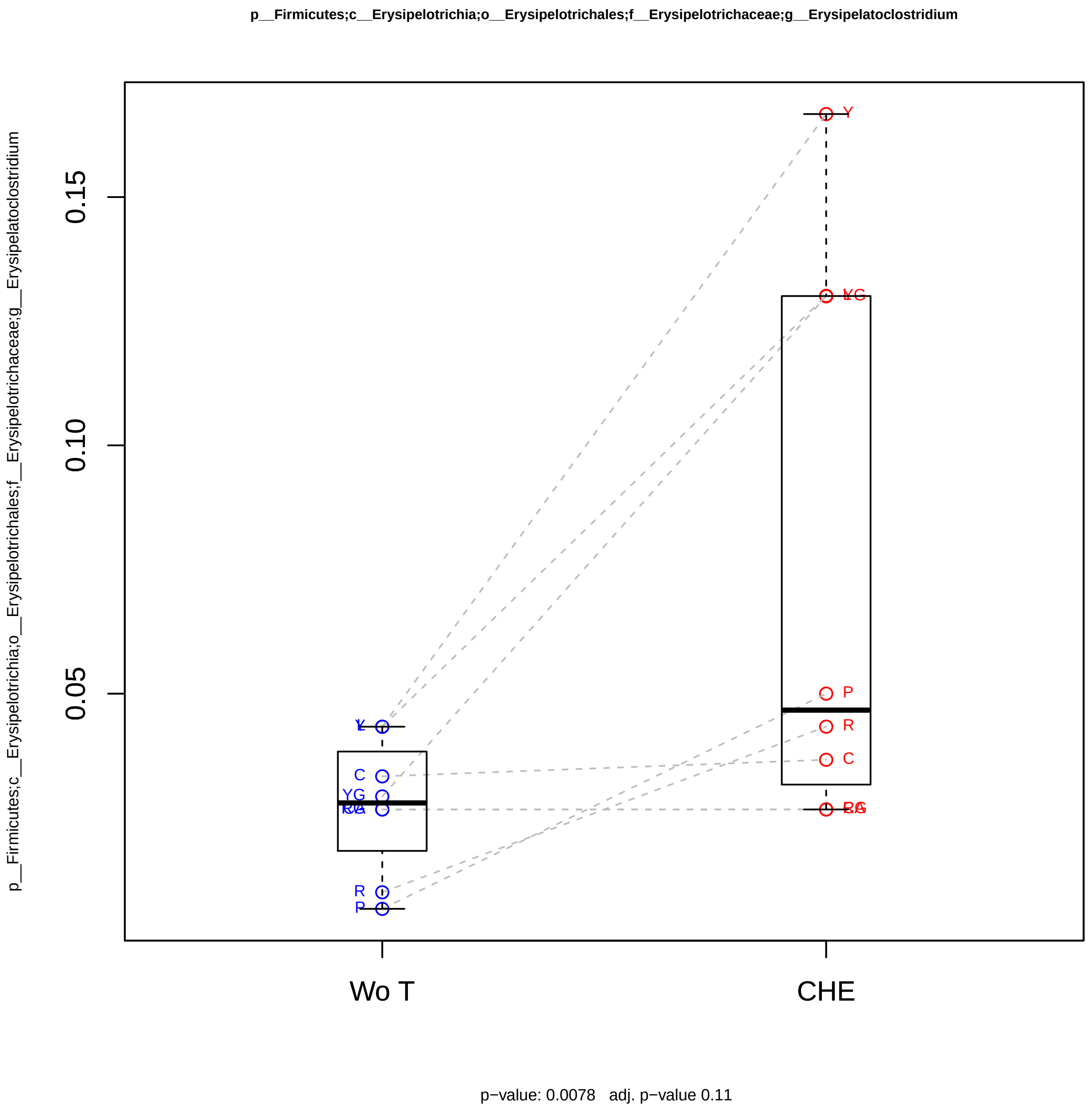

p__Firmicutes;c__Erysipelotrichia;o__Erysipelotrichales;f__Erysipelotrichaceae;g__Erysipelatoclostridium
p__Firmicutes;c__Erysipelotrichia;o__Erysipelotrichales;f__Erysipelotrichaceae;g__Erysipelatoclostridium
0.05
0.10
0.15
Wo T
CHE
Y
L
C
YG
R
P
p−value: 0.0078 adj. p−value 0.11

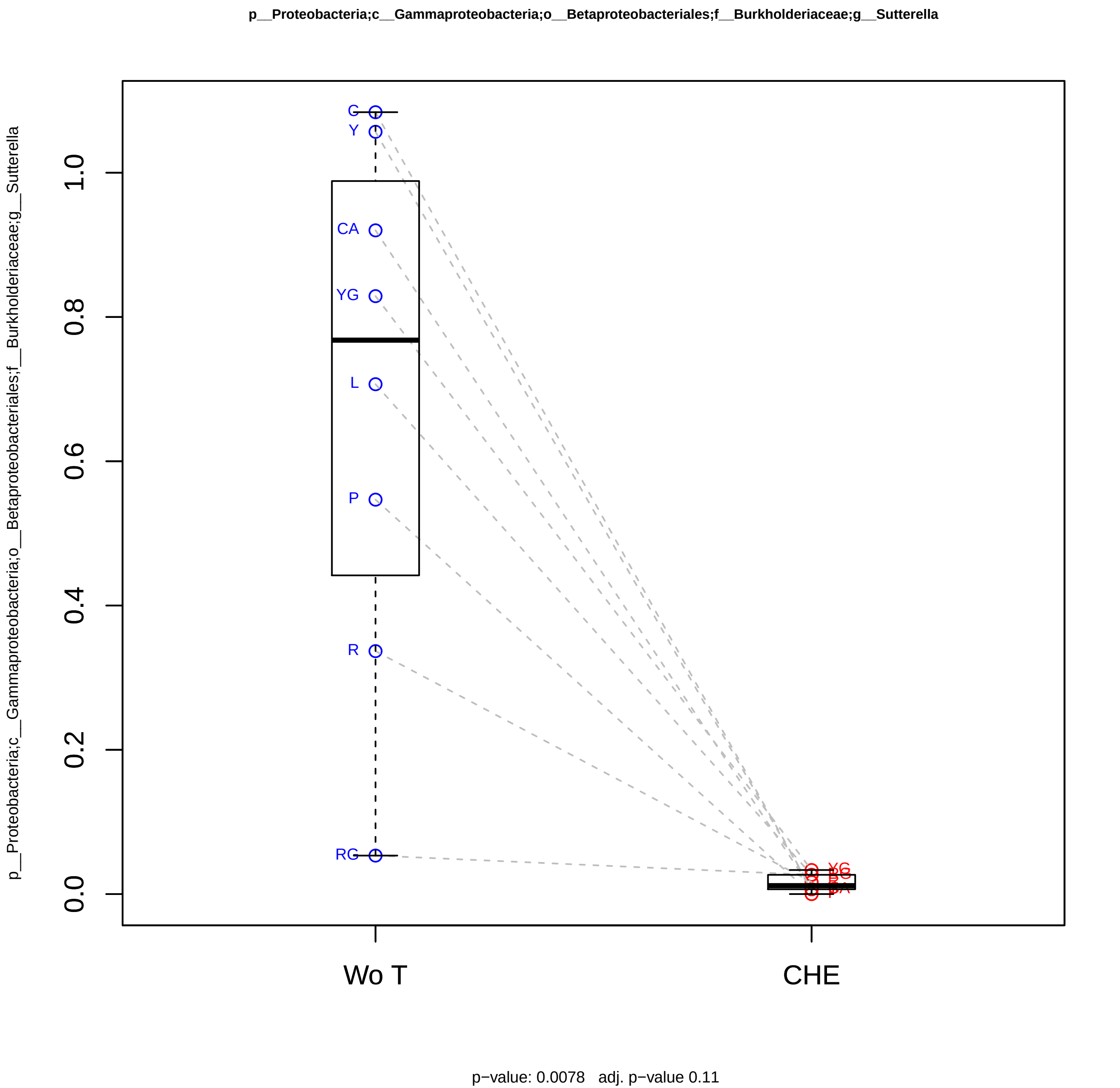
p__Proteobacteria;c__Gammaproteobacteria;o__Betaproteobacteriales;f__Burkholderiaceae;g__Sutterella
p__Proteobacteria;c__Gammaproteobacteria;o__Betaproteobacteriales;f__Burkholderiaceae;g__Sutterella
0.0
0.2
0.4
0.6
0.8
1.0
G
Y
CA
YG
L
P
R
RG
Wo T
CHE
p−value: 0.0078   adj. p−value 0.11

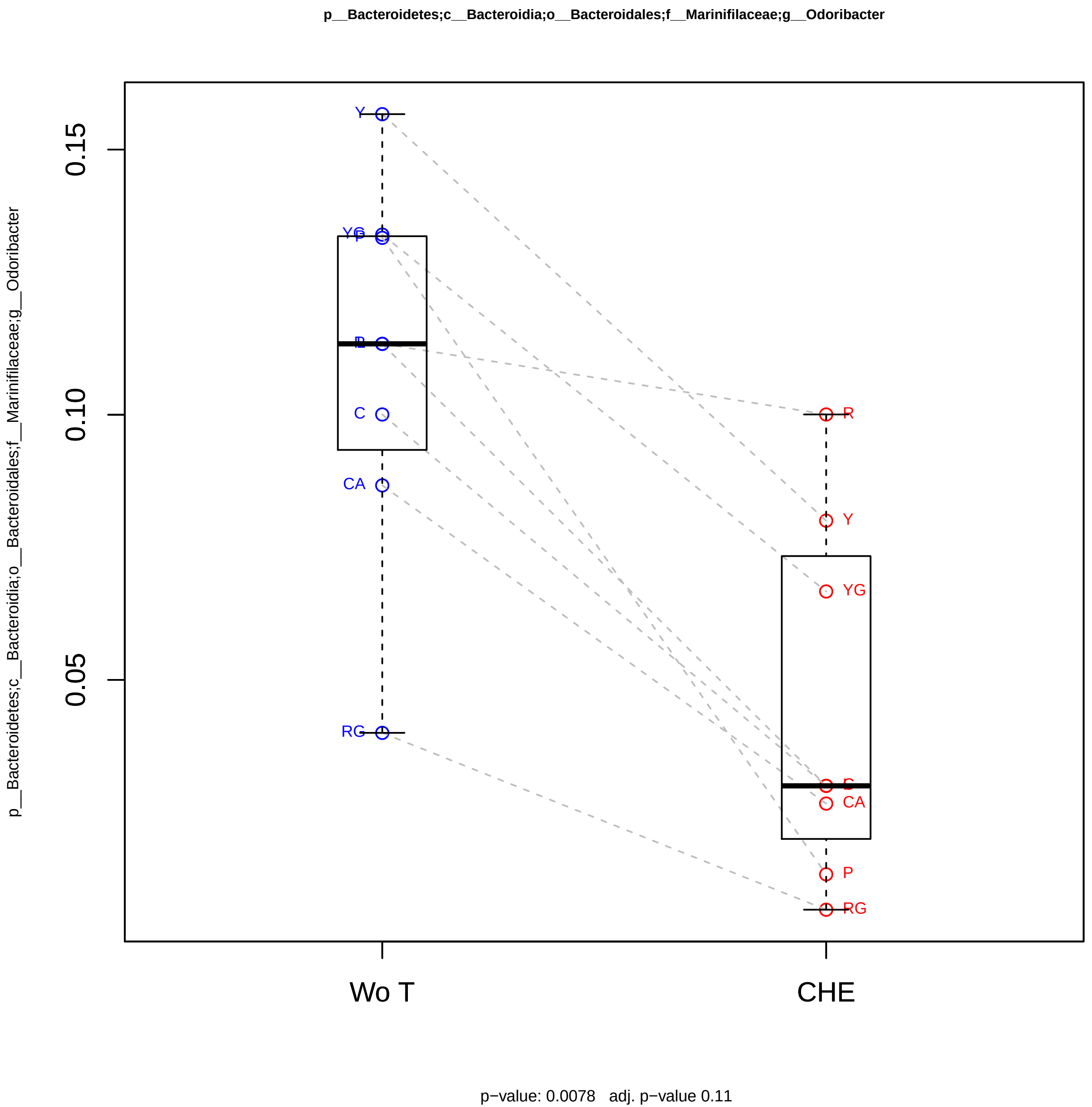

p__Bacteroidetes;c__Bacteroidia;o__Bacteroidales;f__Marinifilaceae;g__Odoribacter
p__Bacteroidetes;c__Bacteroidia;o__Bacteroidales;f__Marinifilaceae;g__Odoribacter
0.15
0.10
0.05
Y
YG
P
R
C
CA
RG
Wo T
CHE
p−value: 0.0078 adj. p−value 0.11

p__Firmicutes;c__Clostridia;o__Clostridiales;f__Lachnospiraceae;g__Hungatella

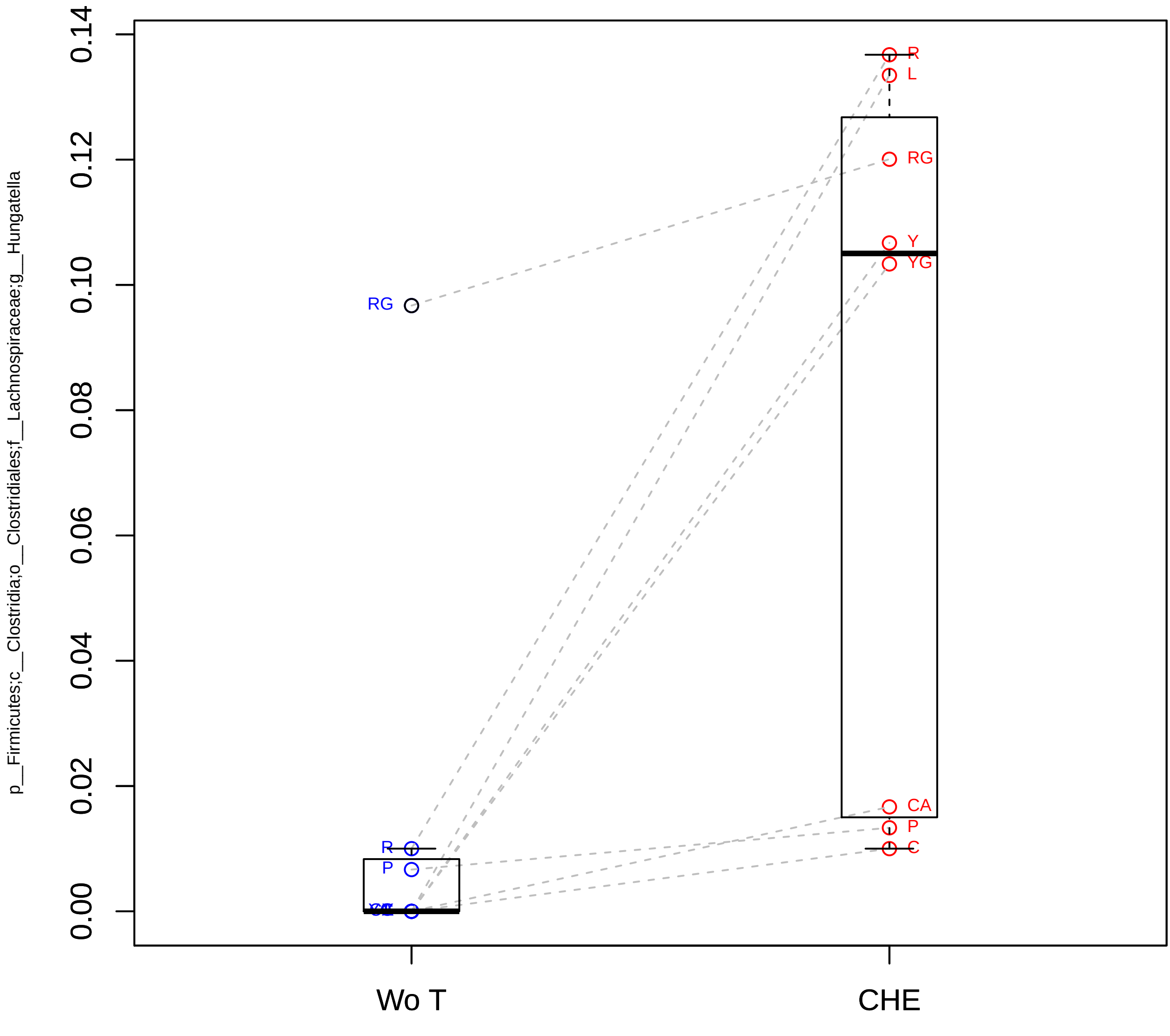


p−value: 0.0078   adj. p−value 0.11

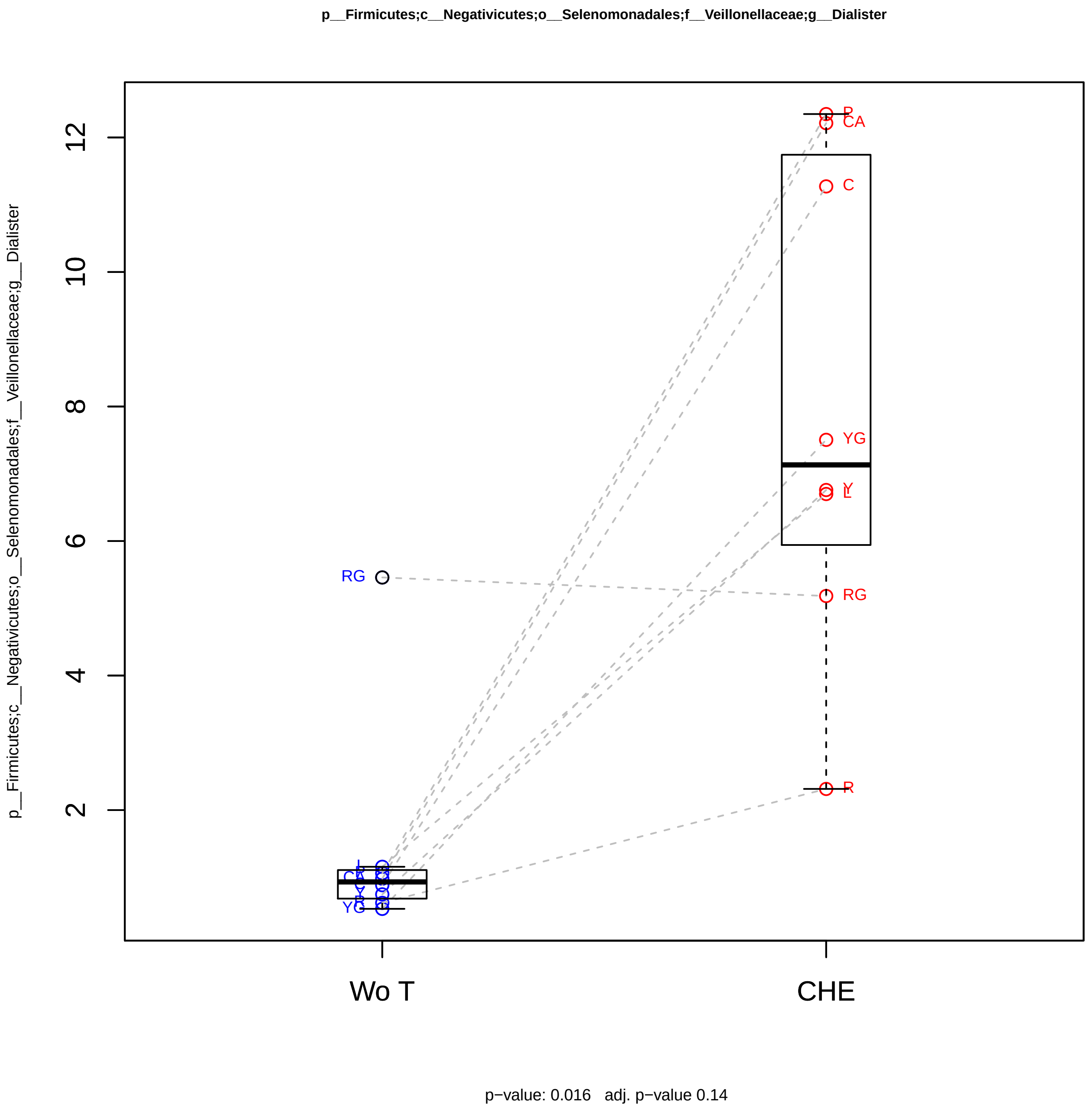
p__Firmicutes;c__Negativicutes;o__Selenomonadales;f__Veillonellaceae;g__Dialister
p__Firmicutes;c__Negativicutes;o__Selenomonadales;f__Veillonellaceae;g__Dialister
2
4
6
8
10
12
Wo T
CHE
RG
P
CA
C
YG
Y
L
R
p−value: 0.016 adj. p−value 0.14

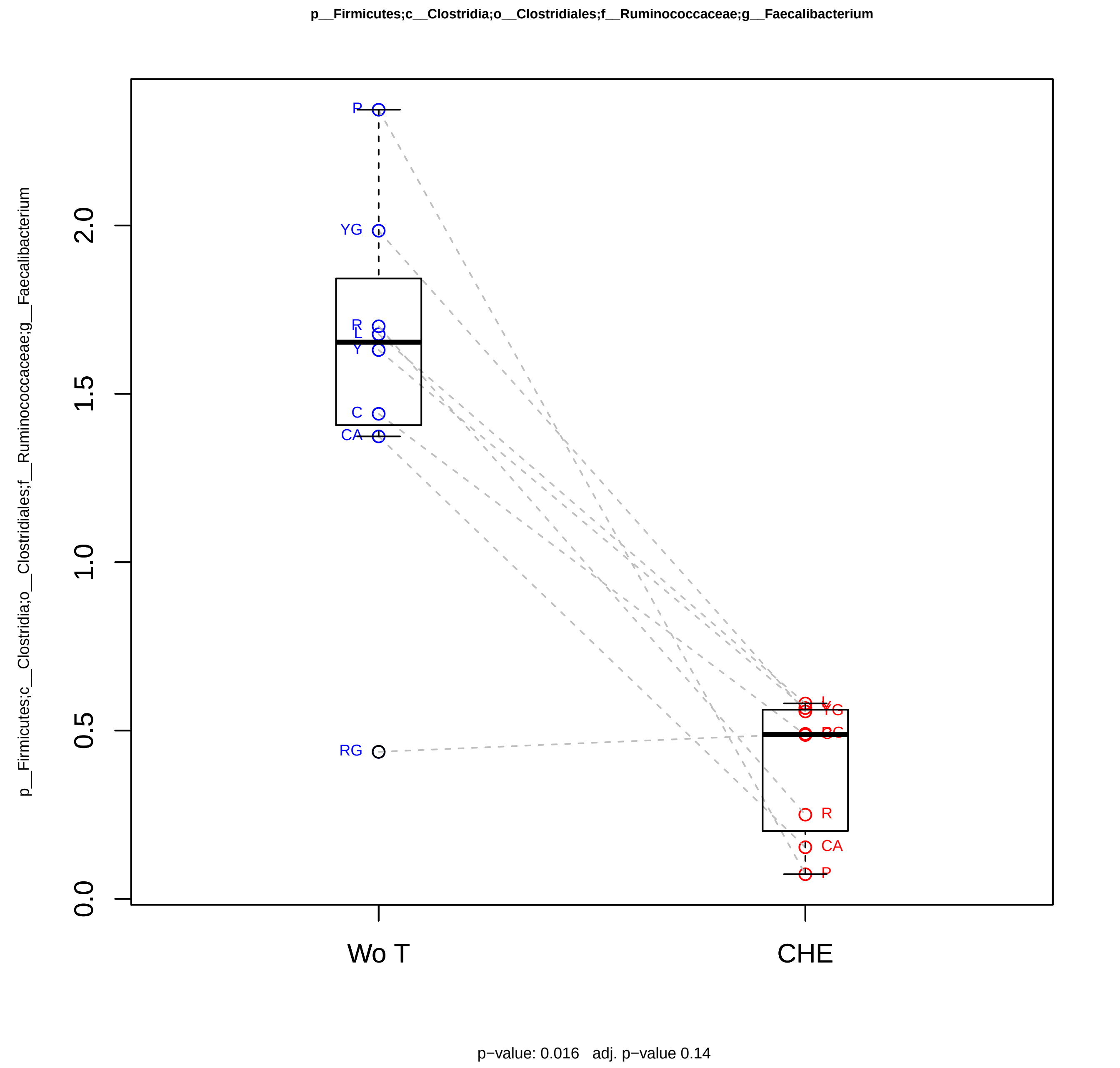
p__Firmicutes;c__Clostridia;o__Clostridiales;f__Ruminococcaceae;g__Faecalibacterium
p__Firmicutes;c__Clostridia;o__Clostridiales;f__Ruminococcaceae;g__Faecalibacterium
0.0
0.5
1.0
1.5
2.0
P
YG
R
L
Y
C
CA
RG
R
CA
P
Wo T
CHE
p−value: 0.016   adj. p−value 0.14

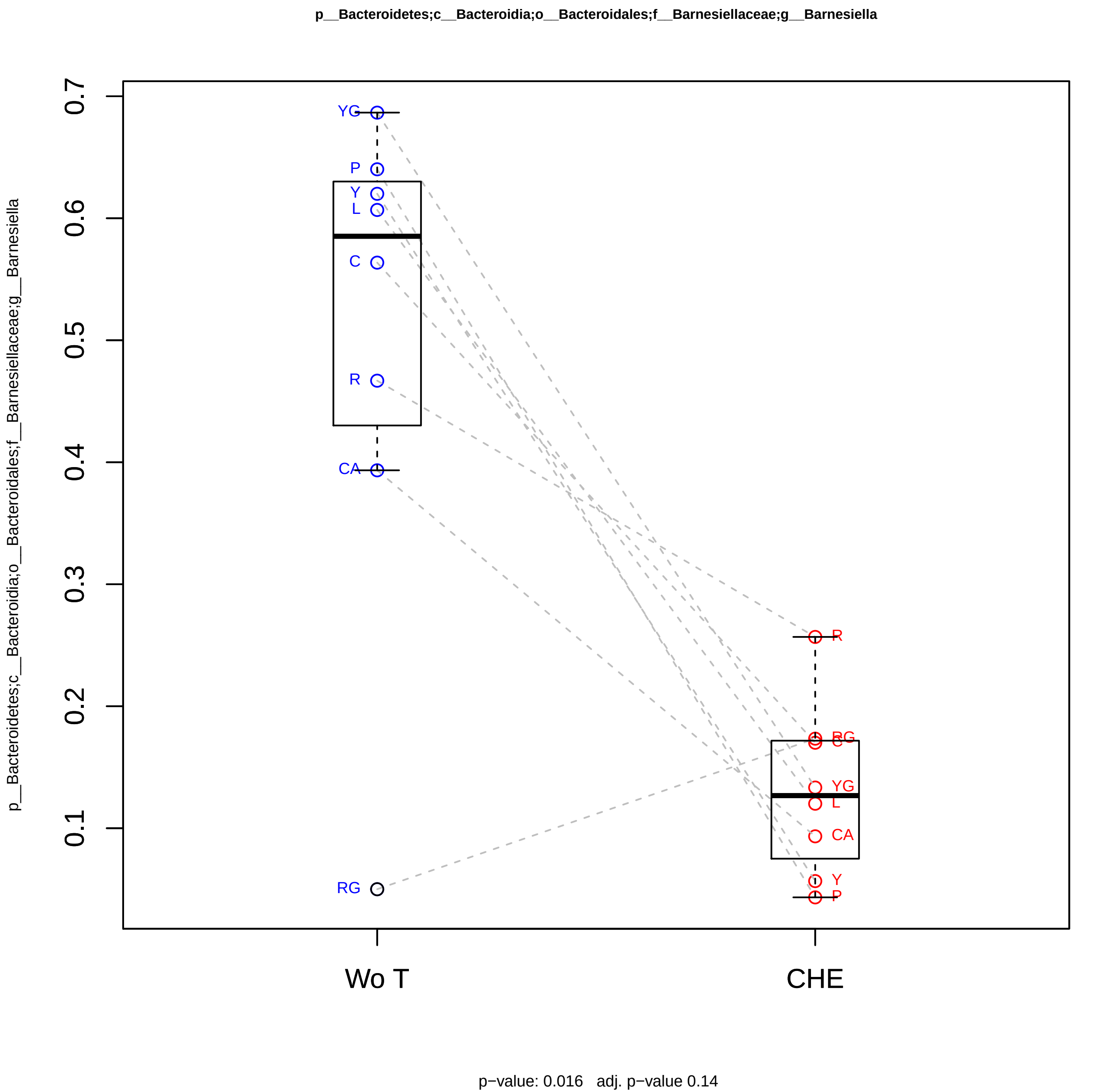
p__Bacteroidetes;c__Bacteroidia;o__Bacteroidales;f__Barnesiellaceae;g__Barnesiella
p__Bacteroidetes;c__Bacteroidia;o__Bacteroidales;f__Barnesiellaceae;g__Barnesiella
0.7
0.6
0.5
0.4
0.3
0.2
0.1
YG
P
Y
L
C
R
CA
RG
R
RG
C
YG
L
CA
Y
P
Wo T
CHE
p−value: 0.016 adj. p−value 0.14

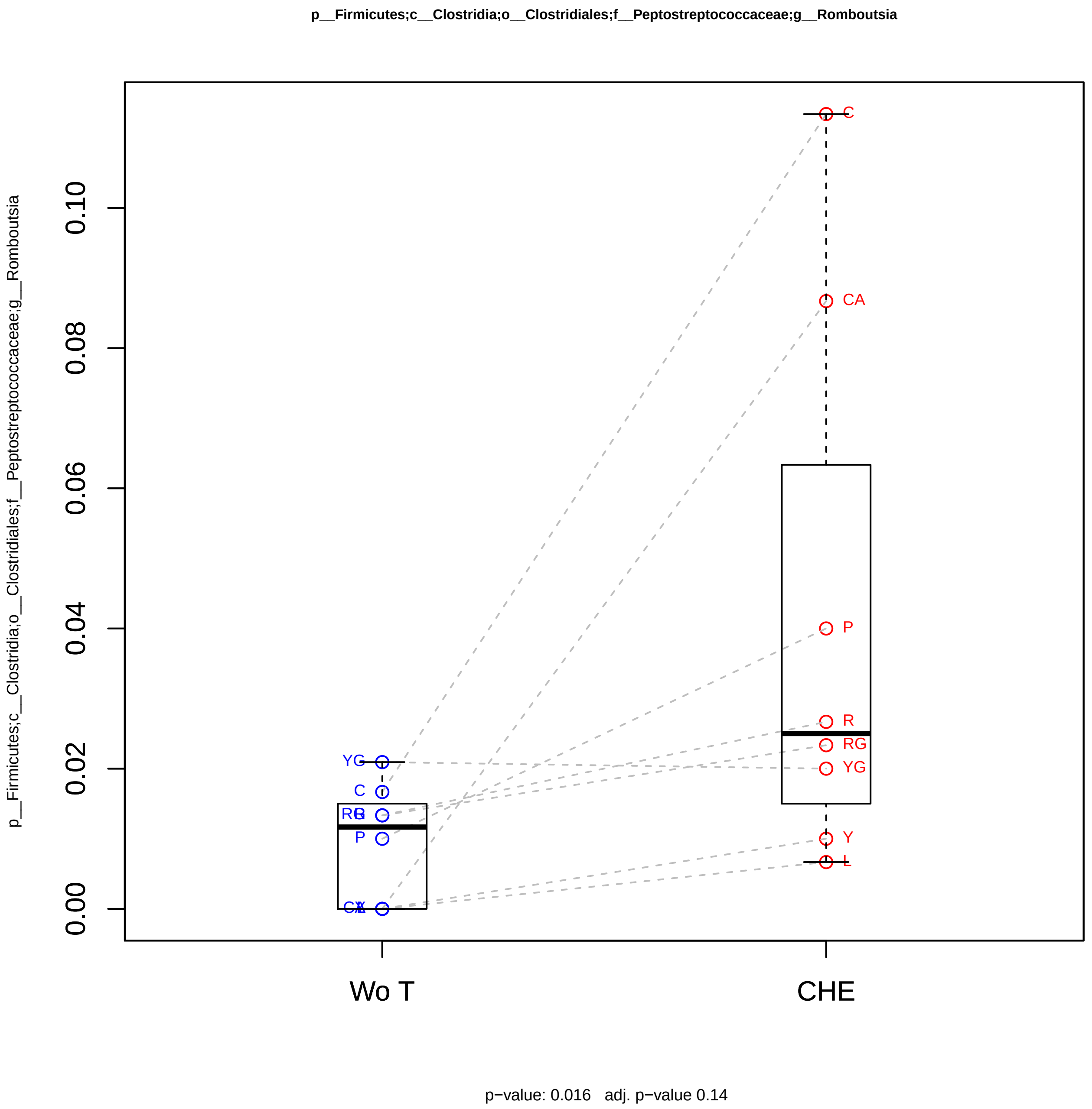
p__Firmicutes;c__Clostridia;o__Clostridiales;f__Peptostreptococcaceae;g__Romboutsia
p__Firmicutes;c__Clostridia;o__Clostridiales;f__Peptostreptococcaceae;g__Romboutsia
0.00
0.02
0.04
0.06
0.08
0.10
Wo T
CHE
YG
C
P
C
CA
P
R
RG
YG
Y
L
p−value: 0.016 adj. p−value 0.14

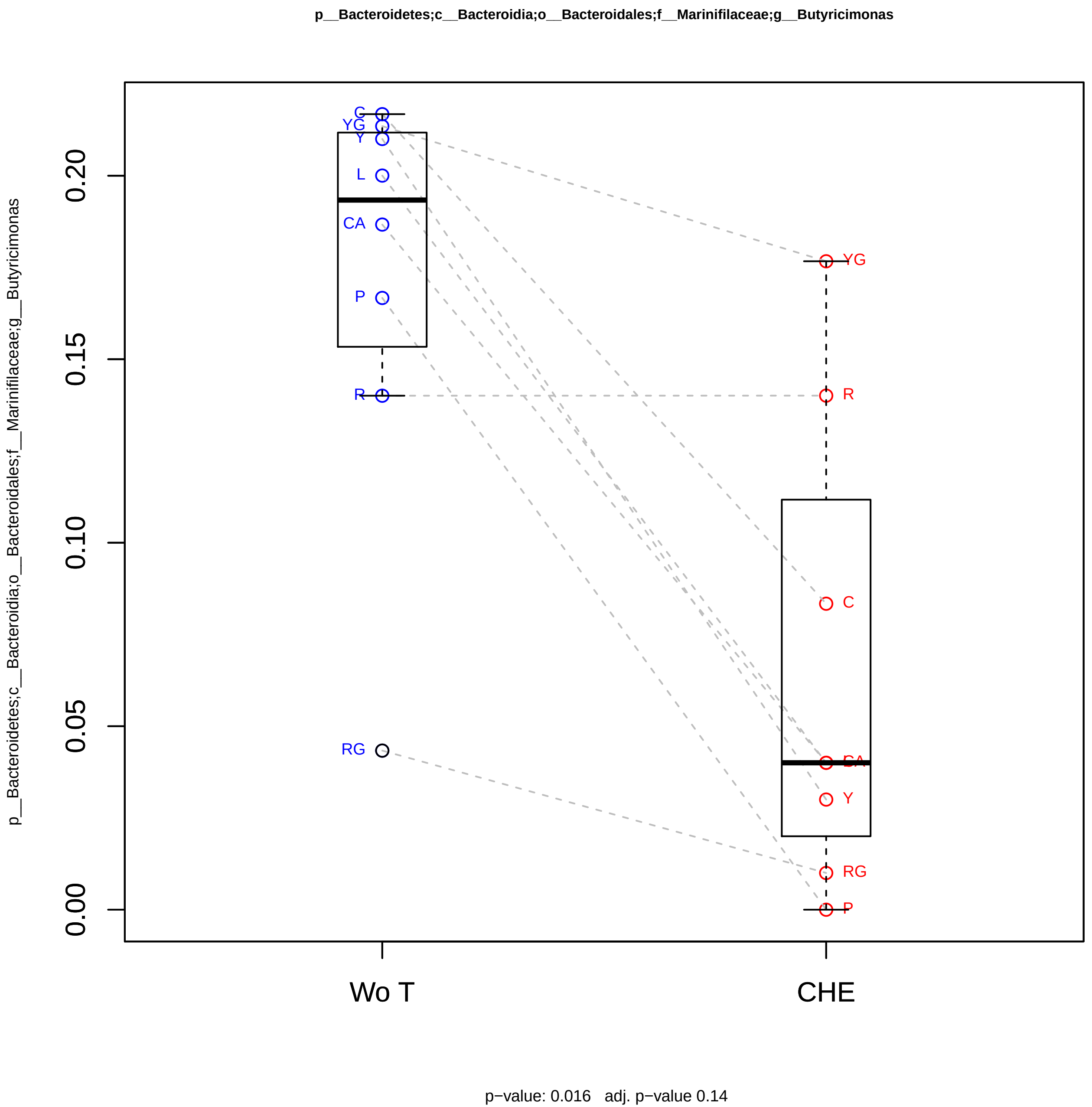
p__Bacteroidetes;c__Bacteroidia;o__Bacteroidales;f__Marinifilaceae;g__Butyricimonas
p__Bacteroidetes;c__Bacteroidia;o__Bacteroidales;f__Marinifilaceae;g__Butyricimonas
0.00
0.05
0.10
0.15
0.20
C
YG
Y
L
CA
P
R
RG
YG
R
C
CA
Y
RG
P
Wo T
CHE
p−value: 0.016 adj. p−value 0.14

p__Bacteroidetes;c__Bacteroidia;o__Bacteroidales;f__Barnesiellaceae;g__Coprobacter

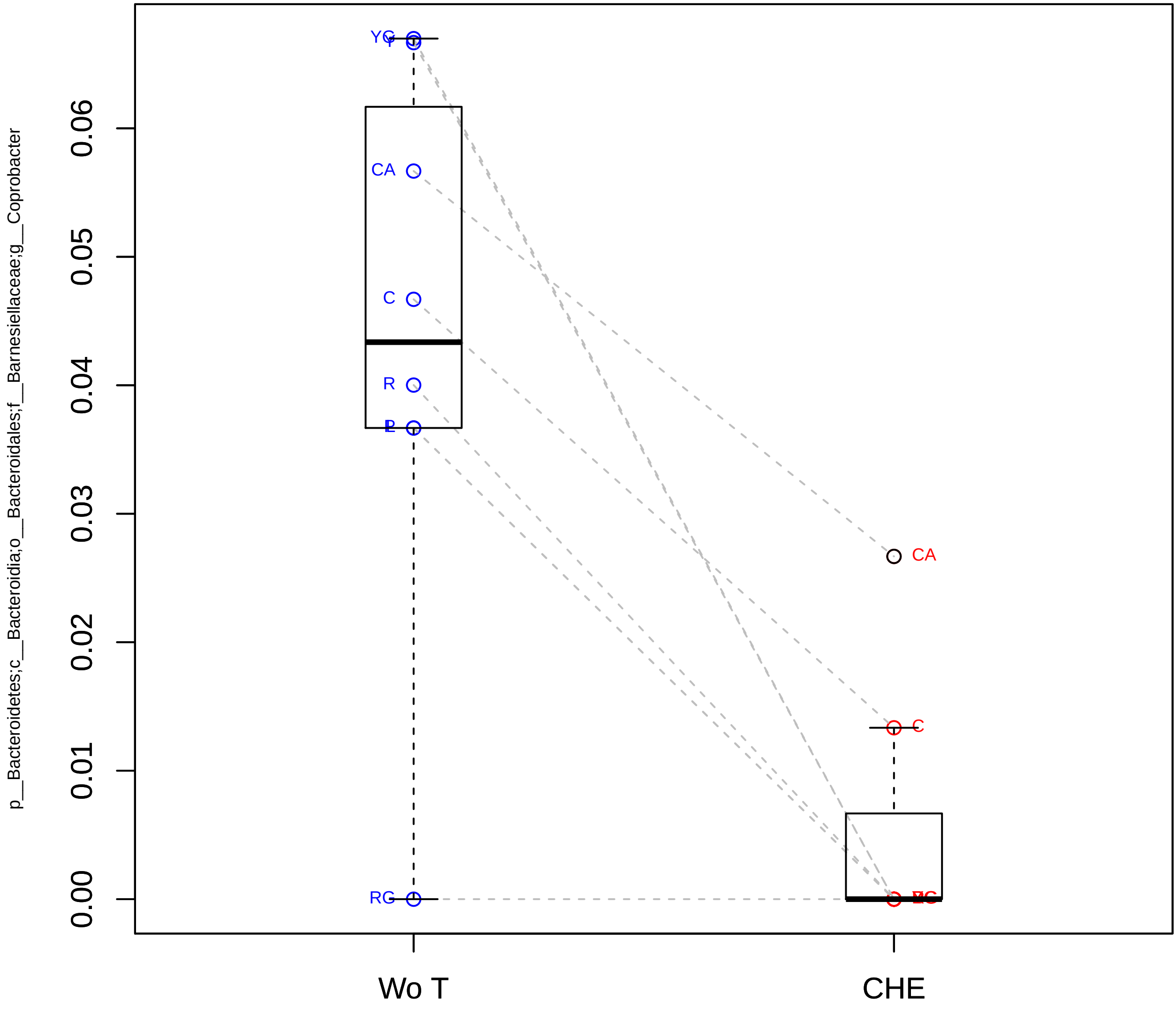


p−value: 0.022 adj. p−value 0.17

p__Firmicutes;c__Negativicutes;o__Selenomonadales;f__Veillonellaceae;g__Veillonella

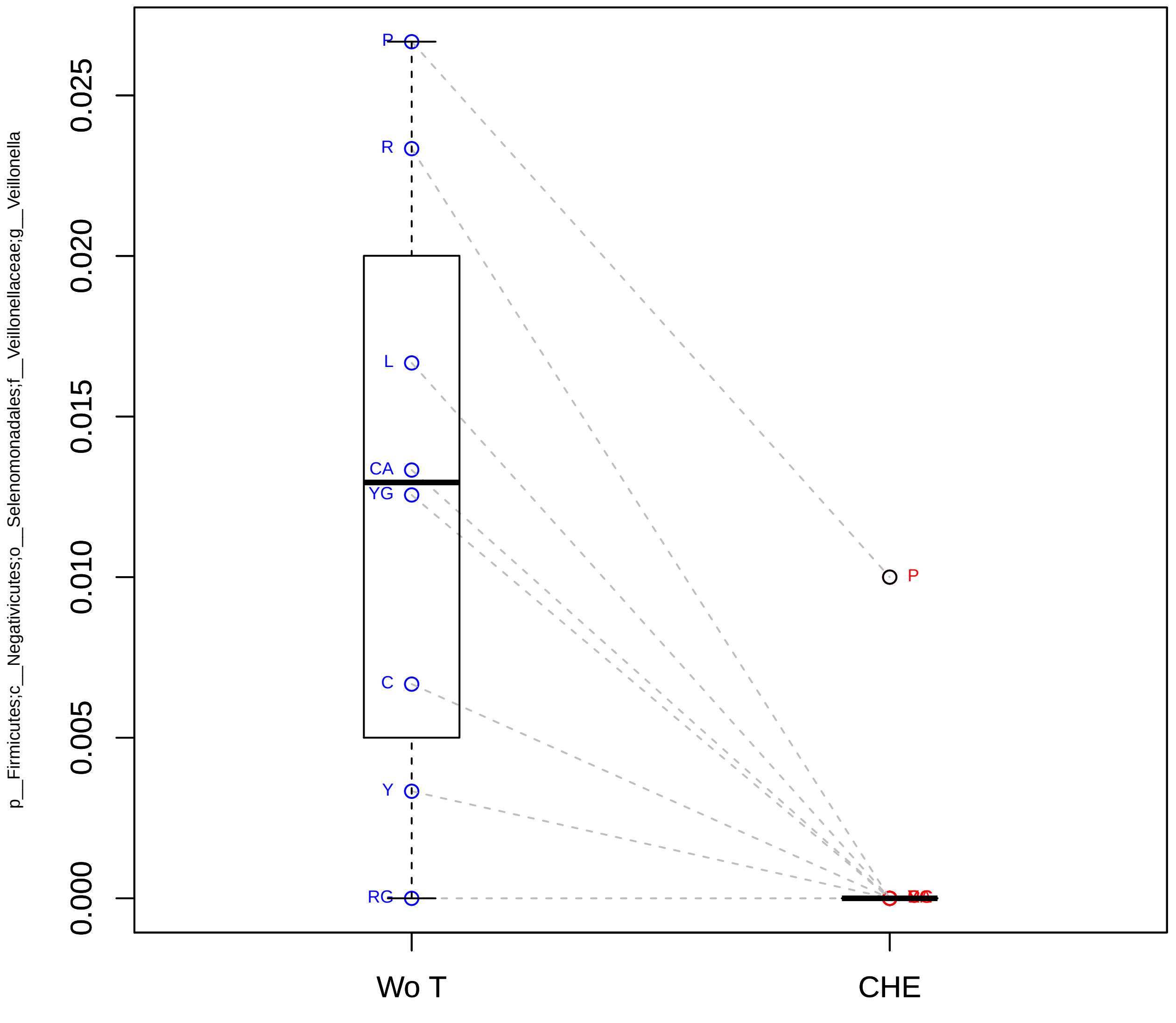


p−value: 0.022 adj. p−value 0.17

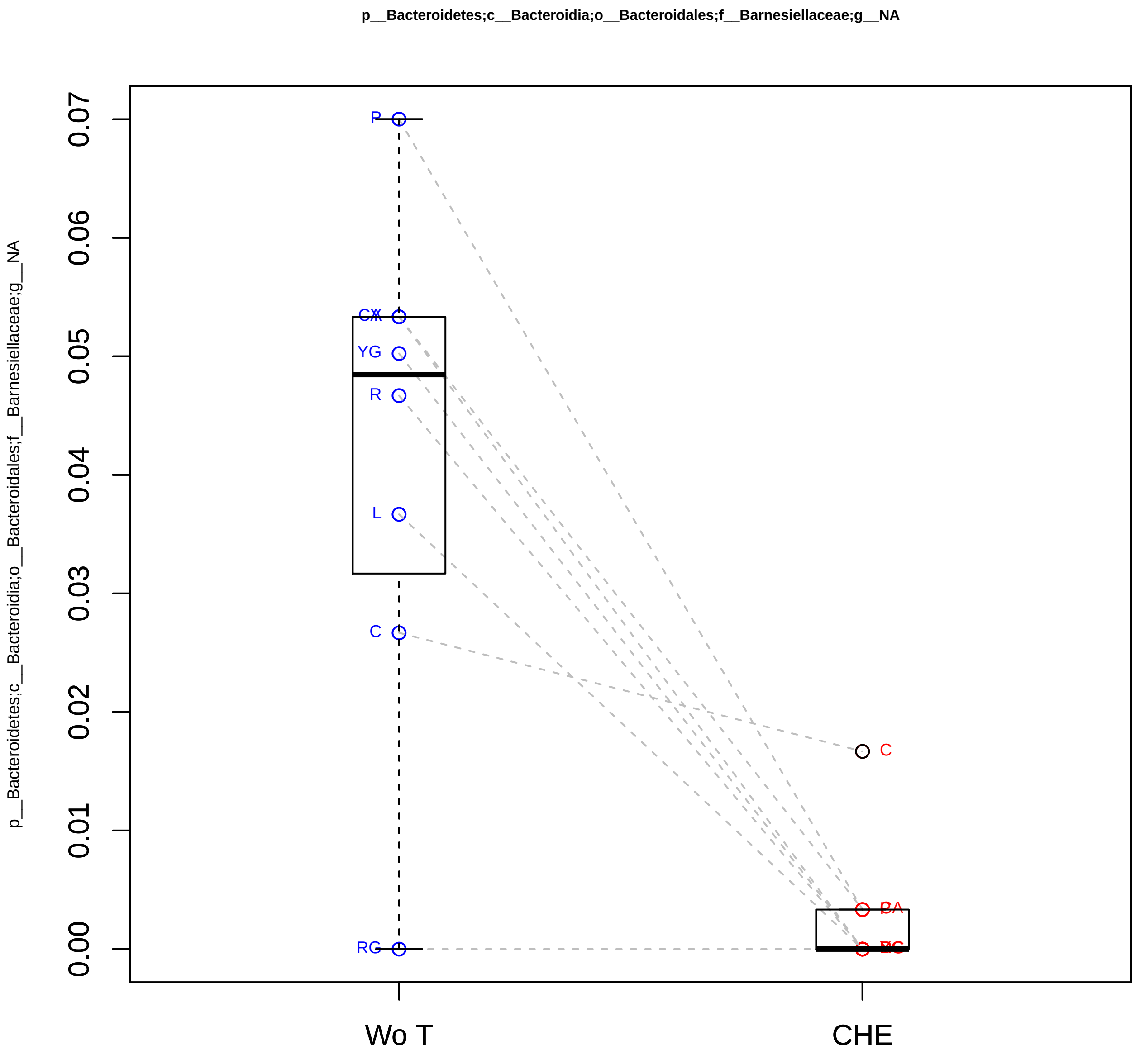


p−value: 0.022 adj. p−value 0.17

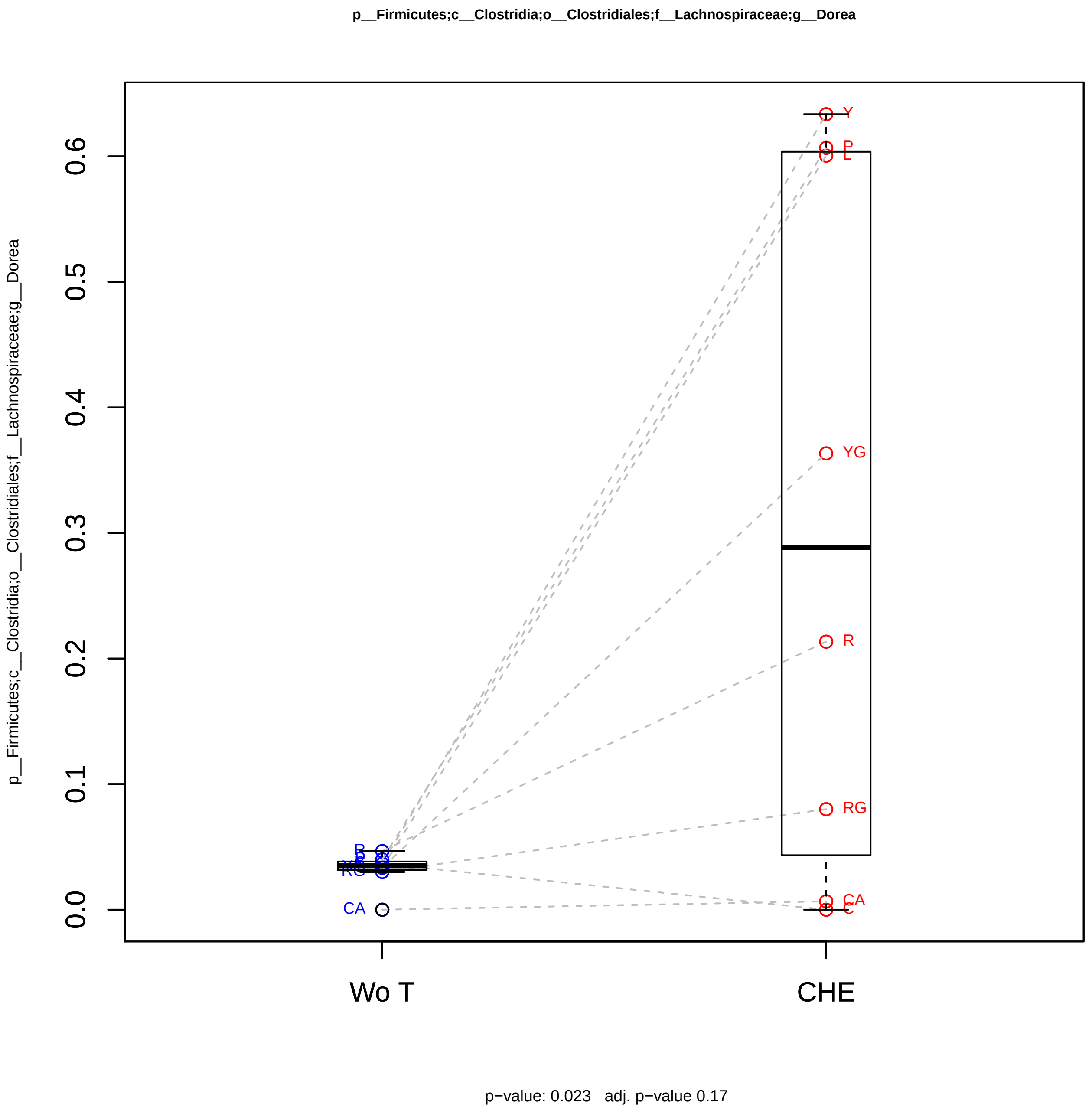
p__Firmicutes;c__Clostridia;o__Clostridiales;f__Lachnospiraceae;g__Dorea
p__Firmicutes;c__Clostridia;o__Clostridiales;f__Lachnospiraceae;g__Dorea
0.0
0.1
0.2
0.3
0.4
0.5
0.6
Y
P
L
YG
R
RG
CA
C
CA
RG
Wo T
CHE
p−value: 0.023 adj. p−value 0.17

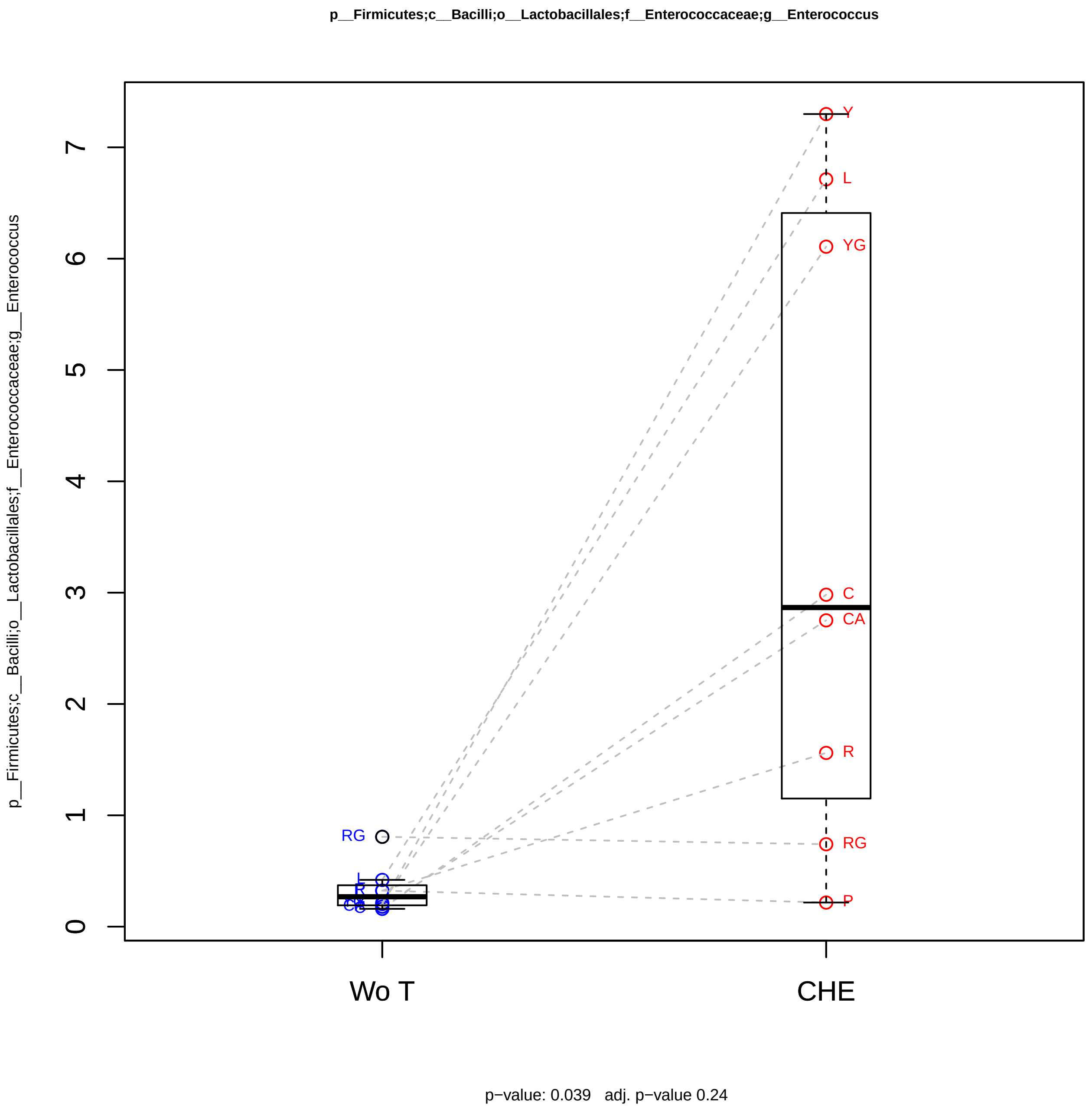
p__Firmicutes;c__Bacilli;o__Lactobacillales;f__Enterococcaceae;g__Enterococcus
p__Firmicutes;c__Bacilli;o__Lactobacillales;f__Enterococcaceae;g__Enterococcus
0
1
2
3
4
5
6
7
Y
L
YG
C
CA
R
RG
P
RG
L
R
Wo T
CHE
p−value: 0.039   adj. p−value 0.24

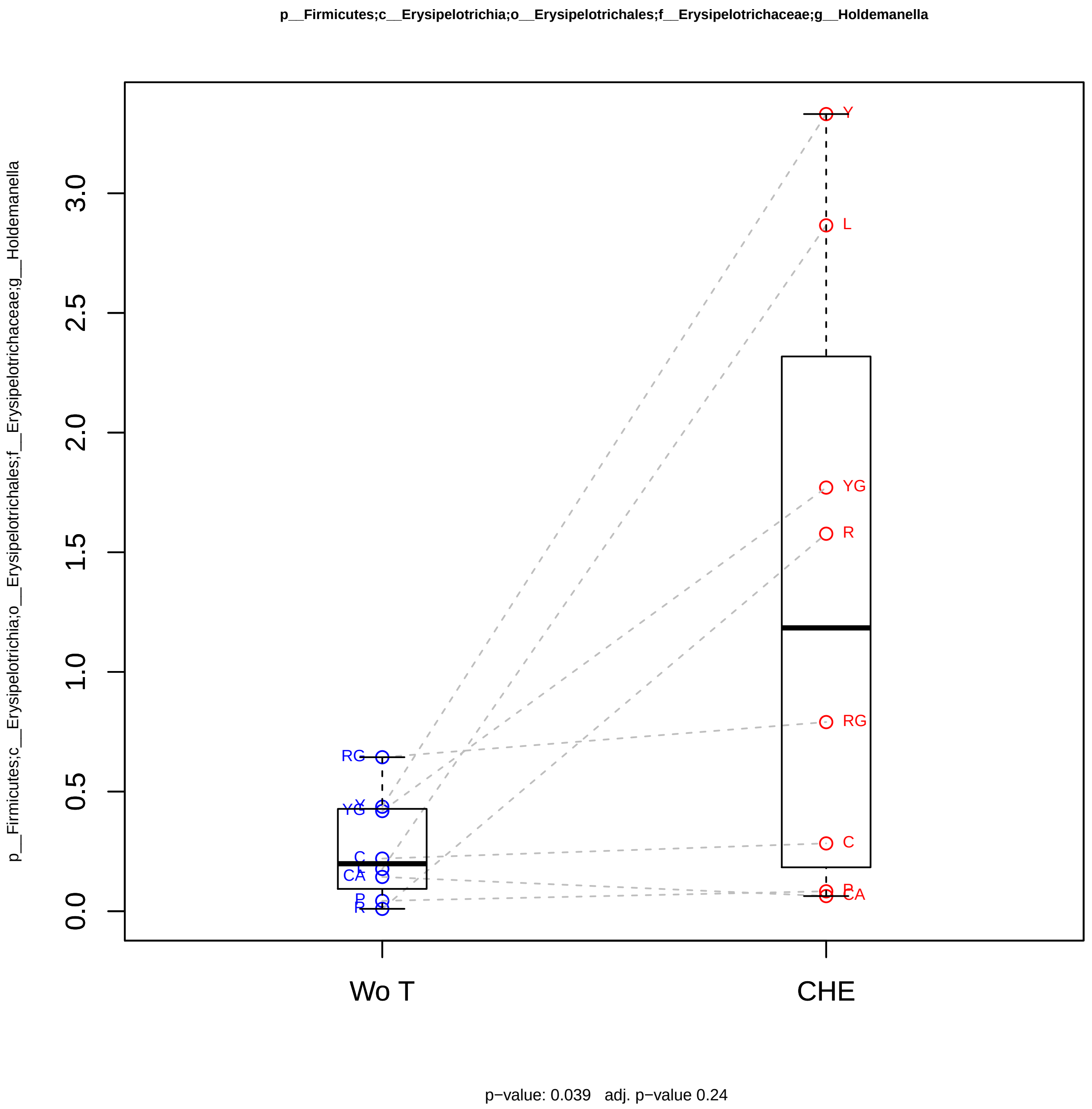

p__Firmicutes;c__Erysipelotrichia;o__Erysipelotrichales;f__Erysipelotrichaceae;g__Holdemanella
p__Firmicutes;c__Erysipelotrichia;o__Erysipelotrichales;f__Erysipelotrichaceae;g__Holdemanella
0.0
0.5
1.0
1.5
2.0
2.5
3.0
Wo T
CHE
RG
Y
YG
C
L
CA
P
R
p−value: 0.039 adj. p−value 0.24

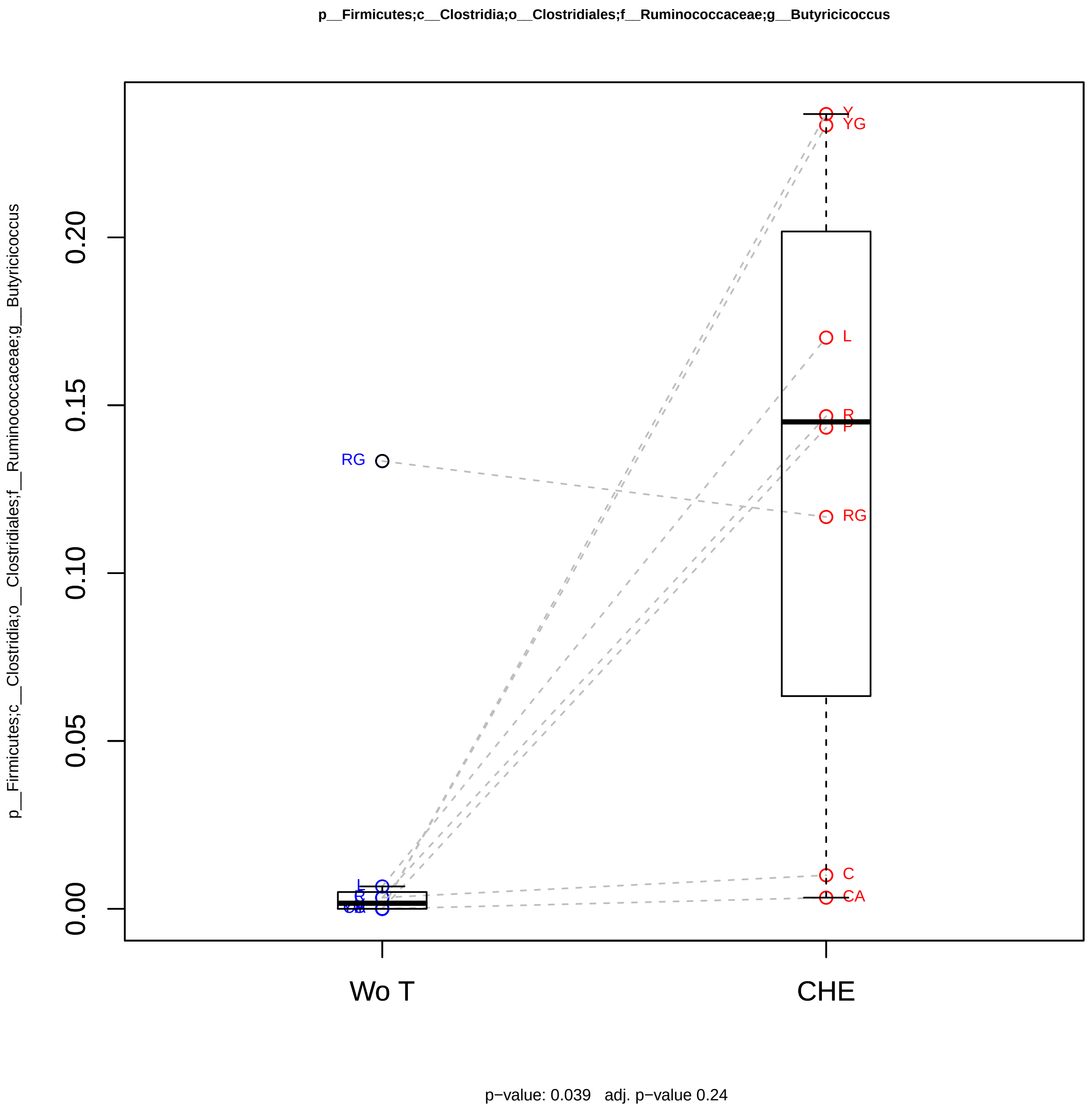
p__Firmicutes;c__Clostridia;o__Clostridiales;f__Ruminococcaceae;g__Butyricicoccus
p__Firmicutes;c__Clostridia;o__Clostridiales;f__Ruminococcaceae;g__Butyricicoccus
0.00
0.05
0.10
0.15
0.20
RG
L
Y
YG
L
R
P
RG
C
CA
Wo T
CHE
p−value: 0.039 adj. p−value 0.24

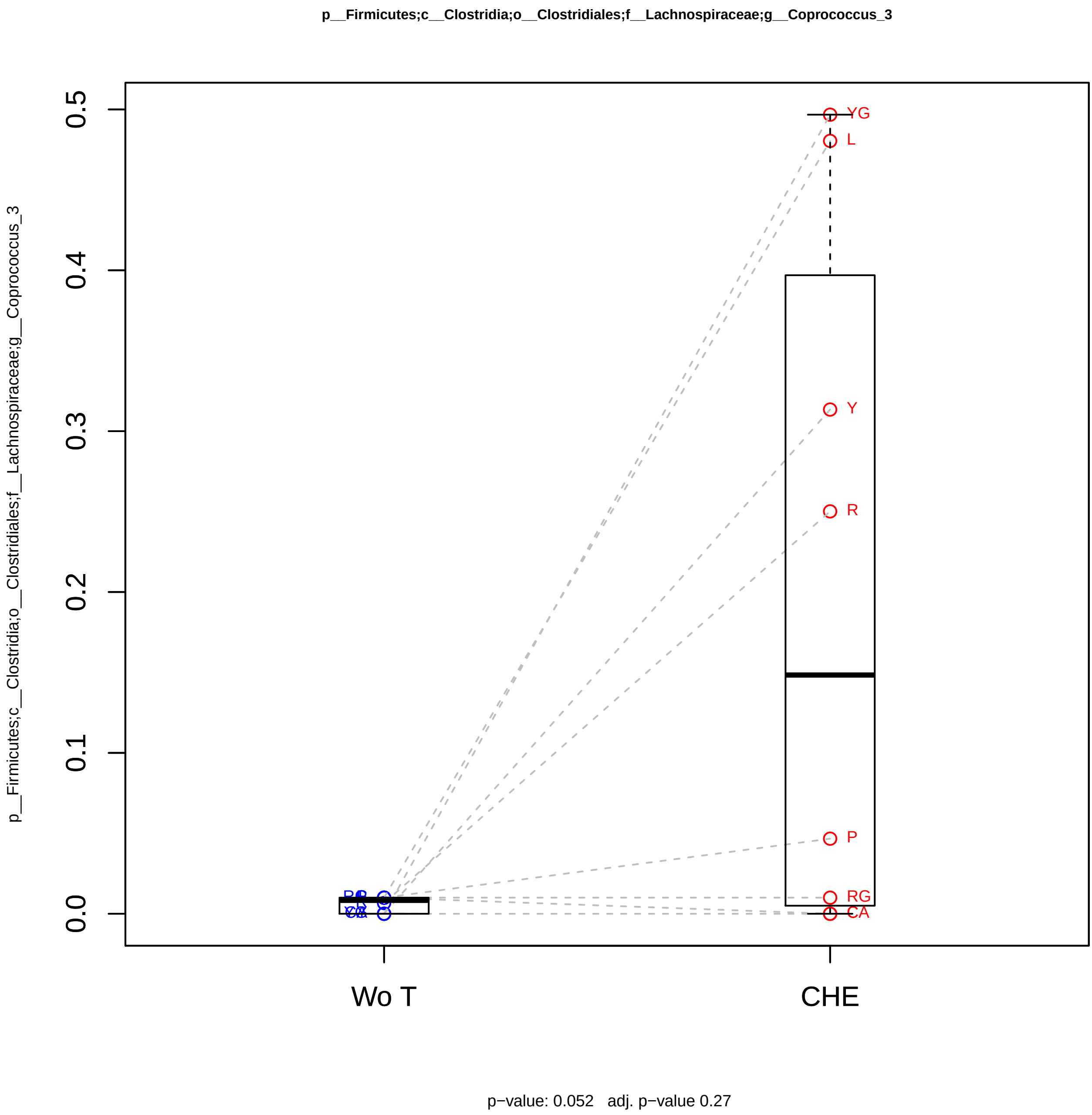

p__Firmicutes;c__Clostridia;o__Clostridiales;f__Lachnospiraceae;g__Coprococcus_3
p__Firmicutes;c__Clostridia;o__Clostridiales;f__Lachnospiraceae;g__Coprococcus_3
0.0
0.1
0.2
0.3
0.4
0.5
YG
L
Y
R
P
RG
CA
Wo T
CHE
p−value: 0.052 adj. p−value 0.27

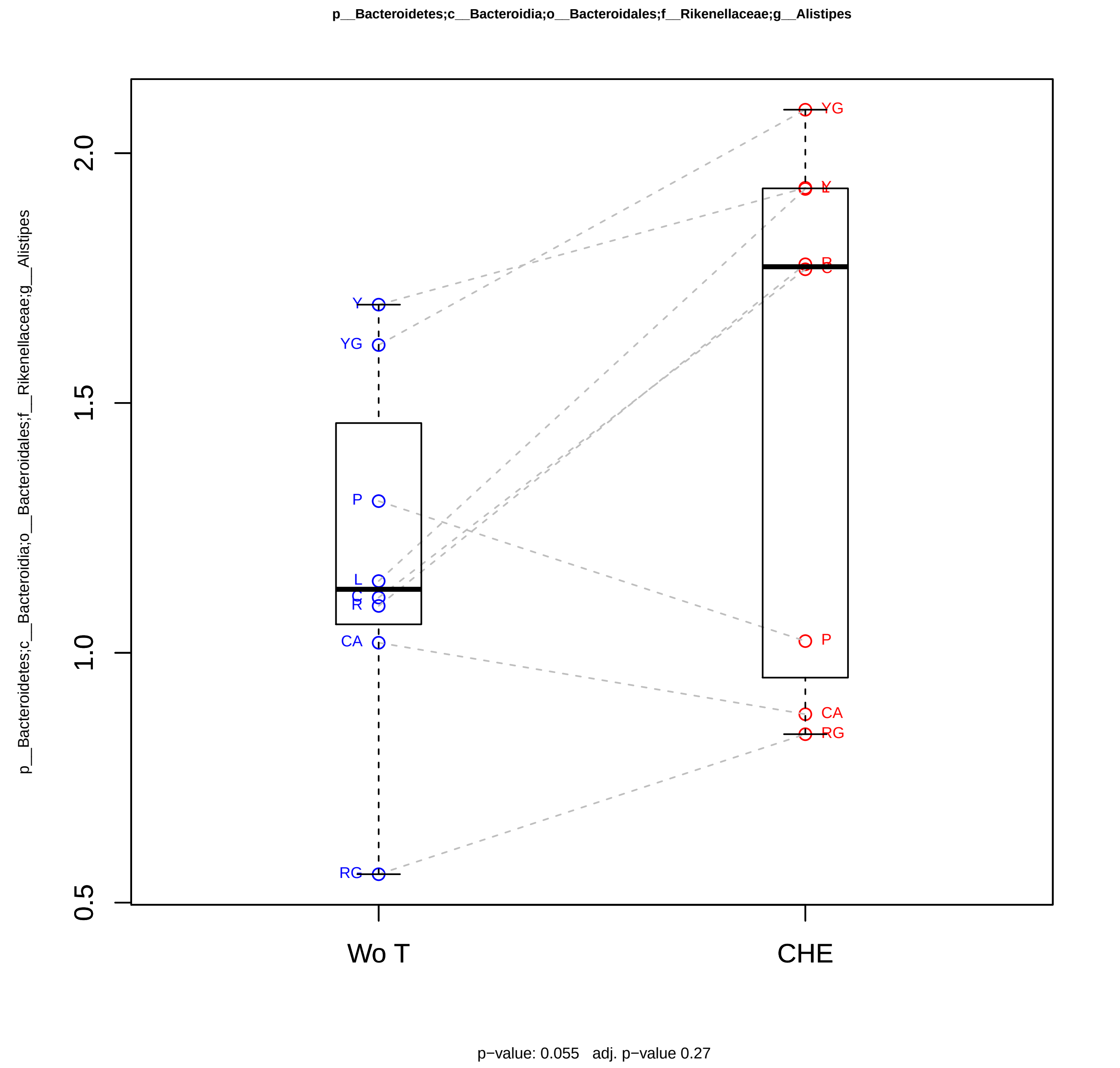
p__Bacteroidetes;c__Bacteroidia;o__Bacteroidales;f__Rikenellaceae;g__Alistipes
p__Bacteroidetes;c__Bacteroidia;o__Bacteroidales;f__Rikenellaceae;g__Alistipes
0.5
1.0
1.5
2.0
Y
YG
P
L
C
R
CA
RG
YG
Y
L
R
C
P
CA
RG
Wo T
CHE
p−value: 0.055 adj. p−value 0.27

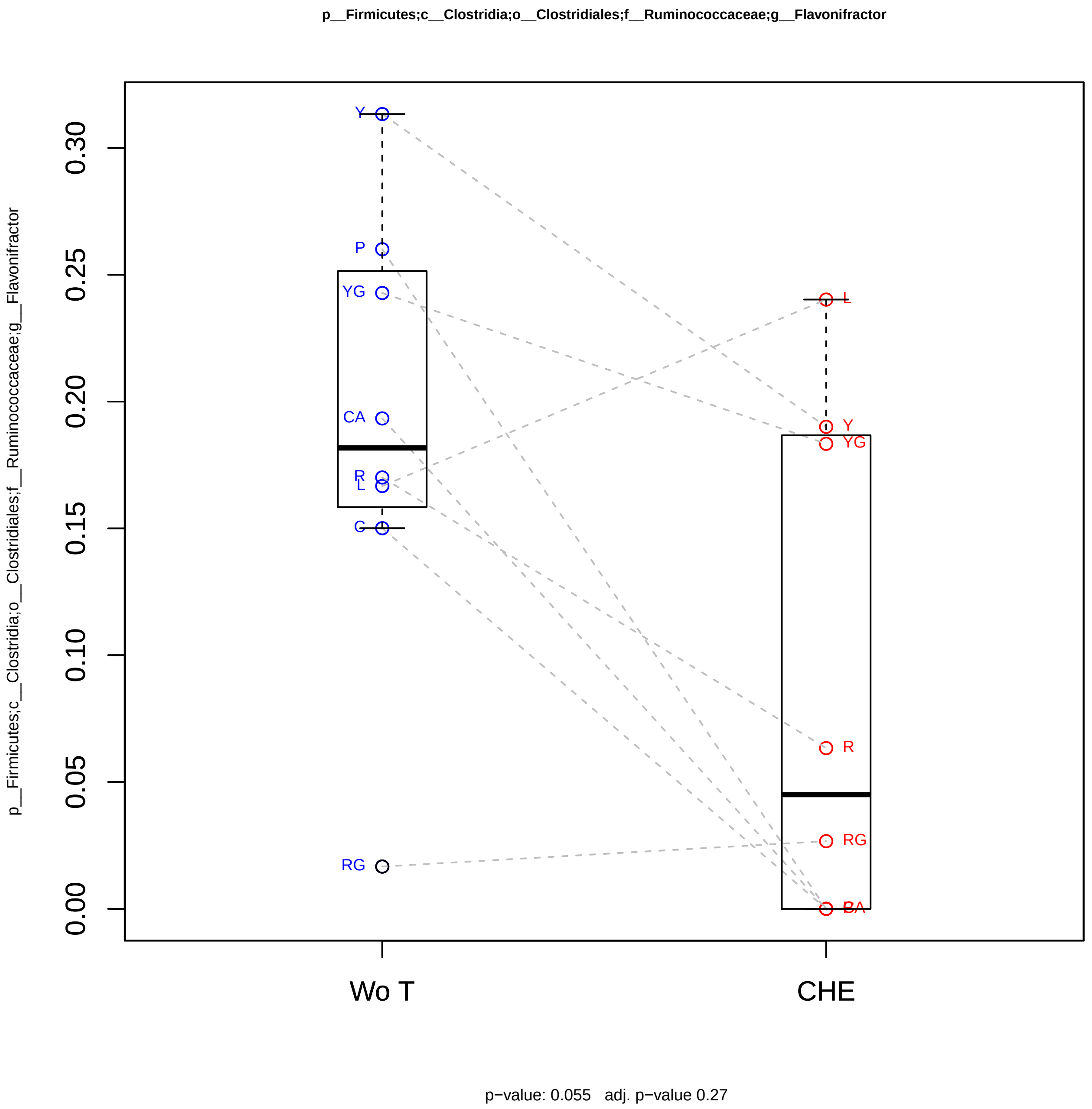
p__Firmicutes;c__Clostridia;o__Clostridiales;f__Ruminococcaceae;g__Flavonifractor
p__Firmicutes;c__Clostridia;o__Clostridiales;f__Ruminococcaceae;g__Flavonifractor
0.00
0.05
0.10
0.15
0.20
0.25
0.30
Y
P
YG
CA
R
L
C
RG
L
Y
YG
R
RG
CA
Wo T
CHE
p−value: 0.055 adj. p−value 0.27

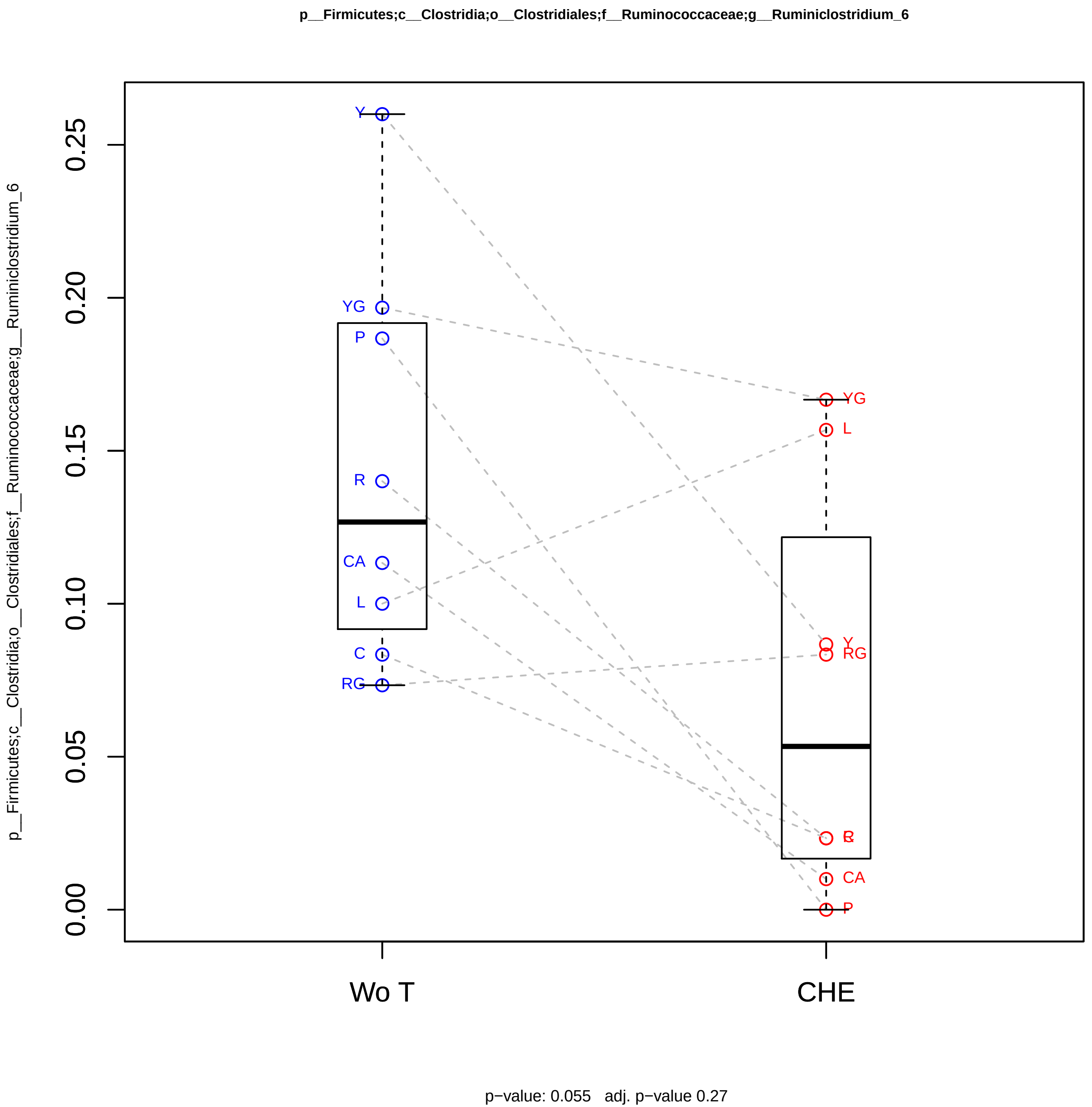
p__Firmicutes;c__Clostridia;o__Clostridiales;f__Ruminococcaceae;g__Ruminiclostridium_6
p__Firmicutes;c__Clostridia;o__Clostridiales;f__Ruminococcaceae;g__Ruminiclostridium_6
0.00
0.05
0.10
0.15
0.20
0.25
Wo T
CHE
Y
YG
P
R
CA
L
C
RG
p−value: 0.055 adj. p−value 0.27

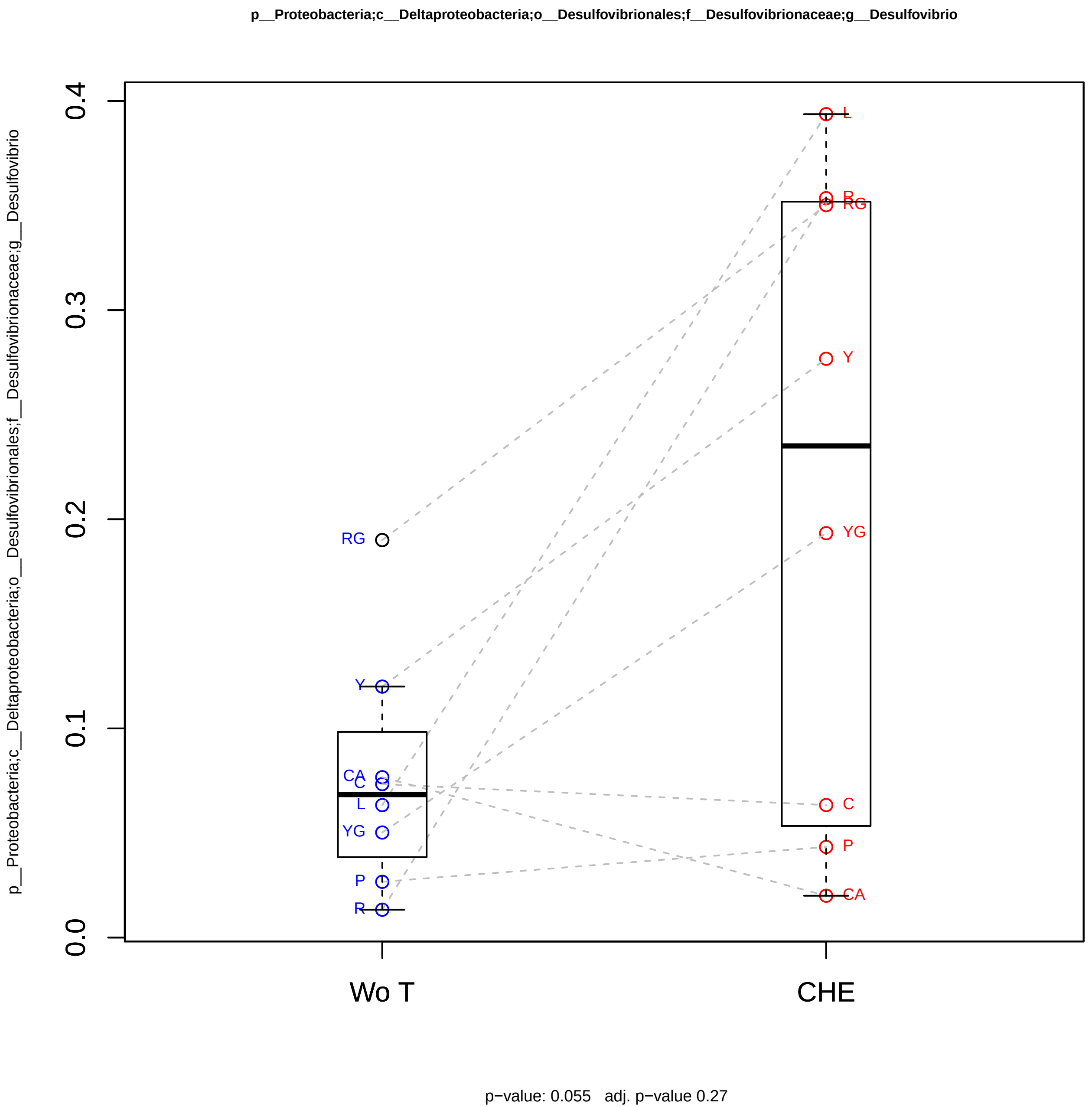

p__Proteobacteria;c__Deltaproteobacteria;o__Desulfovibrionales;f__Desulfovibrionaceae;g__Desulfovibrio
p__Proteobacteria;c__Deltaproteobacteria;o__Desulfovibrionales;f__Desulfovibrionaceae;g__Desulfovibrio
0.0
0.1
0.2
0.3
0.4
Wo T
CHE
RG
Y
CA
C
L
YG
P
R
L
R
RG
Y
YG
C
P
CA
p−value: 0.055 adj. p−value 0.27

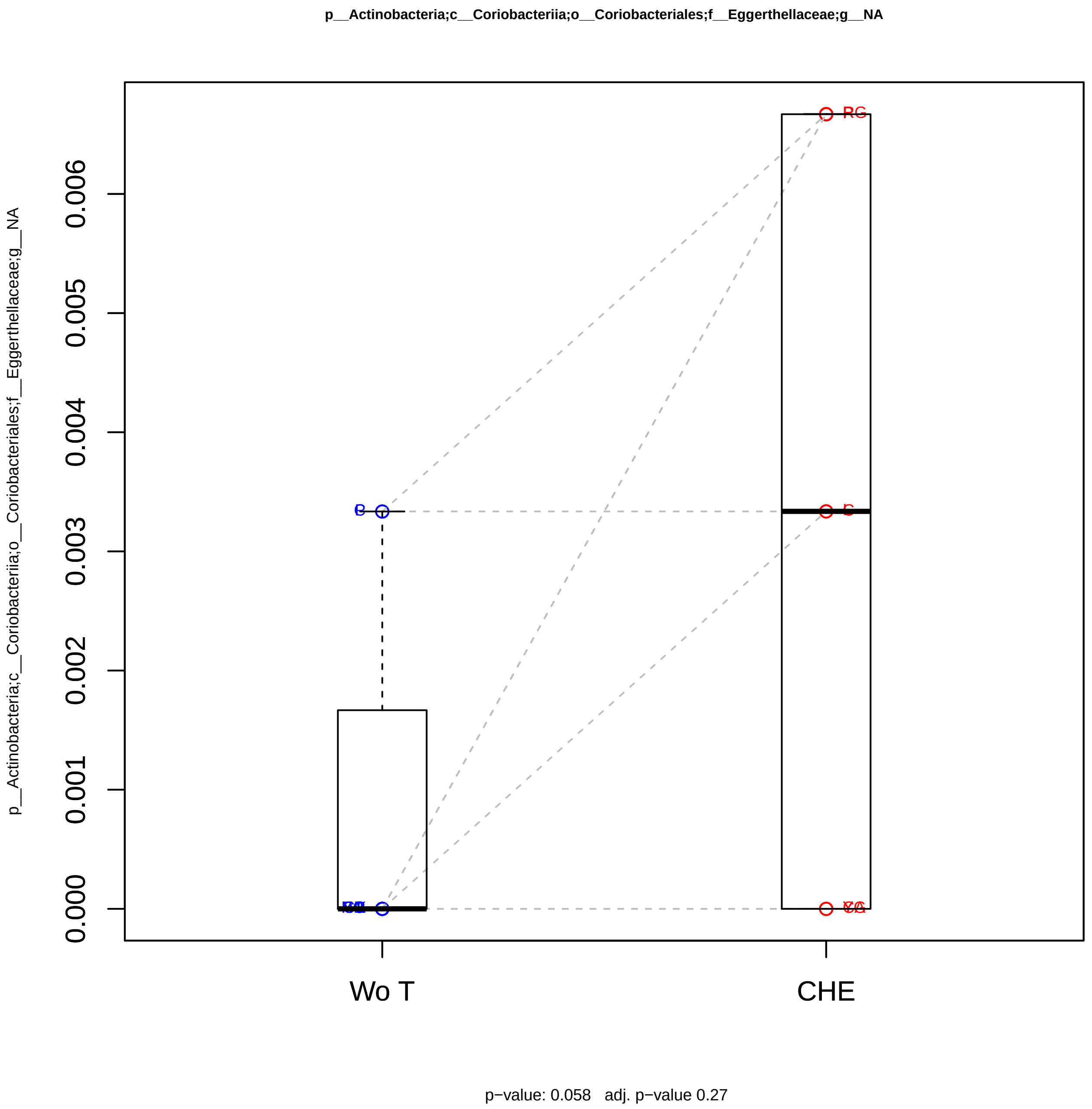
p__Actinobacteria;c__Coriobacteriia;o__Coriobacteriales;f__Eggerthellaceae;g__NA
p__Actinobacteria;c__Coriobacteriia;o__Coriobacteriales;f__Eggerthellaceae;g__NA
0.000
0.001
0.002
0.003
0.004
0.005
0.006
Wo T
CHE
p−value: 0.058 adj. p−value 0.27

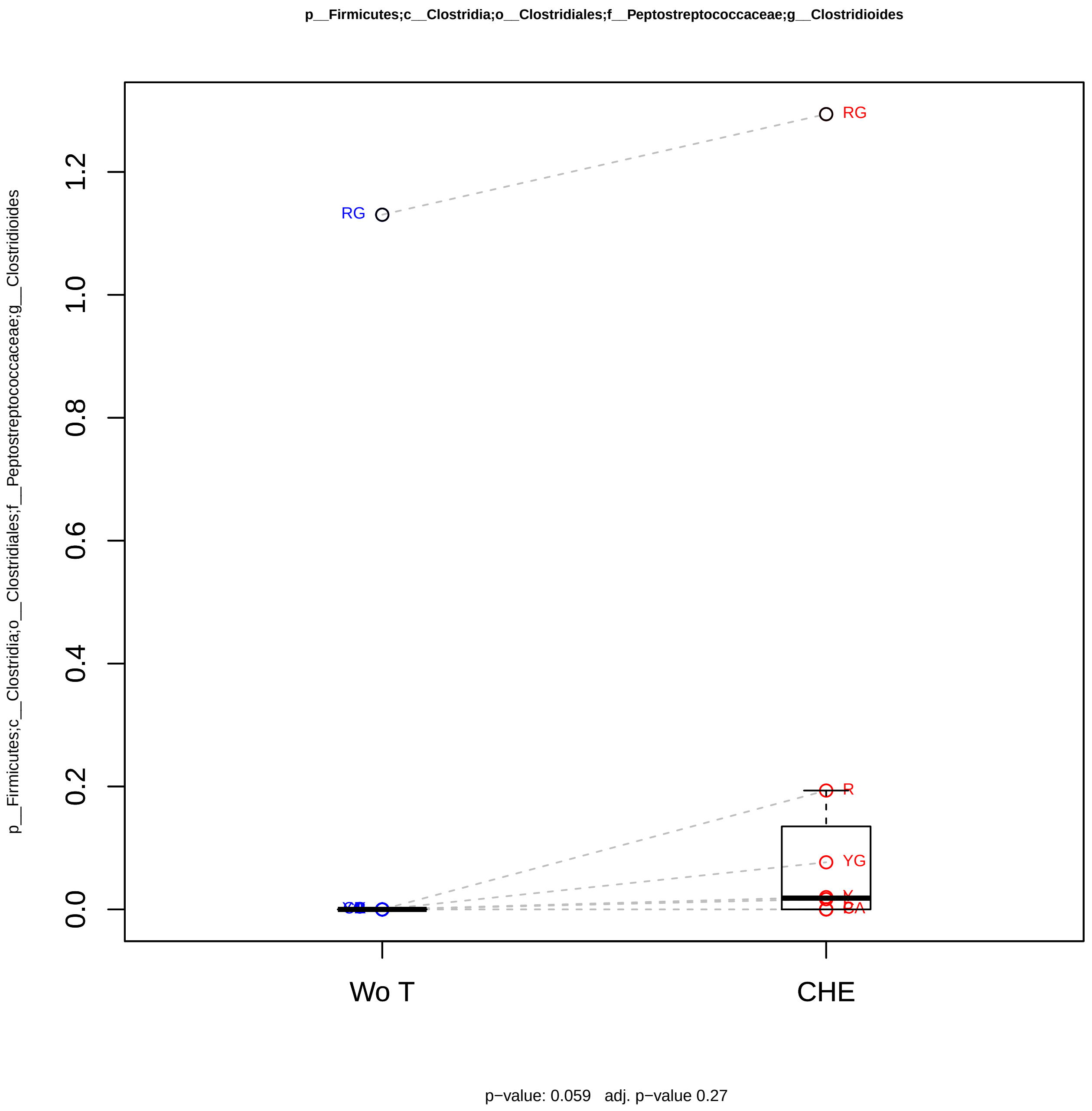
p__Firmicutes;c__Clostridia;o__Clostridiales;f__Peptostreptococcaceae;g__Clostridioides
p__Firmicutes;c__Clostridia;o__Clostridiales;f__Peptostreptococcaceae;g__Clostridioides
0.0
0.2
0.4
0.6
0.8
1.0
1.2
RG
RG
R
YG
Wo T
CHE
p−value: 0.059 adj. p−value 0.27

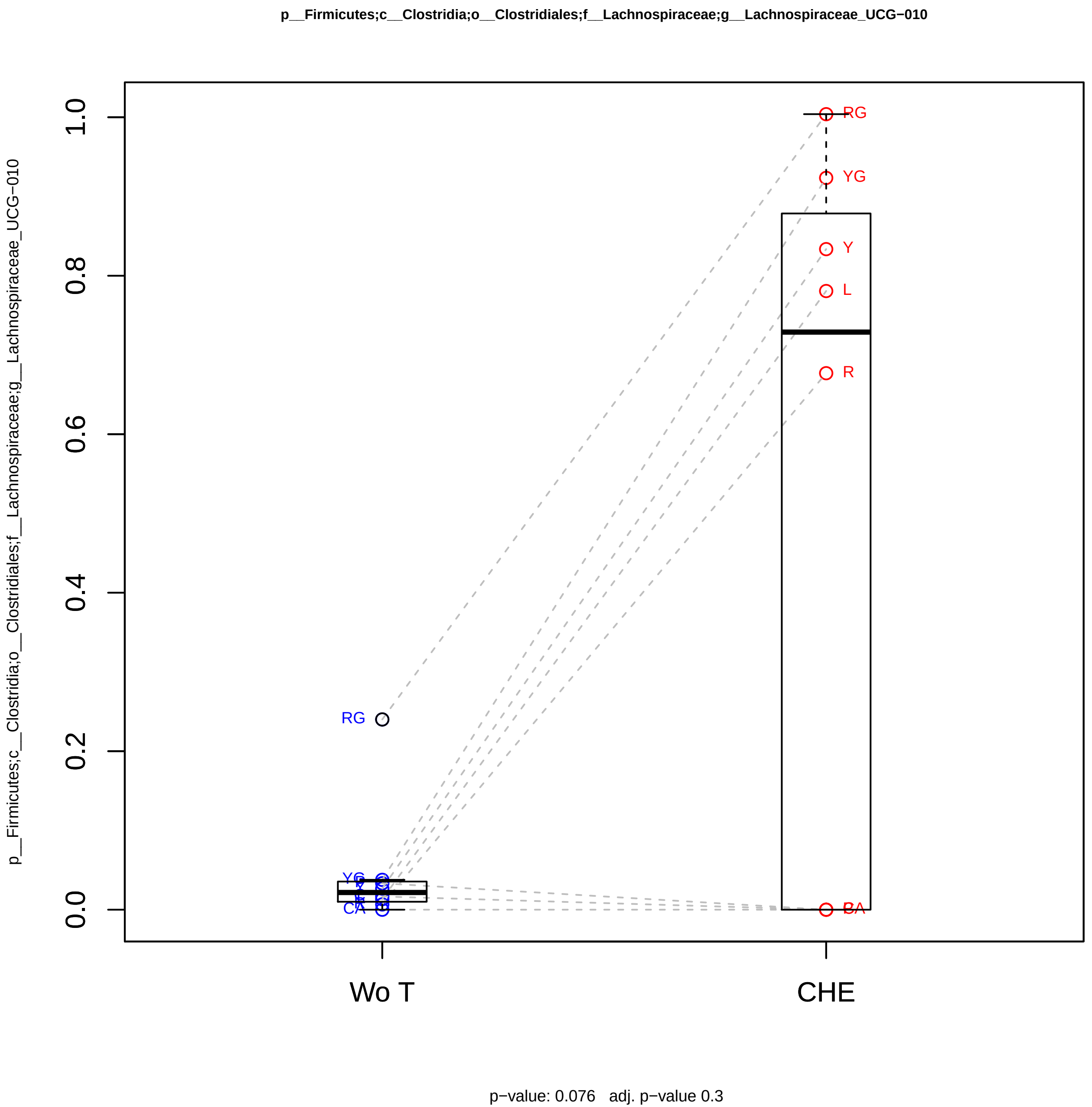
p__Firmicutes;c__Clostridia;o__Clostridiales;f__Lachnospiraceae;g__Lachnospiraceae_UCG−010
p__Firmicutes;c__Clostridia;o__Clostridiales;f__Lachnospiraceae;g__Lachnospiraceae_UCG−010
0.0
0.2
0.4
0.6
0.8
1.0
RG
YG
Y
L
R
CA
RG
YG
Y
L
R
Wo T
CHE
p−value: 0.076 adj. p−value 0.3

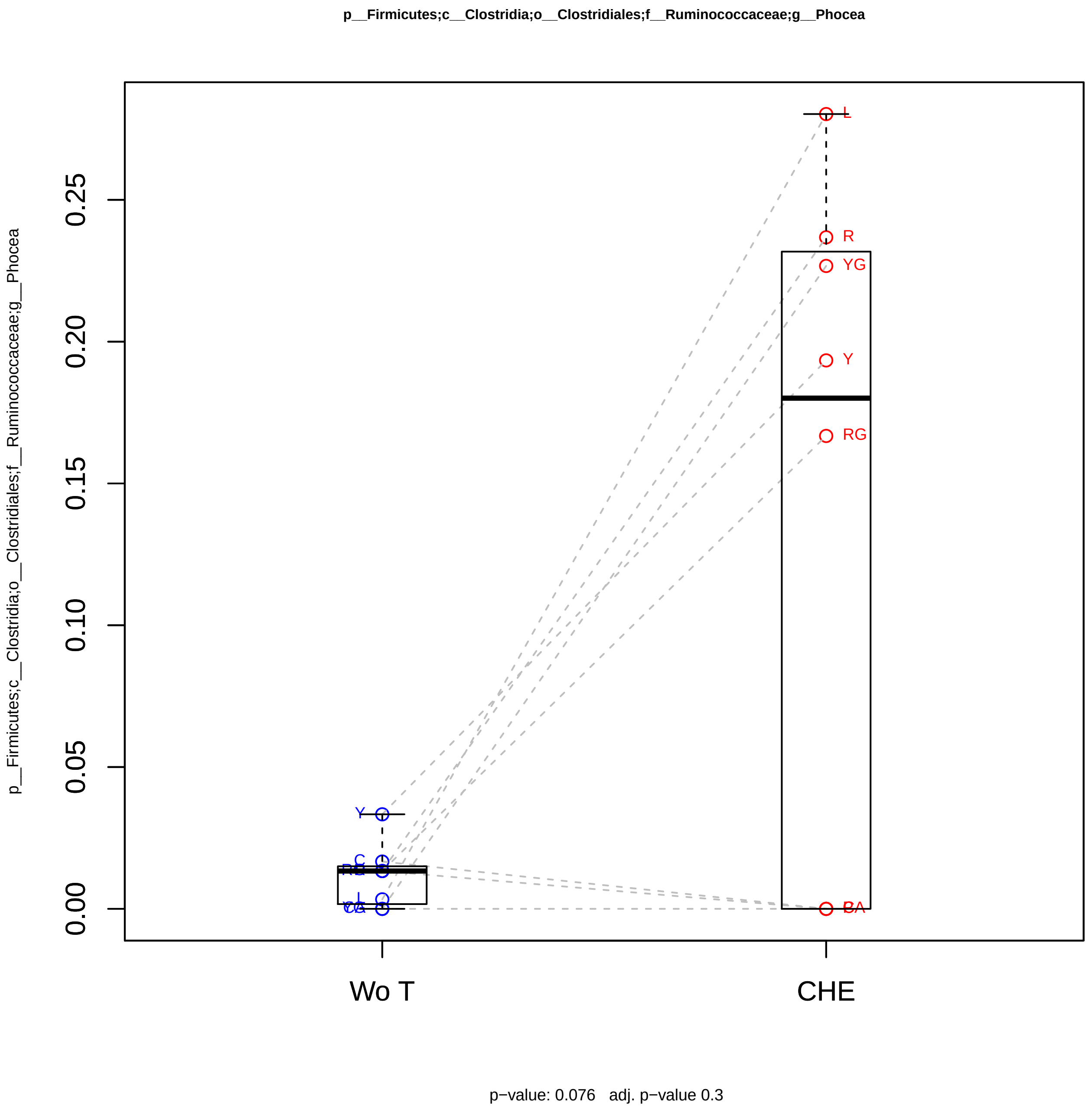

p__Firmicutes;c__Clostridia;o__Clostridiales;f__Ruminococcaceae;g__Phocea
p__Firmicutes;c__Clostridia;o__Clostridiales;f__Ruminococcaceae;g__Phocea
0.00
0.05
0.10
0.15
0.20
0.25
Wo T
CHE
L
R
YG
Y
RG
C
p-value: 0.076 adj. p-value 0.3

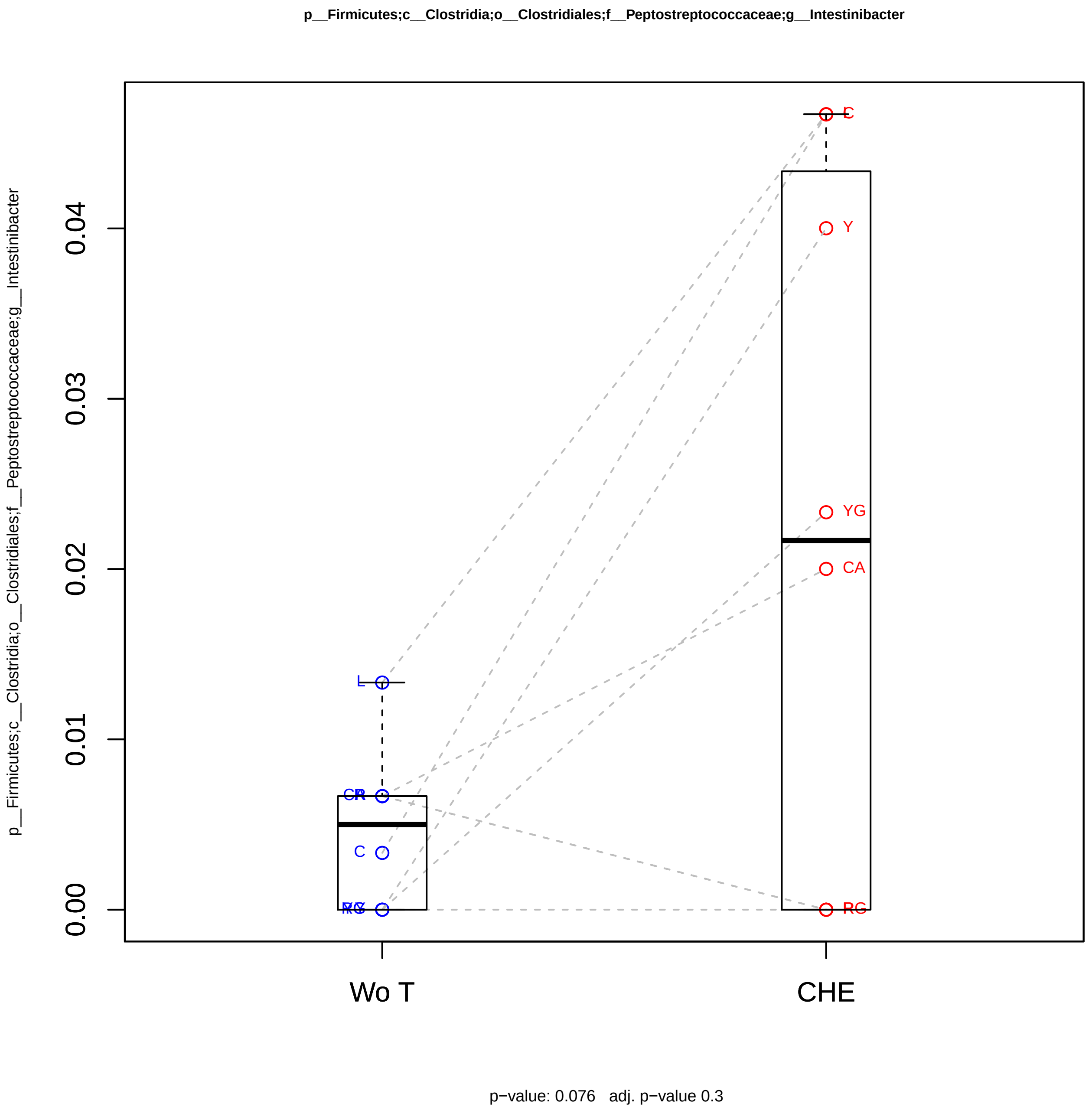
p__Firmicutes;c__Clostridia;o__Clostridiales;f__Peptostreptococcaceae;g__Intestinibacter
p__Firmicutes;c__Clostridia;o__Clostridiales;f__Peptostreptococcaceae;g__Intestinibacter
0.00
0.01
0.02
0.03
0.04
Wo T
CHE
L
C
Y
YG
CA
RG
p−value: 0.076 adj. p−value 0.3

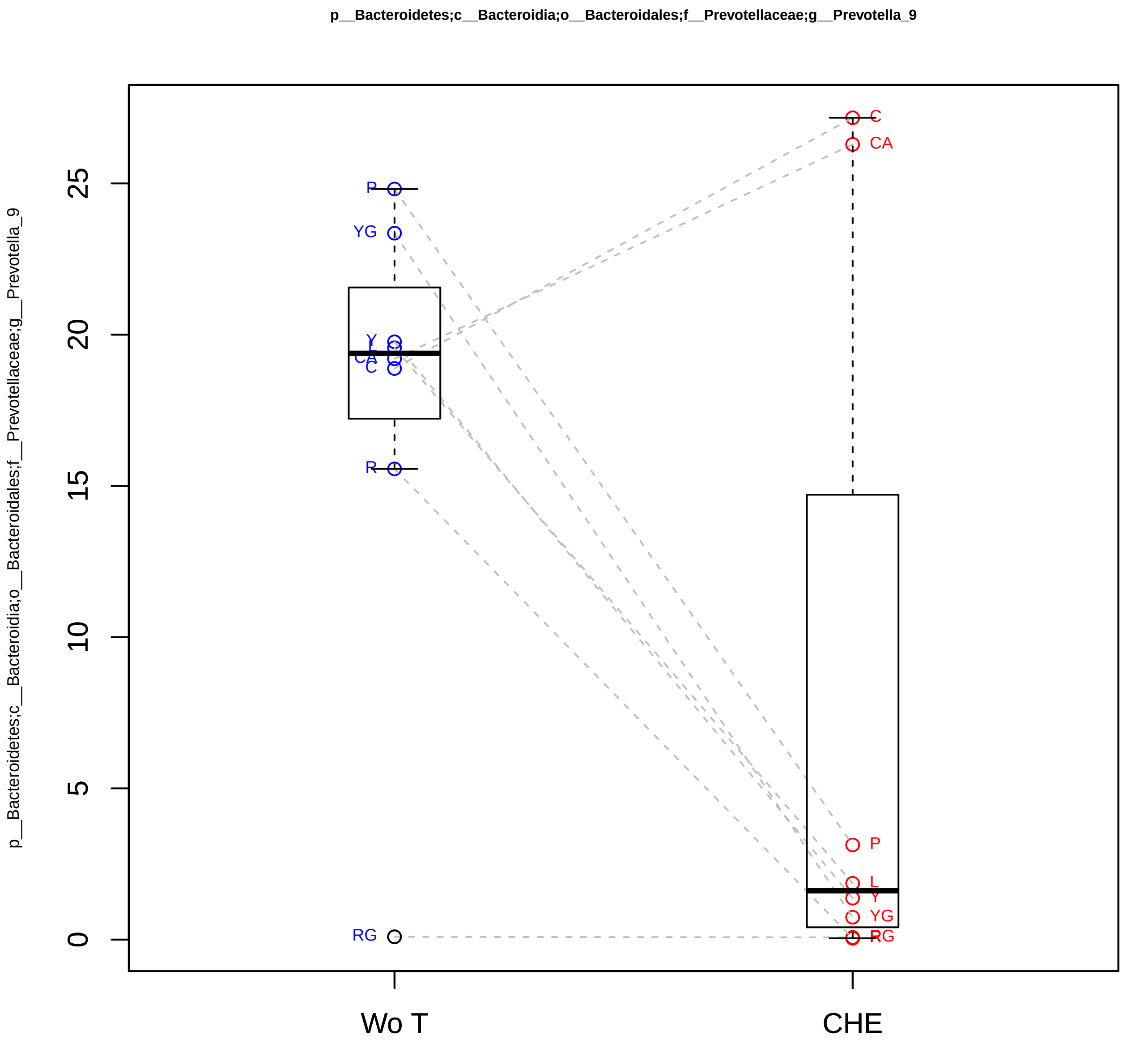


p−value: 0.078 adj. p−value 0.3

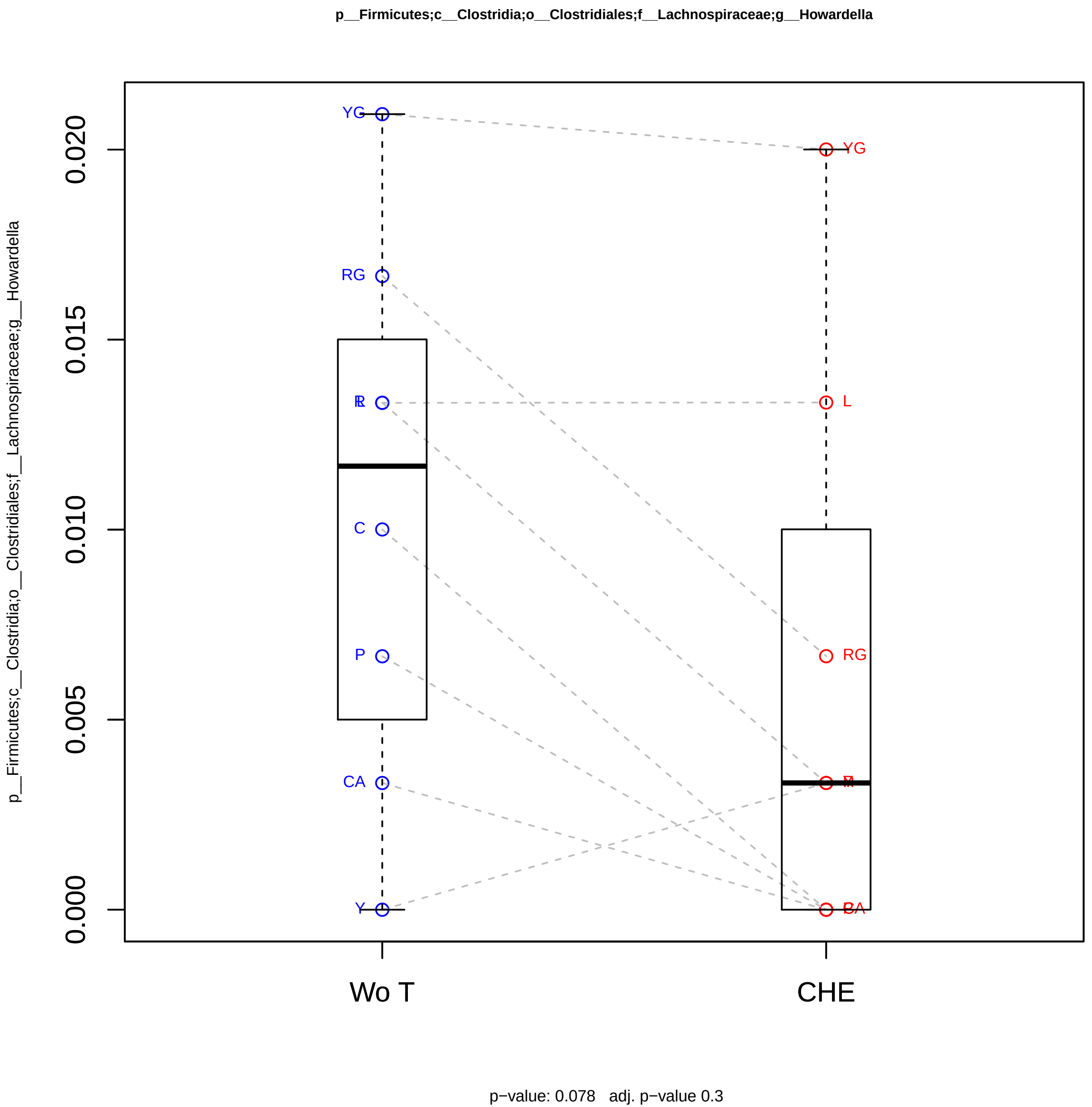
p__Firmicutes;c__Clostridia;o__Clostridiales;f__Lachnospiraceae;g__Howardella
p__Firmicutes;c__Clostridia;o__Clostridiales;f__Lachnospiraceae;g__Howardella
0.000
0.005
0.010
0.015
0.020
YG
RG
C
P
CA
Y
L
Wo T
CHE
p−value: 0.078   adj. p−value 0.3

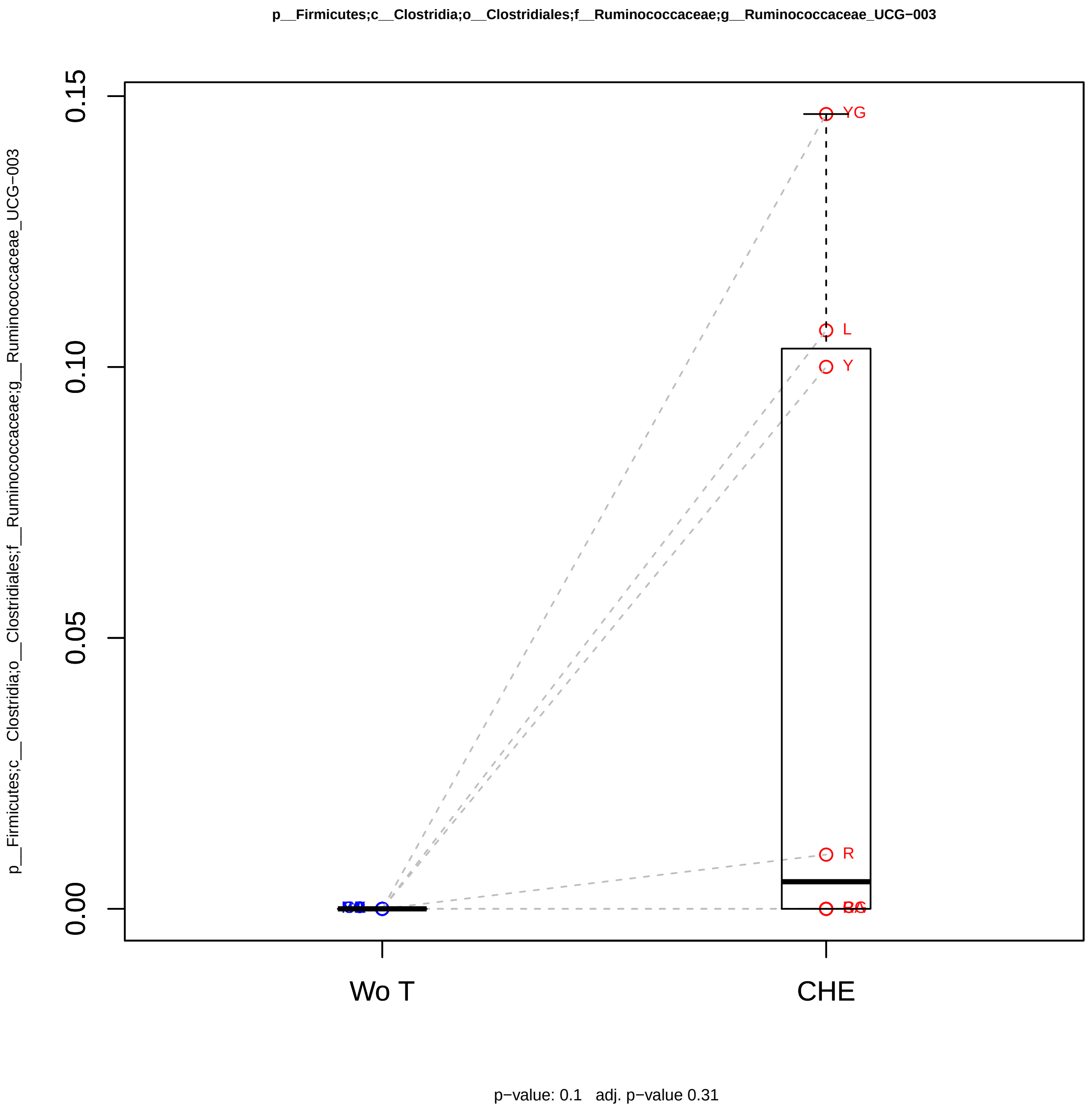
p__Firmicutes;c__Clostridia;o__Clostridiales;f__Ruminococcaceae;g__Ruminococcaceae_UCG−003
p__Firmicutes;c__Clostridia;o__Clostridiales;f__Ruminococcaceae;g__Ruminococcaceae_UCG−003
0.00
0.05
0.10
0.15
YG
L
Y
R
Wo T
CHE
p−value: 0.1 adj. p−value 0.31

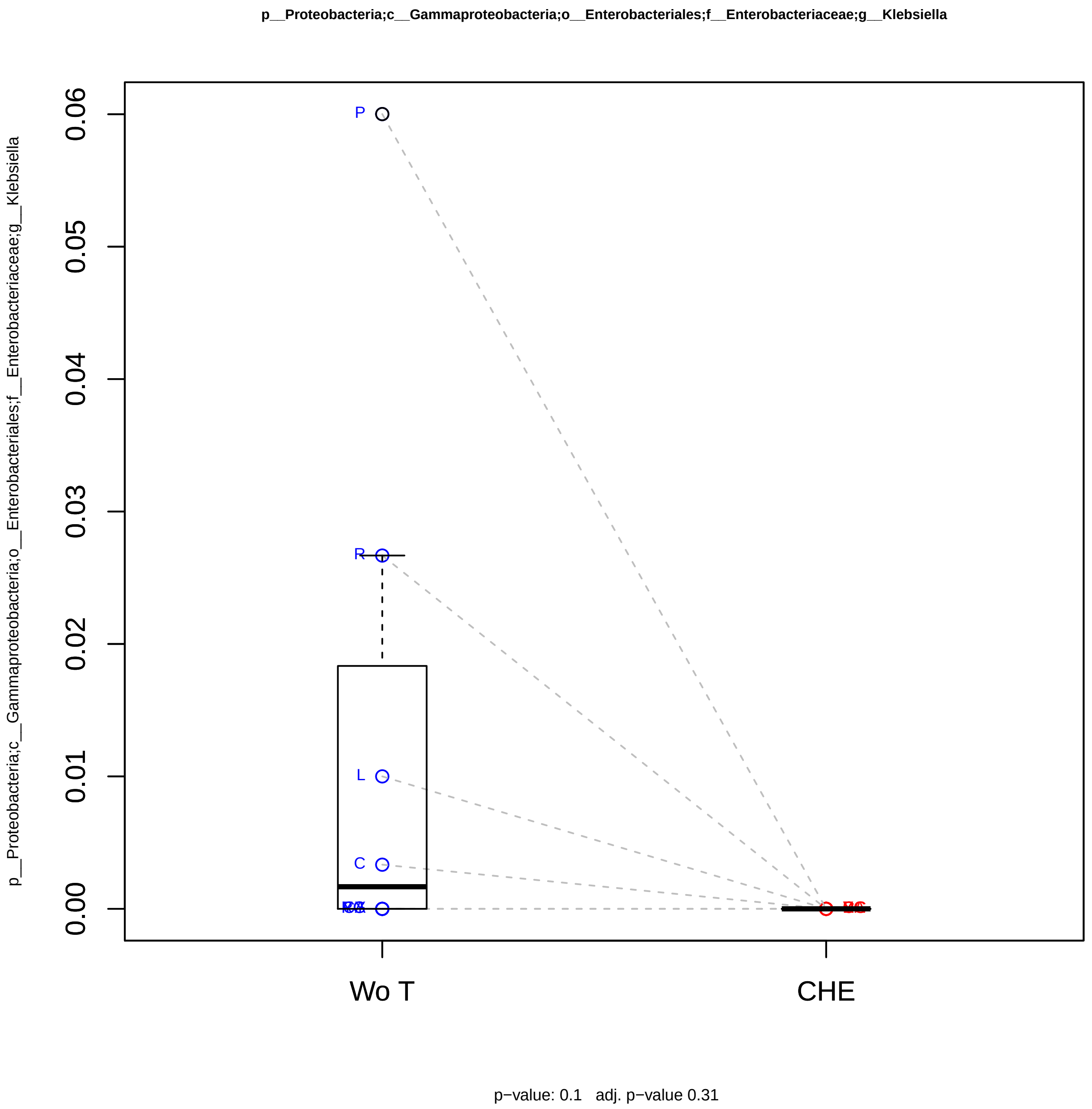
p__Proteobacteria;c__Gammaproteobacteria;o__Enterobacteriales;f__Enterobacteriaceae;g__Klebsiella
p__Proteobacteria;c__Gammaproteobacteria;o__Enterobacteriales;f__Enterobacteriaceae;g__Klebsiella
0.00
0.01
0.02
0.03
0.04
0.05
0.06
P
R
L
C
Wo T
CHE
p−value: 0.1 adj. p−value 0.31

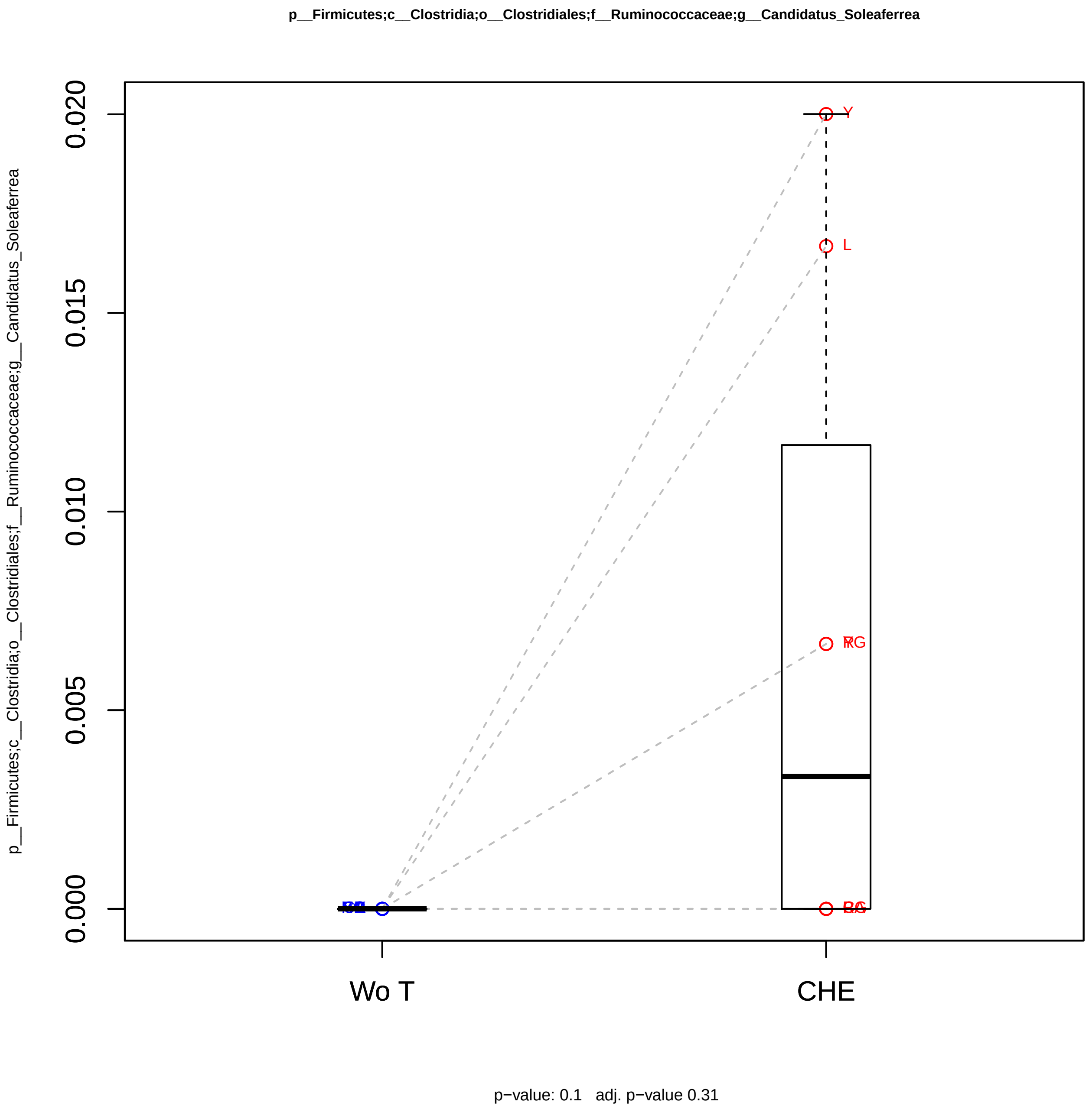
p__Firmicutes;c__Clostridia;o__Clostridiales;f__Ruminococcaceae;g__Candidatus_Soleaferrea
p__Firmicutes;c__Clostridia;o__Clostridiales;f__Ruminococcaceae;g__Candidatus_Soleaferrea
0.000
0.005
0.010
0.015
0.020
Y
L
Wo T
CHE
p−value: 0.1 adj. p−value 0.31

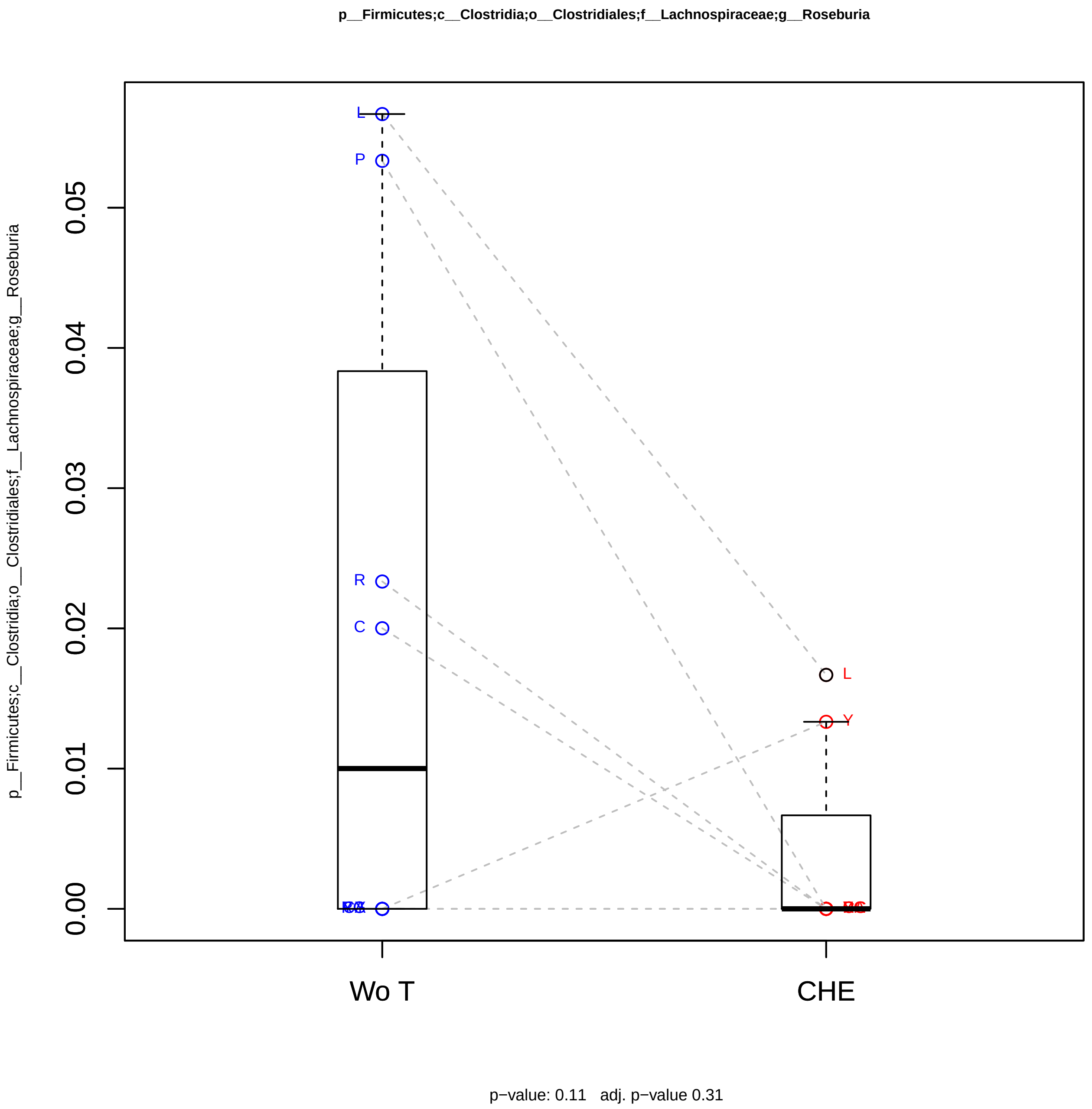
p__Firmicutes;c__Clostridia;o__Clostridiales;f__Lachnospiraceae;g__Roseburia
p__Firmicutes;c__Clostridia;o__Clostridiales;f__Lachnospiraceae;g__Roseburia
0.00
0.01
0.02
0.03
0.04
0.05
L
P
R
C
L
Y
Wo T
CHE
p−value: 0.11 adj. p−value 0.31

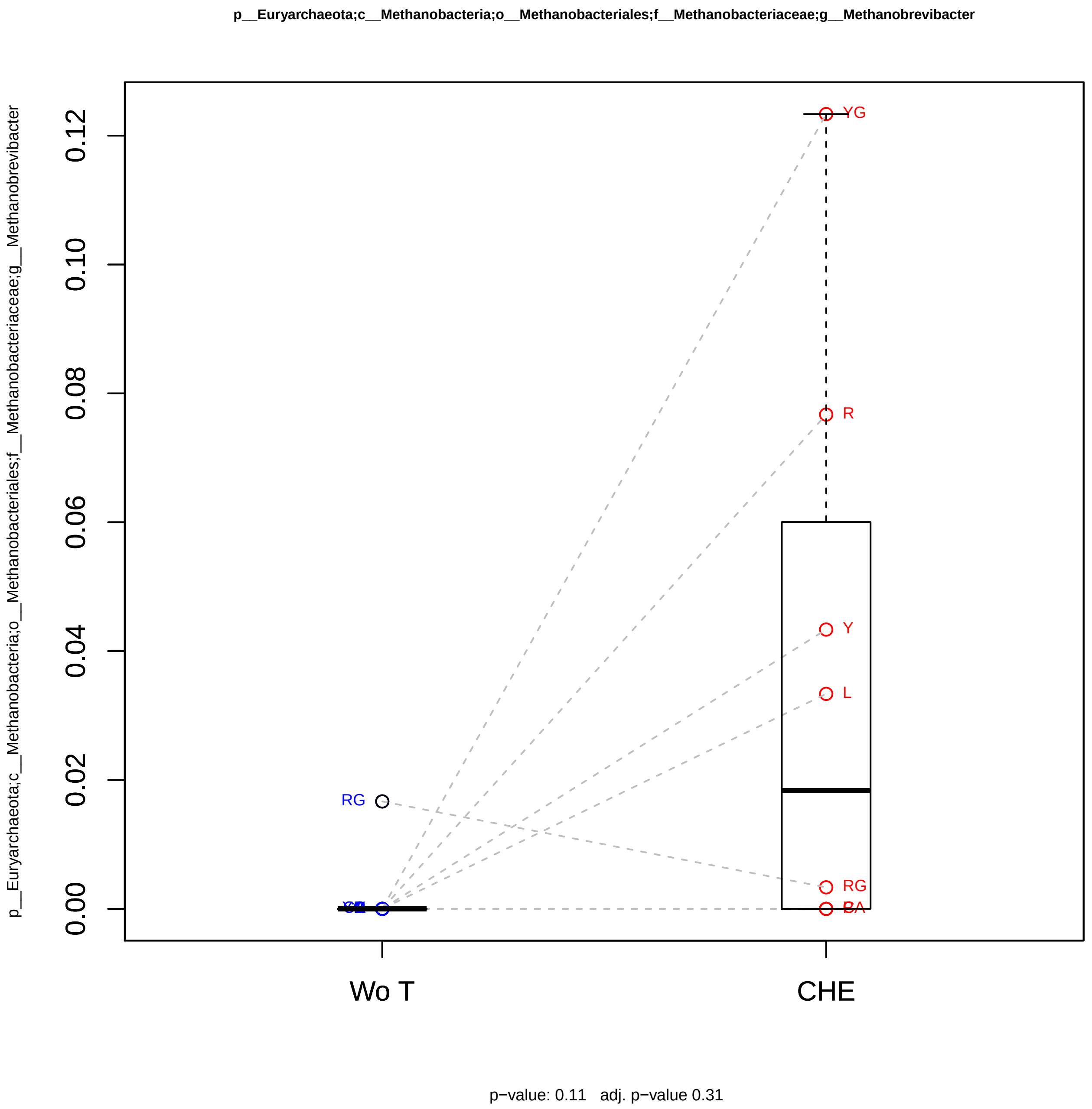
p__Euryarchaeota;c__Methanobacteria;o__Methanobacteriales;f__Methanobacteriaceae;g__Methanobrevibacter
p__Euryarchaeota;c__Methanobacteria;o__Methanobacteriales;f__Methanobacteriaceae;g__Methanobrevibacter
0.12
0.10
0.08
0.06
0.04
0.02
0.00
YG
R
Y
L
RG
RG
Wo T
CHE
p−value: 0.11 adj. p−value 0.31

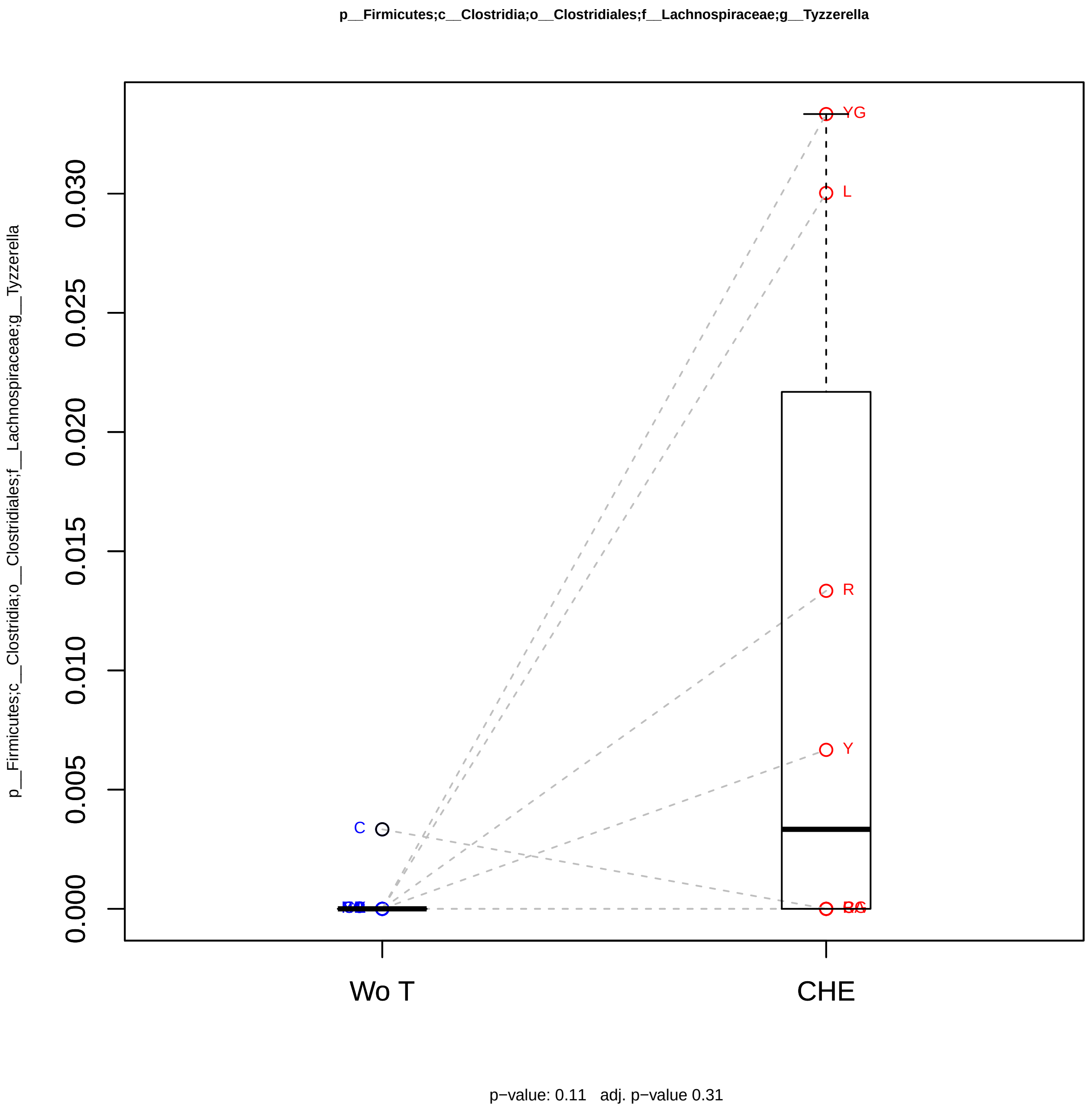
p__Firmicutes;c__Clostridia;o__Clostridiales;f__Lachnospiraceae;g__Tyzzerella
p__Firmicutes;c__Clostridia;o__Clostridiales;f__Lachnospiraceae;g__Tyzzerella
0.000
0.005
0.010
0.015
0.020
0.025
0.030
YG
L
R
Y
C
Wo T
CHE
p−value: 0.11 adj. p−value 0.31

p__Firmicutes;c__Clostridia;o__Clostridiales;f__Ruminococcaceae;g__Oscillibacter

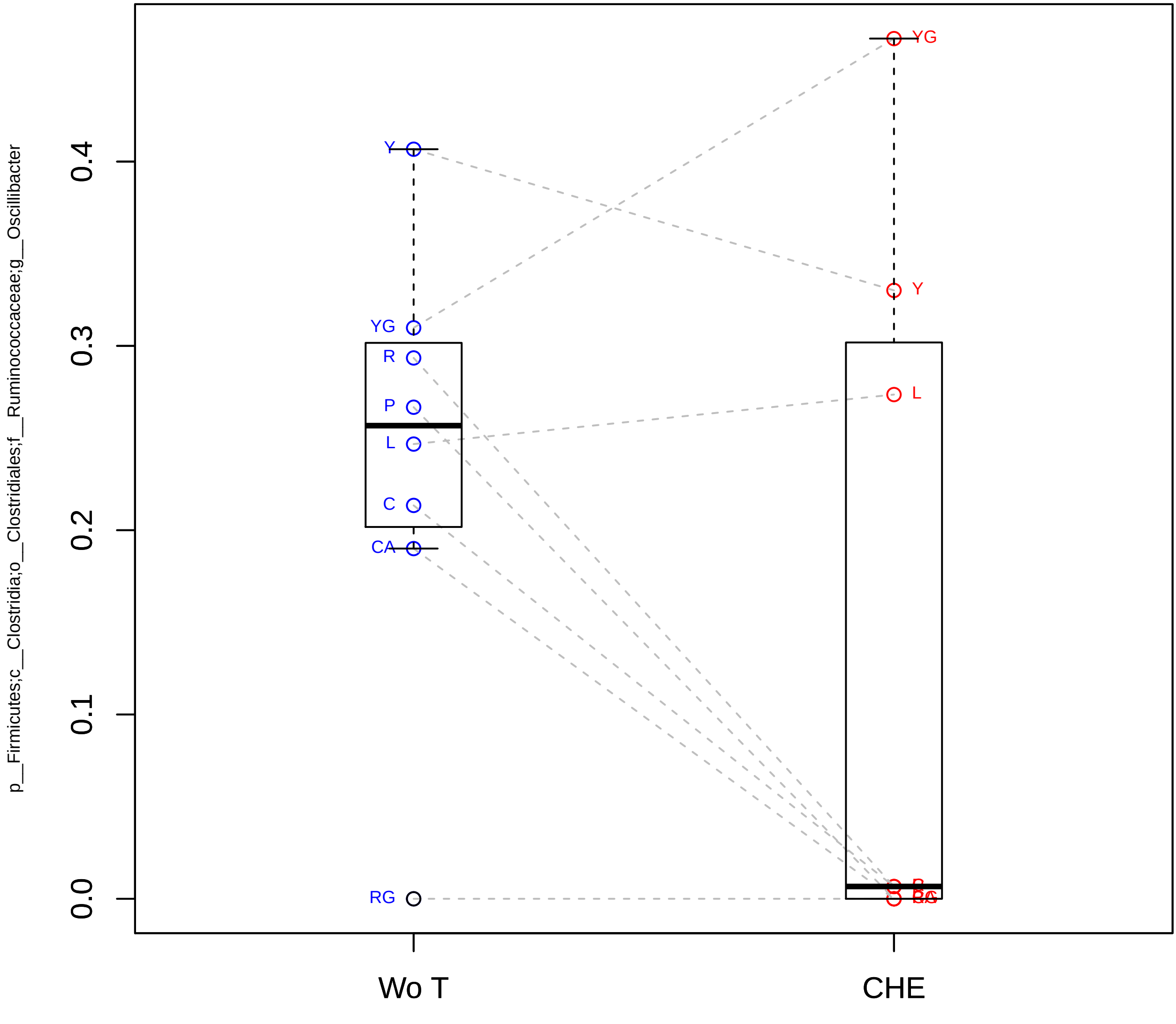


p−value: 0.11 adj. p−value 0.31

p__Firmicutes;c__Clostridia;o__Clostridiales;f__Ruminococcaceae;g__Ruminiclostridium_9

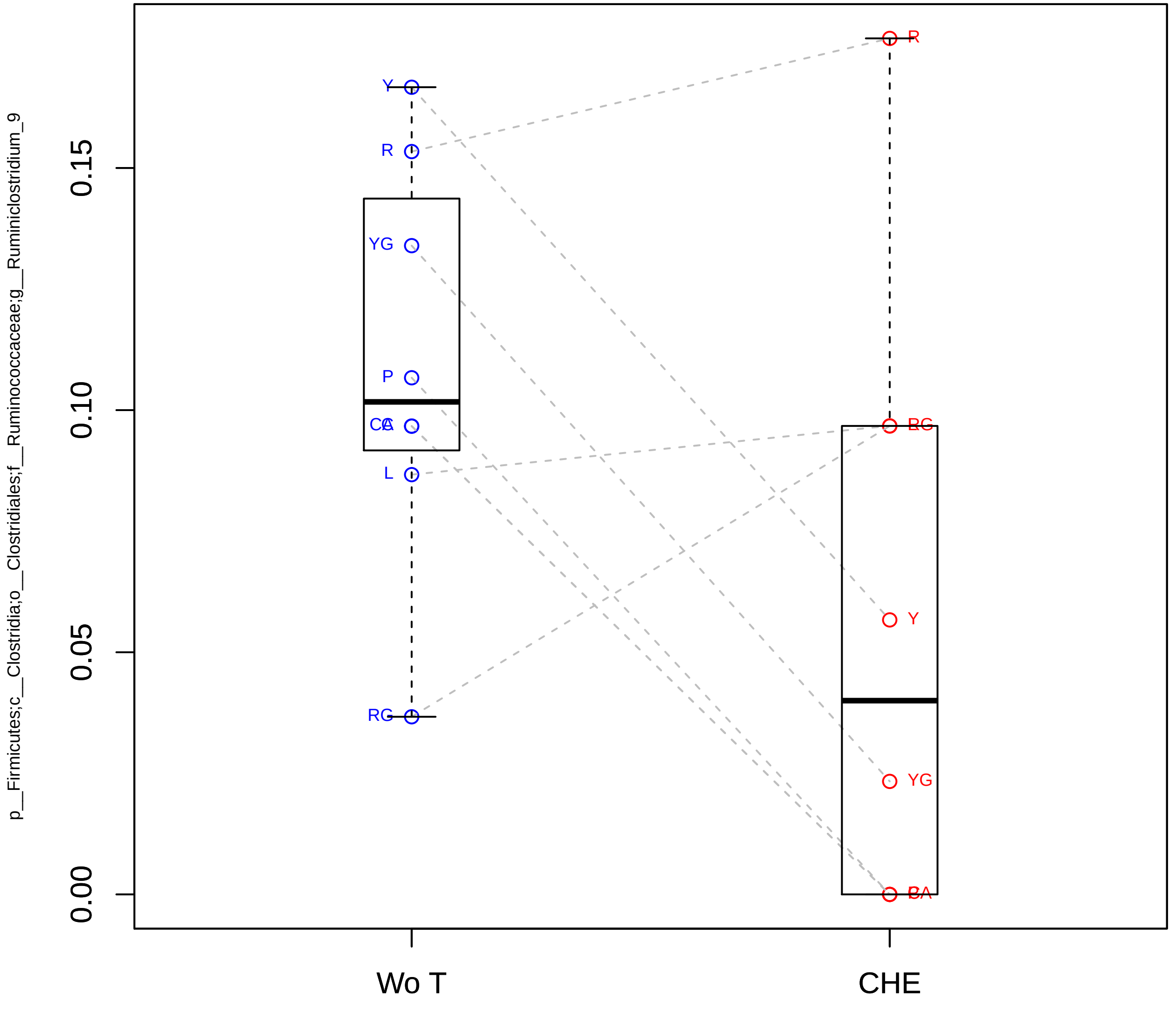


p−value: 0.11   adj. p−value 0.31

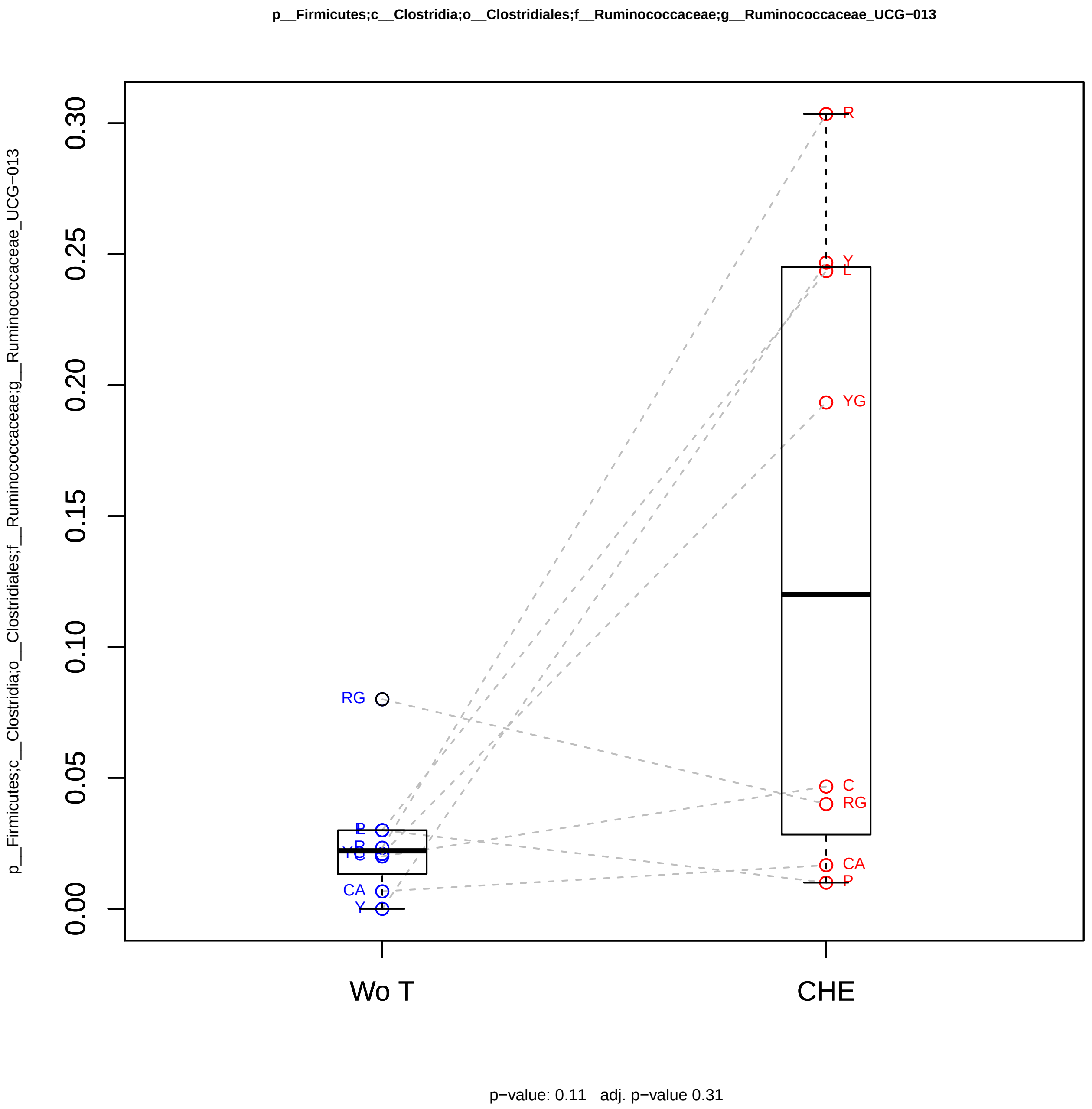
p__Firmicutes;c__Clostridia;o__Clostridiales;f__Ruminococcaceae;g__Ruminococcaceae_UCG−013
p__Firmicutes;c__Clostridia;o__Clostridiales;f__Ruminococcaceae;g__Ruminococcaceae_UCG−013
0.00
0.05
0.10
0.15
0.20
0.25
0.30
R
Y
L
YG
C
RG
CA
P
RG
CA
Y
Wo T
CHE
p−value: 0.11 adj. p−value 0.31

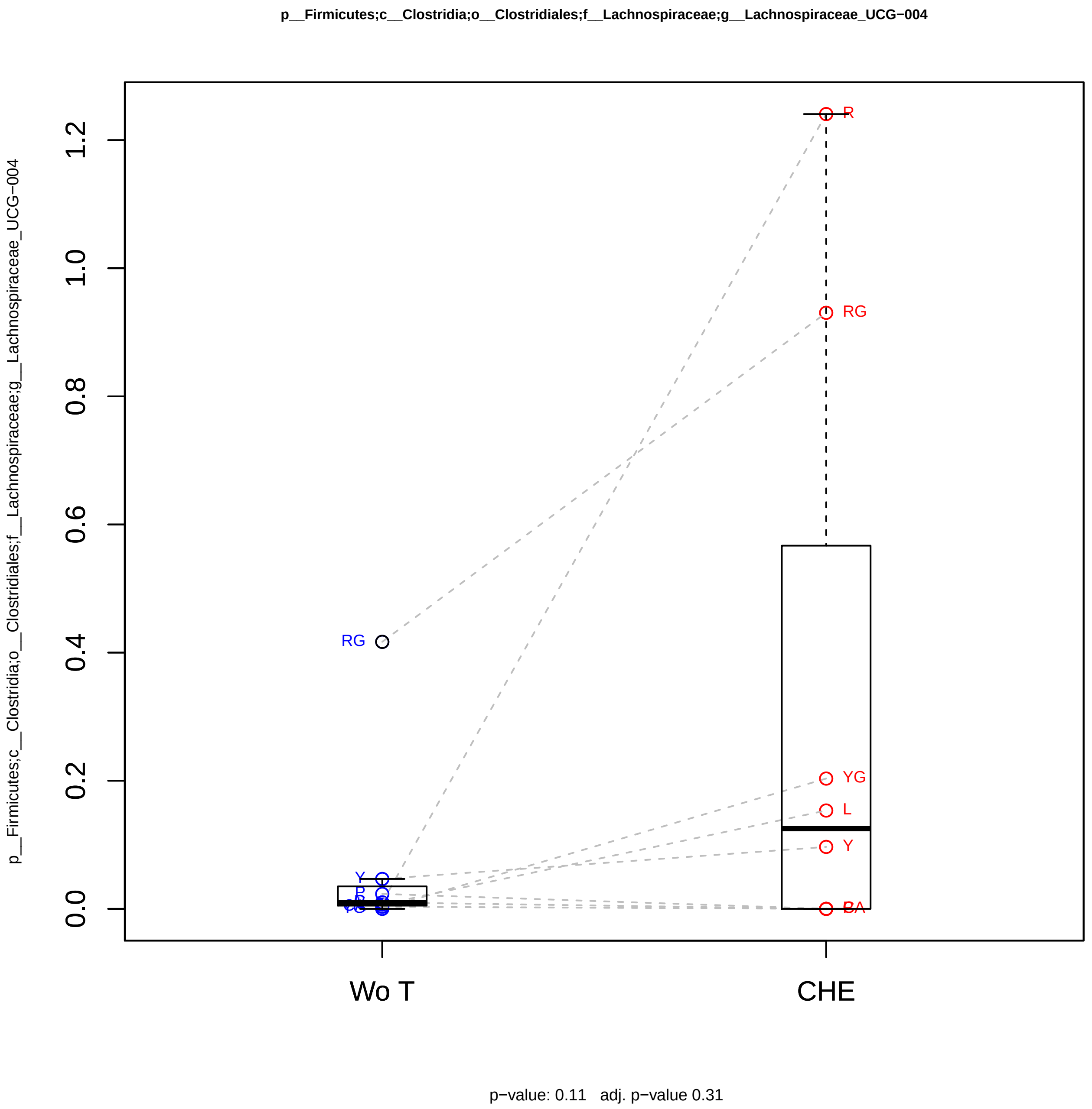
p__Firmicutes;c__Clostridia;o__Clostridiales;f__Lachnospiraceae;g__Lachnospiraceae_UCG−004
p__Firmicutes;c__Clostridia;o__Clostridiales;f__Lachnospiraceae;g__Lachnospiraceae_UCG−004
0.0
0.2
0.4
0.6
0.8
1.0
1.2
RG
Y
P
R
RG
YG
L
Y
Wo T
CHE
p−value: 0.11 adj. p−value 0.31

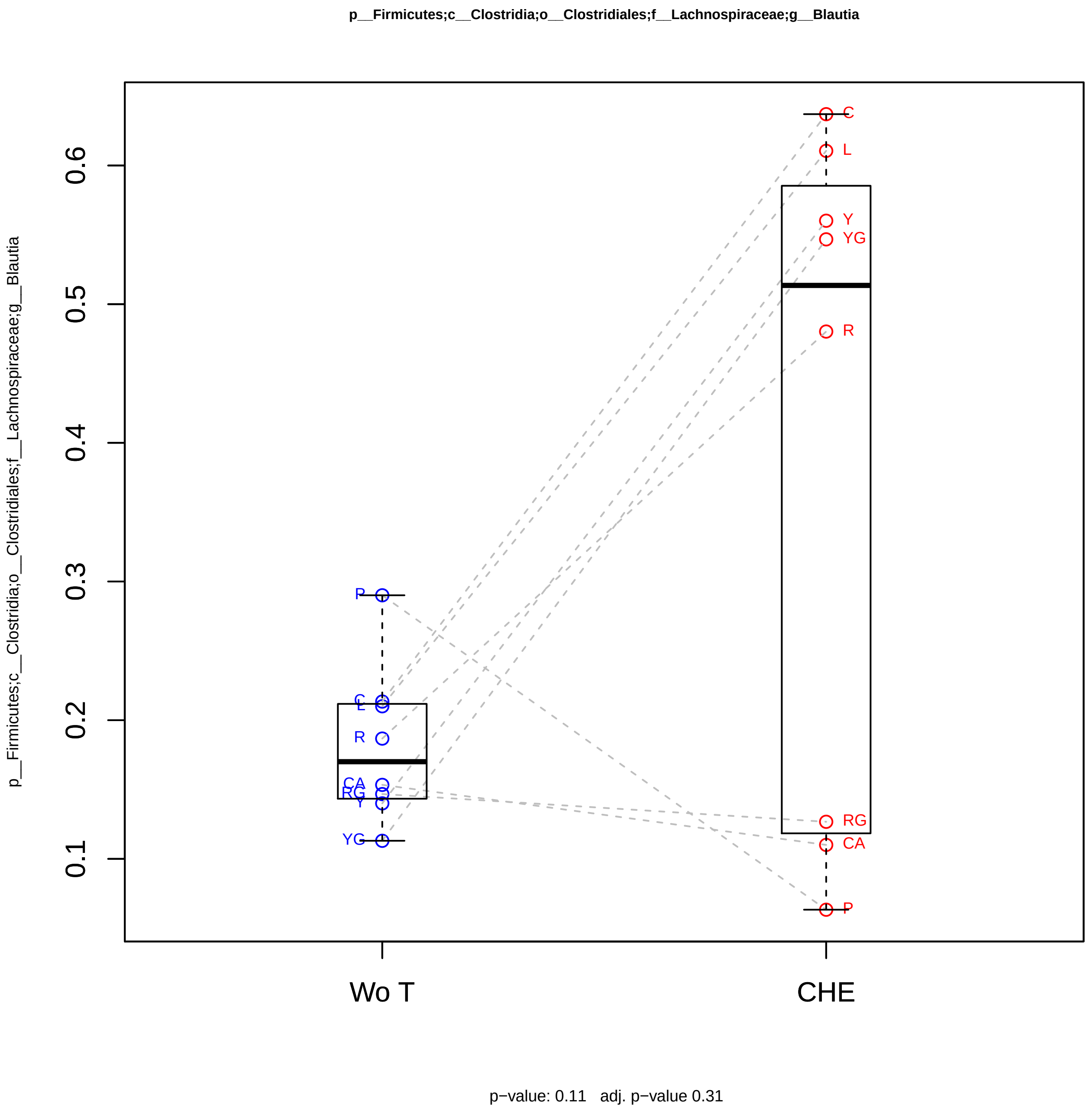
p__Firmicutes;c__Clostridia;o__Clostridiales;f__Lachnospiraceae;g__Blautia
p__Firmicutes;c__Clostridia;o__Clostridiales;f__Lachnospiraceae;g__Blautia
0.1
0.2
0.3
0.4
0.5
0.6
P
C
L
R
CA
RG
Y
YG
C
L
Y
YG
R
RG
CA
P
Wo T
CHE
p−value: 0.11 adj. p−value 0.31

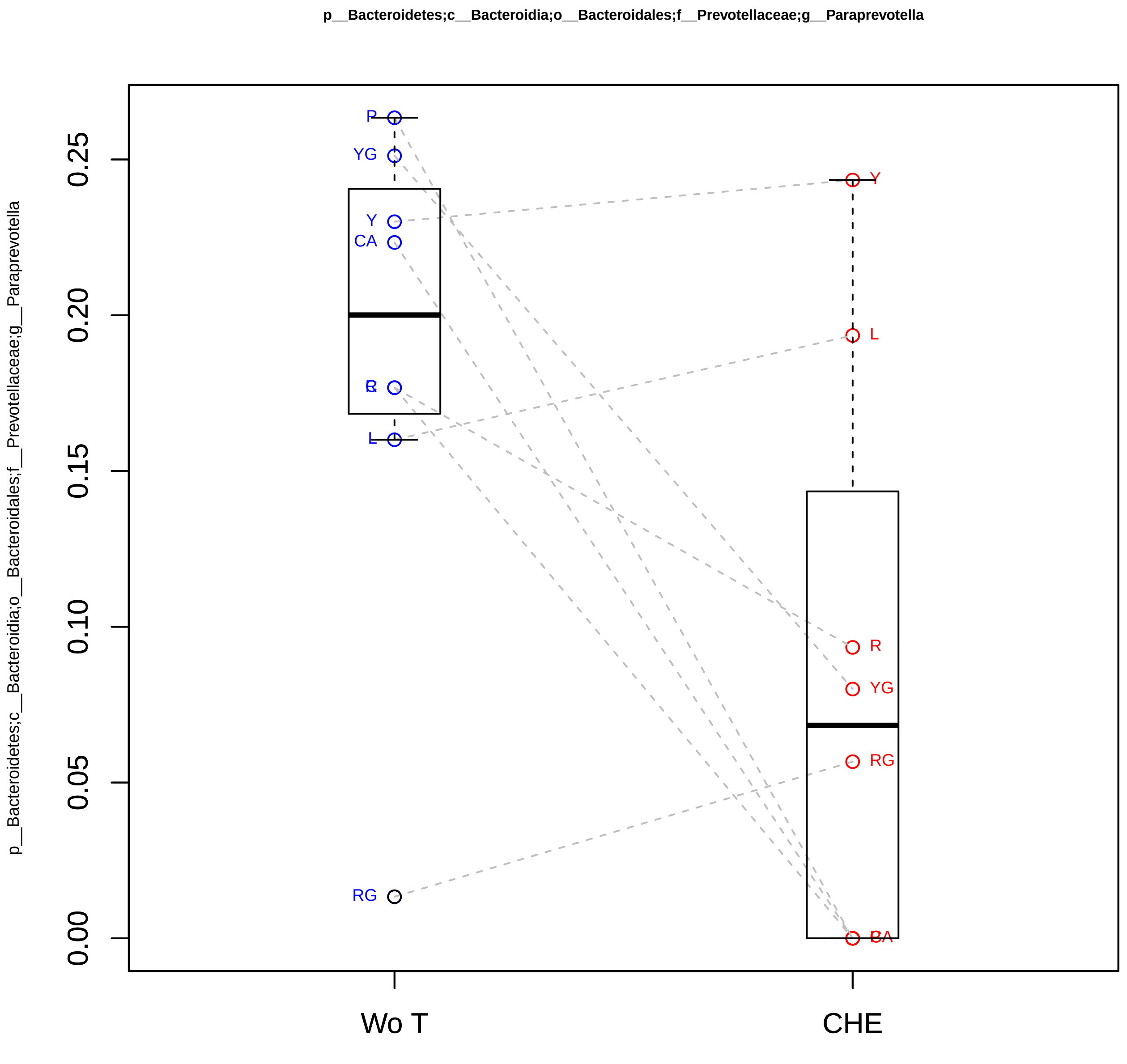

p−value: 0.11 adj. p−value 0.31

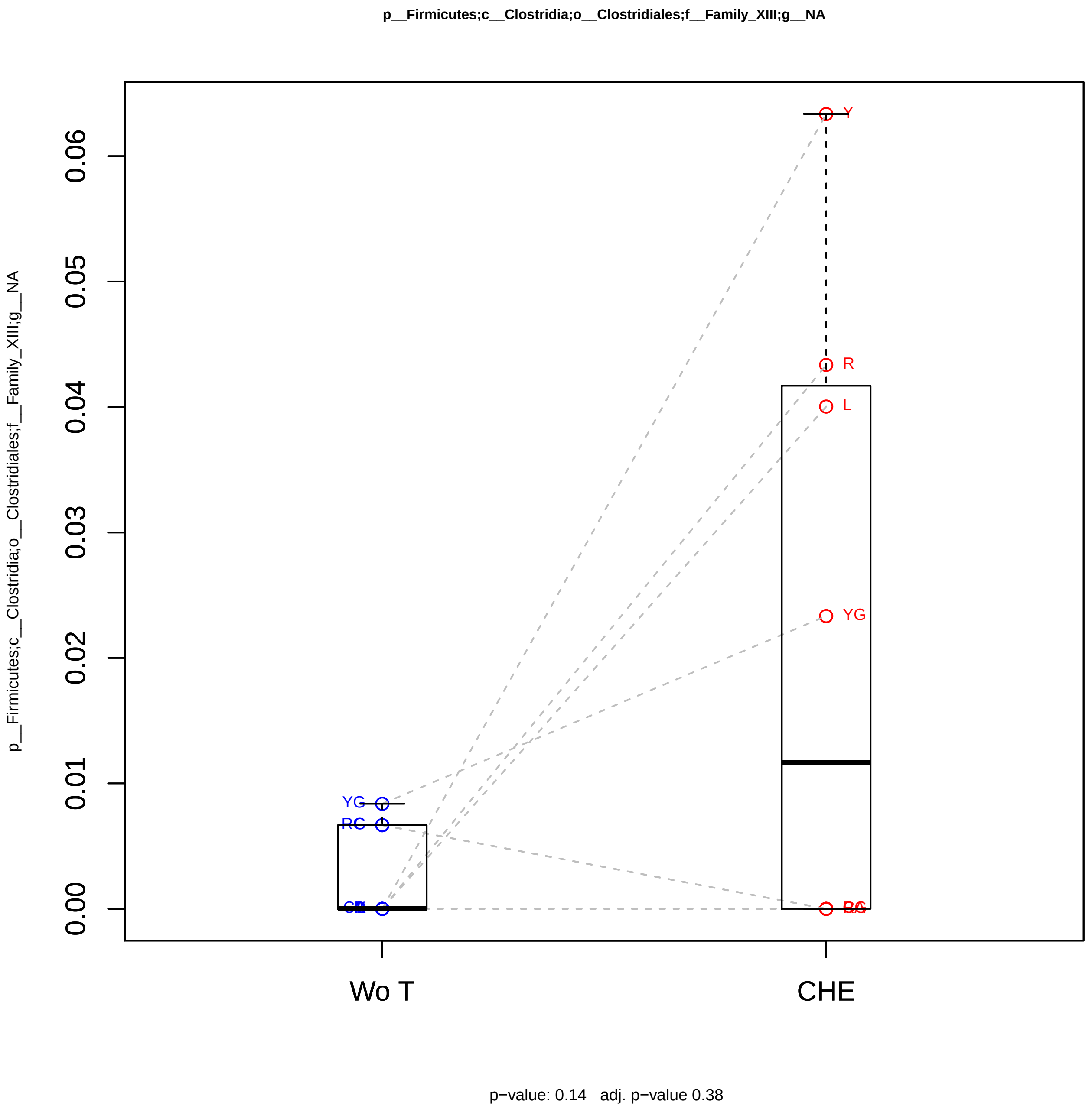
p__Firmicutes;c__Clostridia;o__Clostridiales;f__Family_XIII;g__NA
p__Firmicutes;c__Clostridia;o__Clostridiales;f__Family_XIII;g__NA
0.00
0.01
0.02
0.03
0.04
0.05
0.06
Y
R
L
YG
YG
Wo T
CHE
p−value: 0.14 adj. p−value 0.38

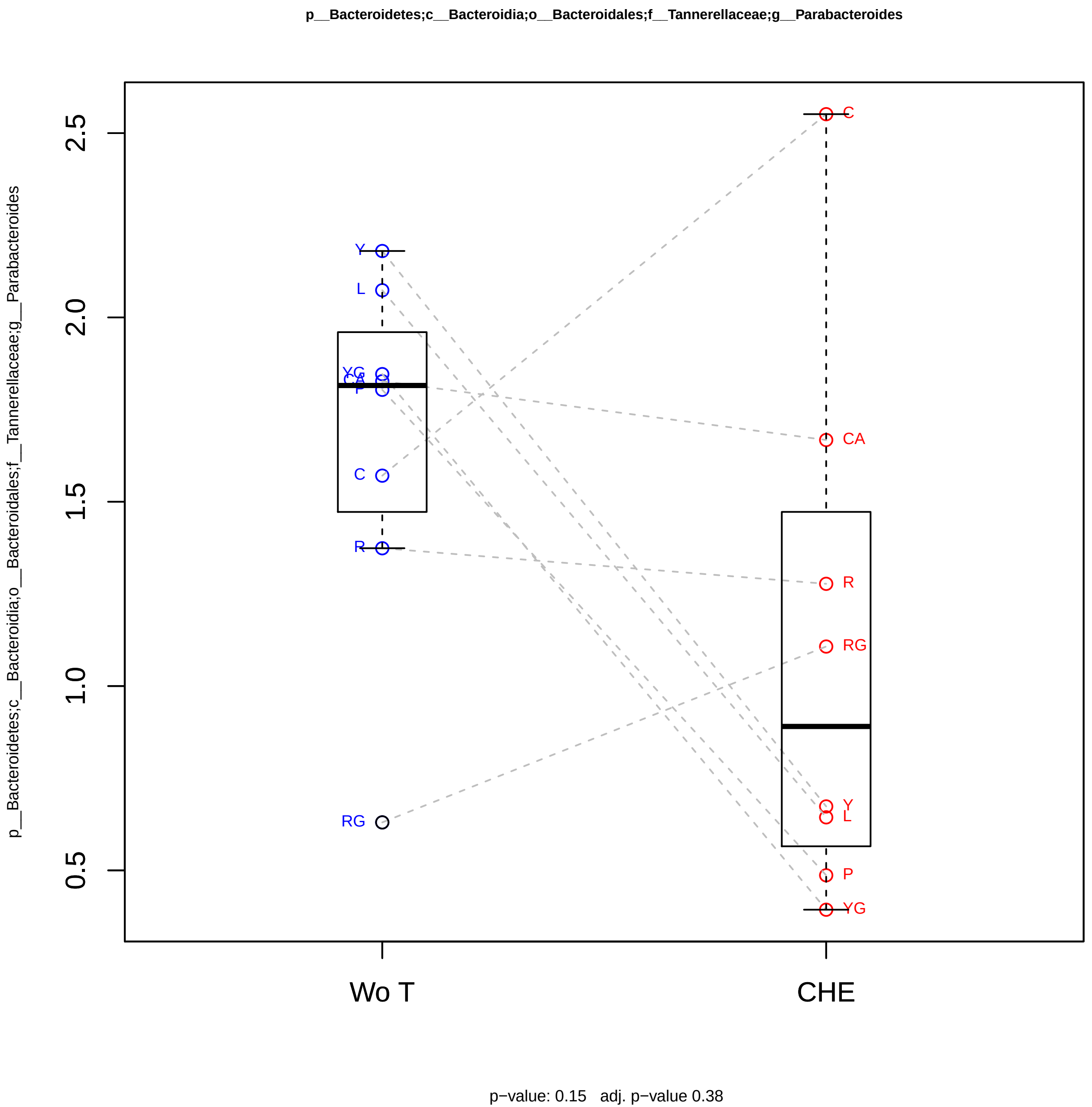

p__Bacteroidetes;c__Bacteroidia;o__Bacteroidales;f__Tannerellaceae;g__Parabacteroides
p__Bacteroidetes;c__Bacteroidia;o__Bacteroidales;f__Tannerellaceae;g__Parabacteroides
0.5
1.0
1.5
2.0
2.5
Y
L
YG
CA
P
C
R
RG
C
CA
R
RG
Y
L
P
YG
Wo T
CHE
p−value: 0.15 adj. p−value 0.38

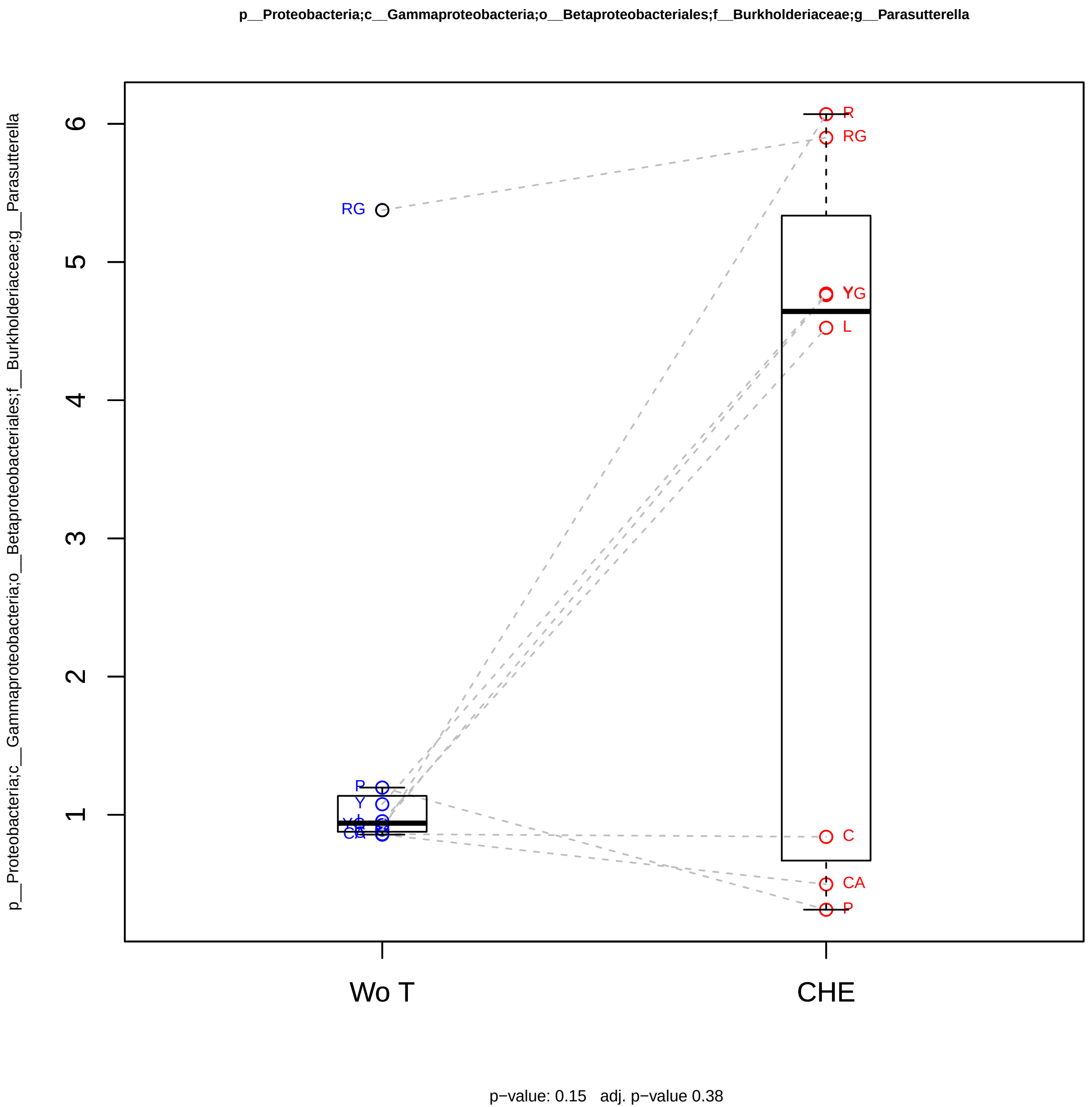

p__Proteobacteria;c__Gammaproteobacteria;o__Betaproteobacteriales;f__Burkholderiaceae;g__Parasutterella
p__Proteobacteria;c__Gammaproteobacteria;o__Betaproteobacteriales;f__Burkholderiaceae;g__Parasutterella
1
2
3
4
5
6
RG
P
Y
R
RG
YG
L
C
CA
P
Wo T
CHE
p−value: 0.15 adj. p−value 0.38

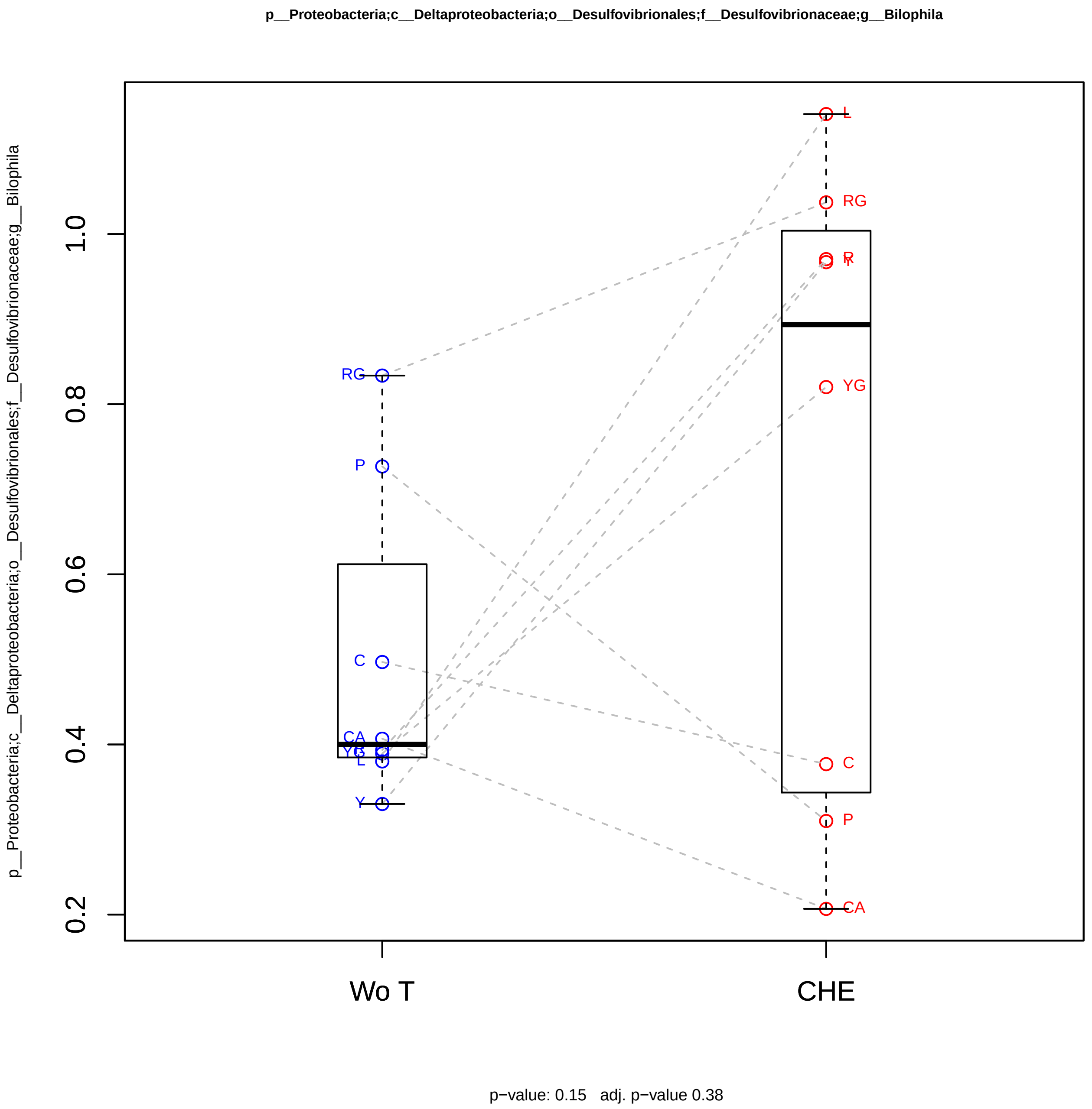

p__Proteobacteria;c__Deltaproteobacteria;o__Desulfovibrionales;f__Desulfovibrionaceae;g__Bilophila
p__Proteobacteria;c__Deltaproteobacteria;o__Desulfovibrionales;f__Desulfovibrionaceae;g__Bilophila
0.2
0.4
0.6
0.8
1.0
Wo T
CHE
RG
P
C
CA
YG
L
Y
RG
R
Y
YG
C
P
CA
L
p−value: 0.15 adj. p−value 0.38

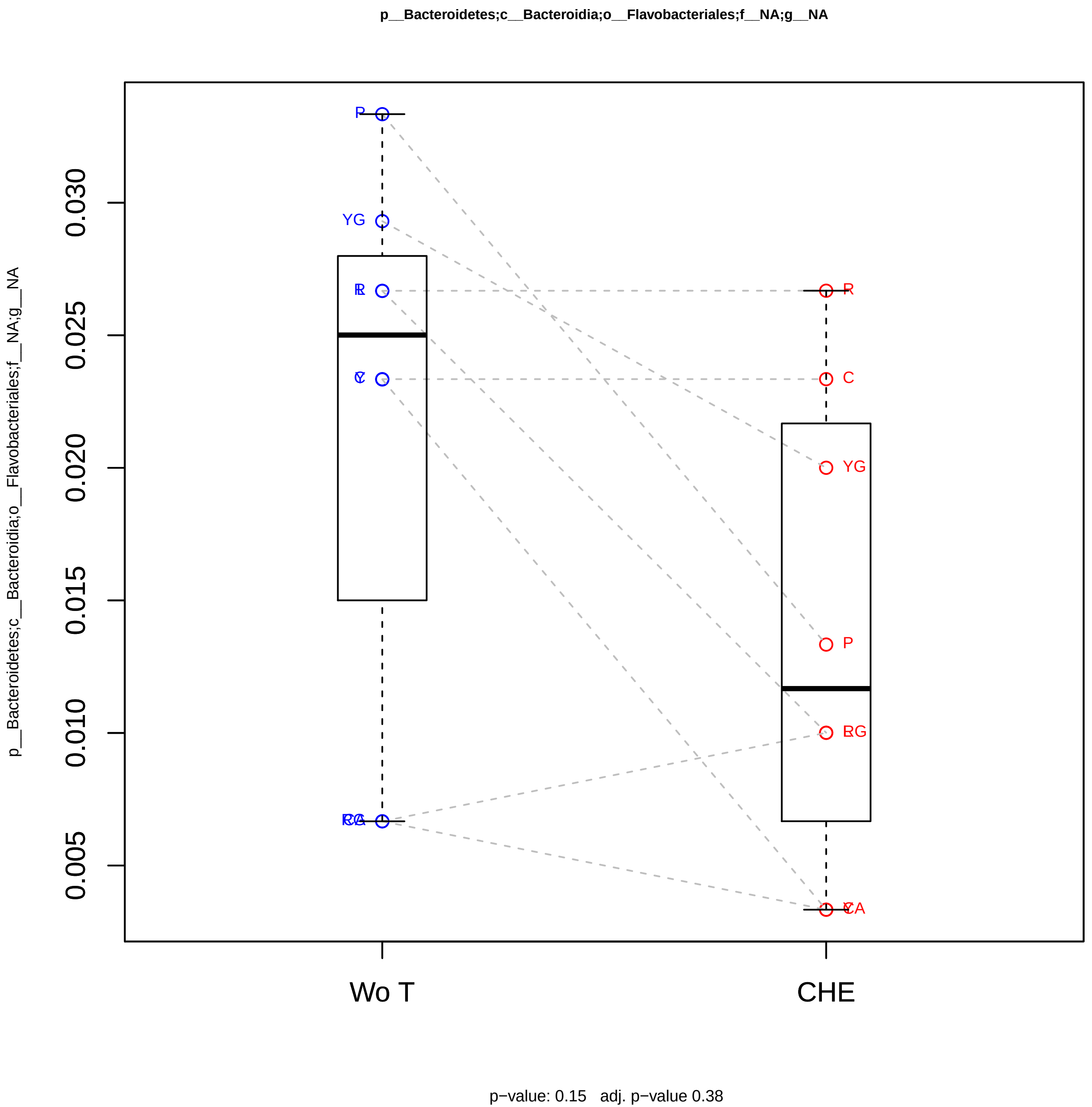
p__Bacteroidetes;c__Bacteroidia;o__Flavobacteriales;f__NA;g__NA
p__Bacteroidetes;c__Bacteroidia;o__Flavobacteriales;f__NA;g__NA
0.005
0.010
0.015
0.020
0.025
0.030
Wo T
CHE
P
YG
R
C
YG
P
RG
CA
p−value: 0.15 adj. p−value 0.38

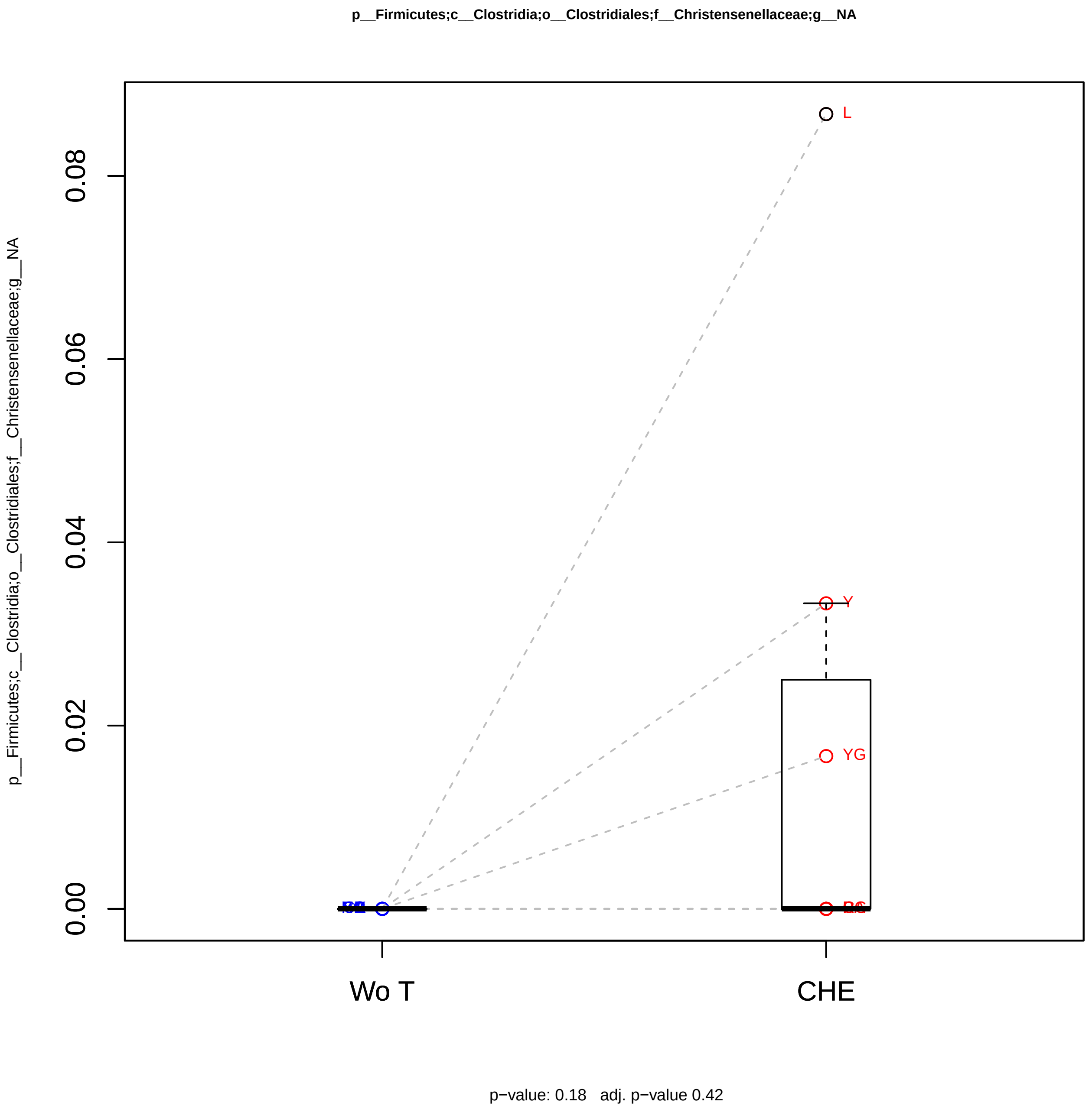
p__Firmicutes;c__Clostridia;o__Clostridiales;f__Christensenellaceae;g__NA
p__Firmicutes;c__Clostridia;o__Clostridiales;f__Christensenellaceae;g__NA
0.00
0.02
0.04
0.06
0.08
L
Y
YG
Wo T
CHE
p−value: 0.18   adj. p−value 0.42

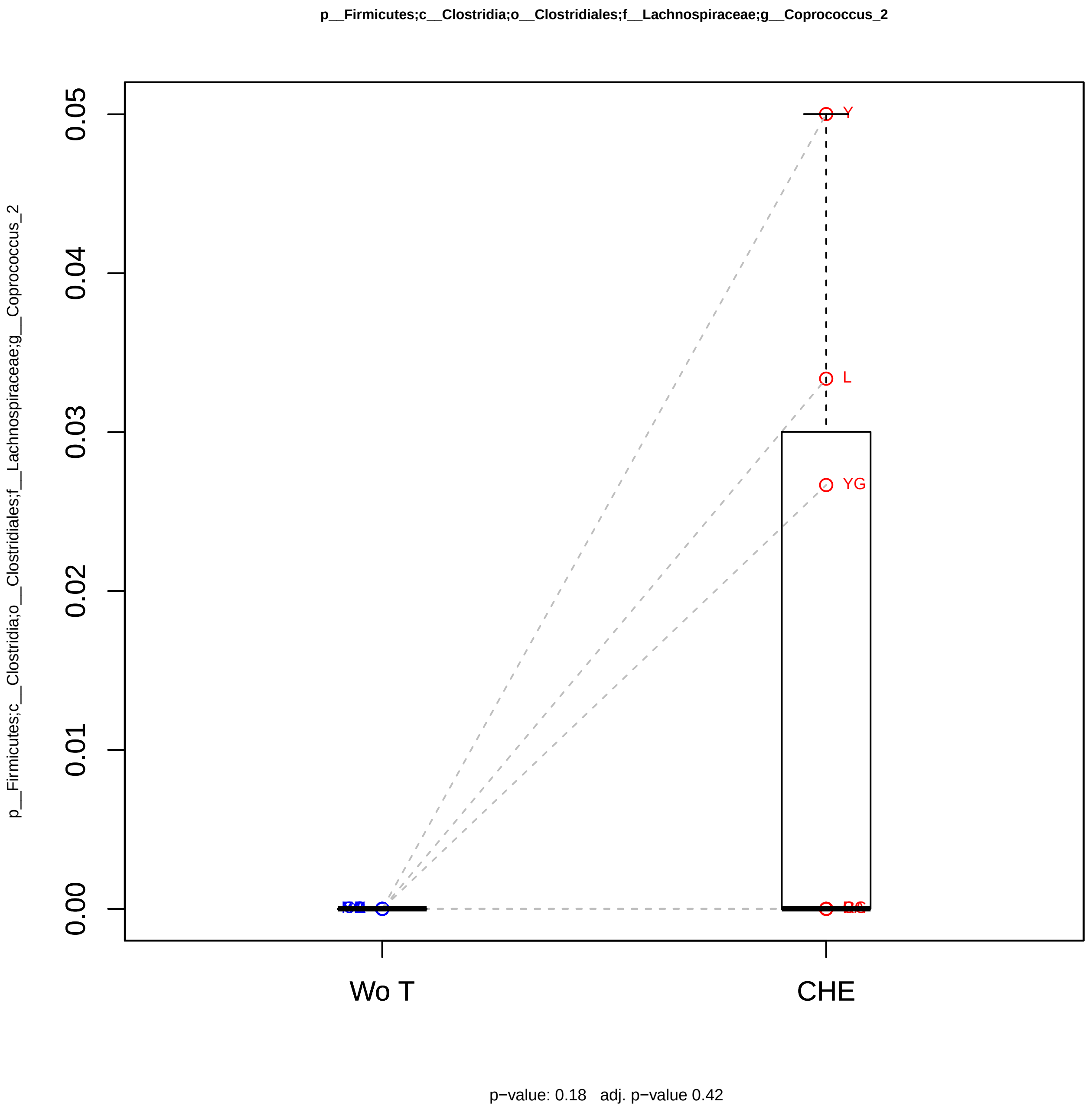
p__Firmicutes;c__Clostridia;o__Clostridiales;f__Lachnospiraceae;g__Coprococcus_2
p__Firmicutes;c__Clostridia;o__Clostridiales;f__Lachnospiraceae;g__Coprococcus_2
0.00
0.01
0.02
0.03
0.04
0.05
Y
L
YG
Wo T
CHE
p−value: 0.18   adj. p−value 0.42

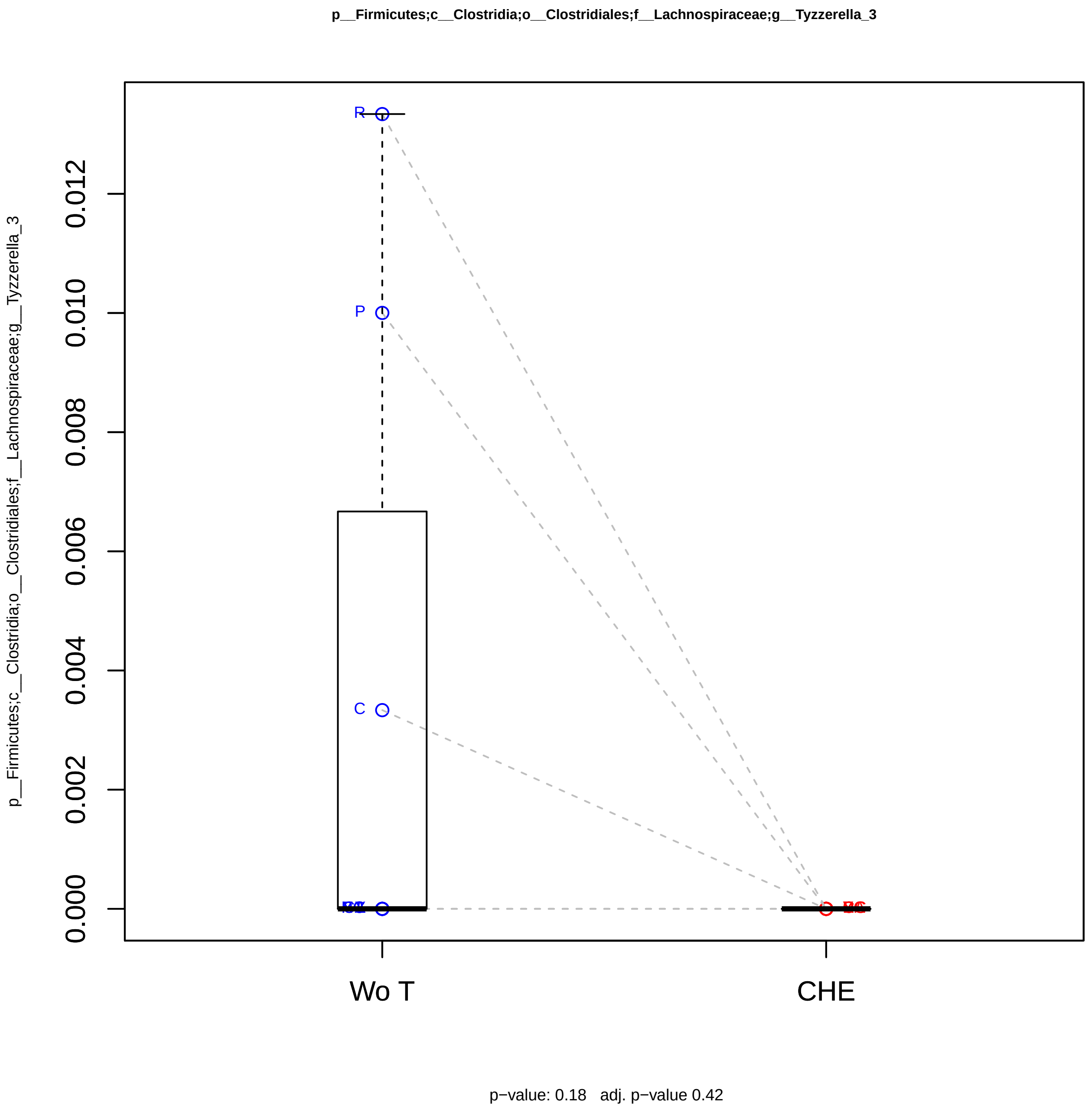

p__Firmicutes;c__Clostridia;o__Clostridiales;f__Lachnospiraceae;g__Tyzzerella_3
p__Firmicutes;c__Clostridia;o__Clostridiales;f__Lachnospiraceae;g__Tyzzerella_3
0.000
0.002
0.004
0.006
0.008
0.010
0.012
R
P
C
Wo T
CHE
p−value: 0.18 adj. p−value 0.42

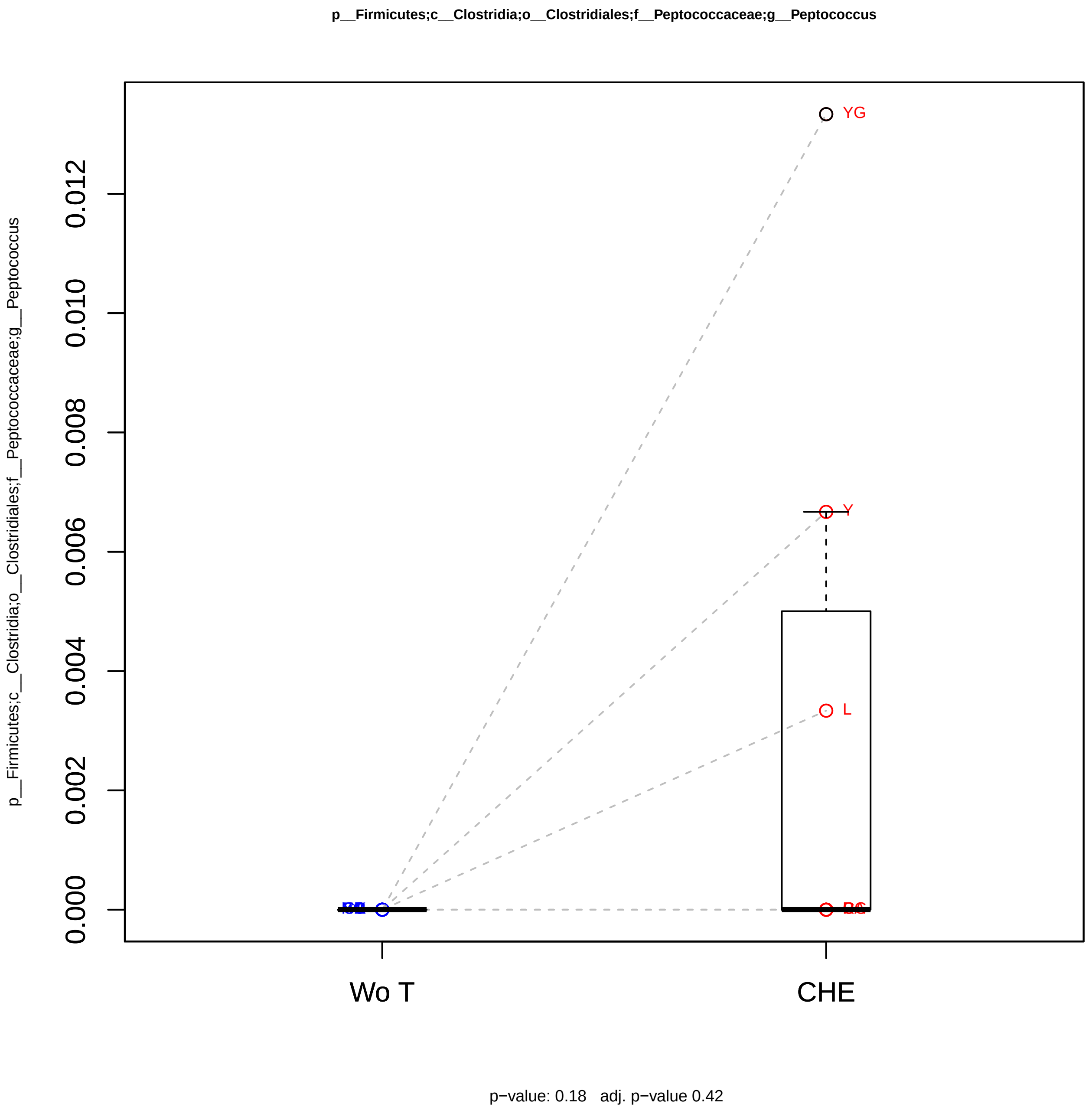
p__Firmicutes;c__Clostridia;o__Clostridiales;f__Peptococcaceae;g__Peptococcus
p__Firmicutes;c__Clostridia;o__Clostridiales;f__Peptococcaceae;g__Peptococcus
0.000
0.002
0.004
0.006
0.008
0.010
0.012
YG
Y
L
Wo T
CHE
p−value: 0.18   adj. p−value 0.42

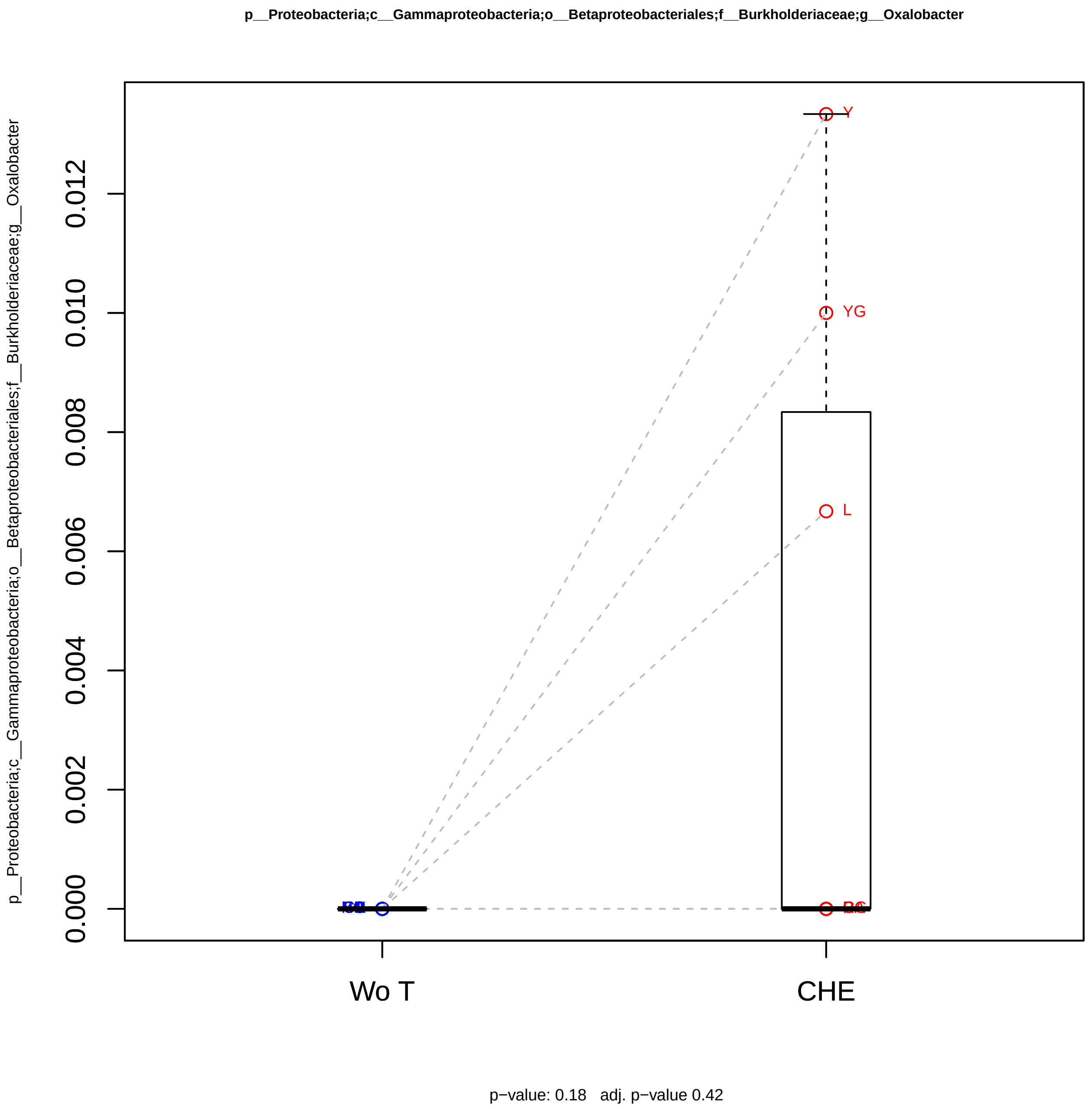

p__Proteobacteria;c__Gammaproteobacteria;o__Betaproteobacteriales;f__Burkholderiaceae;g__Oxalobacter
p__Proteobacteria;c__Gammaproteobacteria;o__Betaproteobacteriales;f__Burkholderiaceae;g__Oxalobacter
0.000
0.002
0.004
0.006
0.008
0.010
0.012
Y
YG
L
Wo T
CHE
p−value: 0.18 adj. p−value 0.42

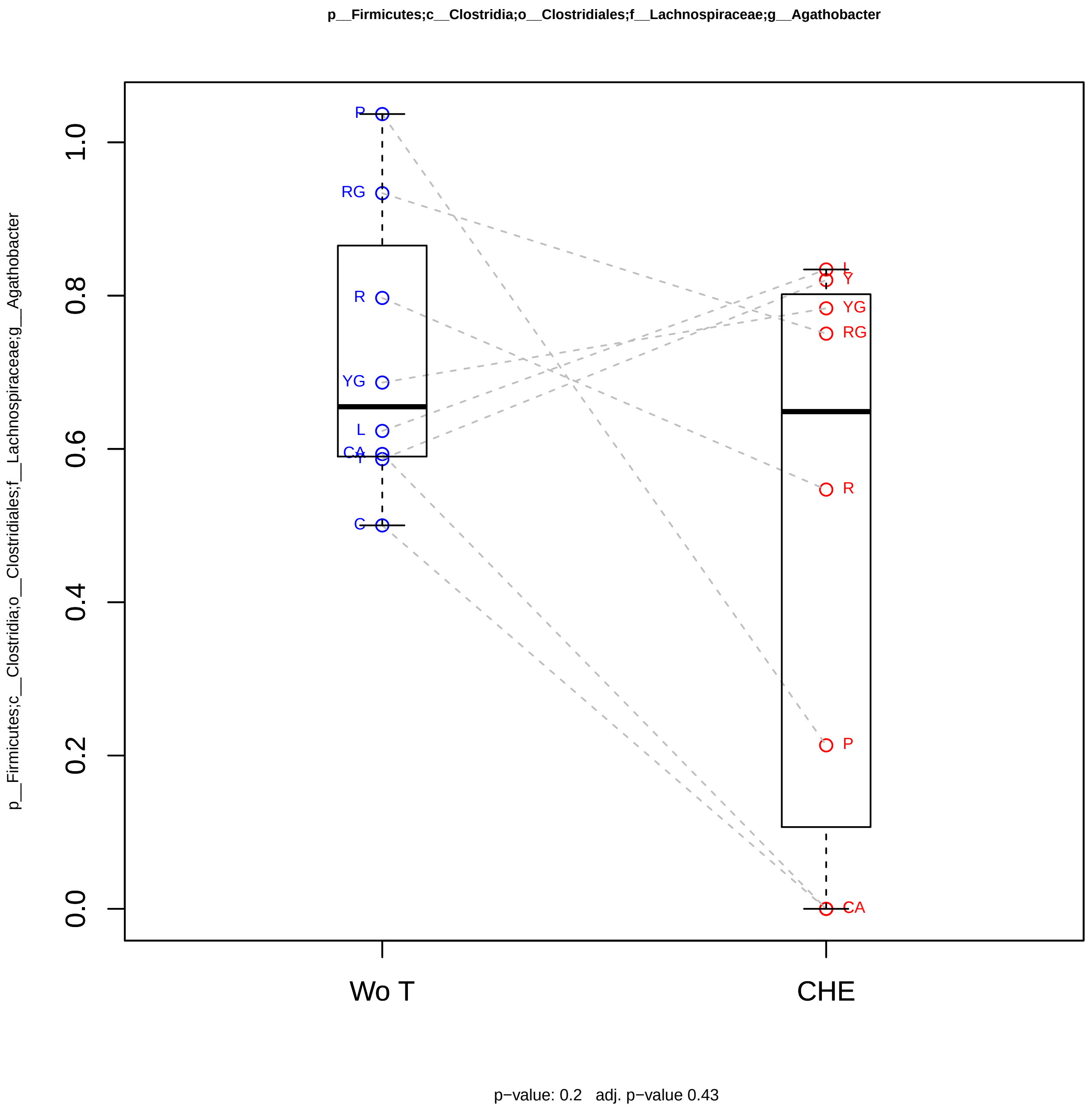
p__Firmicutes;c__Clostridia;o__Clostridiales;f__Lachnospiraceae;g__Agathobacter
p__Firmicutes;c__Clostridia;o__Clostridiales;f__Lachnospiraceae;g__Agathobacter
0.0
0.2
0.4
0.6
0.8
1.0
P
RG
R
YG
L
CA
Y
C
L
Y
YG
RG
R
P
CA
Wo T
CHE
p−value: 0.2 adj. p−value 0.43

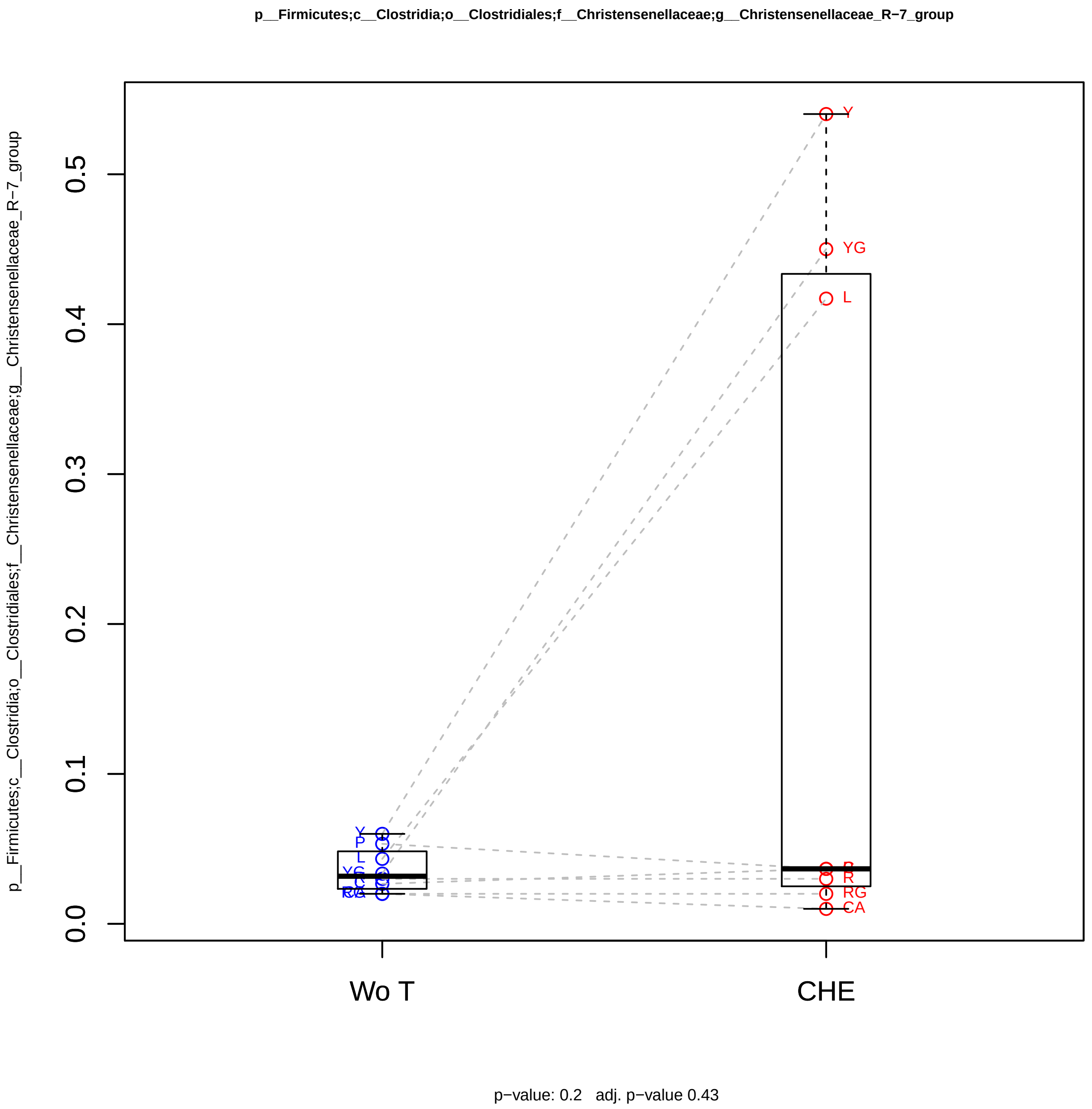

p__Firmicutes;c__Clostridia;o__Clostridiales;f__Christensenellaceae;g__Christensenellaceae_R−7_group
p__Firmicutes;c__Clostridia;o__Clostridiales;f__Christensenellaceae;g__Christensenellaceae_R−7_group
0.0
0.1
0.2
0.3
0.4
0.5
Wo T
CHE
Y
P
L
YG
Y
YG
L
R
RG
CA
p−value: 0.2 adj. p−value 0.43

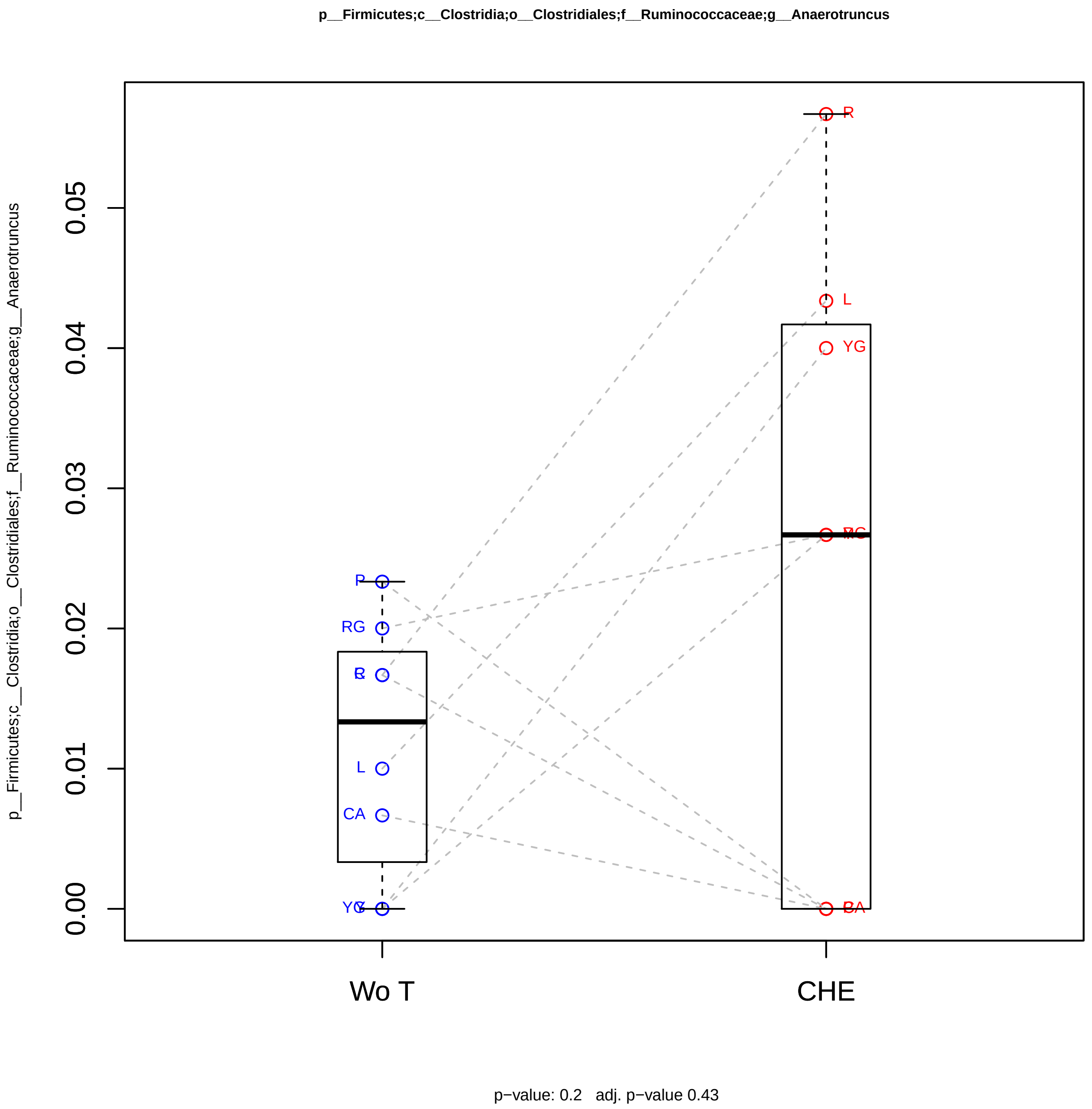

p__Firmicutes;c__Clostridia;o__Clostridiales;f__Ruminococcaceae;g__Anaerotruncus
p__Firmicutes;c__Clostridia;o__Clostridiales;f__Ruminococcaceae;g__Anaerotruncus
0.00
0.01
0.02
0.03
0.04
0.05
P
RG
L
CA
YG
R
L
YG
CA
Wo T
CHE
p−value: 0.2 adj. p−value 0.43

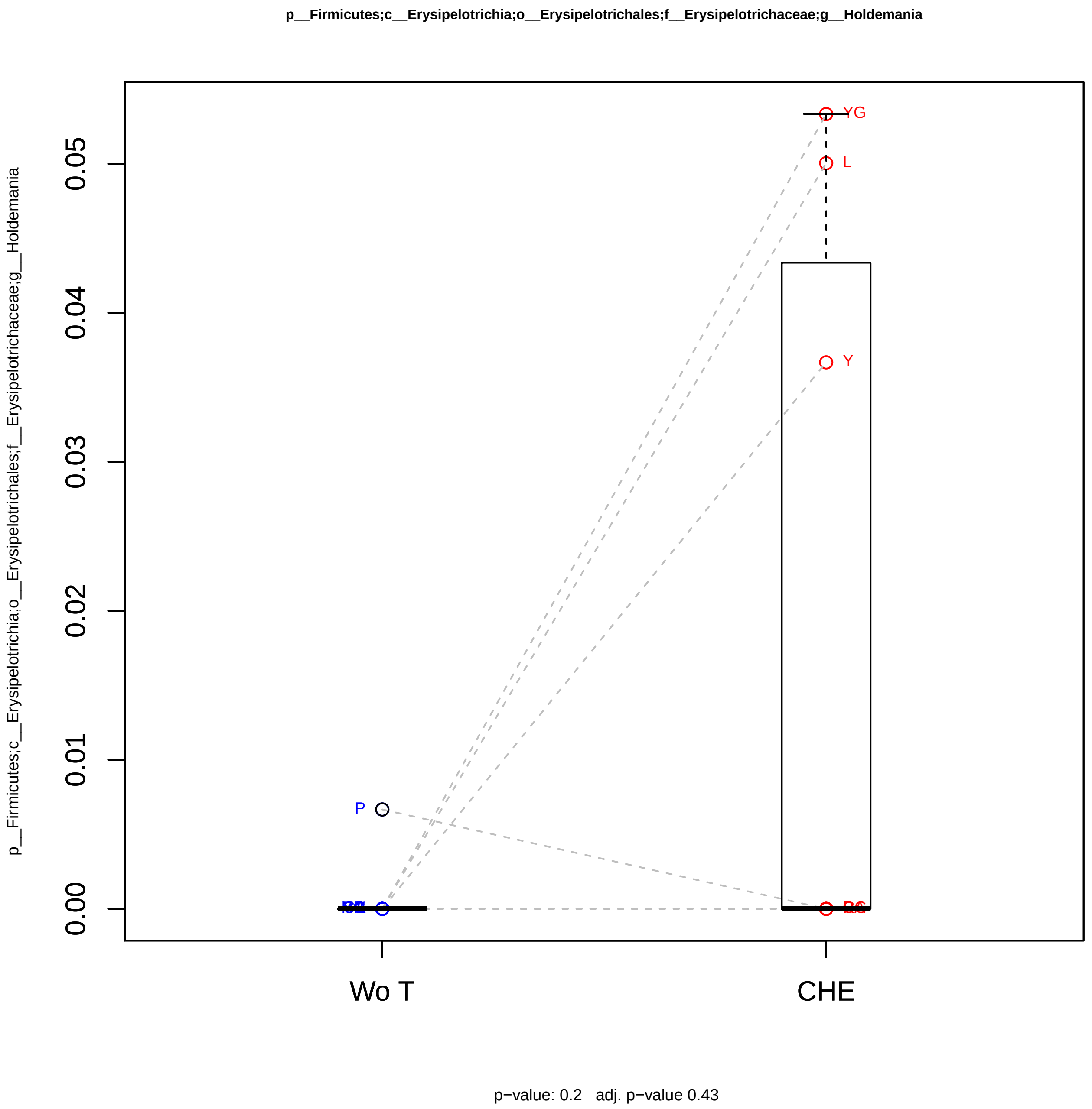
p__Firmicutes;c__Erysipelotrichia;o__Erysipelotrichales;f__Erysipelotrichaceae;g__Holdemania
p__Firmicutes;c__Erysipelotrichia;o__Erysipelotrichales;f__Erysipelotrichaceae;g__Holdemania
0.00
0.01
0.02
0.03
0.04
0.05
YG
L
Y
P
Wo T
CHE
p−value: 0.2   adj. p−value 0.43

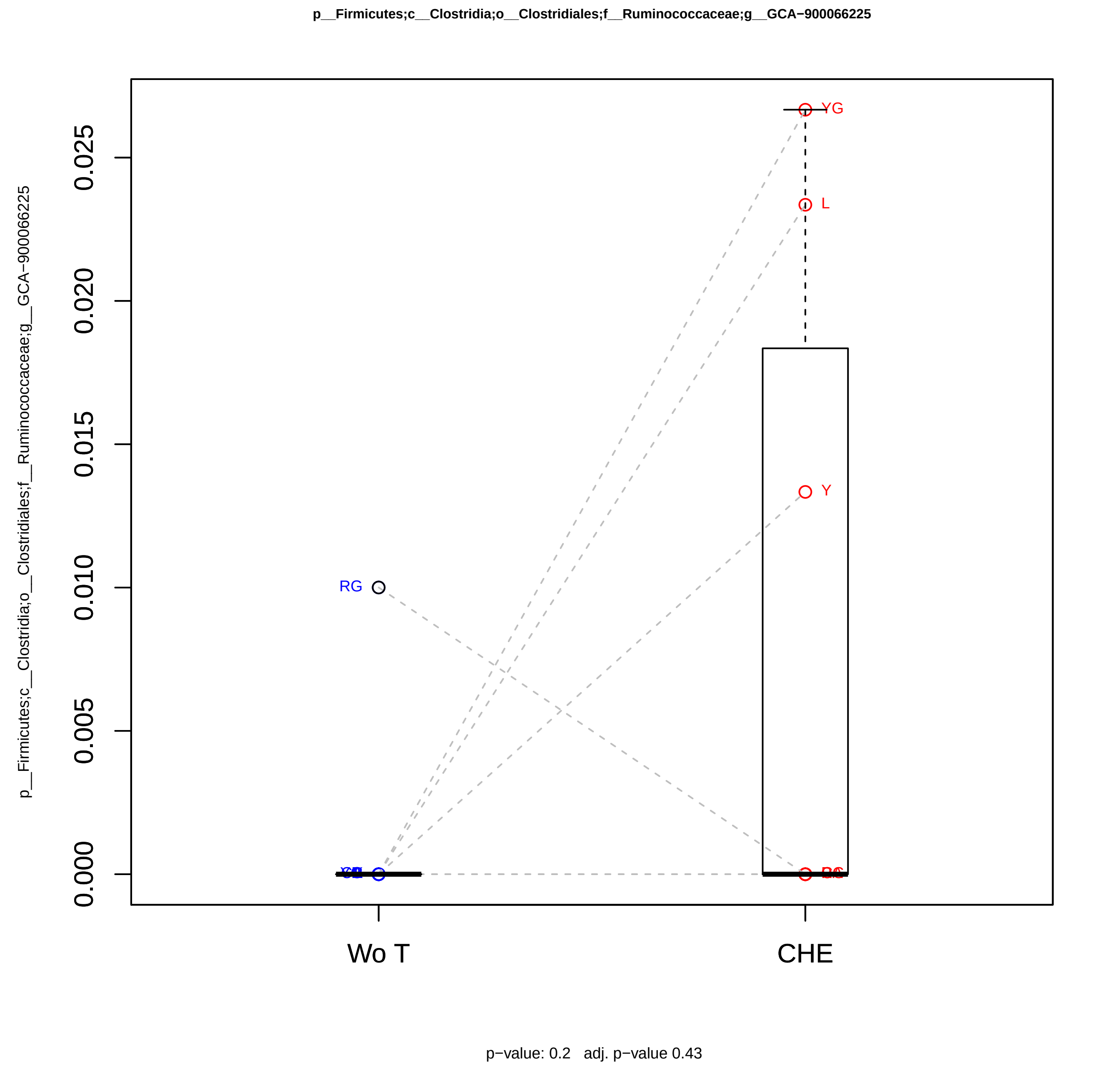

p__Firmicutes;c__Clostridia;o__Clostridiales;f__Ruminococcaceae;g__GCA−900066225
p__Firmicutes;c__Clostridia;o__Clostridiales;f__Ruminococcaceae;g__GCA−900066225
0.000
0.005
0.010
0.015
0.020
0.025
YG
L
Y
RG
Wo T
CHE
p−value: 0.2 adj. p−value 0.43

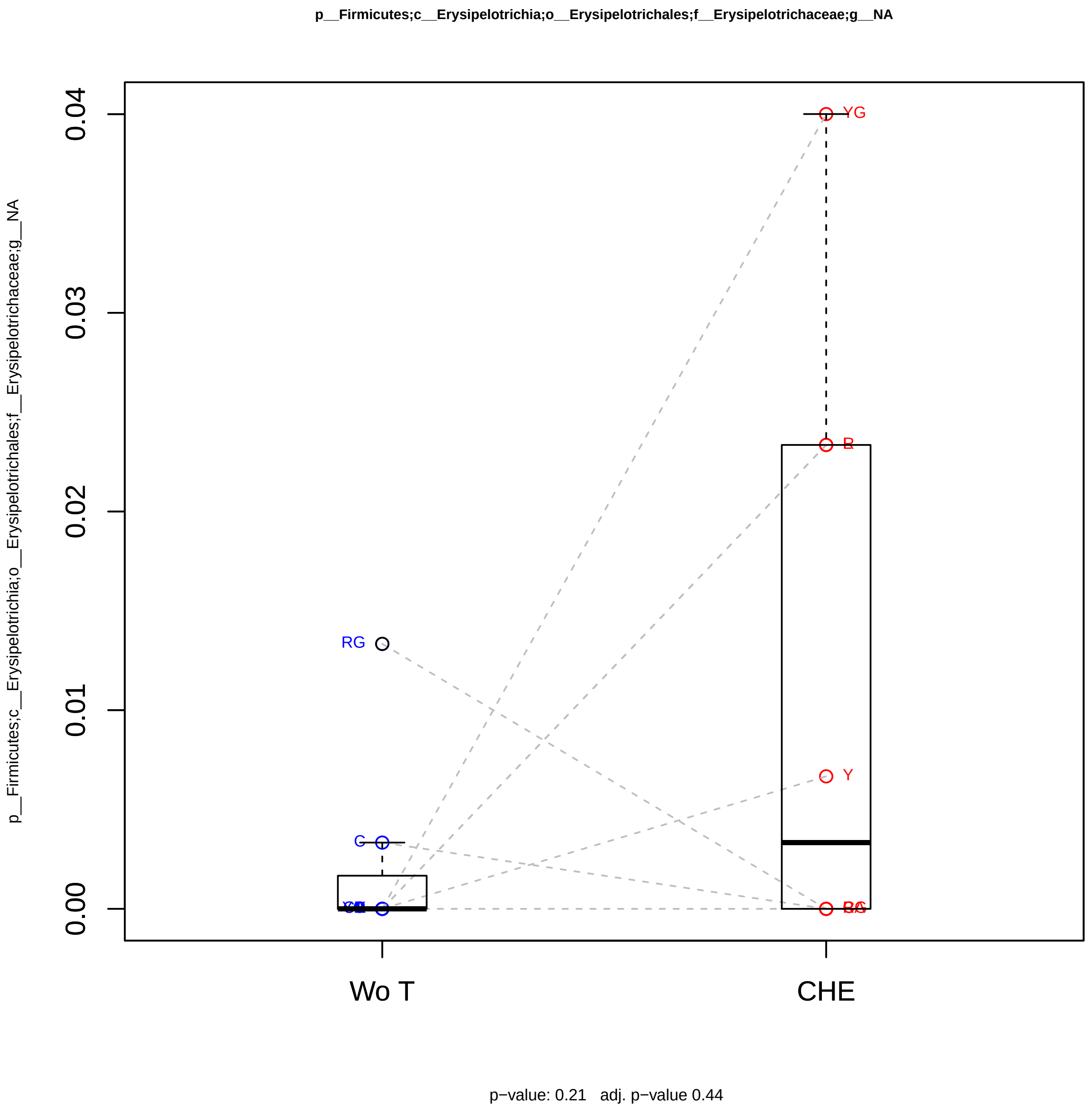
p__Firmicutes;c__Erysipelotrichia;o__Erysipelotrichales;f__Erysipelotrichaceae;g__NA
p__Firmicutes;c__Erysipelotrichia;o__Erysipelotrichales;f__Erysipelotrichaceae;g__NA
0.00
0.01
0.02
0.03
0.04
YG
R
Y
RG
C
Wo T
CHE
p−value: 0.21 adj. p−value 0.44

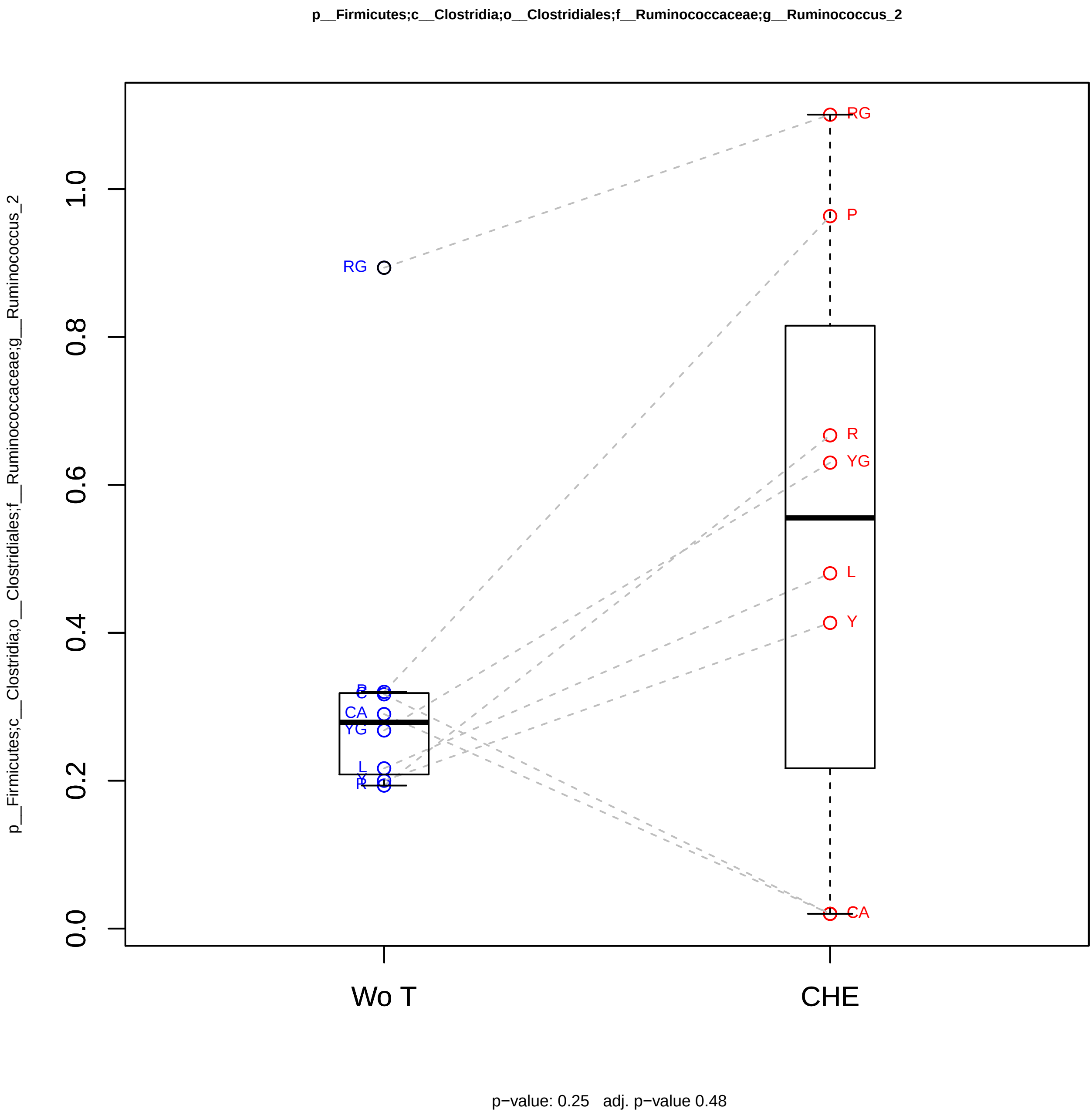
p__Firmicutes;c__Clostridia;o__Clostridiales;f__Ruminococcaceae;g__Ruminococcus_2
p__Firmicutes;c__Clostridia;o__Clostridiales;f__Ruminococcaceae;g__Ruminococcus_2
0.0
0.2
0.4
0.6
0.8
1.0
RG
P
R
YG
L
Y
CA
Wo T
CHE
p−value: 0.25 adj. p−value 0.48

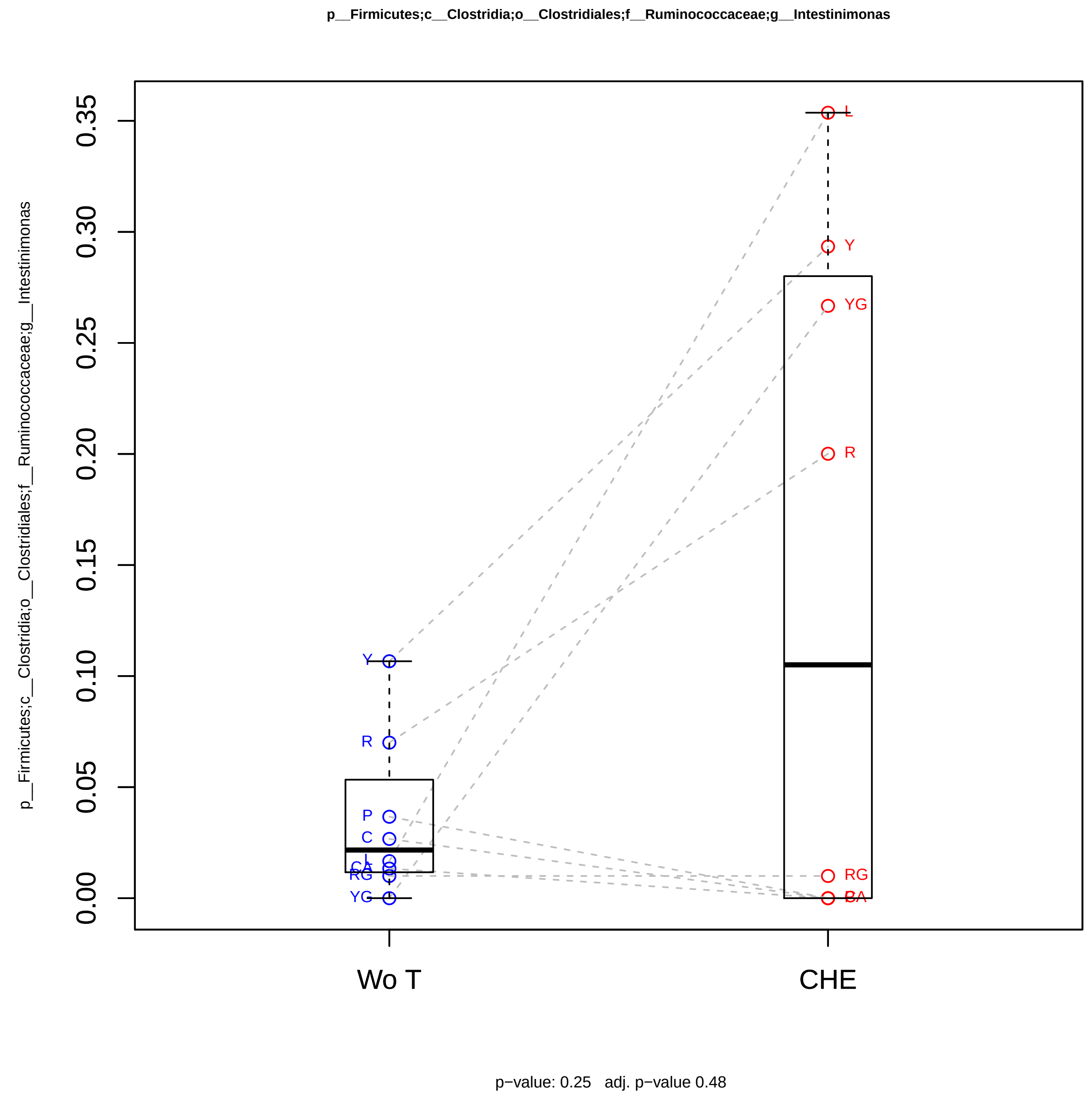
p__Firmicutes;c__Clostridia;o__Clostridiales;f__Ruminococcaceae;g__Intestinimonas
p__Firmicutes;c__Clostridia;o__Clostridiales;f__Ruminococcaceae;g__Intestinimonas
0.00
0.05
0.10
0.15
0.20
0.25
0.30
0.35
Y
R
P
C
L
CA
RG
YG
L
Y
YG
R
RG
P
CA
Wo T
CHE
p−value: 0.25 adj. p−value 0.48

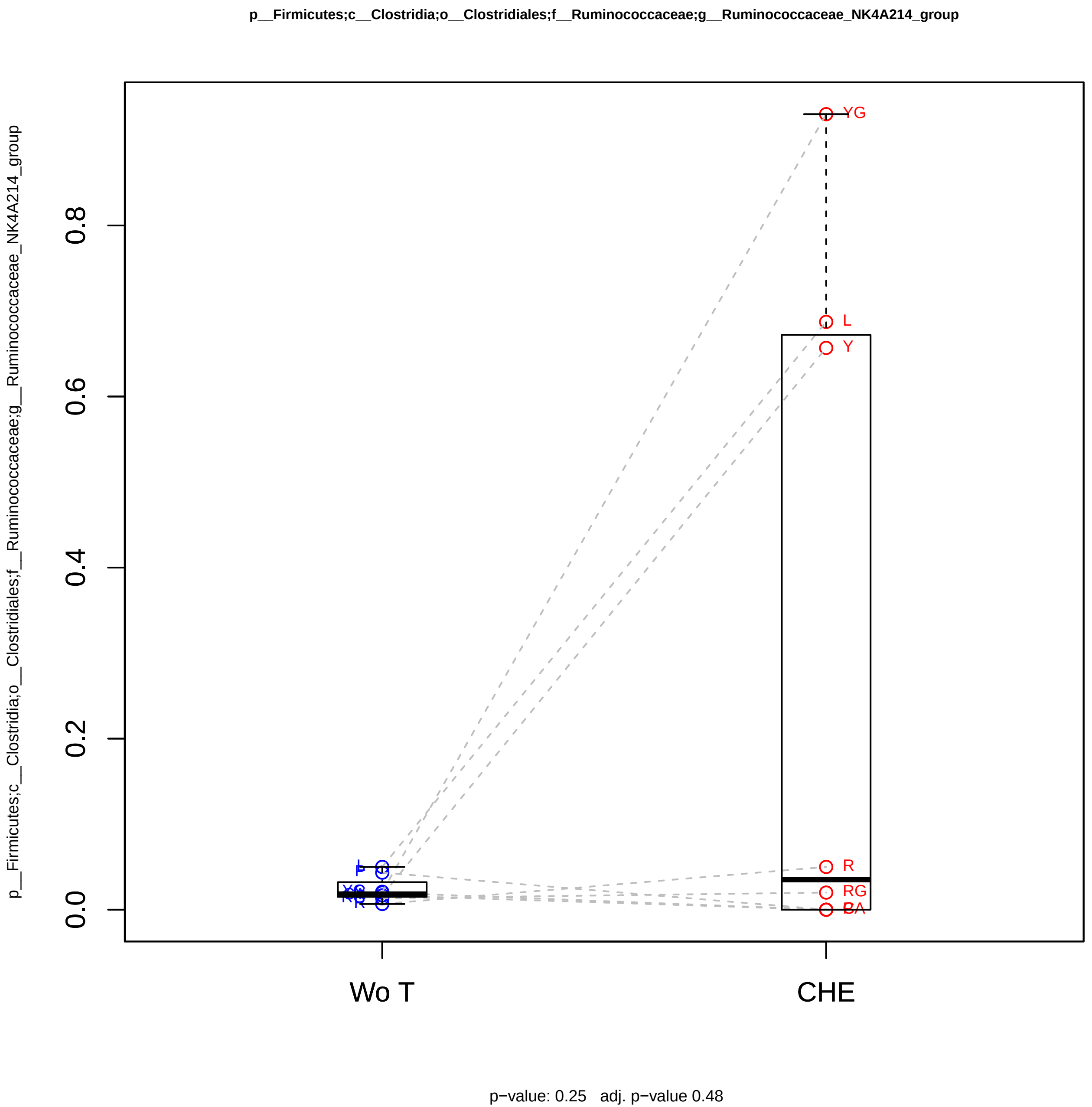
p__Firmicutes;c__Clostridia;o__Clostridiales;f__Ruminococcaceae;g__Ruminococcaceae_NK4A214_group
p__Firmicutes;c__Clostridia;o__Clostridiales;f__Ruminococcaceae;g__Ruminococcaceae_NK4A214_group
0.0
0.2
0.4
0.6
0.8
Wo T
CHE
YG
L
Y
R
RG
p−value: 0.25 adj. p−value 0.48

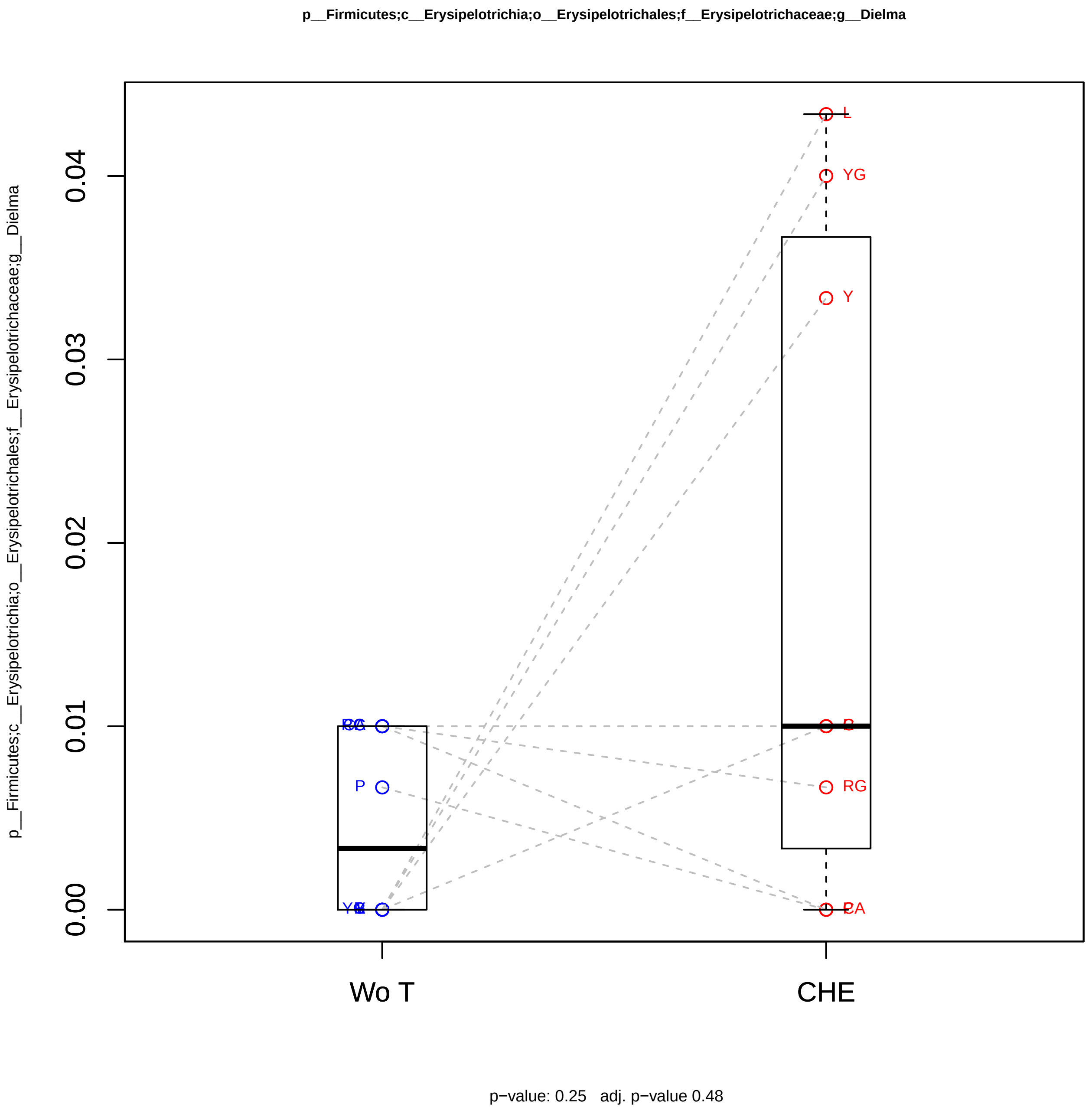

p__Firmicutes;c__Erysipelotrichia;o__Erysipelotrichales;f__Erysipelotrichaceae;g__Dielma
p__Firmicutes;c__Erysipelotrichia;o__Erysipelotrichales;f__Erysipelotrichaceae;g__Dielma
0.00
0.01
0.02
0.03
0.04
Wo T
CHE
L
YG
Y
RG
P
p−value: 0.25 adj. p−value 0.48

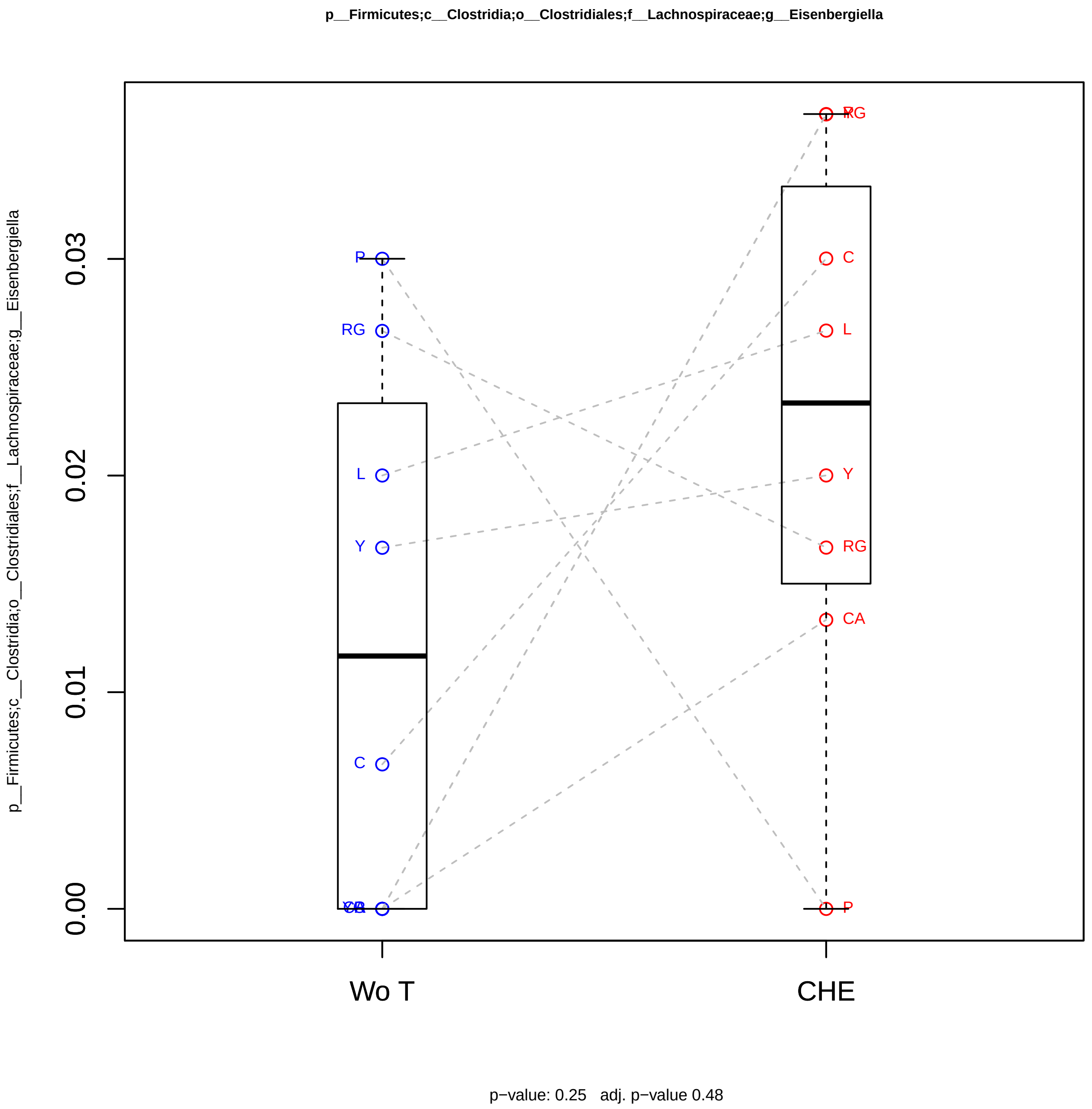
p__Firmicutes;c__Clostridia;o__Clostridiales;f__Lachnospiraceae;g__Eisenbergiella
p__Firmicutes;c__Clostridia;o__Clostridiales;f__Lachnospiraceae;g__Eisenbergiella
0.00
0.01
0.02
0.03
Wo T
CHE
P
RG
L
Y
C
RG
C
L
Y
RG
CA
P
p−value: 0.25 adj. p−value 0.48

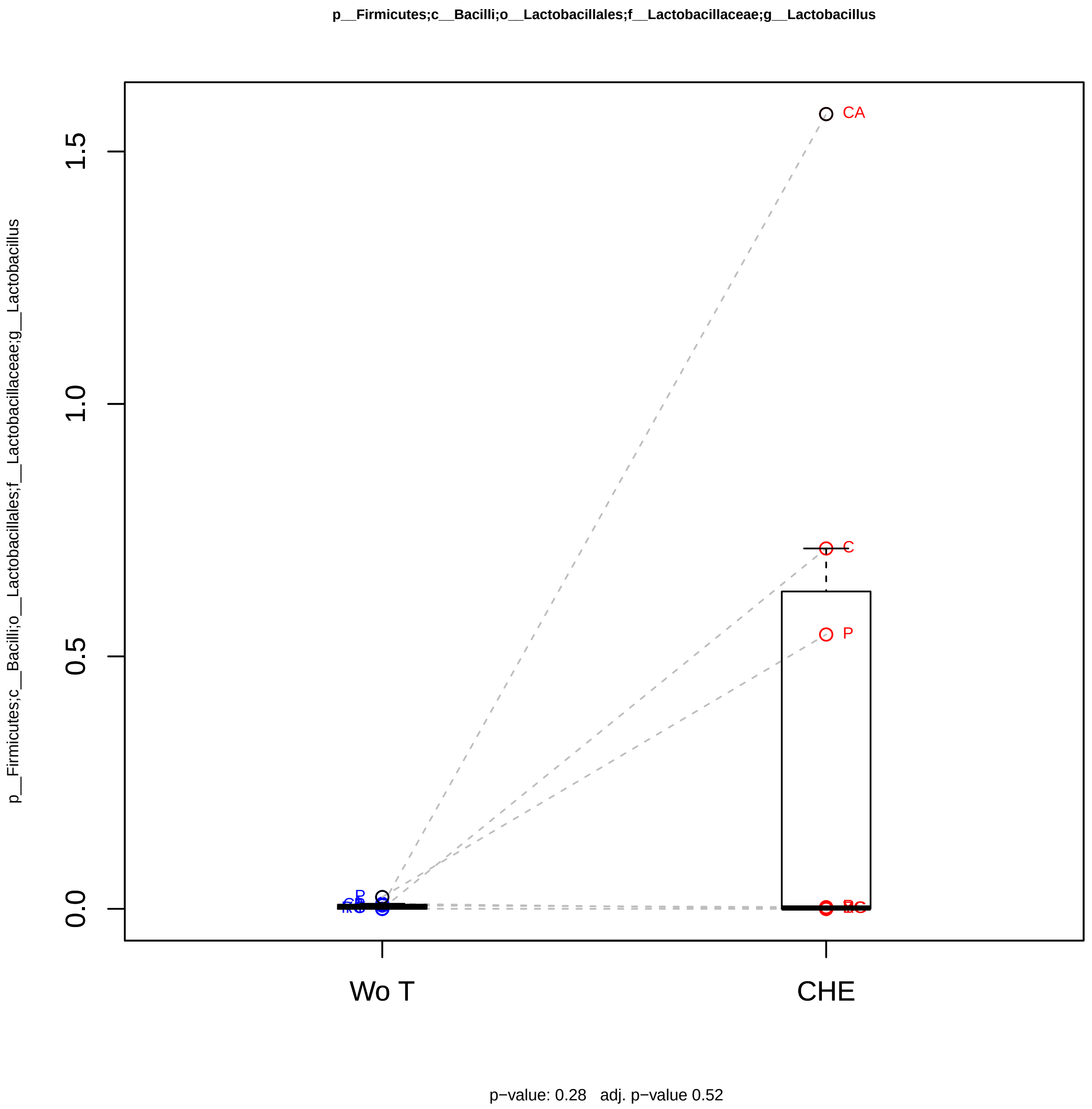

p__Firmicutes;c__Bacilli;o__Lactobacillales;f__Lactobacillaceae;g__Lactobacillus
p__Firmicutes;c__Bacilli;o__Lactobacillales;f__Lactobacillaceae;g__Lactobacillus
0.0
0.5
1.0
1.5
CA
C
P
Wo T
CHE
p−value: 0.28   adj. p−value 0.52

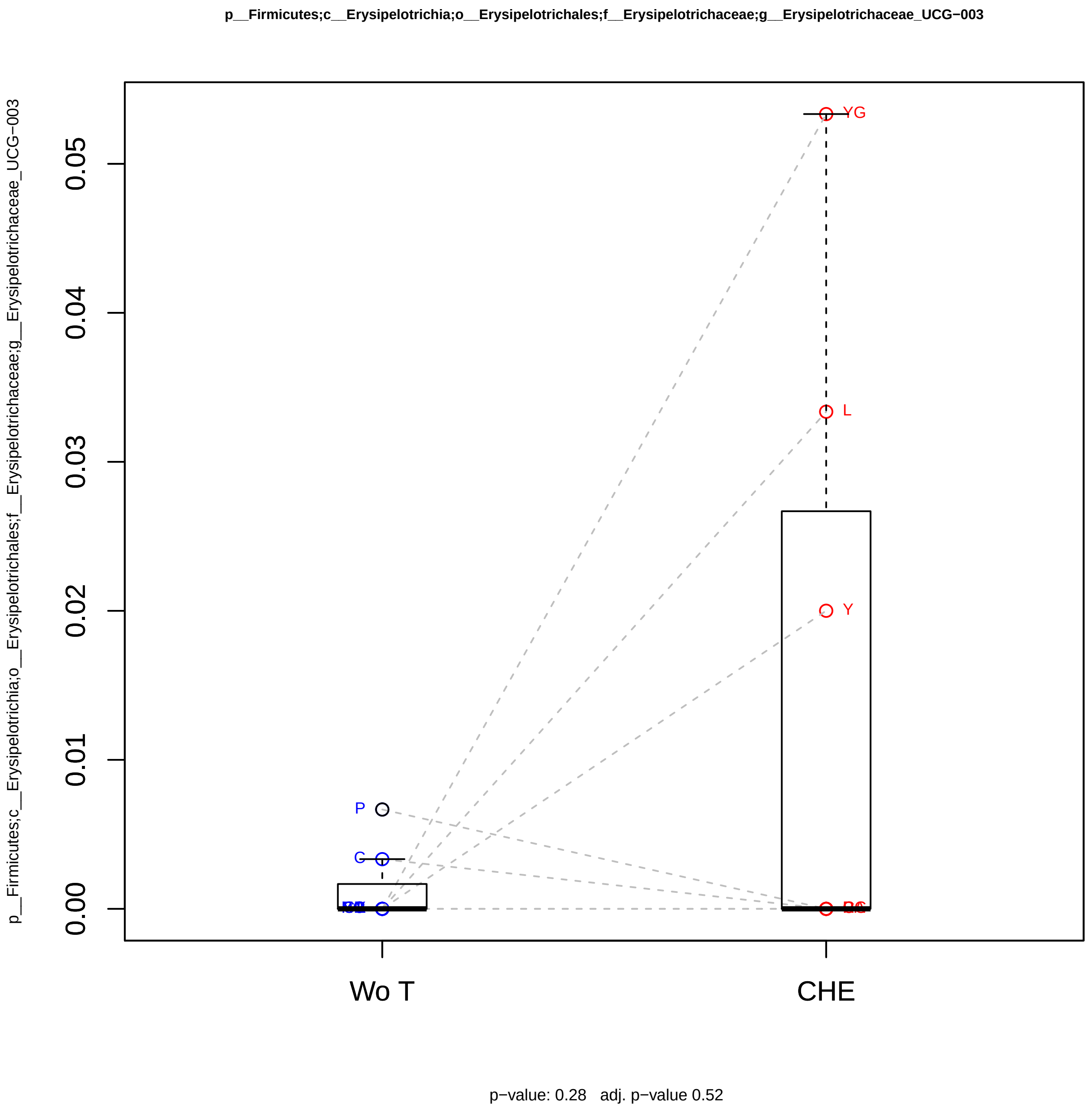
p__Firmicutes;c__Erysipelotrichia;o__Erysipelotrichales;f__Erysipelotrichaceae;g__Erysipelotrichaceae_UCG−003
p__Firmicutes;c__Erysipelotrichia;o__Erysipelotrichales;f__Erysipelotrichaceae;g__Erysipelotrichaceae_UCG−003
0.00
0.01
0.02
0.03
0.04
0.05
YG
L
Y
P
C
Wo T
CHE
p−value: 0.28 adj. p−value 0.52

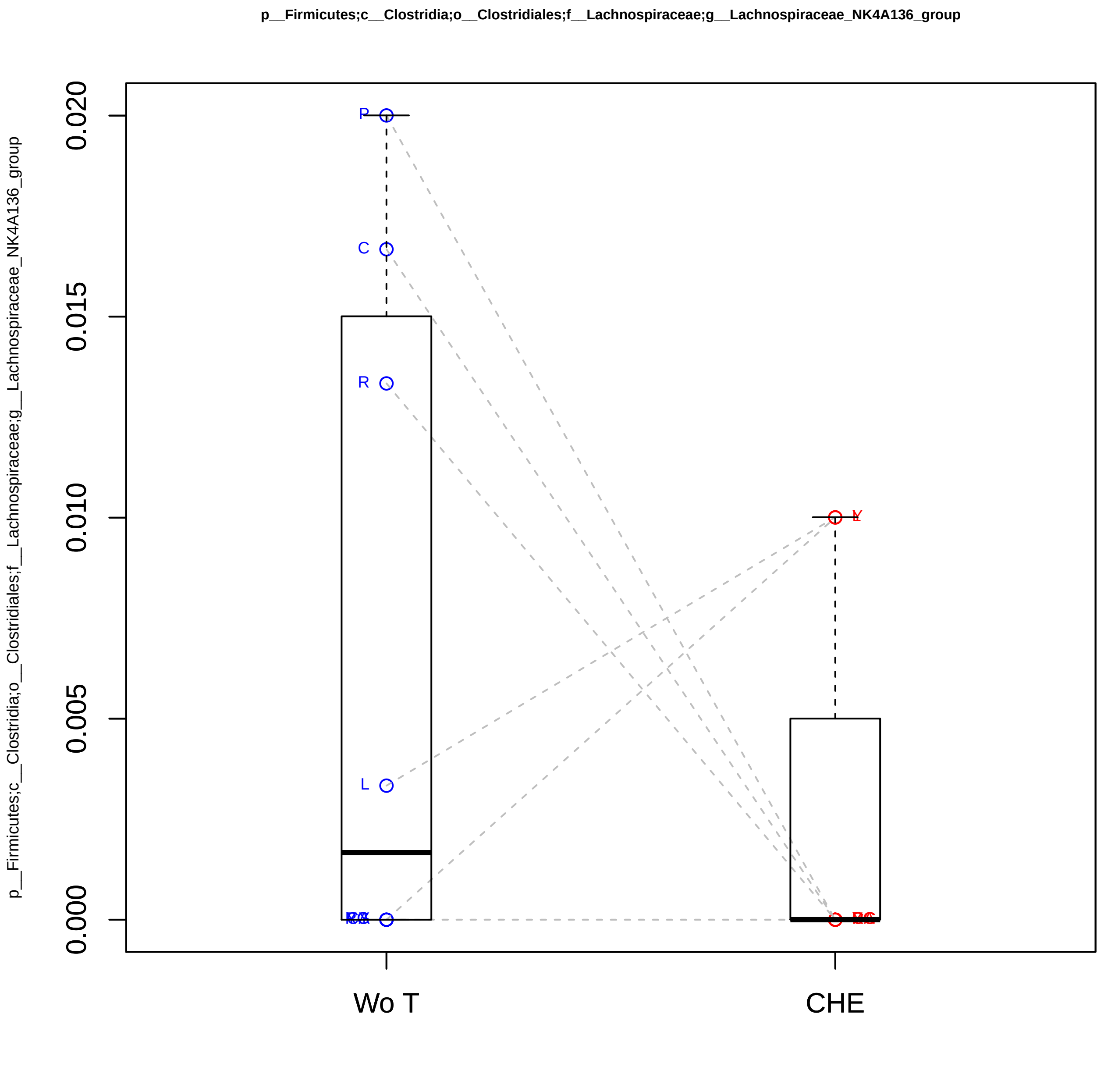
p__Firmicutes;c__Clostridia;o__Clostridiales;f__Lachnospiraceae;g__Lachnospiraceae_NK4A136_group
p__Firmicutes;c__Clostridia;o__Clostridiales;f__Lachnospiraceae;g__Lachnospiraceae_NK4A136_group
0.000
0.005
0.010
0.015
0.020
P
C
R
L
Wo T
CHE
p−value: 0.28 adj. p−value 0.52

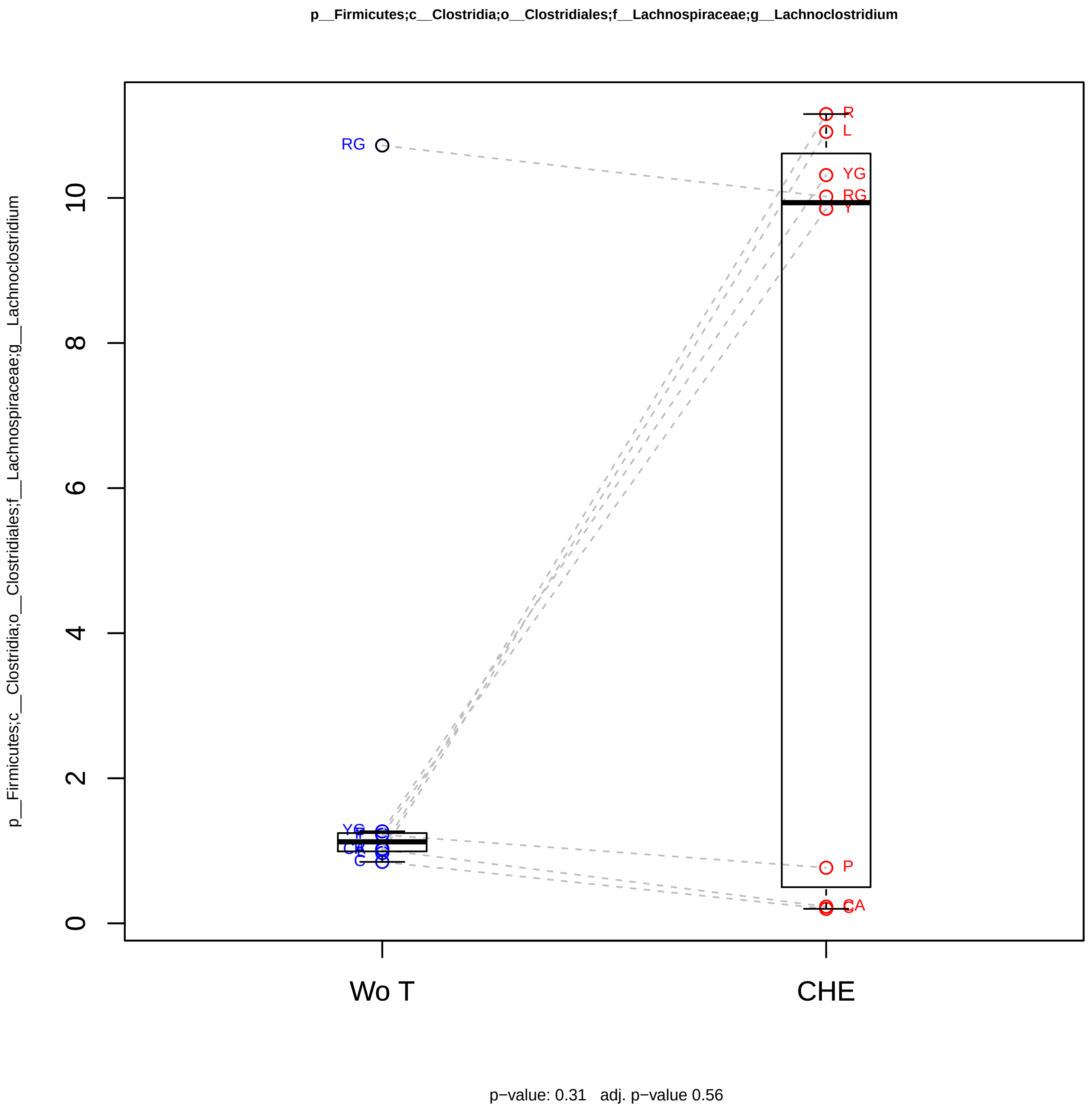
p__Firmicutes;c__Clostridia;o__Clostridiales;f__Lachnospiraceae;g__Lachnoclostridium
p__Firmicutes;c__Clostridia;o__Clostridiales;f__Lachnospiraceae;g__Lachnoclostridium
0
2
4
6
8
10
RG
R
L
YG
RG
Y
P
CA
Wo T
CHE
p−value: 0.31   adj. p−value 0.56

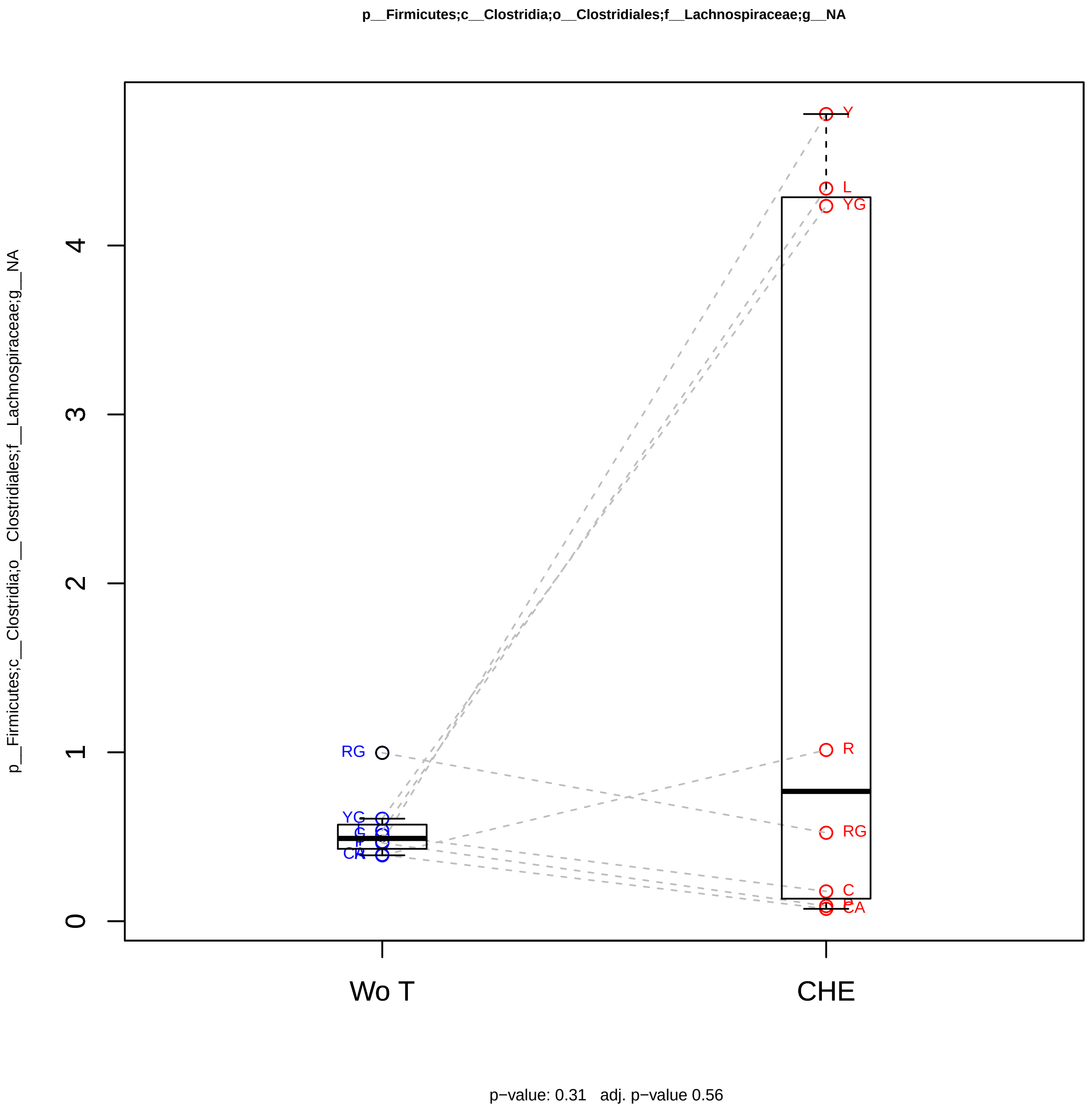

p__Firmicutes;c__Clostridia;o__Clostridiales;f__Lachnospiraceae;g__NA
p__Firmicutes;c__Clostridia;o__Clostridiales;f__Lachnospiraceae;g__NA
0
1
2
3
4
Wo T
CHE
RG
YG
Y
L
YG
R
RG
C
CA
p−value: 0.31 adj. p−value 0.56

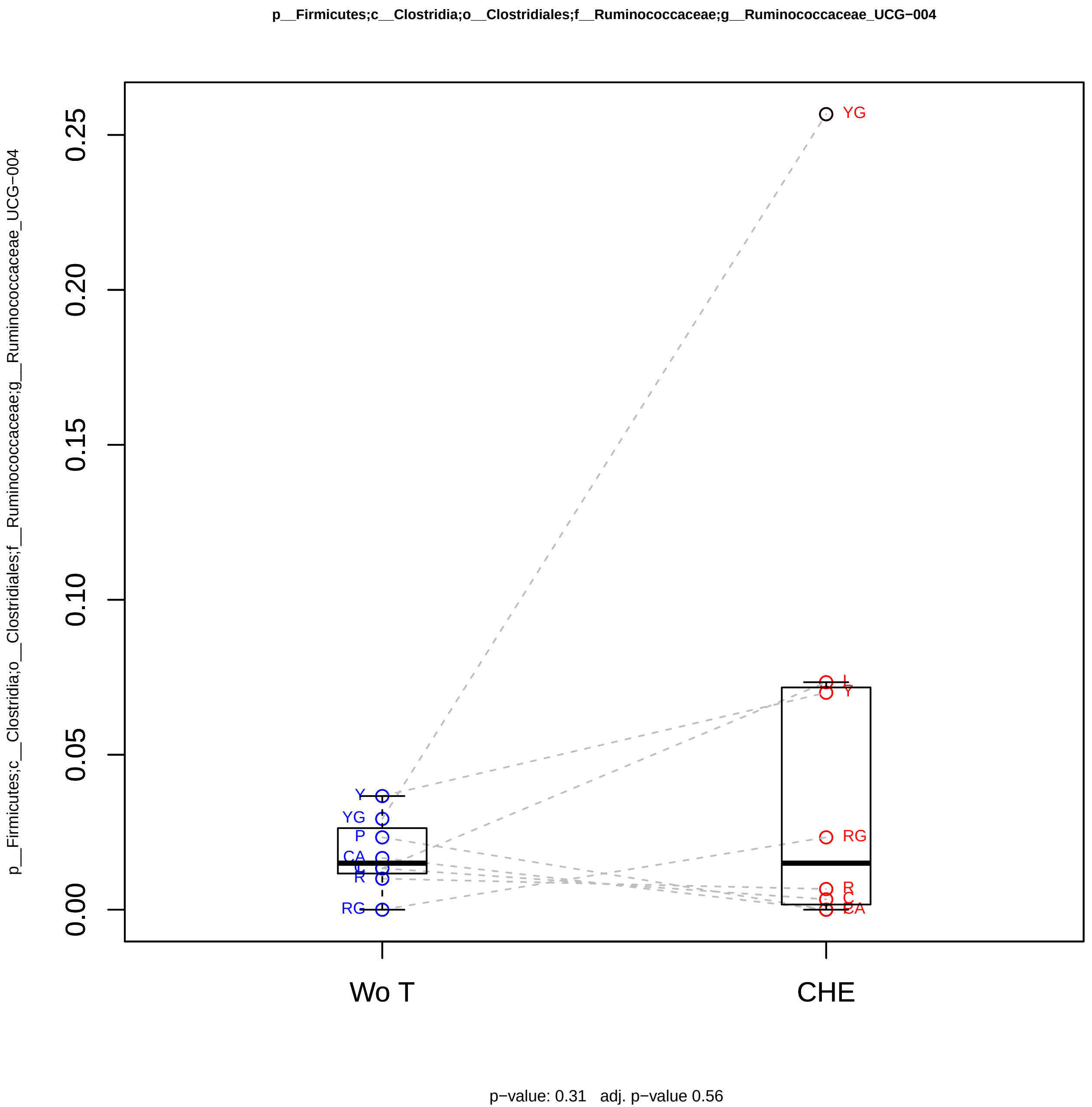
p__Firmicutes;c__Clostridia;o__Clostridiales;f__Ruminococcaceae;g__Ruminococcaceae_UCG−004
p__Firmicutes;c__Clostridia;o__Clostridiales;f__Ruminococcaceae;g__Ruminococcaceae_UCG−004
0.00
0.05
0.10
0.15
0.20
0.25
Y
YG
P
CA
L
R
RG
YG
L
Y
RG
R
C
CA
Wo T
CHE
p−value: 0.31 adj. p−value 0.56

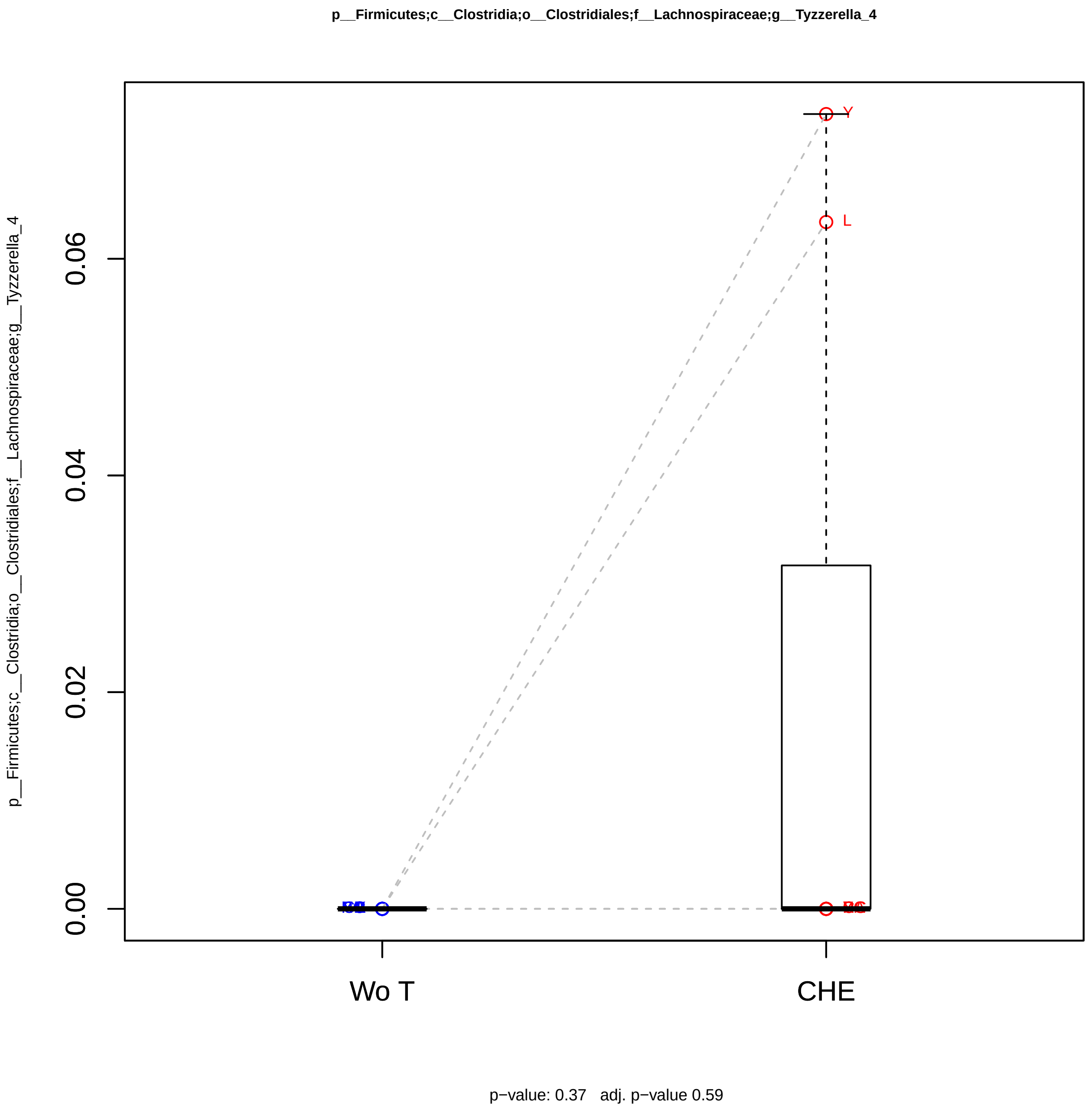

p__Firmicutes;c__Clostridia;o__Clostridiales;f__Lachnospiraceae;g__Tyzzerella_4
p__Firmicutes;c__Clostridia;o__Clostridiales;f__Lachnospiraceae;g__Tyzzerella_4
0.00
0.02
0.04
0.06
Y
L
Wo T
CHE
p−value: 0.37 adj. p−value 0.59

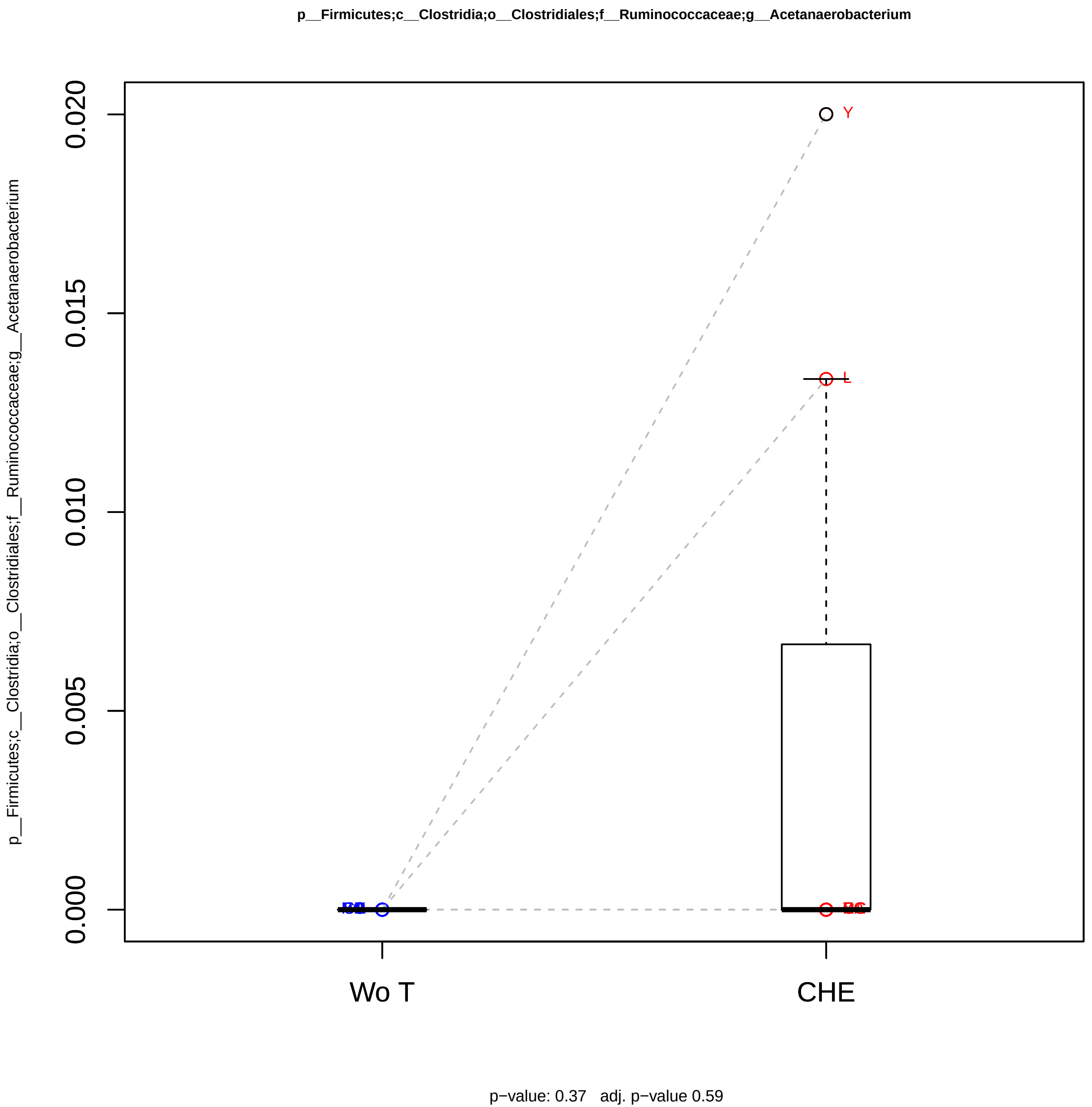
p__Firmicutes;c__Clostridia;o__Clostridiales;f__Ruminococcaceae;g__Acetanaerobacterium
p__Firmicutes;c__Clostridia;o__Clostridiales;f__Ruminococcaceae;g__Acetanaerobacterium
0.000
0.005
0.010
0.015
0.020
Y
L
Wo T
CHE
p−value: 0.37   adj. p−value 0.59

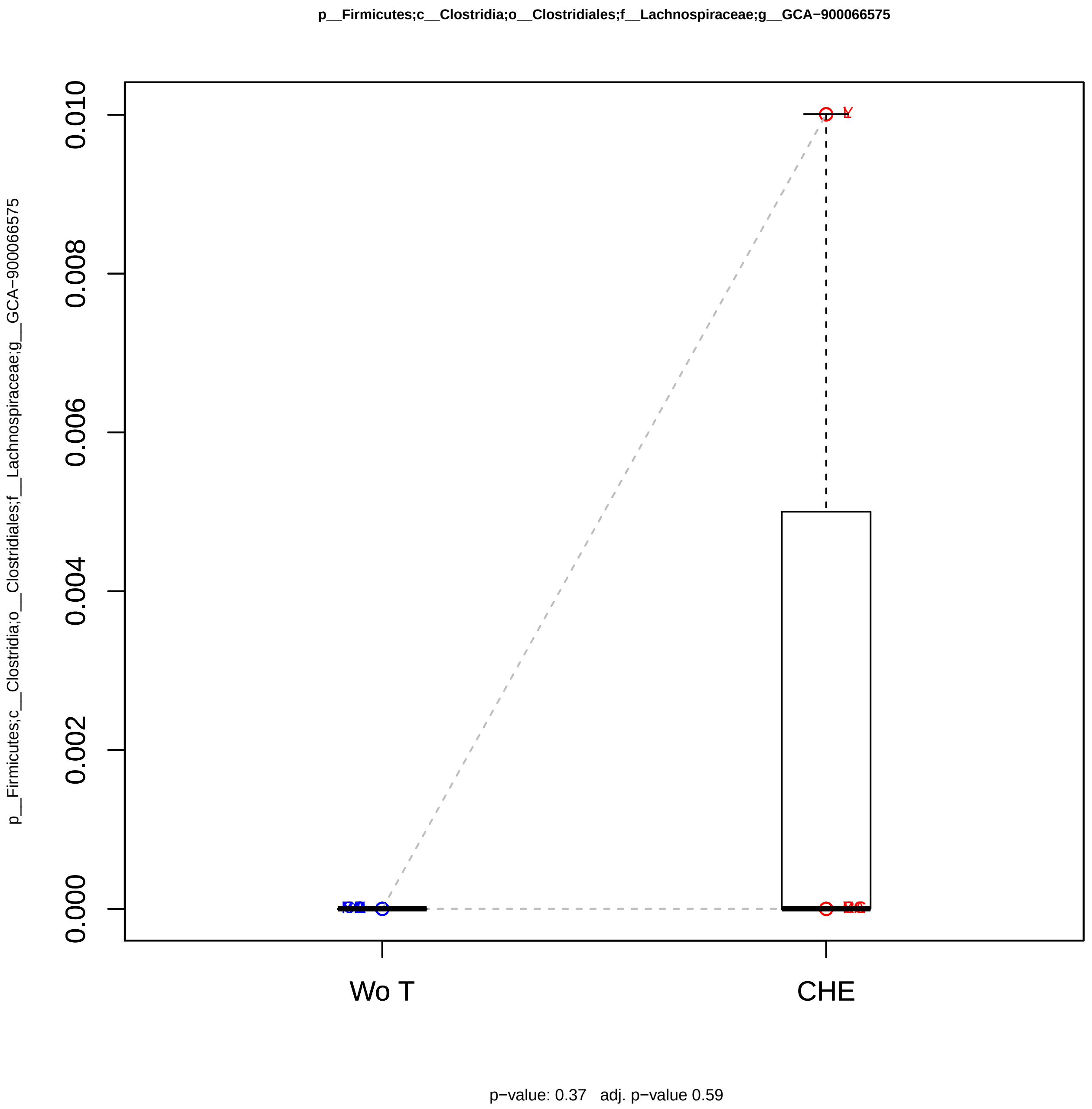
p__Firmicutes;c__Clostridia;o__Clostridiales;f__Lachnospiraceae;g__GCA−900066575
p__Firmicutes;c__Clostridia;o__Clostridiales;f__Lachnospiraceae;g__GCA−900066575
0.000
0.002
0.004
0.006
0.008
0.010
Wo T
CHE
p−value: 0.37   adj. p−value 0.59

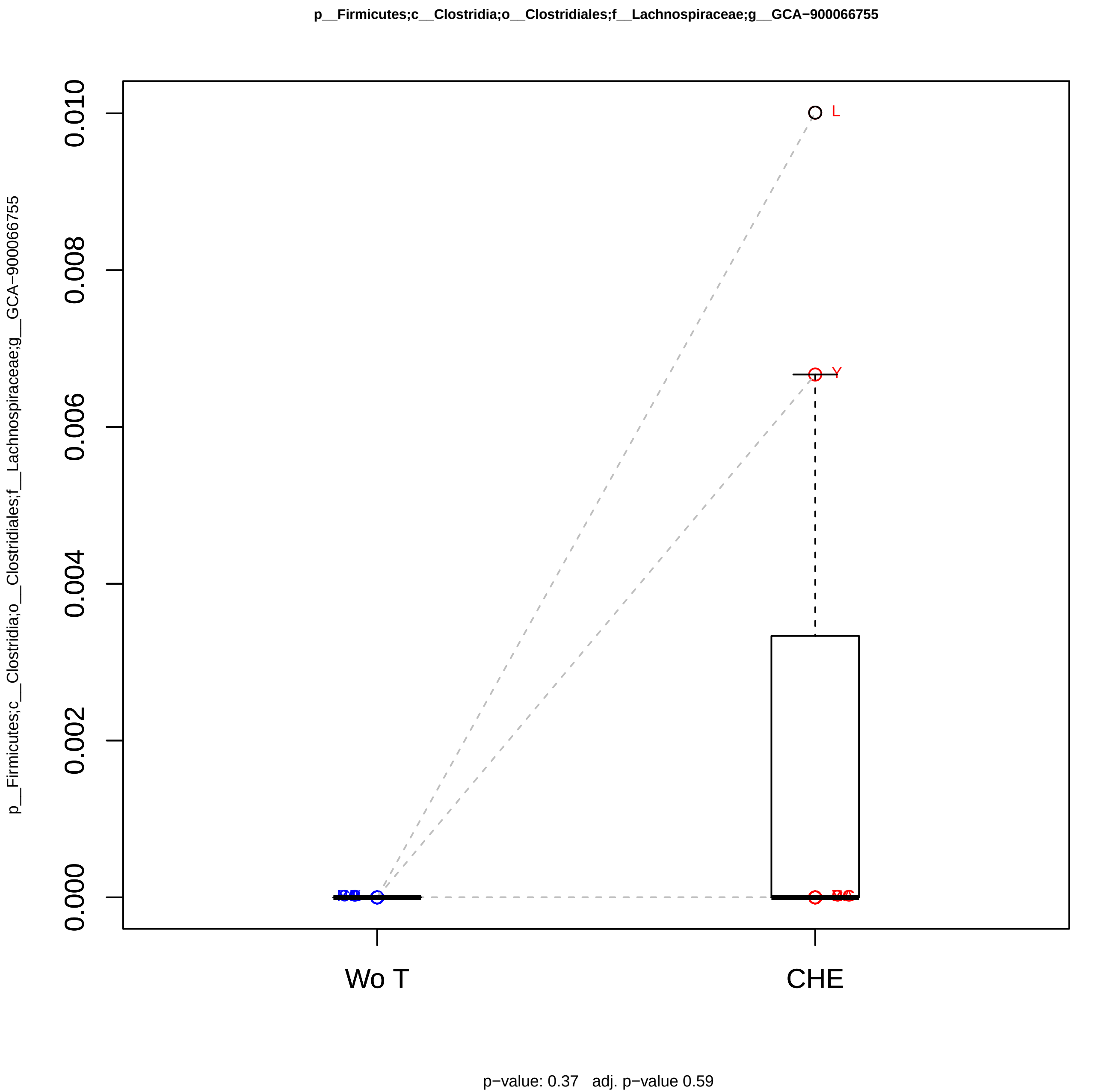
p__Firmicutes;c__Clostridia;o__Clostridiales;f__Lachnospiraceae;g__GCA−900066755
p__Firmicutes;c__Clostridia;o__Clostridiales;f__Lachnospiraceae;g__GCA−900066755
0.000
0.002
0.004
0.006
0.008
0.010
L
Y
Wo T
CHE
p−value: 0.37   adj. p−value 0.59

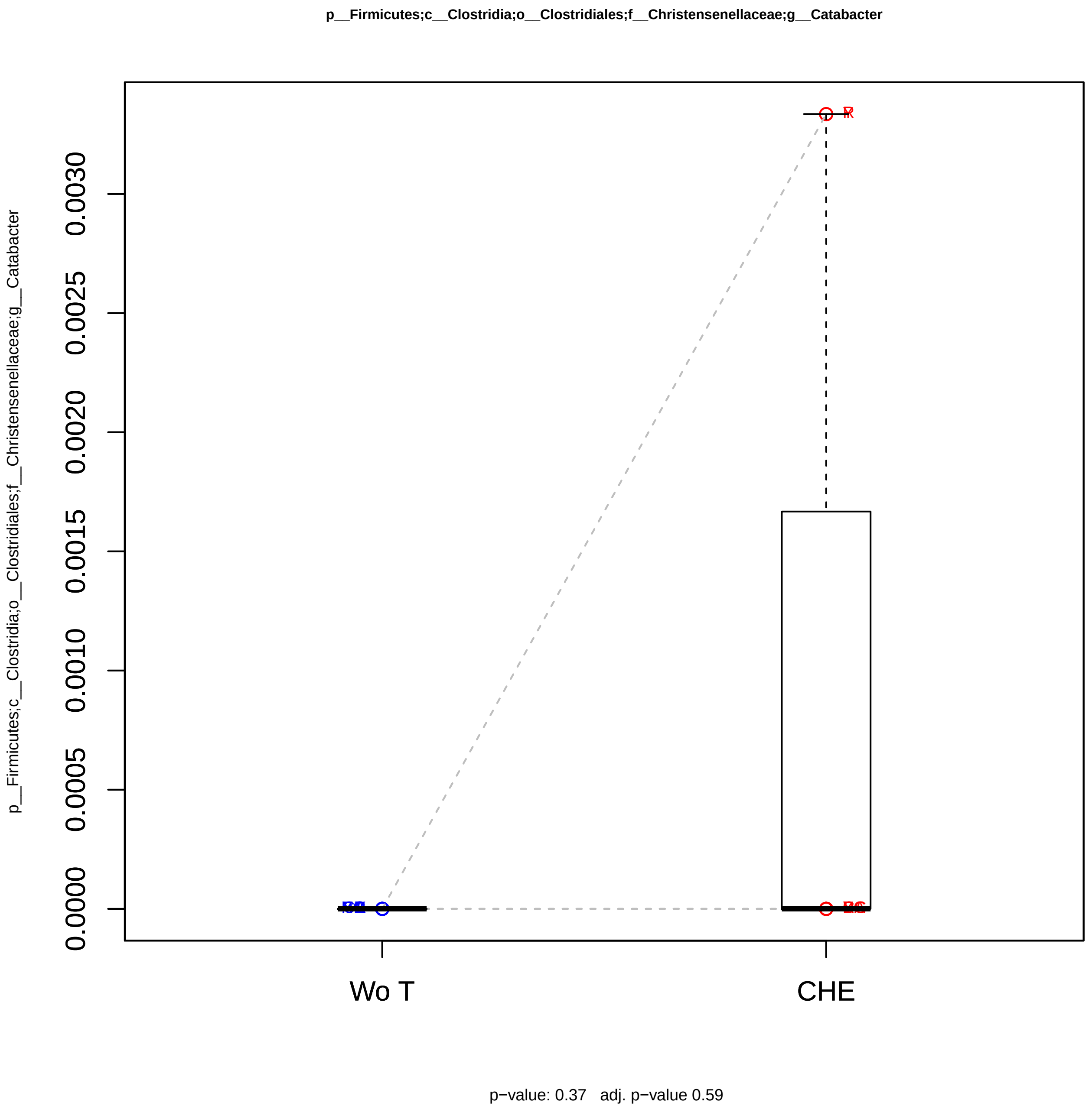

p__Firmicutes;c__Clostridia;o__Clostridiales;f__Christensenellaceae;g__Catabacter
p__Firmicutes;c__Clostridia;o__Clostridiales;f__Christensenellaceae;g__Catabacter
0.0000
0.0005
0.0010
0.0015
0.0020
0.0025
0.0030
Wo T
CHE
p−value: 0.37 adj. p−value 0.59

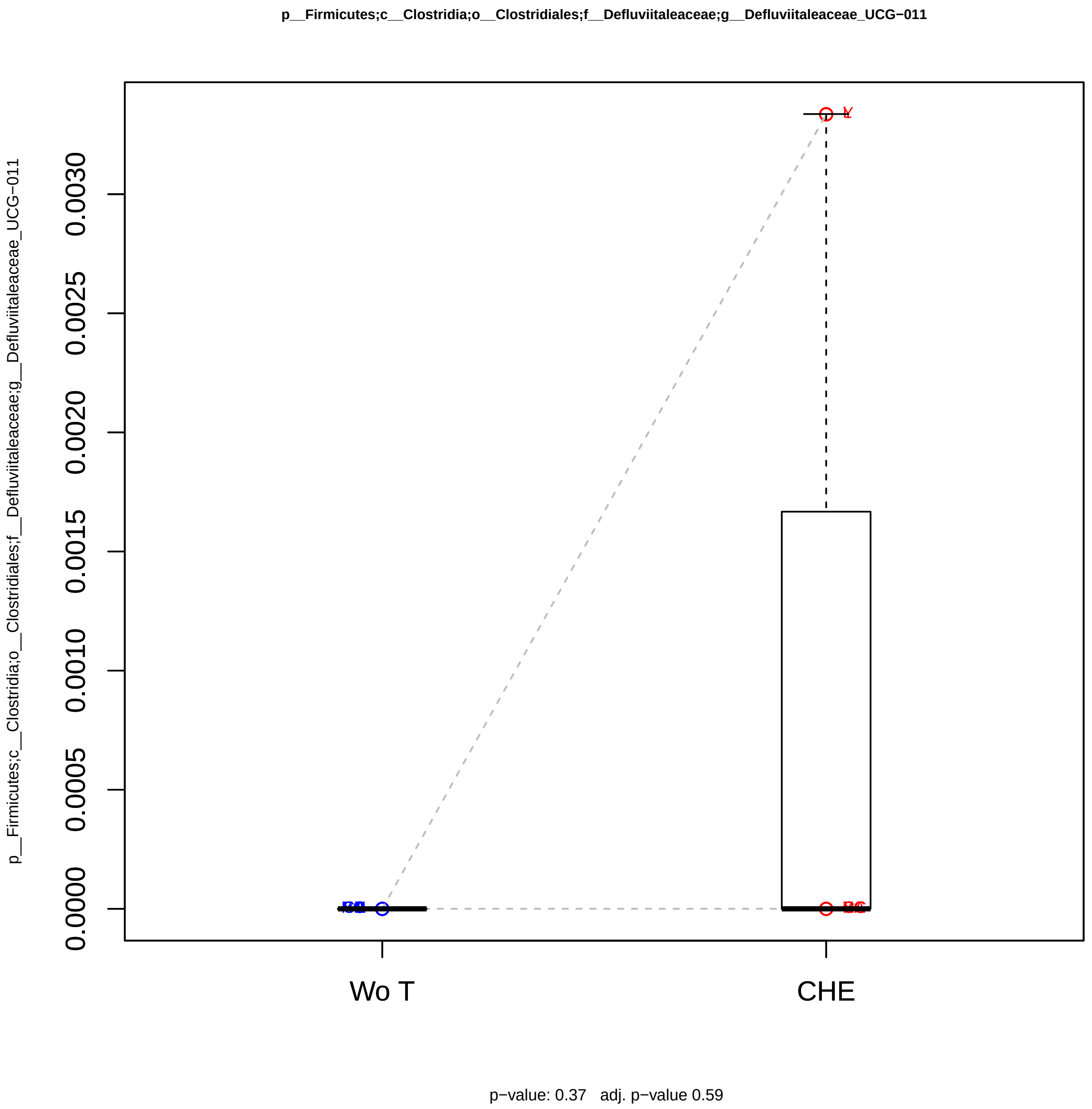
p__Firmicutes;c__Clostridia;o__Clostridiales;f__Defluviitaleaceae;g__Defluviitaleaceae_UCG−011
p__Firmicutes;c__Clostridia;o__Clostridiales;f__Defluviitaleaceae;g__Defluviitaleaceae_UCG−011
0.0000
0.0005
0.0010
0.0015
0.0020
0.0025
0.0030
Wo T
CHE
p−value: 0.37 adj. p−value 0.59

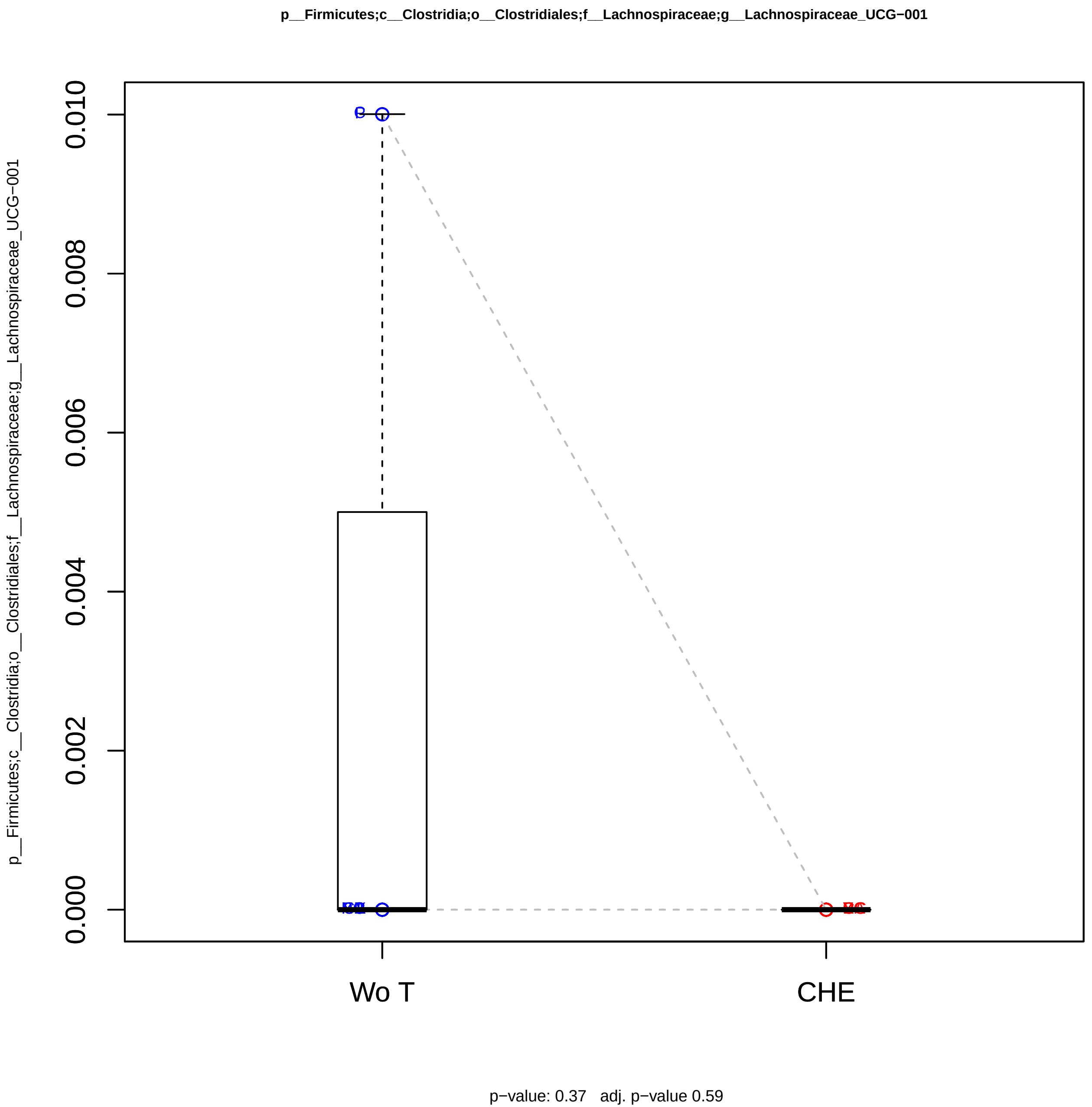
p__Firmicutes;c__Clostridia;o__Clostridiales;f__Lachnospiraceae;g__Lachnospiraceae_UCG−001
p__Firmicutes;c__Clostridia;o__Clostridiales;f__Lachnospiraceae;g__Lachnospiraceae_UCG−001
0.000
0.002
0.004
0.006
0.008
0.010
Wo T
CHE
p−value: 0.37 adj. p−value 0.59

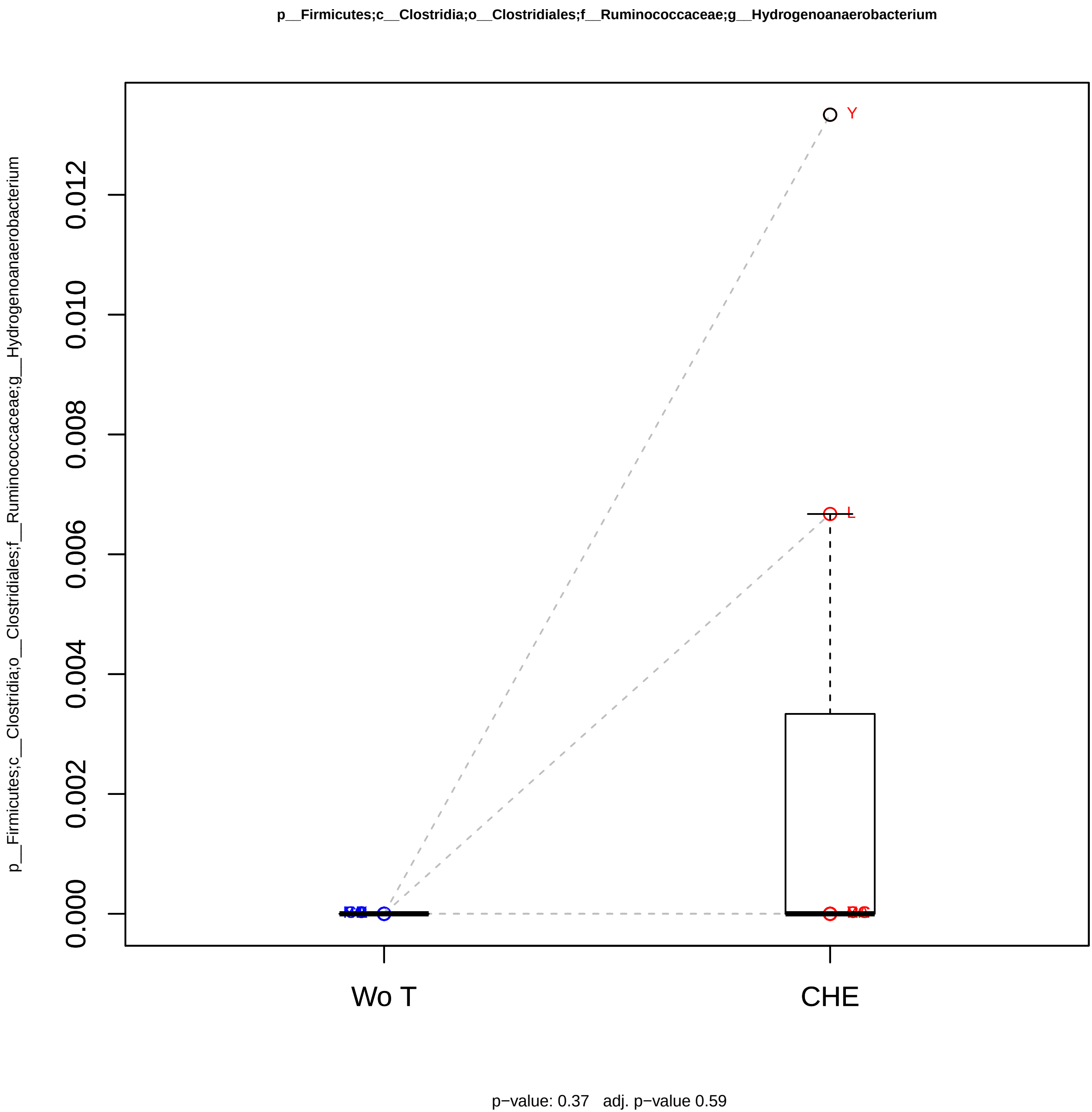
p__Firmicutes;c__Clostridia;o__Clostridiales;f__Ruminococcaceae;g__Hydrogenoanaerobacterium
p__Firmicutes;c__Clostridia;o__Clostridiales;f__Ruminococcaceae;g__Hydrogenoanaerobacterium
0.000
0.002
0.004
0.006
0.008
0.010
0.012
Y
L
Wo T
CHE
p−value: 0.37 adj. p−value 0.59

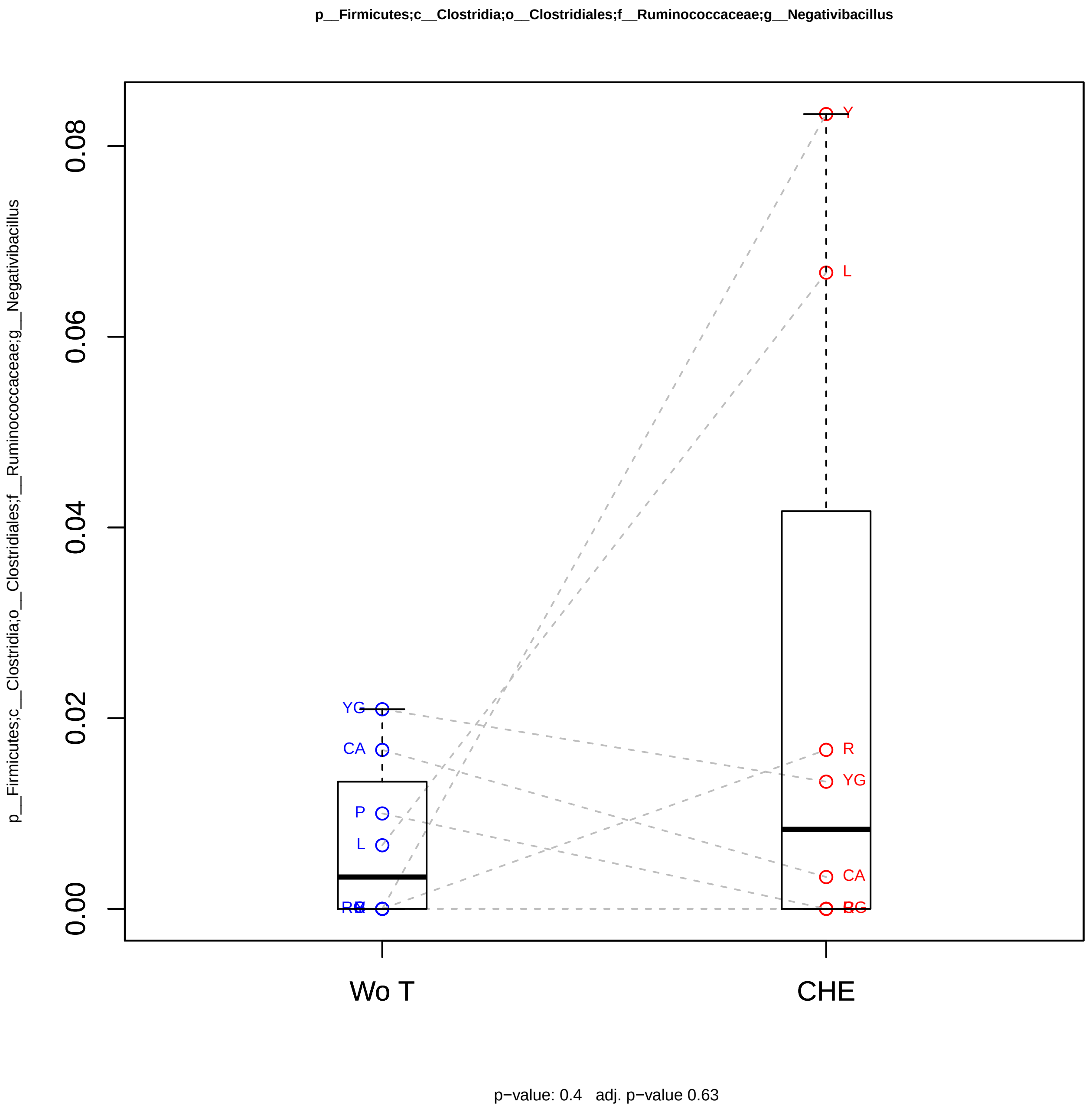
p__Firmicutes;c__Clostridia;o__Clostridiales;f__Ruminococcaceae;g__Negativibacillus
p__Firmicutes;c__Clostridia;o__Clostridiales;f__Ruminococcaceae;g__Negativibacillus
0.00
0.02
0.04
0.06
0.08
Wo T
CHE
YG
CA
P
L
Y
R
p−value: 0.4 adj. p−value 0.63

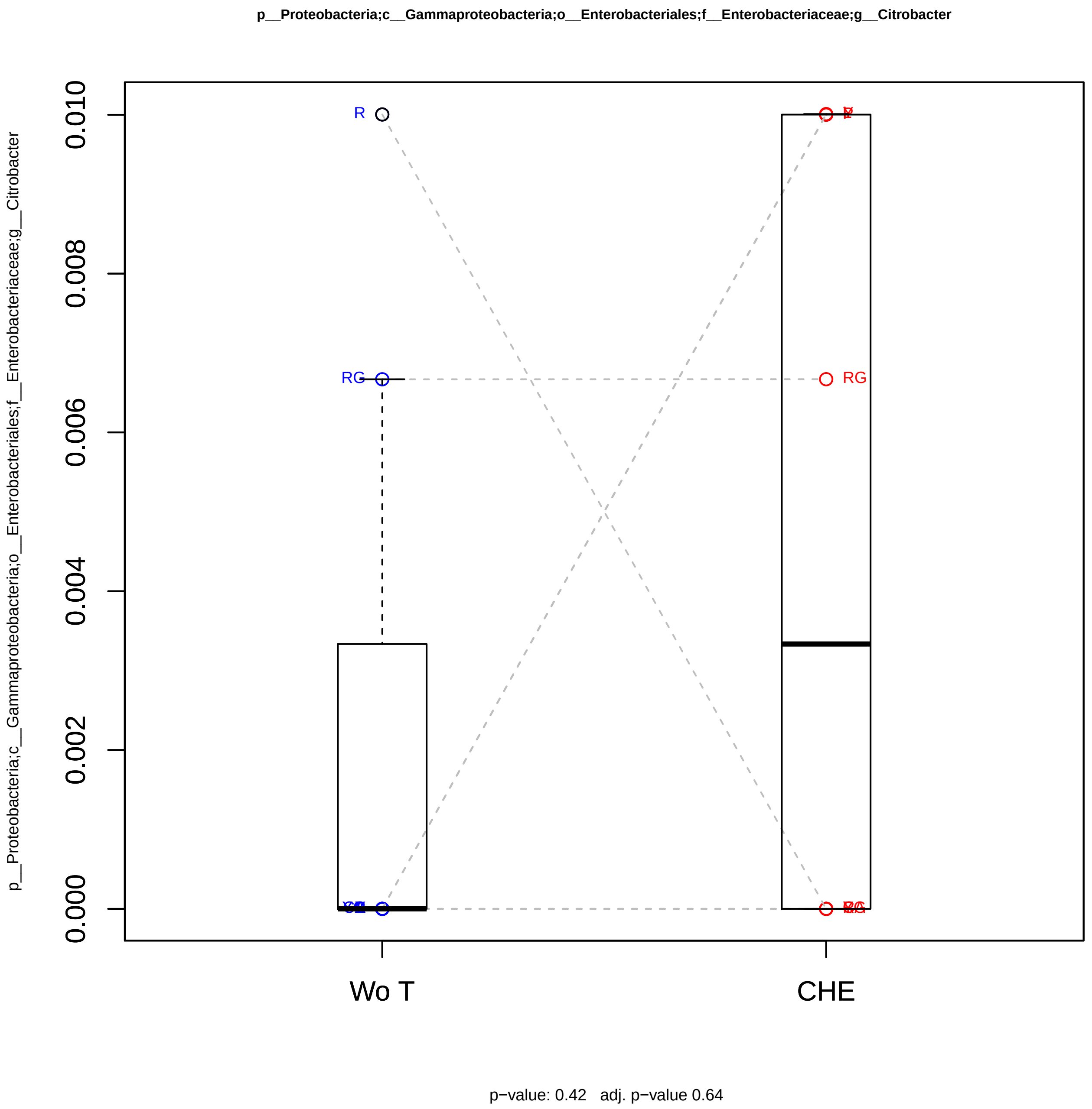
p__Proteobacteria;c__Gammaproteobacteria;o__Enterobacteriales;f__Enterobacteriaceae;g__Citrobacter
p__Proteobacteria;c__Gammaproteobacteria;o__Enterobacteriales;f__Enterobacteriaceae;g__Citrobacter
0.000
0.002
0.004
0.006
0.008
0.010
R
RG
RG
Wo T
CHE
p−value: 0.42 adj. p−value 0.64

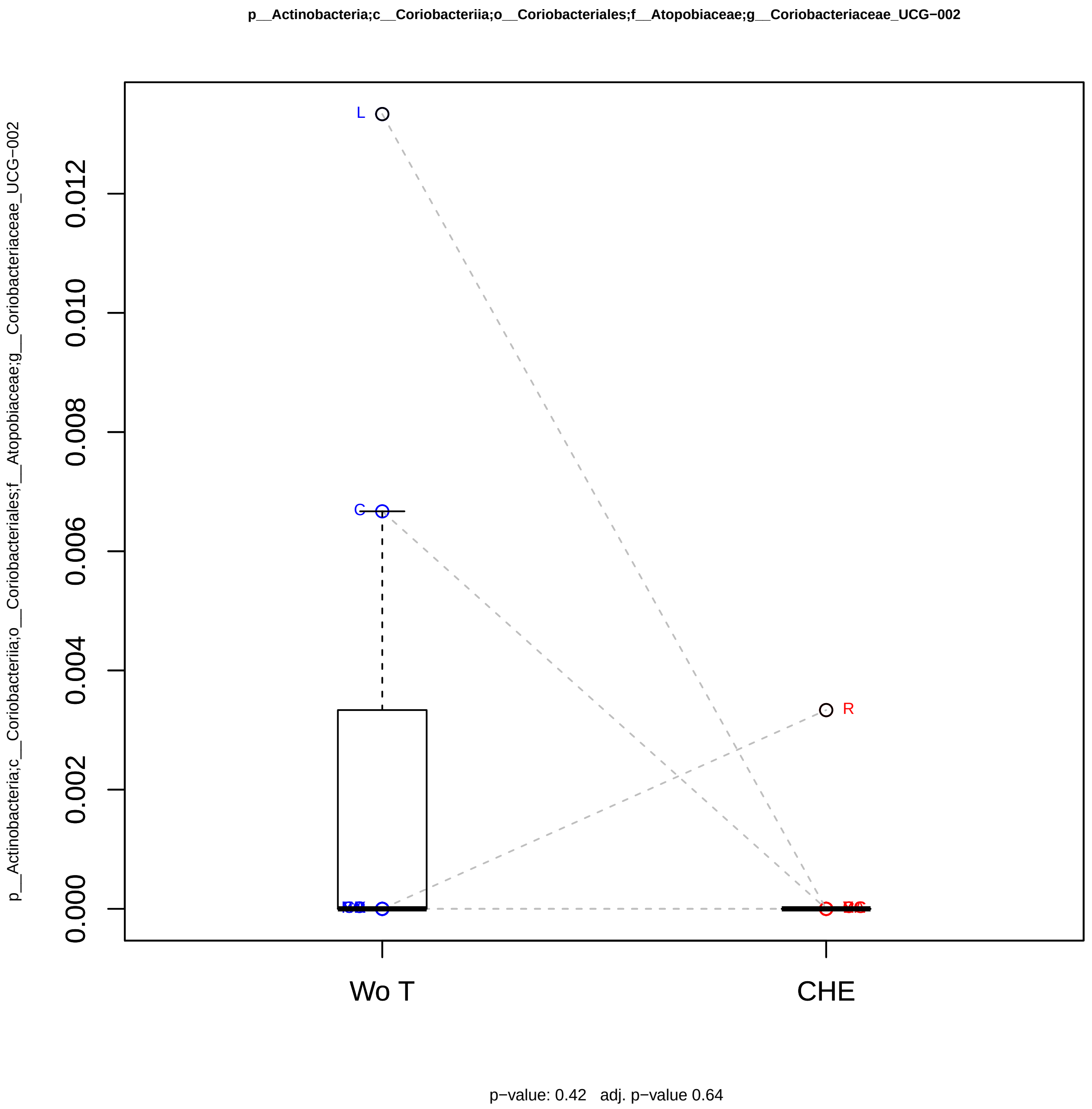
p__Actinobacteria;c__Coriobacteriia;o__Coriobacteriales;f__Atopobiaceae;g__Coriobacteriaceae_UCG−002
p__Actinobacteria;c__Coriobacteriia;o__Coriobacteriales;f__Atopobiaceae;g__Coriobacteriaceae_UCG−002
0.000
0.002
0.004
0.006
0.008
0.010
0.012
L
G
R
Wo T
CHE
p−value: 0.42 adj. p−value 0.64

p__Firmicutes;c__Clostridia;o__Clostridiales;f__Family_XIII;g__Family_XIII_UCG−001

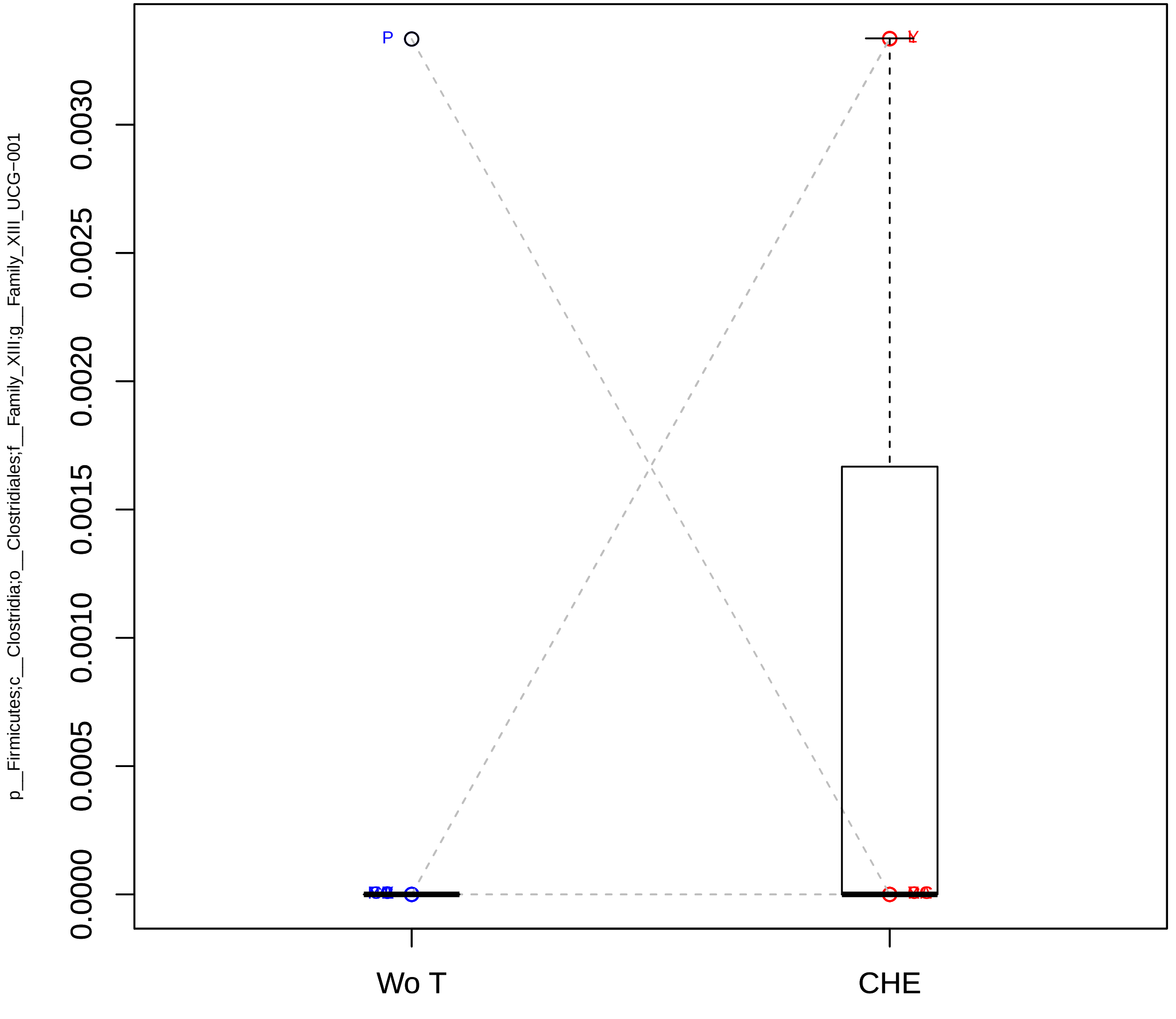


p−value: 0.42   adj. p−value 0.64

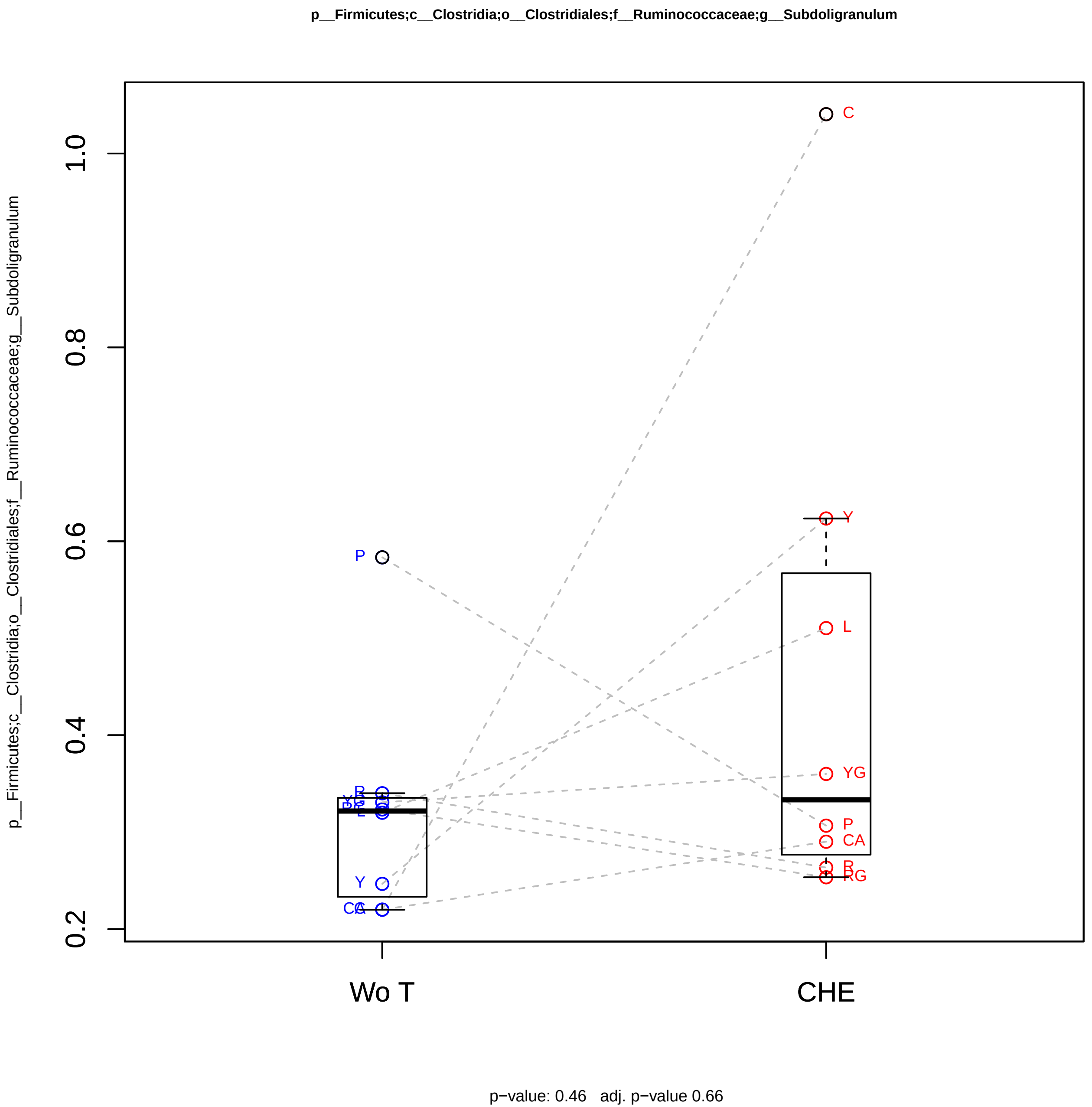
p__Firmicutes;c__Clostridia;o__Clostridiales;f__Ruminococcaceae;g__Subdoligranulum
p__Firmicutes;c__Clostridia;o__Clostridiales;f__Ruminococcaceae;g__Subdoligranulum
0.2
0.4
0.6
0.8
1.0
Wo T
CHE
P
Y
C
L
YG
CA
p−value: 0.46 adj. p−value 0.66

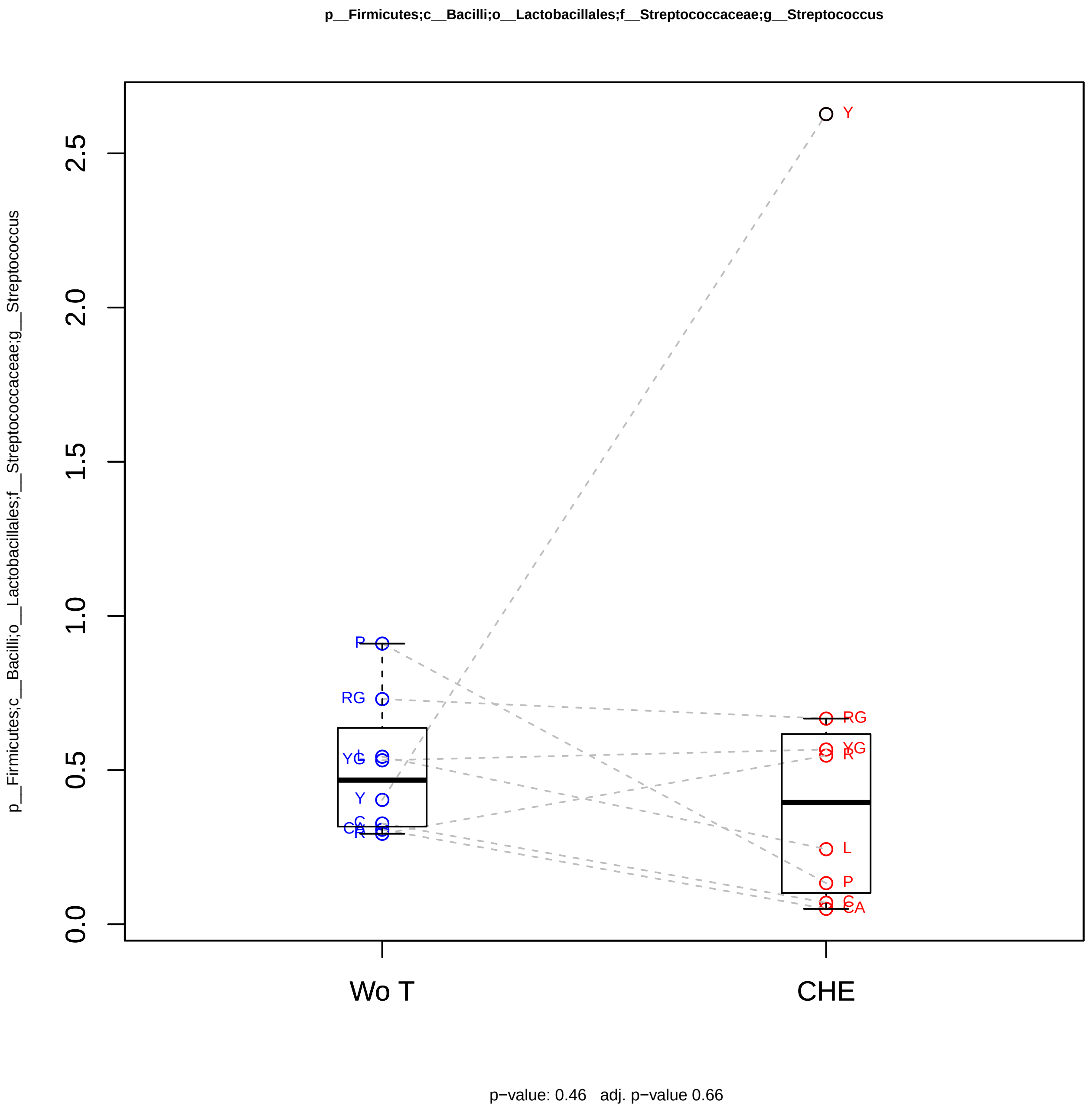
p__Firmicutes;c__Bacilli;o__Lactobacillales;f__Streptococcaceae;g__Streptococcus
p__Firmicutes;c__Bacilli;o__Lactobacillales;f__Streptococcaceae;g__Streptococcus
0.0
0.5
1.0
1.5
2.0
2.5
Wo T
CHE
P
RG
YG
L
Y
C
CA
R
Y
RG
YG
R
L
P
C
CA
p−value: 0.46   adj. p−value 0.66

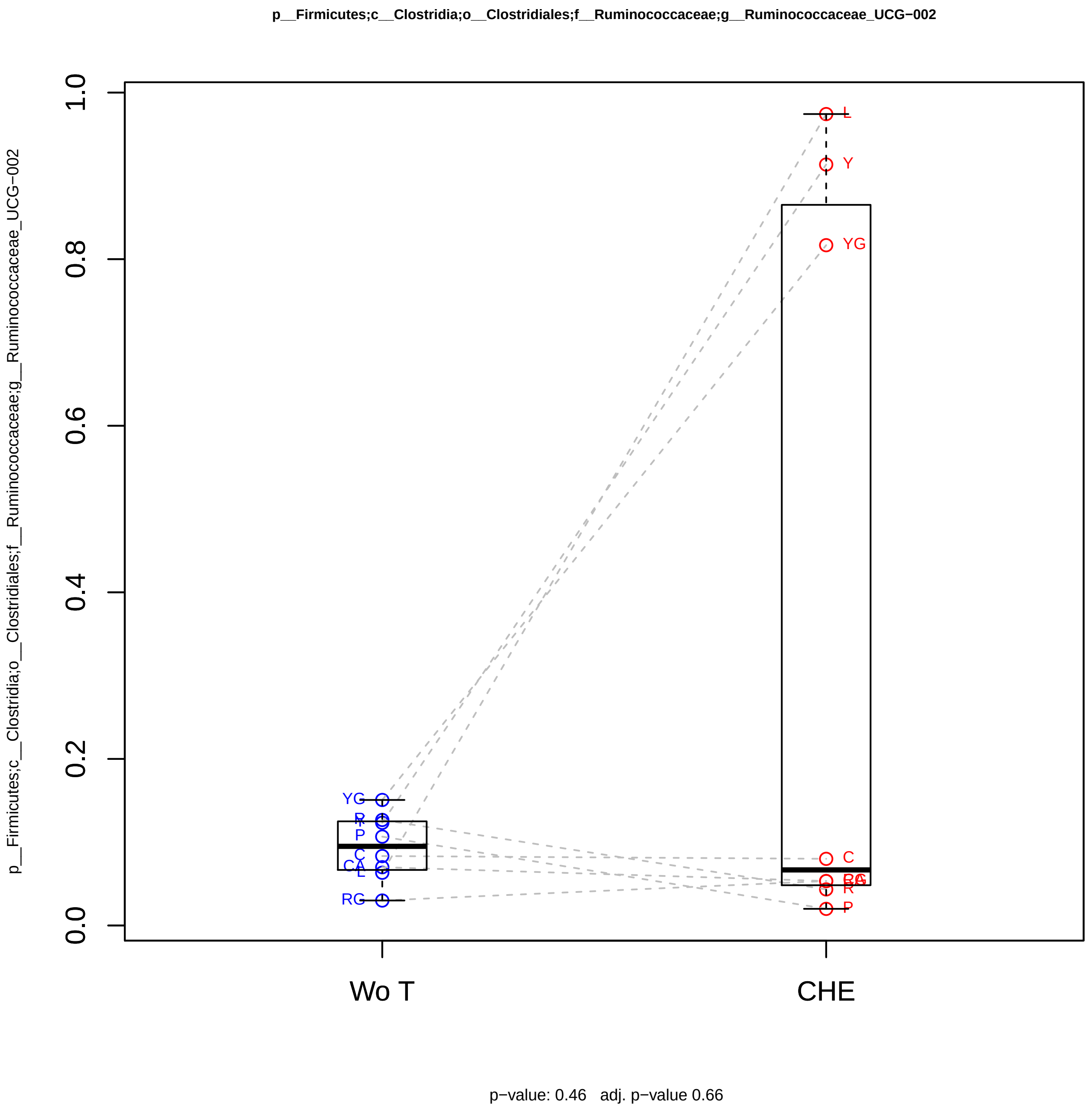
p__Firmicutes;c__Clostridia;o__Clostridiales;f__Ruminococcaceae;g__Ruminococcaceae_UCG−002
p__Firmicutes;c__Clostridia;o__Clostridiales;f__Ruminococcaceae;g__Ruminococcaceae_UCG−002
0.0
0.2
0.4
0.6
0.8
1.0
YG
P
C
L
RG
Y
Wo T
CHE
p−value: 0.46 adj. p−value 0.66

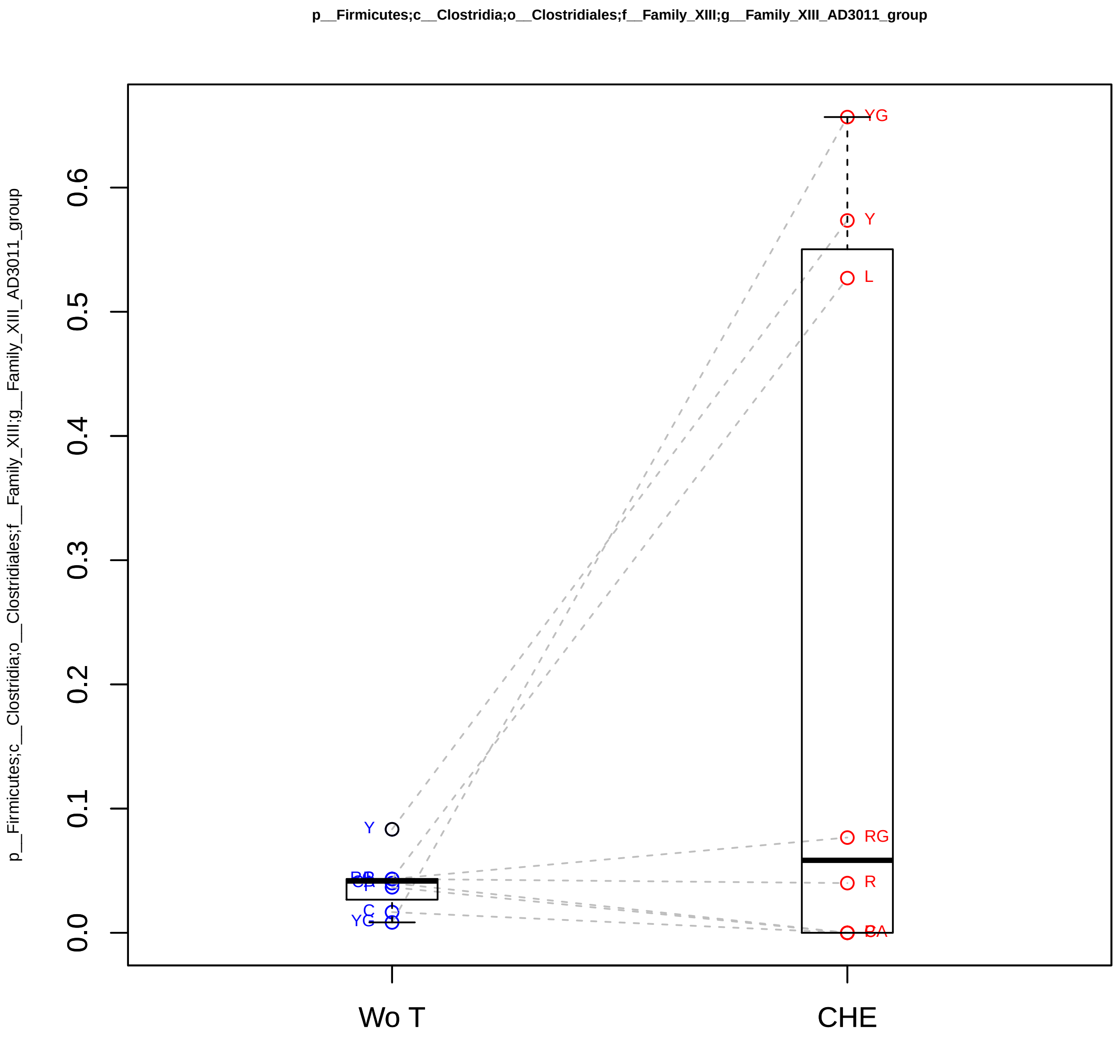


p−value: 0.46 adj. p−value 0.66

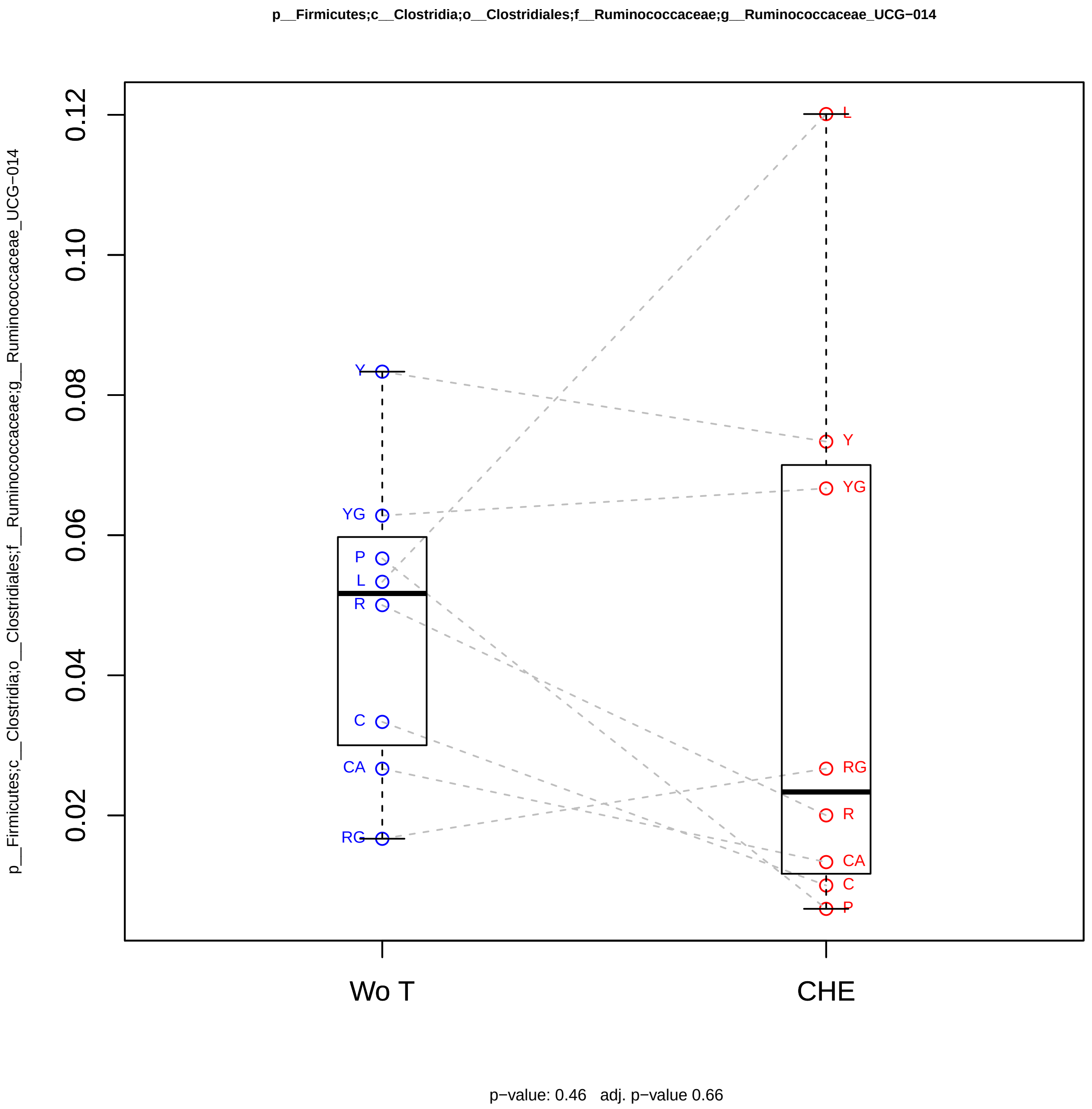

p__Firmicutes;c__Clostridia;o__Clostridiales;f__Ruminococcaceae;g__Ruminococcaceae_UCG−014
p__Firmicutes;c__Clostridia;o__Clostridiales;f__Ruminococcaceae;g__Ruminococcaceae_UCG−014
0.02
0.04
0.06
0.08
0.10
0.12
Y
YG
P
L
R
C
CA
RG
L
Y
YG
RG
R
CA
C
P
Wo T
CHE
p−value: 0.46 adj. p−value 0.66

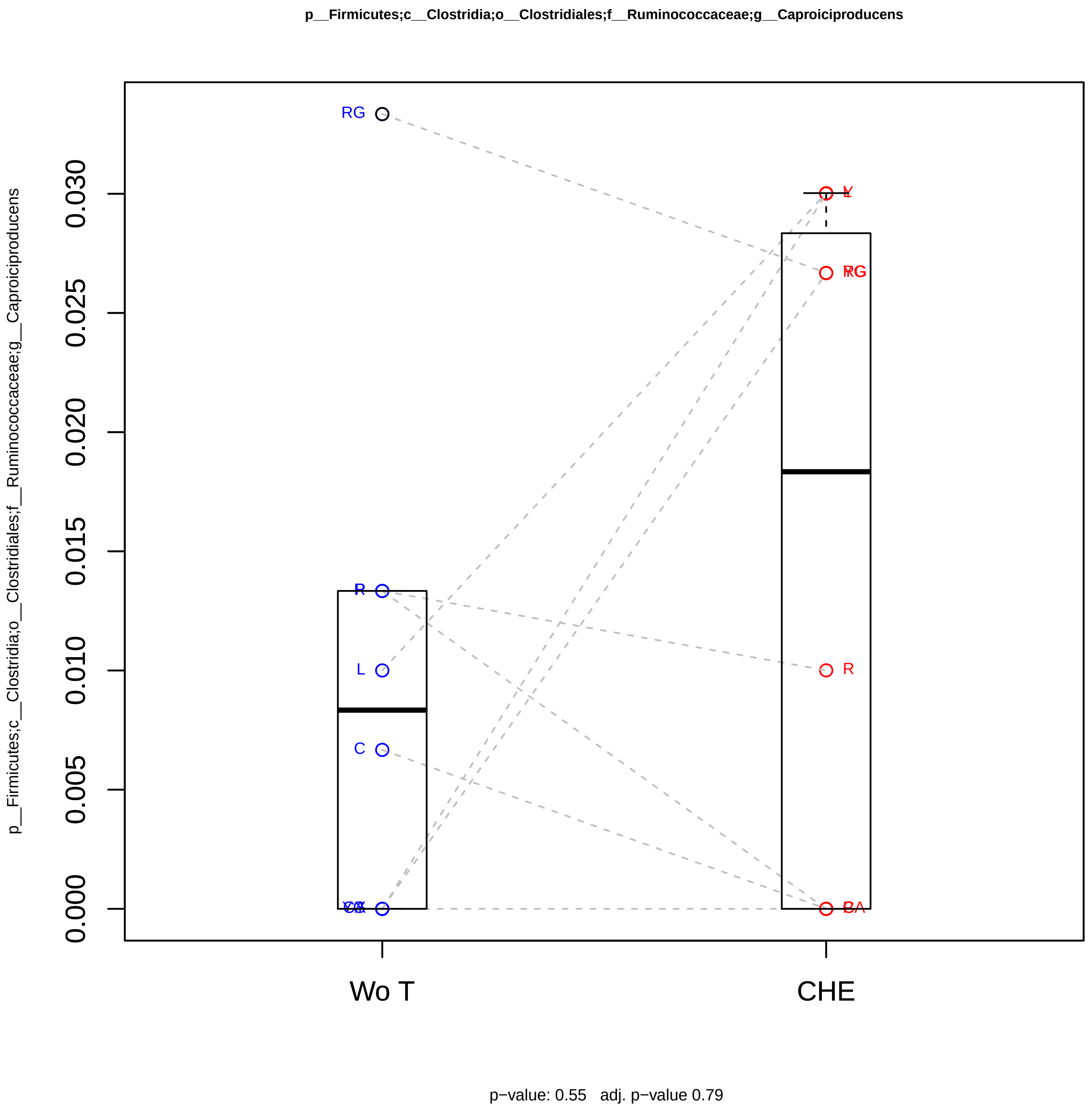
p__Firmicutes;c__Clostridia;o__Clostridiales;f__Ruminococcaceae;g__Caproiciproducens
p__Firmicutes;c__Clostridia;o__Clostridiales;f__Ruminococcaceae;g__Caproiciproducens
0.000
0.005
0.010
0.015
0.020
0.025
0.030
Wo T
CHE
p−value: 0.55 adj. p−value 0.79

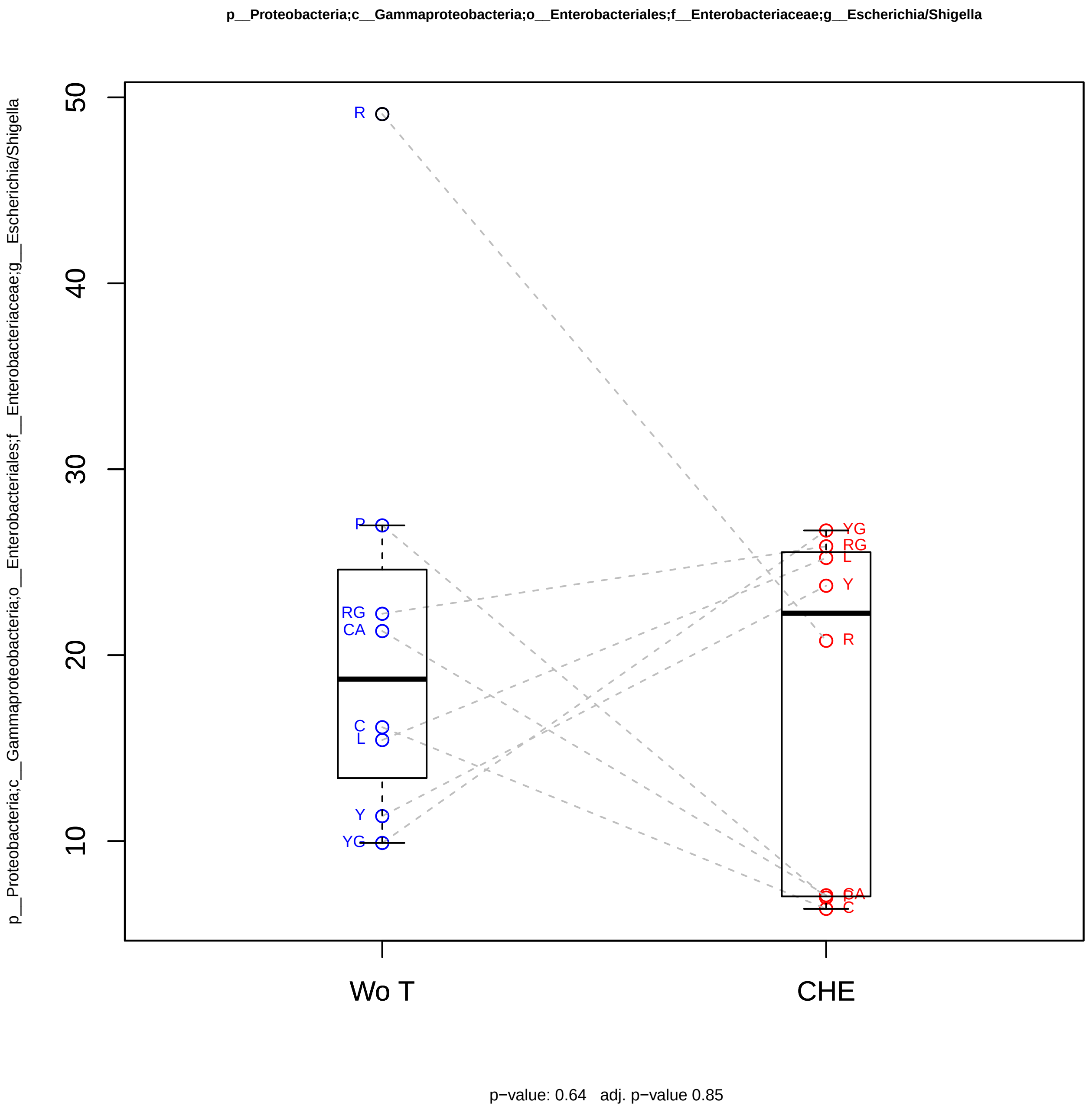
p__Proteobacteria;c__Gammaproteobacteria;o__Enterobacteriales;f__Enterobacteriaceae;g__Escherichia/Shigella
p__Proteobacteria;c__Gammaproteobacteria;o__Enterobacteriales;f__Enterobacteriaceae;g__Escherichia/Shigella
10
20
30
40
50
Wo T
CHE
R
P
RG
CA
C
L
Y
YG
YG
RG
L
Y
R
CA
C
p−value: 0.64 adj. p−value 0.85

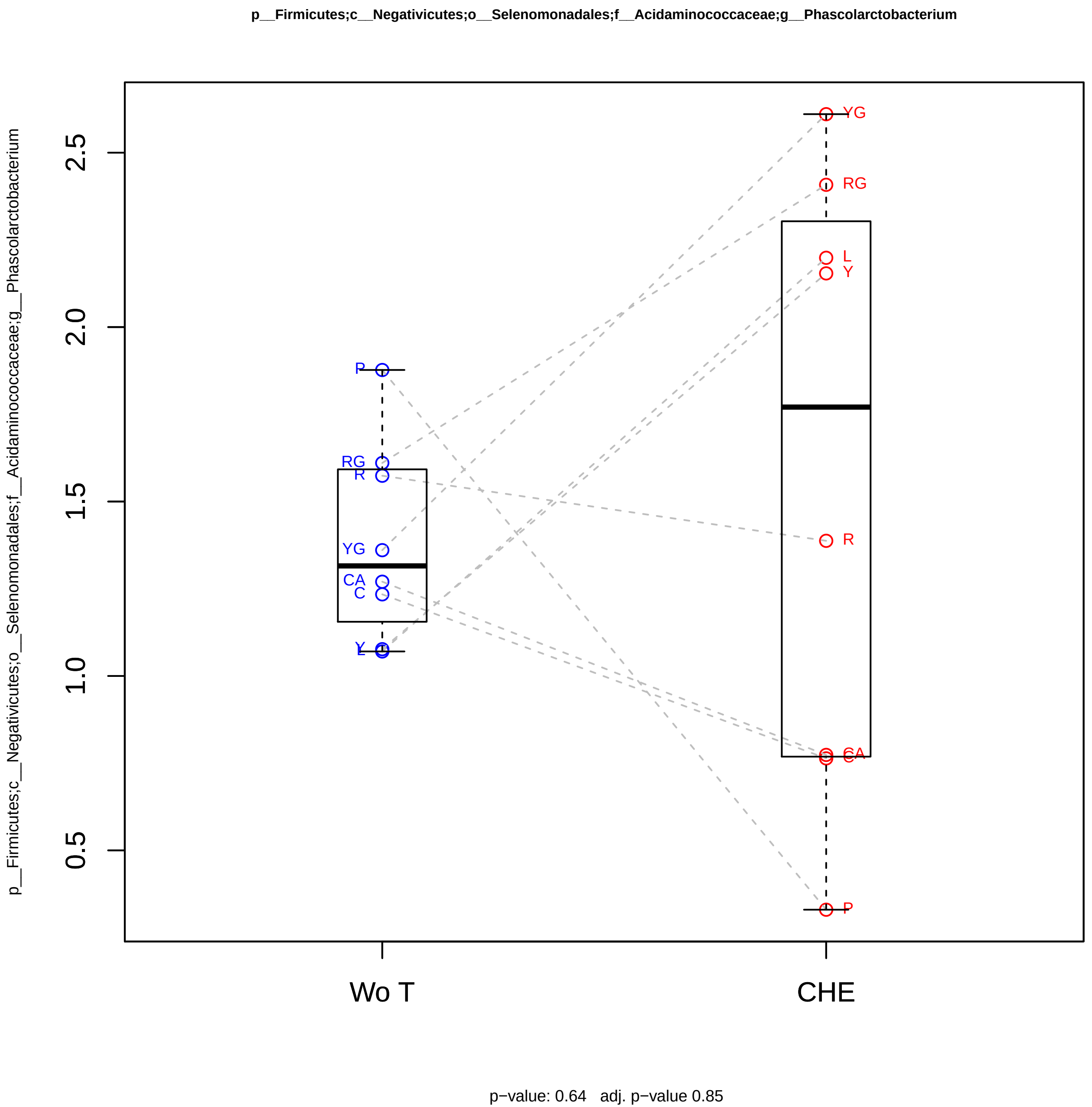

p__Firmicutes;c__Negativicutes;o__Selenomonadales;f__Acidaminococcaceae;g__Phascolarctobacterium
p__Firmicutes;c__Negativicutes;o__Selenomonadales;f__Acidaminococcaceae;g__Phascolarctobacterium
0.5
1.0
1.5
2.0
2.5
P
RG
R
YG
CA
C
Y
L
YG
RG
L
Y
R
CA
C
P
Wo T
CHE
p−value: 0.64 adj. p−value 0.85

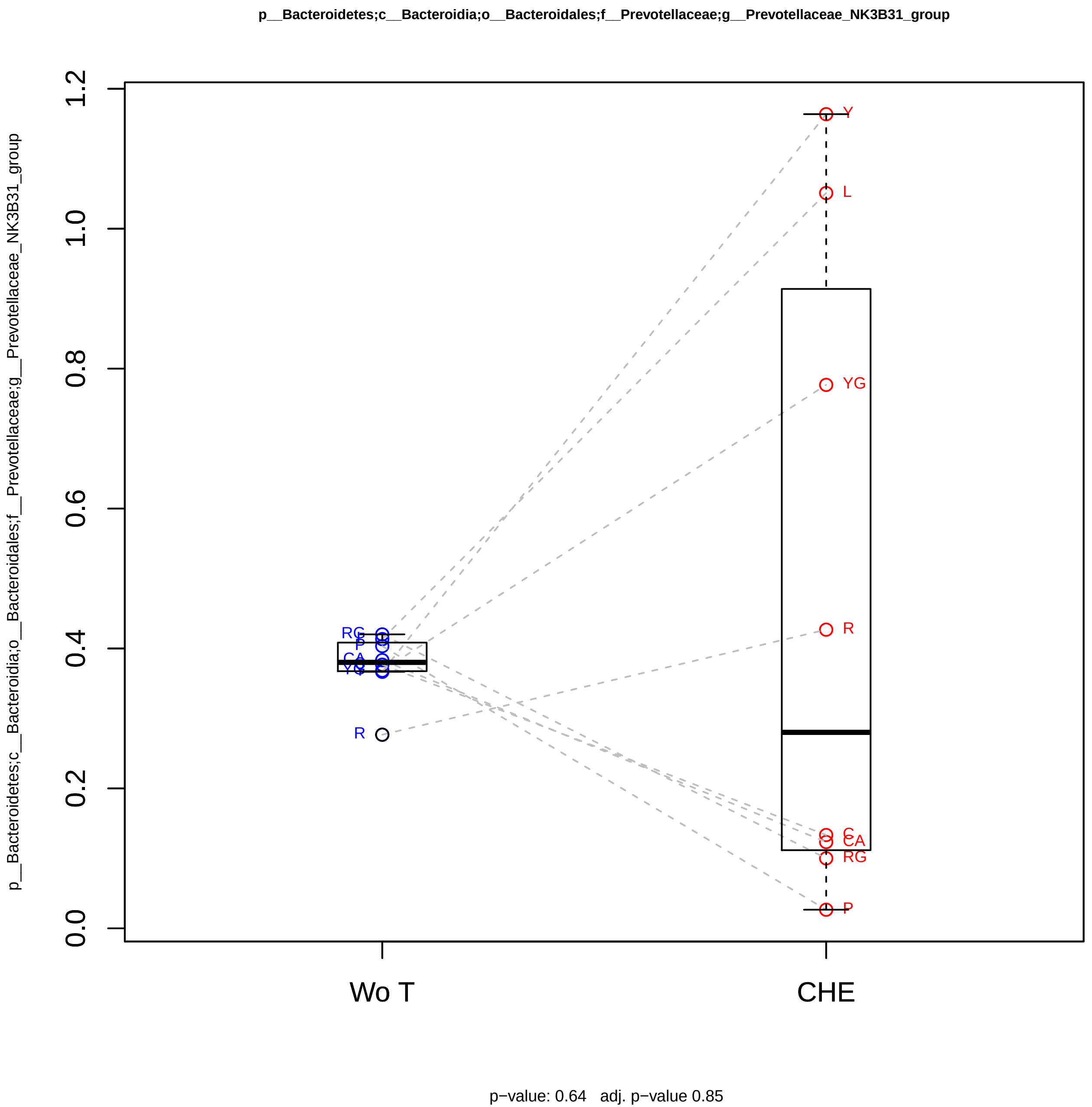
p__Bacteroidetes;c__Bacteroidia;o__Bacteroidales;f__Prevotellaceae;g__Prevotellaceae_NK3B31_group
p__Bacteroidetes;c__Bacteroidia;o__Bacteroidales;f__Prevotellaceae;g__Prevotellaceae_NK3B31_group
0.0
0.2
0.4
0.6
0.8
1.0
1.2
Wo T
CHE
Y
L
YG
R
C
CA
RG
P
p−value: 0.64 adj. p−value 0.85

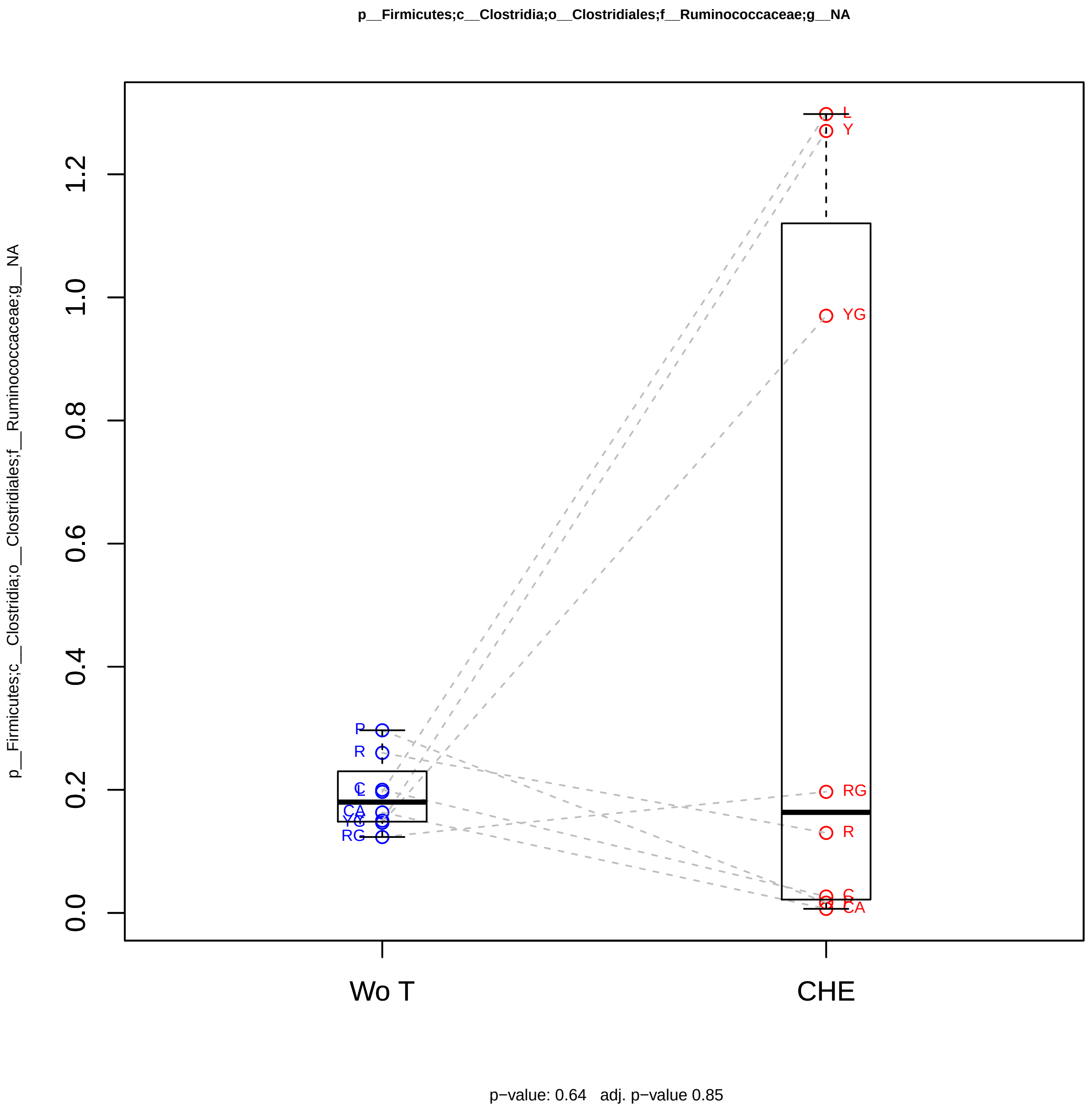

p__Firmicutes;c__Clostridia;o__Clostridiales;f__Ruminococcaceae;g__NA
p__Firmicutes;c__Clostridia;o__Clostridiales;f__Ruminococcaceae;g__NA
0.0
0.2
0.4
0.6
0.8
1.0
1.2
Wo T
CHE
P
R
C
L
CA
YG
RG
L
Y
YG
RG
R
C
CA
p−value: 0.64 adj. p−value 0.85

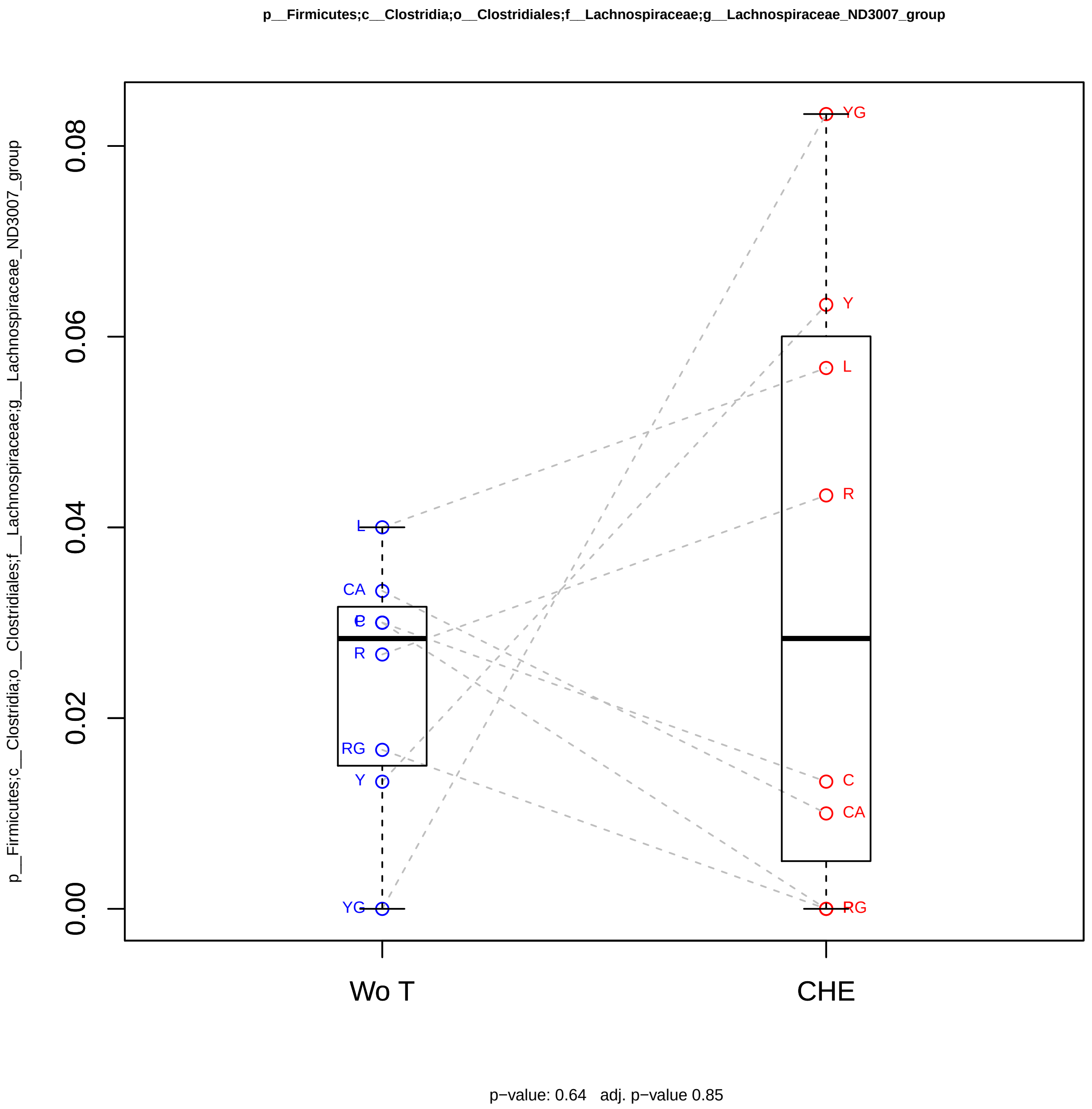

p__Firmicutes;c__Clostridia;o__Clostridiales;f__Lachnospiraceae;g__Lachnospiraceae_ND3007_group
p__Firmicutes;c__Clostridia;o__Clostridiales;f__Lachnospiraceae;g__Lachnospiraceae_ND3007_group
0.00
0.02
0.04
0.06
0.08
L
CA
C
R
RG
Y
YG
YG
Y
L
R
C
CA
RG
Wo T
CHE
p−value: 0.64 adj. p−value 0.85

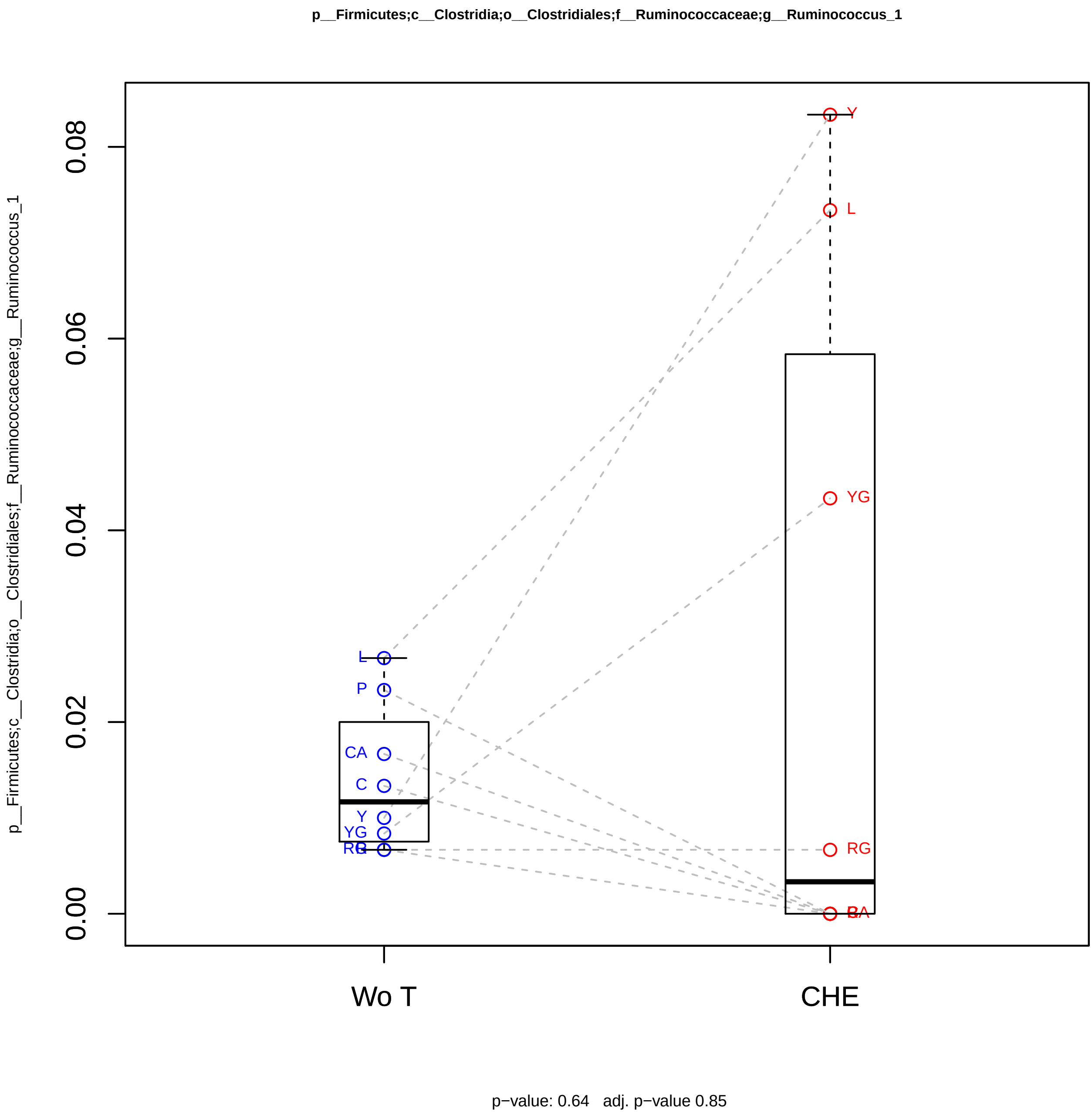
p__Firmicutes;c__Clostridia;o__Clostridiales;f__Ruminococcaceae;g__Ruminococcus_1
p__Firmicutes;c__Clostridia;o__Clostridiales;f__Ruminococcaceae;g__Ruminococcus_1
0.00
0.02
0.04
0.06
0.08
L
P
CA
C
Y
YG
RG
Y
L
YG
RG
Wo T
CHE
p−value: 0.64 adj. p−value 0.85

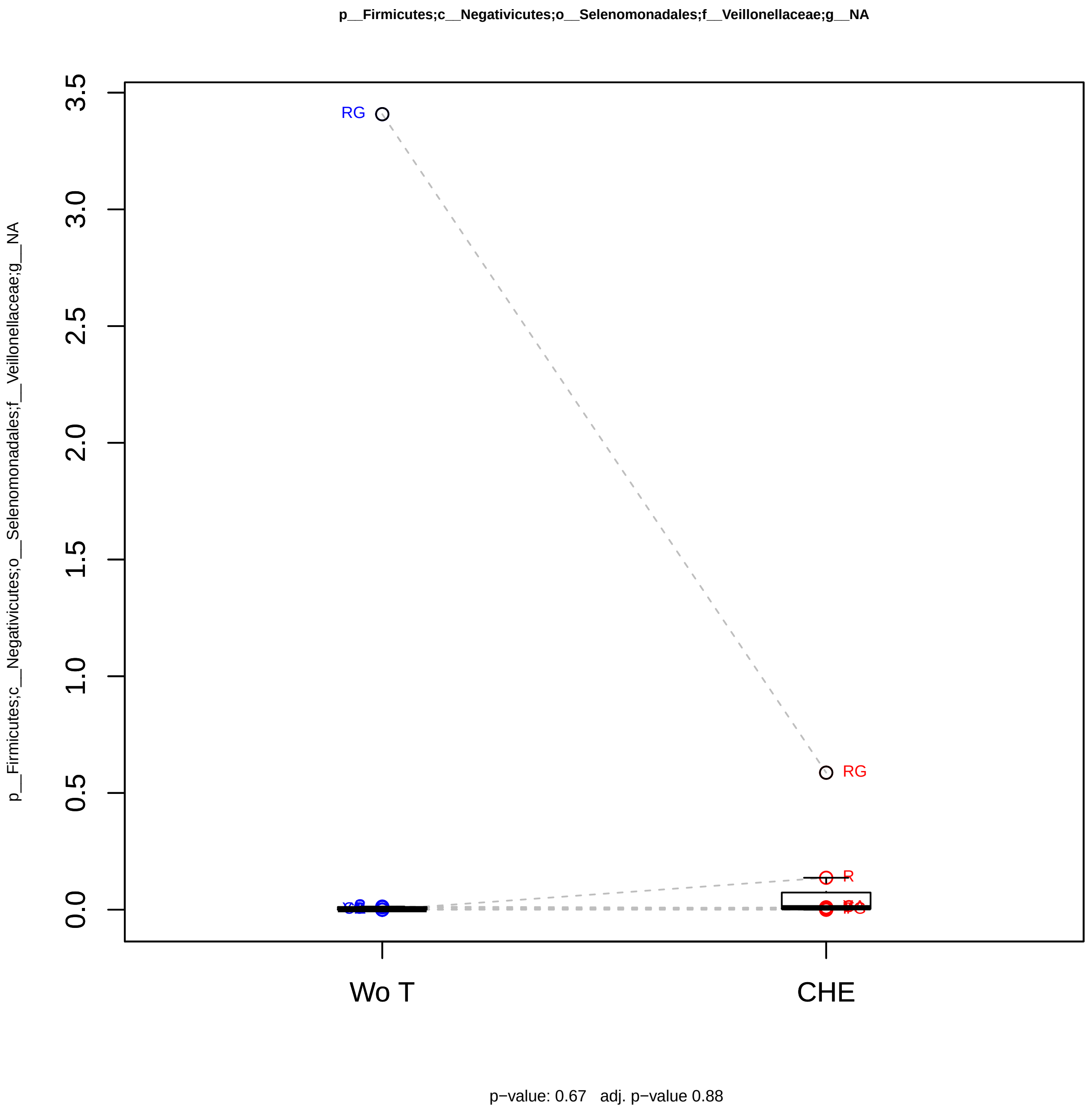
p__Firmicutes;c__Negativicutes;o__Selenomonadales;f__Veillonellaceae;g__NA
p__Firmicutes;c__Negativicutes;o__Selenomonadales;f__Veillonellaceae;g__NA
0.0
0.5
1.0
1.5
2.0
2.5
3.0
3.5
RG
RG
R
Wo T
CHE
p−value: 0.67 adj. p−value 0.88

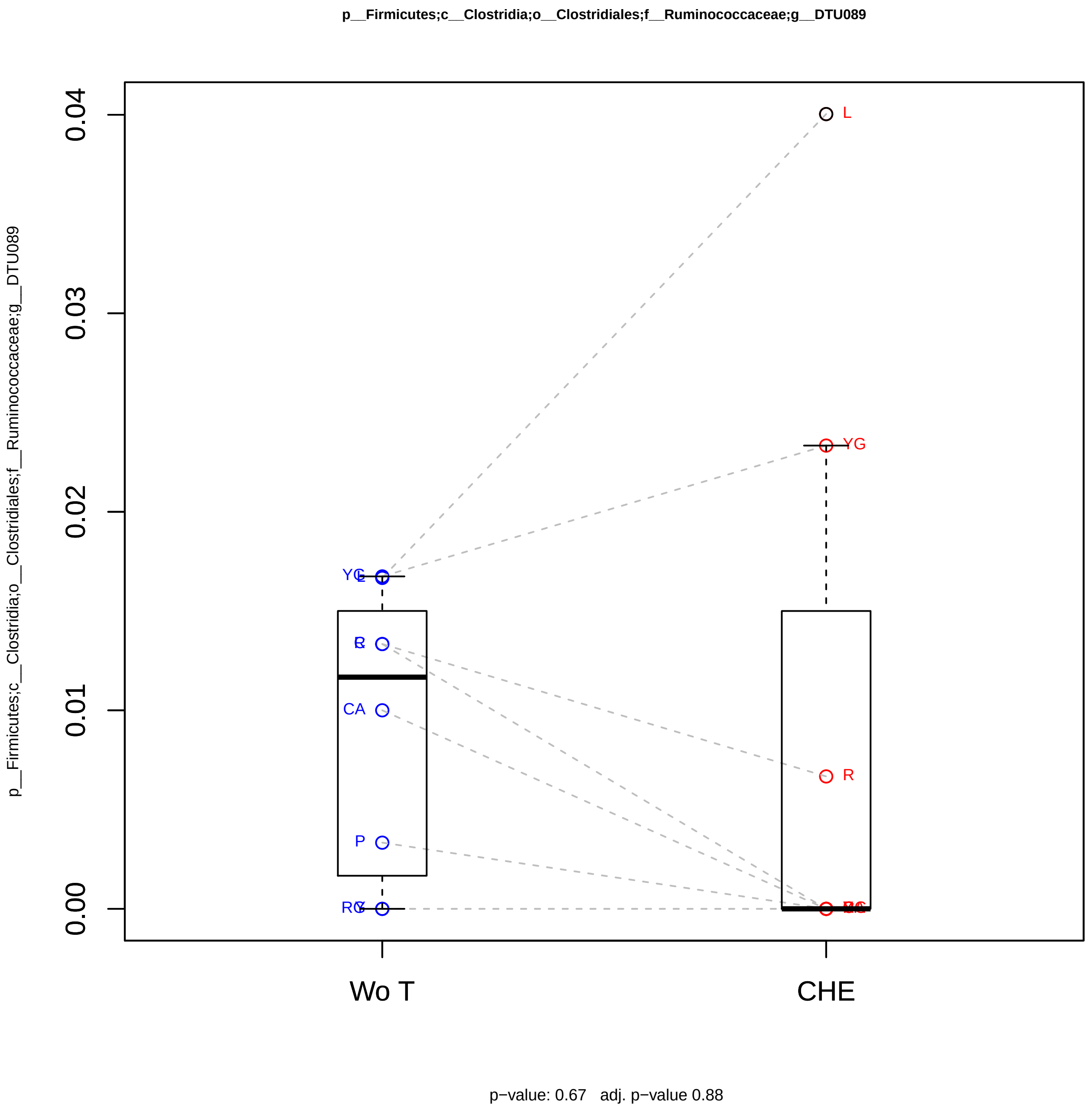
p__Firmicutes;c__Clostridia;o__Clostridiales;f__Ruminococcaceae;g__DTU089
p__Firmicutes;c__Clostridia;o__Clostridiales;f__Ruminococcaceae;g__DTU089
0.00
0.01
0.02
0.03
0.04
Wo T
CHE
L
YG
R
CA
P
p−value: 0.67 adj. p−value 0.88

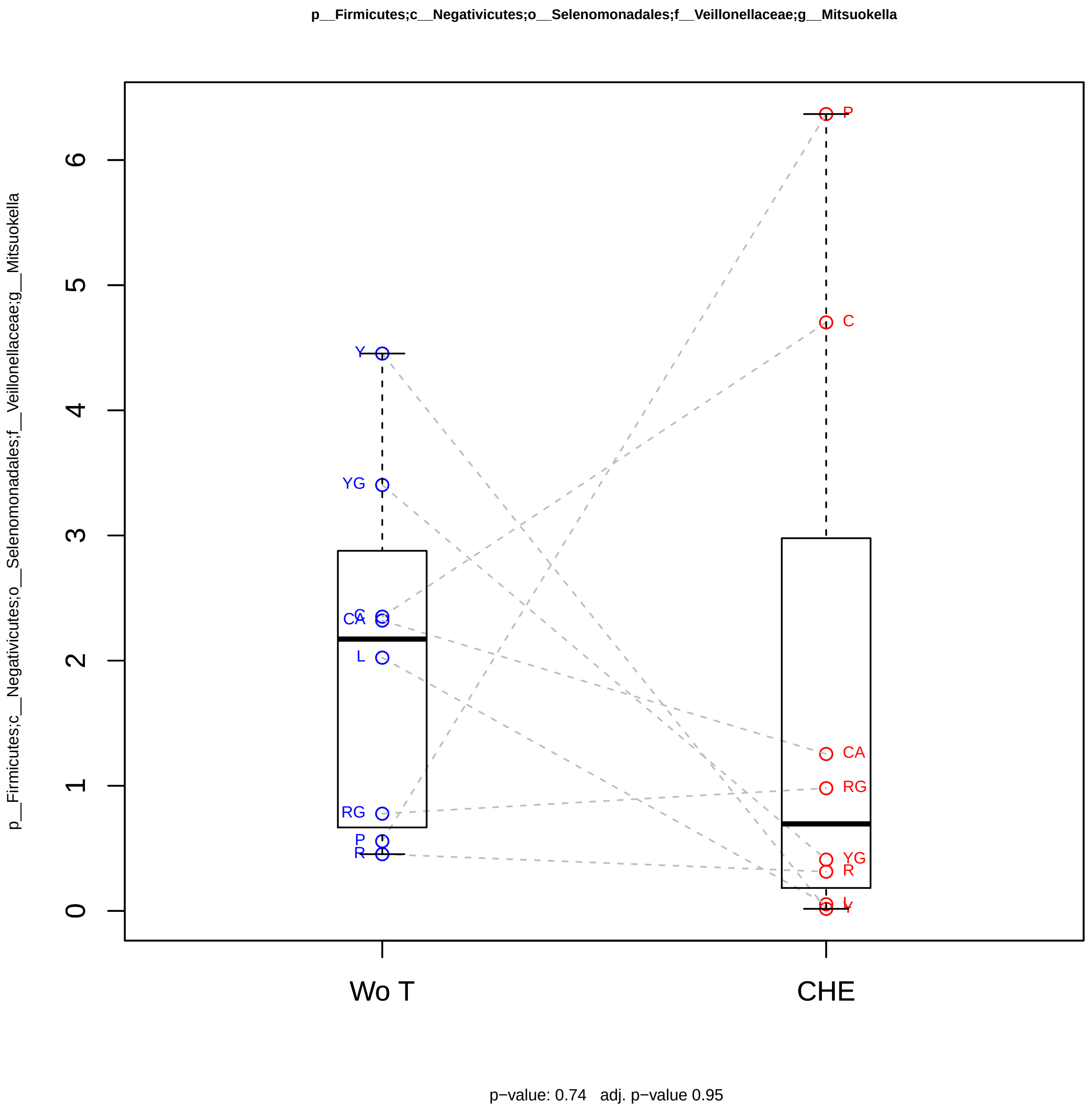
p__Firmicutes;c__Negativicutes;o__Selenomonadales;f__Veillonellaceae;g__Mitsuokella
p__Firmicutes;c__Negativicutes;o__Selenomonadales;f__Veillonellaceae;g__Mitsuokella
0
1
2
3
4
5
6
Y
YG
C
CA
L
RG
P
R
P
C
CA
RG
YG
R
L
Y
Wo T
CHE
p−value: 0.74 adj. p−value 0.95

p__Firmicutes;c__Clostridia;o__Clostridiales;f__Lachnospiraceae;g__Fusicatenibacter

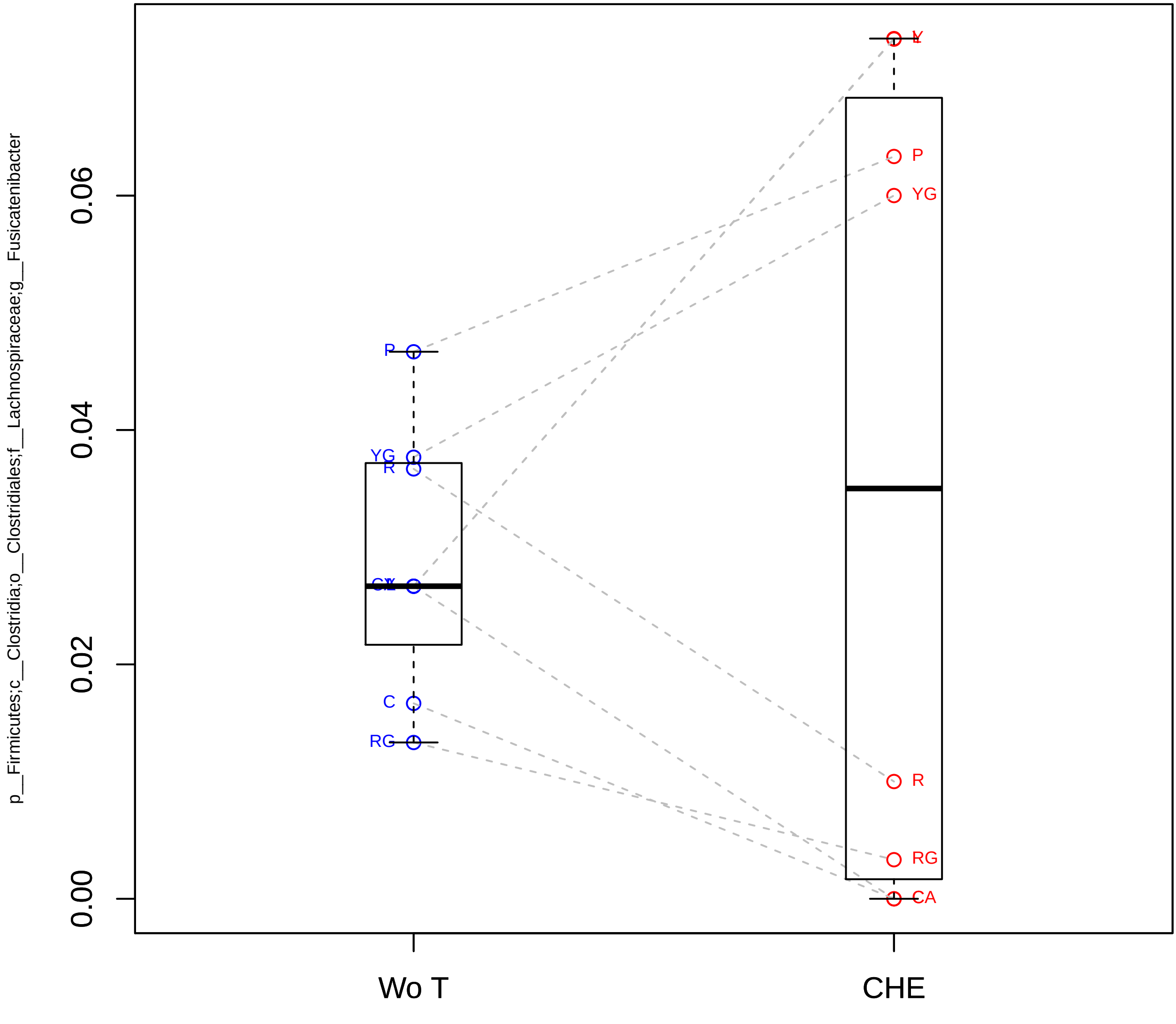


p−value: 0.74 adj. p−value 0.95

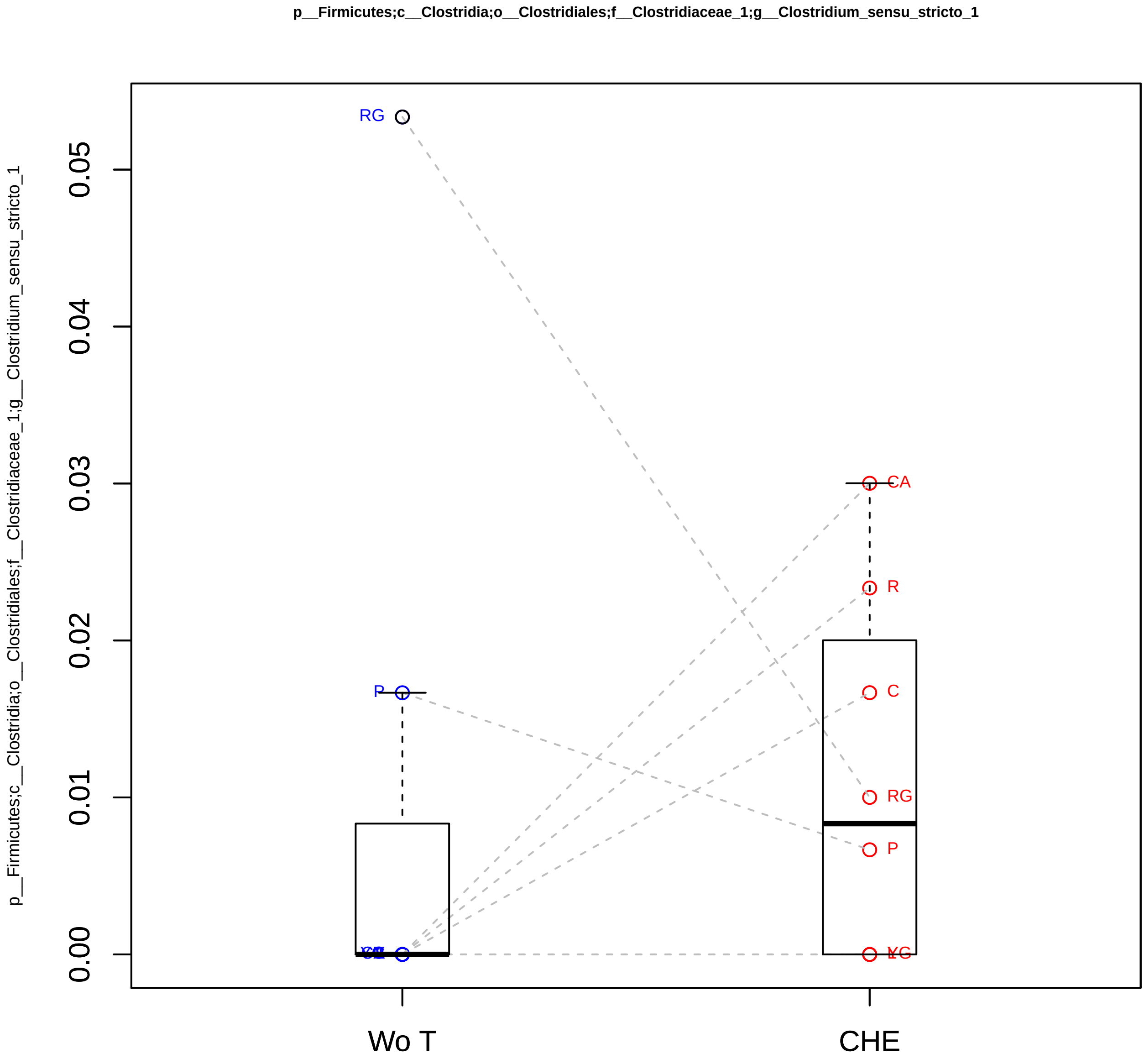


p−value: 0.79 adj. p−value 1

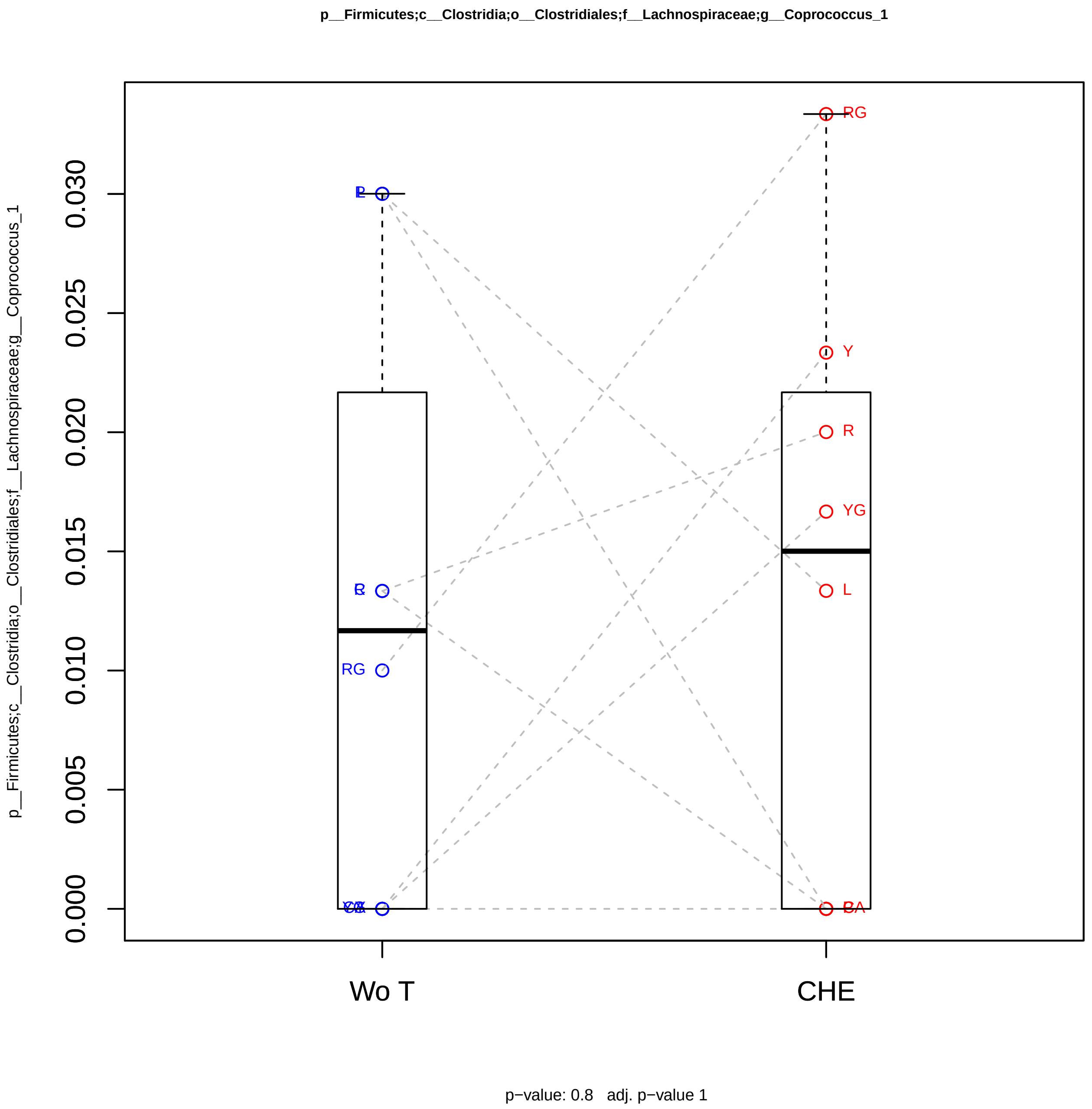

p__Firmicutes;c__Clostridia;o__Clostridiales;f__Lachnospiraceae;g__Coprococcus_1
p__Firmicutes;c__Clostridia;o__Clostridiales;f__Lachnospiraceae;g__Coprococcus_1
0.000
0.005
0.010
0.015
0.020
0.025
0.030
Wo T
CHE
RG
Y
R
YG
L
p−value: 0.8 adj. p−value 1

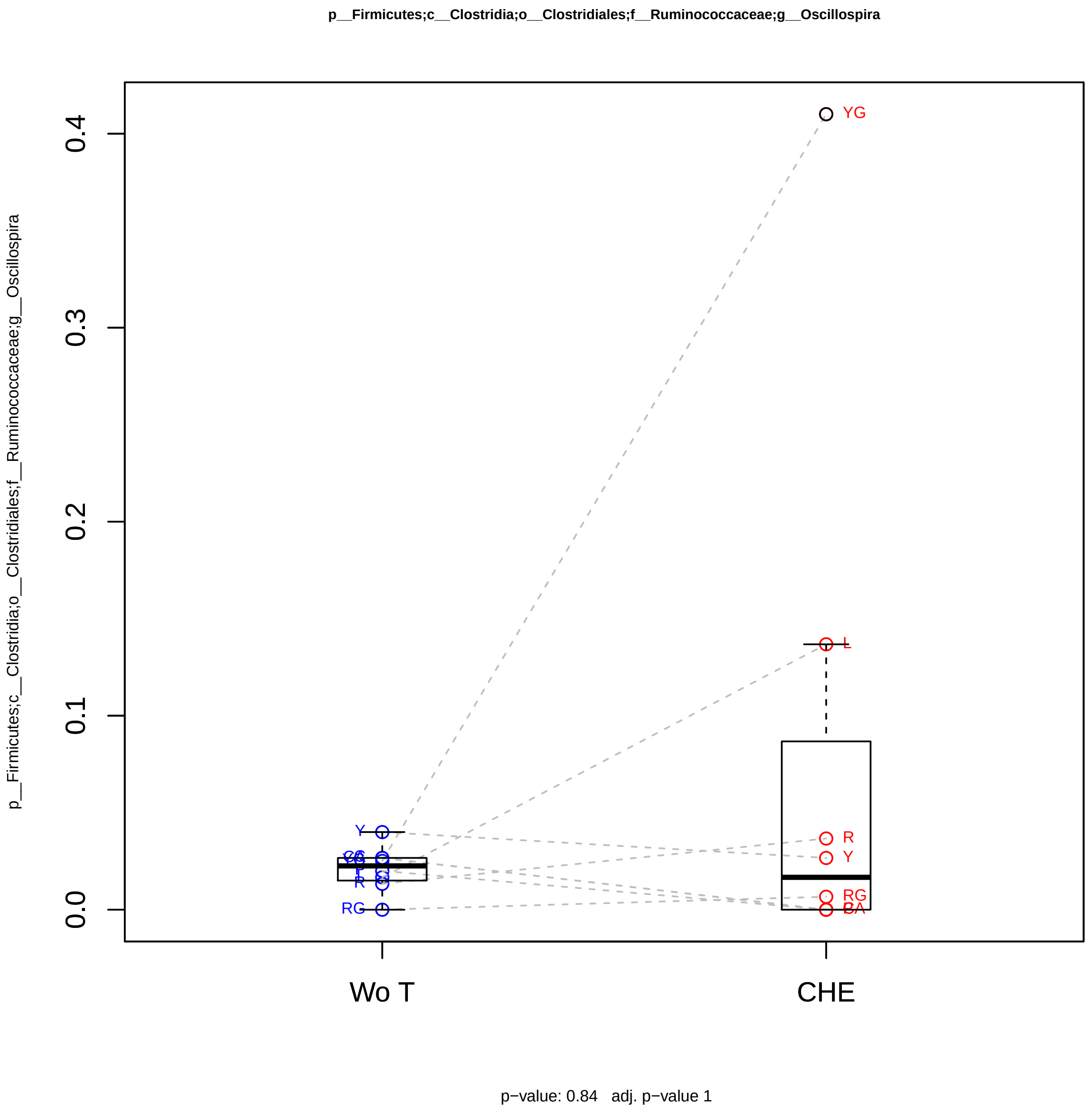
p__Firmicutes;c__Clostridia;o__Clostridiales;f__Ruminococcaceae;g__Oscillospira
p__Firmicutes;c__Clostridia;o__Clostridiales;f__Ruminococcaceae;g__Oscillospira
0.0
0.1
0.2
0.3
0.4
Wo T
CHE
YG
L
R
Y
RG
Y
R
RG
p−value: 0.84 adj. p−value 1

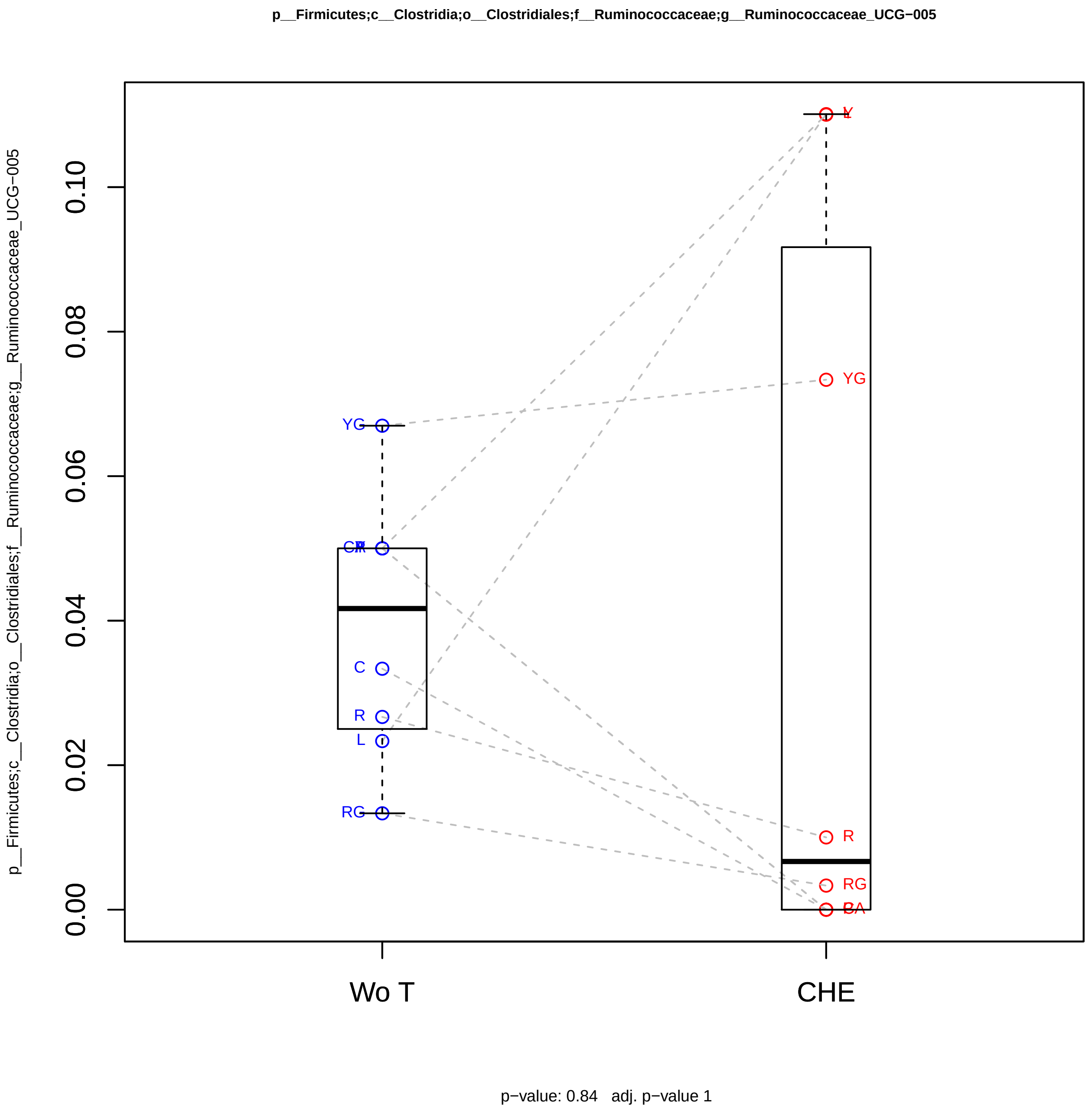
p__Firmicutes;c__Clostridia;o__Clostridiales;f__Ruminococcaceae;g__Ruminococcaceae_UCG−005
p__Firmicutes;c__Clostridia;o__Clostridiales;f__Ruminococcaceae;g__Ruminococcaceae_UCG−005
0.00
0.02
0.04
0.06
0.08
0.10
YG
C
R
L
RG
R
YG
Wo T
CHE
p−value: 0.84 adj. p−value 1

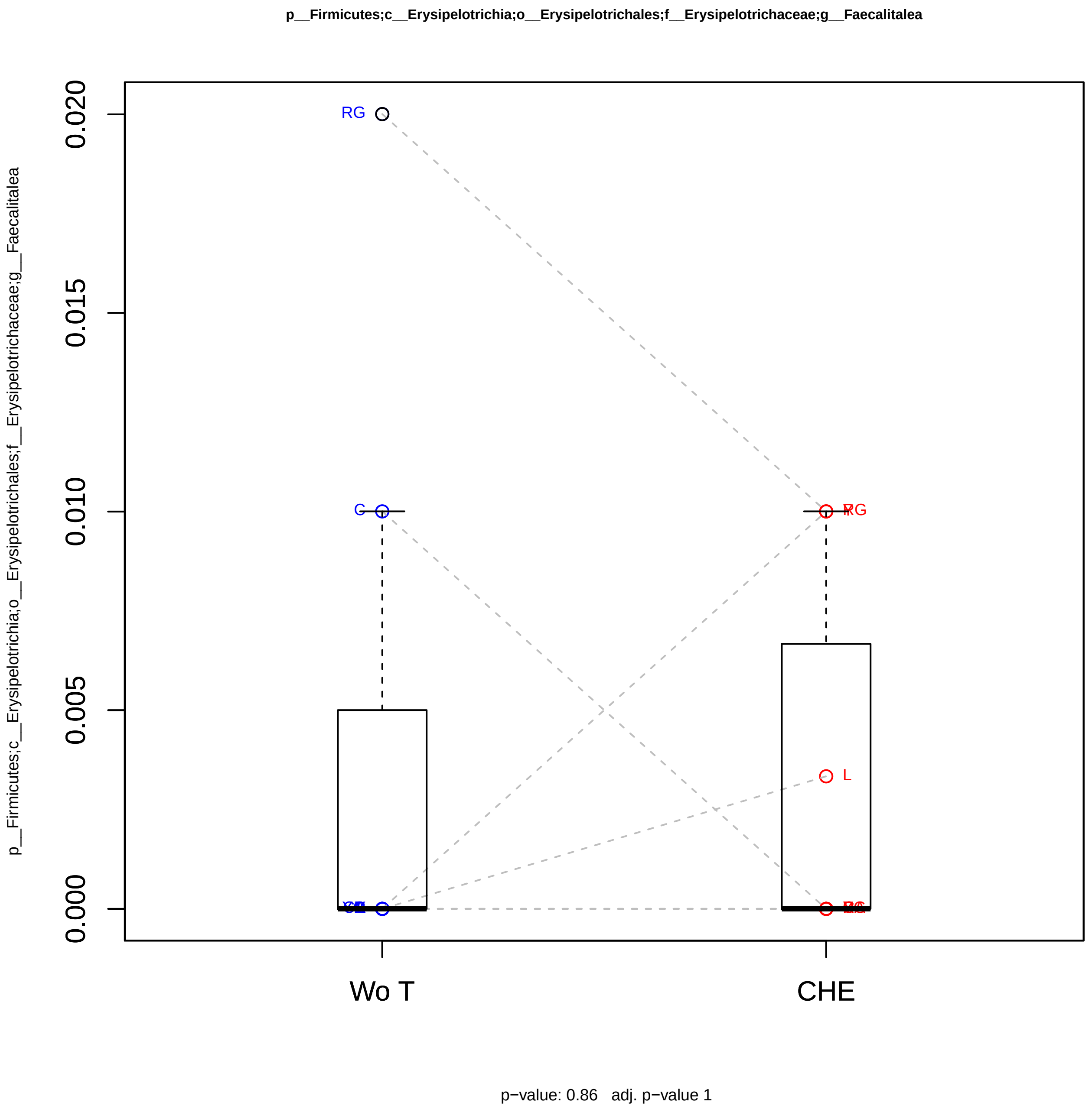

p__Firmicutes;c__Erysipelotrichia;o__Erysipelotrichales;f__Erysipelotrichaceae;g__Faecalitalea
p__Firmicutes;c__Erysipelotrichia;o__Erysipelotrichales;f__Erysipelotrichaceae;g__Faecalitalea
0.000
0.005
0.010
0.015
0.020
RG
C
L
Wo T
CHE
p−value: 0.86 adj. p−value 1

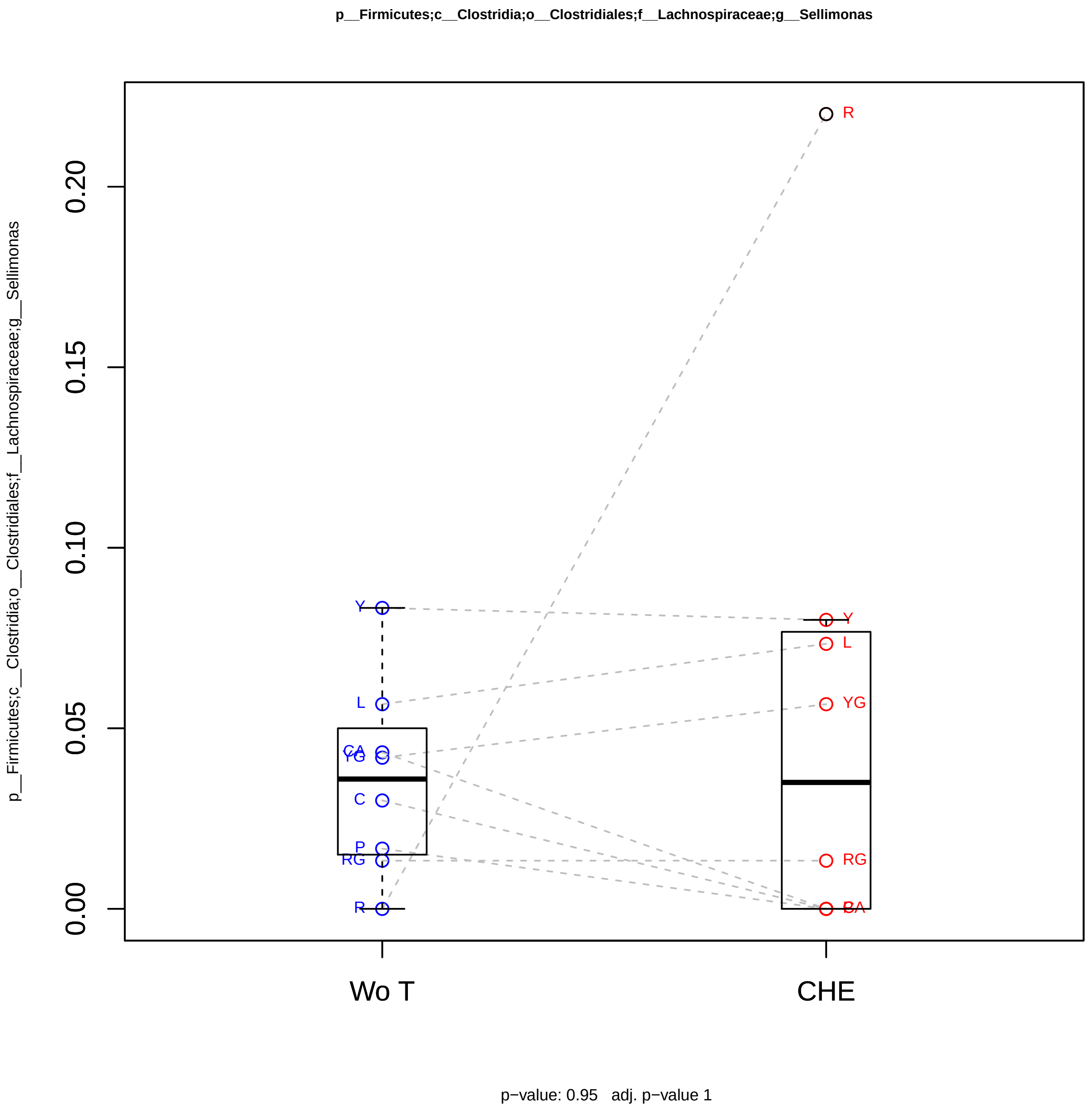
p__Firmicutes;c__Clostridia;o__Clostridiales;f__Lachnospiraceae;g__Sellimonas
p__Firmicutes;c__Clostridia;o__Clostridiales;f__Lachnospiraceae;g__Sellimonas
0.00
0.05
0.10
0.15
0.20
Y
L
CA
YG
C
P
RG
R
R
Y
L
YG
RG
CA
Wo T
CHE
p−value: 0.95 adj. p−value 1

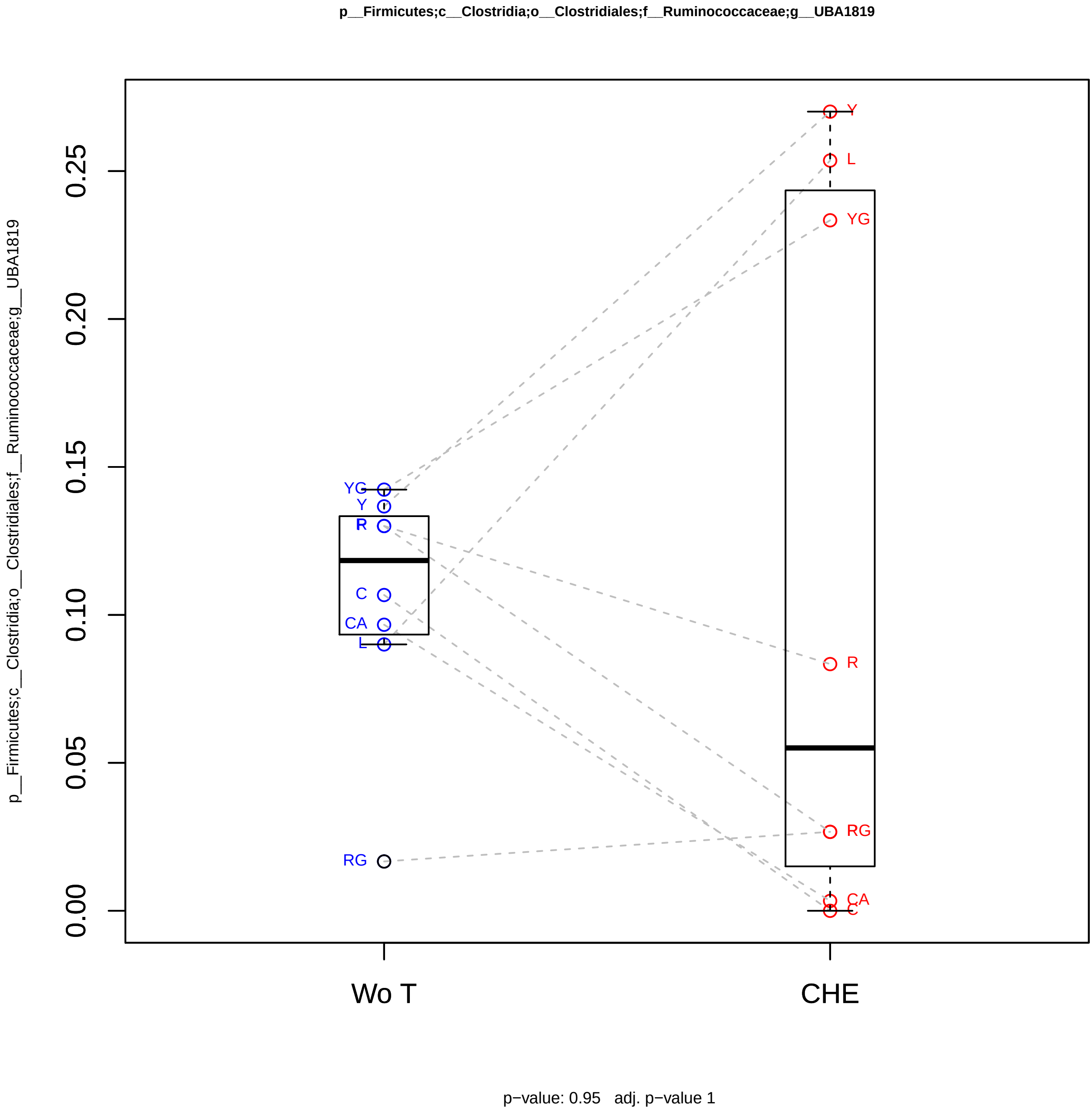
p__Firmicutes;c__Clostridia;o__Clostridiales;f__Ruminococcaceae;g__UBA1819
p__Firmicutes;c__Clostridia;o__Clostridiales;f__Ruminococcaceae;g__UBA1819
0.00
0.05
0.10
0.15
0.20
0.25
YG
Y
R
C
CA
L
RG
Y
L
YG
R
RG
CA
C
Wo T
CHE
p−value: 0.95 adj. p−value 1

p__Firmicutes;c__Clostridia;o__Clostridiales;f__Ruminococcaceae;g__Ruminiclostridium_5

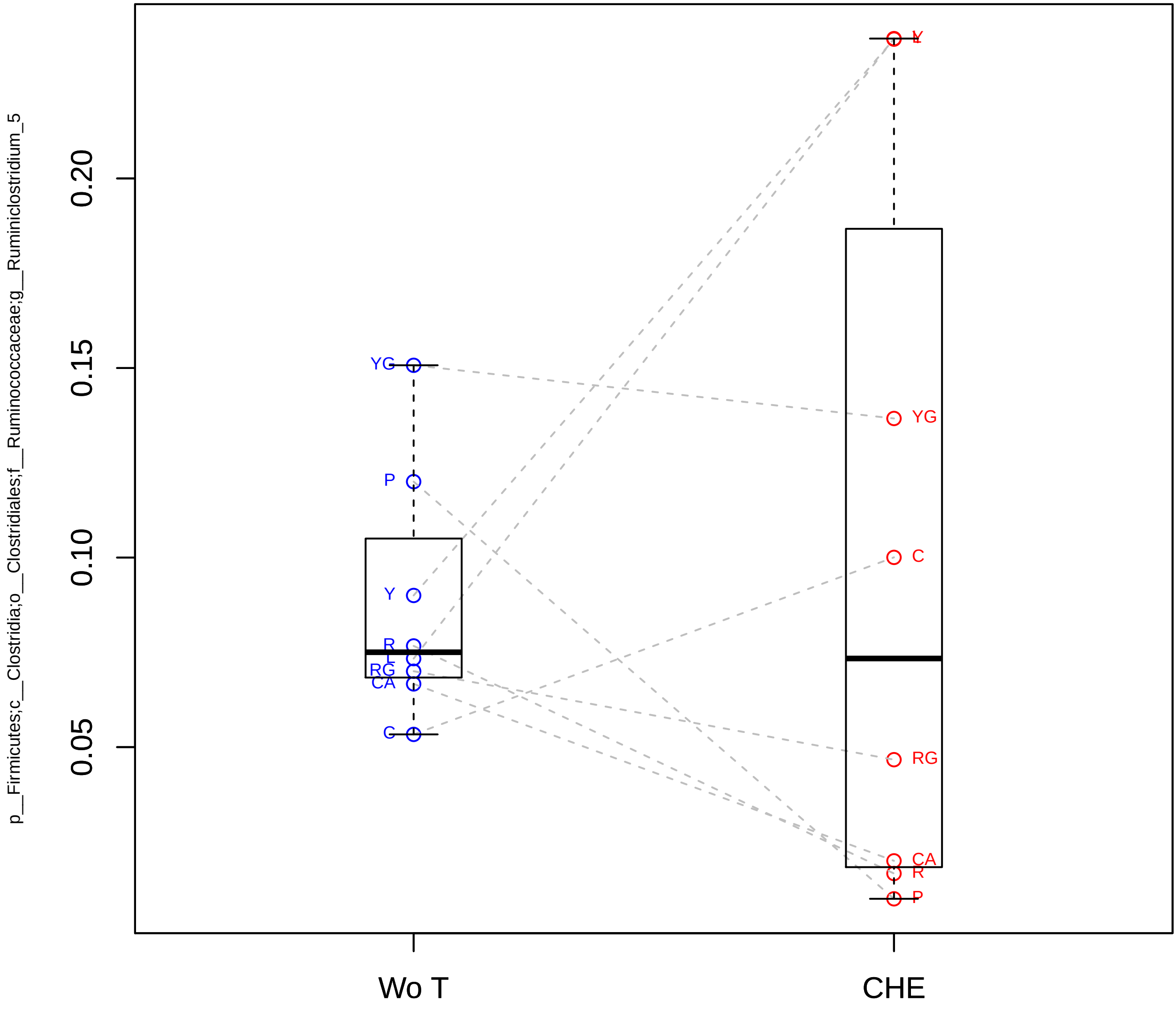


p−value: 0.95 adj. p−value 1

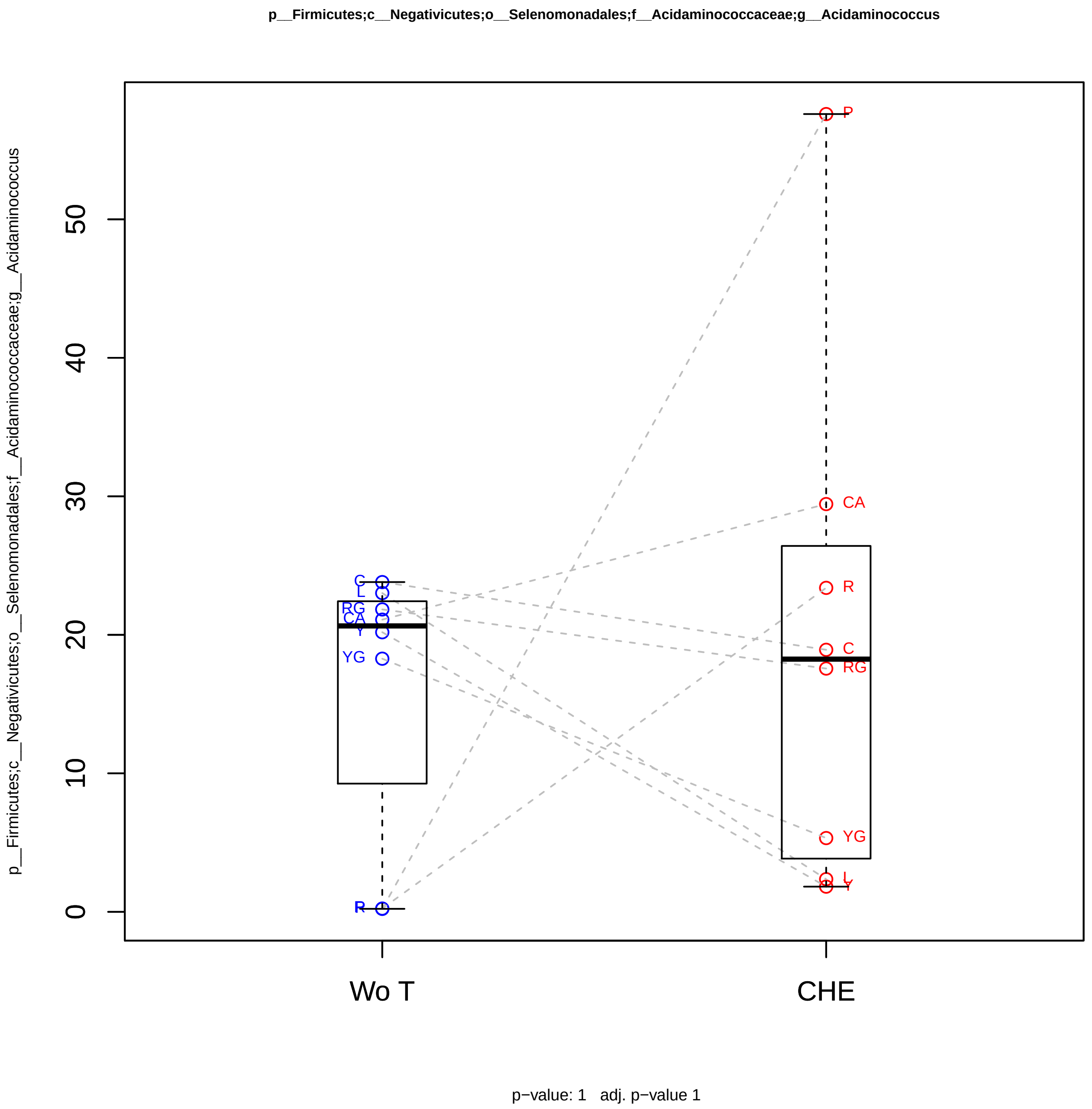

p__Firmicutes;c__Negativicutes;o__Selenomonadales;f__Acidaminococcaceae;g__Acidaminococcus
p__Firmicutes;c__Negativicutes;o__Selenomonadales;f__Acidaminococcaceae;g__Acidaminococcus
0
10
20
30
40
50
C
L
RG
CA
Y
YG
R
P
CA
R
C
RG
YG
L
Y
Wo T
CHE
p−value: 1 adj. p−value 1

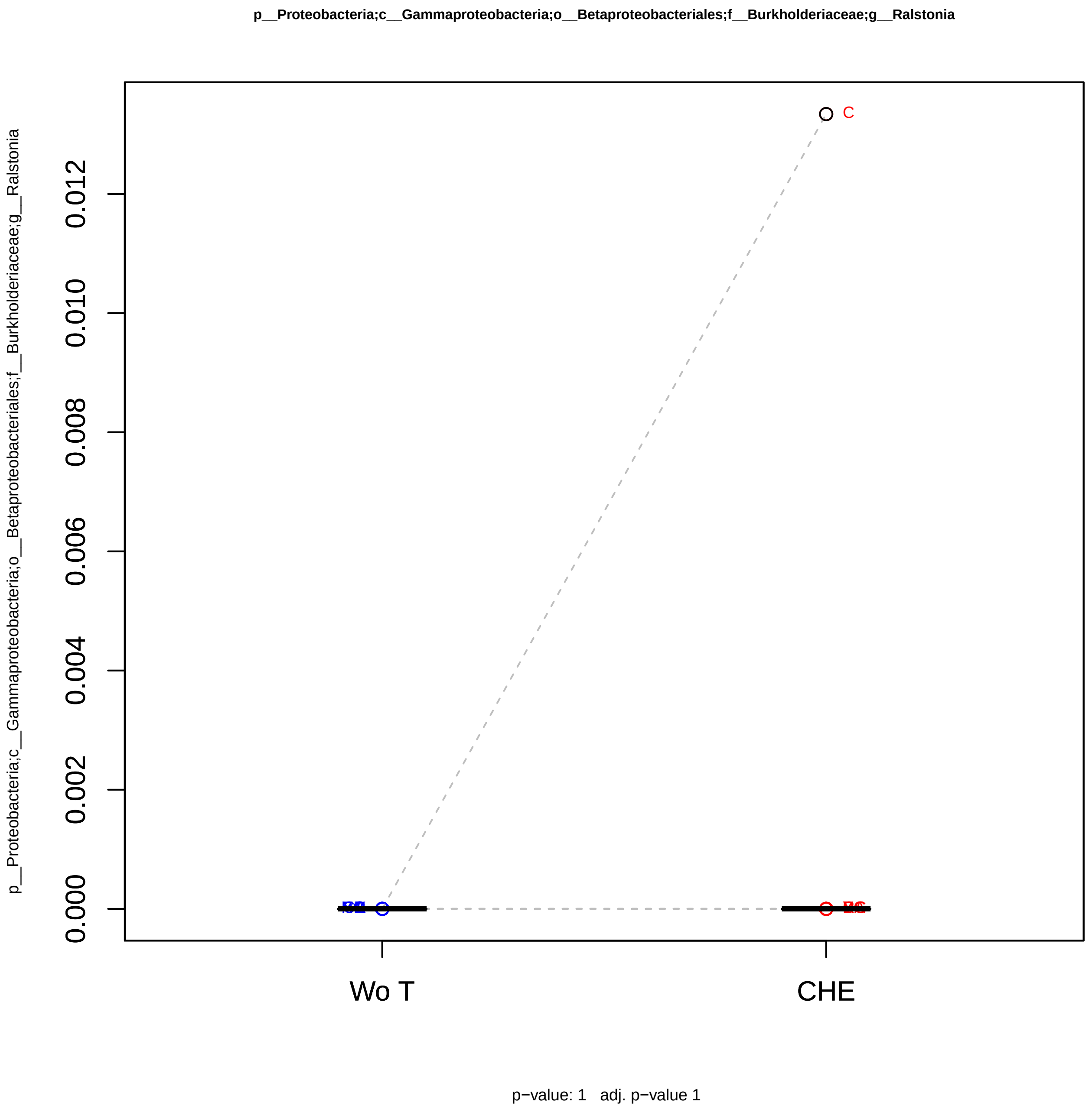
p__Proteobacteria;c__Gammaproteobacteria;o__Betaproteobacteriales;f__Burkholderiaceae;g__Ralstonia
p__Proteobacteria;c__Gammaproteobacteria;o__Betaproteobacteriales;f__Burkholderiaceae;g__Ralstonia
0.000
0.002
0.004
0.006
0.008
0.010
0.012
C
Wo T
CHE
p−value: 1 adj. p−value 1

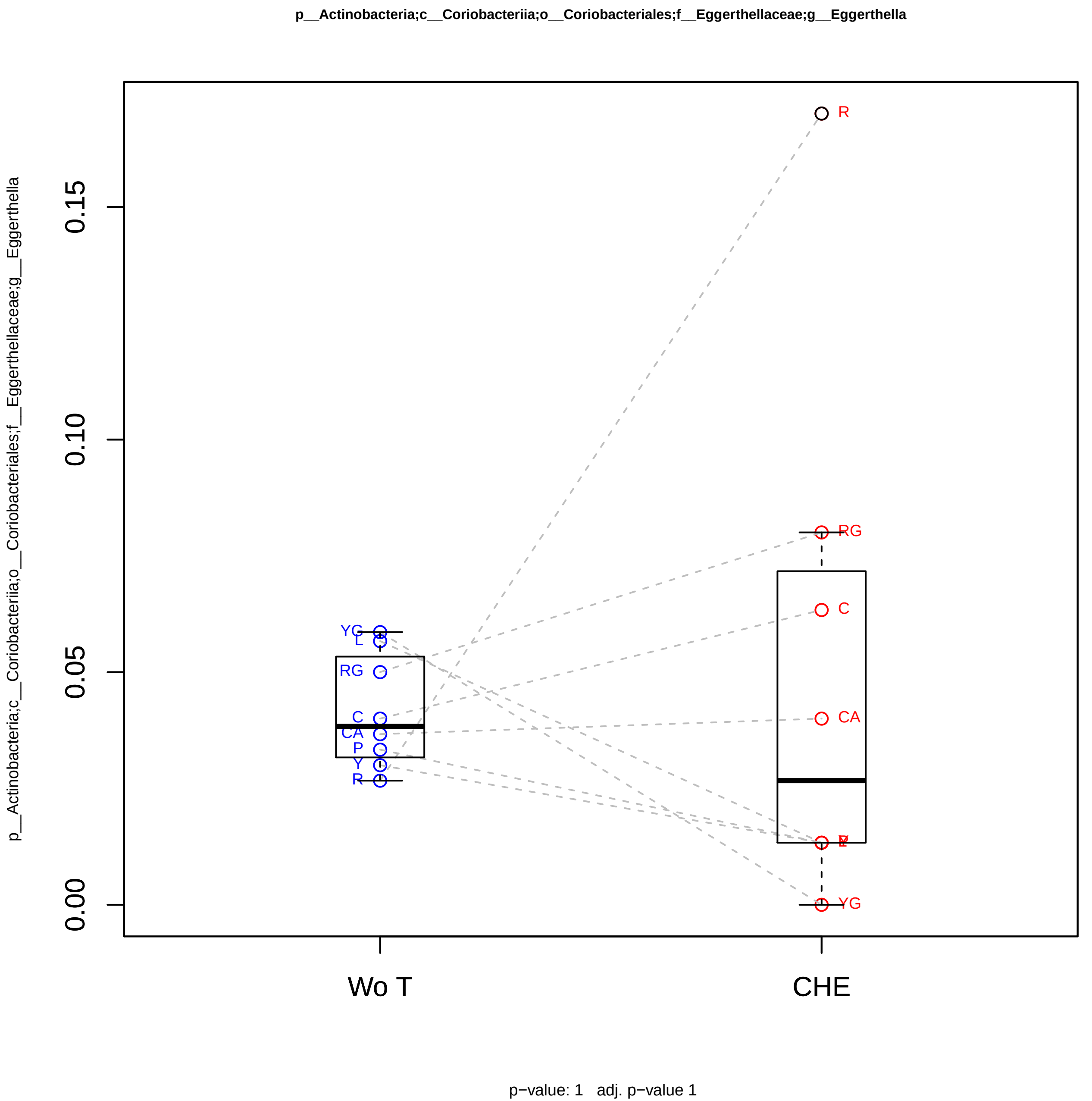
p__Actinobacteria;c__Coriobacteriia;o__Coriobacteriales;f__Eggerthellaceae;g__Eggerthella
p__Actinobacteria;c__Coriobacteriia;o__Coriobacteriales;f__Eggerthellaceae;g__Eggerthella
0.00
0.05
0.10
0.15
Wo T
CHE
YG
L
RG
C
CA
P
Y
R
p−value: 1   adj. p−value 1

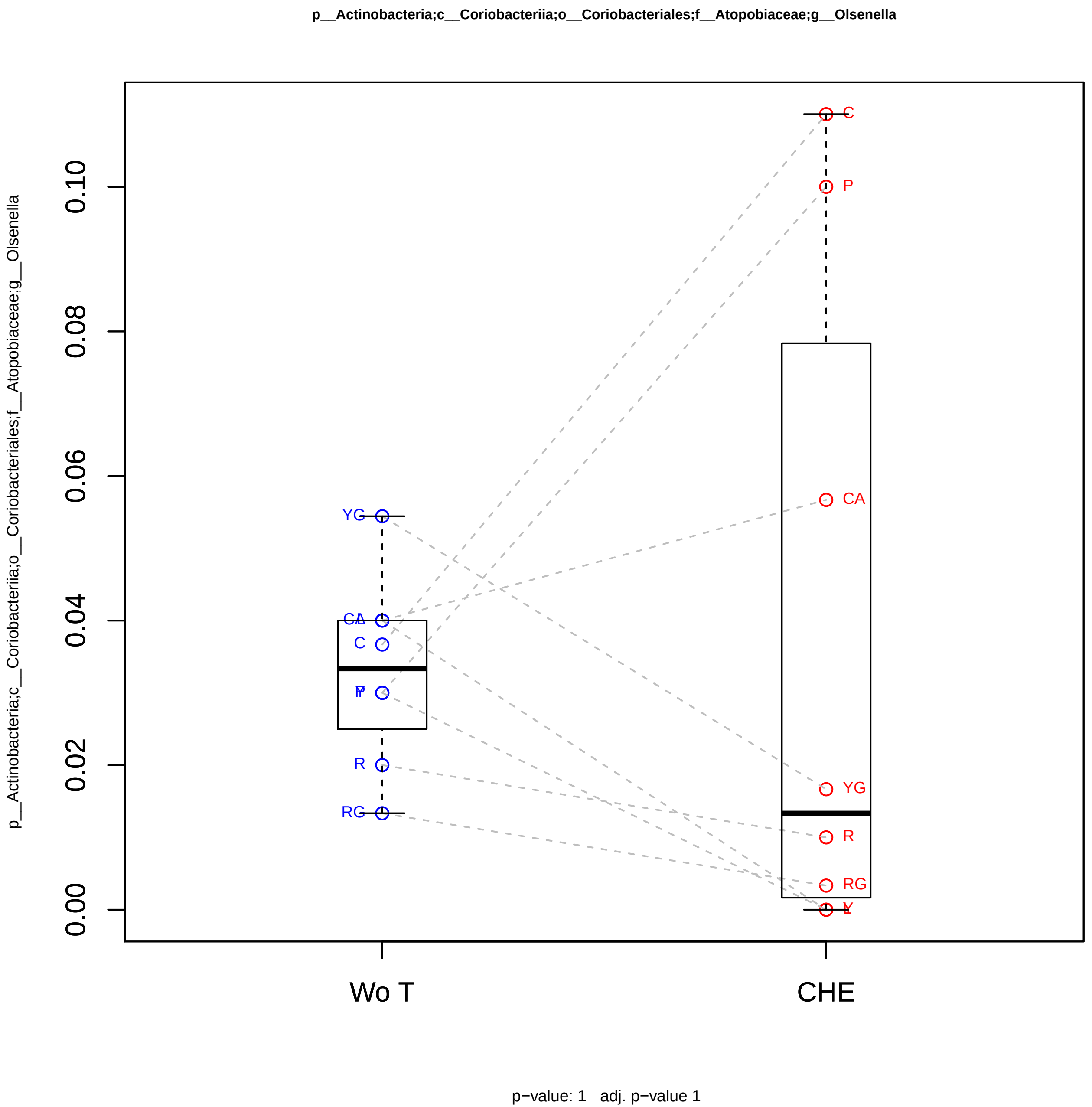
p__Actinobacteria;c__Coriobacteriia;o__Coriobacteriales;f__Atopobiaceae;g__Olsenella
p__Actinobacteria;c__Coriobacteriia;o__Coriobacteriales;f__Atopobiaceae;g__Olsenella
0.00
0.02
0.04
0.06
0.08
0.10
Wo T
CHE
YG
CA
C
P
R
RG
YG
CA
C
P
R
RG
p−value: 1 adj. p−value 1

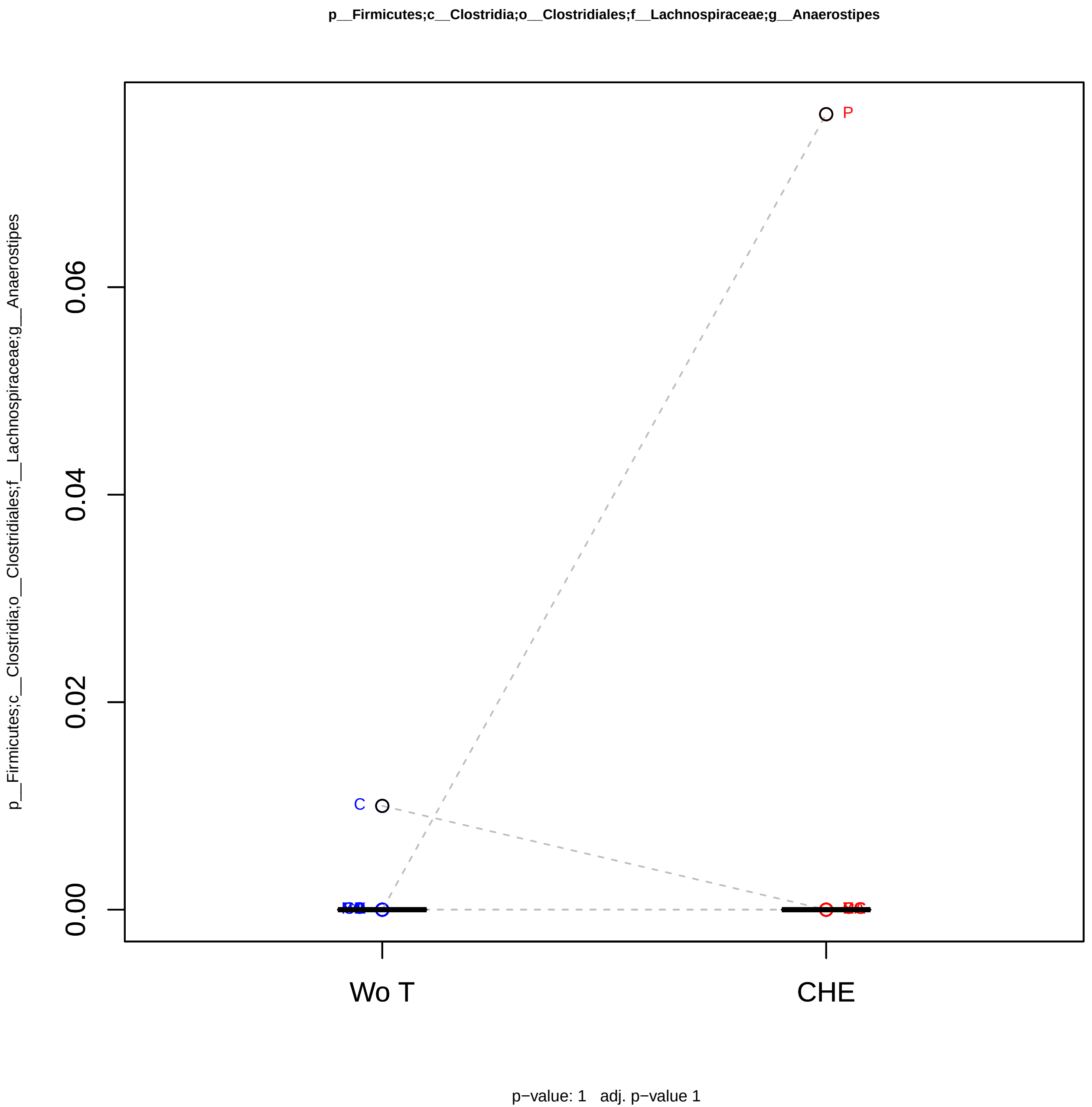
p__Firmicutes;c__Clostridia;o__Clostridiales;f__Lachnospiraceae;g__Anaerostipes
p__Firmicutes;c__Clostridia;o__Clostridiales;f__Lachnospiraceae;g__Anaerostipes
0.00
0.02
0.04
0.06
P
C
Wo T
CHE
p−value: 1 adj. p−value 1

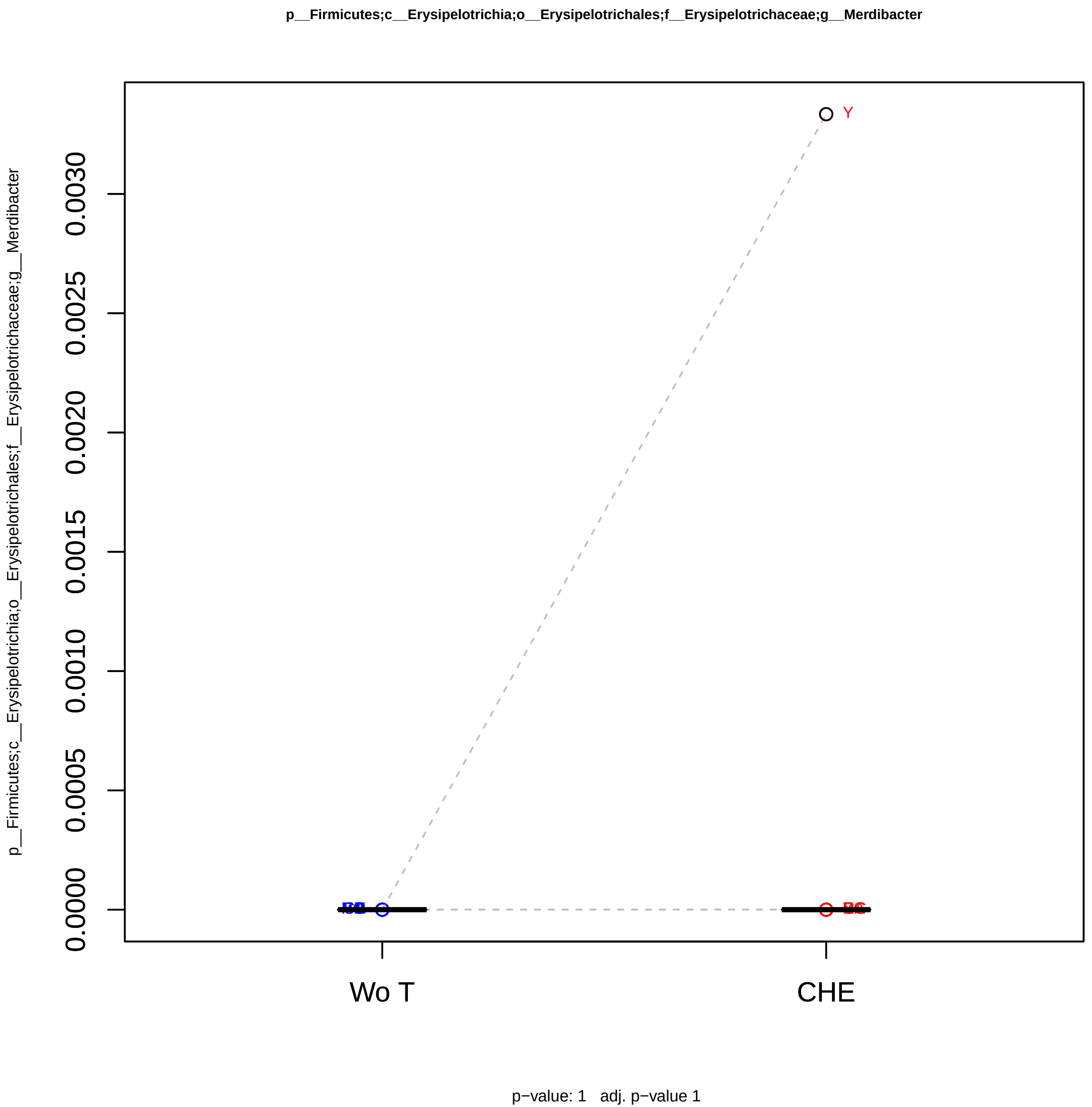
p__Firmicutes;c__Erysipelotrichia;o__Erysipelotrichales;f__Erysipelotrichaceae;g__Merdibacter
p__Firmicutes;c__Erysipelotrichia;o__Erysipelotrichales;f__Erysipelotrichaceae;g__Merdibacter
0.0000
0.0005
0.0010
0.0015
0.0020
0.0025
0.0030
Y
Wo T
CHE
p−value: 1   adj. p−value 1

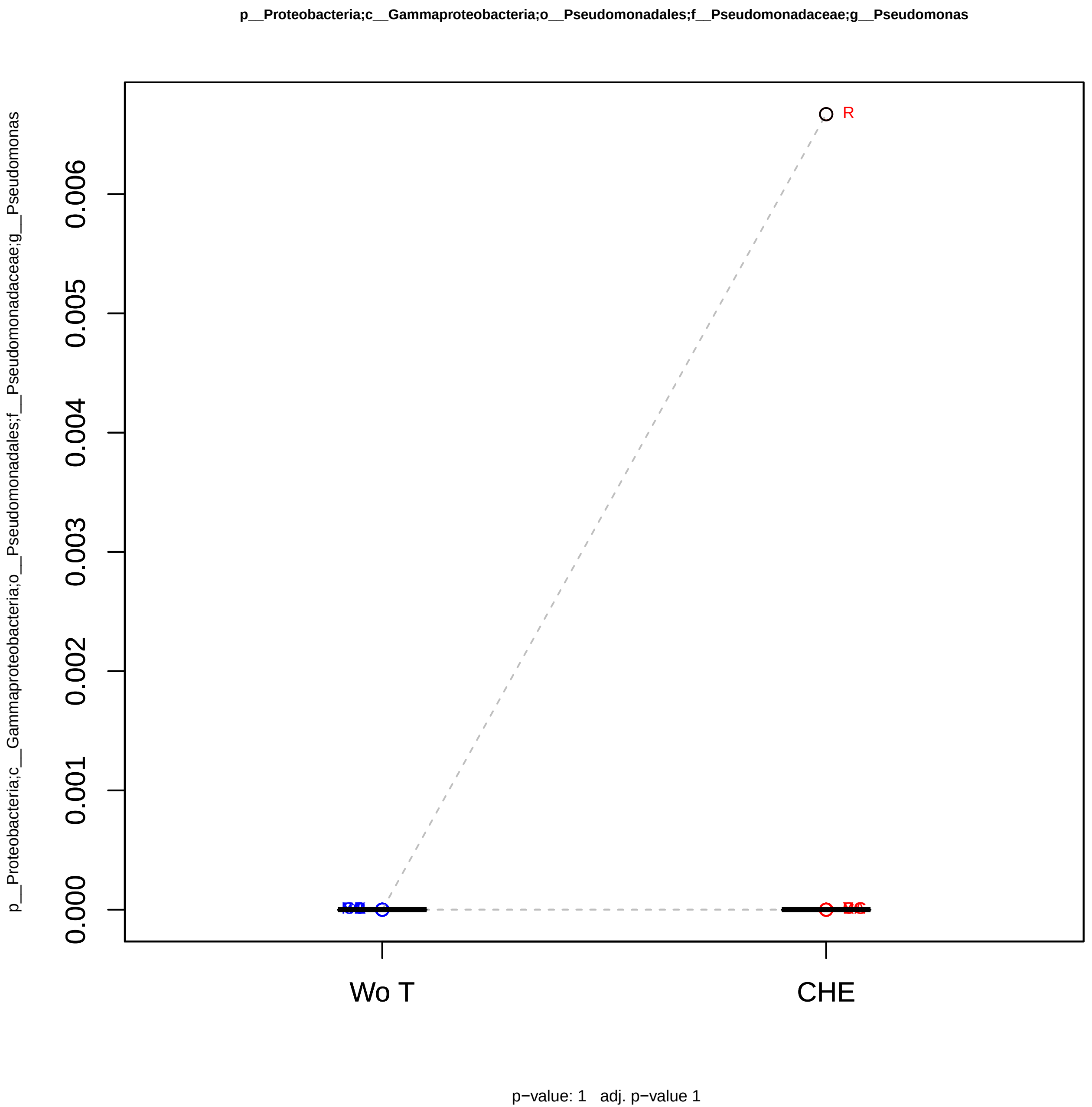
p__Proteobacteria;c__Gammaproteobacteria;o__Pseudomonadales;f__Pseudomonadaceae;g__Pseudomonas
p__Proteobacteria;c__Gammaproteobacteria;o__Pseudomonadales;f__Pseudomonadaceae;g__Pseudomonas
0.000
0.001
0.002
0.003
0.004
0.005
0.006
R
Wo T
CHE
p−value: 1   adj. p−value 1

p__Proteobacteria;c__Deltaproteobacteria;o__Desulfovibrionales;f__Desulfovibrionaceae;g__NA

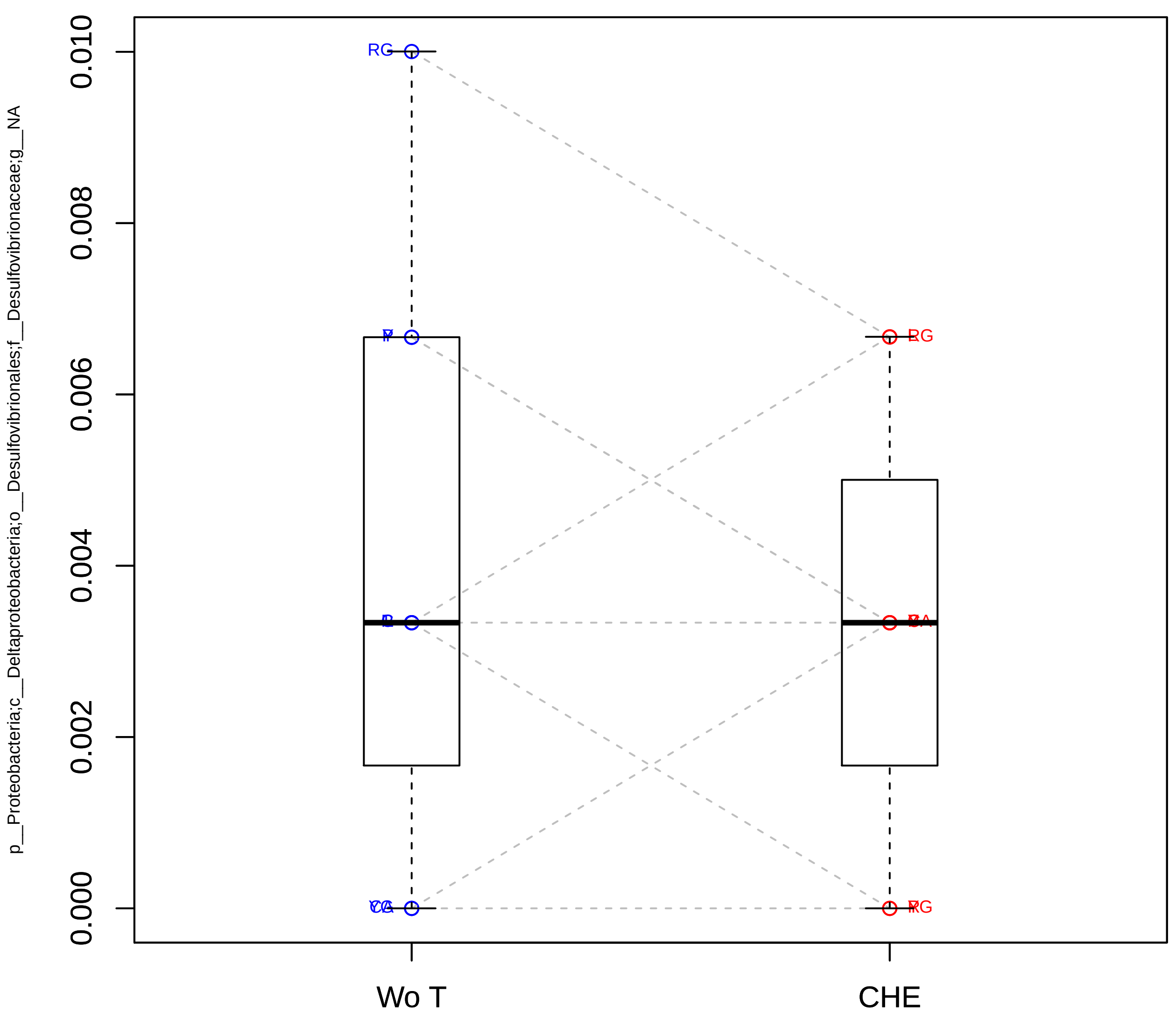


p−value: 1 adj. p−value 1

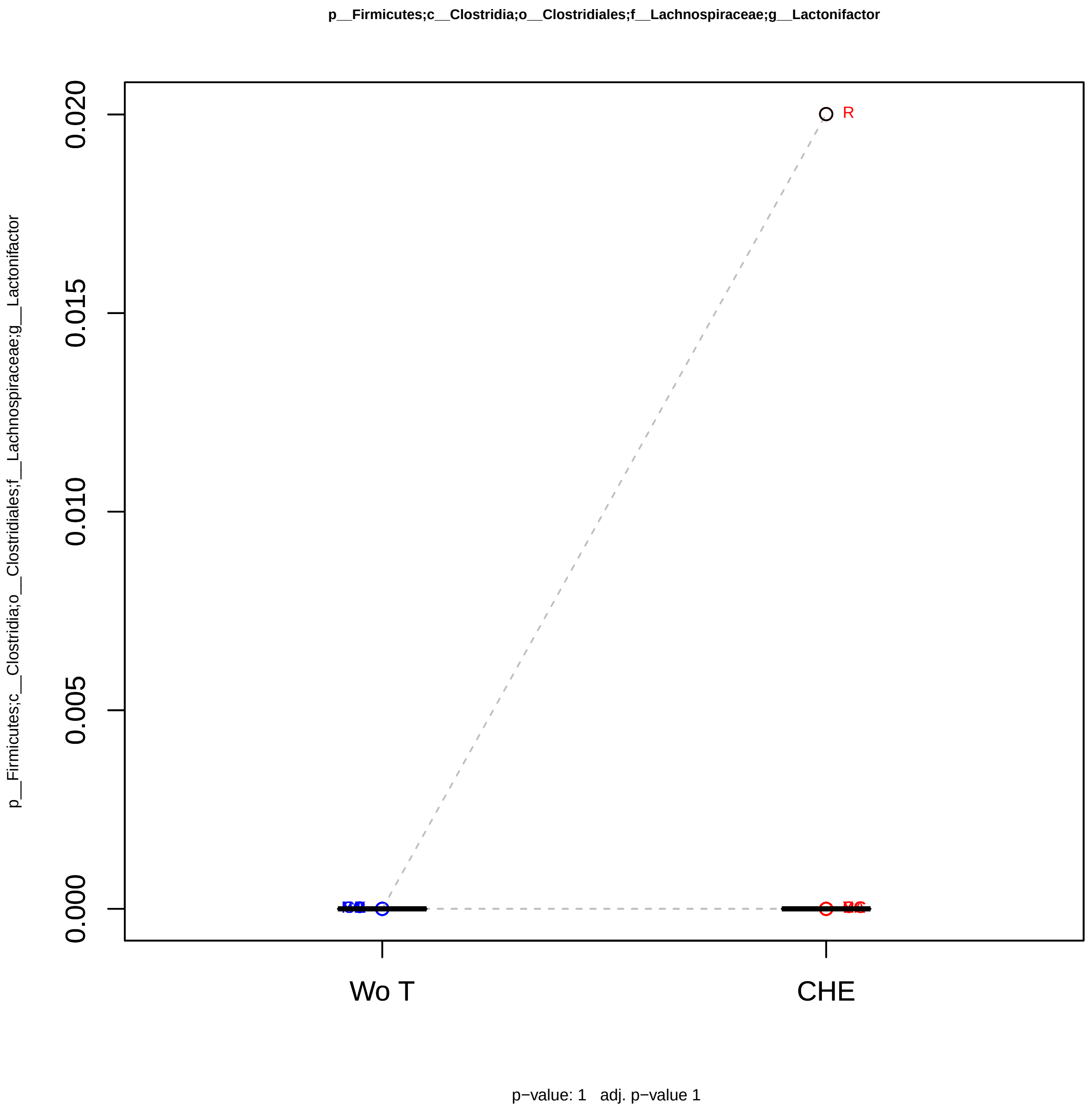
p__Firmicutes;c__Clostridia;o__Clostridiales;f__Lachnospiraceae;g__Lactonifactor
p__Firmicutes;c__Clostridia;o__Clostridiales;f__Lachnospiraceae;g__Lactonifactor
0.000
0.005
0.010
0.015
0.020
R
Wo T
CHE
p−value: 1   adj. p−value 1

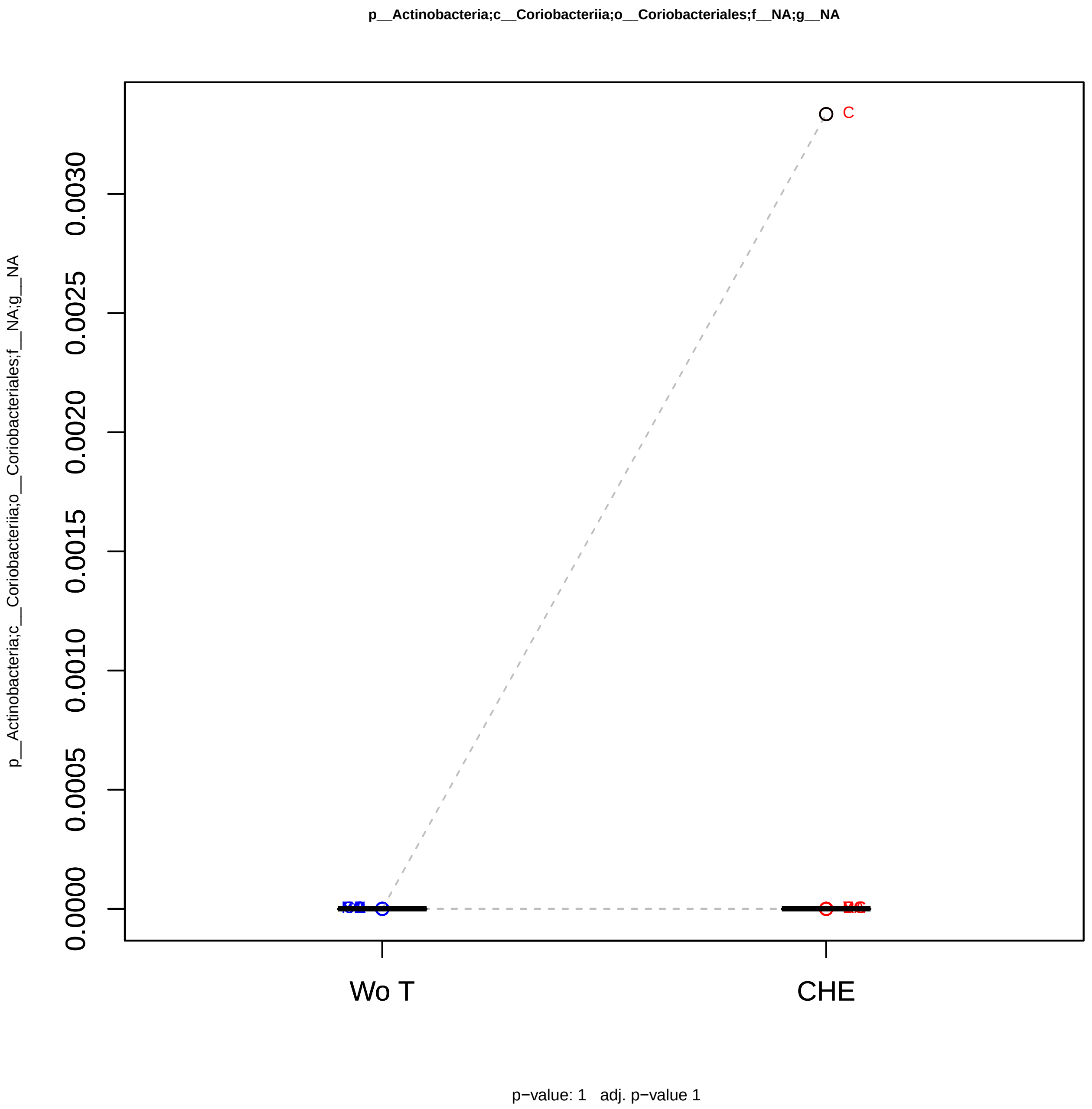
p__Actinobacteria;c__Coriobacteriia;o__Coriobacteriales;f__NA;g__NA
p__Actinobacteria;c__Coriobacteriia;o__Coriobacteriales;f__NA;g__NA
0.0000
0.0005
0.0010
0.0015
0.0020
0.0025
0.0030
C
Wo T
CHE
p−value: 1 adj. p−value 1

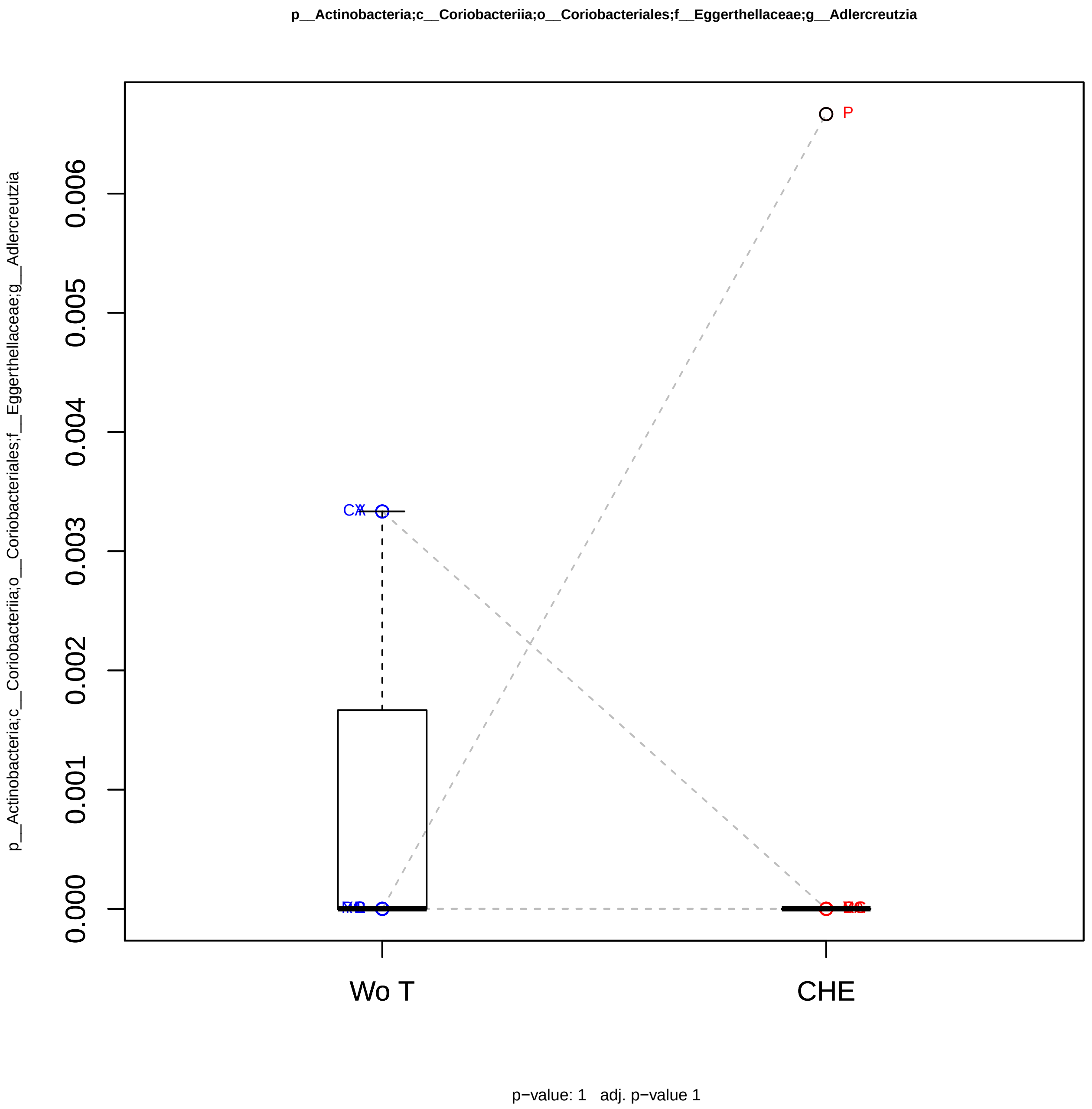
p__Actinobacteria;c__Coriobacteriia;o__Coriobacteriales;f__Eggerthellaceae;g__Adlercreutzia
p__Actinobacteria;c__Coriobacteriia;o__Coriobacteriales;f__Eggerthellaceae;g__Adlercreutzia
0.000
0.001
0.002
0.003
0.004
0.005
0.006
P
Wo T
CHE
p−value: 1 adj. p−value 1

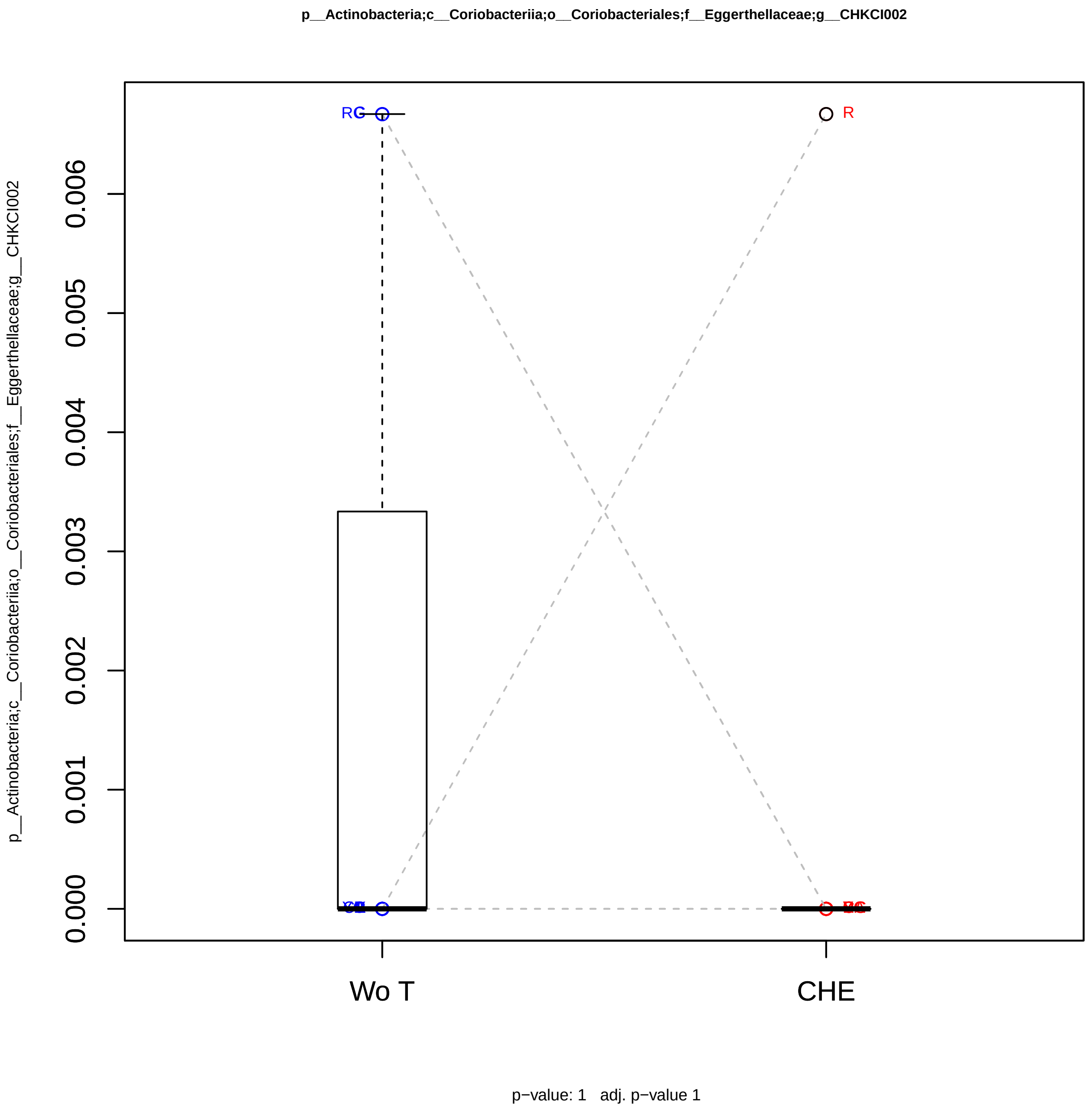

p__Actinobacteria;c__Coriobacteriia;o__Coriobacteriales;f__Eggerthellaceae;g__CHKCI002
p__Actinobacteria;c__Coriobacteriia;o__Coriobacteriales;f__Eggerthellaceae;g__CHKCI002
0.000
0.001
0.002
0.003
0.004
0.005
0.006
Wo T
CHE
R
p−value: 1 adj. p−value 1

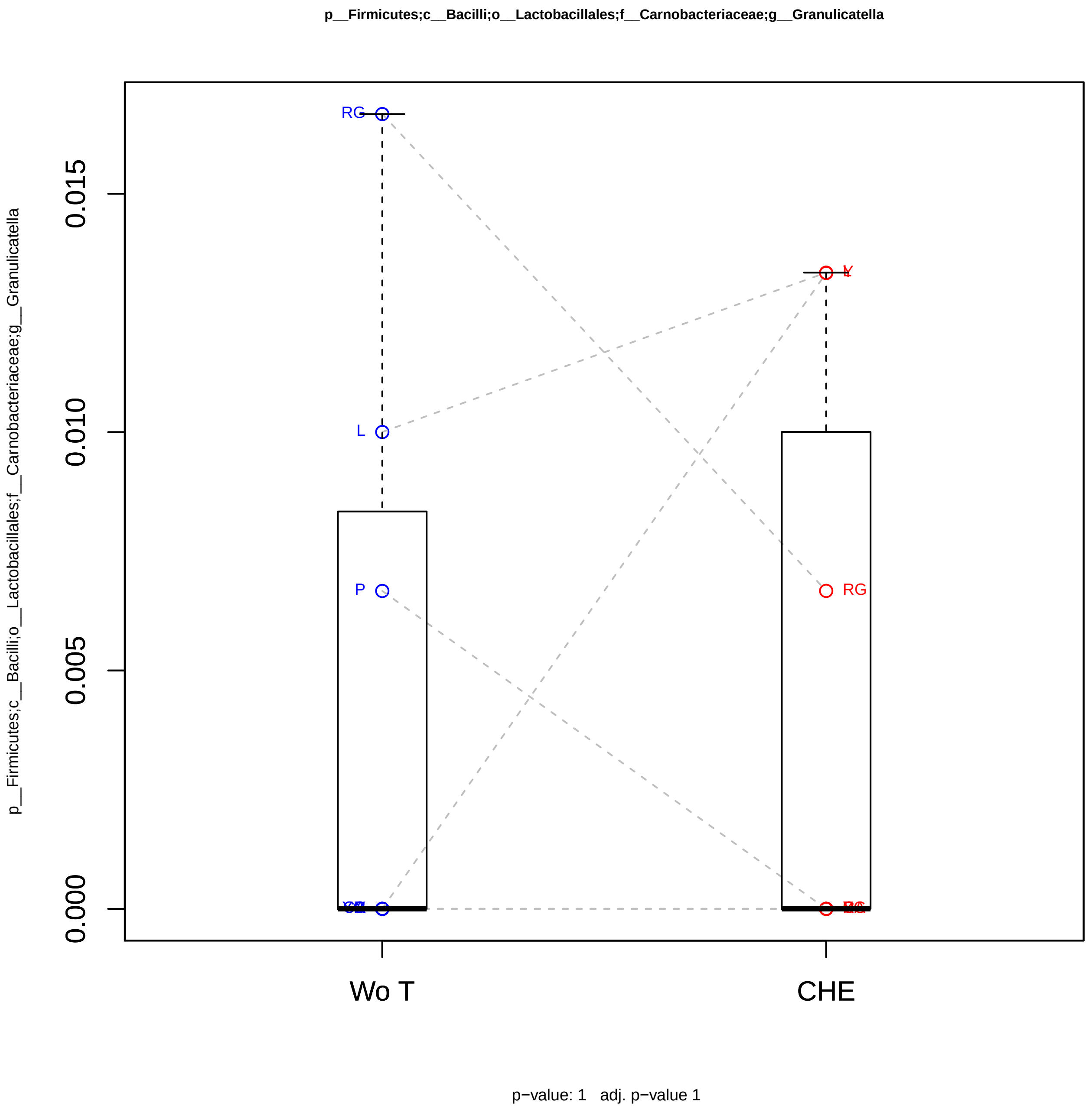
p__Firmicutes;c__Bacilli;o__Lactobacillales;f__Carnobacteriaceae;g__Granulicatella
p__Firmicutes;c__Bacilli;o__Lactobacillales;f__Carnobacteriaceae;g__Granulicatella
0.000
0.005
0.010
0.015
RG
L
P
RG
Wo T
CHE
p−value: 1 adj. p−value 1

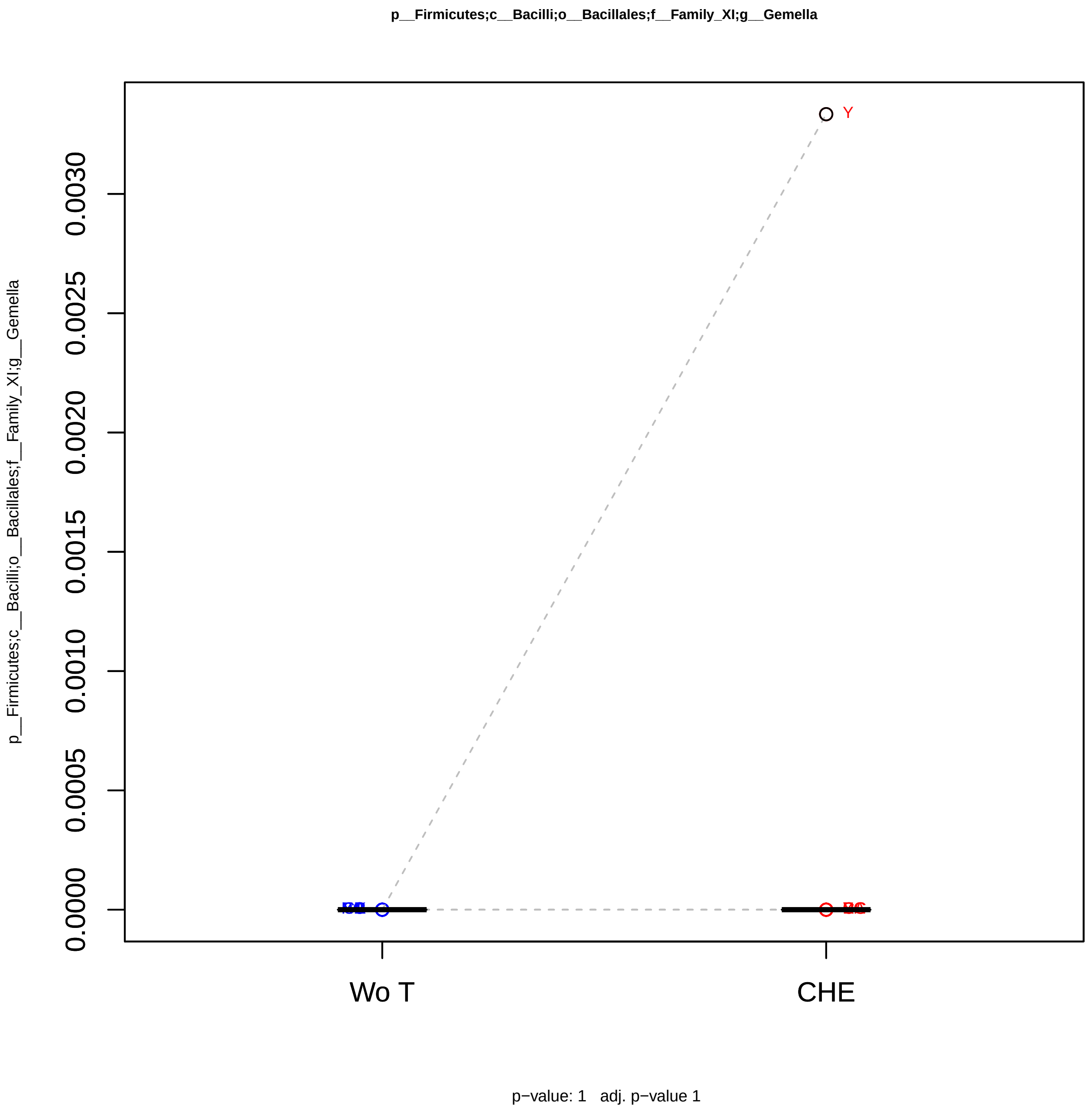
p__Firmicutes;c__Bacilli;o__Bacillales;f__Family_XI;g__Gemella
p__Firmicutes;c__Bacilli;o__Bacillales;f__Family_XI;g__Gemella
0.0000
0.0005
0.0010
0.0015
0.0020
0.0025
0.0030
Y
Wo T
CHE
p−value: 1 adj. p−value 1

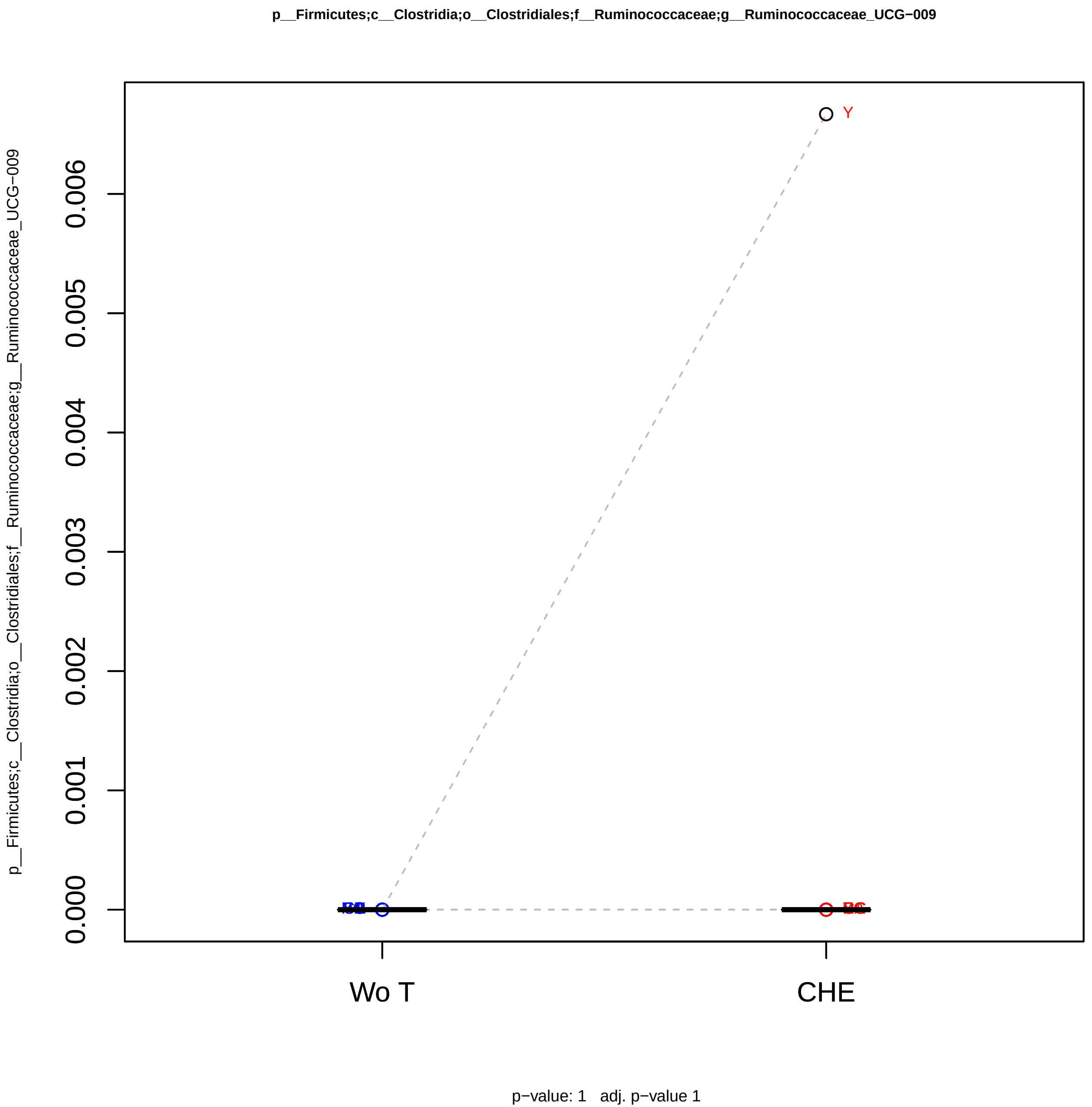
p__Firmicutes;c__Clostridia;o__Clostridiales;f__Ruminococcaceae;g__Ruminococcaceae_UCG−009
p__Firmicutes;c__Clostridia;o__Clostridiales;f__Ruminococcaceae;g__Ruminococcaceae_UCG−009
0.000
0.001
0.002
0.003
0.004
0.005
0.006
Y
Wo T
CHE
p−value: 1   adj. p−value 1

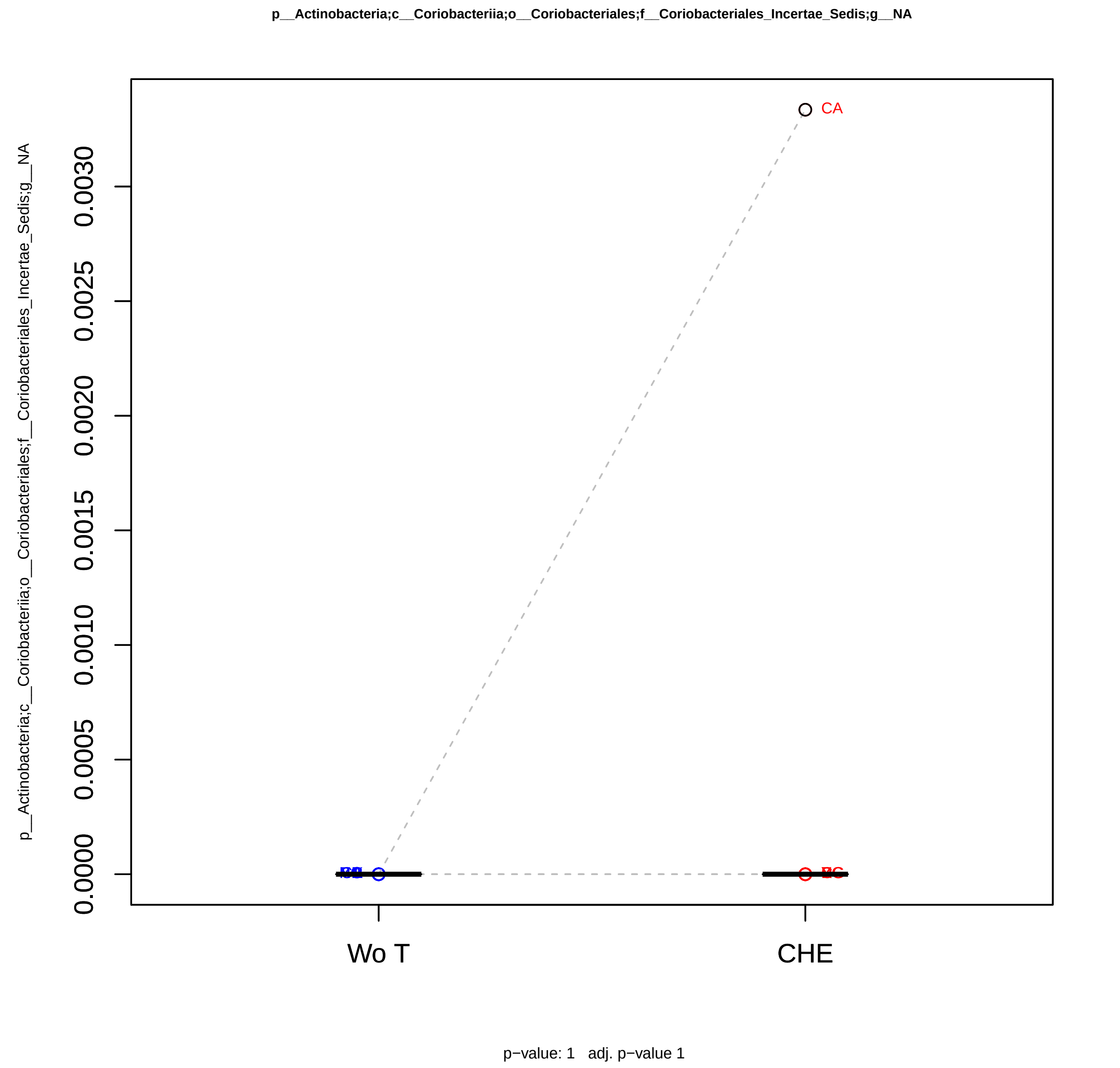

p__Actinobacteria;c__Coriobacteriia;o__Coriobacteriales;f__Coriobacteriales_Incertae_Sedis;g__NA
p__Actinobacteria;c__Coriobacteriia;o__Coriobacteriales;f__Coriobacteriales_Incertae_Sedis;g__NA
0.0000
0.0005
0.0010
0.0015
0.0020
0.0025
0.0030
CA
Wo T
CHE
p−value: 1   adj. p−value 1

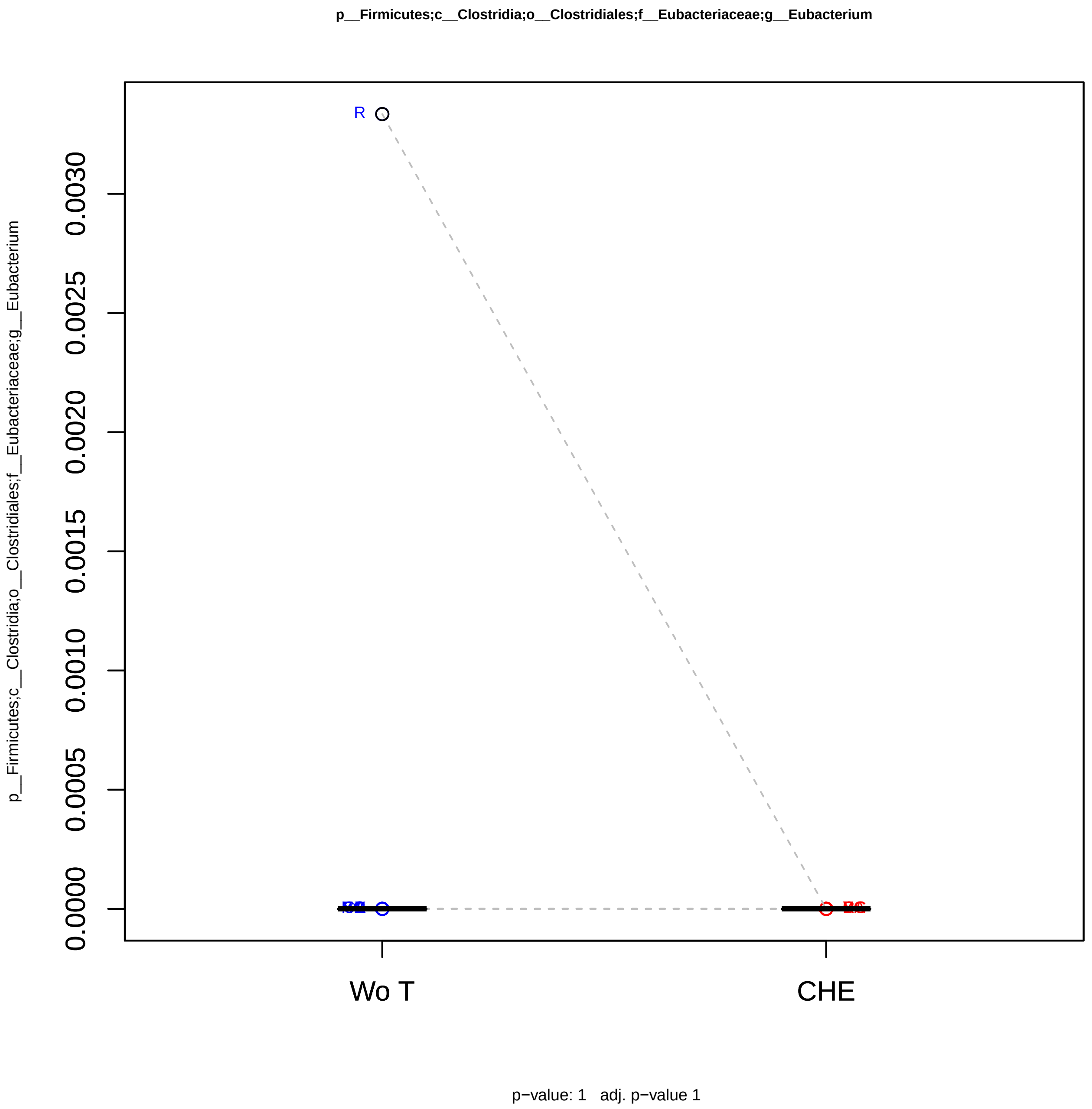

p__Firmicutes;c__Clostridia;o__Clostridiales;f__Eubacteriaceae;g__Eubacterium
p__Firmicutes;c__Clostridia;o__Clostridiales;f__Eubacteriaceae;g__Eubacterium
0.0000
0.0005
0.0010
0.0015
0.0020
0.0025
0.0030
R
Wo T
CHE
p−value: 1 adj. p−value 1

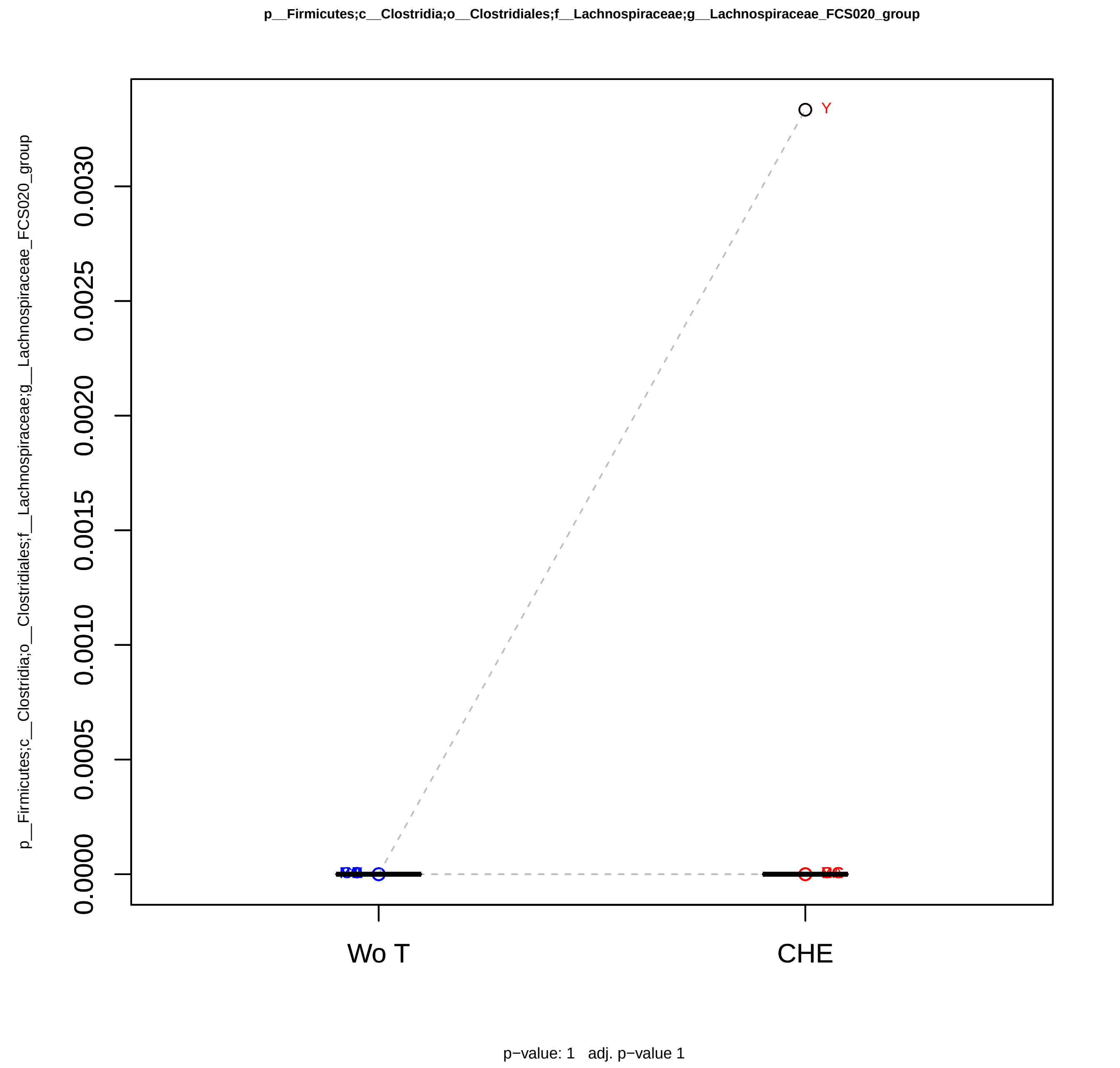
p__Firmicutes;c__Clostridia;o__Clostridiales;f__Lachnospiraceae;g__Lachnospiraceae_FCS020_group
p__Firmicutes;c__Clostridia;o__Clostridiales;f__Lachnospiraceae;g__Lachnospiraceae_FCS020_group
0.0000
0.0005
0.0010
0.0015
0.0020
0.0025
0.0030
Y
Wo T
CHE
p−value: 1   adj. p−value 1

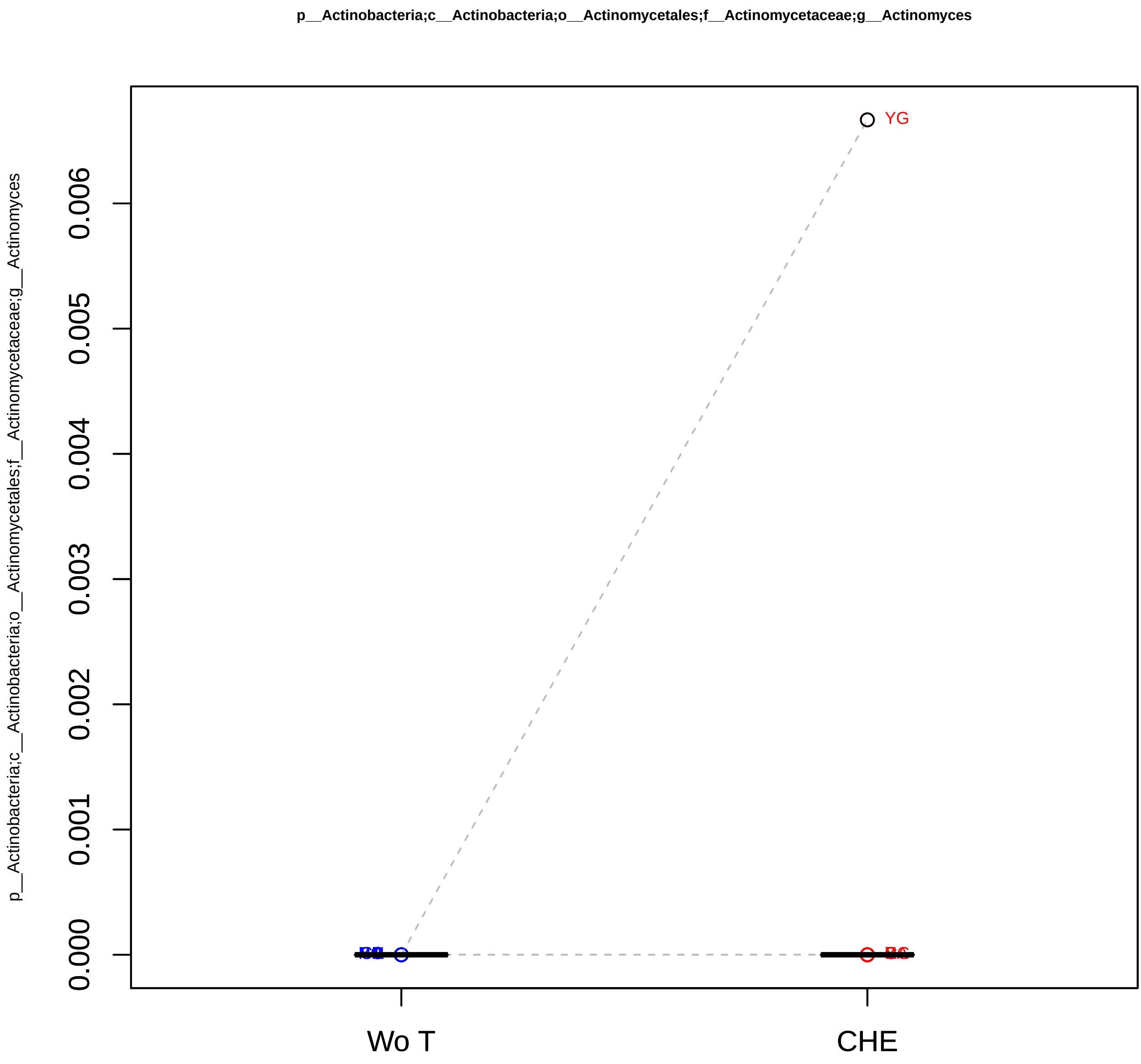

p−value: 1   adj. p−value 1

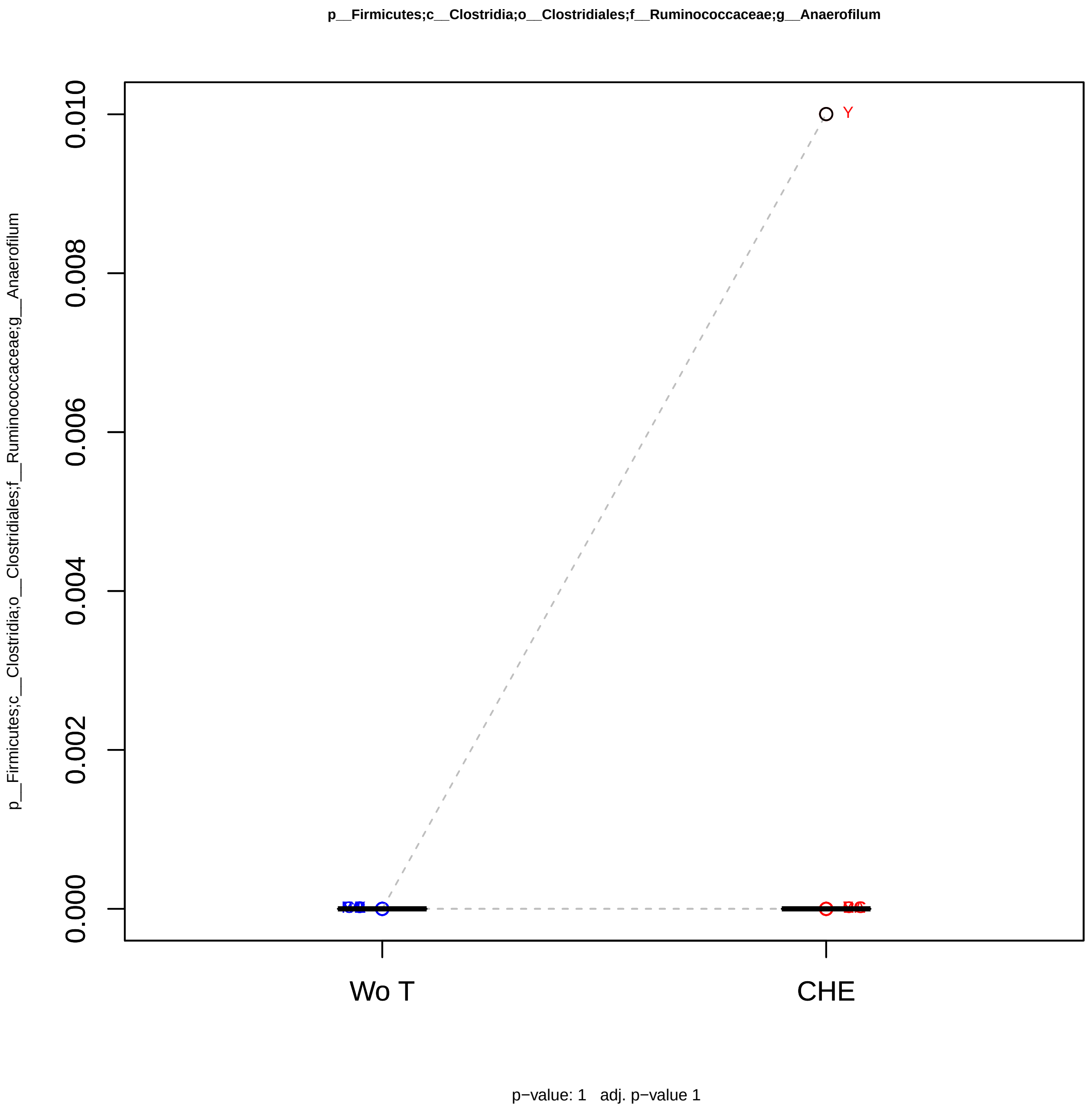

p__Firmicutes;c__Clostridia;o__Clostridiales;f__Ruminococcaceae;g__Anaerofilum
p__Firmicutes;c__Clostridia;o__Clostridiales;f__Ruminococcaceae;g__Anaerofilum
0.000
0.002
0.004
0.006
0.008
0.010
Y
Wo T
CHE
p−value: 1 adj. p−value 1
