# Supplementary material for: Enrichment of Food With Tannin Extracts Promotes Healthy Changes in the Human Gut Microbiota
Source: Front Microbiol. 2021 Mar 16;12:625782. doi: 10.3389/fmicb.2021.625782 (PMC8008114; doi:10.3389/fmicb.2021.625782)
Supplement: Supplementary file 4 [file Data_Sheet_4.pdf]

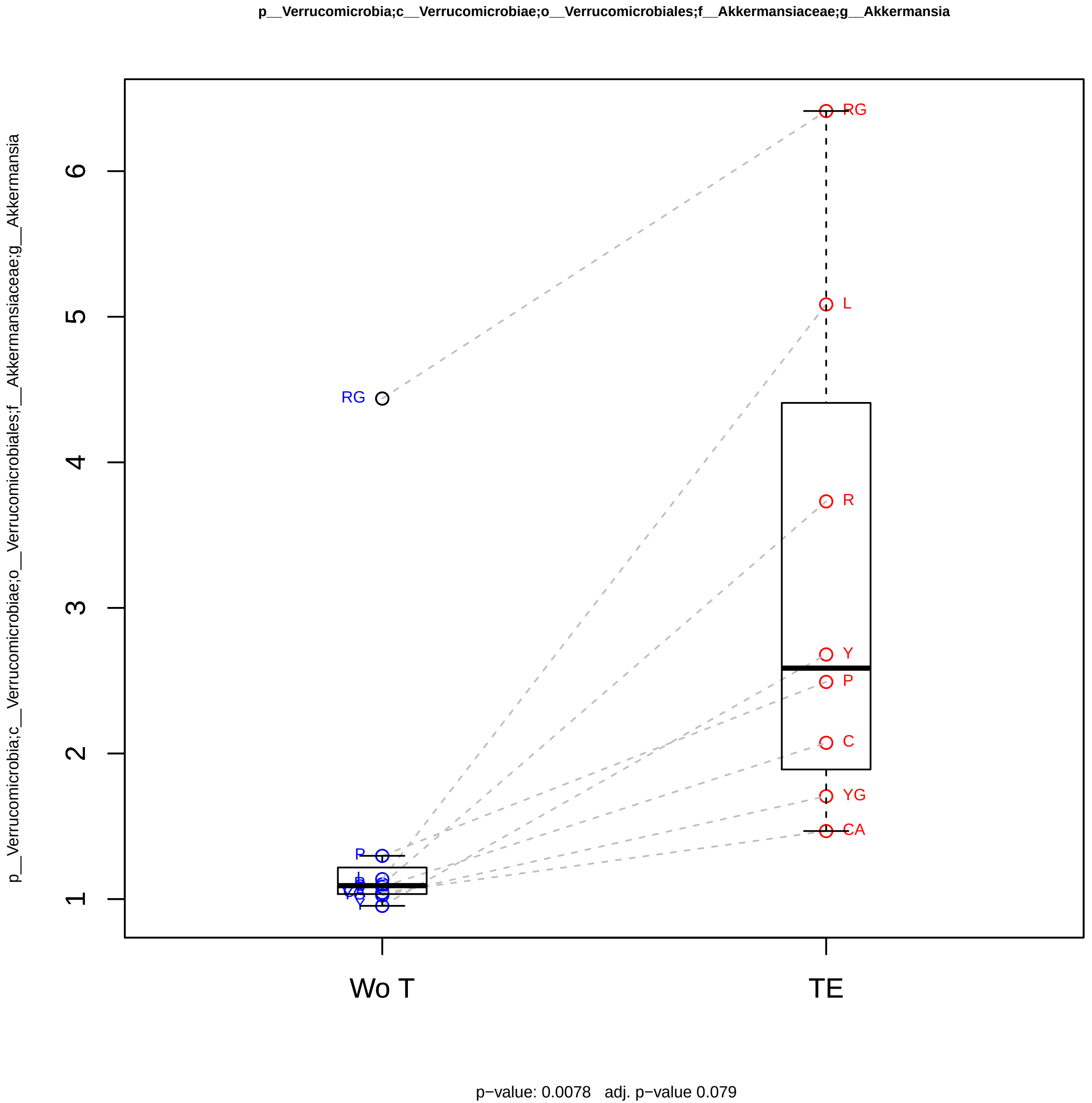
p__Verrucomicrobia;c__Verrucomicrobiae;o__Verrucomicrobiales;f__Akkermansiaceae;g__Akkermansia
p__Verrucomicrobia;c__Verrucomicrobiae;o__Verrucomicrobiales;f__Akkermansiaceae;g__Akkermansia
1
2
3
4
5
6
Wo T
TE
RG
P
RG
L
R
Y
P
C
YG
CA
p−value: 0.0078 adj. p−value 0.079

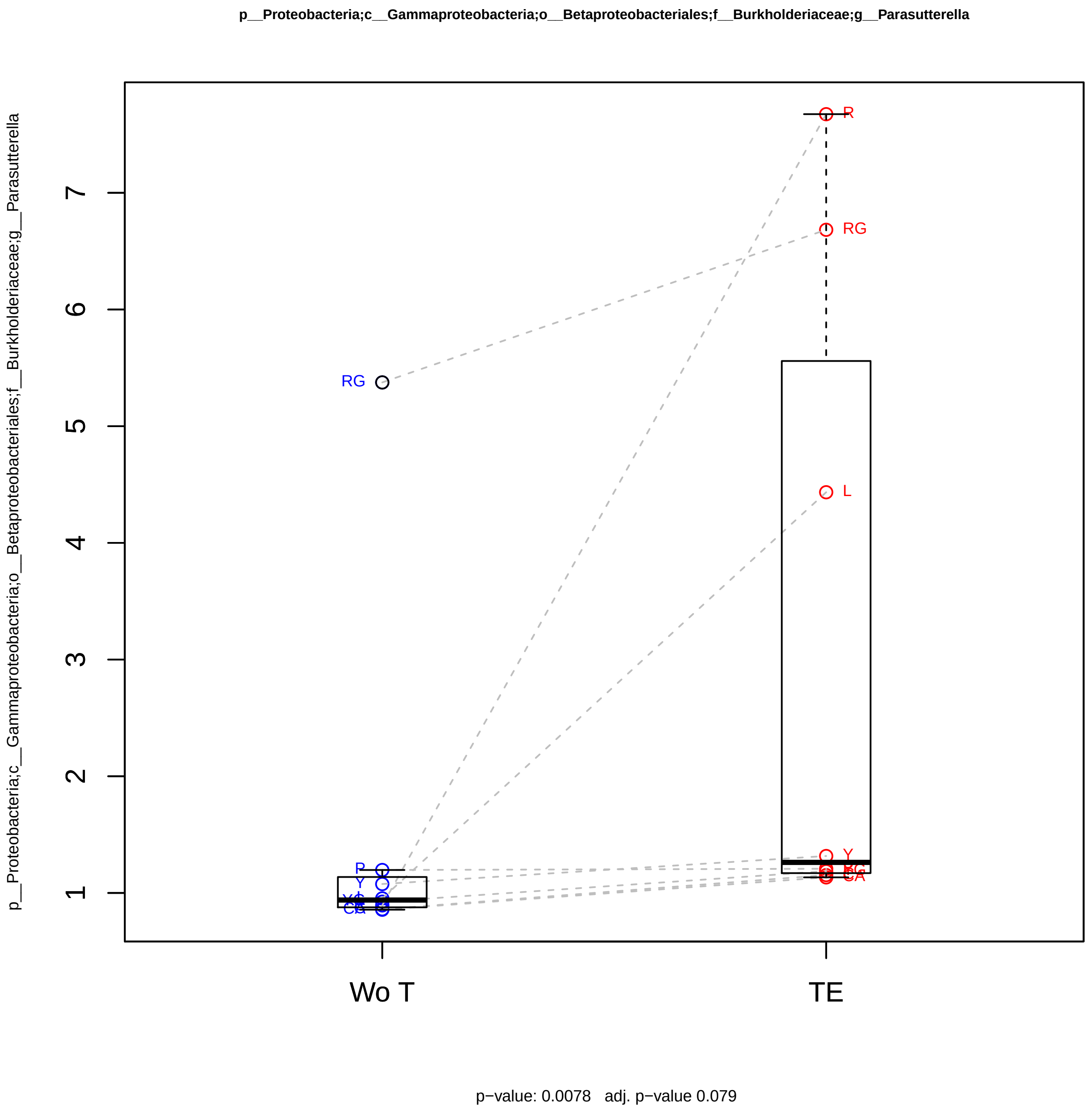
p__Proteobacteria;c__Gammaproteobacteria;o__Betaproteobacteriales;f__Burkholderiaceae;g__Parasutterella
p__Proteobacteria;c__Gammaproteobacteria;o__Betaproteobacteriales;f__Burkholderiaceae;g__Parasutterella
1
2
3
4
5
6
7
Wo T
TE
RG
P
Y
R
RG
L
Y
CA
p−value: 0.0078   adj. p−value 0.079

p__Firmicutes;c__Clostridia;o__Clostridiales;f__Lachnospiraceae;g__NA

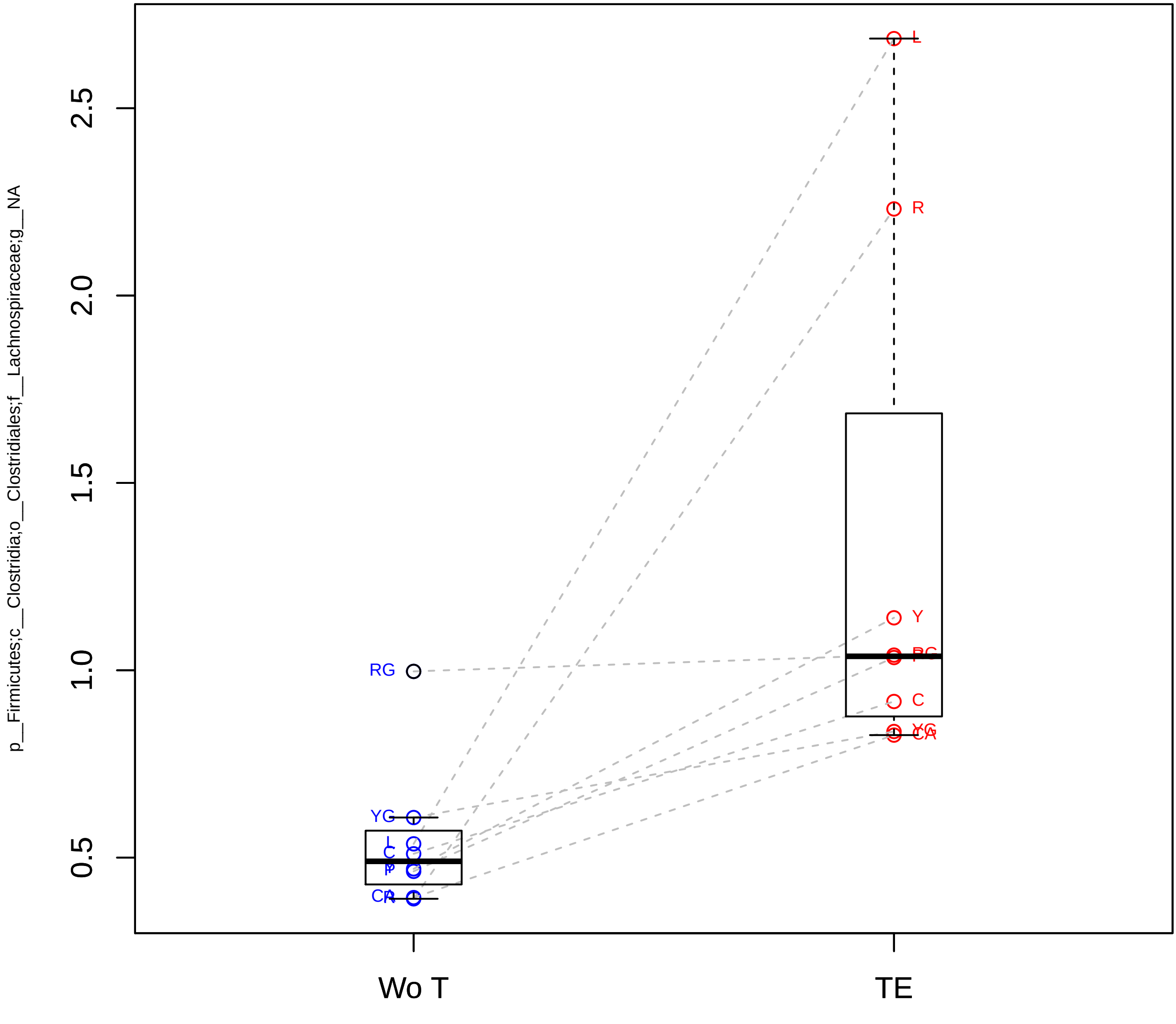

p−value: 0.0078 adj. p−value 0.079

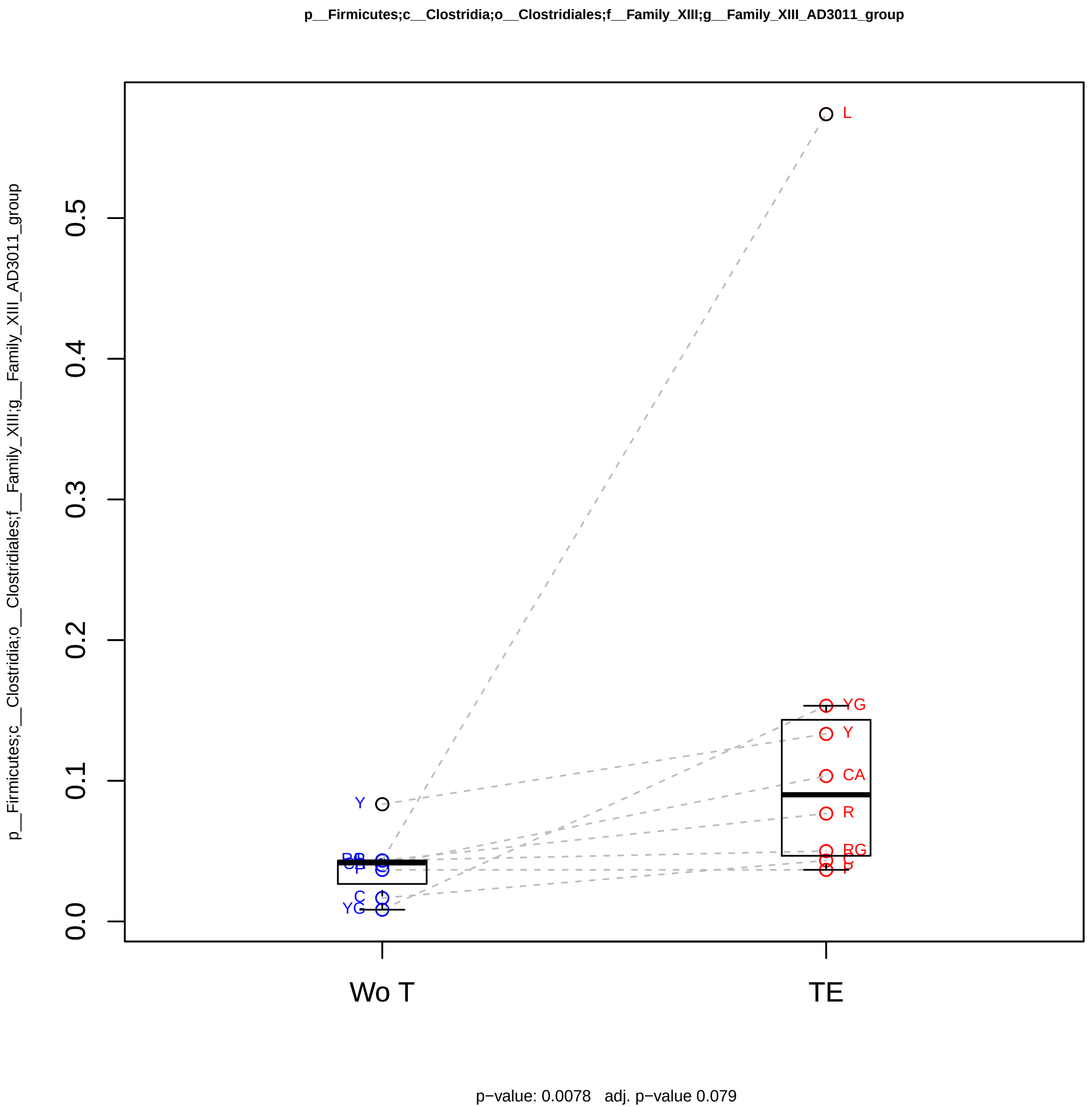
p__Firmicutes;c__Clostridia;o__Clostridiales;f__Family_XIII;g__Family_XIII_AD3011_group
p__Firmicutes;c__Clostridia;o__Clostridiales;f__Family_XIII;g__Family_XIII_AD3011_group
0.0
0.1
0.2
0.3
0.4
0.5
Wo T
TE
Y
C
YG
L
YG
Y
CA
R
RG
p−value: 0.0078   adj. p−value 0.079

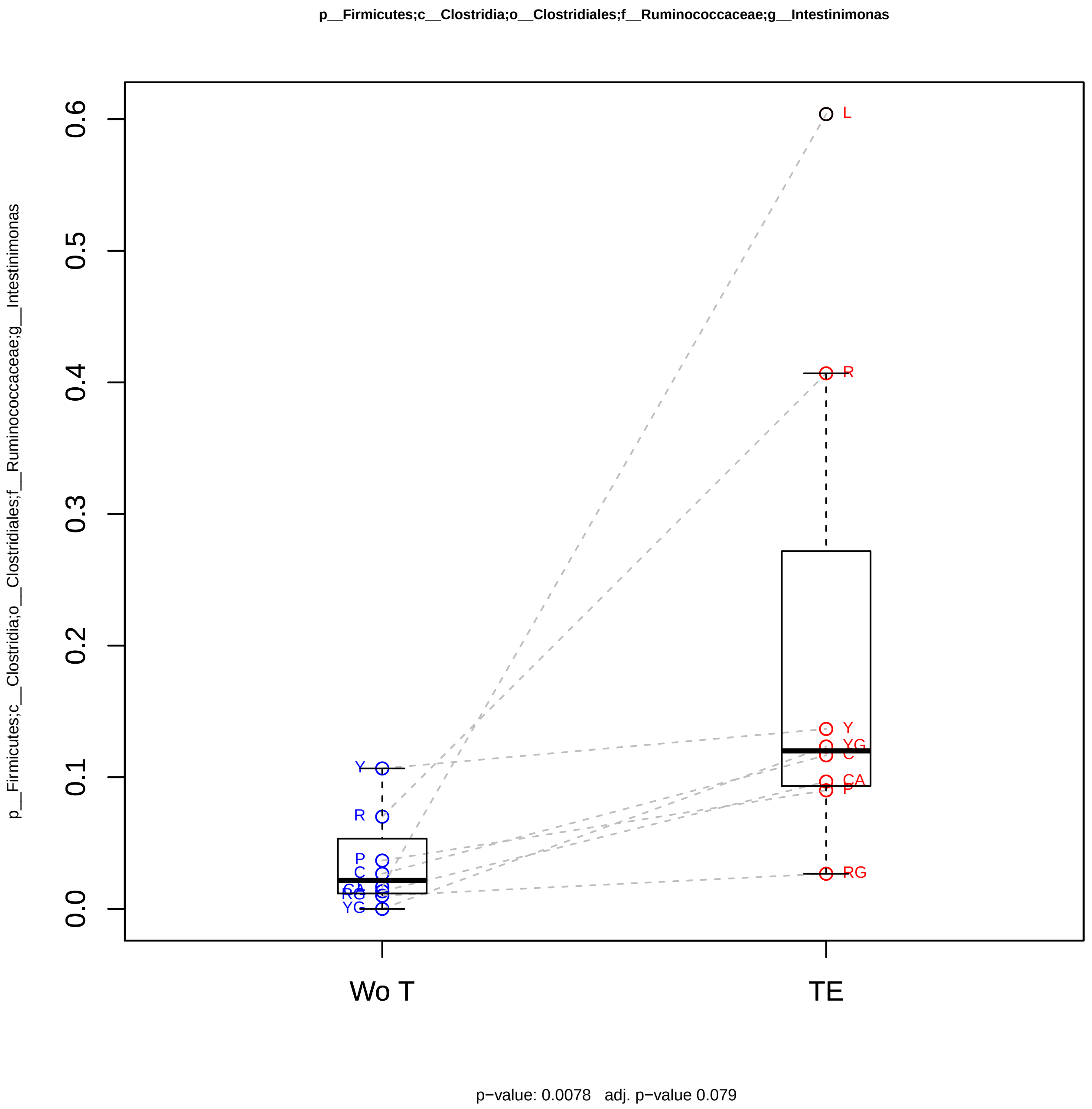

p__Firmicutes;c__Clostridia;o__Clostridiales;f__Ruminococcaceae;g__Intestinimonas
p__Firmicutes;c__Clostridia;o__Clostridiales;f__Ruminococcaceae;g__Intestinimonas
0.0
0.1
0.2
0.3
0.4
0.5
0.6
Wo T
TE
Y
R
P
C
CA
RG
YG
L
R
Y
YG
C
CA
P
RG
p−value: 0.0078 adj. p−value 0.079

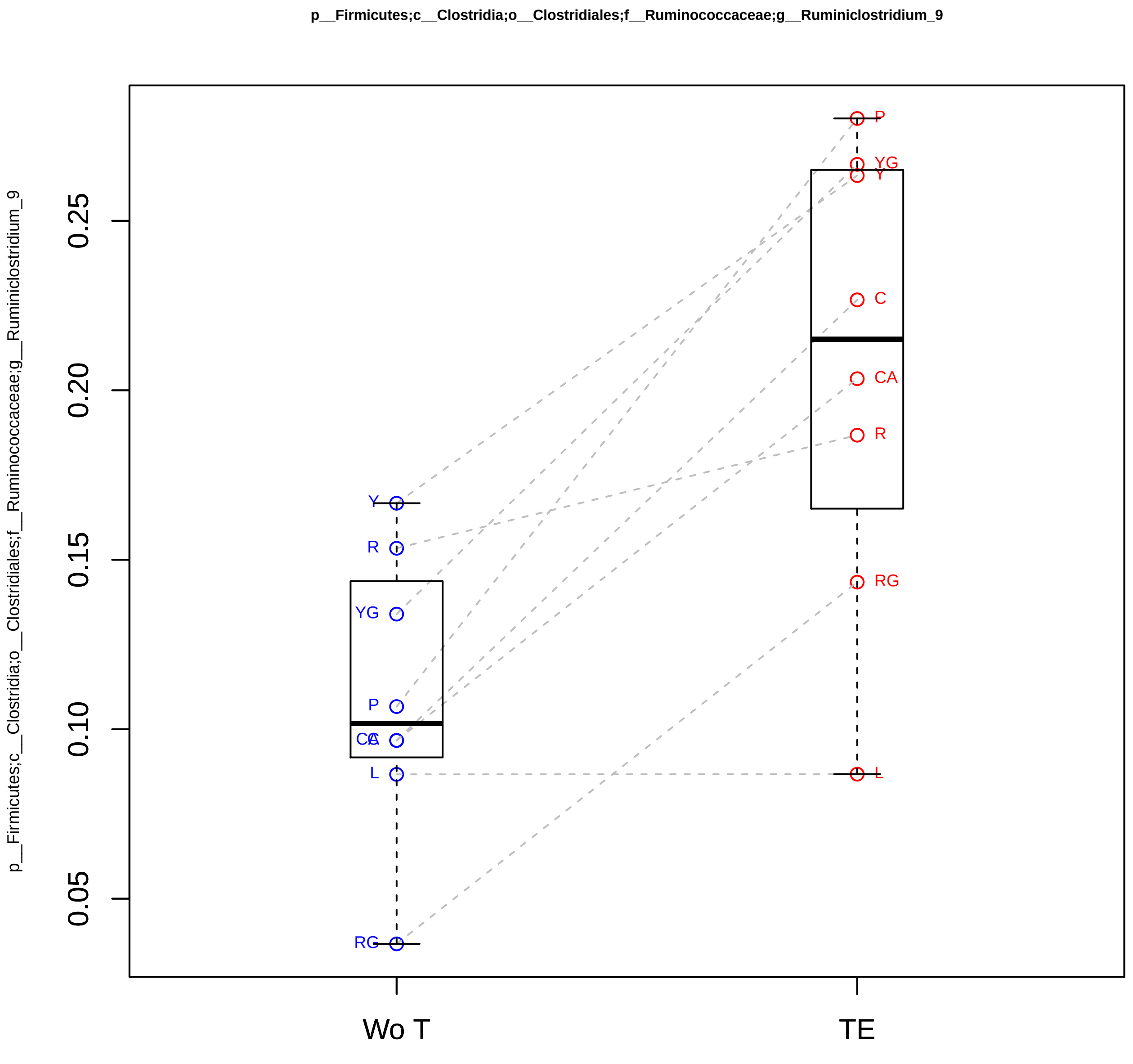

p−value: 0.0078 adj. p−value 0.079

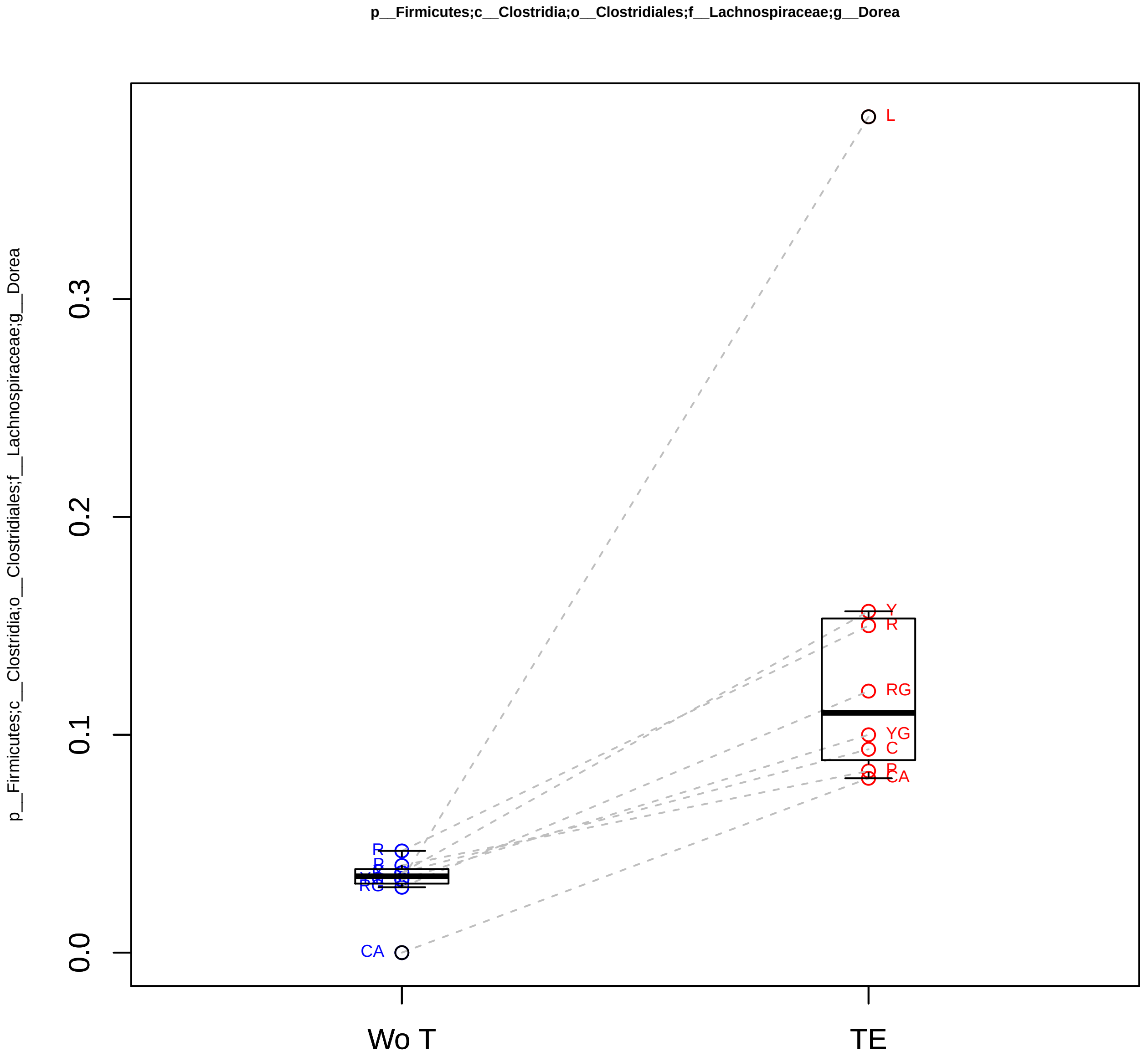

p−value: 0.0078 adj. p−value 0.079

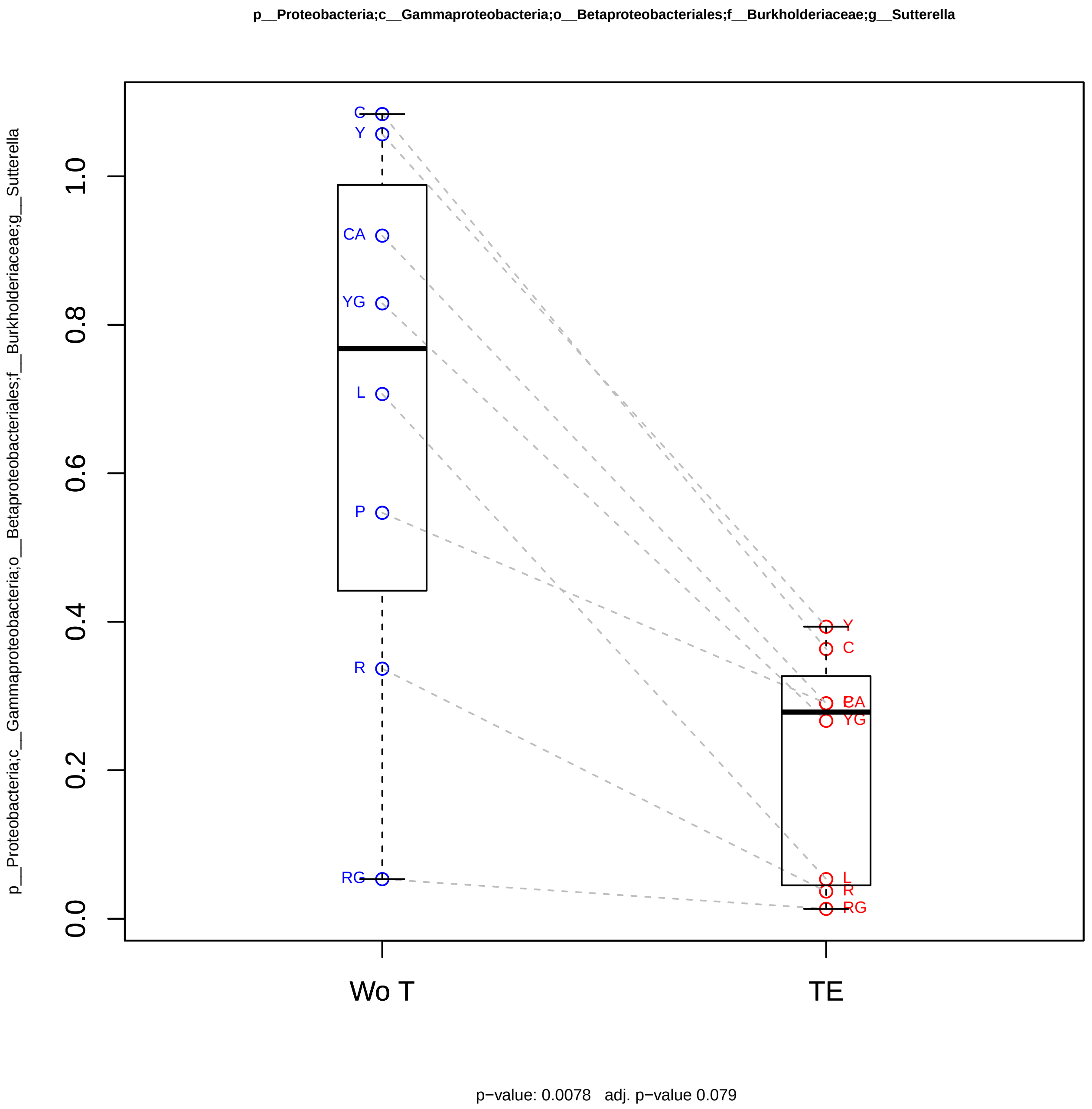
p__Proteobacteria;c__Gammaproteobacteria;o__Betaproteobacteriales;f__Burkholderiaceae;g__Sutterella
p__Proteobacteria;c__Gammaproteobacteria;o__Betaproteobacteriales;f__Burkholderiaceae;g__Sutterella
0.0
0.2
0.4
0.6
0.8
1.0
C
Y
CA
YG
L
P
R
RG
Wo T
TE
p−value: 0.0078 adj. p−value 0.079

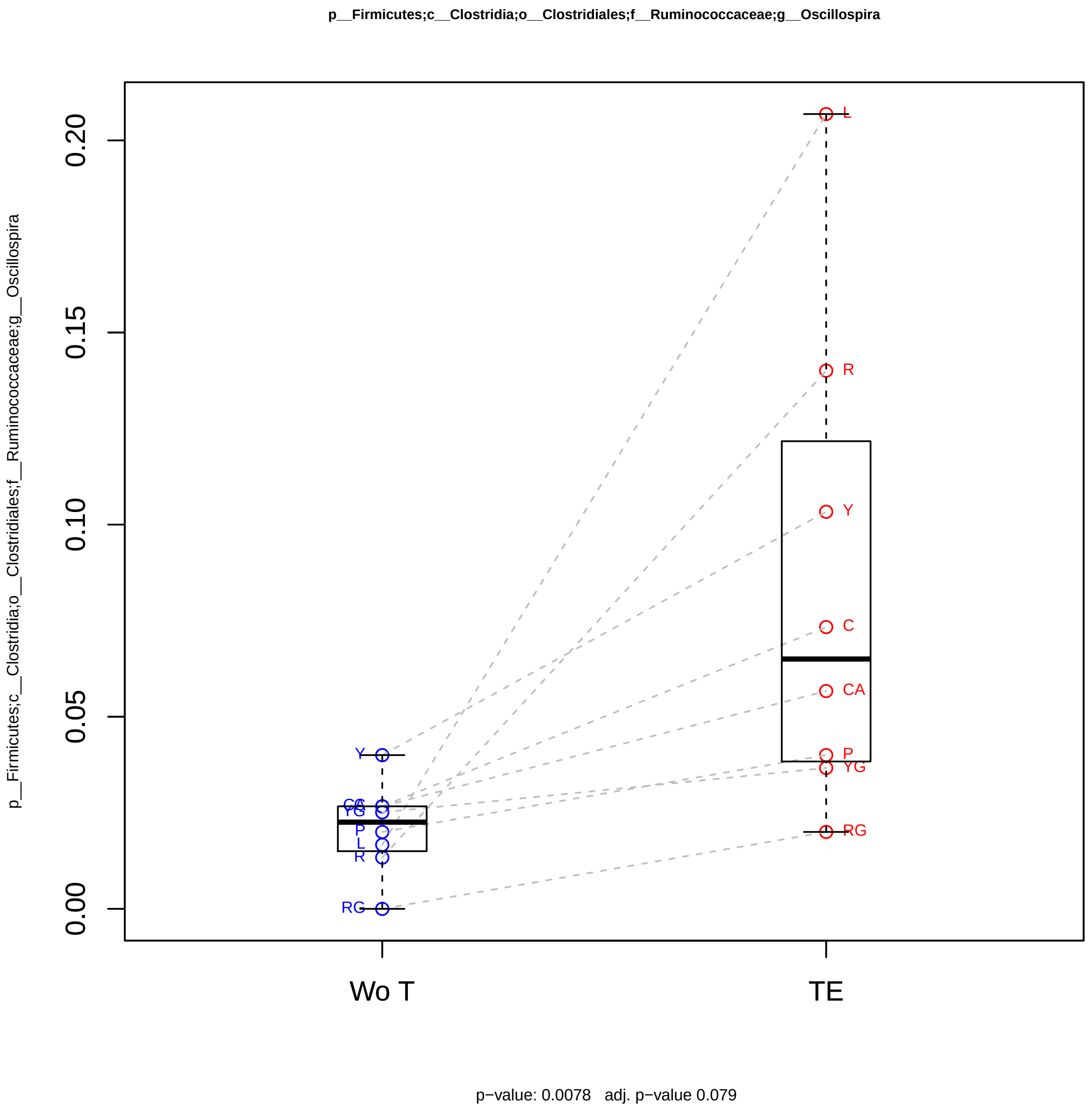
p__Firmicutes;c__Clostridia;o__Clostridiales;f__Ruminococcaceae;g__Oscillospira
p__Firmicutes;c__Clostridia;o__Clostridiales;f__Ruminococcaceae;g__Oscillospira
0.00
0.05
0.10
0.15
0.20
Y
CA
C
YG
P
L
R
RG
L
R
Y
C
CA
P
YG
RG
Wo T
TE
p−value: 0.0078 adj. p−value 0.079

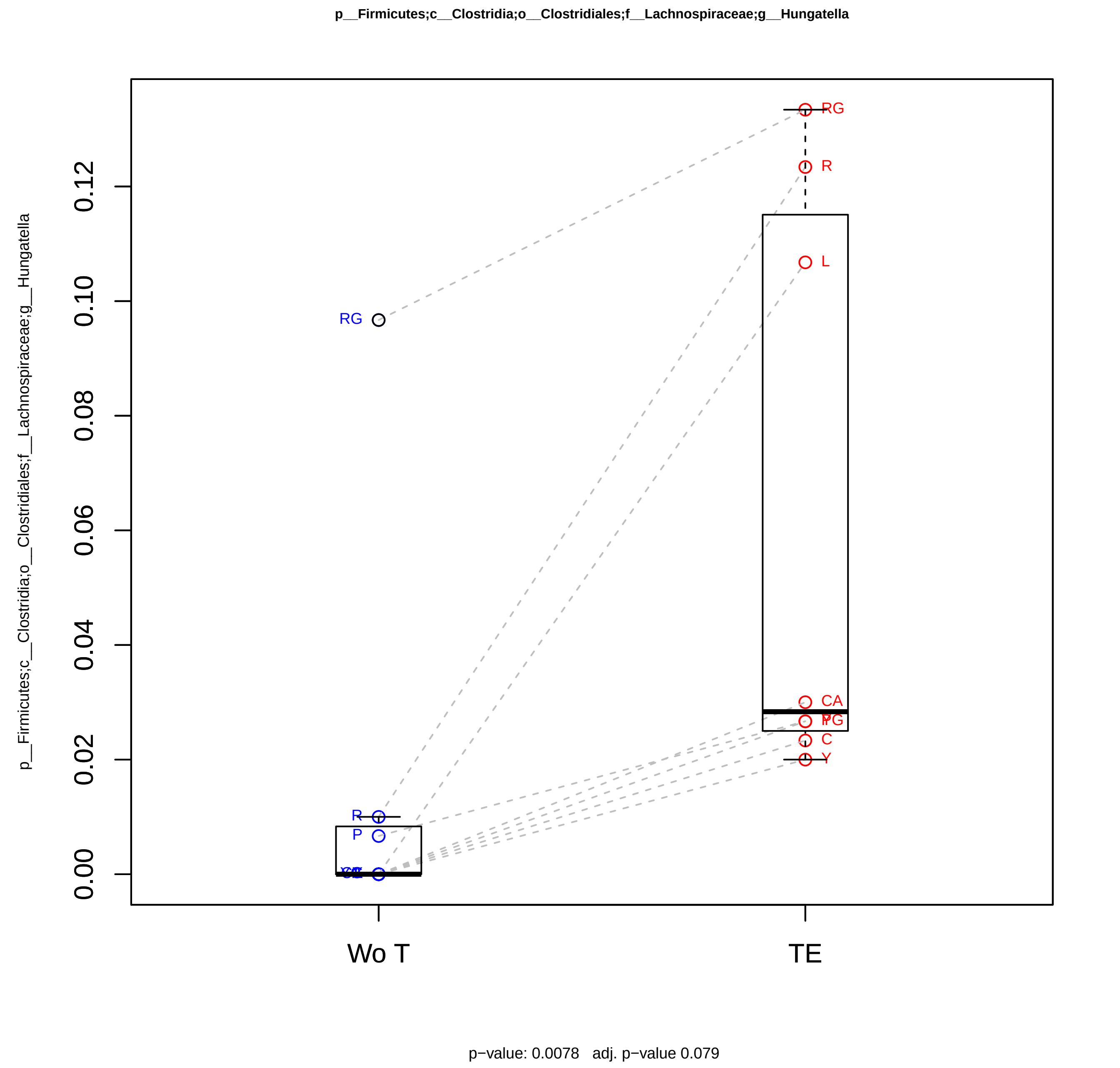
p__Firmicutes;c__Clostridia;o__Clostridiales;f__Lachnospiraceae;g__Hungatella
p__Firmicutes;c__Clostridia;o__Clostridiales;f__Lachnospiraceae;g__Hungatella
0.00
0.02
0.04
0.06
0.08
0.10
0.12
RG
R
P
RG
R
L
CA
PG
C
Y
Wo T
TE
p−value: 0.0078   adj. p−value 0.079

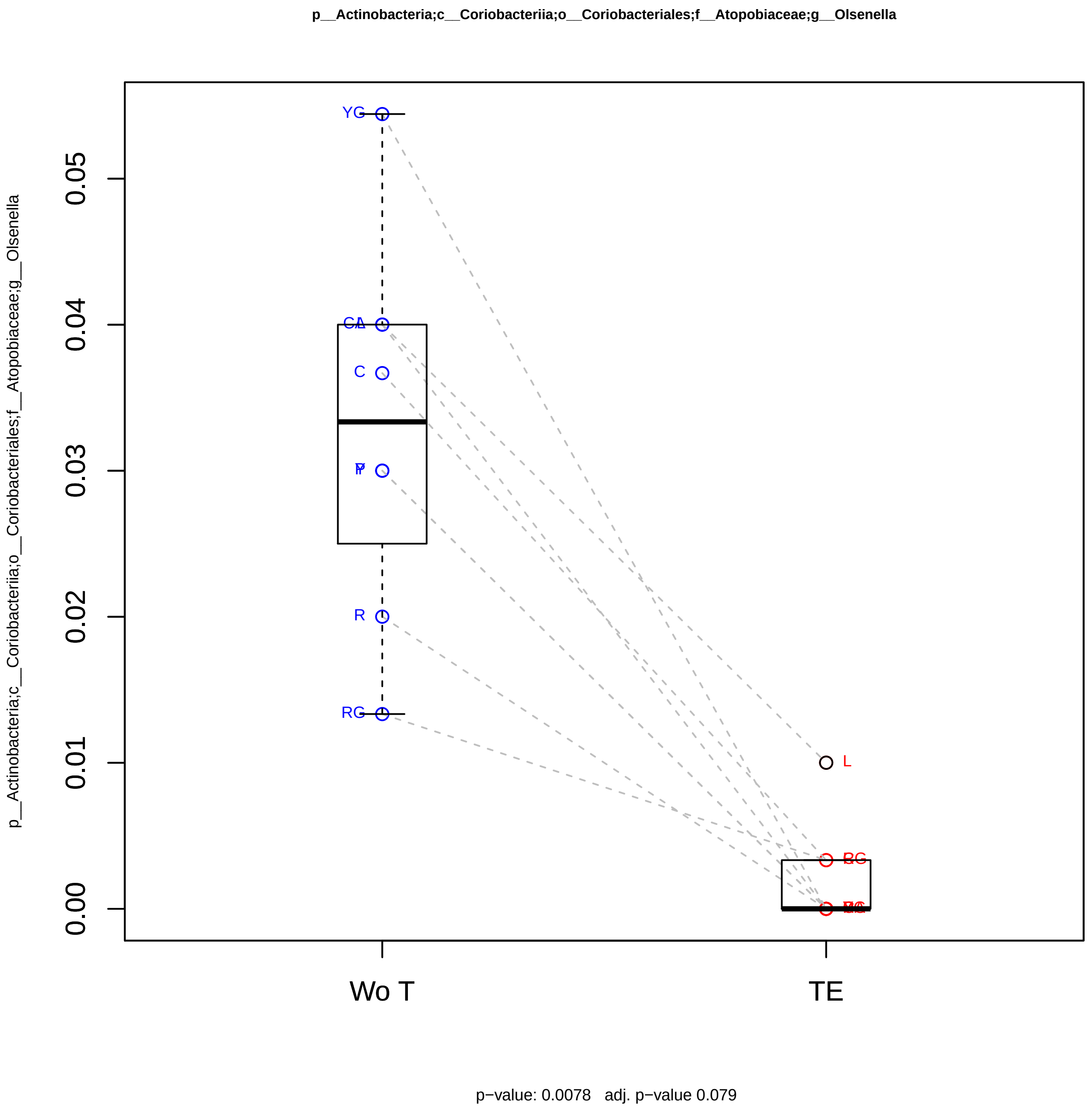
p__Actinobacteria;c__Coriobacteriia;o__Coriobacteriales;f__Atopobiaceae;g__Olsenella
p__Actinobacteria;c__Coriobacteriia;o__Coriobacteriales;f__Atopobiaceae;g__Olsenella
0.00
0.01
0.02
0.03
0.04
0.05
YG
CA
C
P
R
RG
L
Wo T
TE
p−value: 0.0078 adj. p−value 0.079

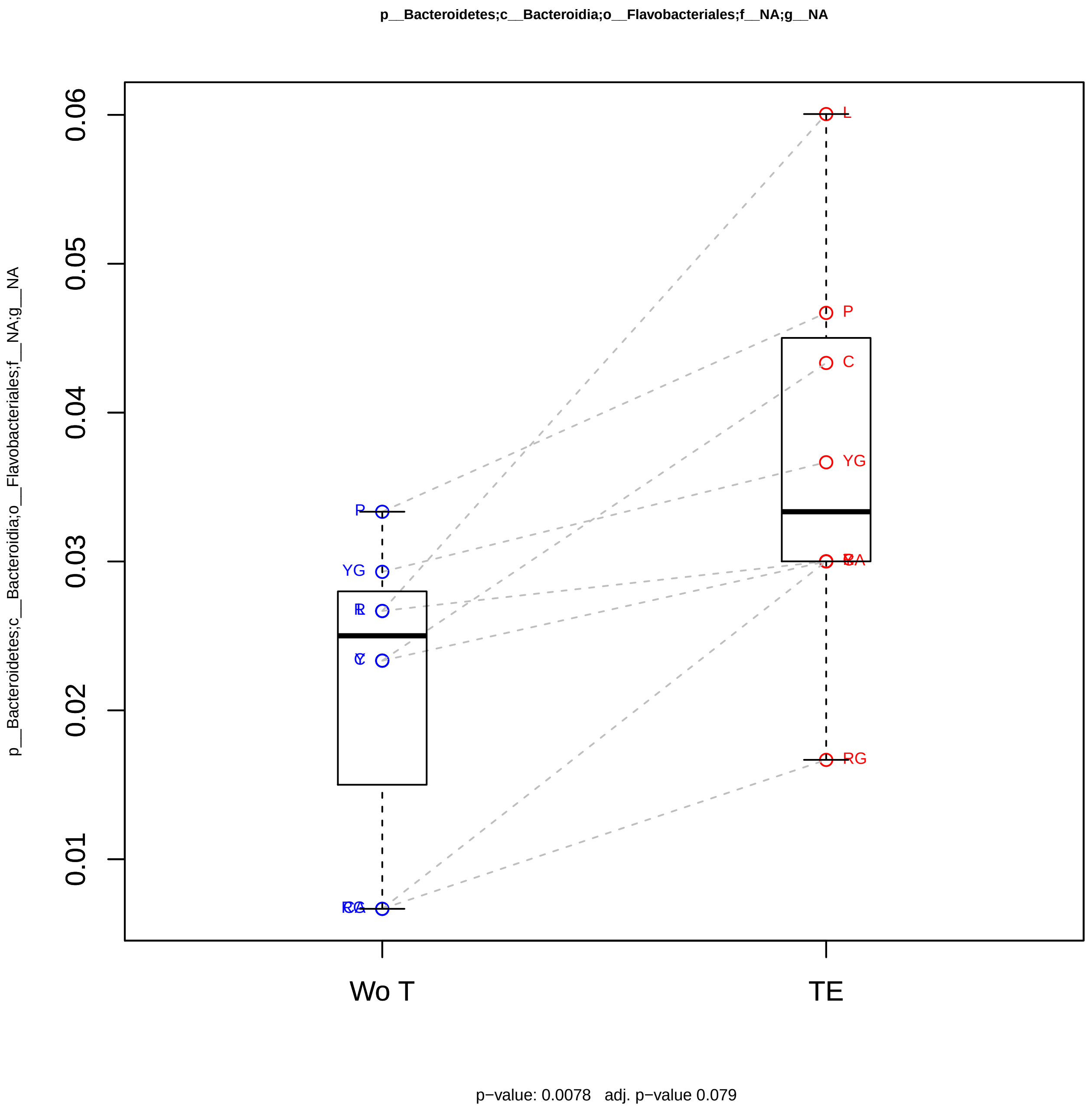
p__Bacteroidetes;c__Bacteroidia;o__Flavobacteriales;f__NA;g__NA
p__Bacteroidetes;c__Bacteroidia;o__Flavobacteriales;f__NA;g__NA
0.06
0.05
0.04
0.03
0.02
0.01
Wo T
TE
L
P
C
YG
RG
p−value: 0.0078 adj. p−value 0.079

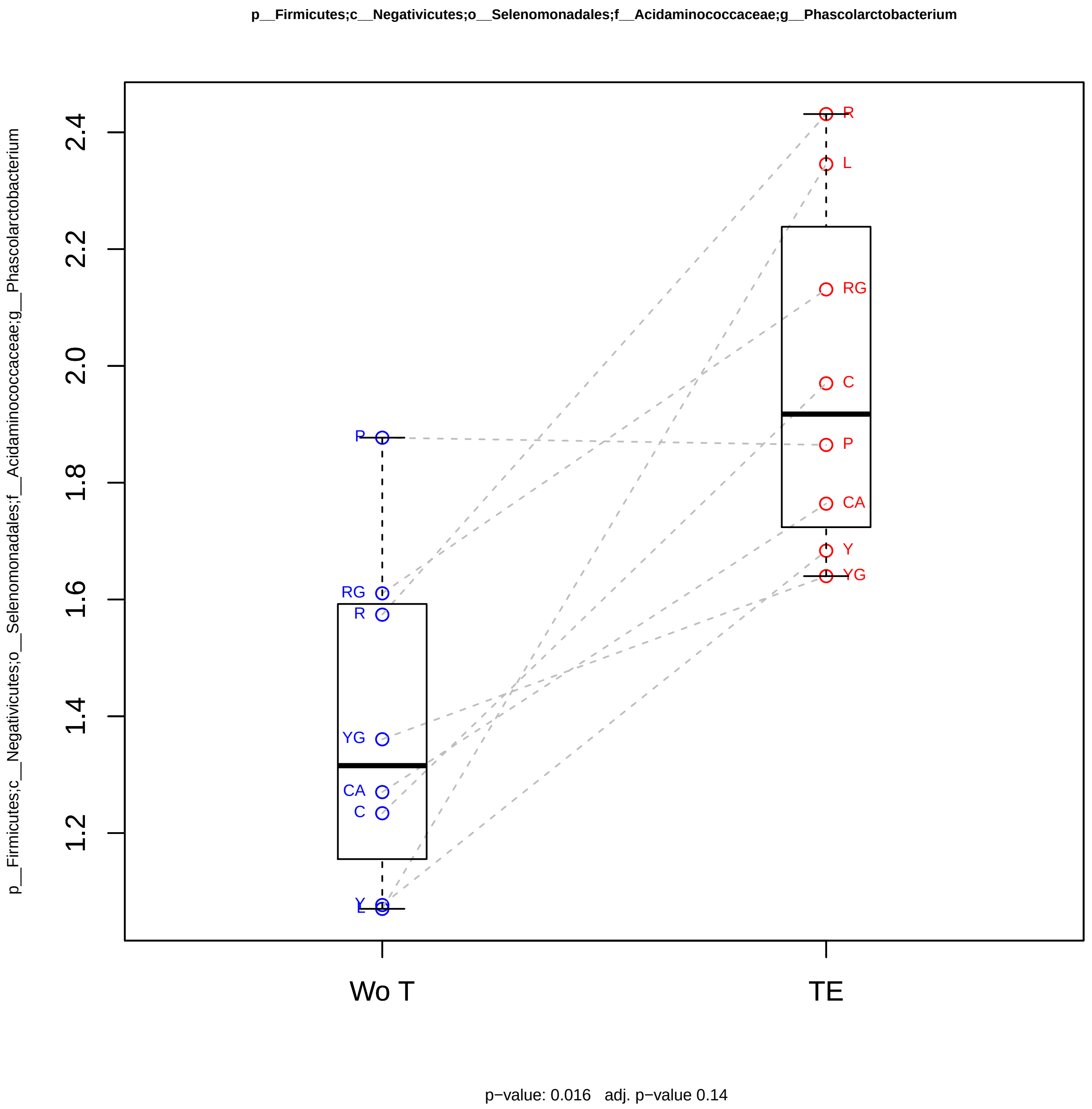
p__Firmicutes;c__Negativicutes;o__Selenomonadales;f__Acidaminococcaceae;g__Phascolarctobacterium
p__Firmicutes;c__Negativicutes;o__Selenomonadales;f__Acidaminococcaceae;g__Phascolarctobacterium
2.4
2.2
2.0
1.8
1.6
1.4
1.2
P
RG
R
YG
CA
C
Y
L
R
L
RG
C
P
CA
Y
YG
Wo T
TE
p−value: 0.016 adj. p−value 0.14

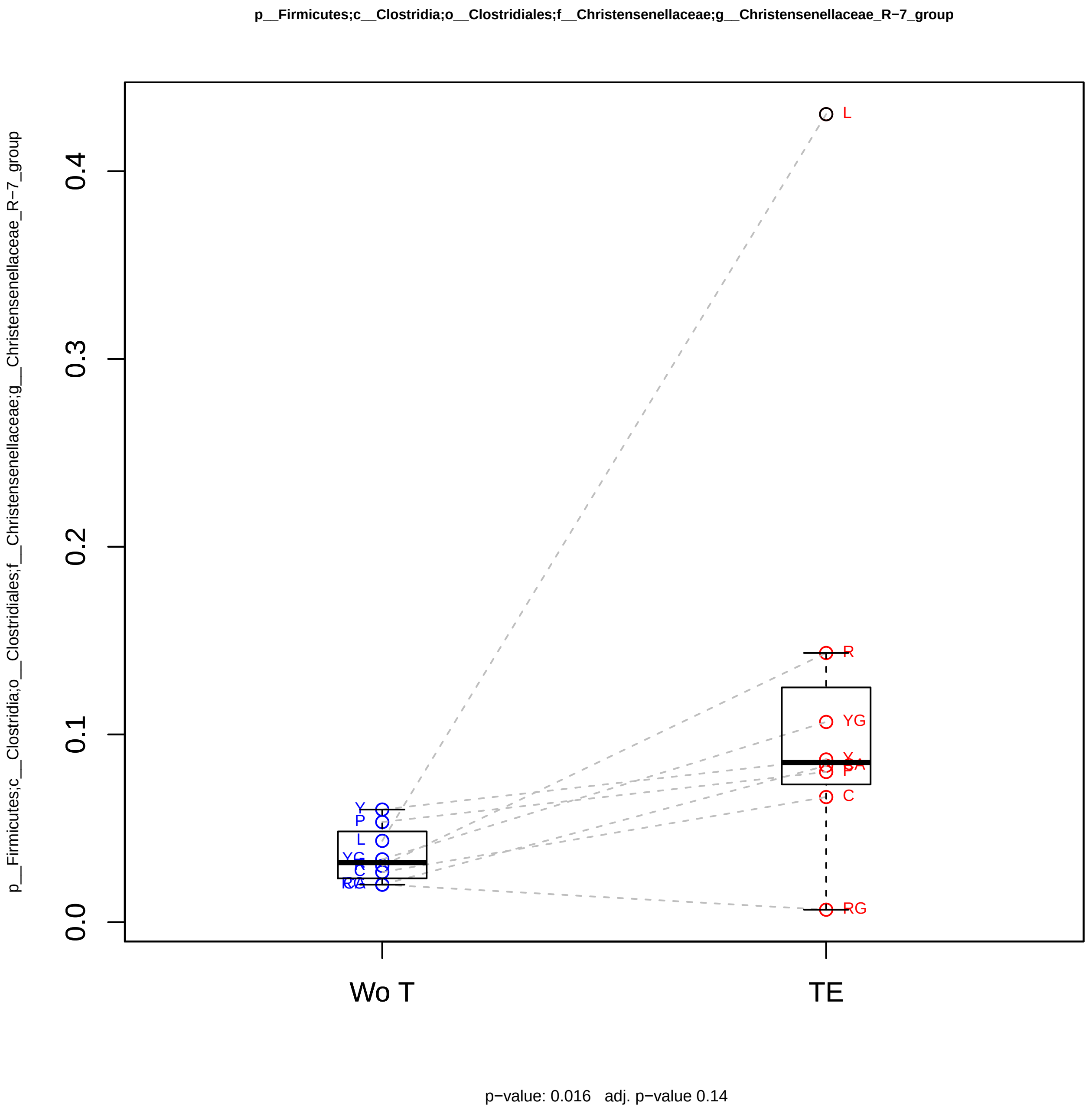
p__Firmicutes;c__Clostridia;o__Clostridiales;f__Christensenellaceae;g__Christensenellaceae_R−7_group
p__Firmicutes;c__Clostridia;o__Clostridiales;f__Christensenellaceae;g__Christensenellaceae_R−7_group
0.0
0.1
0.2
0.3
0.4
Wo T
TE
L
R
YG
C
RG
Y
P
p−value: 0.016 adj. p−value 0.14

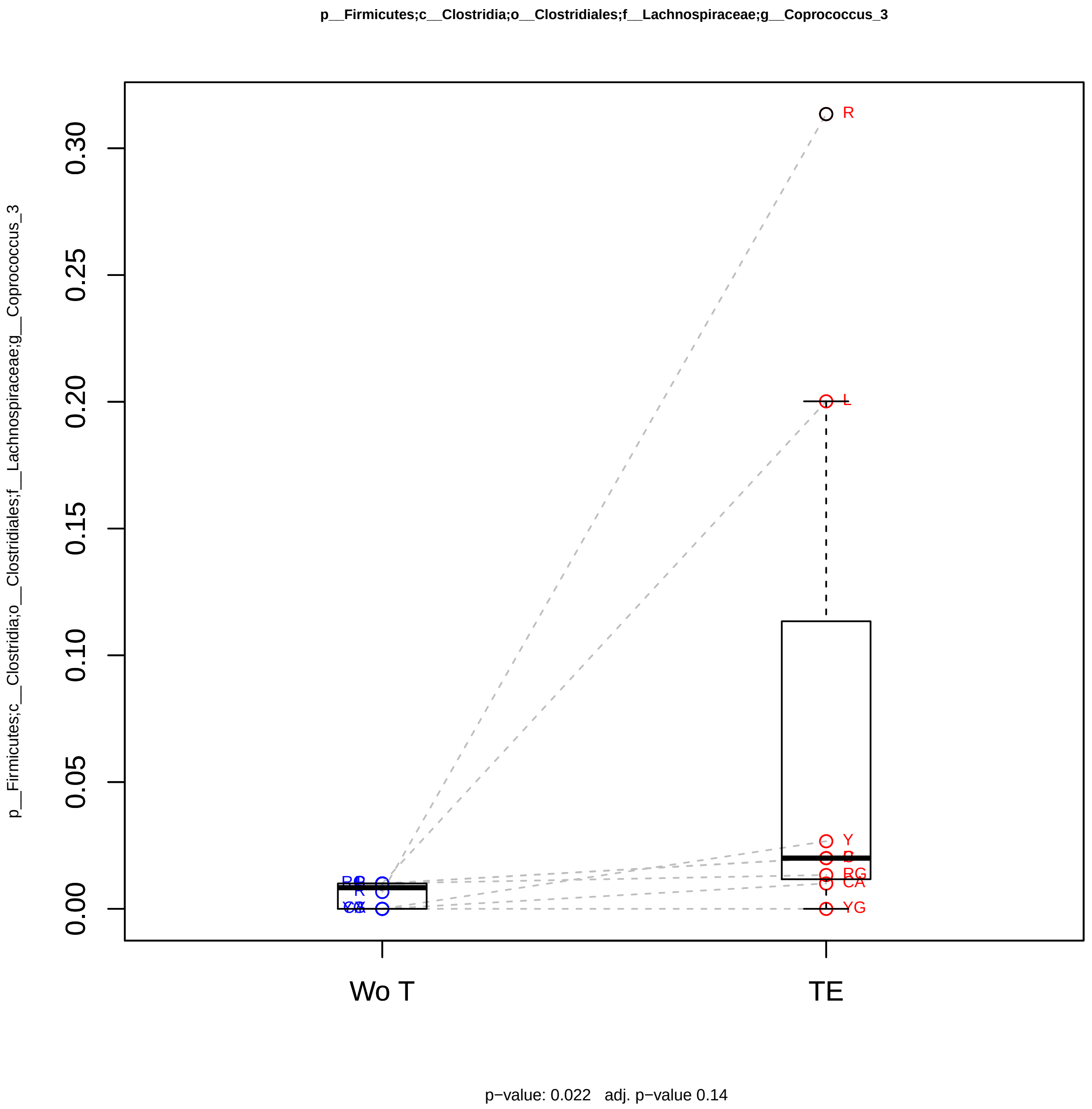

p__Firmicutes;c__Clostridia;o__Clostridiales;f__Lachnospiraceae;g__Coprococcus_3
p__Firmicutes;c__Clostridia;o__Clostridiales;f__Lachnospiraceae;g__Coprococcus_3
0.00
0.05
0.10
0.15
0.20
0.25
0.30
R
L
Y
RG
CA
YG
Wo T
TE
p−value: 0.022 adj. p−value 0.14

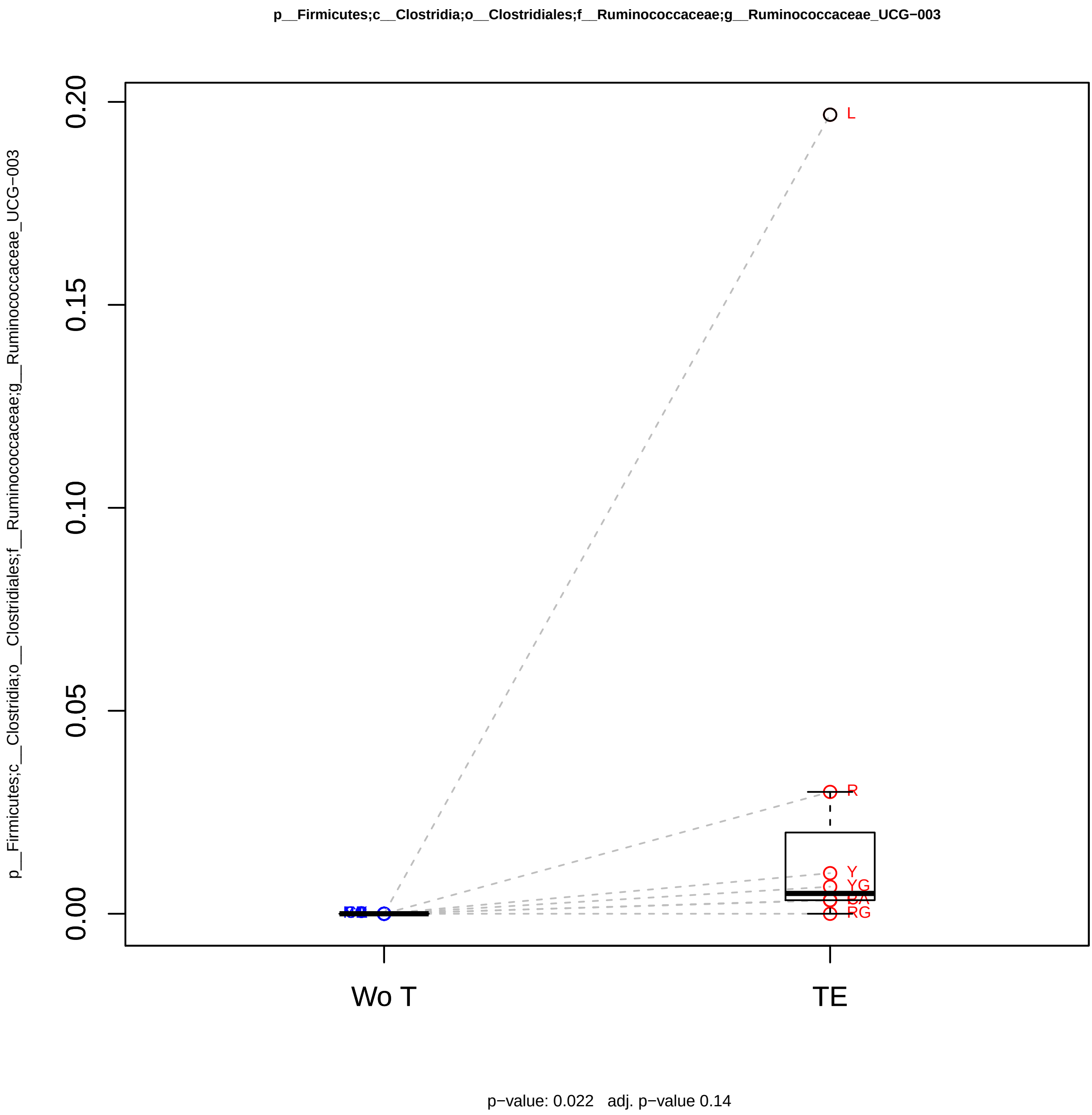
p__Firmicutes;c__Clostridia;o__Clostridiales;f__Ruminococcaceae;g__Ruminococcaceae_UCG−003
p__Firmicutes;c__Clostridia;o__Clostridiales;f__Ruminococcaceae;g__Ruminococcaceae_UCG−003
0.00
0.05
0.10
0.15
0.20
L
R
Y
YG
RG
Wo T
TE
p−value: 0.022 adj. p−value 0.14

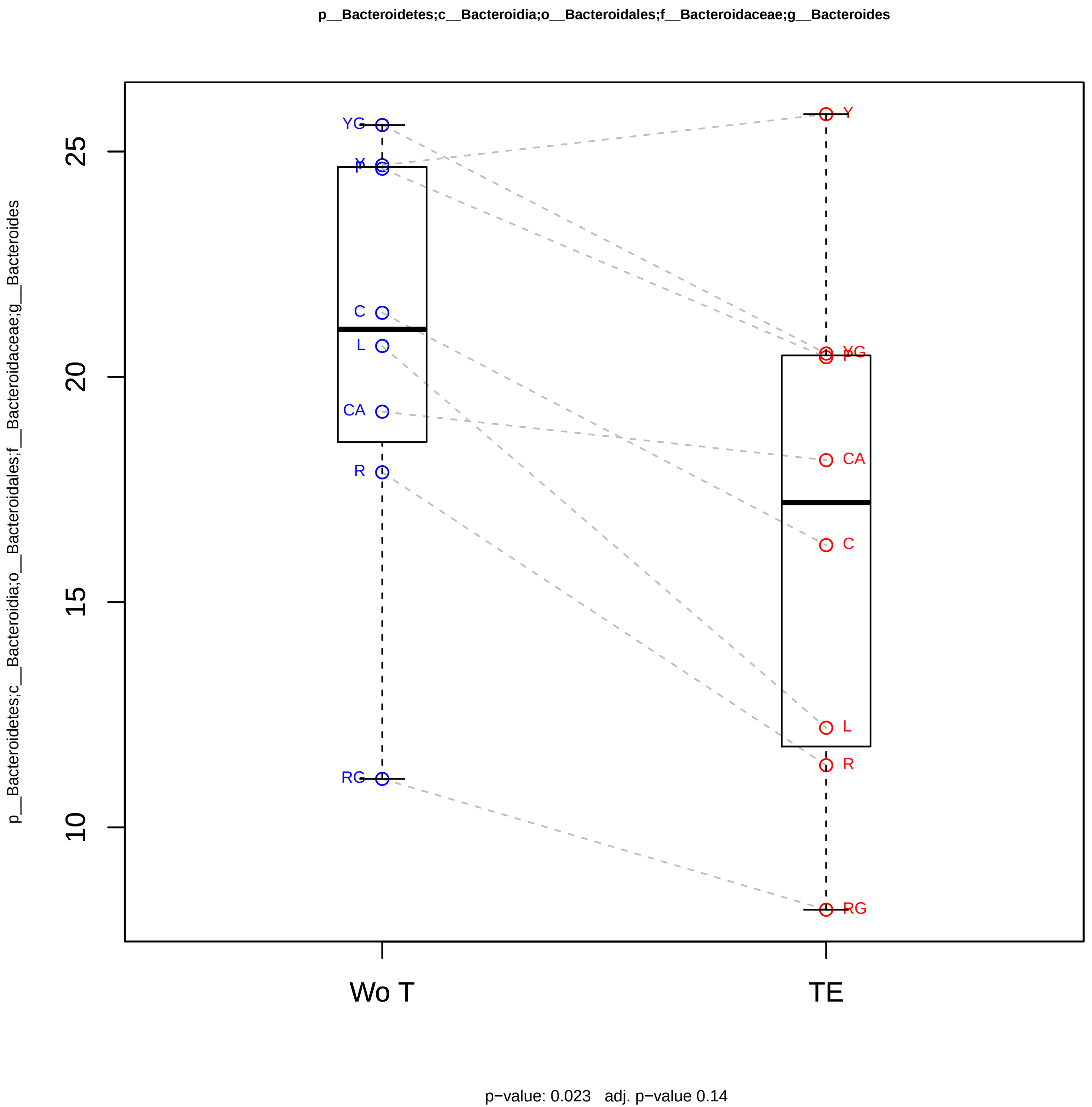
p__Bacteroidetes;c__Bacteroidia;o__Bacteroidales;f__Bacteroidaceae;g__Bacteroides
p__Bacteroidetes;c__Bacteroidia;o__Bacteroidales;f__Bacteroidaceae;g__Bacteroides
10
15
20
25
Wo T
TE
YG
Y
P
C
L
CA
R
RG
p−value: 0.023 adj. p−value 0.14

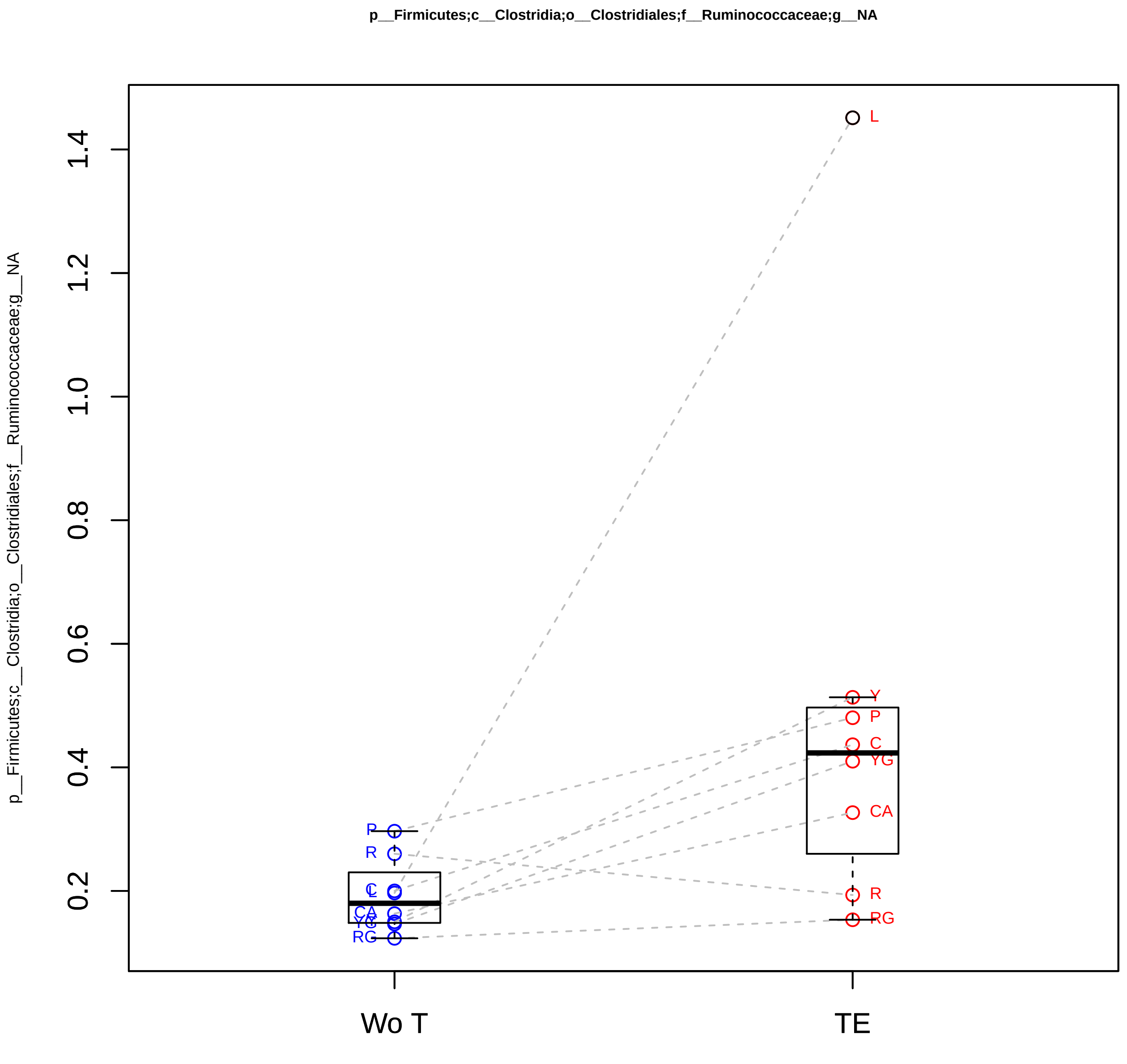

p−value: 0.023 adj. p−value 0.14

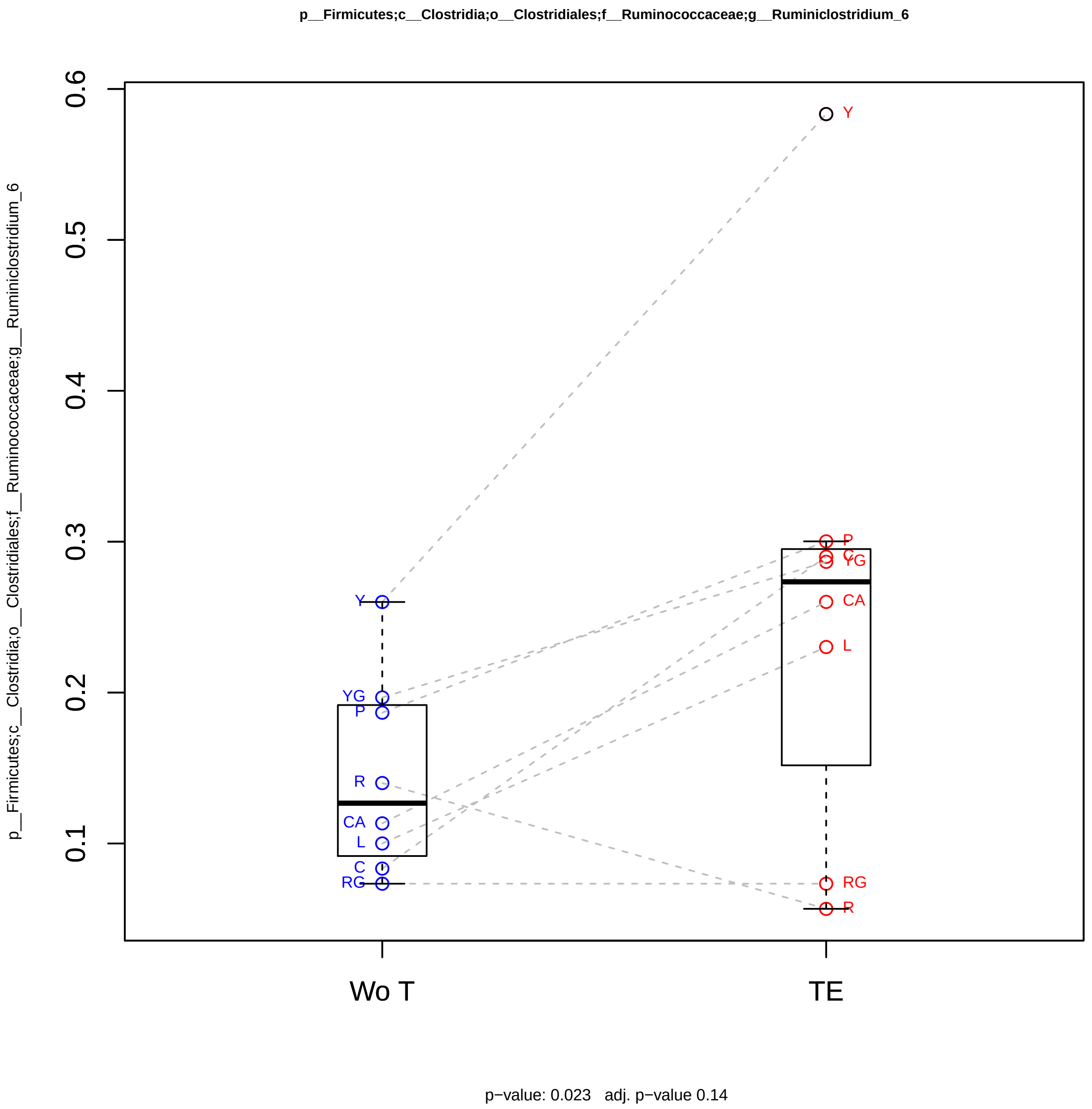
p__Firmicutes;c__Clostridia;o__Clostridiales;f__Ruminococcaceae;g__Ruminiclostridium_6
p__Firmicutes;c__Clostridia;o__Clostridiales;f__Ruminococcaceae;g__Ruminiclostridium_6
0.6
0.5
0.4
0.3
0.2
0.1
Y
YG
P
R
CA
L
C
RG
Y
P
C
YG
CA
L
RG
R
Wo T
TE
p−value: 0.023 adj. p−value 0.14

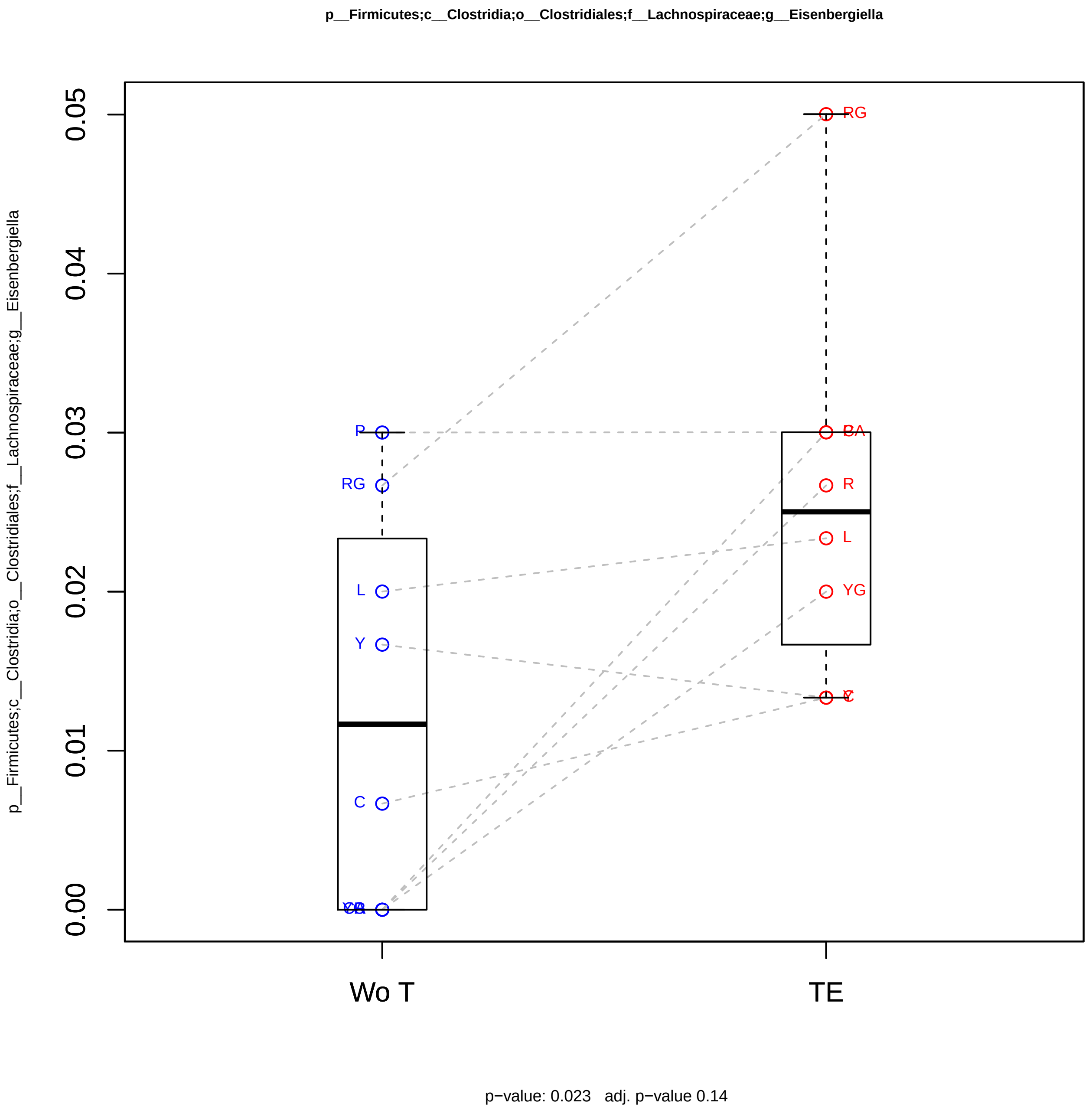
p__Firmicutes;c__Clostridia;o__Clostridiales;f__Lachnospiraceae;g__Eisenbergiella
p__Firmicutes;c__Clostridia;o__Clostridiales;f__Lachnospiraceae;g__Eisenbergiella
0.00
0.01
0.02
0.03
0.04
0.05
P
RG
L
Y
C
RG
R
L
YG
Wo T
TE
p−value: 0.023   adj. p−value 0.14

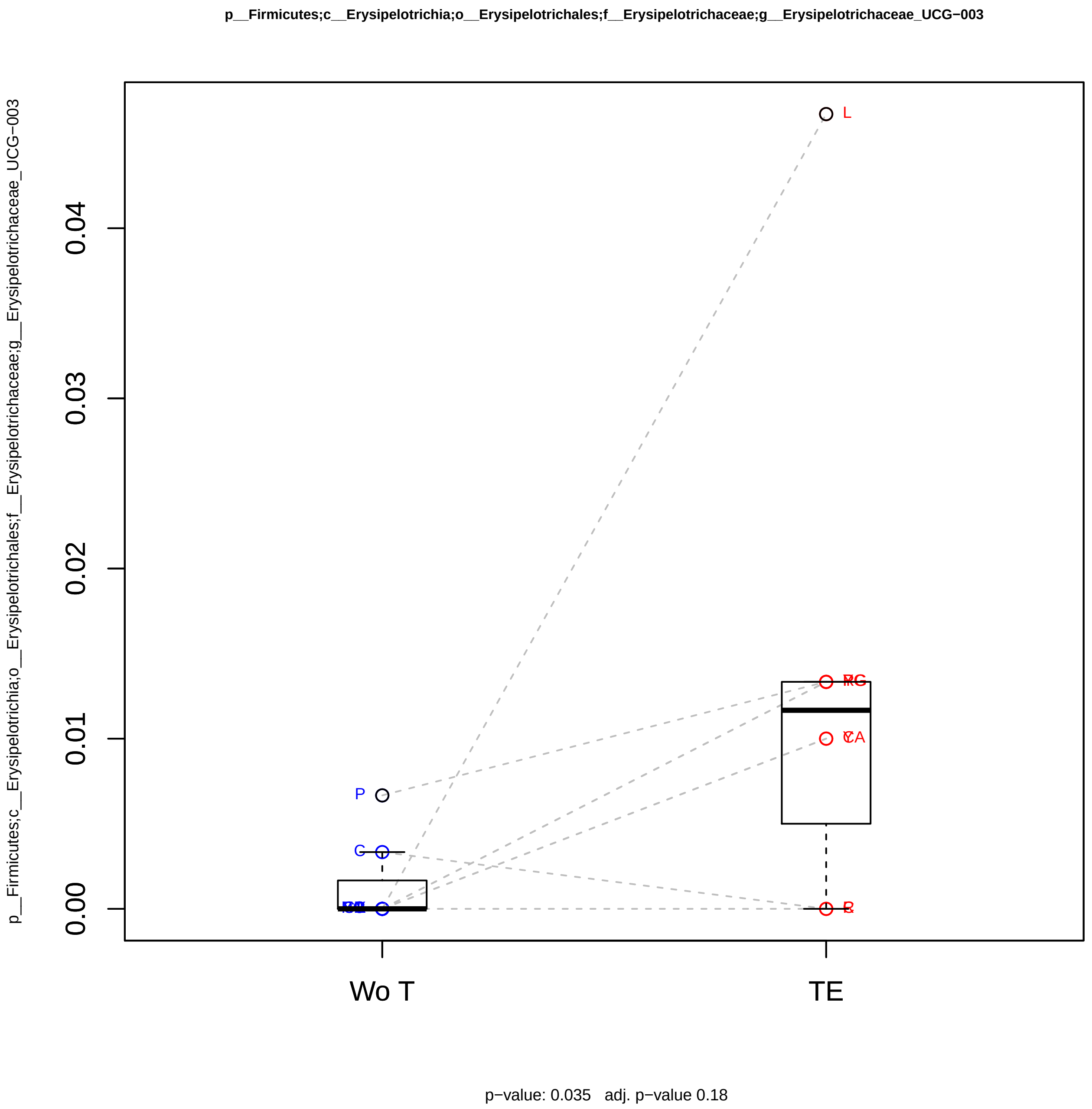
p__Firmicutes;c__Erysipelotrichia;o__Erysipelotrichales;f__Erysipelotrichaceae;g__Erysipelotrichaceae_UCG−003
p__Firmicutes;c__Erysipelotrichia;o__Erysipelotrichales;f__Erysipelotrichaceae;g__Erysipelotrichaceae_UCG−003
0.00
0.01
0.02
0.03
0.04
L
P
G
CA
Wo T
TE
p−value: 0.035 adj. p−value 0.18

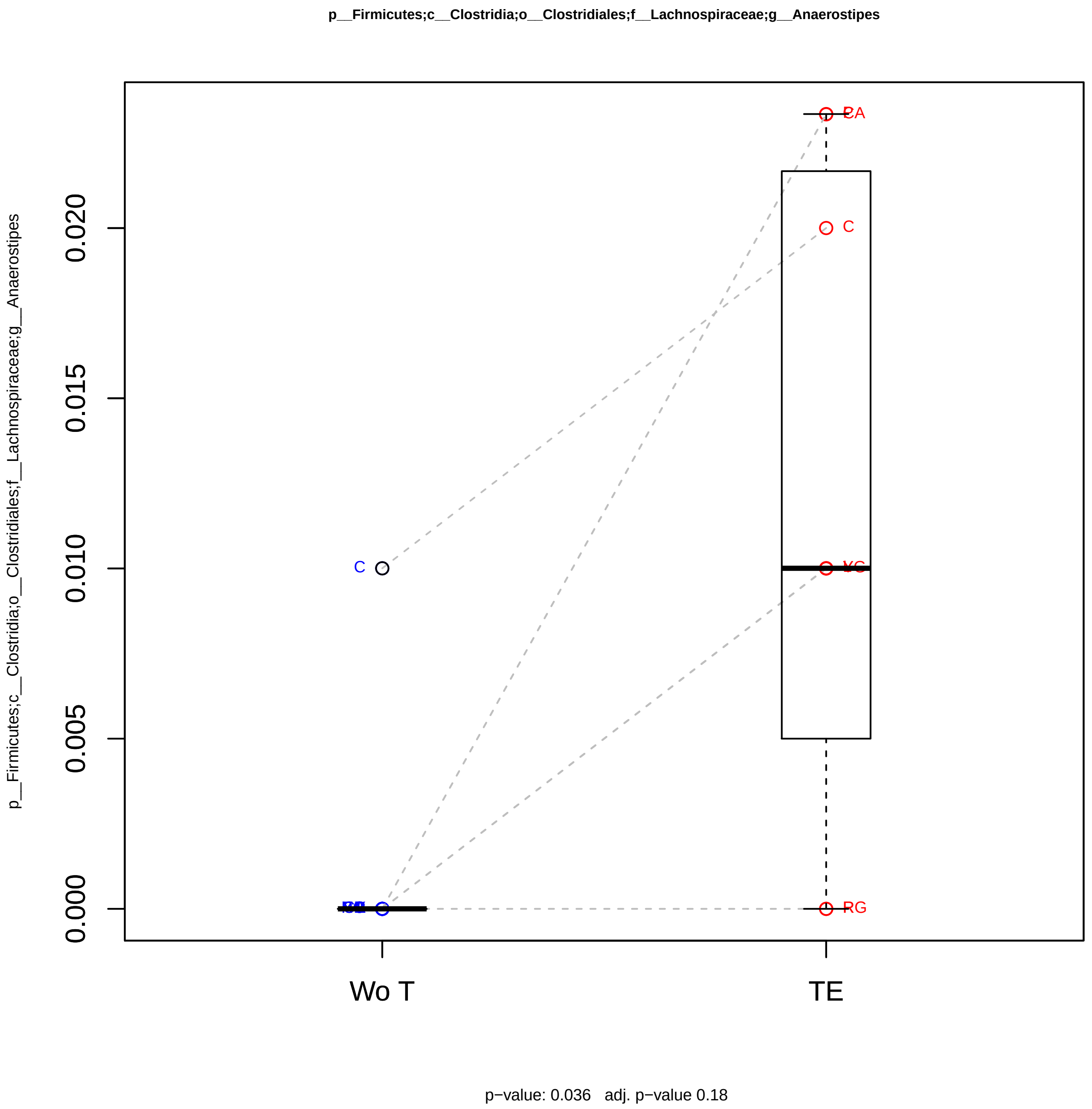
p__Firmicutes;c__Clostridia;o__Clostridiales;f__Lachnospiraceae;g__Anaerostipes
p__Firmicutes;c__Clostridia;o__Clostridiales;f__Lachnospiraceae;g__Anaerostipes
0.000
0.005
0.010
0.015
0.020
Wo T
TE
C
CA
C
RG
p−value: 0.036 adj. p−value 0.18

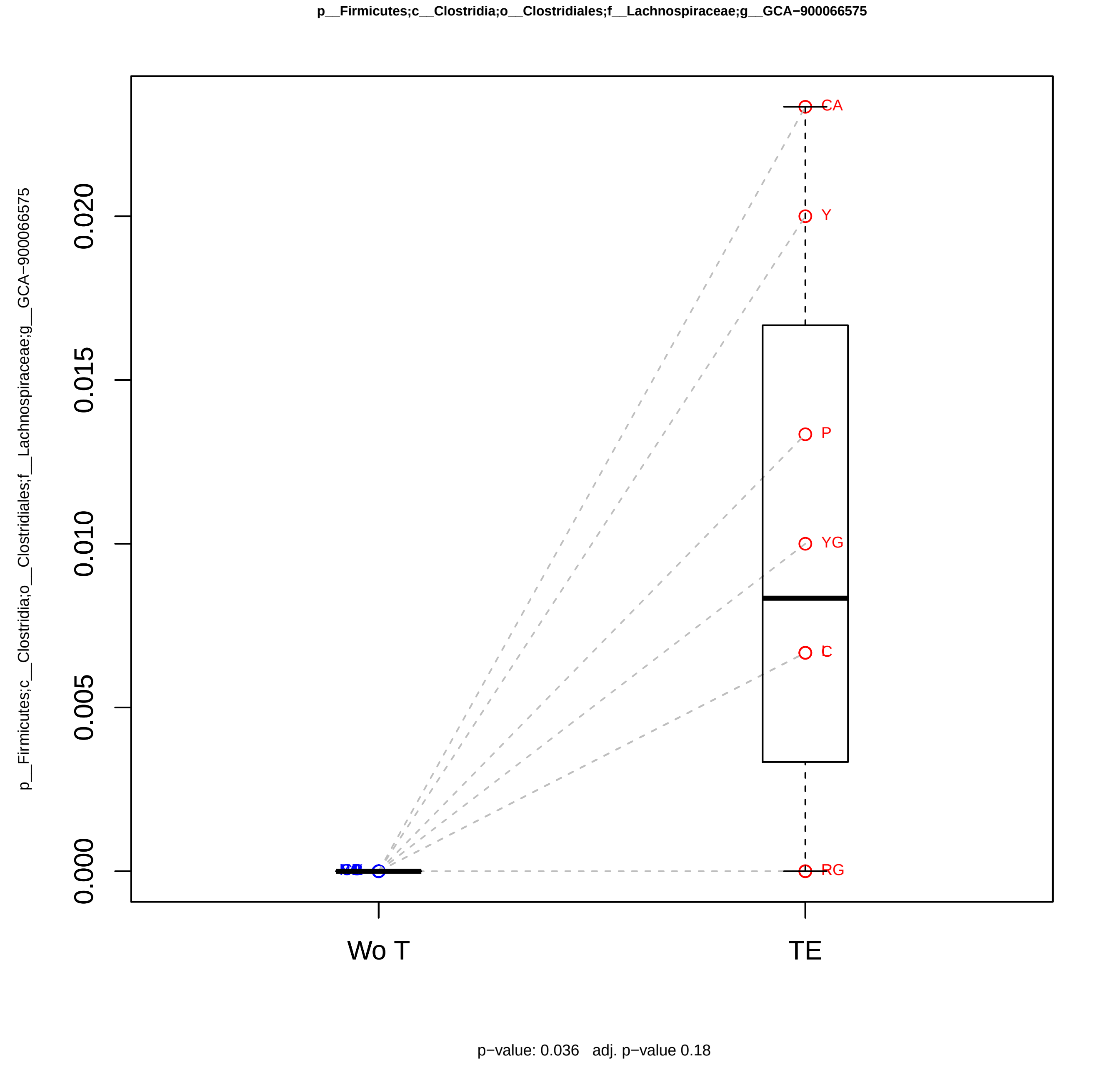
p__Firmicutes;c__Clostridia;o__Clostridiales;f__Lachnospiraceae;g__GCA−900066575
p__Firmicutes;c__Clostridia;o__Clostridiales;f__Lachnospiraceae;g__GCA−900066575
0.000
0.005
0.010
0.015
0.020
CA
Y
P
YG
C
RG
Wo T
TE
p−value: 0.036 adj. p−value 0.18

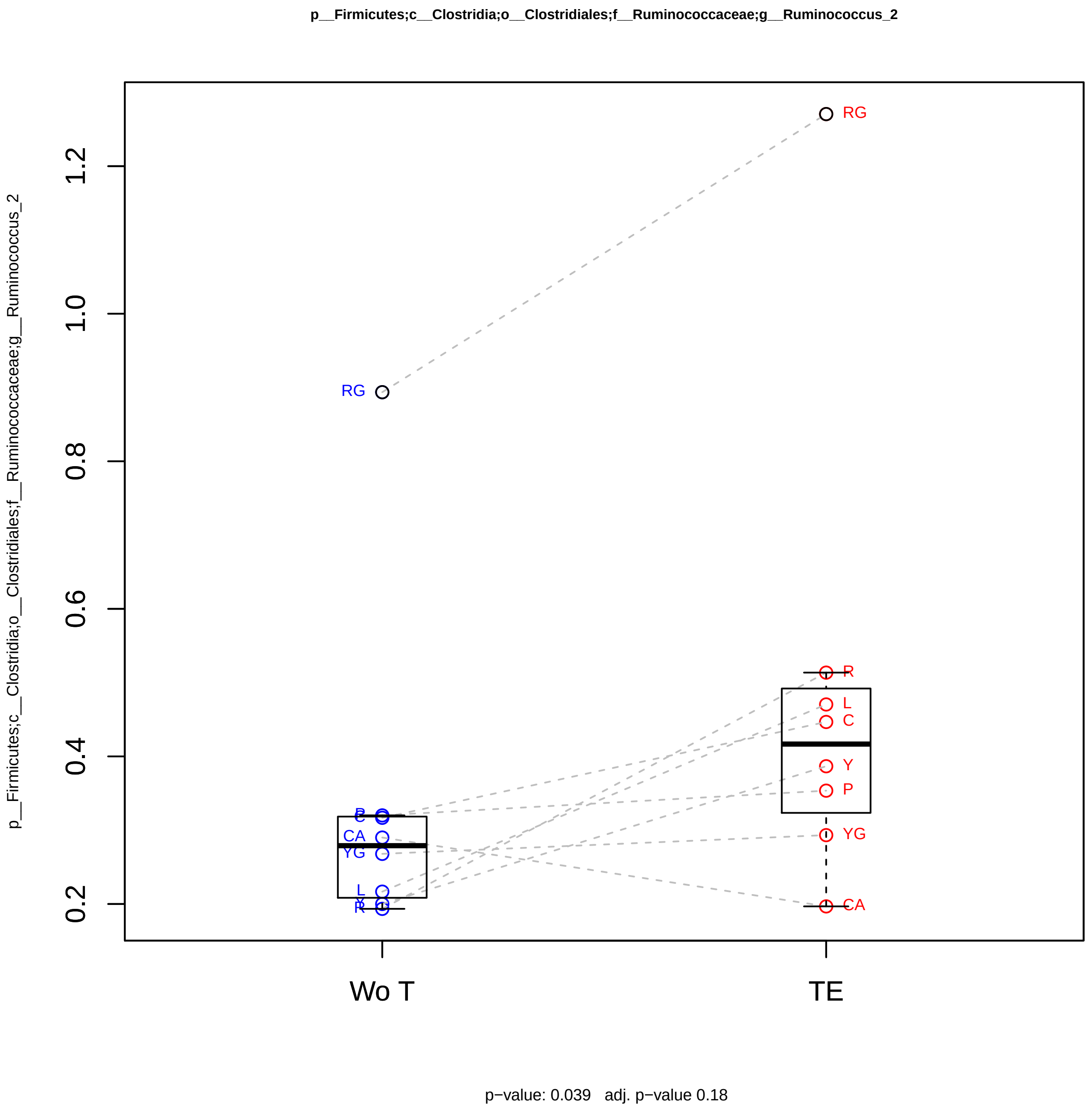
p__Firmicutes;c__Clostridia;o__Clostridiales;f__Ruminococcaceae;g__Ruminococcus_2
p__Firmicutes;c__Clostridia;o__Clostridiales;f__Ruminococcaceae;g__Ruminococcus_2
0.2
0.4
0.6
0.8
1.0
1.2
RG
CA
YG
L
R
P
C
Y
Wo T
TE
p−value: 0.039 adj. p−value 0.18

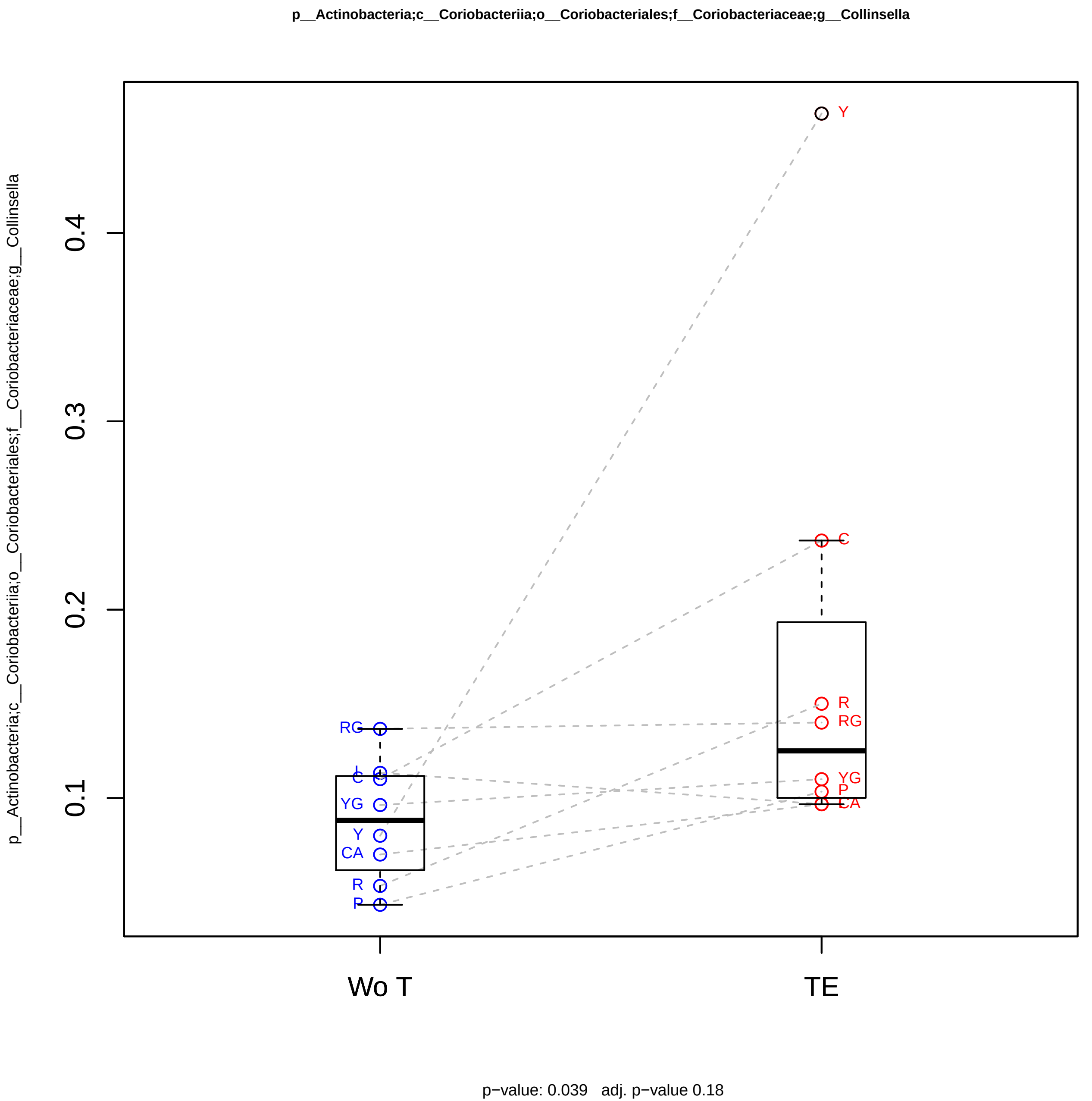
p__Actinobacteria;c__Coriobacteriia;o__Coriobacteriales;f__Coriobacteriaceae;g__Collinsella
p__Actinobacteria;c__Coriobacteriia;o__Coriobacteriales;f__Coriobacteriaceae;g__Collinsella
0.1
0.2
0.3
0.4
Wo T
TE
RG
L
C
YG
Y
CA
R
P
Y
C
R
RG
YG
P
CA
p−value: 0.039 adj. p−value 0.18

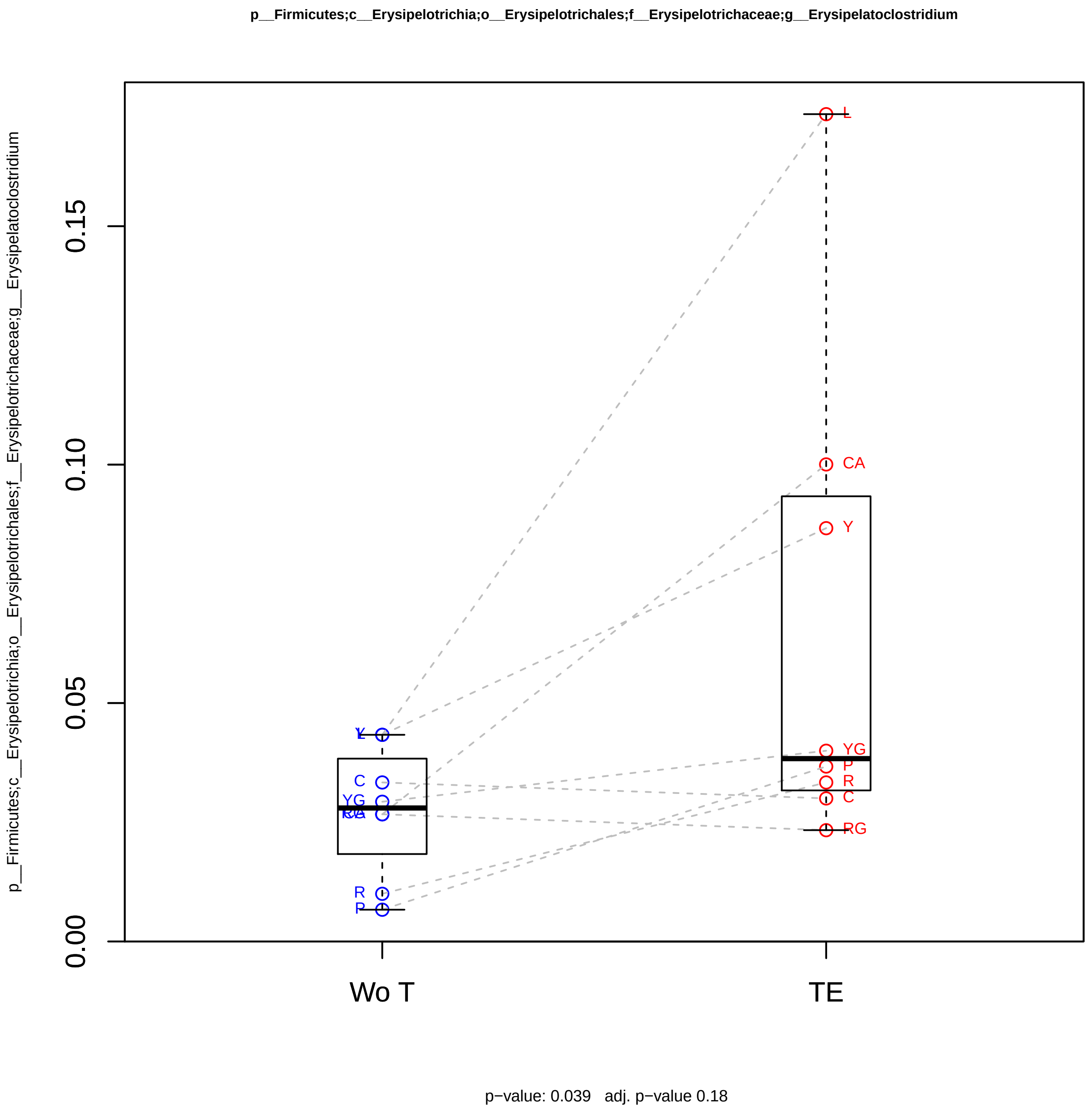
p__Firmicutes;c__Erysipelotrichia;o__Erysipelotrichales;f__Erysipelotrichaceae;g__Erysipelatoclostridium
p__Firmicutes;c__Erysipelotrichia;o__Erysipelotrichales;f__Erysipelotrichaceae;g__Erysipelatoclostridium
0.00
0.05
0.10
0.15
Wo T
TE
L
CA
Y
YG
P
R
C
RG
p−value: 0.039 adj. p−value 0.18

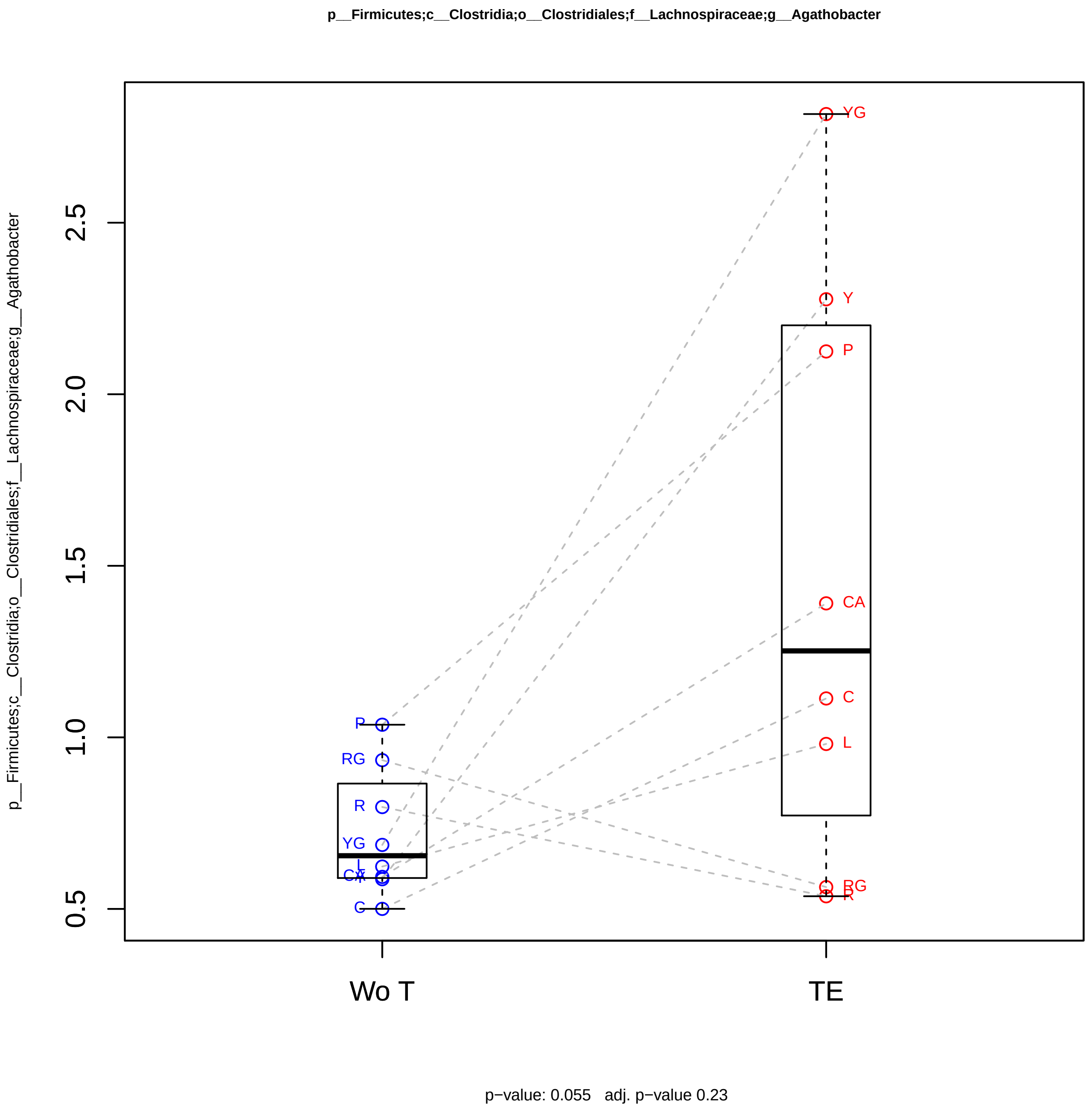
p__Firmicutes;c__Clostridia;o__Clostridiales;f__Lachnospiraceae;g__Agathobacter
p__Firmicutes;c__Clostridia;o__Clostridiales;f__Lachnospiraceae;g__Agathobacter
0.5
1.0
1.5
2.0
2.5
Wo T
TE
P
RG
R
YG
L
CA
Y
C
p−value: 0.055   adj. p−value 0.23

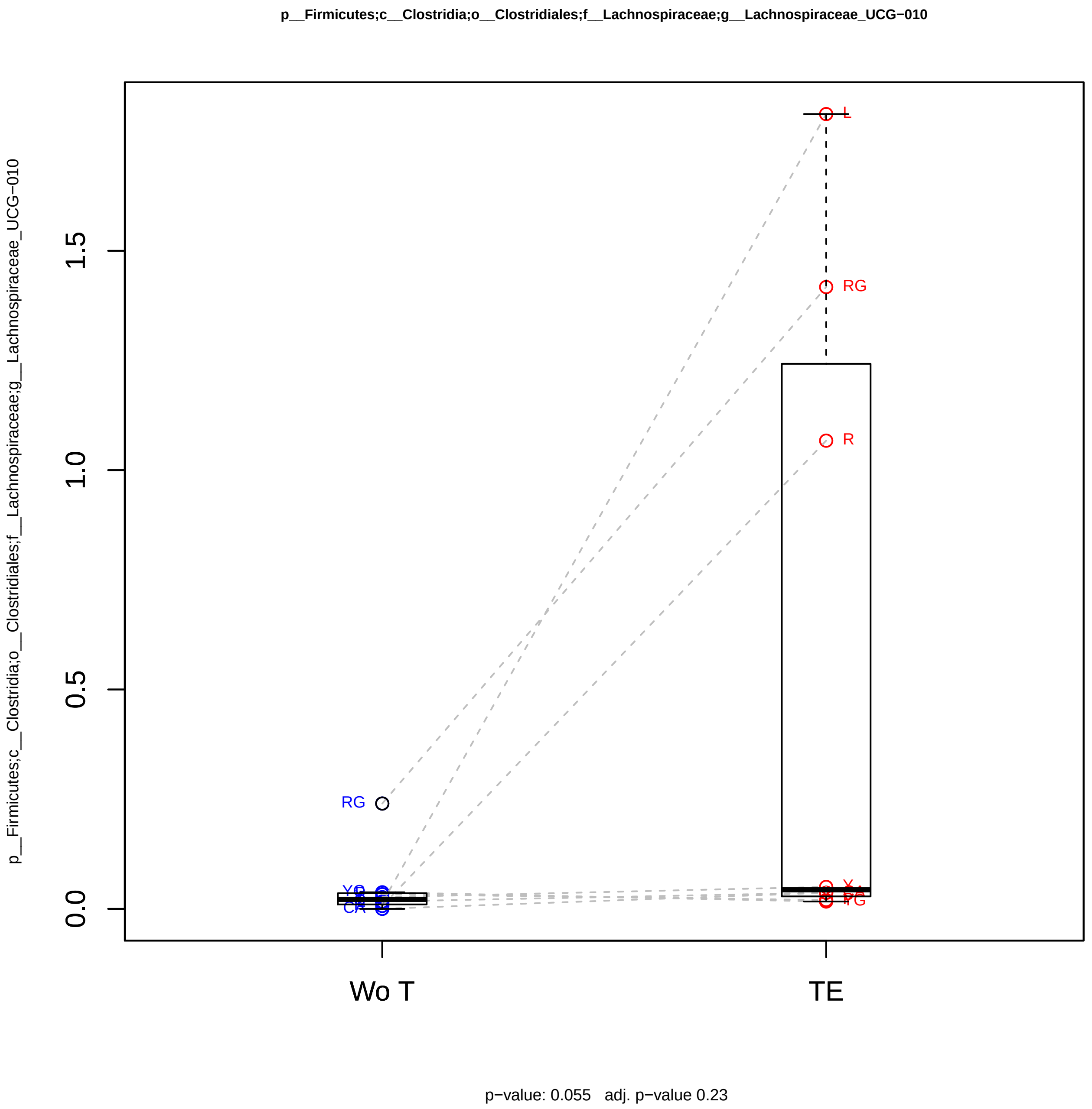

p__Firmicutes;c__Clostridia;o__Clostridiales;f__Lachnospiraceae;g__Lachnospiraceae_UCG−010
p__Firmicutes;c__Clostridia;o__Clostridiales;f__Lachnospiraceae;g__Lachnospiraceae_UCG−010
0.0
0.5
1.0
1.5
L
RG
R
RG
Wo T
TE
p−value: 0.055 adj. p−value 0.23

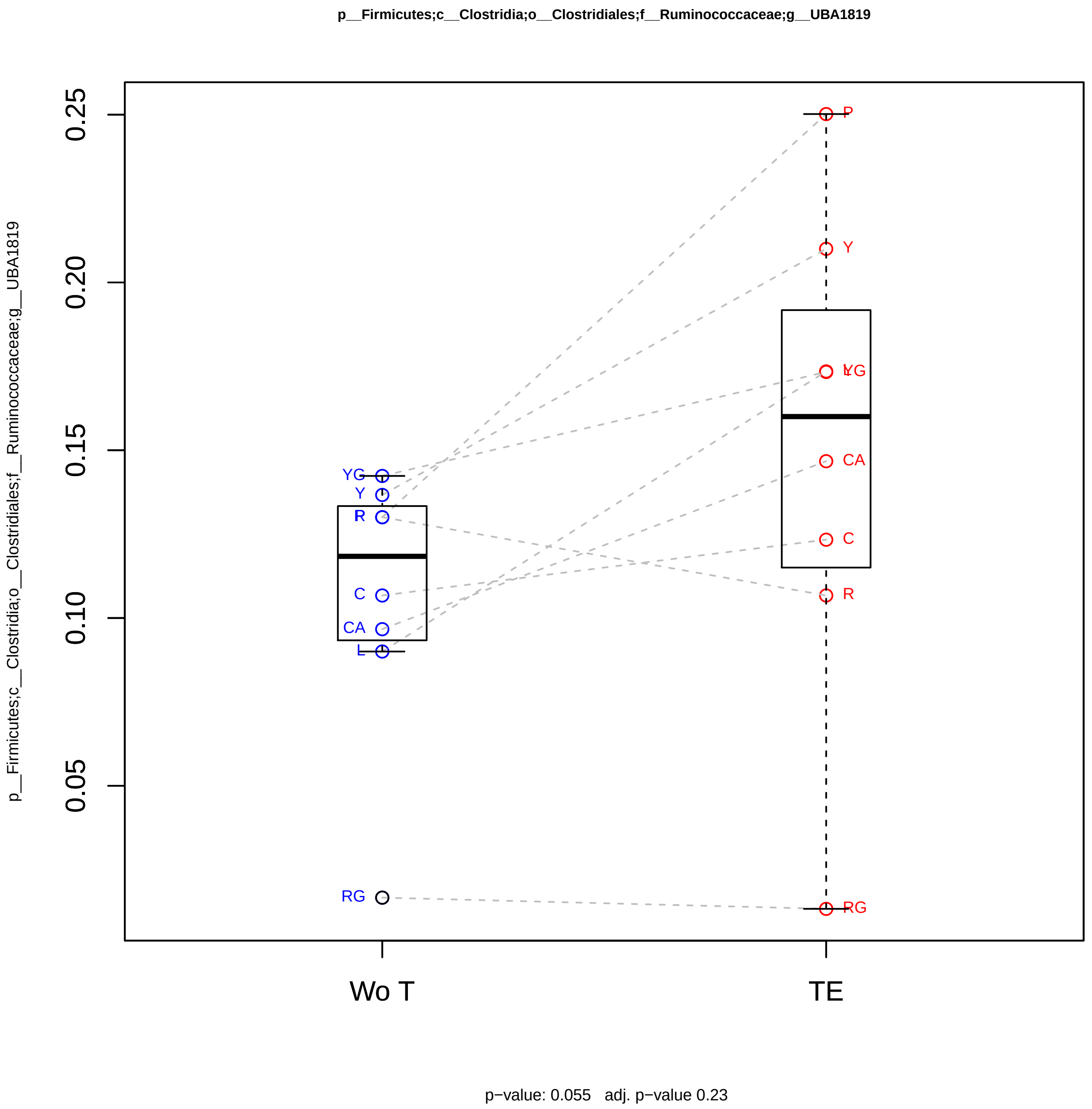
p__Firmicutes;c__Clostridia;o__Clostridiales;f__Ruminococcaceae;g__UBA1819
p__Firmicutes;c__Clostridia;o__Clostridiales;f__Ruminococcaceae;g__UBA1819
0.05
0.10
0.15
0.20
0.25
YG
Y
R
C
CA
L
RG
P
Y
YG
CA
C
R
RG
Wo T
TE
p−value: 0.055 adj. p−value 0.23

p__Firmicutes;c__Clostridia;o__Clostridiales;f__Lachnospiraceae;g__Roseburia

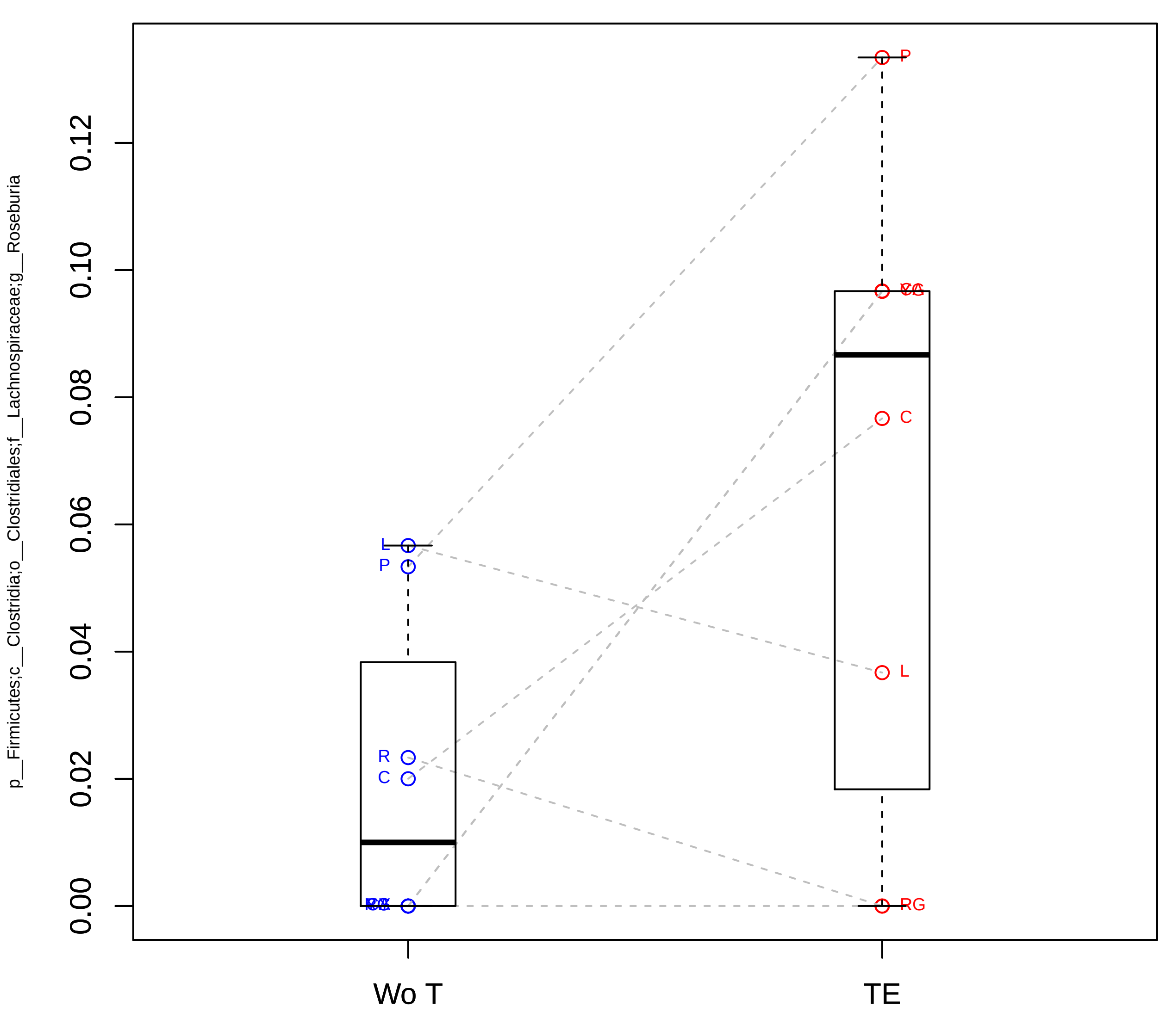

p−value: 0.076 adj. p−value 0.29

p__Firmicutes;c__Clostridia;o__Clostridiales;f__Lachnospiraceae;g__Lachnoclostridium

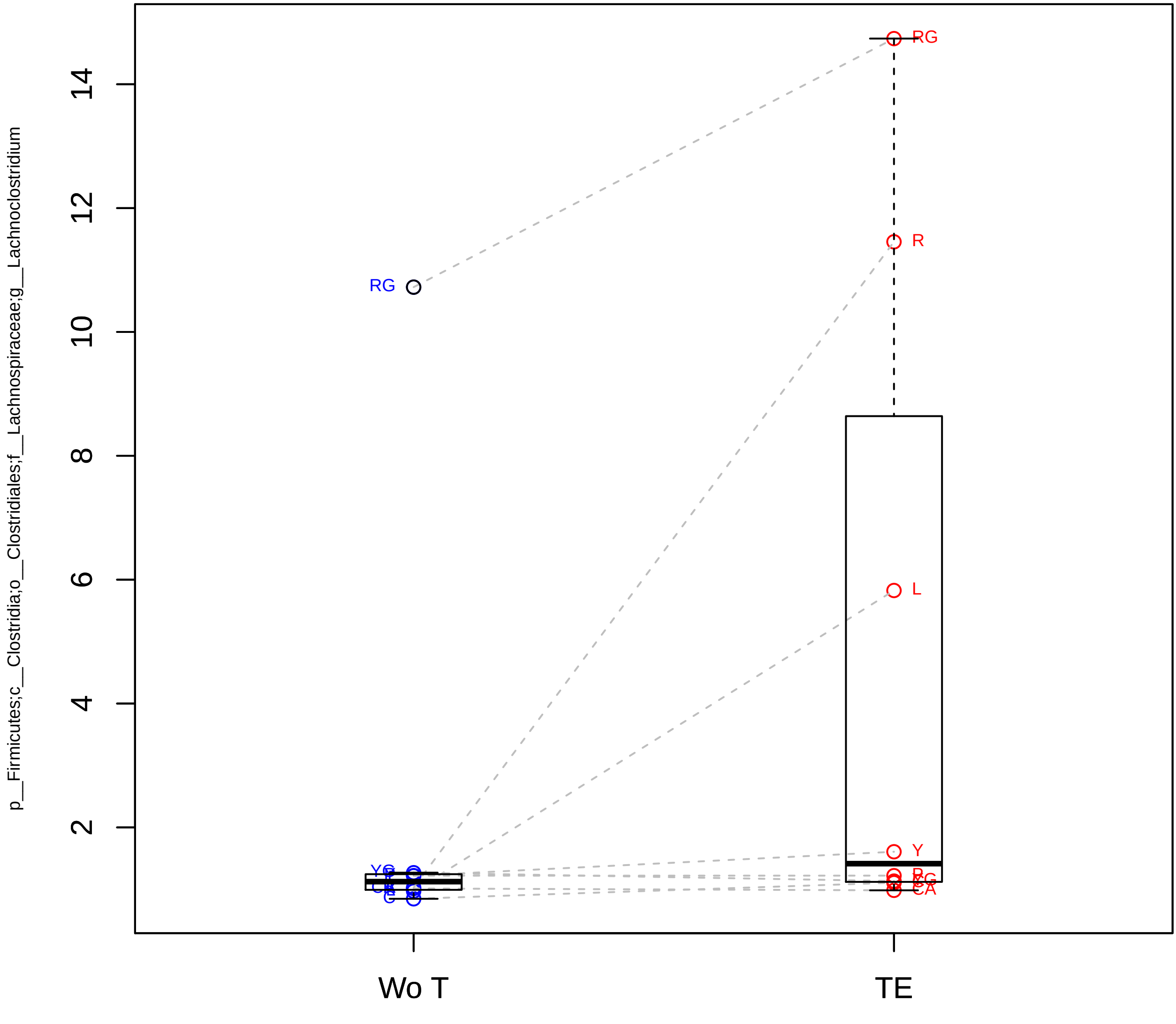

p−value: 0.078   adj. p−value 0.29

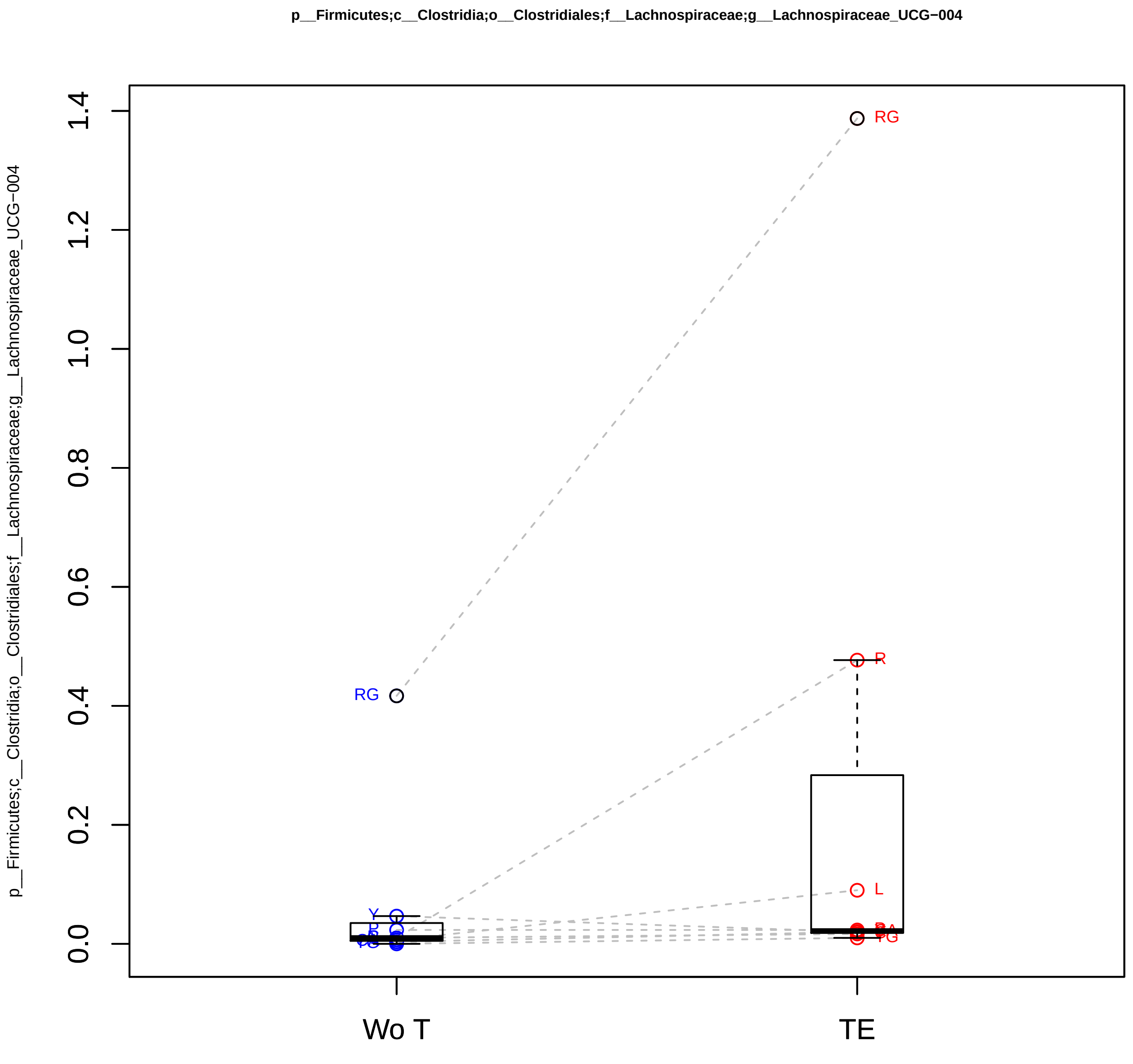

p−value: 0.078 adj. p−value 0.29

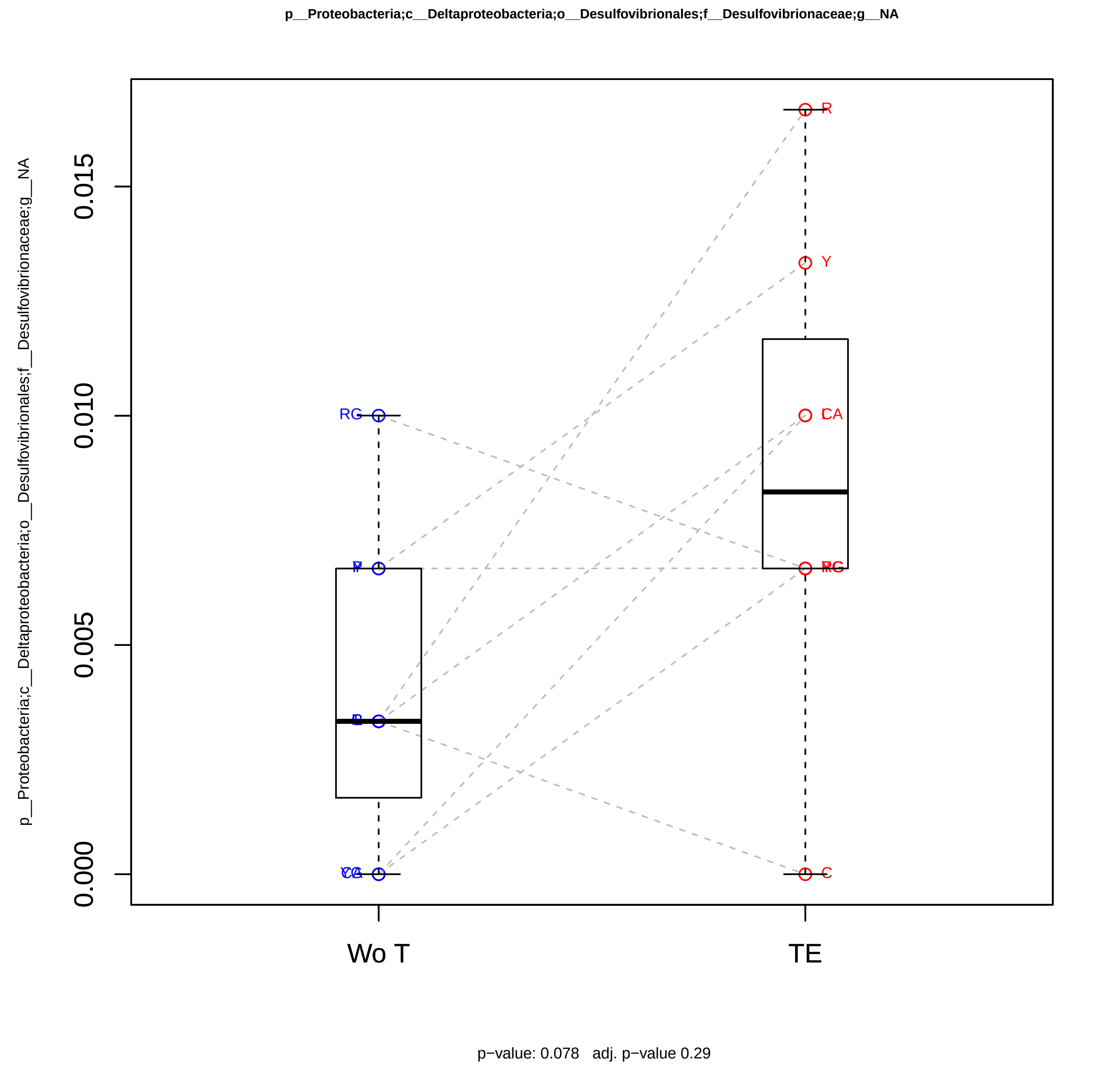
p__Proteobacteria;c__Deltaproteobacteria;o__Desulfovibrionales;f__Desulfovibrionaceae;g__NA
p__Proteobacteria;c__Deltaproteobacteria;o__Desulfovibrionales;f__Desulfovibrionaceae;g__NA
0.000
0.005
0.010
0.015
Wo T
TE
RG
R
Y
CA
C
p−value: 0.078   adj. p−value 0.29

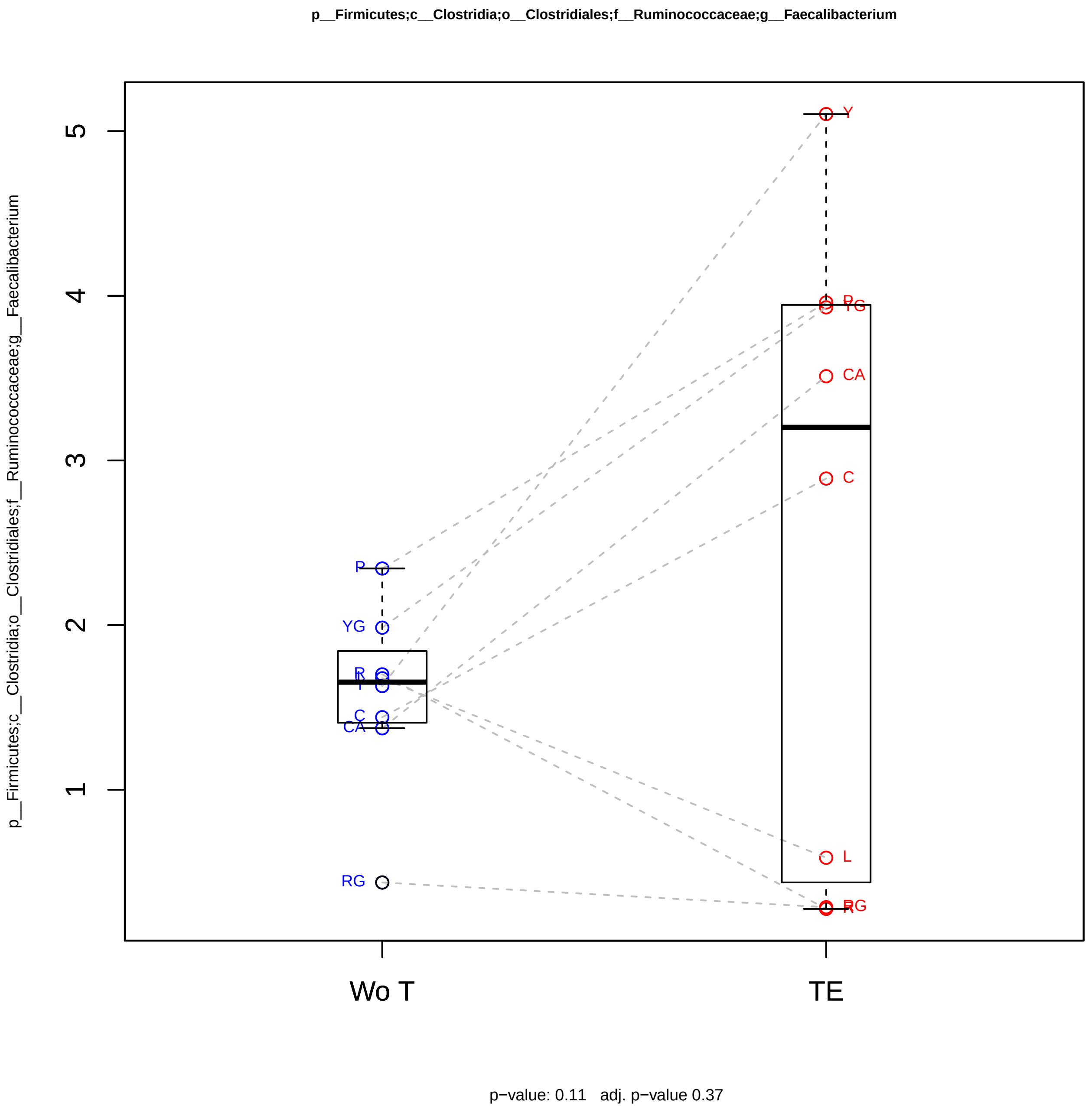

p__Firmicutes;c__Clostridia;o__Clostridiales;f__Ruminococcaceae;g__Faecalibacterium
p__Firmicutes;c__Clostridia;o__Clostridiales;f__Ruminococcaceae;g__Faecalibacterium
1
2
3
4
5
Wo T
TE
P
YG
R
T
C
CA
RG
Y
P
YG
CA
C
L
RG
R
p−value: 0.11 adj. p−value 0.37

p__Firmicutes;c__Clostridia;o__Clostridiales;f__Ruminococcaceae;g__Butyricicoccus

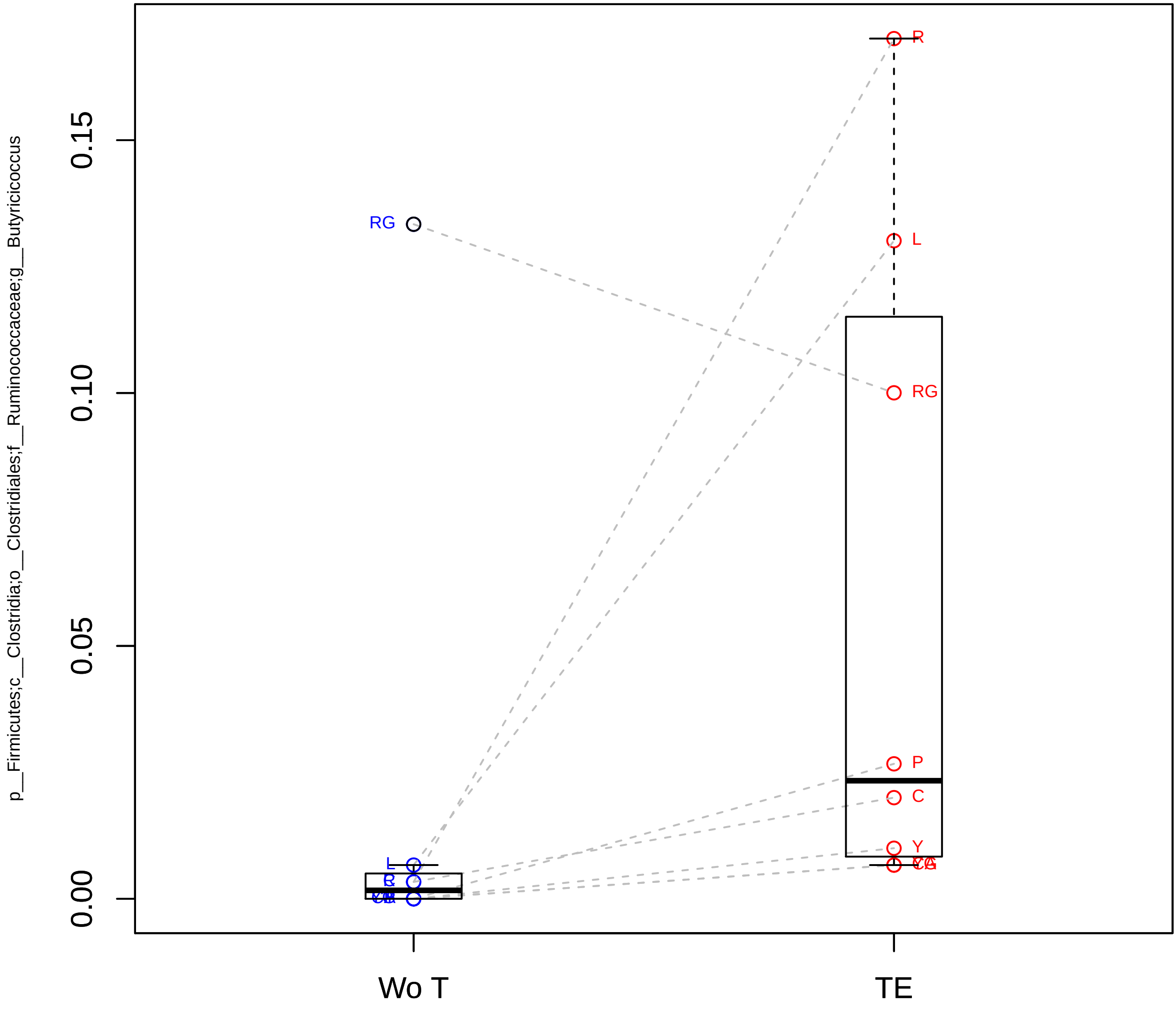

p−value: 0.11   adj. p−value 0.37

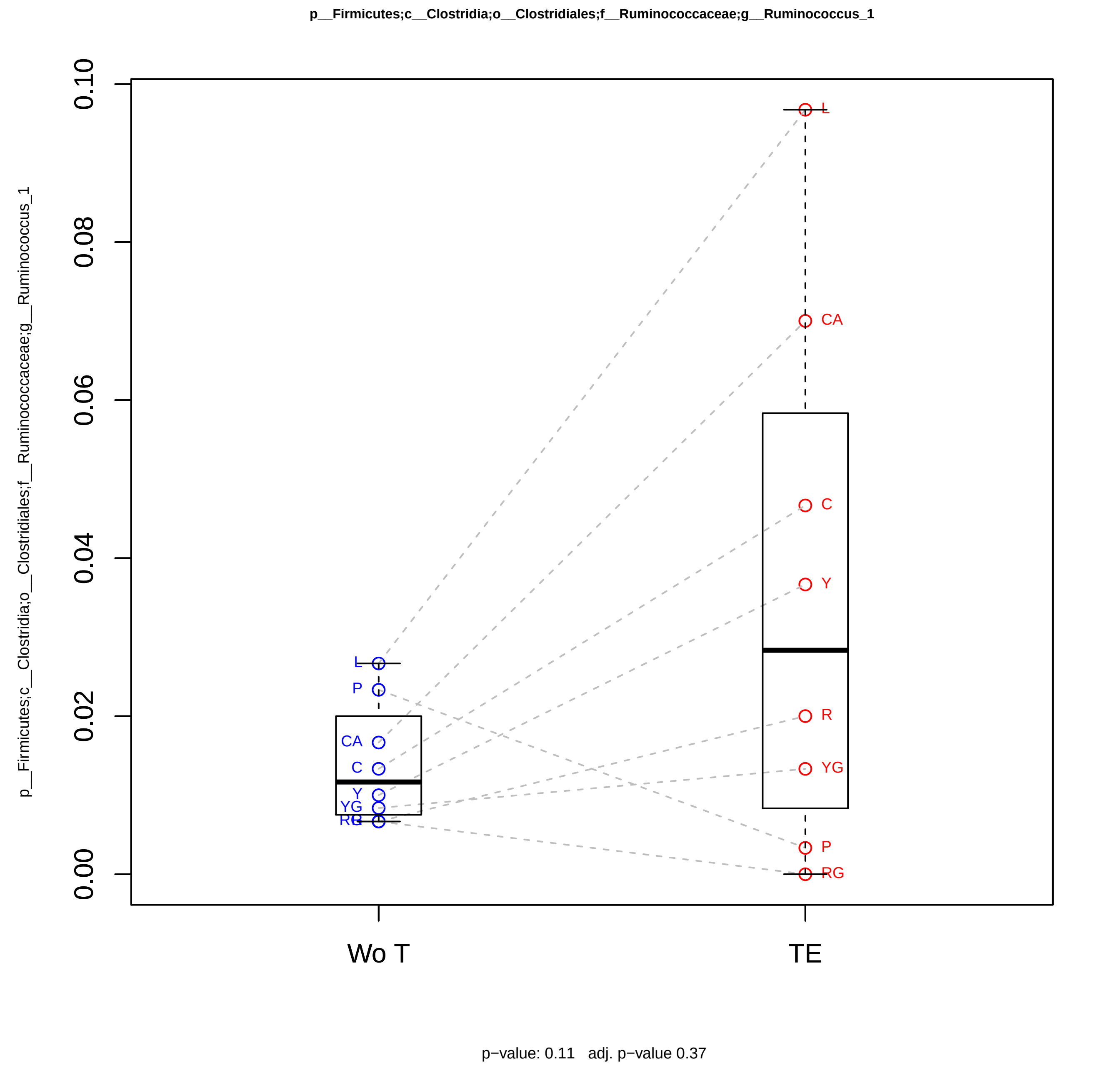
p__Firmicutes;c__Clostridia;o__Clostridiales;f__Ruminococcaceae;g__Ruminococcus_1
p__Firmicutes;c__Clostridia;o__Clostridiales;f__Ruminococcaceae;g__Ruminococcus_1
0.00
0.02
0.04
0.06
0.08
0.10
Wo T
TE
L
P
CA
C
Y
YG
R
RG
p−value: 0.11 adj. p−value 0.37

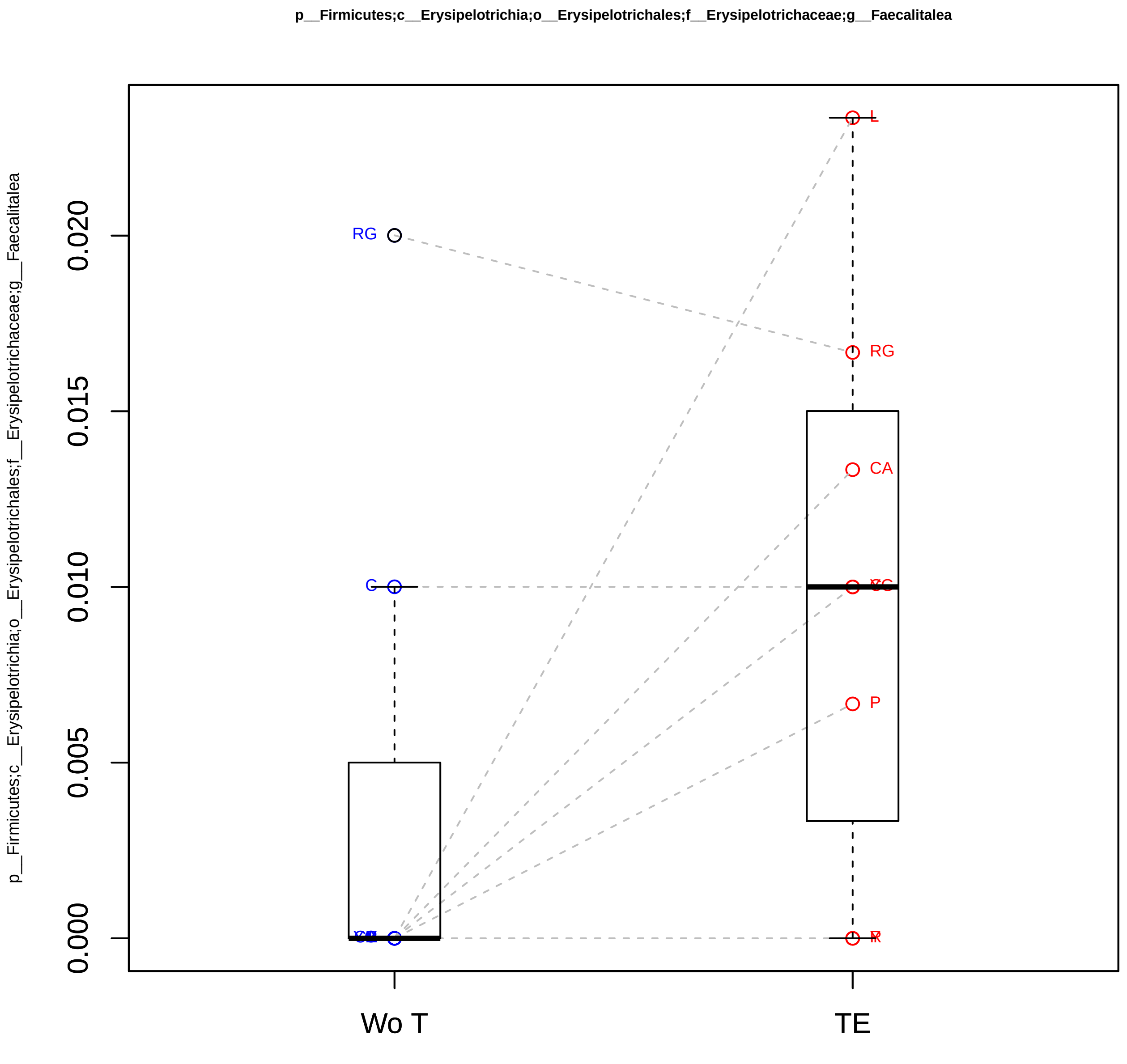

p−value: 0.14 adj. p−value 0.4

p__Firmicutes;c__Negativicutes;o__Selenomonadales;f__Acidaminococcaceae;g__Acidaminococcus

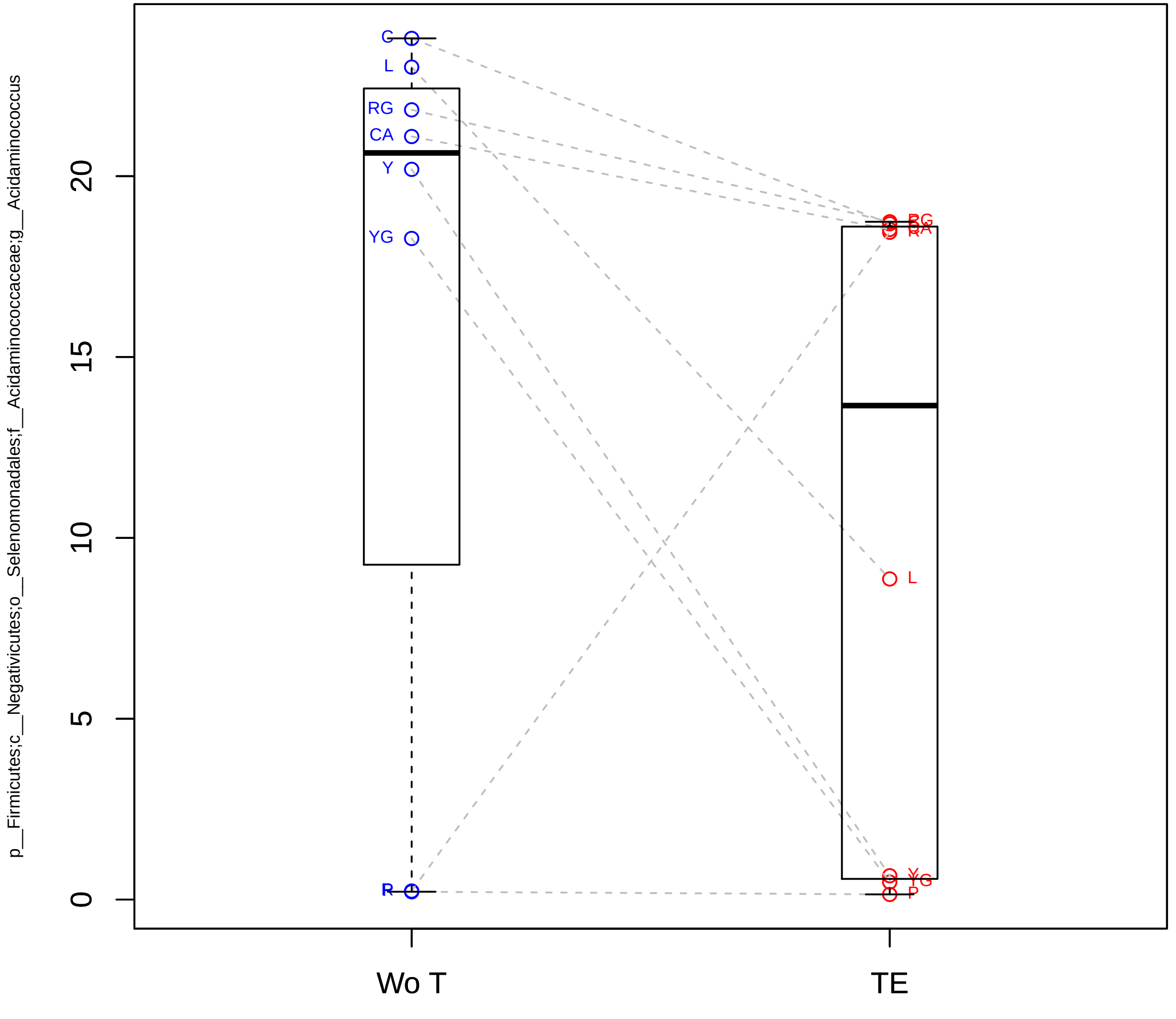

p−value: 0.15 adj. p−value 0.4

p__Firmicutes;c__Erysipelotrichia;o__Erysipelotrichales;f__Erysipelotrichaceae;g__Holdemanella

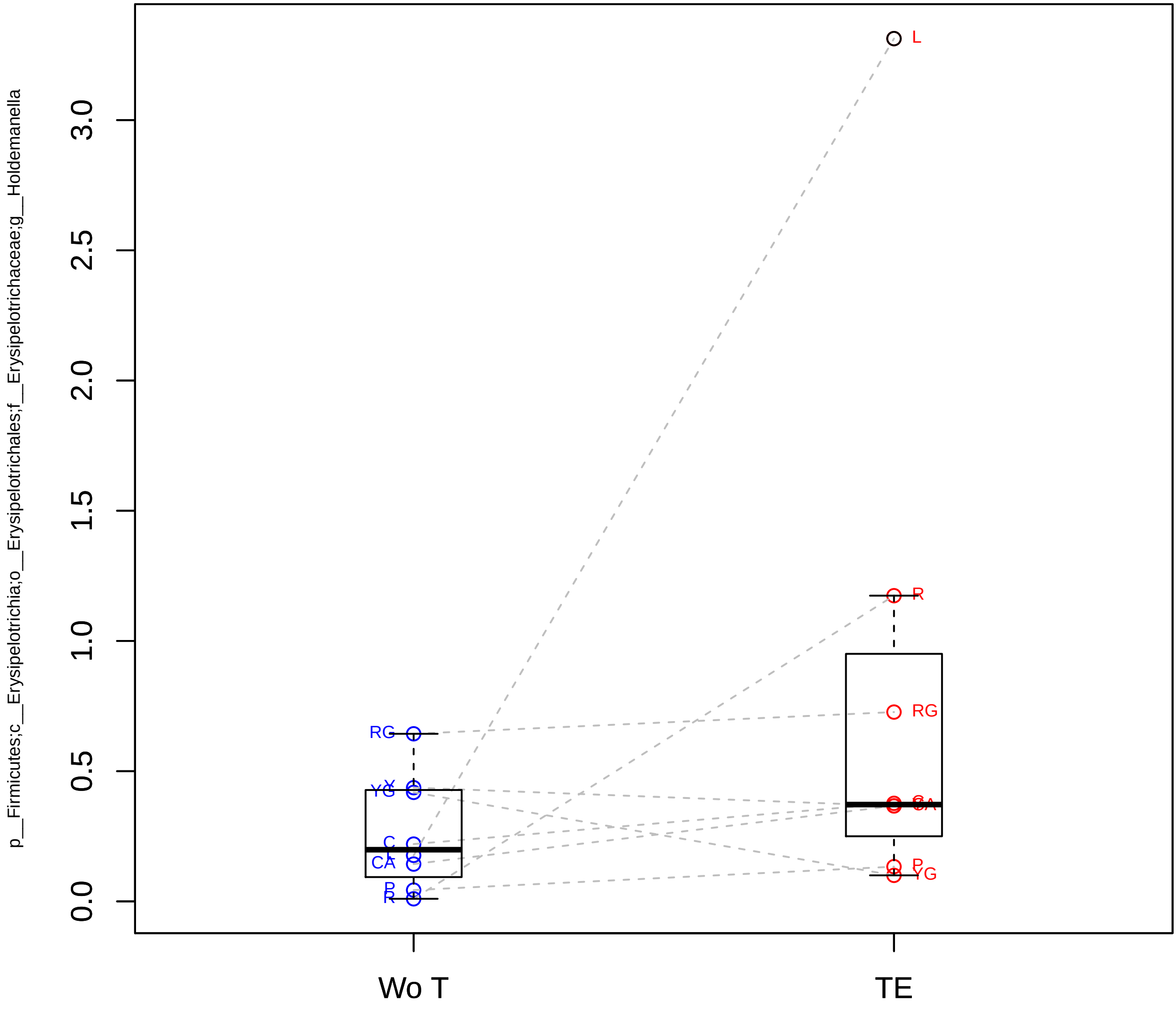

p−value: 0.15   adj. p−value 0.4

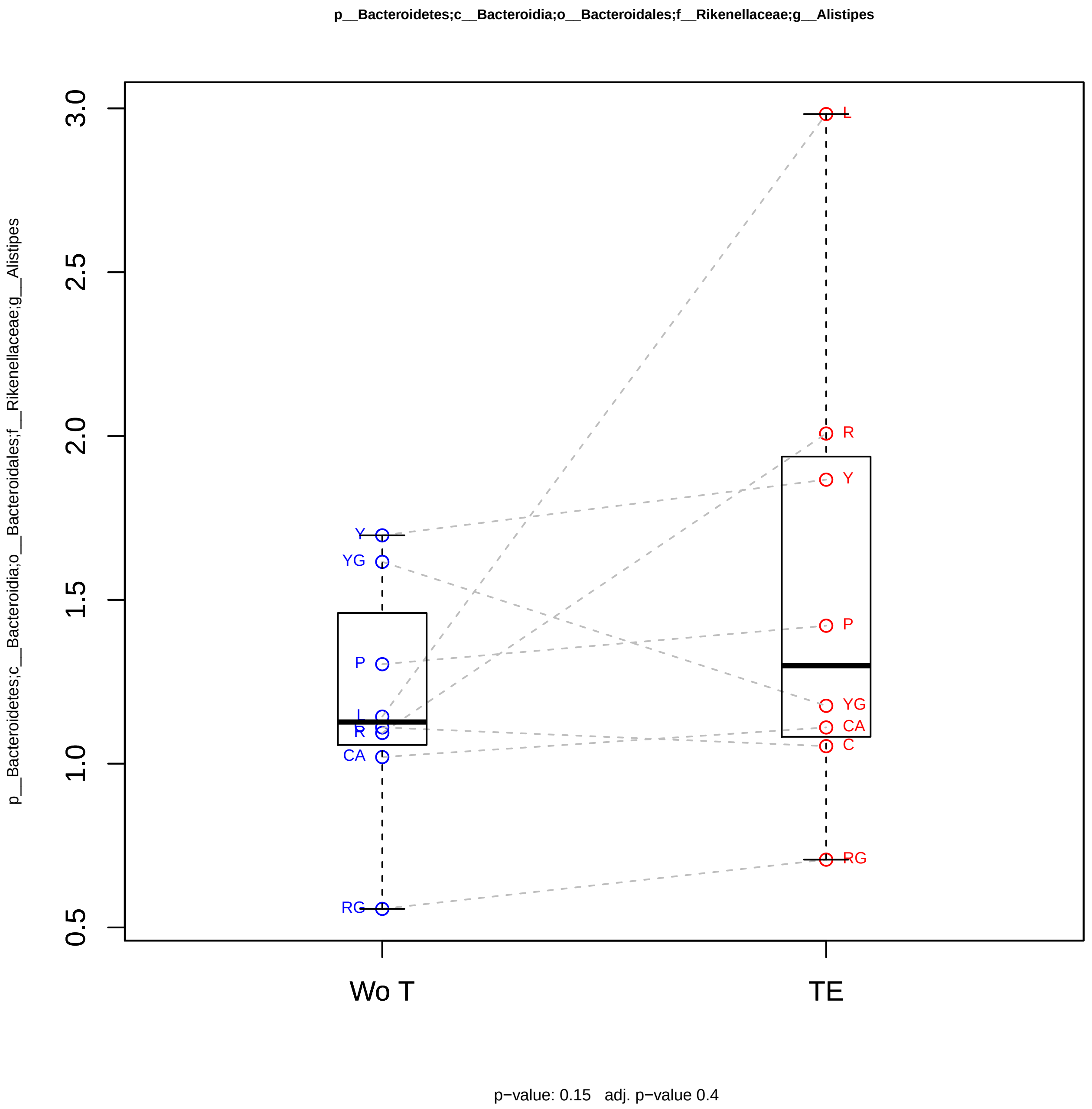

p__Bacteroidetes;c__Bacteroidia;o__Bacteroidales;f__Rikenellaceae;g__Alistipes
p__Bacteroidetes;c__Bacteroidia;o__Bacteroidales;f__Rikenellaceae;g__Alistipes
0.5
1.0
1.5
2.0
2.5
3.0
Wo T
TE
Y
YG
P
L
R
CA
RG
L
R
Y
P
YG
CA
C
RG
p−value: 0.15 adj. p−value 0.4

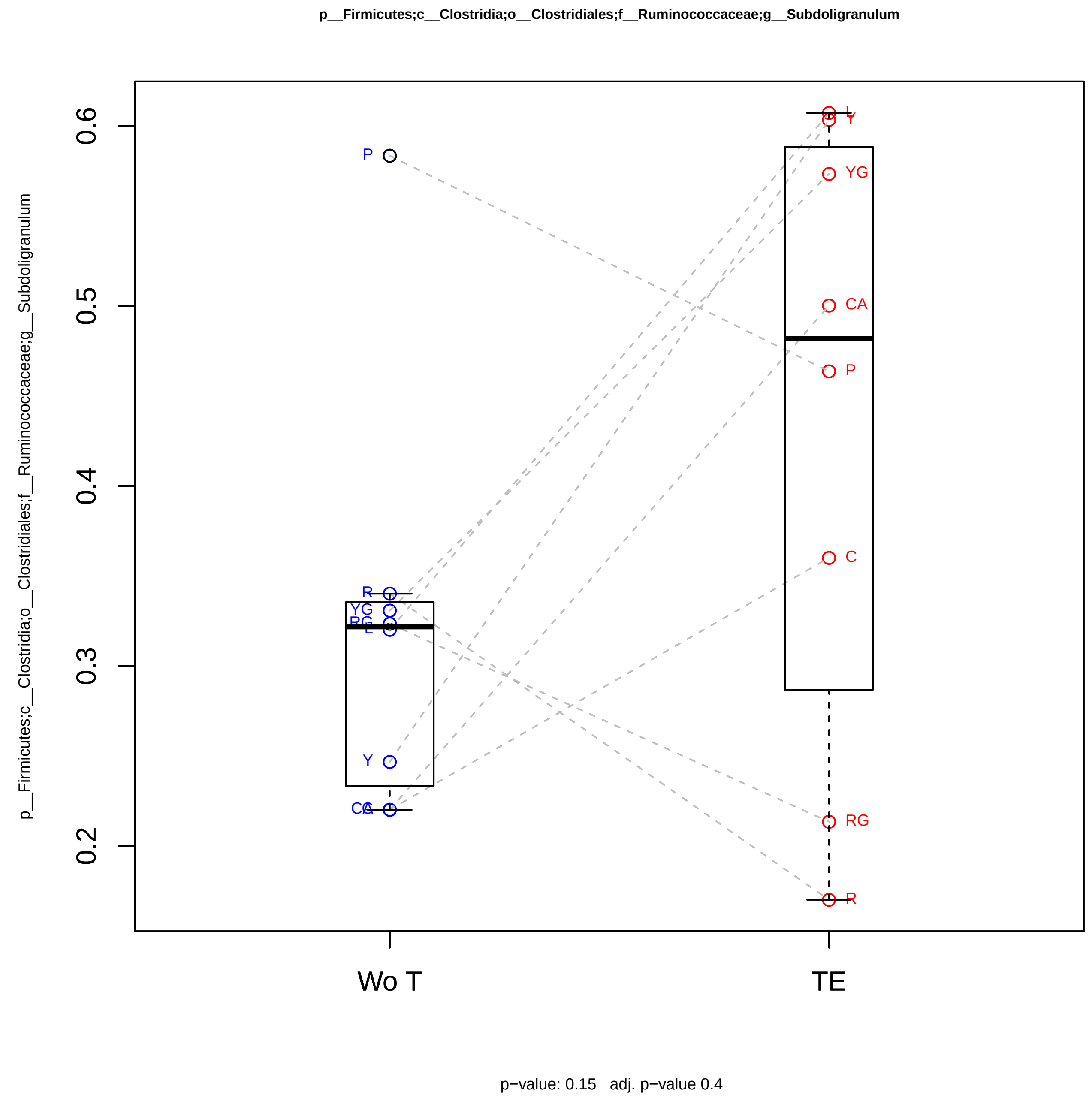

p__Firmicutes;c__Clostridia;o__Clostridiales;f__Ruminococcaceae;g__Subdoligranulum
p__Firmicutes;c__Clostridia;o__Clostridiales;f__Ruminococcaceae;g__Subdoligranulum
0.2
0.3
0.4
0.5
0.6
P
R
YG
RG
L
Y
CA
C
L
Y
YG
CA
P
C
RG
R
Wo T
TE
p−value: 0.15   adj. p−value 0.4

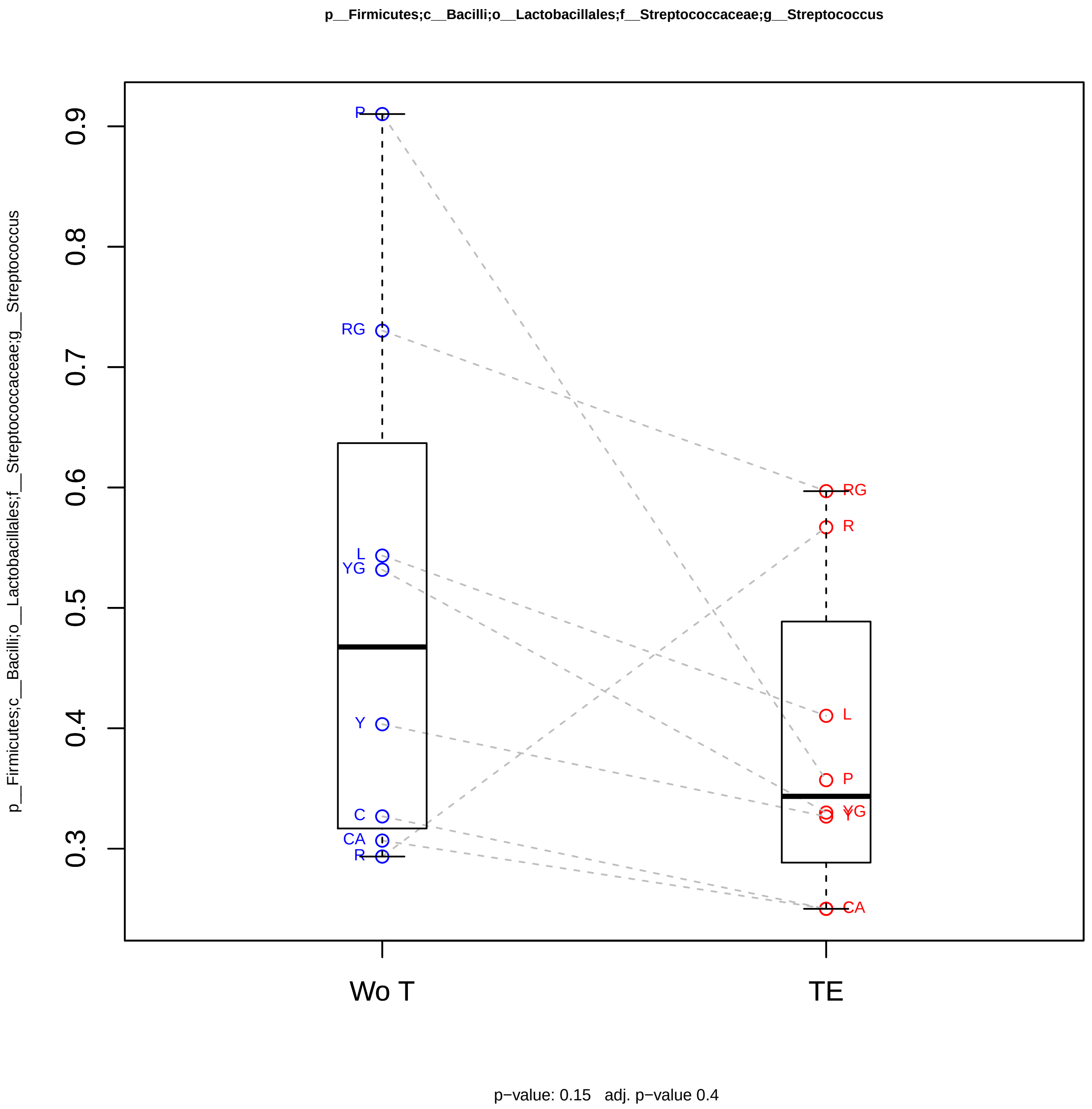
p__Firmicutes;c__Bacilli;o__Lactobacillales;f__Streptococcaceae;g__Streptococcus
p__Firmicutes;c__Bacilli;o__Lactobacillales;f__Streptococcaceae;g__Streptococcus
0.9
0.8
0.7
0.6
0.5
0.4
0.3
P
RG
L
YG
Y
C
CA
R
RG
R
L
P
YG
Y
CA
Wo T
TE
p−value: 0.15 adj. p−value 0.4

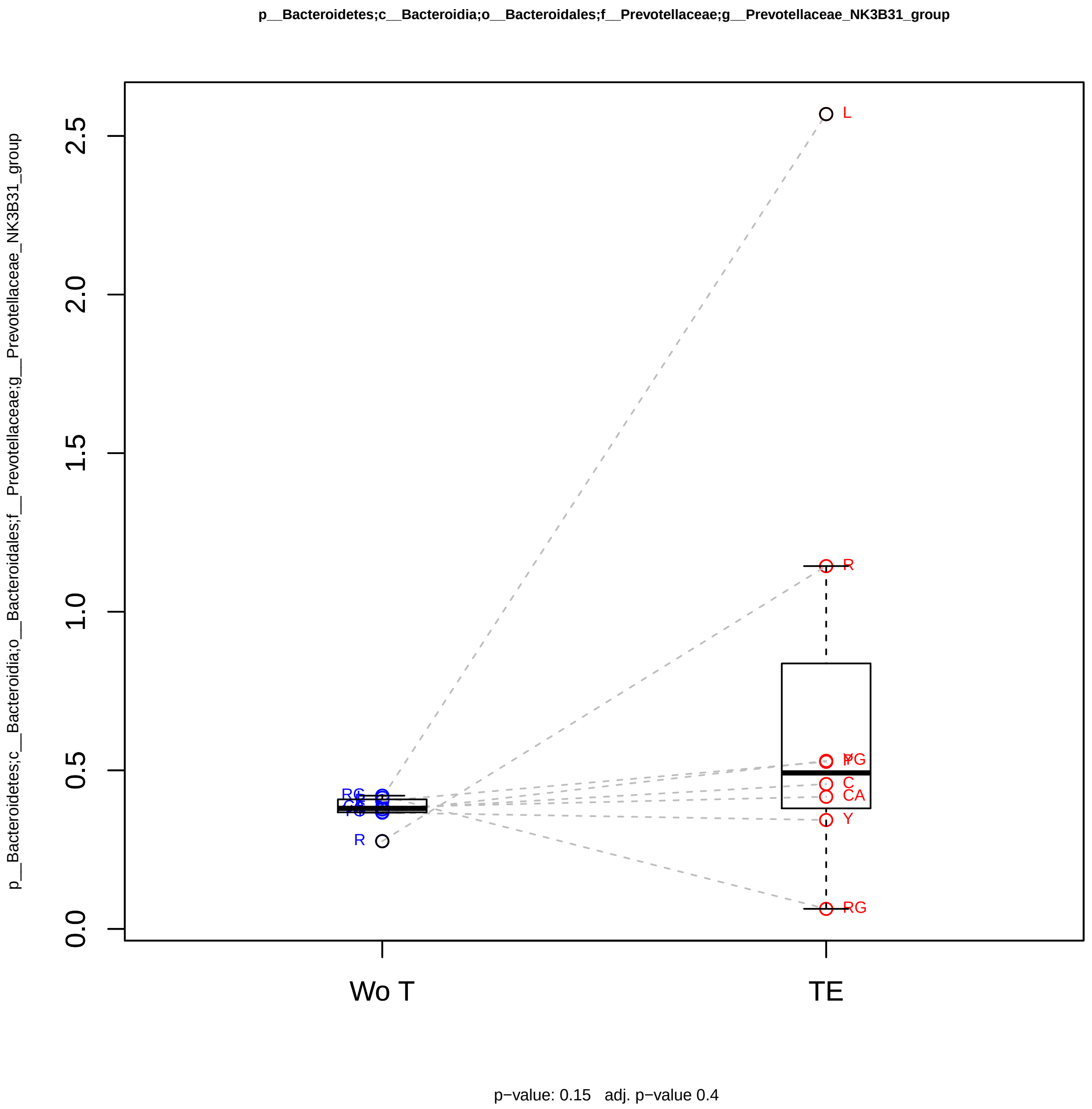
p__Bacteroidetes;c__Bacteroidia;o__Bacteroidales;f__Prevotellaceae;g__Prevotellaceae_NK3B31_group
p__Bacteroidetes;c__Bacteroidia;o__Bacteroidales;f__Prevotellaceae;g__Prevotellaceae_NK3B31_group
0.0
0.5
1.0
1.5
2.0
2.5
Wo T
TE
L
R
C
CA
Y
RG
p−value: 0.15 adj. p−value 0.4

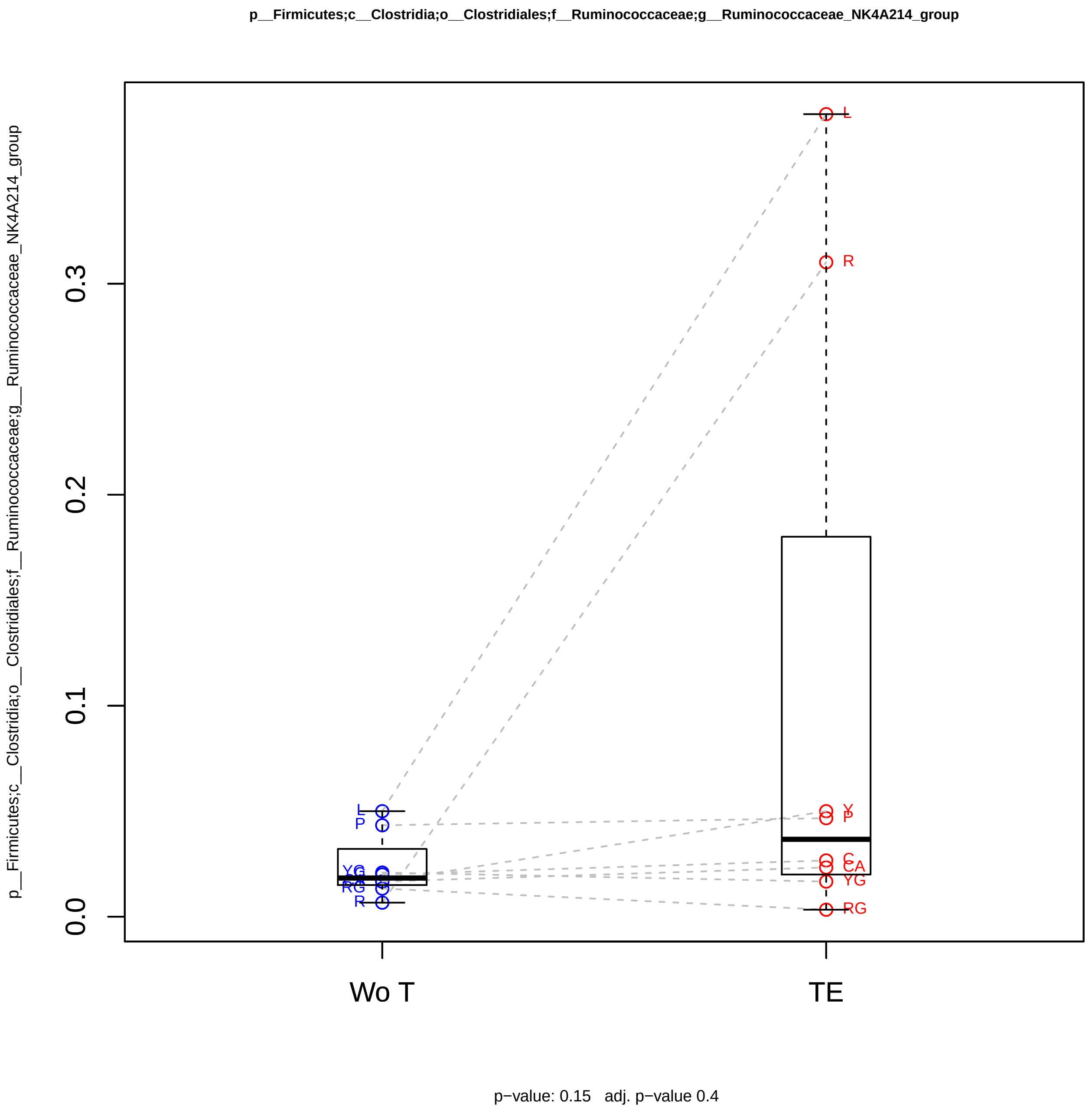

p__Firmicutes;c__Clostridia;o__Clostridiales;f__Ruminococcaceae;g__Ruminococcaceae_NK4A214_group
p__Firmicutes;c__Clostridia;o__Clostridiales;f__Ruminococcaceae;g__Ruminococcaceae_NK4A214_group
0.0
0.1
0.2
0.3
Wo T
TE
L
P
YG
RG
R
L
R
Y
P
C
CA
YG
RG
p−value: 0.15 adj. p−value 0.4

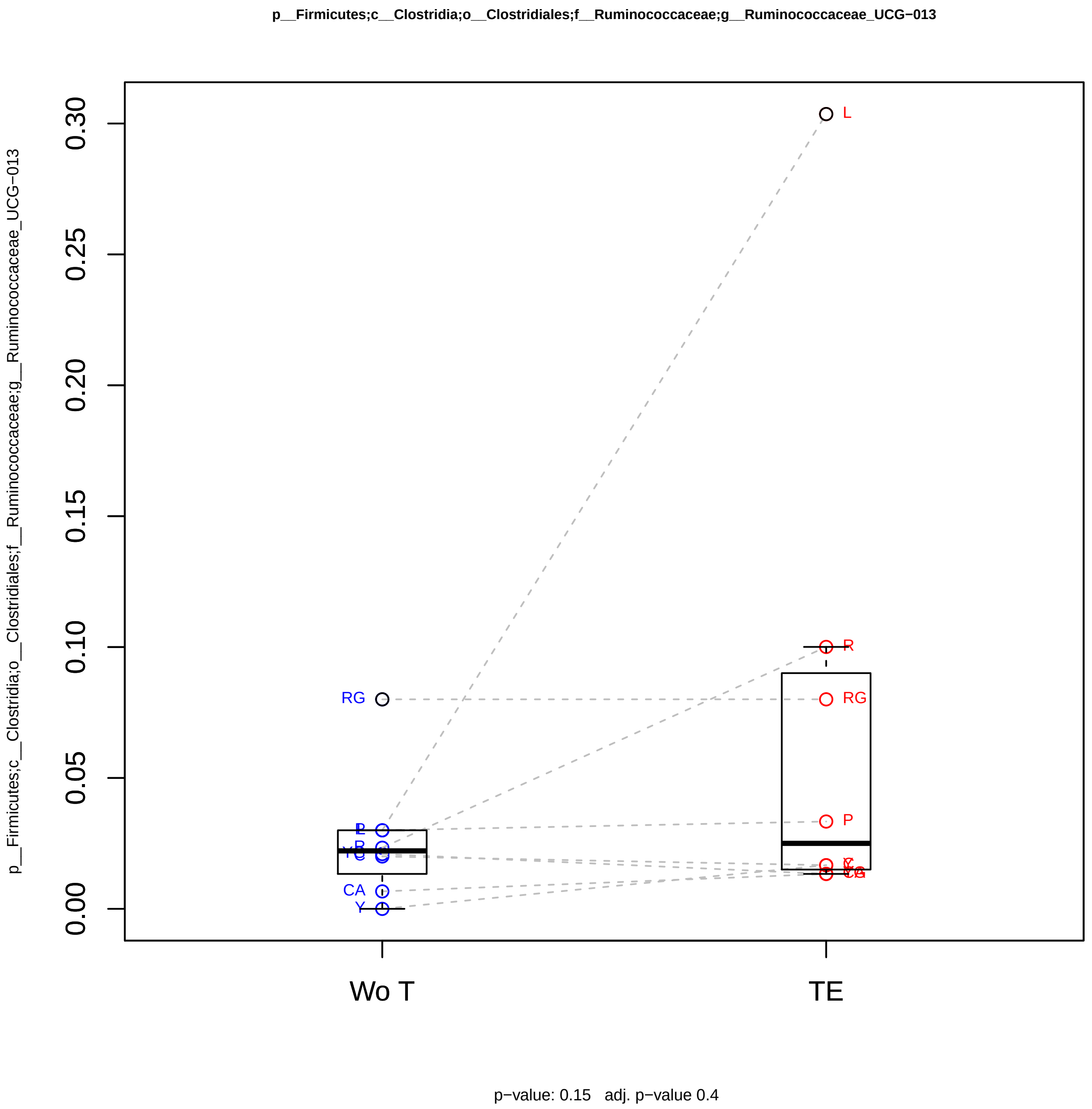

p__Firmicutes;c__Clostridia;o__Clostridiales;f__Ruminococcaceae;g__Ruminococcaceae_UCG−013
p__Firmicutes;c__Clostridia;o__Clostridiales;f__Ruminococcaceae;g__Ruminococcaceae_UCG−013
0.00
0.05
0.10
0.15
0.20
0.25
0.30
Wo T
TE
RG
CA
Y
L
R
P
p−value: 0.15 adj. p−value 0.4

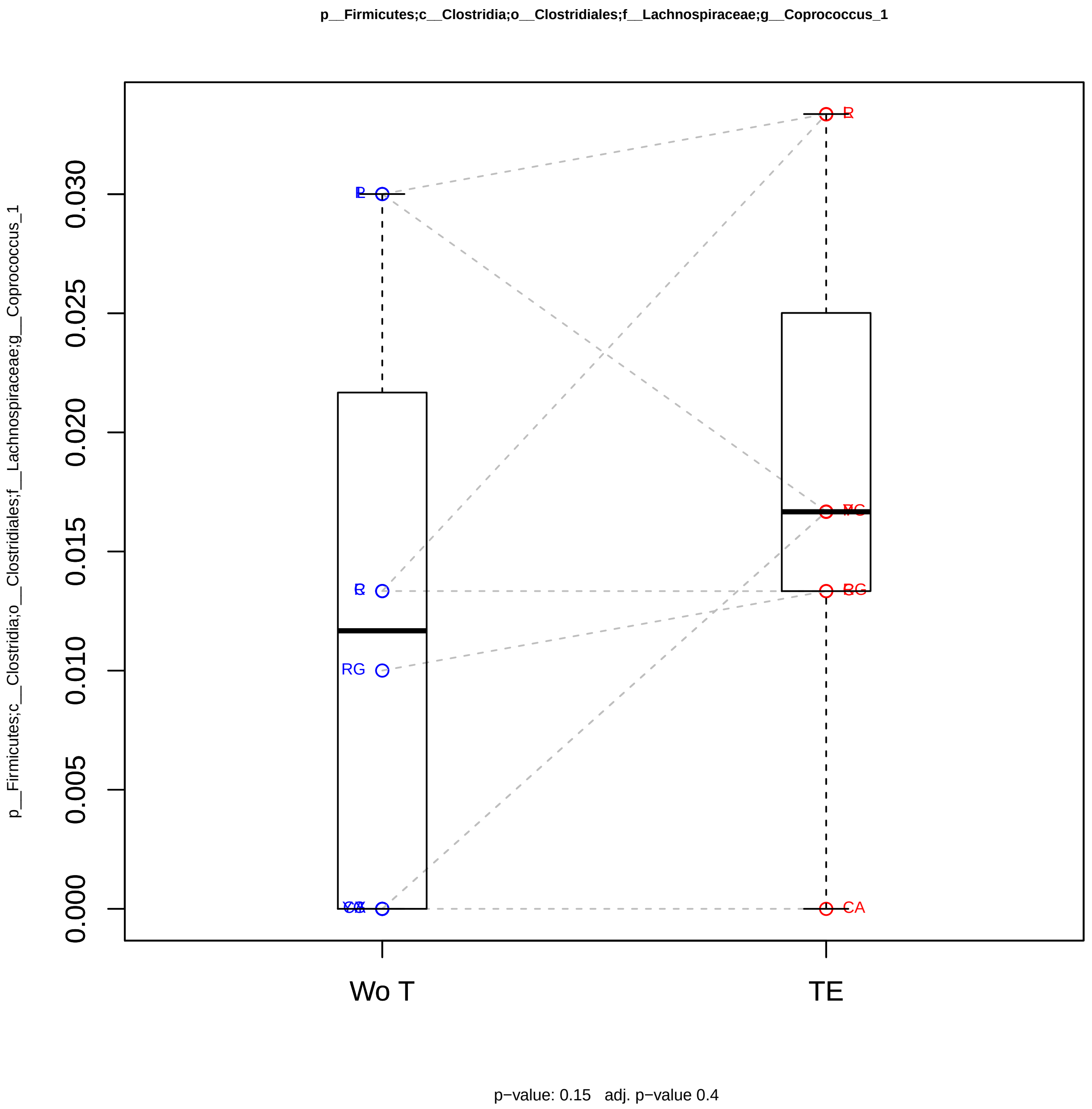
p__Firmicutes;c__Clostridia;o__Clostridiales;f__Lachnospiraceae;g__Coprococcus_1
p__Firmicutes;c__Clostridia;o__Clostridiales;f__Lachnospiraceae;g__Coprococcus_1
0.000
0.005
0.010
0.015
0.020
0.025
0.030
RG
CA
Wo T
TE
p−value: 0.15 adj. p−value 0.4

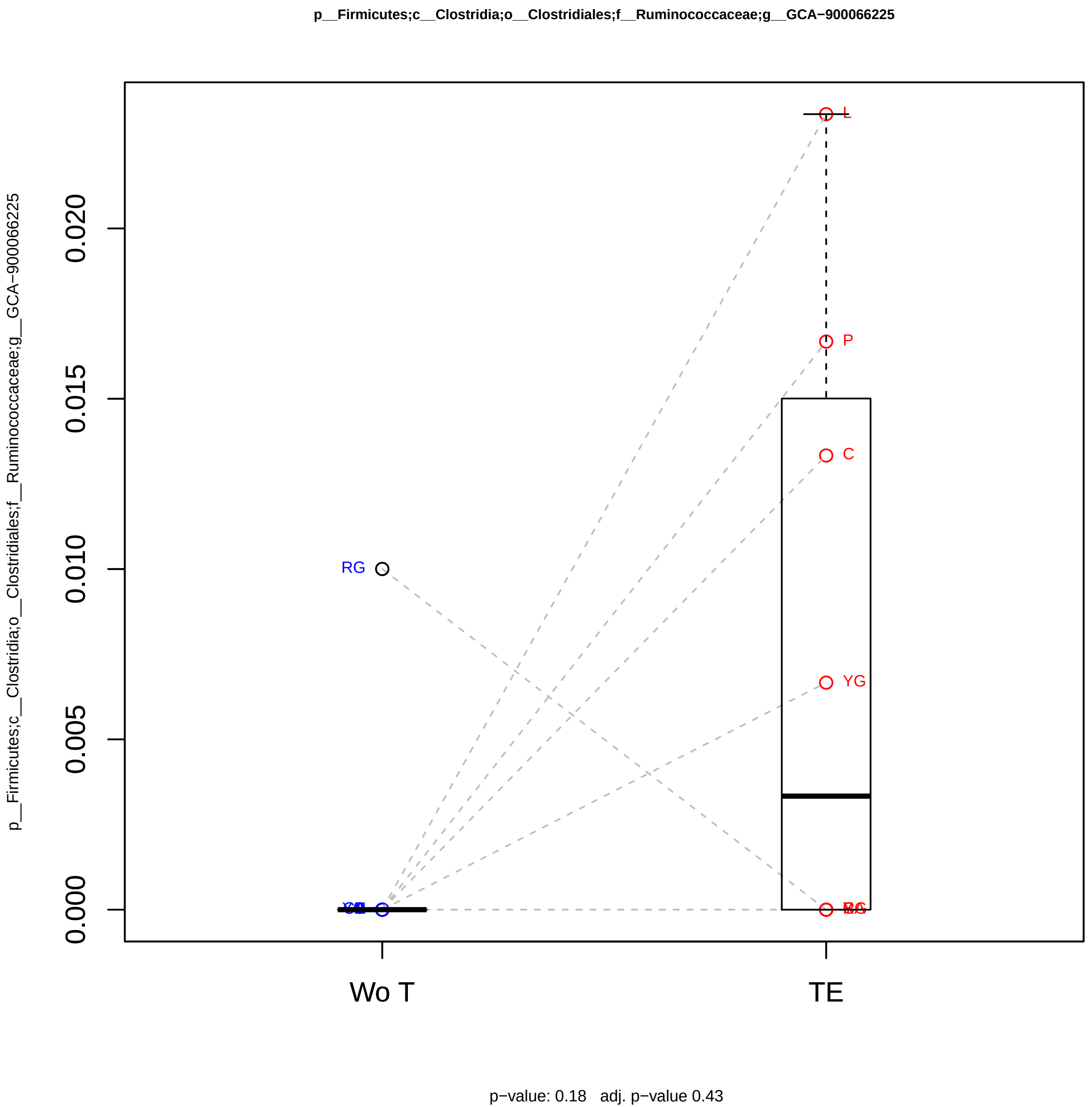
p__Firmicutes;c__Clostridia;o__Clostridiales;f__Ruminococcaceae;g__GCA−900066225
p__Firmicutes;c__Clostridia;o__Clostridiales;f__Ruminococcaceae;g__GCA−900066225
0.000
0.005
0.010
0.015
0.020
Wo T
TE
RG
L
P
C
YG
p−value: 0.18   adj. p−value 0.43

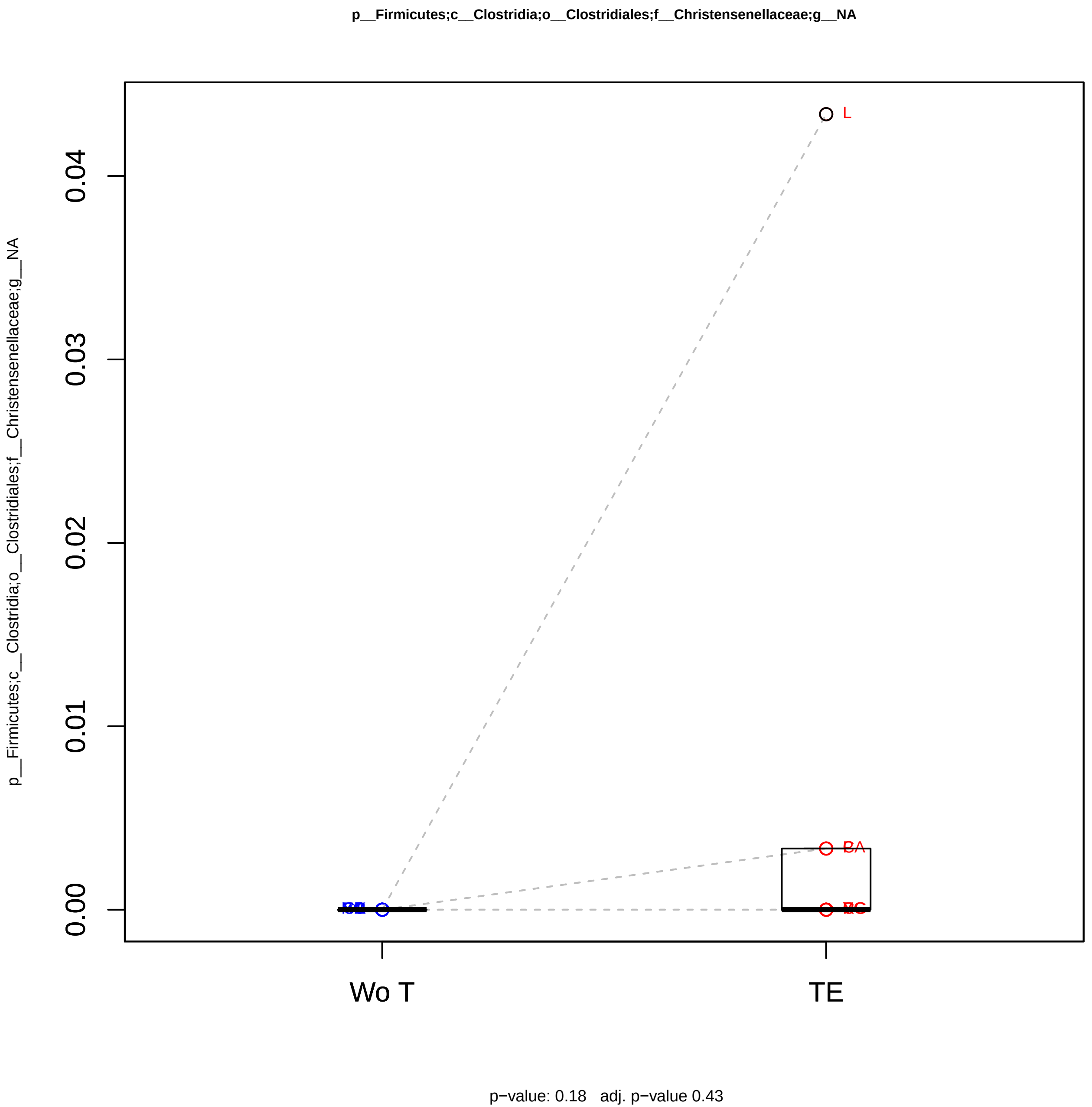
p__Firmicutes;c__Clostridia;o__Clostridiales;f__Christensenellaceae;g__NA
p__Firmicutes;c__Clostridia;o__Clostridiales;f__Christensenellaceae;g__NA
0.04
0.03
0.02
0.01
0.00
L
Wo T
TE
p−value: 0.18   adj. p−value 0.43

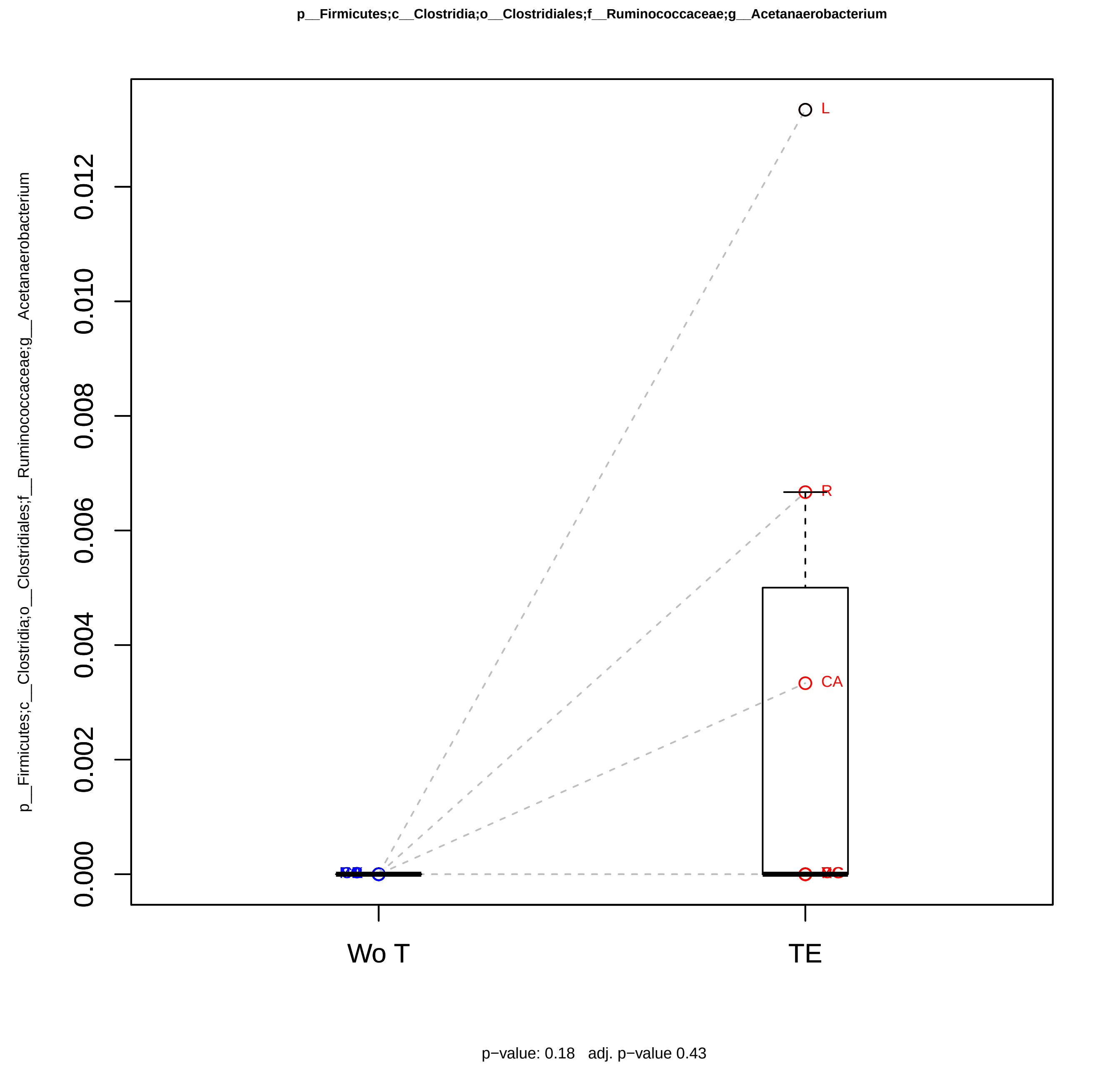

p__Firmicutes;c__Clostridia;o__Clostridiales;f__Ruminococcaceae;g__Acetanaerobacterium
p__Firmicutes;c__Clostridia;o__Clostridiales;f__Ruminococcaceae;g__Acetanaerobacterium
0.000
0.002
0.004
0.006
0.008
0.010
0.012
L
R
CA
Wo T
TE
p−value: 0.18 adj. p−value 0.43

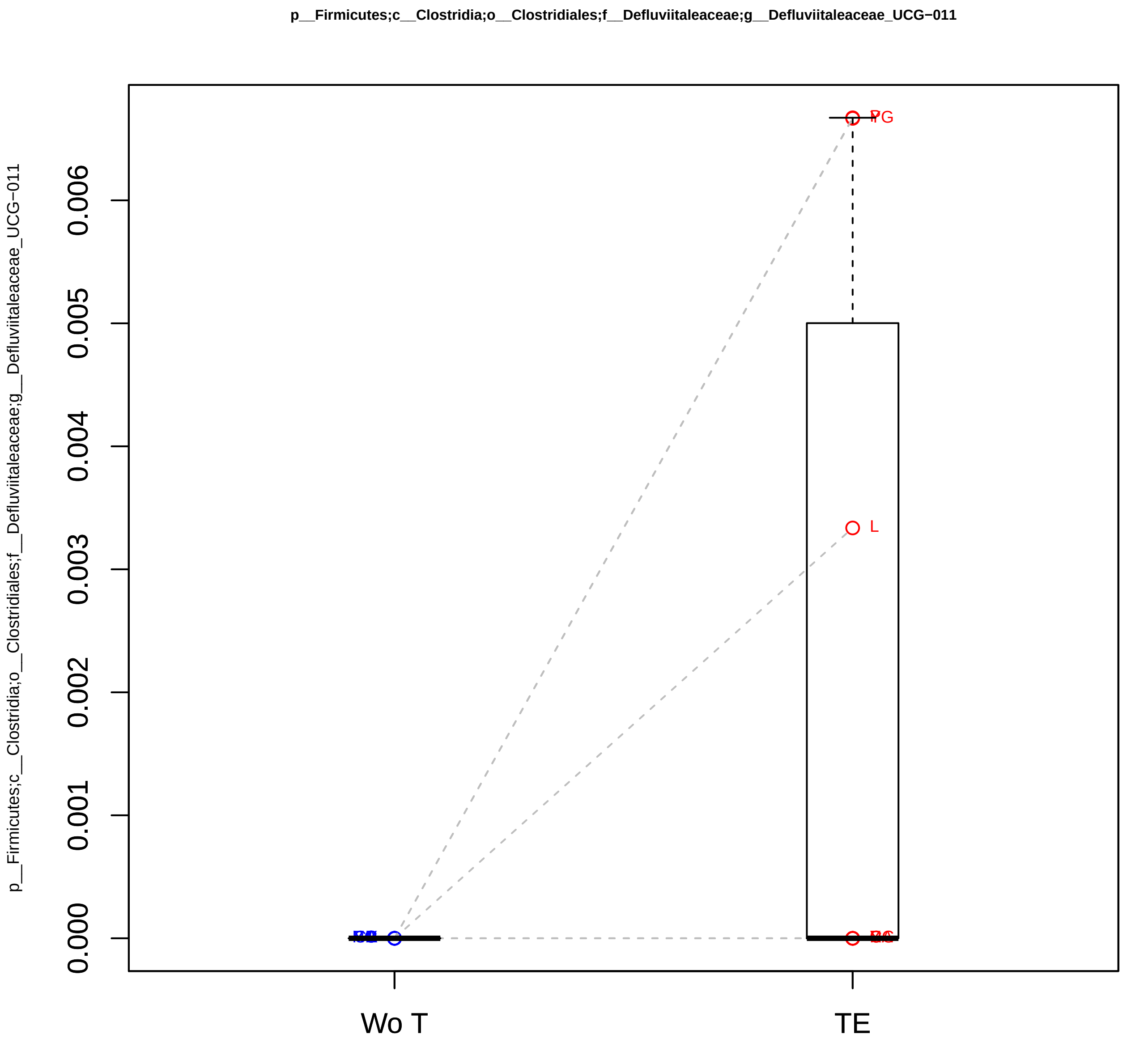

p−value: 0.18   adj. p−value 0.43

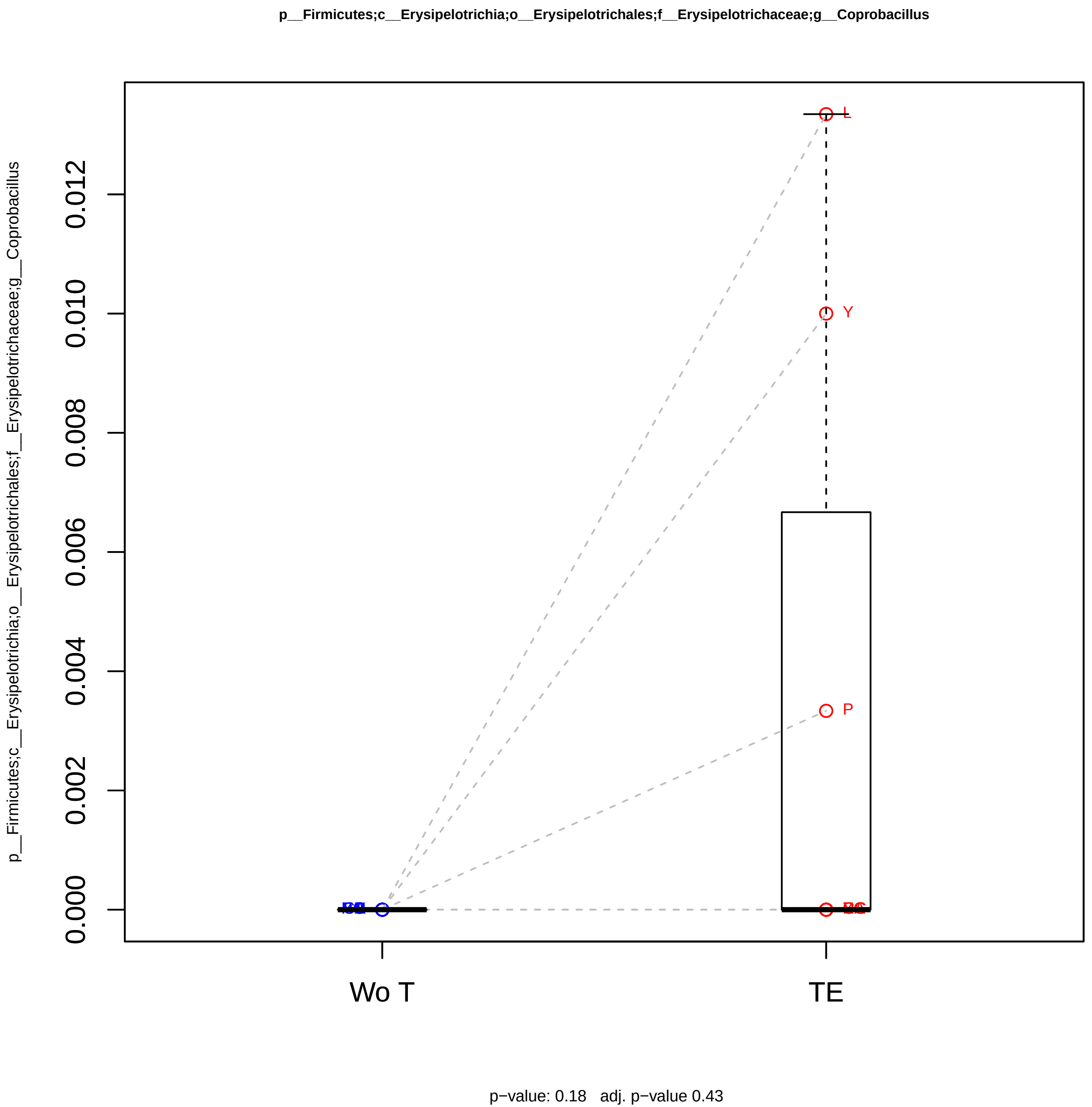

p__Firmicutes;c__Erysipelotrichia;o__Erysipelotrichales;f__Erysipelotrichaceae;g__Coprobacillus
p__Firmicutes;c__Erysipelotrichia;o__Erysipelotrichales;f__Erysipelotrichaceae;g__Coprobacillus
0.000
0.002
0.004
0.006
0.008
0.010
0.012
L
Y
P
Wo T
TE
p−value: 0.18 adj. p−value 0.43

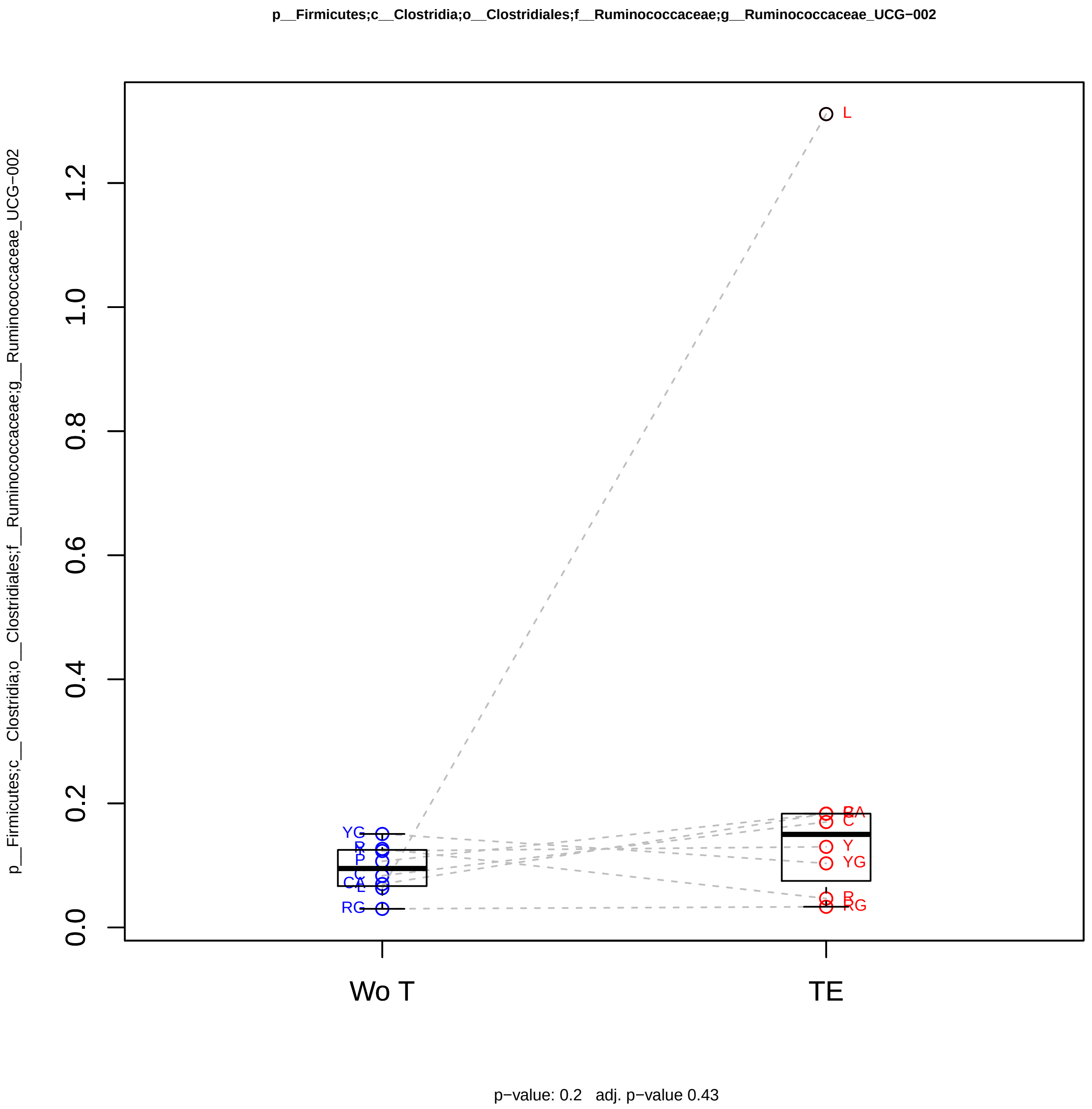

p__Firmicutes;c__Clostridia;o__Clostridiales;f__Ruminococcaceae;g__Ruminococcaceae_UCG−002
p__Firmicutes;c__Clostridia;o__Clostridiales;f__Ruminococcaceae;g__Ruminococcaceae_UCG−002
0.0
0.2
0.4
0.6
0.8
1.0
1.2
Wo T
TE
L
YG
P
C
RG
Y
p−value: 0.2 adj. p−value 0.43

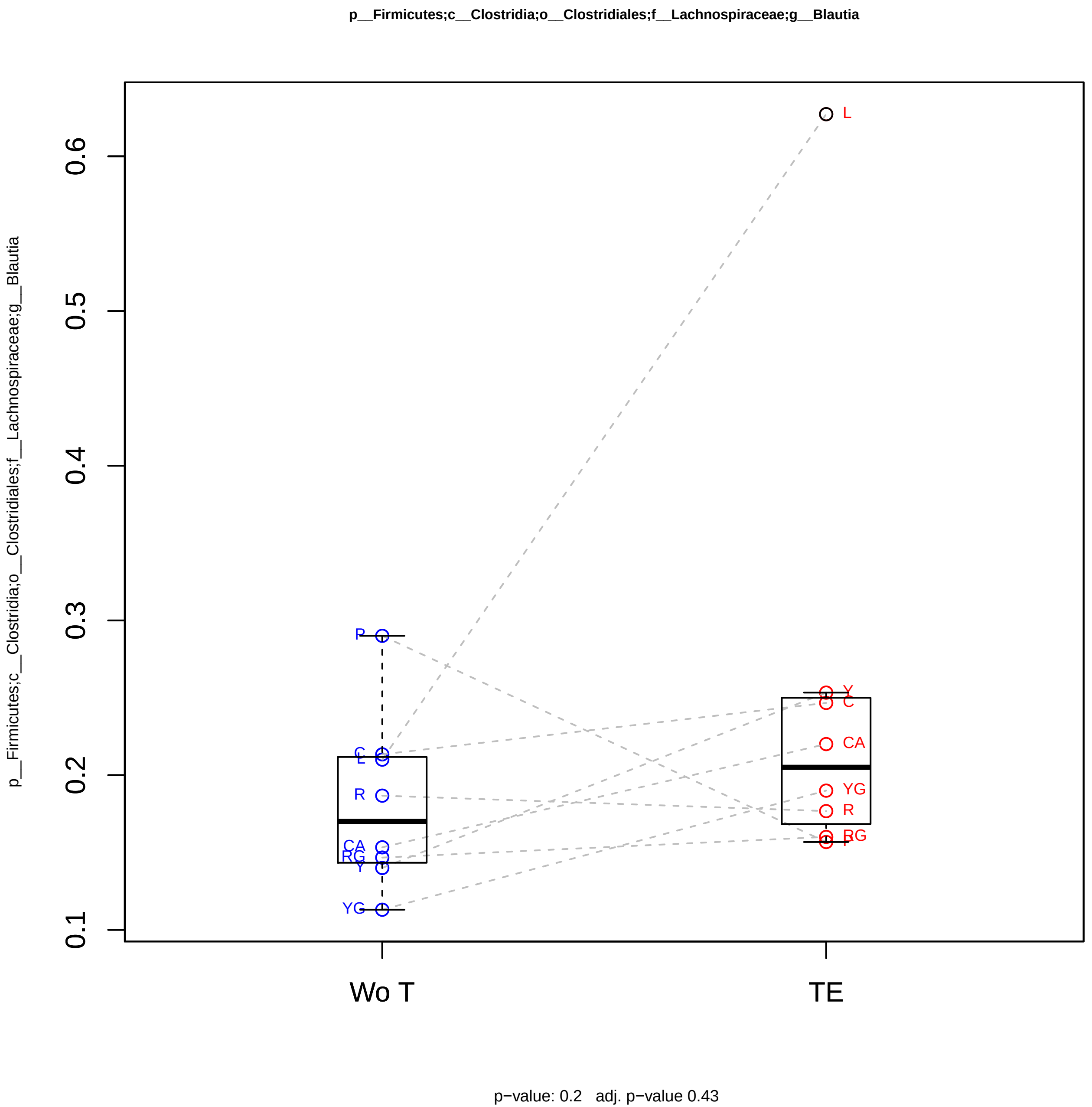
p__Firmicutes;c__Clostridia;o__Clostridiales;f__Lachnospiraceae;g__Blautia
p__Firmicutes;c__Clostridia;o__Clostridiales;f__Lachnospiraceae;g__Blautia
0.1
0.2
0.3
0.4
0.5
0.6
Wo T
TE
P
C
L
R
CA
RG
Y
YG
L
Y
C
CA
YG
R
RG
P
p−value: 0.2 adj. p−value 0.43

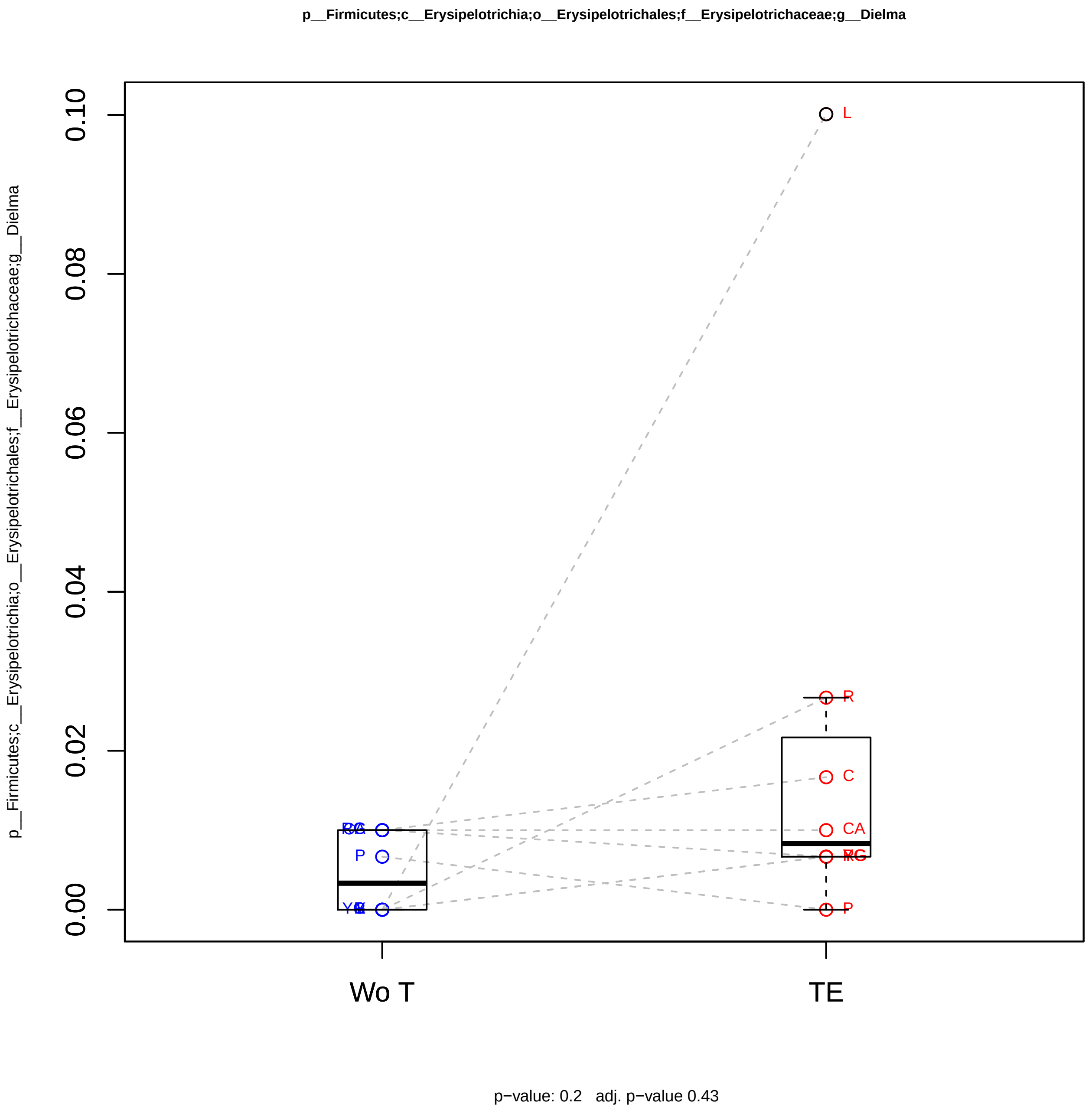
p__Firmicutes;c__Erysipelotrichia;o__Erysipelotrichales;f__Erysipelotrichaceae;g__Dielma
p__Firmicutes;c__Erysipelotrichia;o__Erysipelotrichales;f__Erysipelotrichaceae;g__Dielma
0.00
0.02
0.04
0.06
0.08
0.10
Wo T
TE
P
L
R
C
CA
P
p−value: 0.2 adj. p−value 0.43

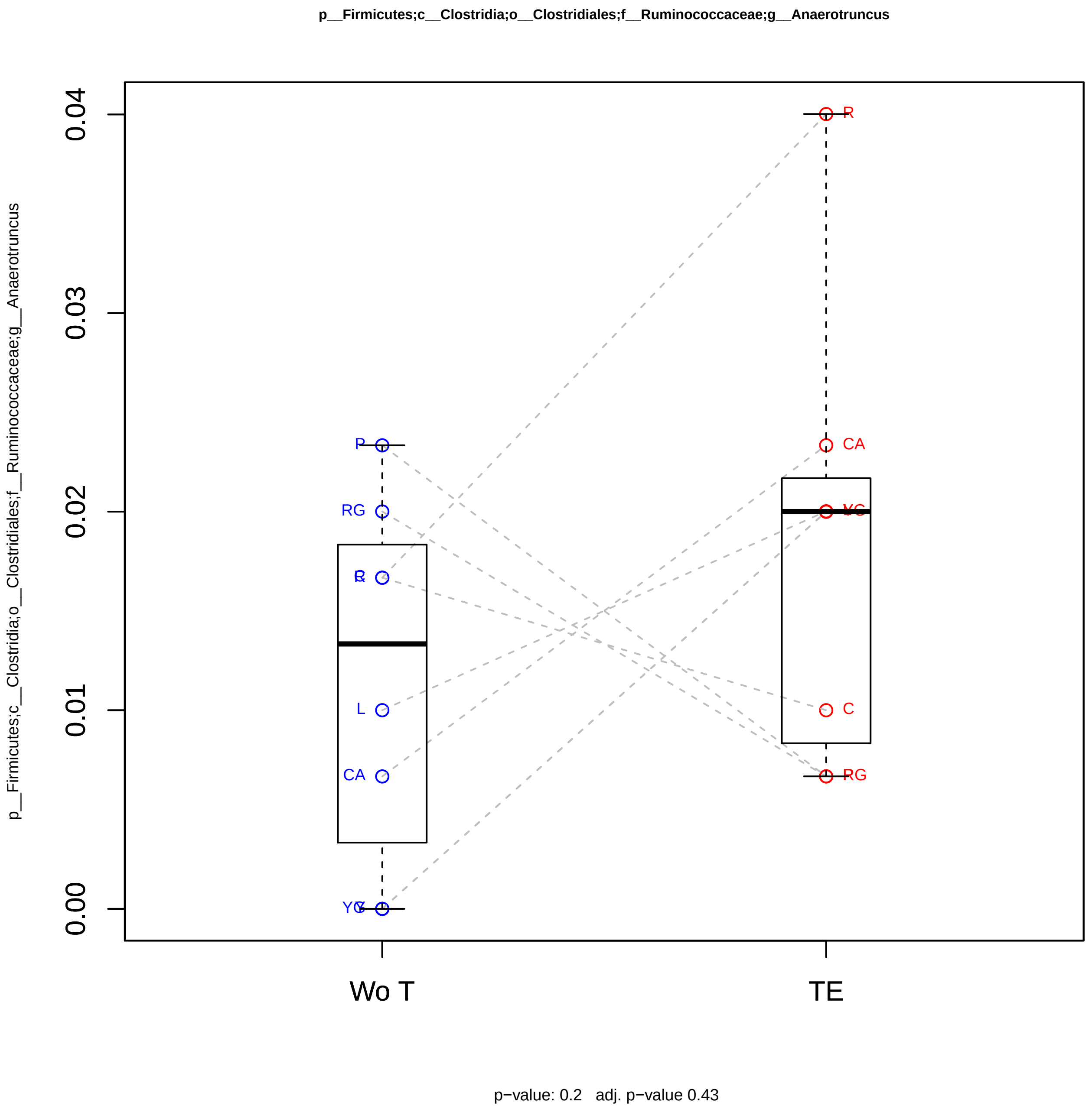
p__Firmicutes;c__Clostridia;o__Clostridiales;f__Ruminococcaceae;g__Anaerotruncus
p__Firmicutes;c__Clostridia;o__Clostridiales;f__Ruminococcaceae;g__Anaerotruncus
0.00
0.01
0.02
0.03
0.04
Wo T
TE
P
RG
L
CA
R
CA
C
RG
p−value: 0.2 adj. p−value 0.43

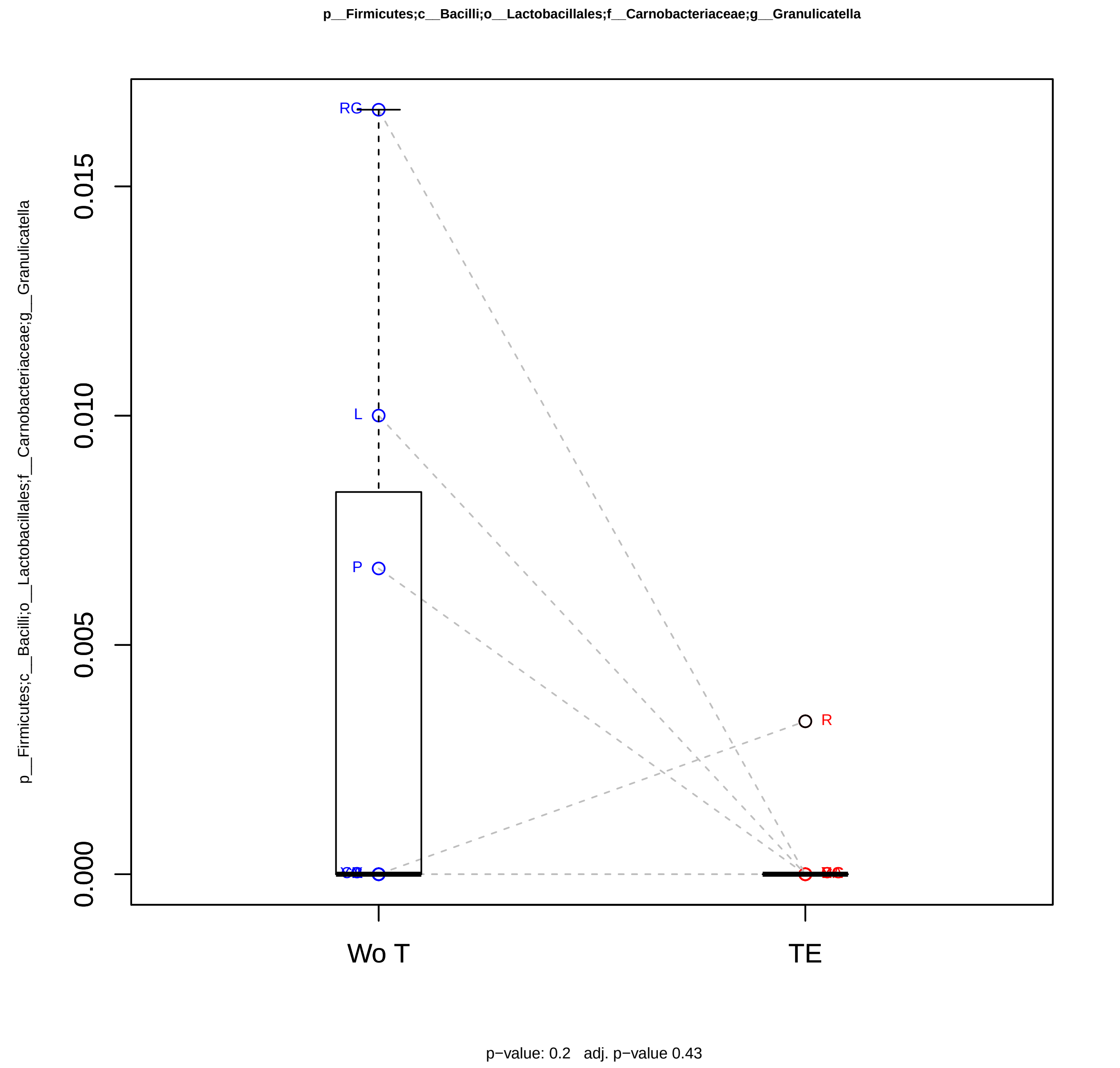

p__Firmicutes;c__Bacilli;o__Lactobacillales;f__Carnobacteriaceae;g__Granulicatella
p__Firmicutes;c__Bacilli;o__Lactobacillales;f__Carnobacteriaceae;g__Granulicatella
0.000
0.005
0.010
0.015
RG
L
P
R
Wo T
TE
p−value: 0.2 adj. p−value 0.43

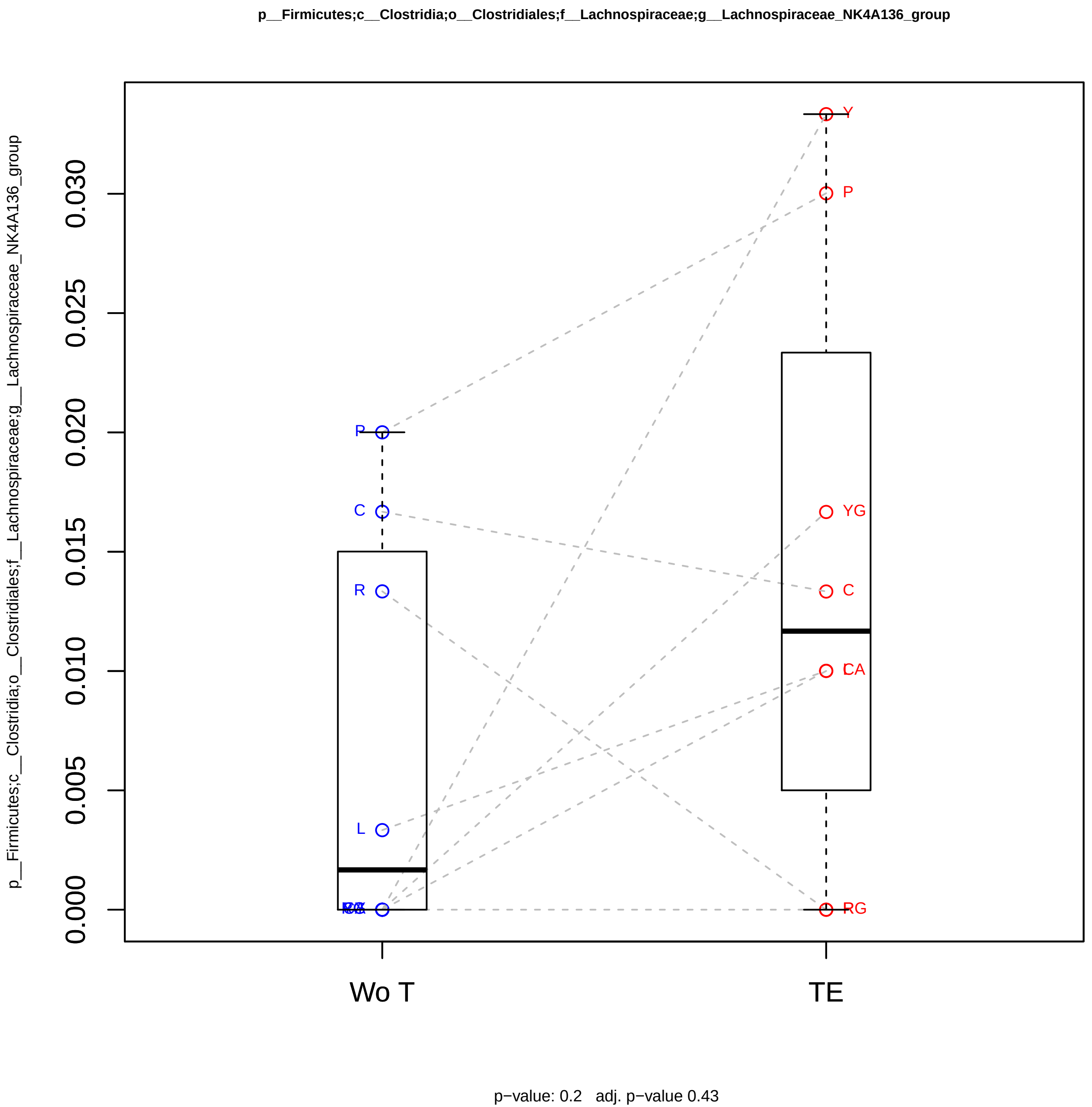

p__Firmicutes;c__Clostridia;o__Clostridiales;f__Lachnospiraceae;g__Lachnospiraceae_NK4A136_group
p__Firmicutes;c__Clostridia;o__Clostridiales;f__Lachnospiraceae;g__Lachnospiraceae_NK4A136_group
0.000
0.005
0.010
0.015
0.020
0.025
0.030
Wo T
TE
P
C
R
L
Y
P
YG
C
CA
RG
p−value: 0.2 adj. p−value 0.43

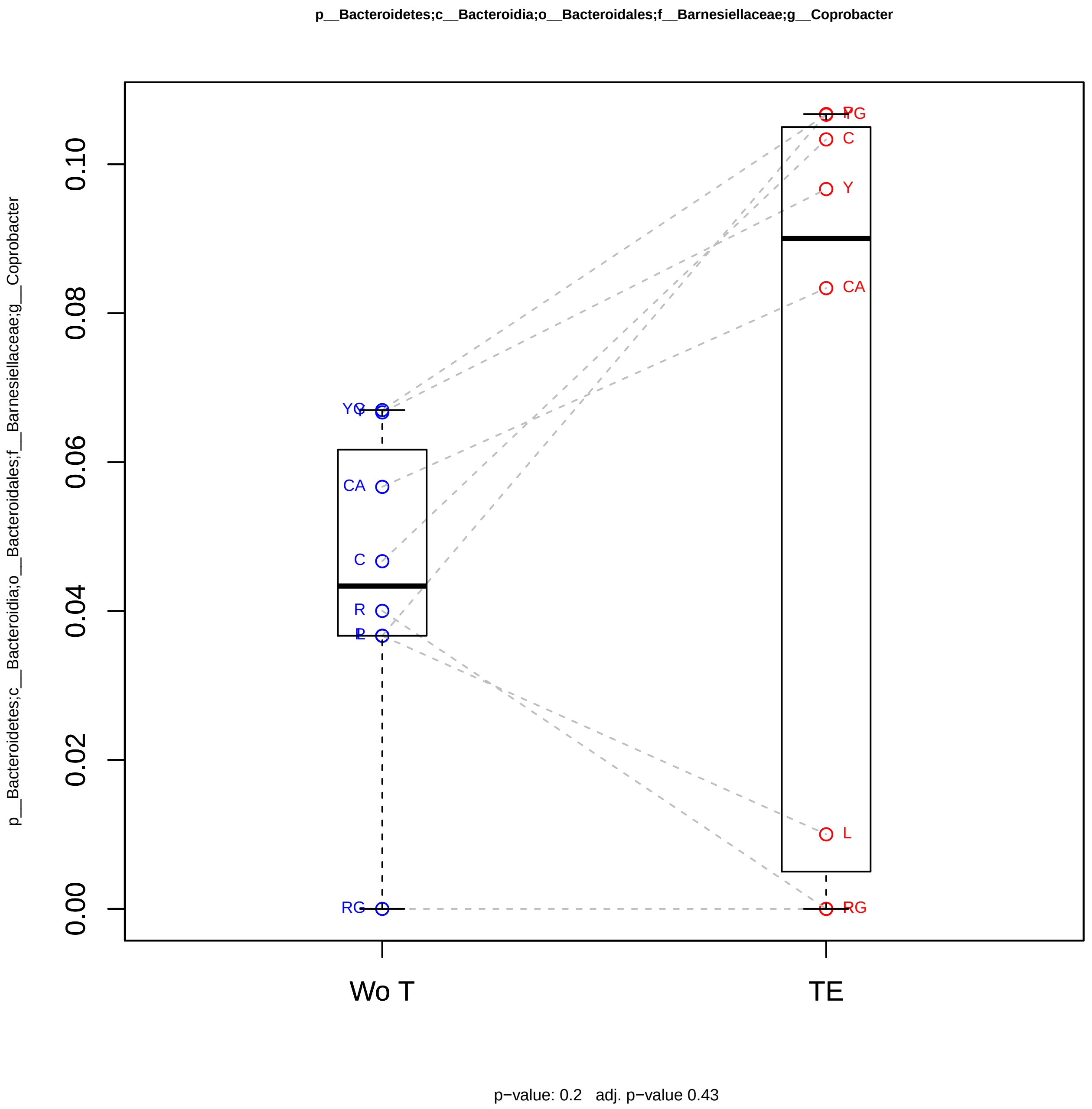
p__Bacteroidetes;c__Bacteroidia;o__Bacteroidales;f__Barnesiellaceae;g__Coprobacter
p__Bacteroidetes;c__Bacteroidia;o__Bacteroidales;f__Barnesiellaceae;g__Coprobacter
0.00
0.02
0.04
0.06
0.08
0.10
Wo T
TE
YC
CA
C
R
RG
PG
Y
L
p−value: 0.2 adj. p−value 0.43

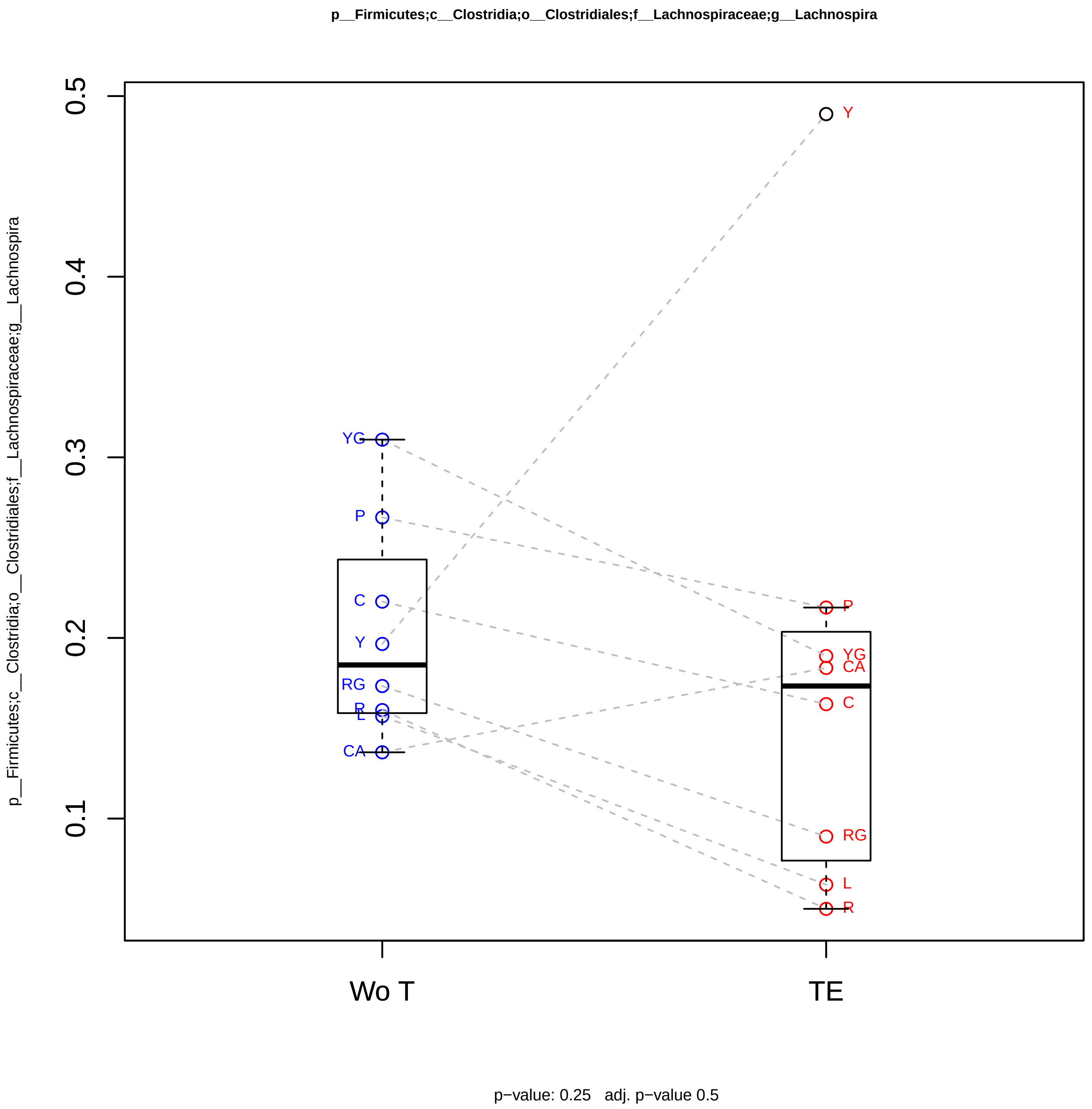
p__Firmicutes;c__Clostridia;o__Clostridiales;f__Lachnospiraceae;g__Lachnospira
p__Firmicutes;c__Clostridia;o__Clostridiales;f__Lachnospiraceae;g__Lachnospira
0.1
0.2
0.3
0.4
0.5
YG
P
C
Y
RG
R
L
CA
Y
P
YG
CA
C
RG
L
R
Wo T
TE
p−value: 0.25 adj. p−value 0.5

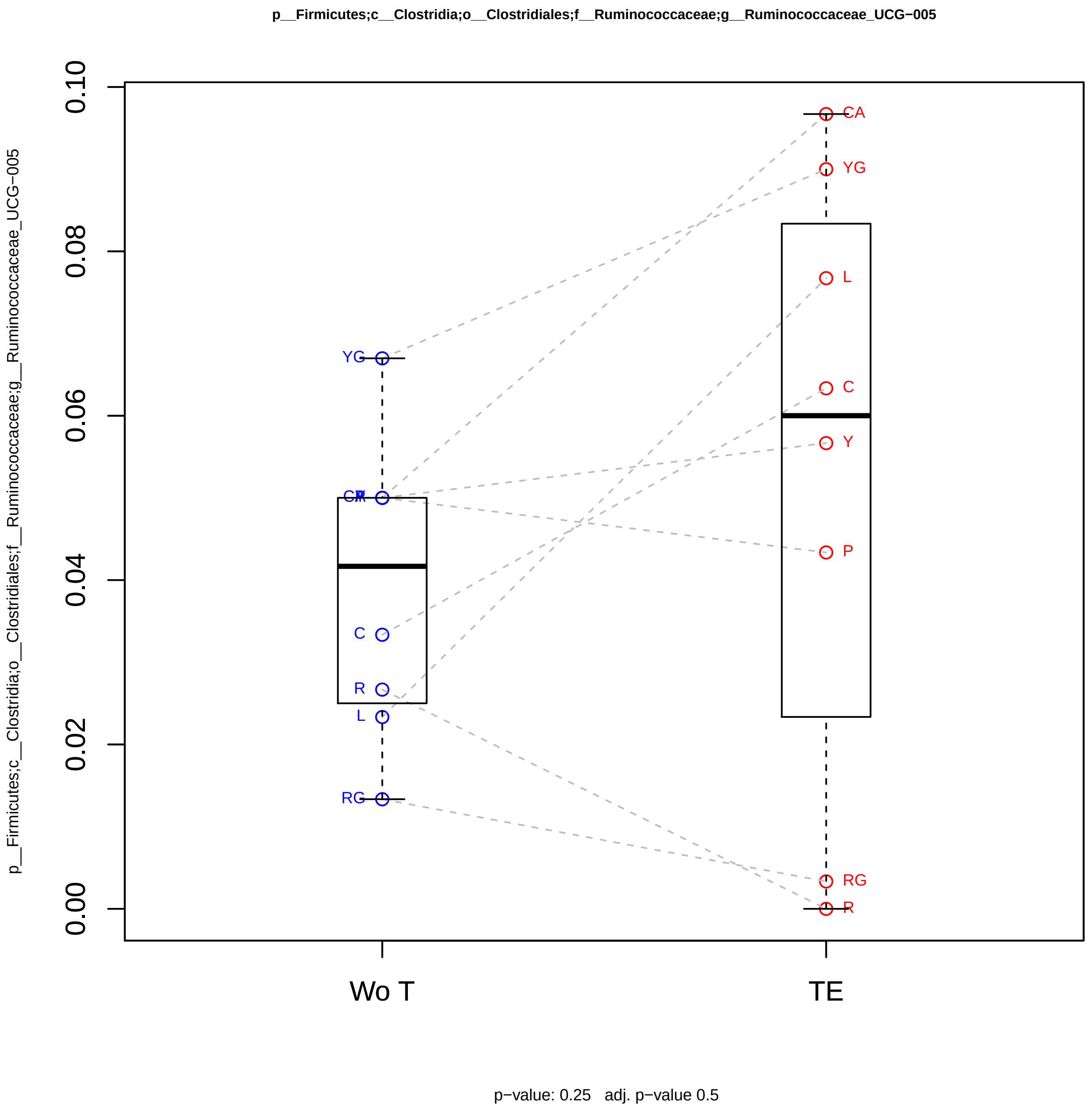
p__Firmicutes;c__Clostridia;o__Clostridiales;f__Ruminococcaceae;g__Ruminococcaceae_UCG−005
p__Firmicutes;c__Clostridia;o__Clostridiales;f__Ruminococcaceae;g__Ruminococcaceae_UCG−005
0.00
0.02
0.04
0.06
0.08
0.10
YG
C
R
L
RG
CA
YG
L
C
Y
P
RG
R
Wo T
TE
p−value: 0.25 adj. p−value 0.5

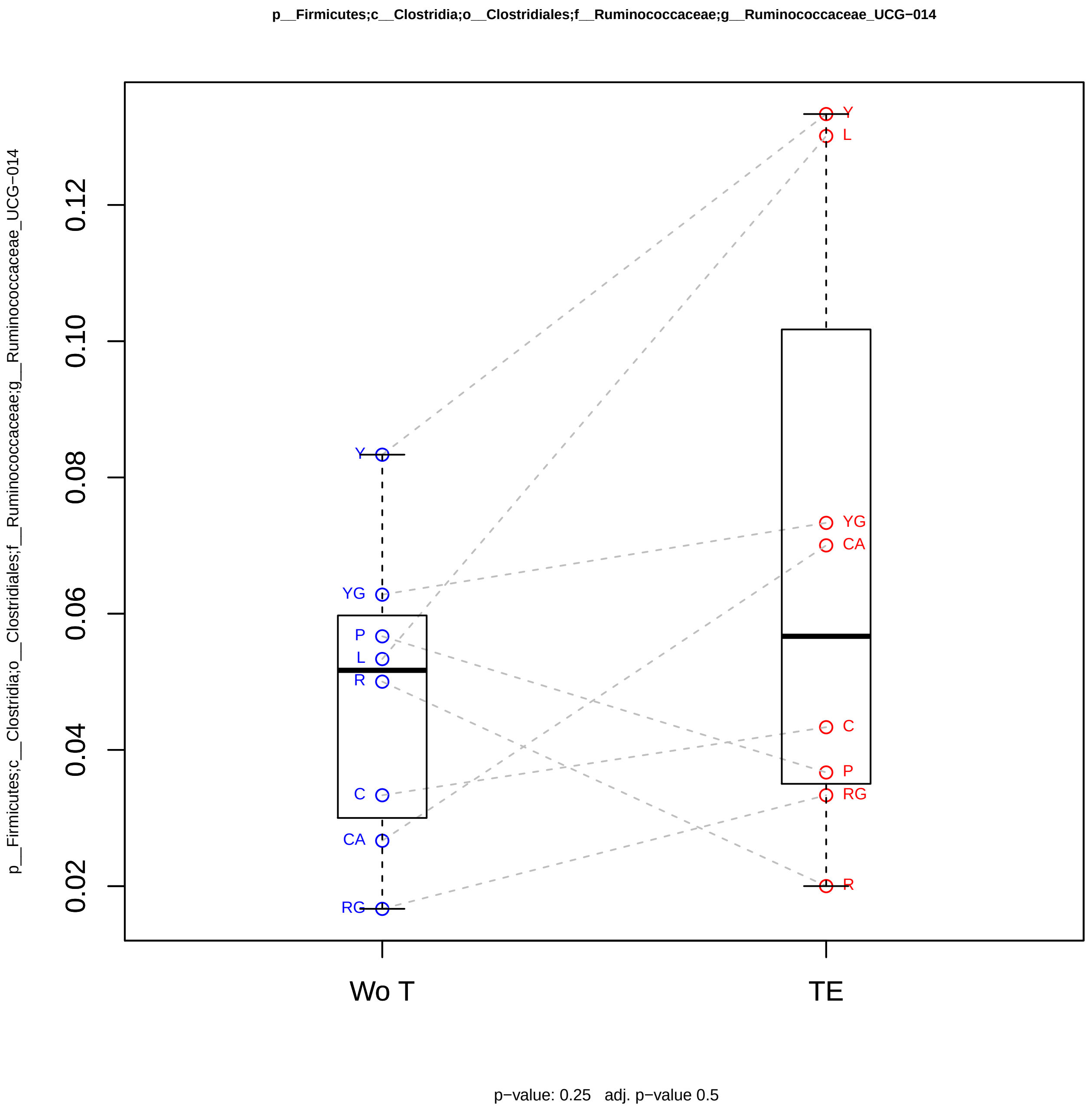
p__Firmicutes;c__Clostridia;o__Clostridiales;f__Ruminococcaceae;g__Ruminococcaceae_UCG−014
p__Firmicutes;c__Clostridia;o__Clostridiales;f__Ruminococcaceae;g__Ruminococcaceae_UCG−014
0.02
0.04
0.06
0.08
0.10
0.12
Y
YG
P
L
R
C
CA
RG
Y
L
YG
CA
C
P
RG
R
Wo T
TE
p−value: 0.25 adj. p−value 0.5

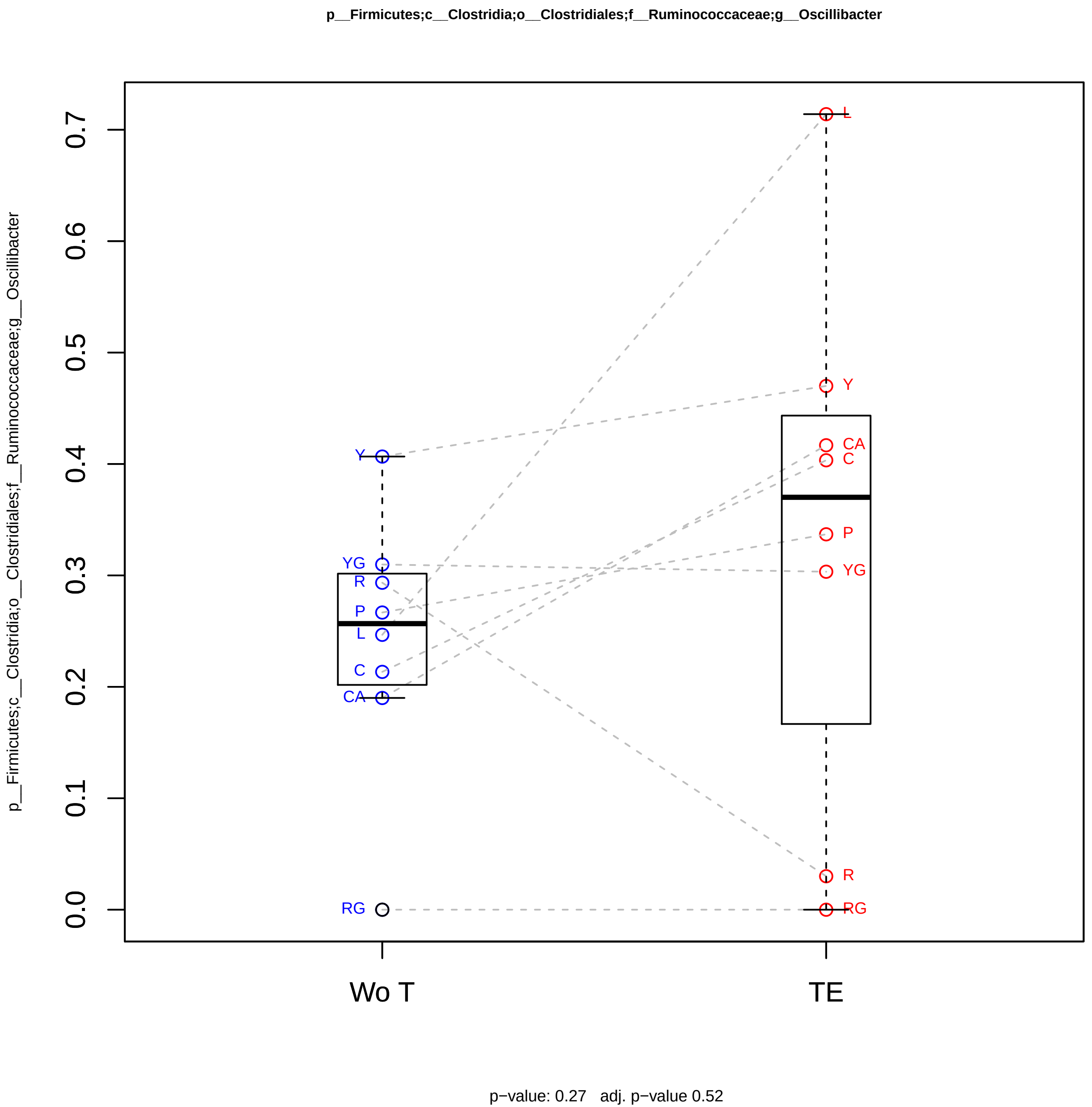
p__Firmicutes;c__Clostridia;o__Clostridiales;f__Ruminococcaceae;g__Oscillibacter
p__Firmicutes;c__Clostridia;o__Clostridiales;f__Ruminococcaceae;g__Oscillibacter
0.0
0.1
0.2
0.3
0.4
0.5
0.6
0.7
Y
YG
R
P
L
C
CA
RG
L
Y
CA
C
P
YG
R
RG
Wo T
TE
p−value: 0.27 adj. p−value 0.52

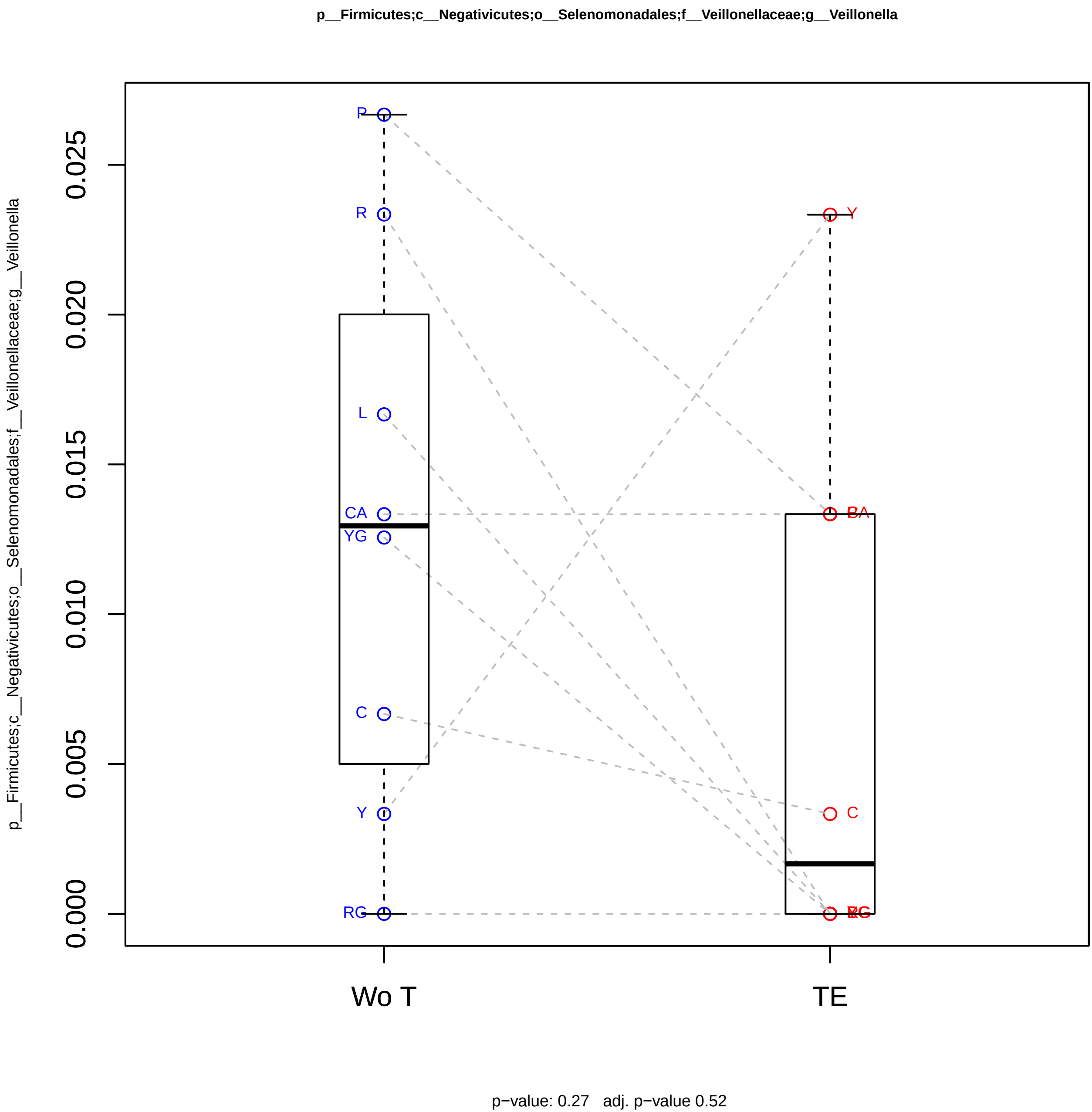
p__Firmicutes;c__Negativicutes;o__Selenomonadales;f__Veillonellaceae;g__Veillonella
p__Firmicutes;c__Negativicutes;o__Selenomonadales;f__Veillonellaceae;g__Veillonella
0.000
0.005
0.010
0.015
0.020
0.025
P
R
L
CA
YG
C
Y
RG
Y
C
Wo T
TE
p−value: 0.27 adj. p−value 0.52

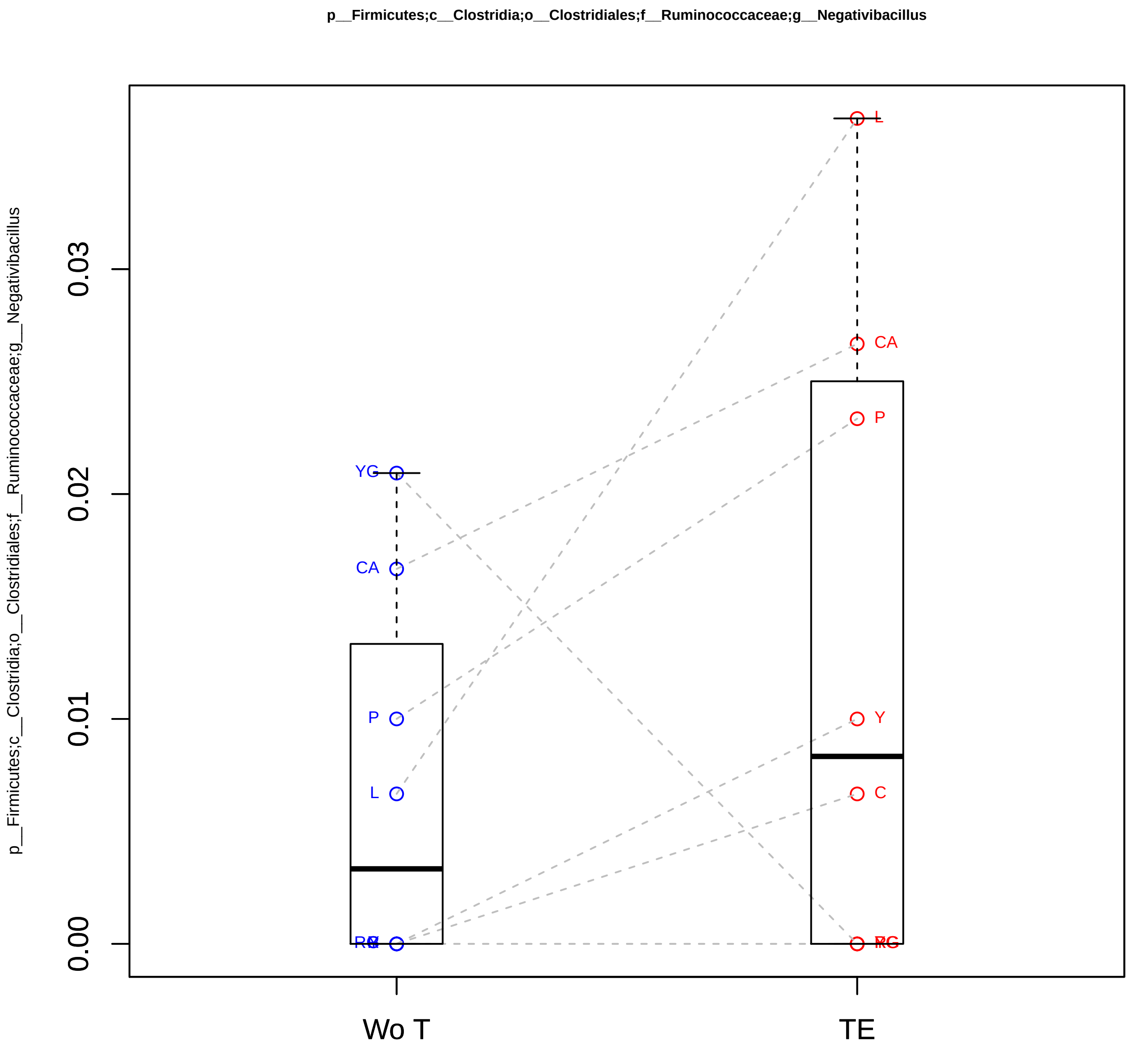

p−value: 0.29 adj. p−value 0.55

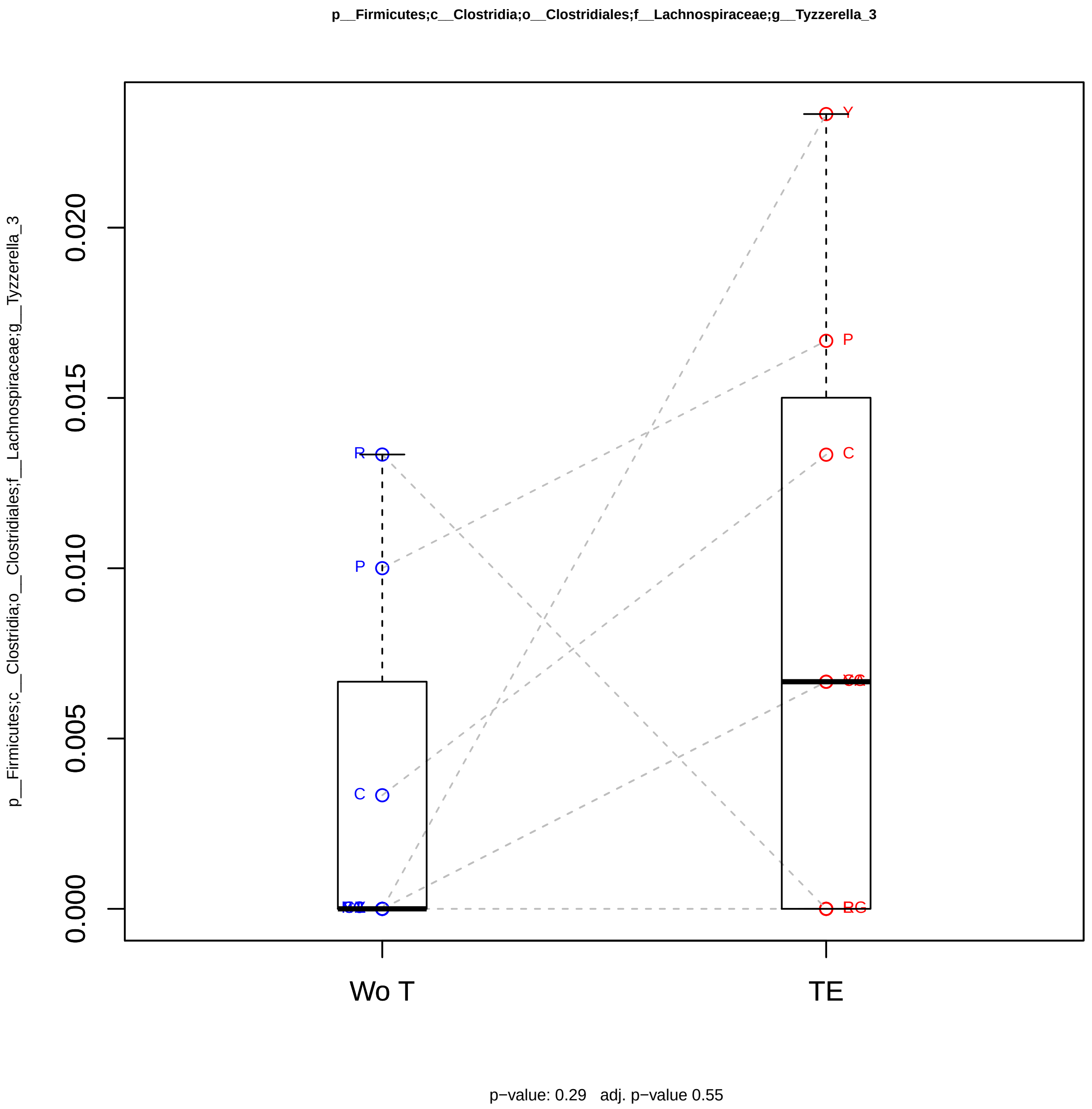
p__Firmicutes;c__Clostridia;o__Clostridiales;f__Lachnospiraceae;g__Tyzzerella_3
p__Firmicutes;c__Clostridia;o__Clostridiales;f__Lachnospiraceae;g__Tyzzerella_3
0.000
0.005
0.010
0.015
0.020
R
P
C
Y
P
C
Wo T
TE
p−value: 0.29 adj. p−value 0.55

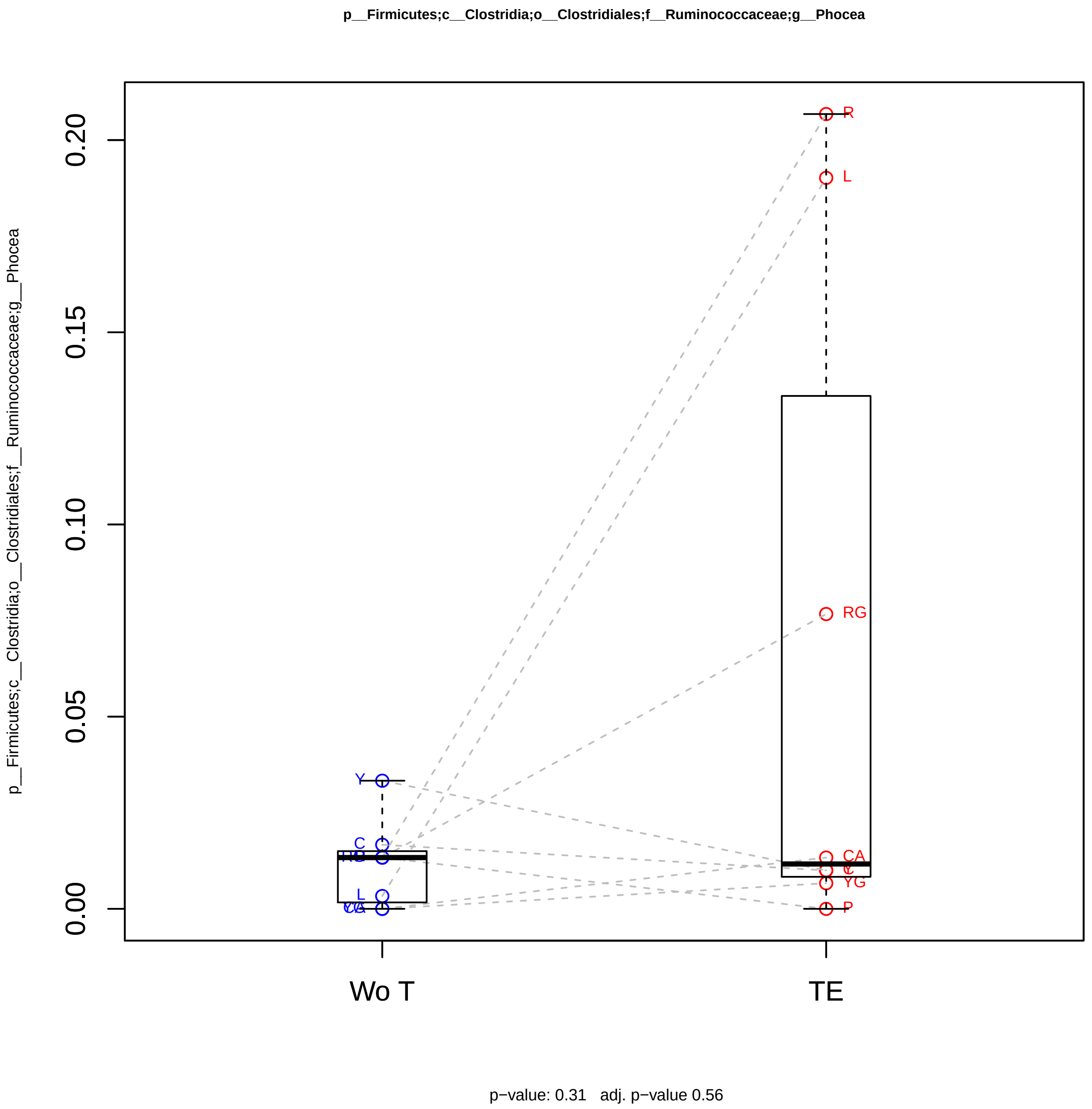
p__Firmicutes;c__Clostridia;o__Clostridiales;f__Ruminococcaceae;g__Phocea
p__Firmicutes;c__Clostridia;o__Clostridiales;f__Ruminococcaceae;g__Phocea
0.00
0.05
0.10
0.15
0.20
Wo T
TE
Y
C
L
R
L
RG
CA
YG
P
p−value: 0.31   adj. p−value 0.56

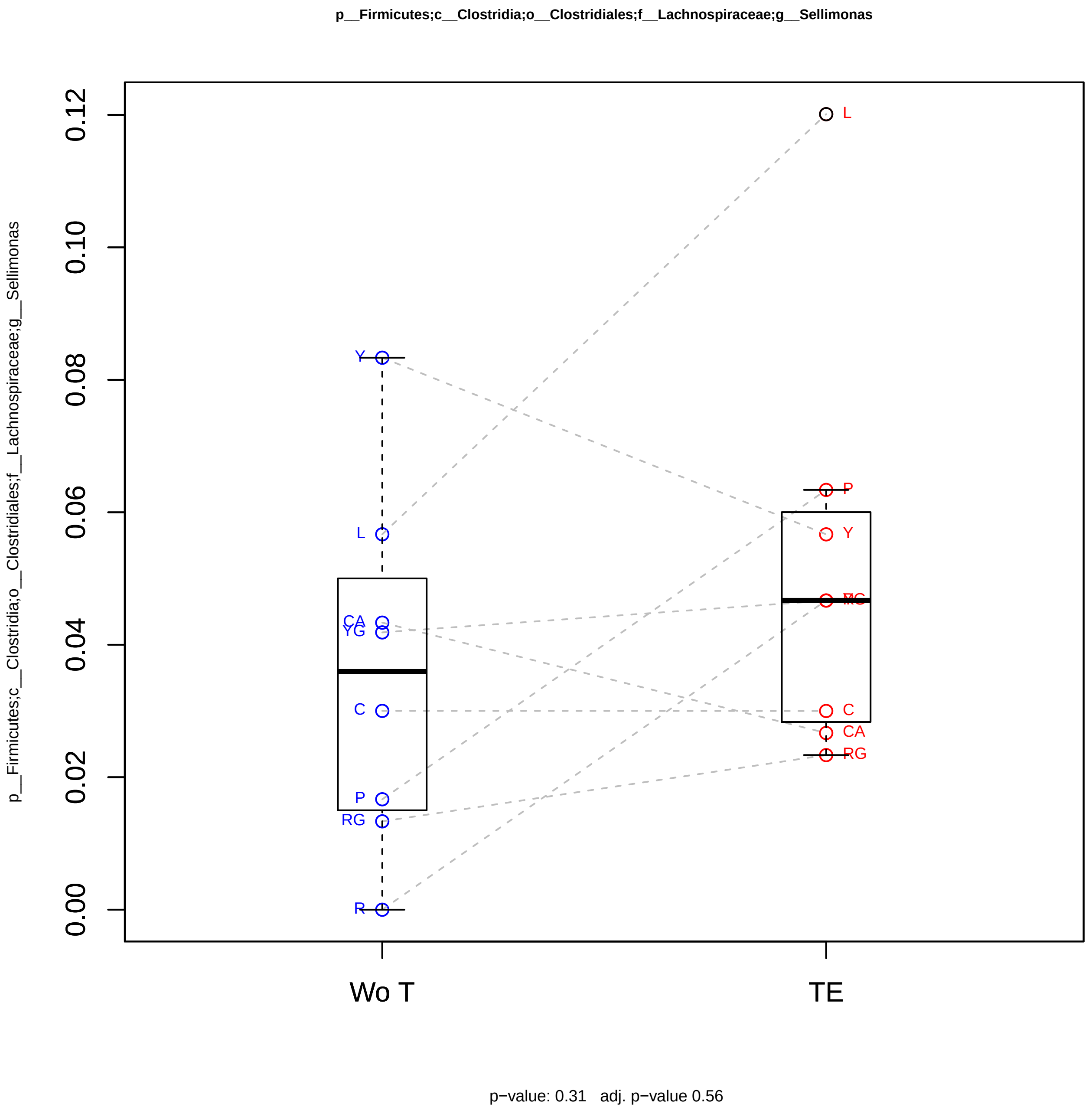
p__Firmicutes;c__Clostridia;o__Clostridiales;f__Lachnospiraceae;g__Sellimonas
p__Firmicutes;c__Clostridia;o__Clostridiales;f__Lachnospiraceae;g__Sellimonas
0.00
0.02
0.04
0.06
0.08
0.10
0.12
Wo T
TE
Y
L
CA
YG
C
P
RG
R
L
P
Y
C
CA
RG
p−value: 0.31 adj. p−value 0.56

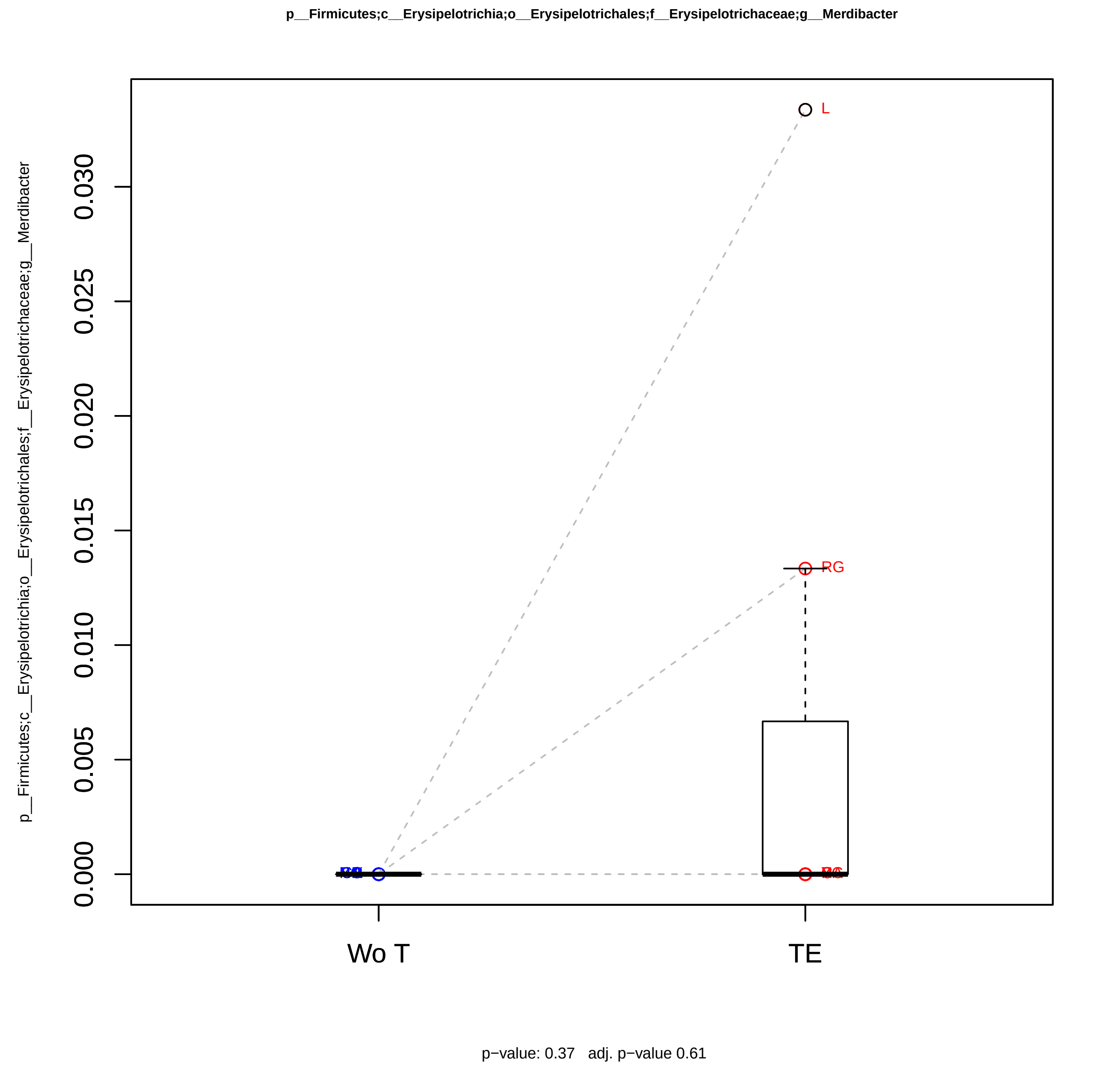

p__Firmicutes;c__Erysipelotrichia;o__Erysipelotrichales;f__Erysipelotrichaceae;g__Merdibacter
p__Firmicutes;c__Erysipelotrichia;o__Erysipelotrichales;f__Erysipelotrichaceae;g__Merdibacter
0.000
0.005
0.010
0.015
0.020
0.025
0.030
L
RG
Wo T
TE
p−value: 0.37   adj. p−value 0.61

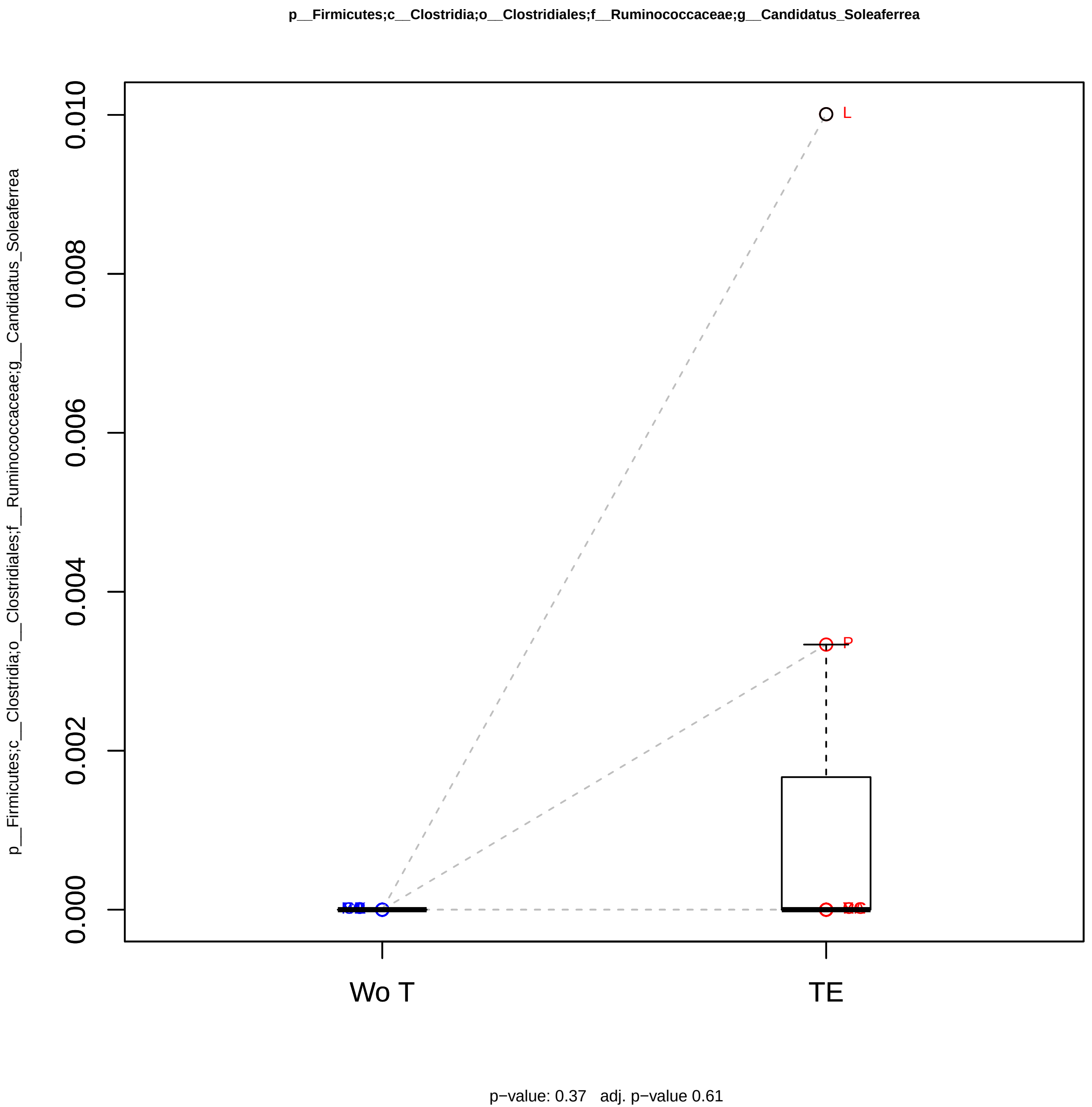
p__Firmicutes;c__Clostridia;o__Clostridiales;f__Ruminococcaceae;g__Candidatus_Soleaferrea
p__Firmicutes;c__Clostridia;o__Clostridiales;f__Ruminococcaceae;g__Candidatus_Soleaferrea
0.010
0.008
0.006
0.004
0.002
0.000
L
P
Wo T
TE
p−value: 0.37 adj. p−value 0.61

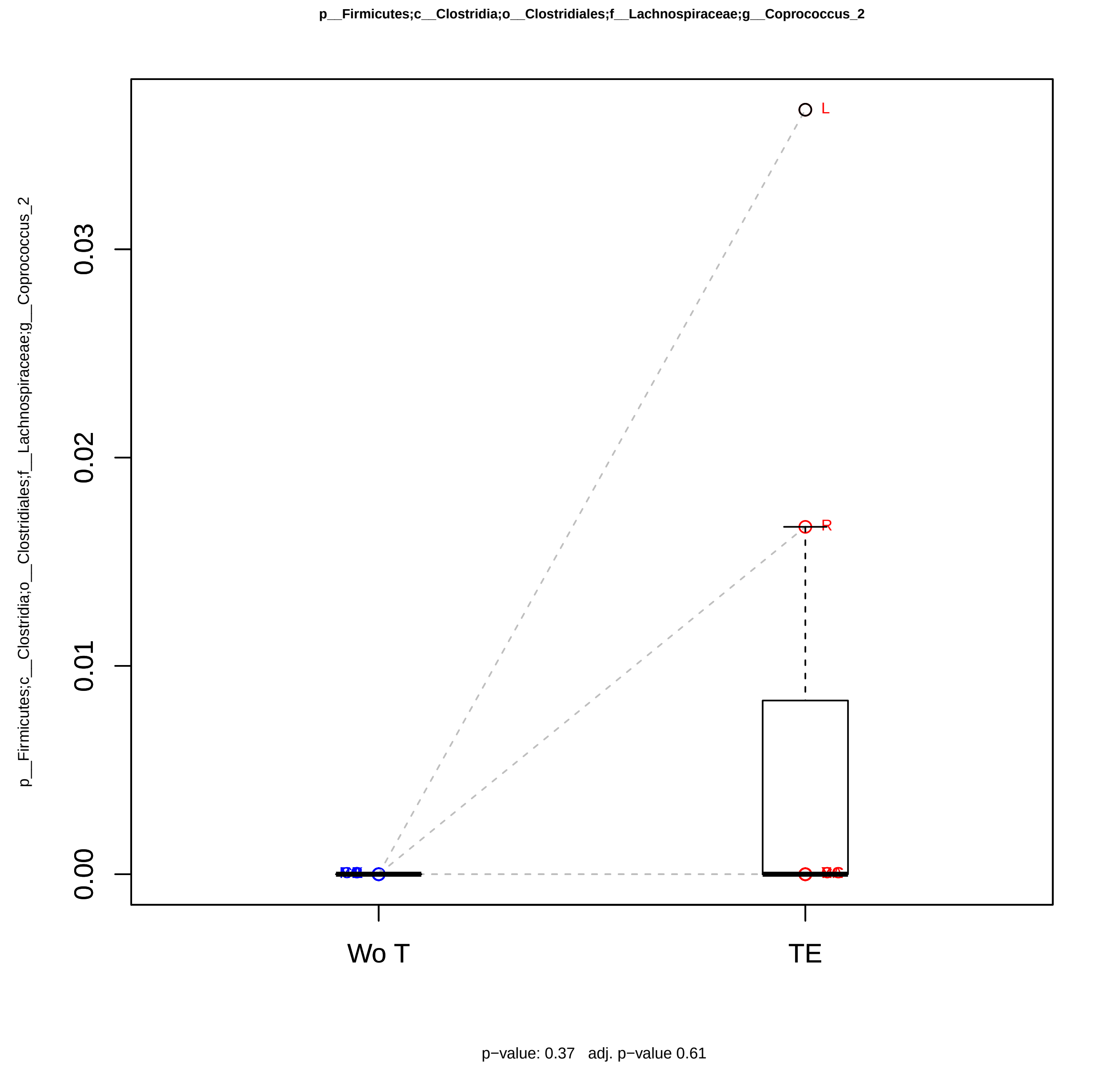
p__Firmicutes;c__Clostridia;o__Clostridiales;f__Lachnospiraceae;g__Coprococcus_2
p__Firmicutes;c__Clostridia;o__Clostridiales;f__Lachnospiraceae;g__Coprococcus_2
0.00
0.01
0.02
0.03
L
R
Wo T
TE
p−value: 0.37 adj. p−value 0.61

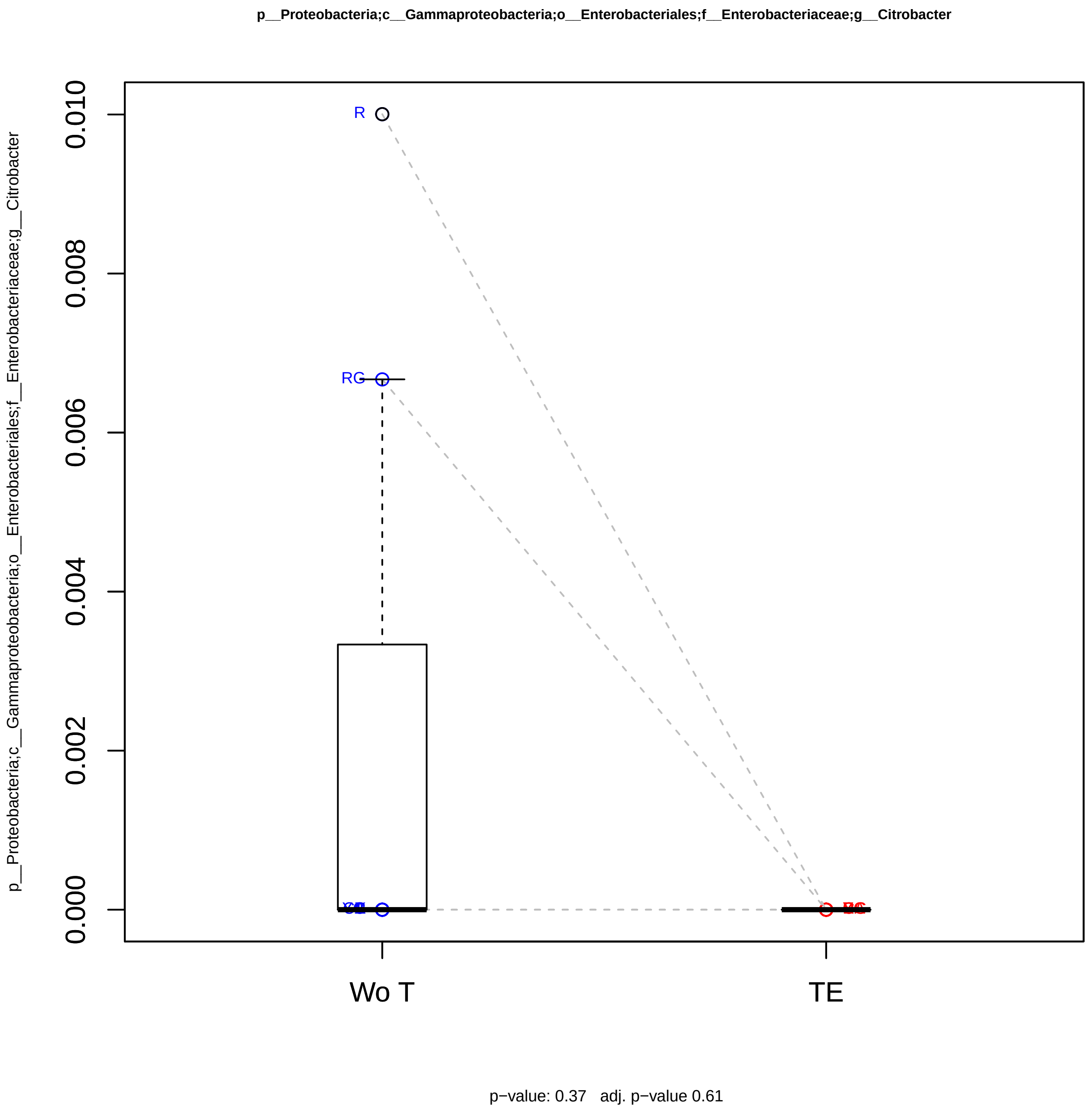
p__Proteobacteria;c__Gammaproteobacteria;o__Enterobacteriales;f__Enterobacteriaceae;g__Citrobacter
p__Proteobacteria;c__Gammaproteobacteria;o__Enterobacteriales;f__Enterobacteriaceae;g__Citrobacter
0.000
0.002
0.004
0.006
0.008
0.010
R
RG
Wo T
TE
p−value: 0.37 adj. p−value 0.61

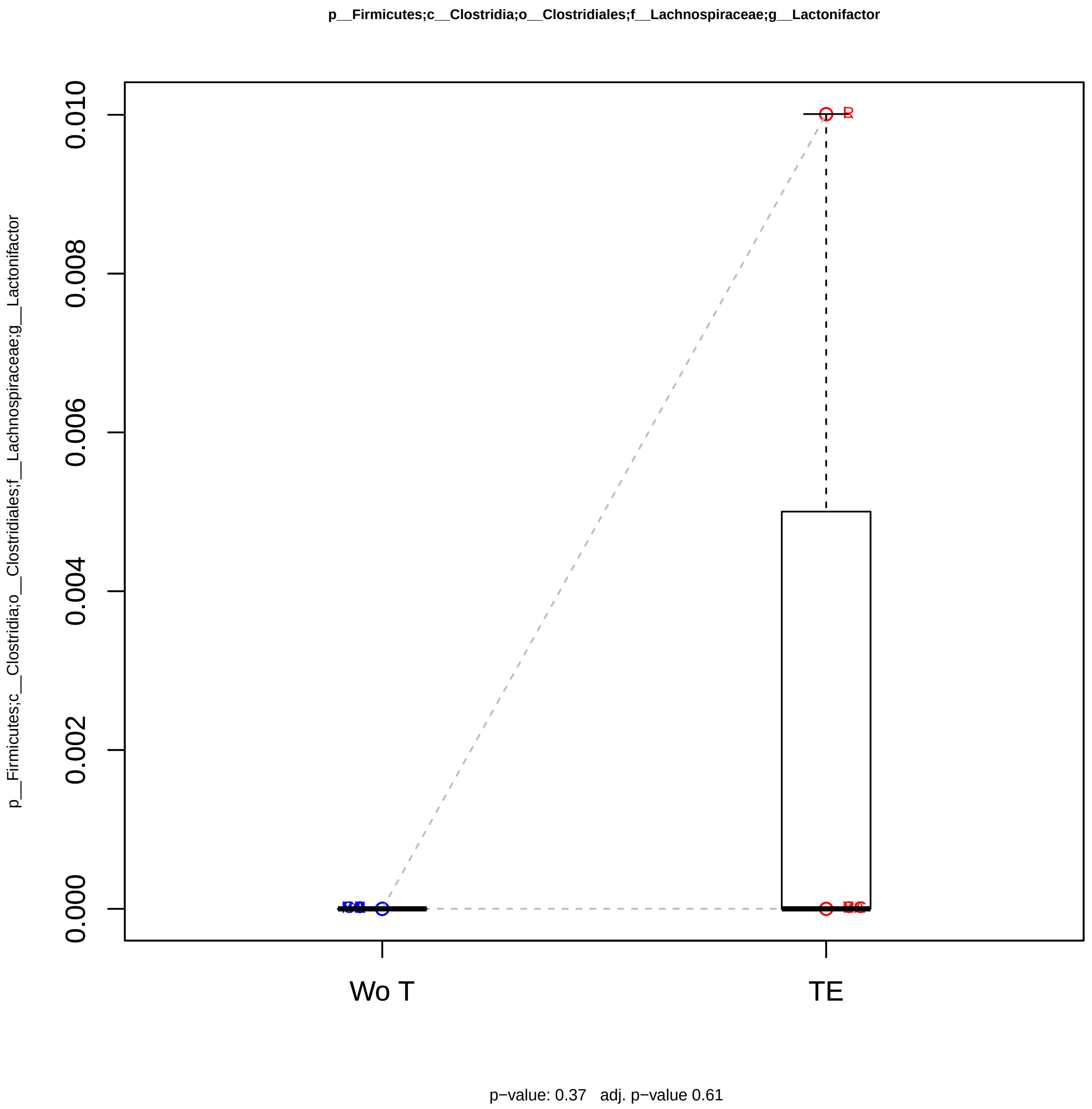
p__Firmicutes;c__Clostridia;o__Clostridiales;f__Lachnospiraceae;g__Lactonifactor
p__Firmicutes;c__Clostridia;o__Clostridiales;f__Lachnospiraceae;g__Lactonifactor
0.000
0.002
0.004
0.006
0.008
0.010
Wo T
TE
p−value: 0.37   adj. p−value 0.61

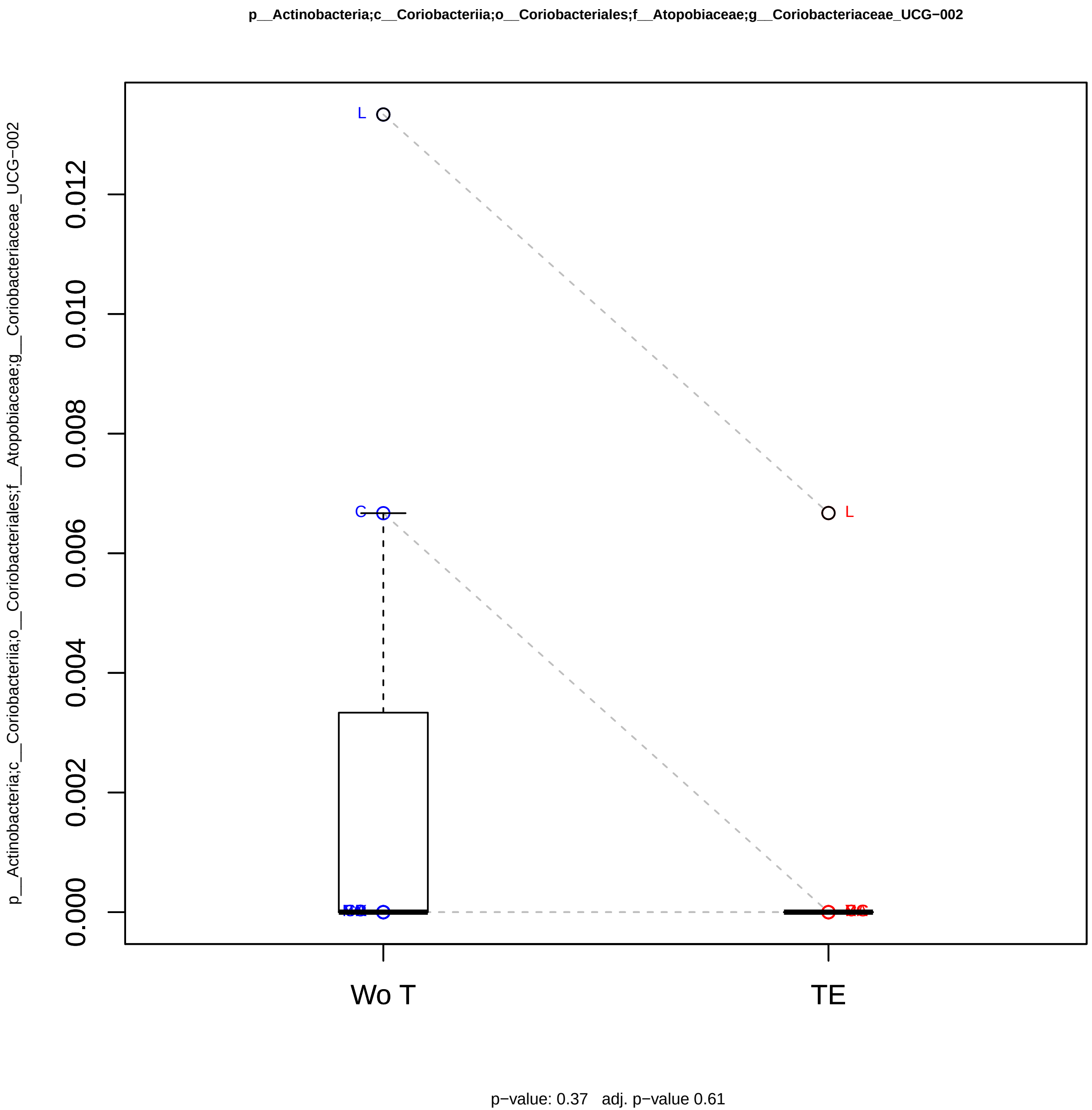

p__Actinobacteria;c__Coriobacteriia;o__Coriobacteriales;f__Atopobiaceae;g__Coriobacteriaceae_UCG−002
p__Actinobacteria;c__Coriobacteriia;o__Coriobacteriales;f__Atopobiaceae;g__Coriobacteriaceae_UCG−002
0.000
0.002
0.004
0.006
0.008
0.010
0.012
L
G
L
Wo T
TE
p−value: 0.37   adj. p−value 0.61

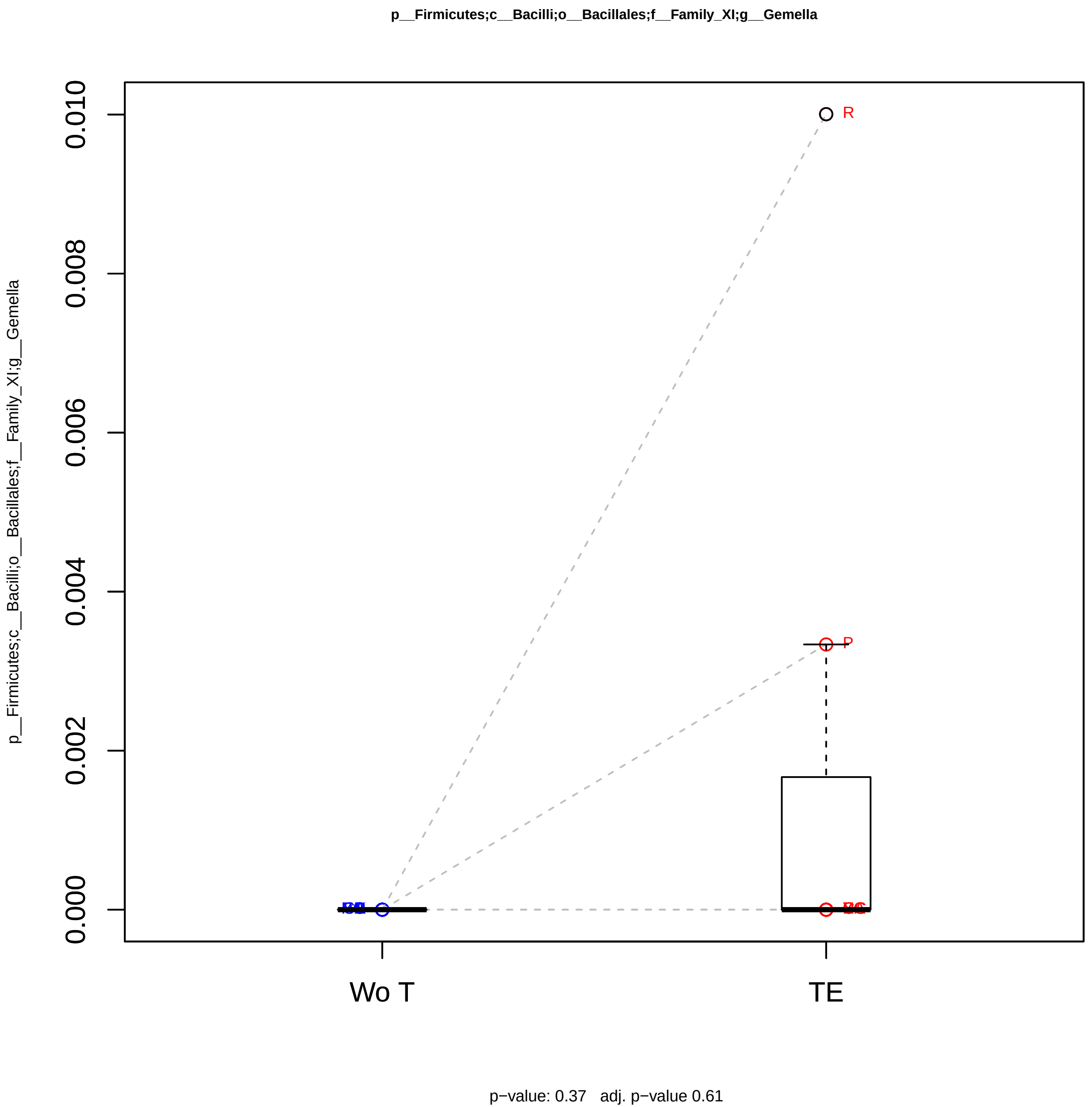
p__Firmicutes;c__Bacilli;o__Bacillales;f__Family_XI;g__Gemella
p__Firmicutes;c__Bacilli;o__Bacillales;f__Family_XI;g__Gemella
0.000
0.002
0.004
0.006
0.008
0.010
R
P
Wo T
TE
p−value: 0.37 adj. p−value 0.61

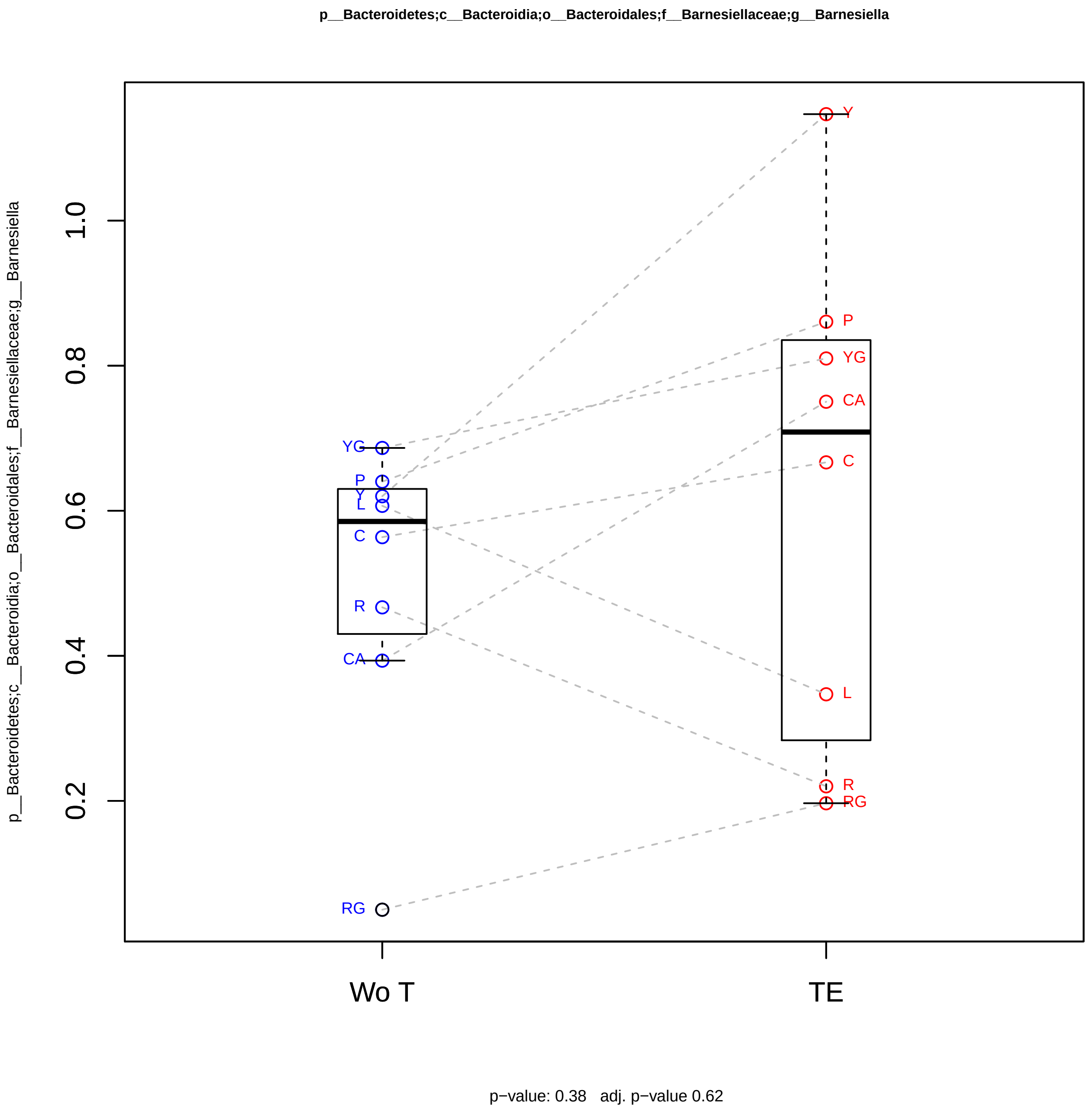

p__Bacteroidetes;c__Bacteroidia;o__Bacteroidales;f__Barnesiellaceae;g__Barnesiella
p__Bacteroidetes;c__Bacteroidia;o__Bacteroidales;f__Barnesiellaceae;g__Barnesiella
0.2
0.4
0.6
0.8
1.0
YG
P
Y
L
C
R
CA
RG
Y
P
YG
CA
C
L
R
RG
Wo T
TE
p−value: 0.38 adj. p−value 0.62

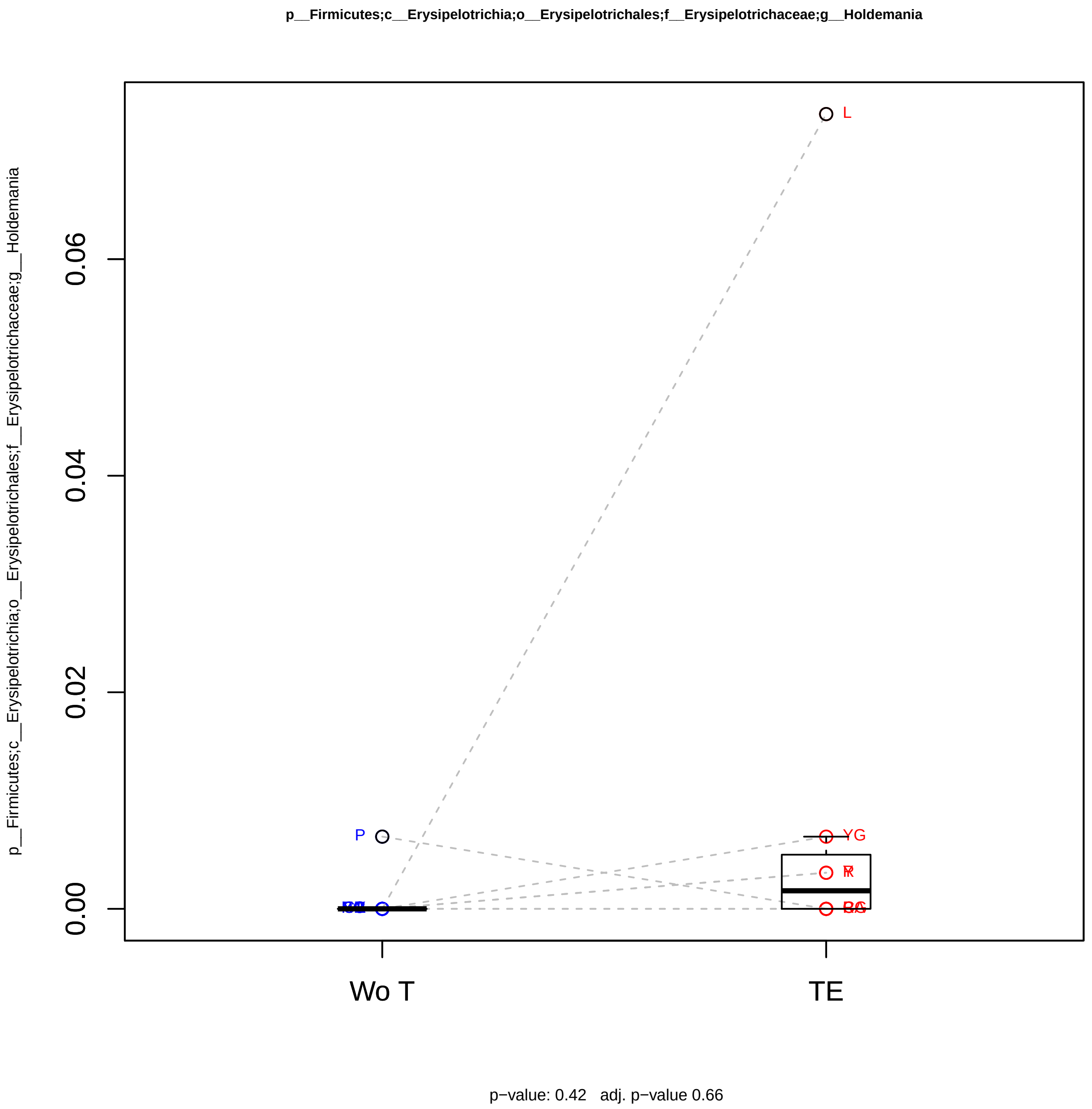
p__Firmicutes;c__Erysipelotrichia;o__Erysipelotrichales;f__Erysipelotrichaceae;g__Holdemania
p__Firmicutes;c__Erysipelotrichia;o__Erysipelotrichales;f__Erysipelotrichaceae;g__Holdemania
0.00
0.02
0.04
0.06
L
P
YG
Wo T
TE
p−value: 0.42 adj. p−value 0.66

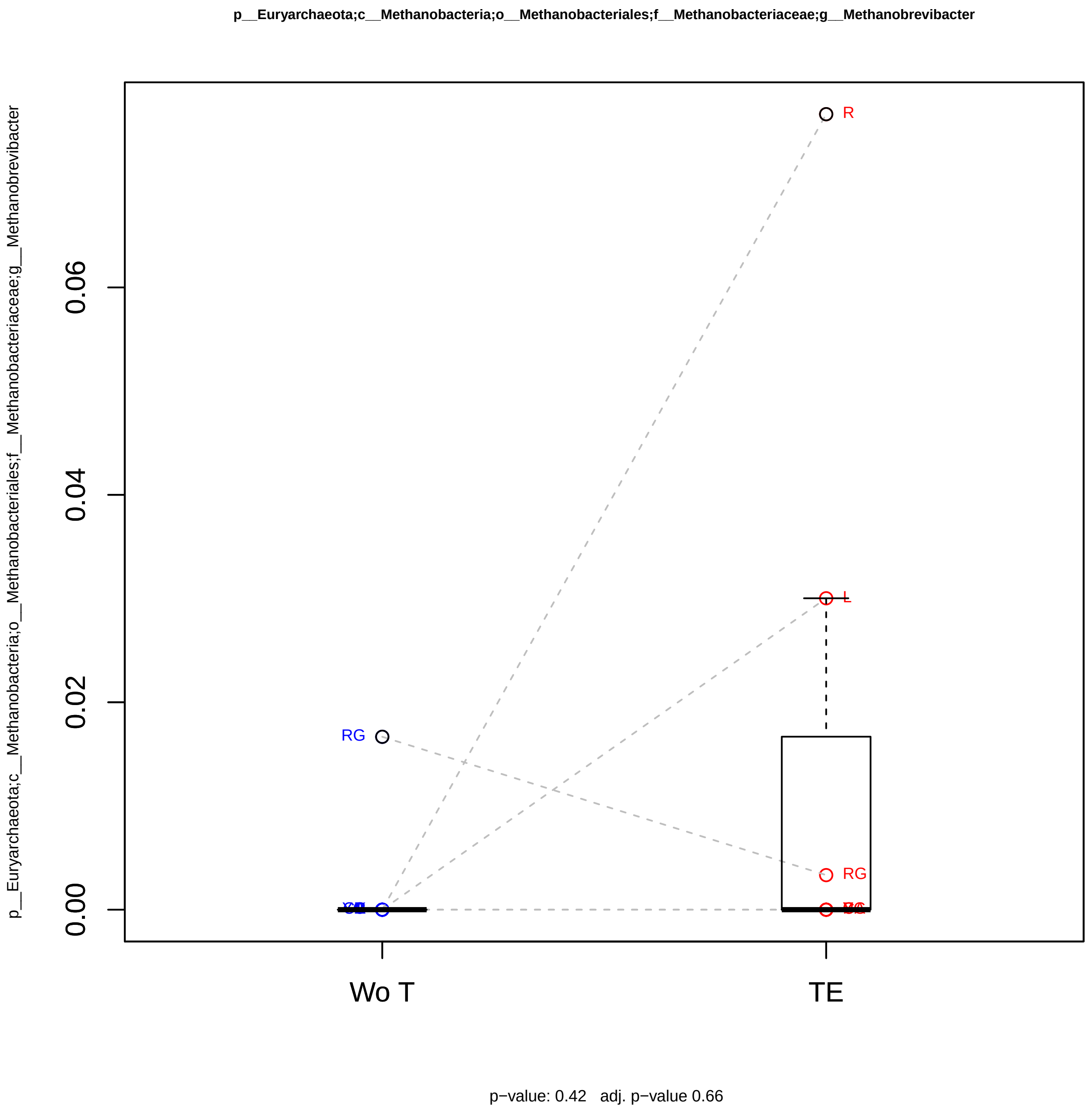
p__Euryarchaeota;c__Methanobacteria;o__Methanobacteriales;f__Methanobacteriaceae;g__Methanobrevibacter
p__Euryarchaeota;c__Methanobacteria;o__Methanobacteriales;f__Methanobacteriaceae;g__Methanobrevibacter
0.00
0.02
0.04
0.06
R
L
RG
RG
Wo T
TE
p−value: 0.42 adj. p−value 0.66

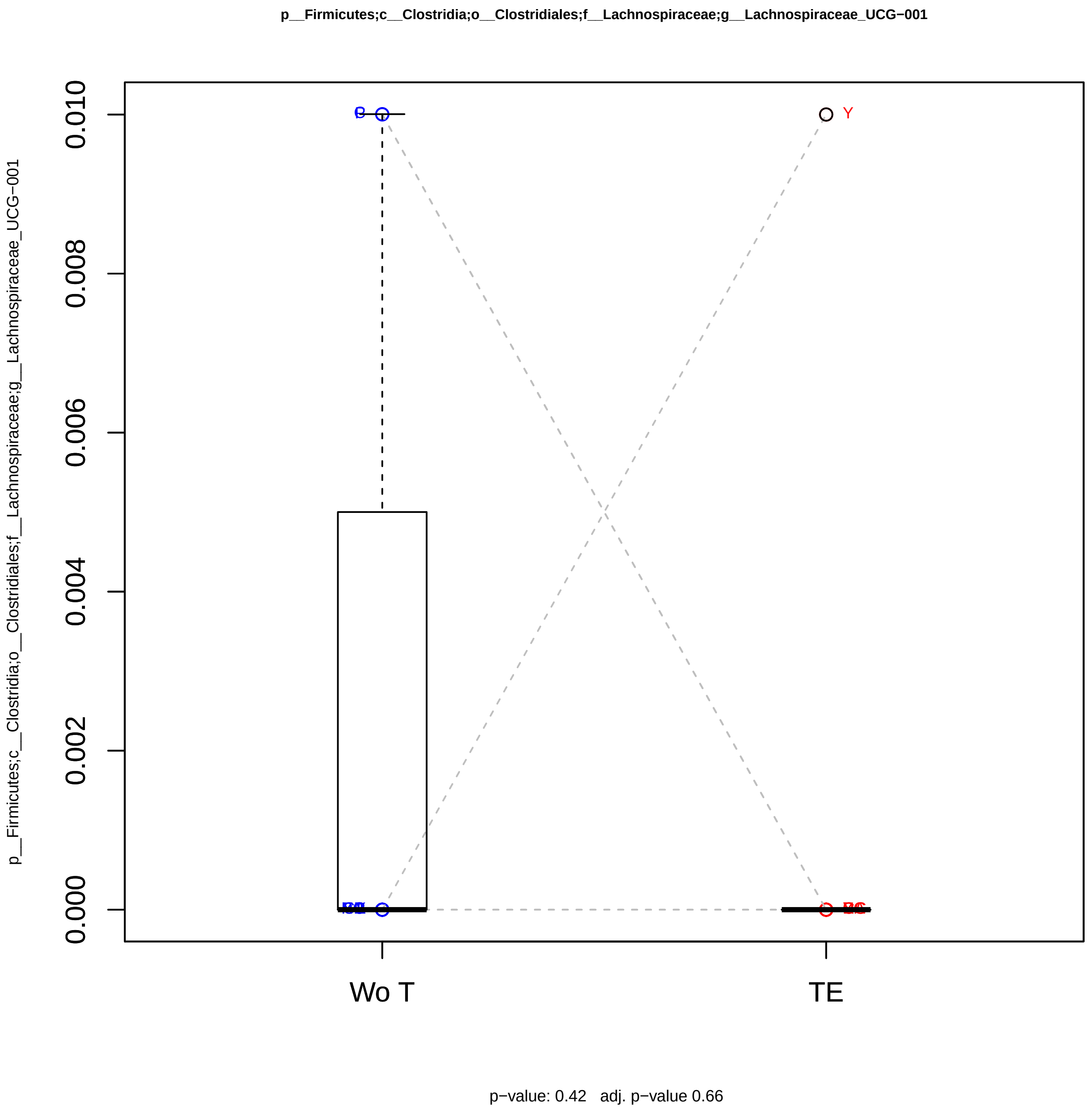

p__Firmicutes;c__Clostridia;o__Clostridiales;f__Lachnospiraceae;g__Lachnospiraceae_UCG−001
p__Firmicutes;c__Clostridia;o__Clostridiales;f__Lachnospiraceae;g__Lachnospiraceae_UCG−001
0.000
0.002
0.004
0.006
0.008
0.010
Y
Wo T
TE
p−value: 0.42 adj. p−value 0.66

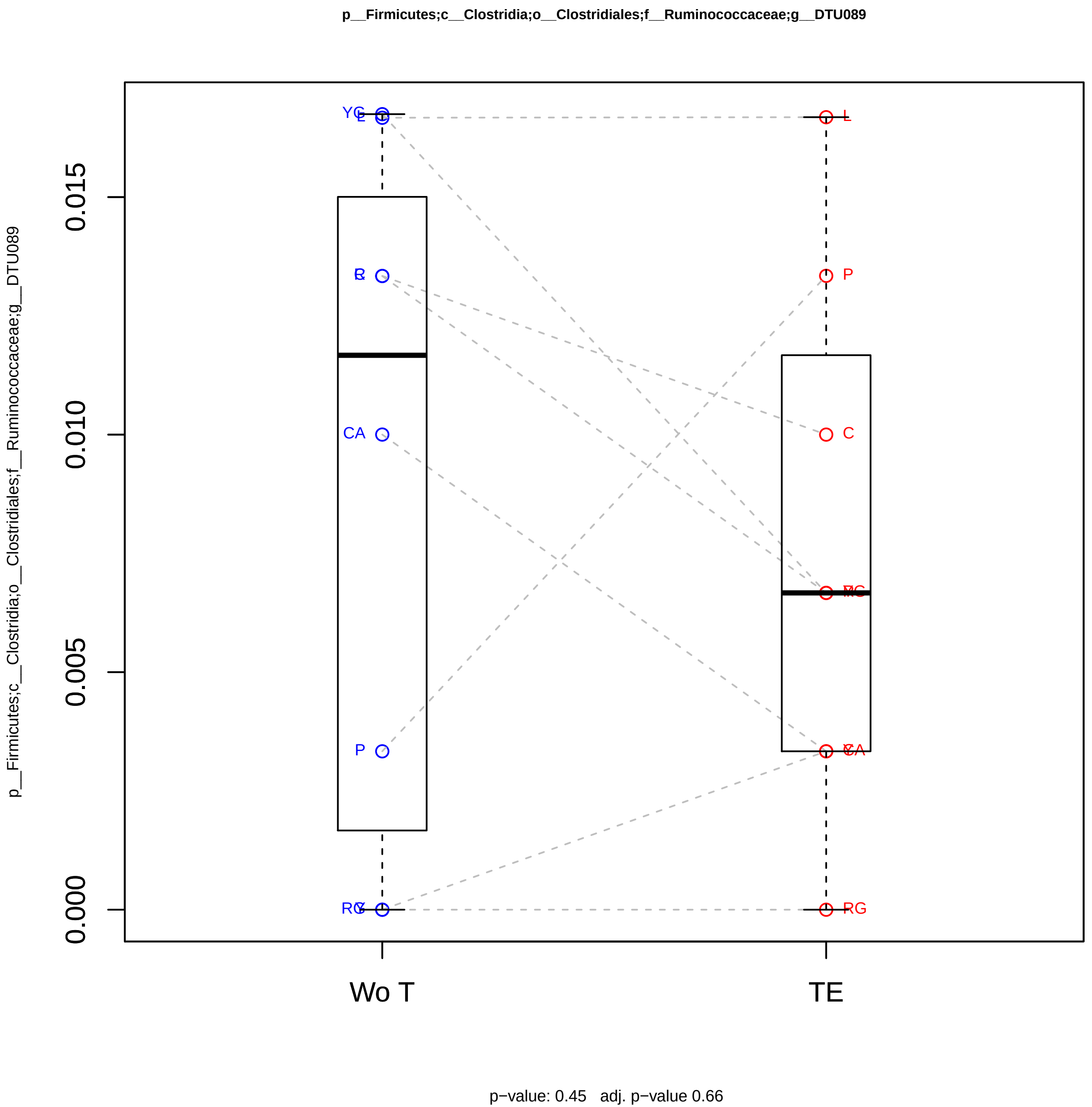
p__Firmicutes;c__Clostridia;o__Clostridiales;f__Ruminococcaceae;g__DTU089
p__Firmicutes;c__Clostridia;o__Clostridiales;f__Ruminococcaceae;g__DTU089
0.000
0.005
0.010
0.015
Wo T
TE
YC
L
P
C
CA
RG
p−value: 0.45 adj. p−value 0.66

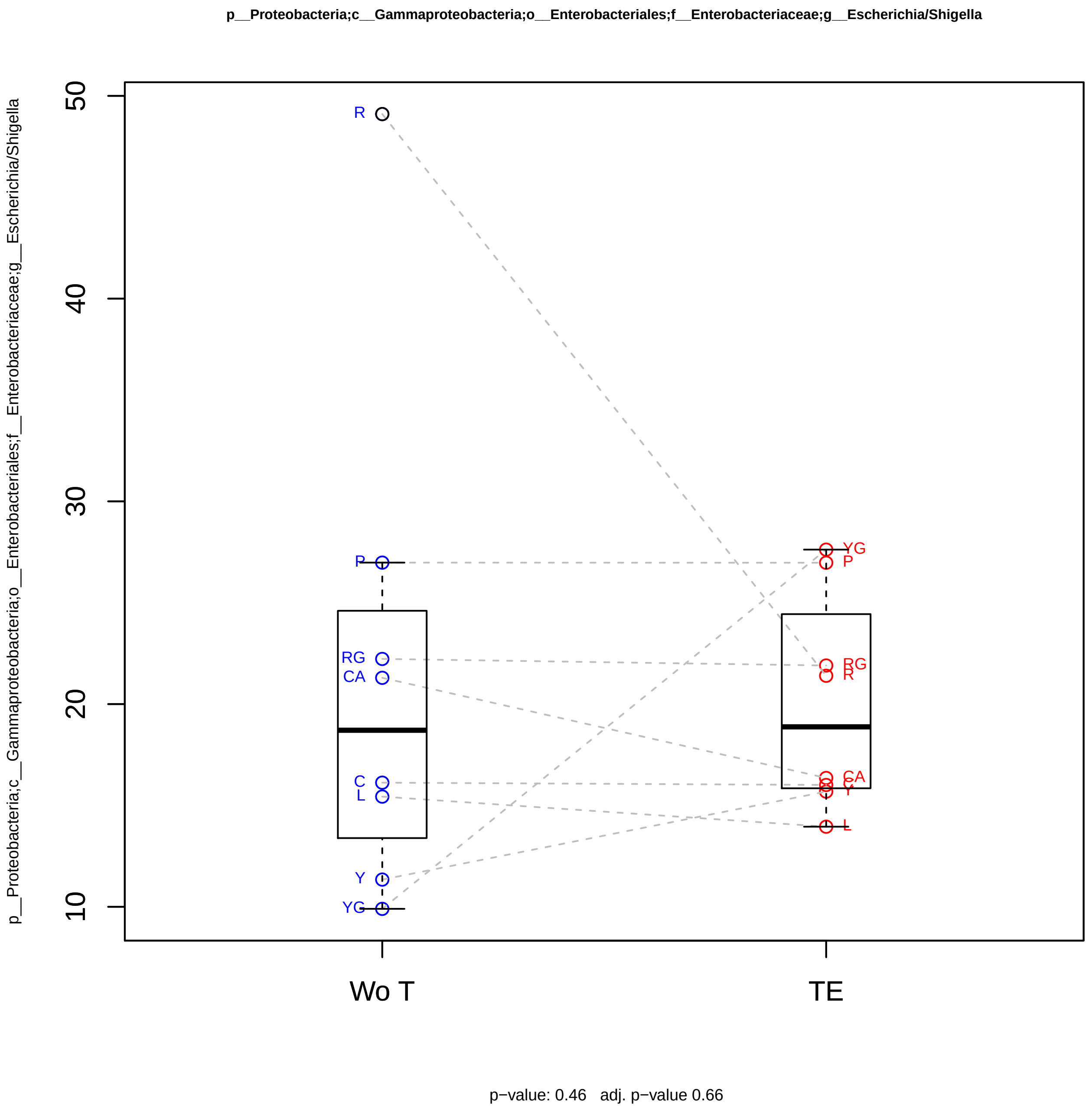
p__Proteobacteria;c__Gammaproteobacteria;o__Enterobacteriales;f__Enterobacteriaceae;g__Escherichia/Shigella
p__Proteobacteria;c__Gammaproteobacteria;o__Enterobacteriales;f__Enterobacteriaceae;g__Escherichia/Shigella
10
20
30
40
50
R
P
RG
CA
C
L
Y
YG
YG
P
RG
R
CA
C
Y
L
Wo T
TE
p−value: 0.46 adj. p−value 0.66

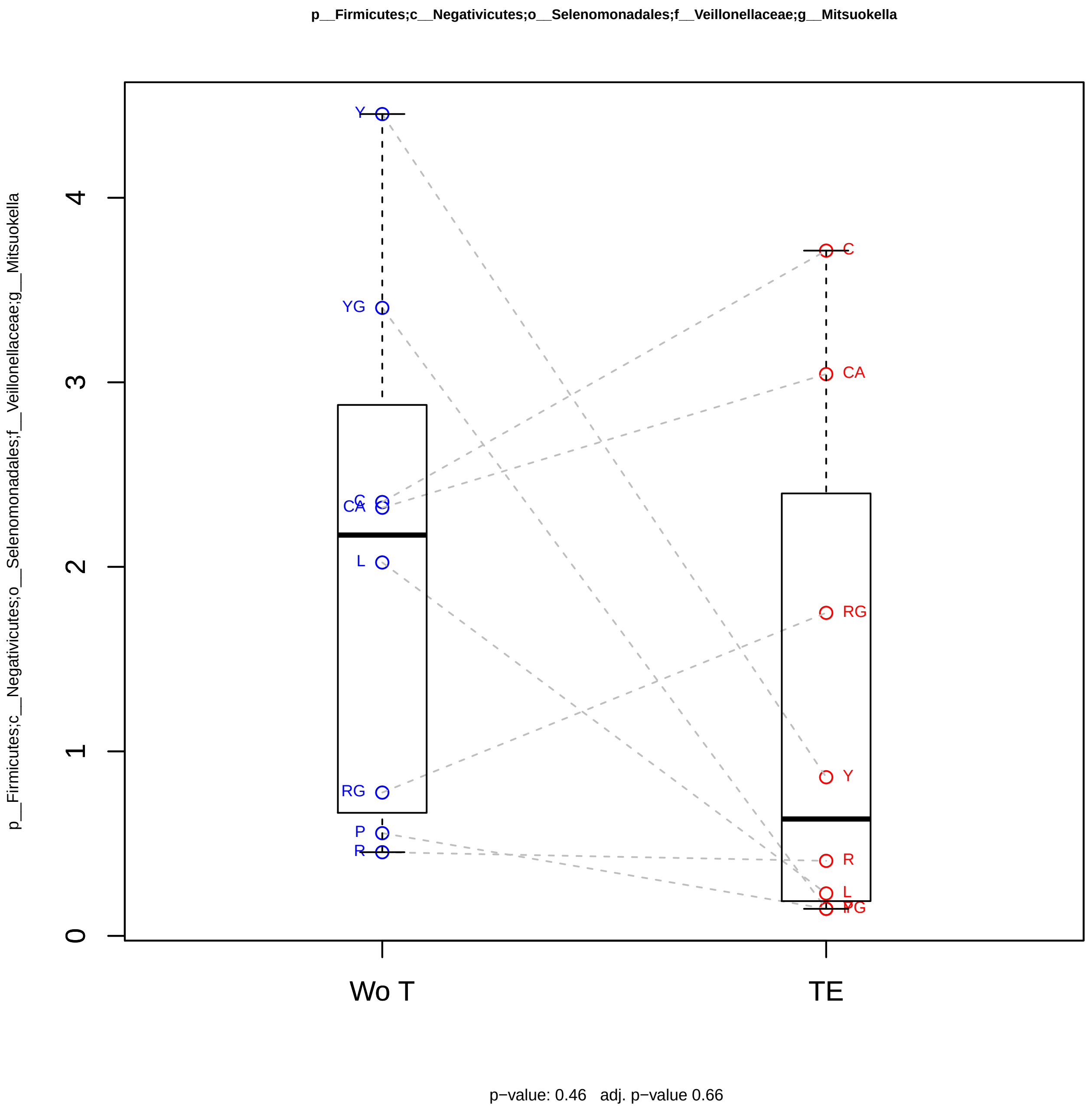

p__Firmicutes;c__Negativicutes;o__Selenomonadales;f__Veillonellaceae;g__Mitsuokella
p__Firmicutes;c__Negativicutes;o__Selenomonadales;f__Veillonellaceae;g__Mitsuokella
0
1
2
3
4
Y
YG
C
CA
L
RG
P
R
C
CA
RG
Y
R
L
PG
Wo T
TE
p−value: 0.46 adj. p−value 0.66

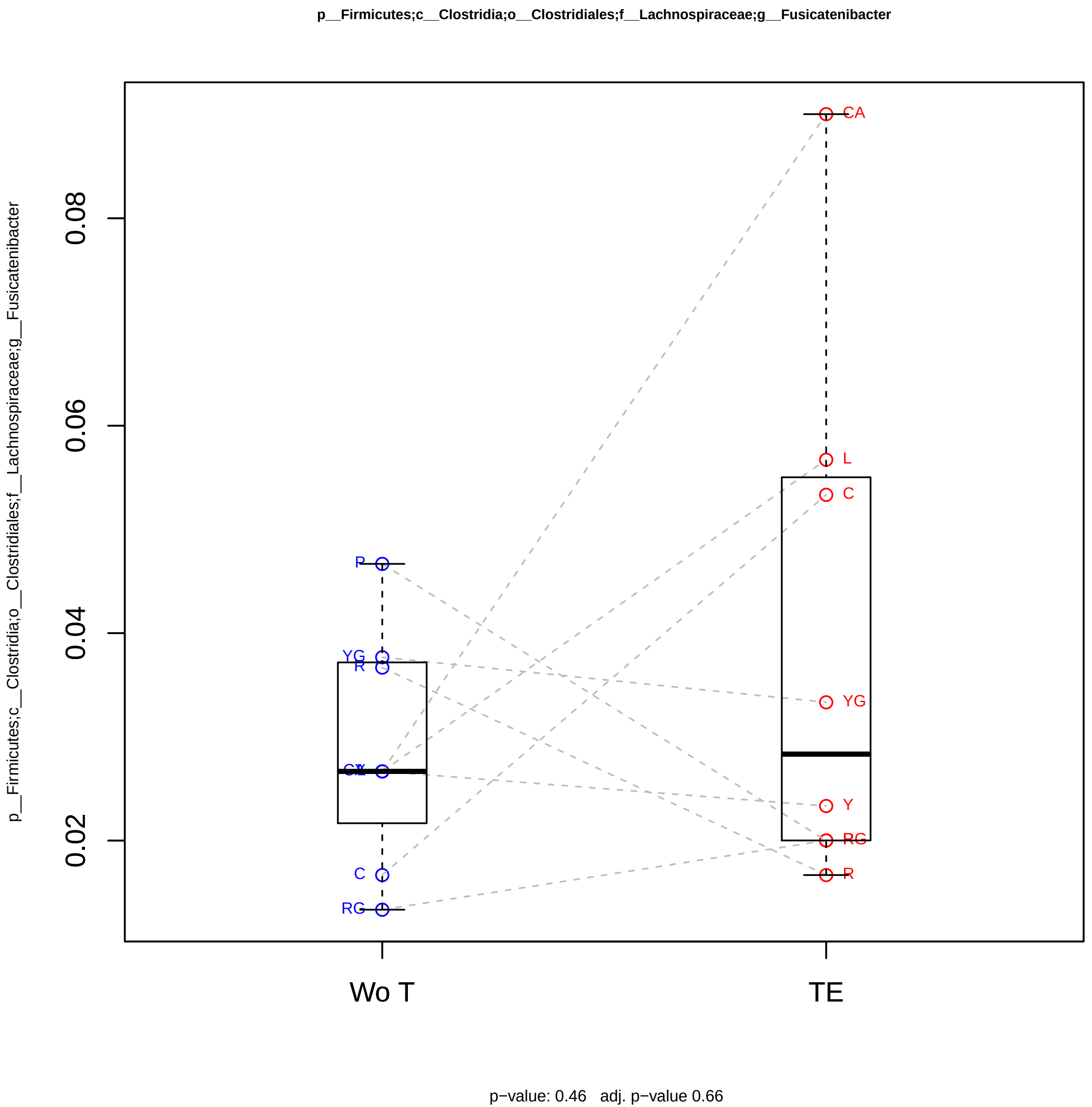
p__Firmicutes;c__Clostridia;o__Clostridiales;f__Lachnospiraceae;g__Fusicatenibacter
p__Firmicutes;c__Clostridia;o__Clostridiales;f__Lachnospiraceae;g__Fusicatenibacter
0.02
0.04
0.06
0.08
P
YG
R
C
RG
CA
L
C
YG
Y
RG
R
Wo T
TE
p−value: 0.46   adj. p−value 0.66

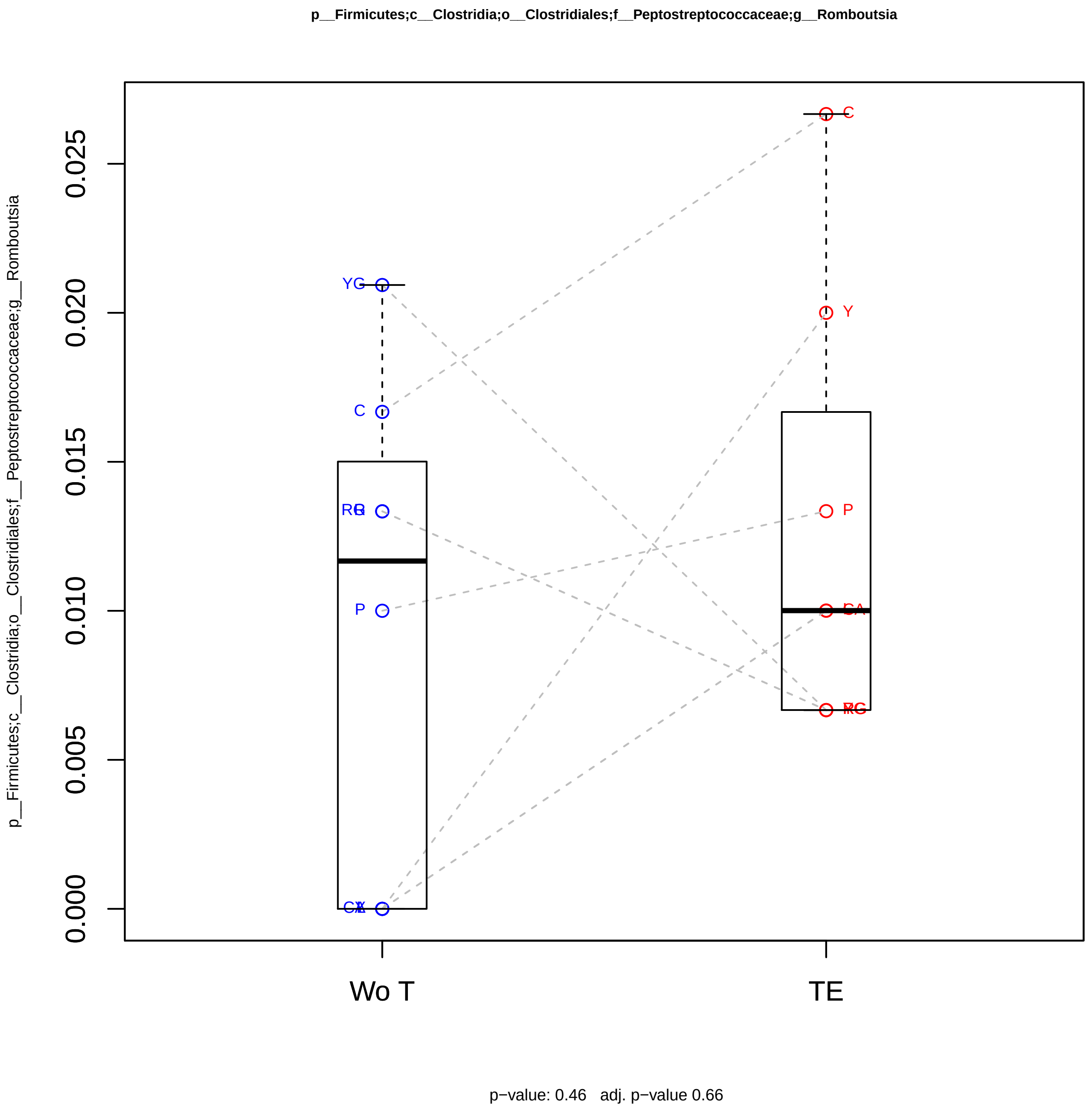
p__Firmicutes;c__Clostridia;o__Clostridiales;f__Peptostreptococcaceae;g__Romboutsia
p__Firmicutes;c__Clostridia;o__Clostridiales;f__Peptostreptococcaceae;g__Romboutsia
0.000
0.005
0.010
0.015
0.020
0.025
Wo T
TE
YG
C
P
Y
p−value: 0.46 adj. p−value 0.66

p__Firmicutes;c__Clostridia;o__Clostridiales;f__Lachnospiraceae;g__Howardella

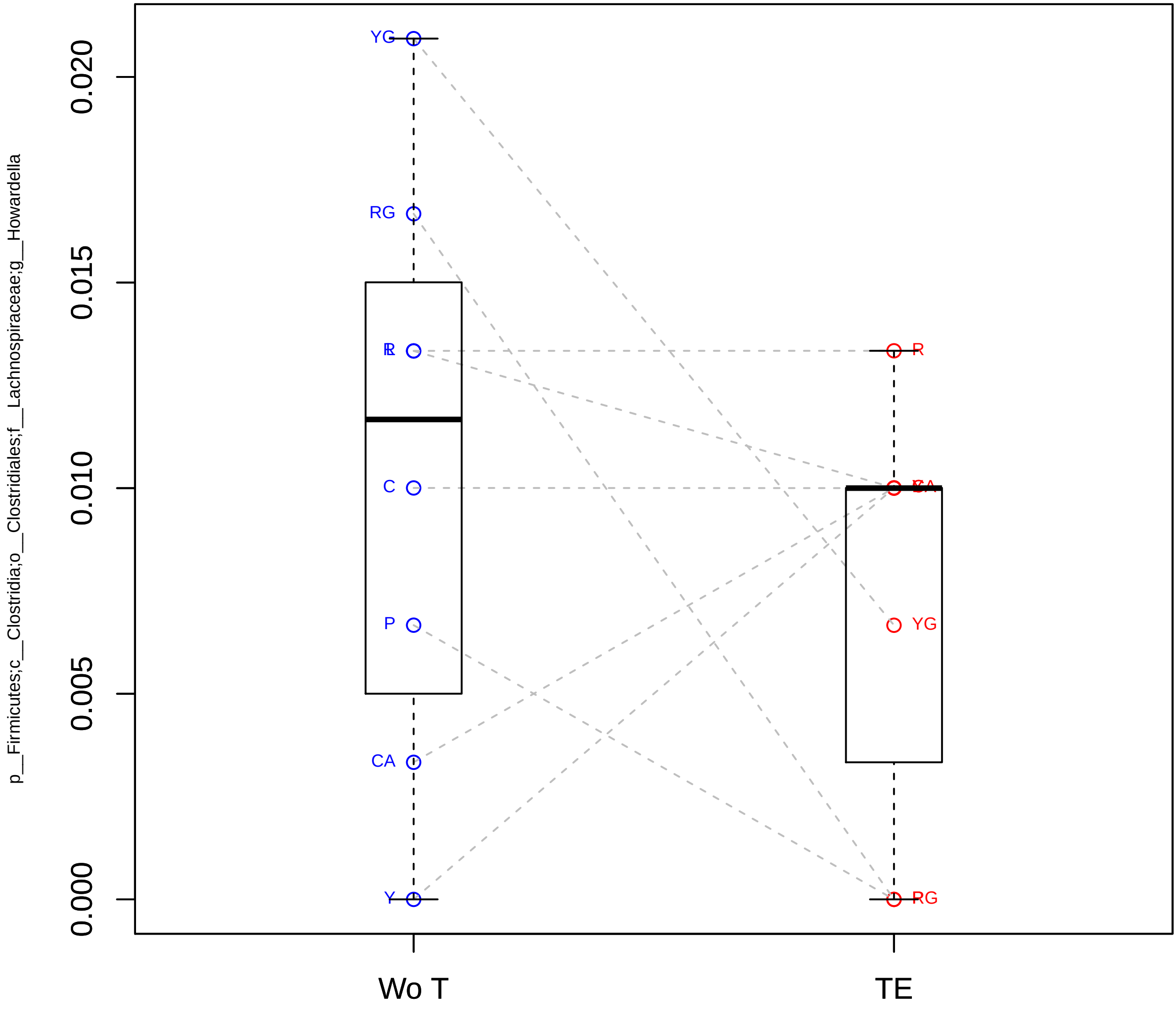

p−value: 0.46 adj. p−value 0.66

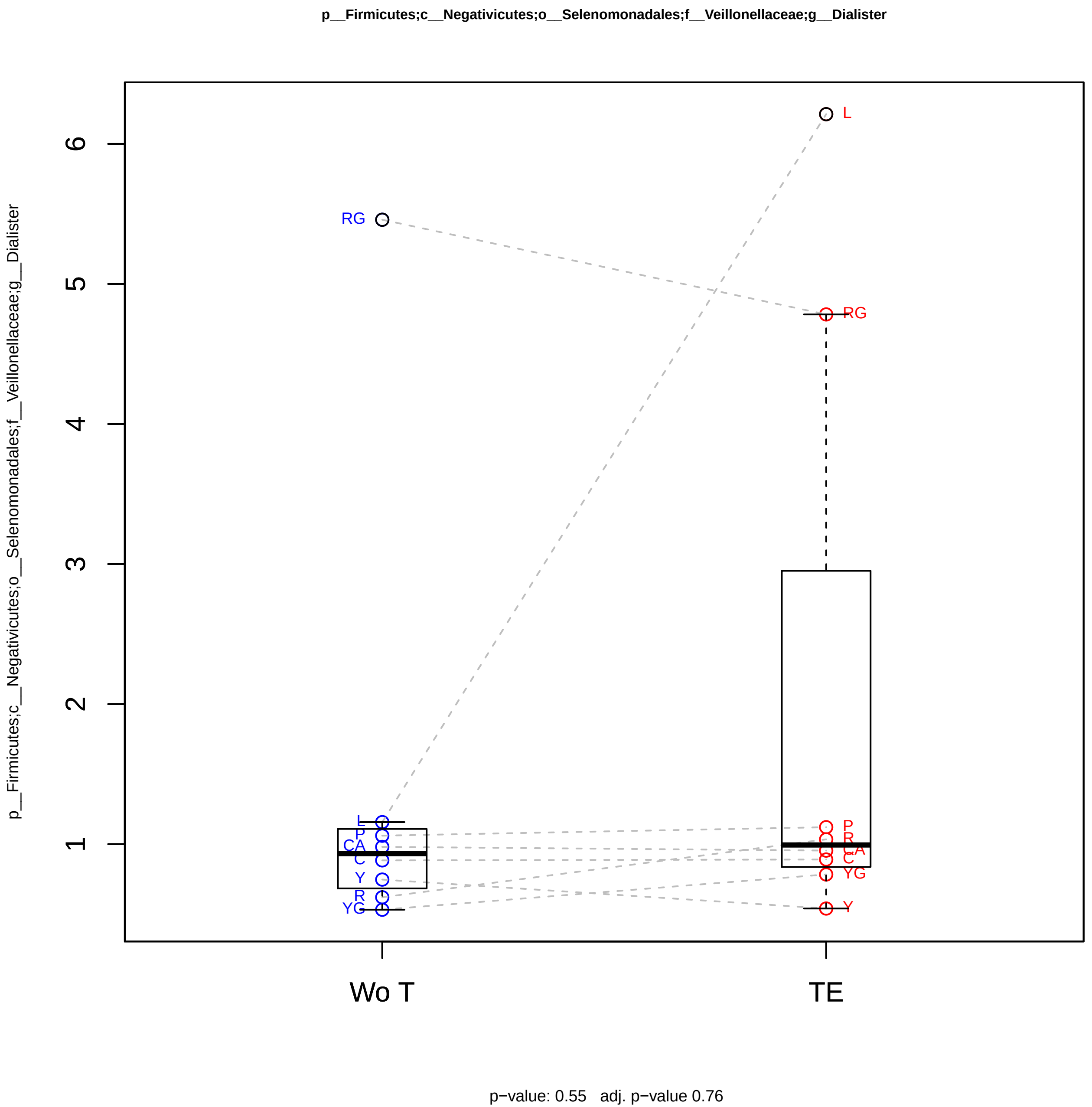
p__Firmicutes;c__Negativicutes;o__Selenomonadales;f__Veillonellaceae;g__Dialister
p__Firmicutes;c__Negativicutes;o__Selenomonadales;f__Veillonellaceae;g__Dialister
1
2
3
4
5
6
Wo T
TE
RG
L
P
CA
C
Y
R
YG
p−value: 0.55 adj. p−value 0.76

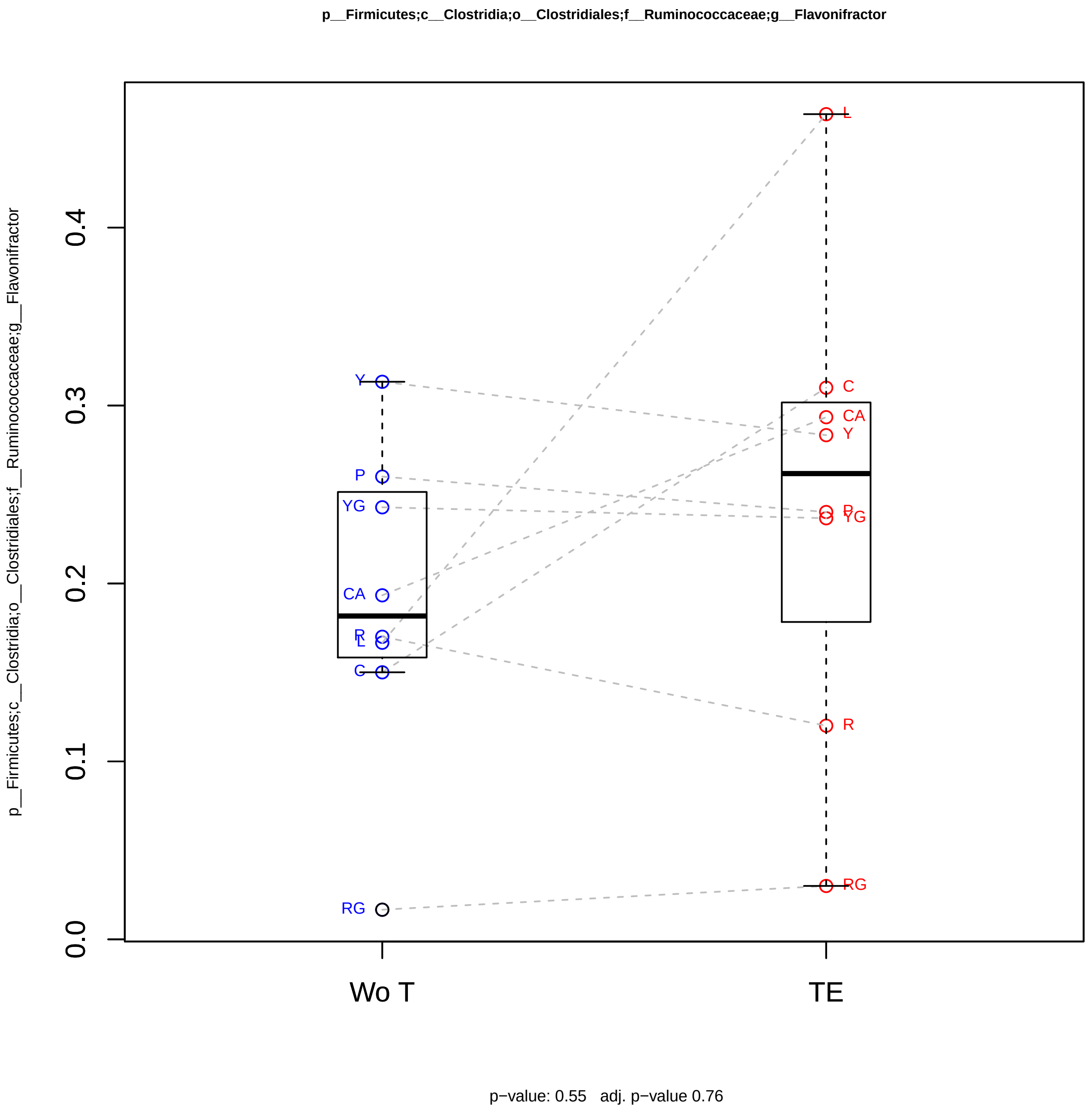
p__Firmicutes;c__Clostridia;o__Clostridiales;f__Ruminococcaceae;g__Flavonifractor
p__Firmicutes;c__Clostridia;o__Clostridiales;f__Ruminococcaceae;g__Flavonifractor
0.0
0.1
0.2
0.3
0.4
Wo T
TE
Y
P
YG
CA
R
L
C
RG
L
C
CA
Y
P
YG
R
RG
p−value: 0.55 adj. p−value 0.76

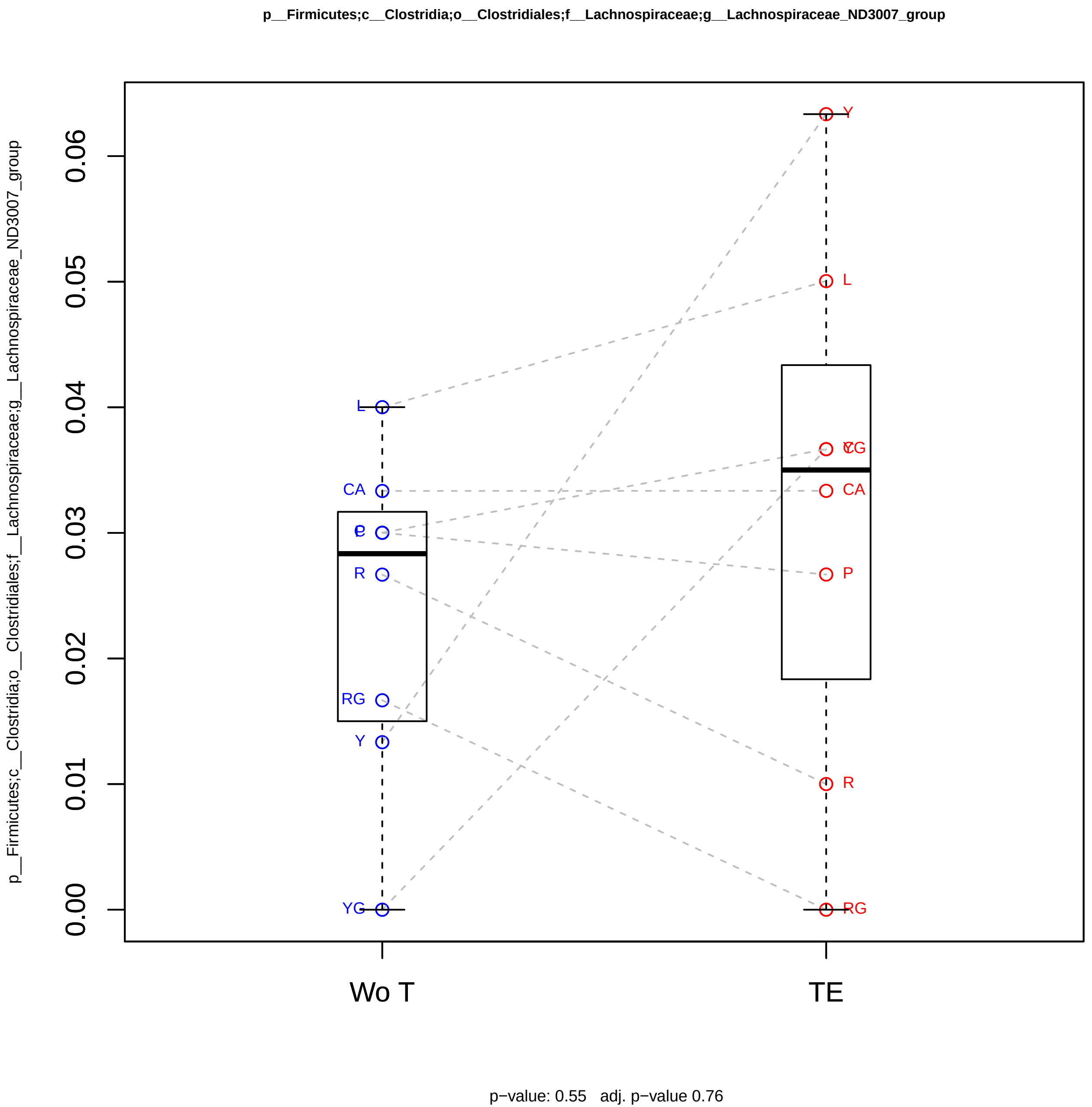

p__Firmicutes;c__Clostridia;o__Clostridiales;f__Lachnospiraceae;g__Lachnospiraceae_ND3007_group
p__Firmicutes;c__Clostridia;o__Clostridiales;f__Lachnospiraceae;g__Lachnospiraceae_ND3007_group
0.00
0.01
0.02
0.03
0.04
0.05
0.06
Wo T
TE
L
CA
R
RG
Y
YG
Y
L
CA
P
R
RG
p−value: 0.55 adj. p−value 0.76

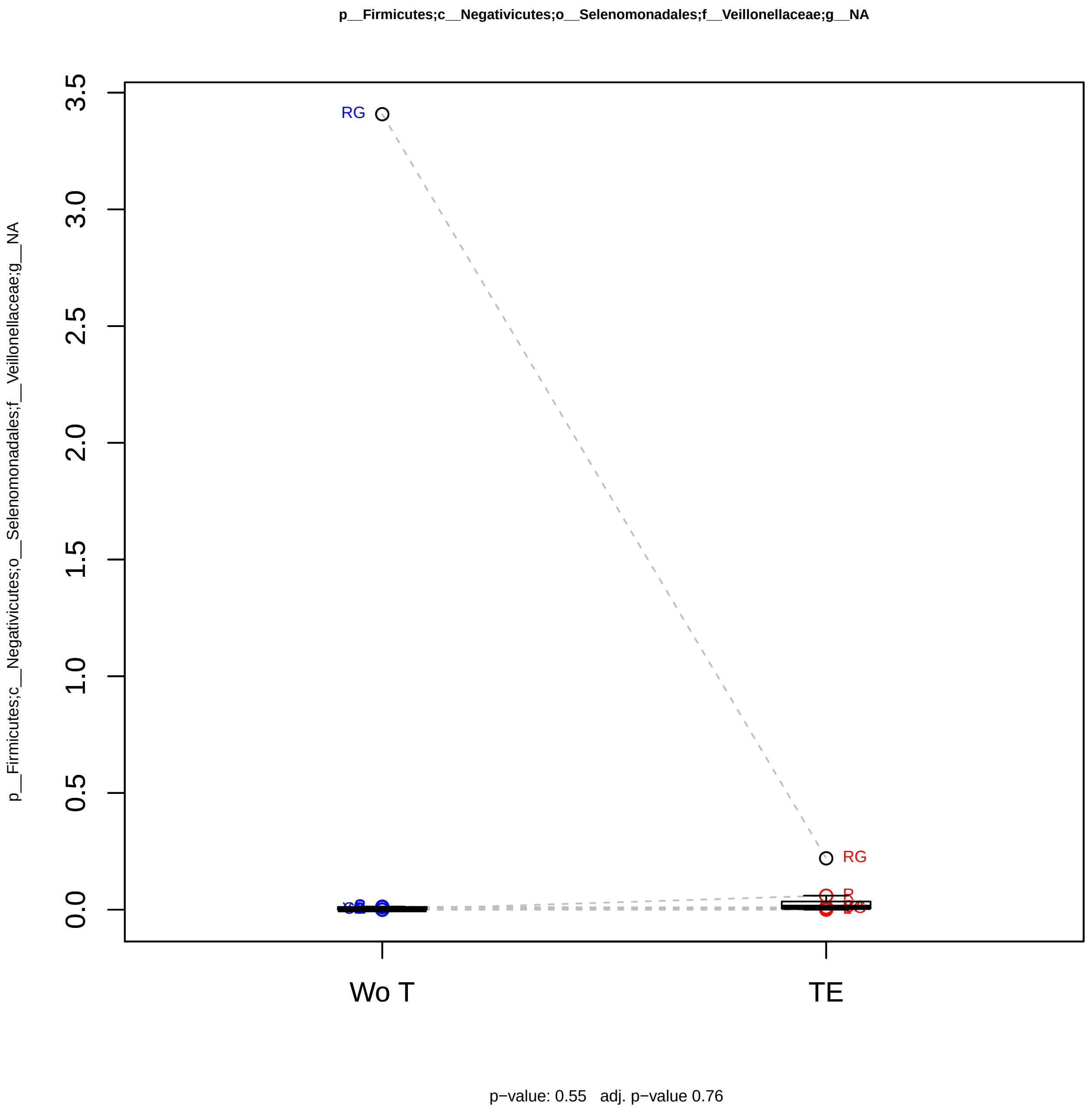
p__Firmicutes;c__Negativicutes;o__Selenomonadales;f__Veillonellaceae;g__NA
p__Firmicutes;c__Negativicutes;o__Selenomonadales;f__Veillonellaceae;g__NA
0.0
0.5
1.0
1.5
2.0
2.5
3.0
3.5
RG
RG
R
Wo T
TE
p−value: 0.55 adj. p−value 0.76

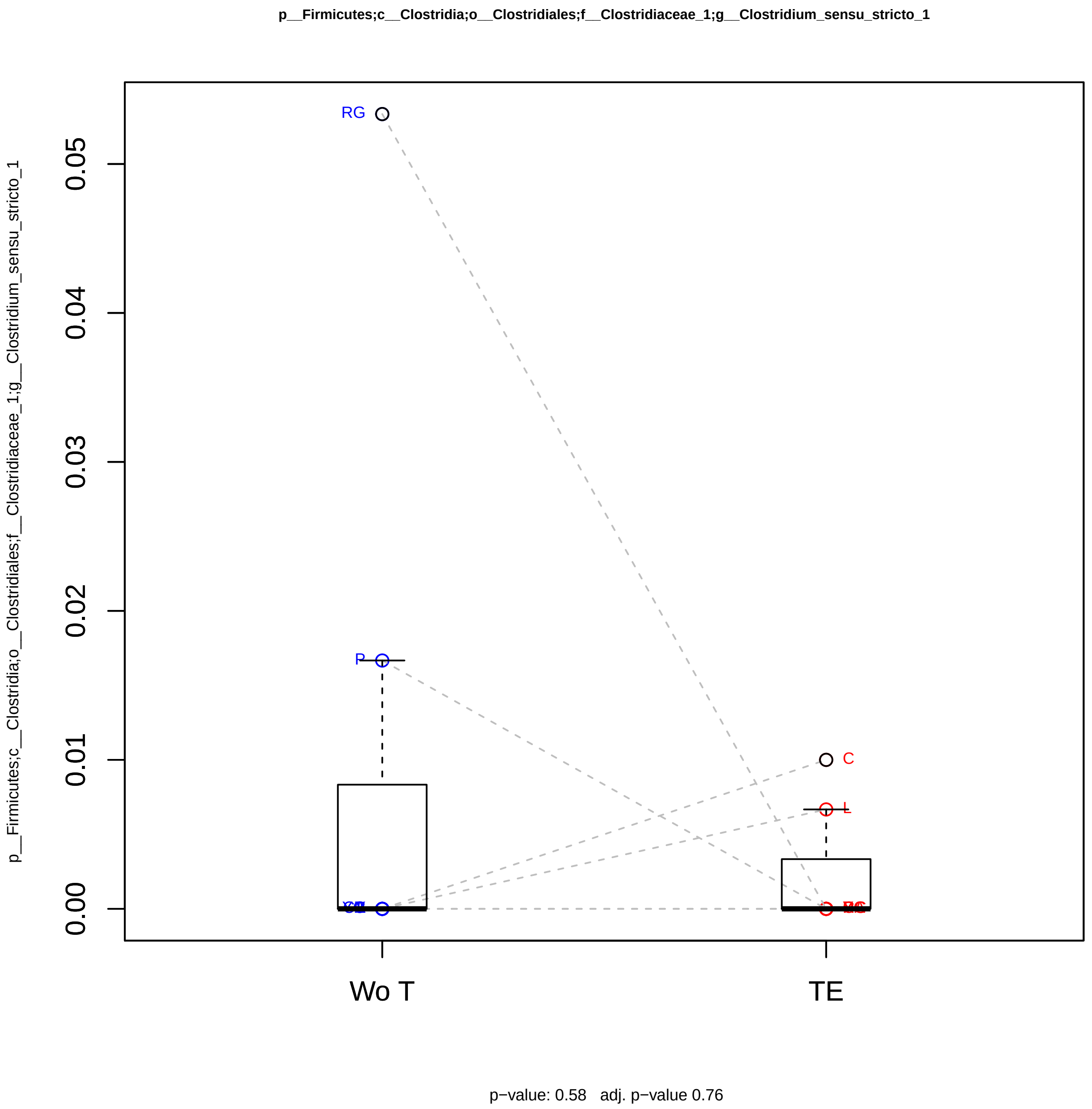
p__Firmicutes;c__Clostridia;o__Clostridiales;f__Clostridiaceae_1;g__Clostridium_sensu_stricto_1
p__Firmicutes;c__Clostridia;o__Clostridiales;f__Clostridiaceae_1;g__Clostridium_sensu_stricto_1
0.00
0.01
0.02
0.03
0.04
0.05
RG
P
C
L
Wo T
TE
p−value: 0.58 adj. p−value 0.76

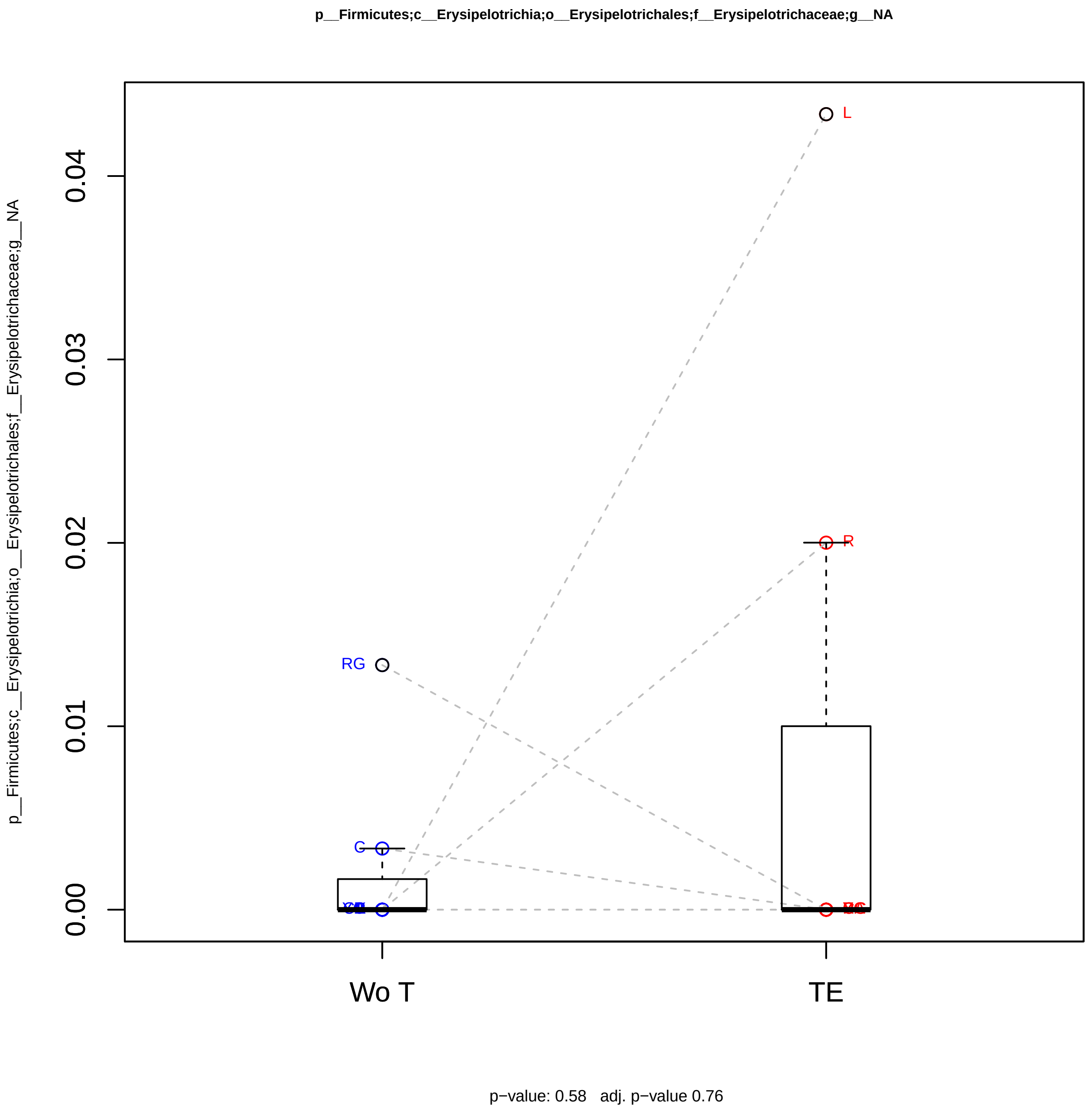
p__Firmicutes;c__Erysipelotrichia;o__Erysipelotrichales;f__Erysipelotrichaceae;g__NA
p__Firmicutes;c__Erysipelotrichia;o__Erysipelotrichales;f__Erysipelotrichaceae;g__NA
0.00
0.01
0.02
0.03
0.04
Wo T
TE
RG
C
L
R
p−value: 0.58 adj. p−value 0.76

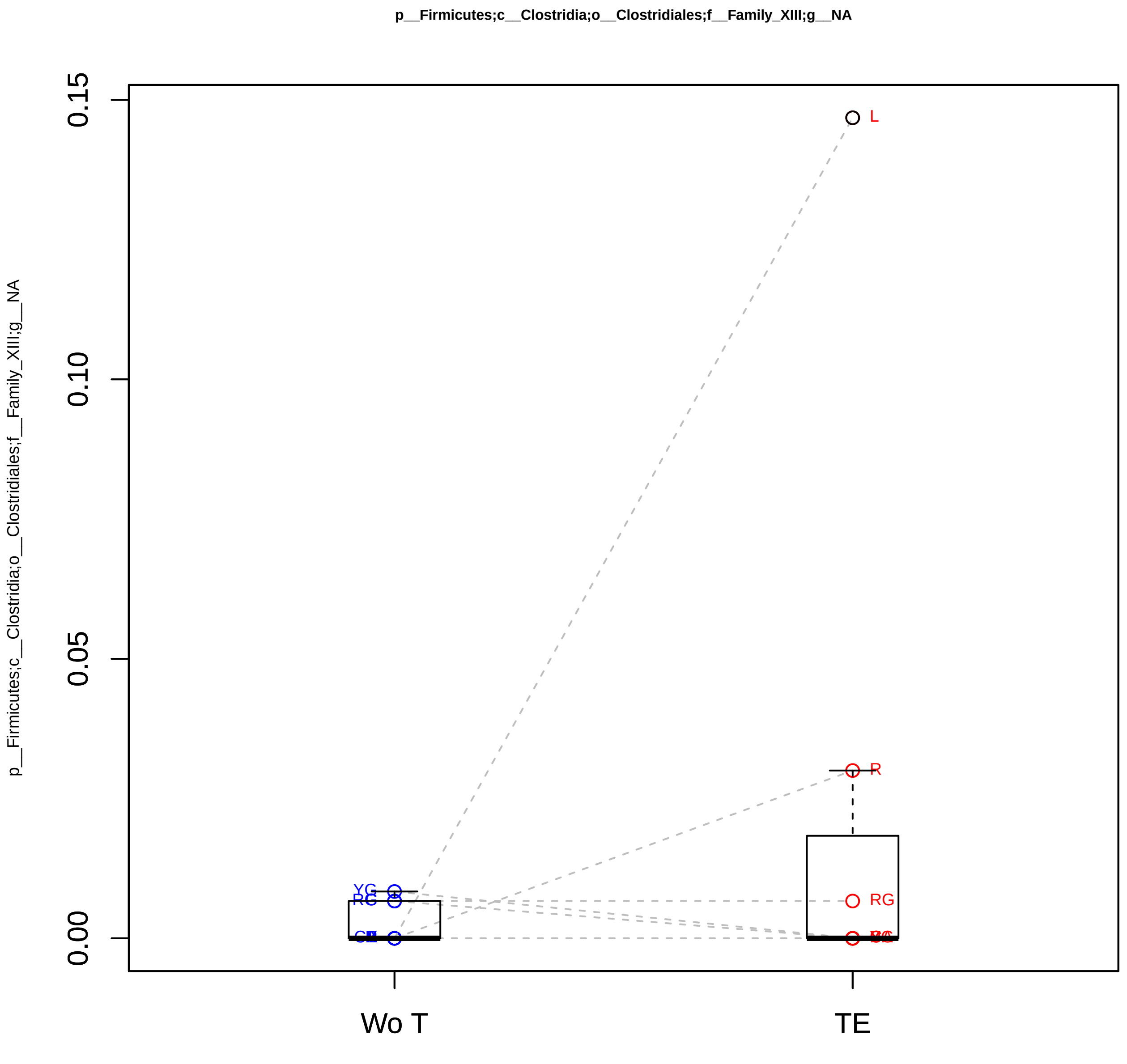

p−value: 0.59 adj. p−value 0.76

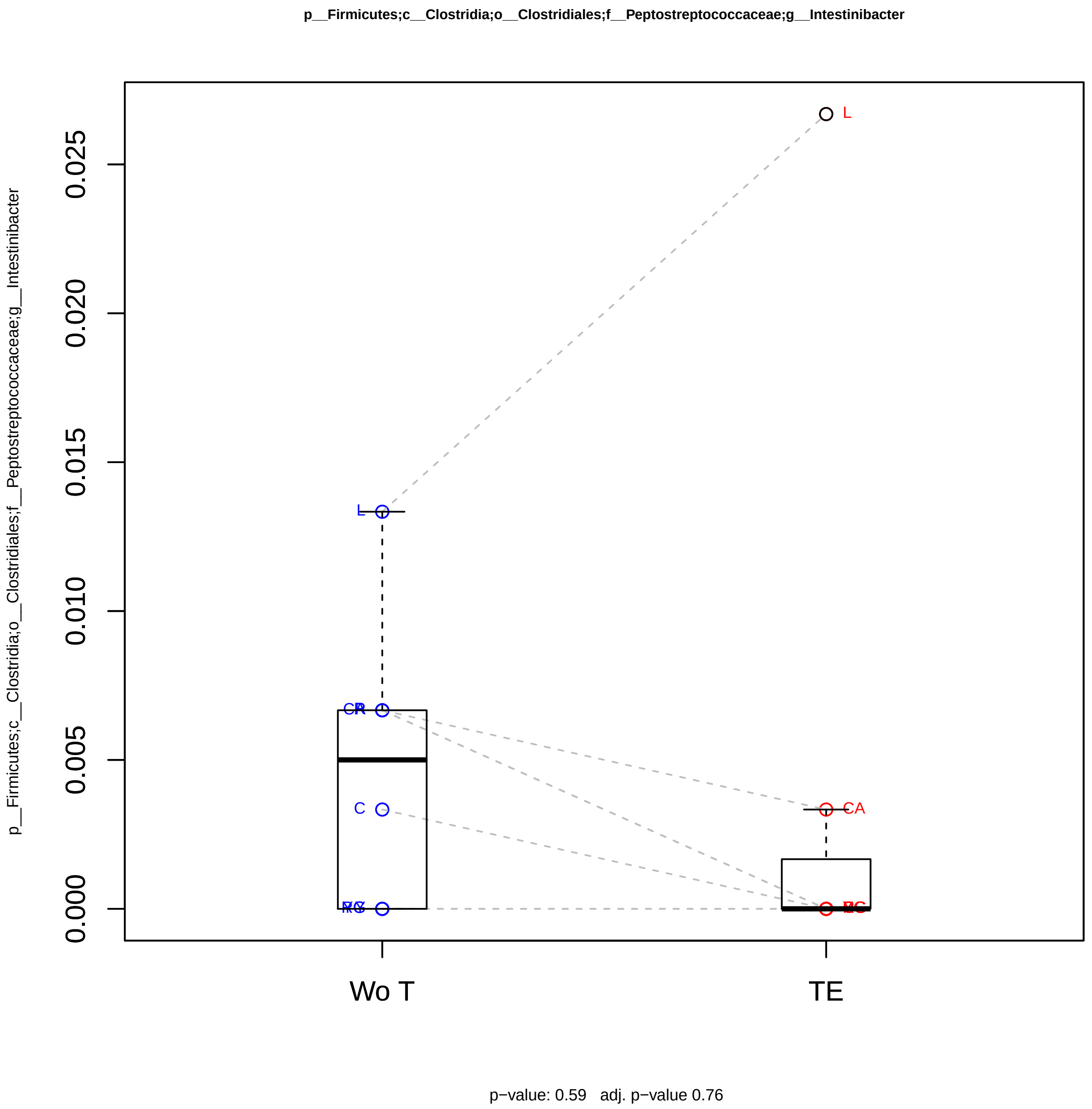
p__Firmicutes;c__Clostridia;o__Clostridiales;f__Peptostreptococcaceae;g__Intestinibacter
p__Firmicutes;c__Clostridia;o__Clostridiales;f__Peptostreptococcaceae;g__Intestinibacter
0.000
0.005
0.010
0.015
0.020
0.025
Wo T
TE
L
CA
C
p−value: 0.59 adj. p−value 0.76

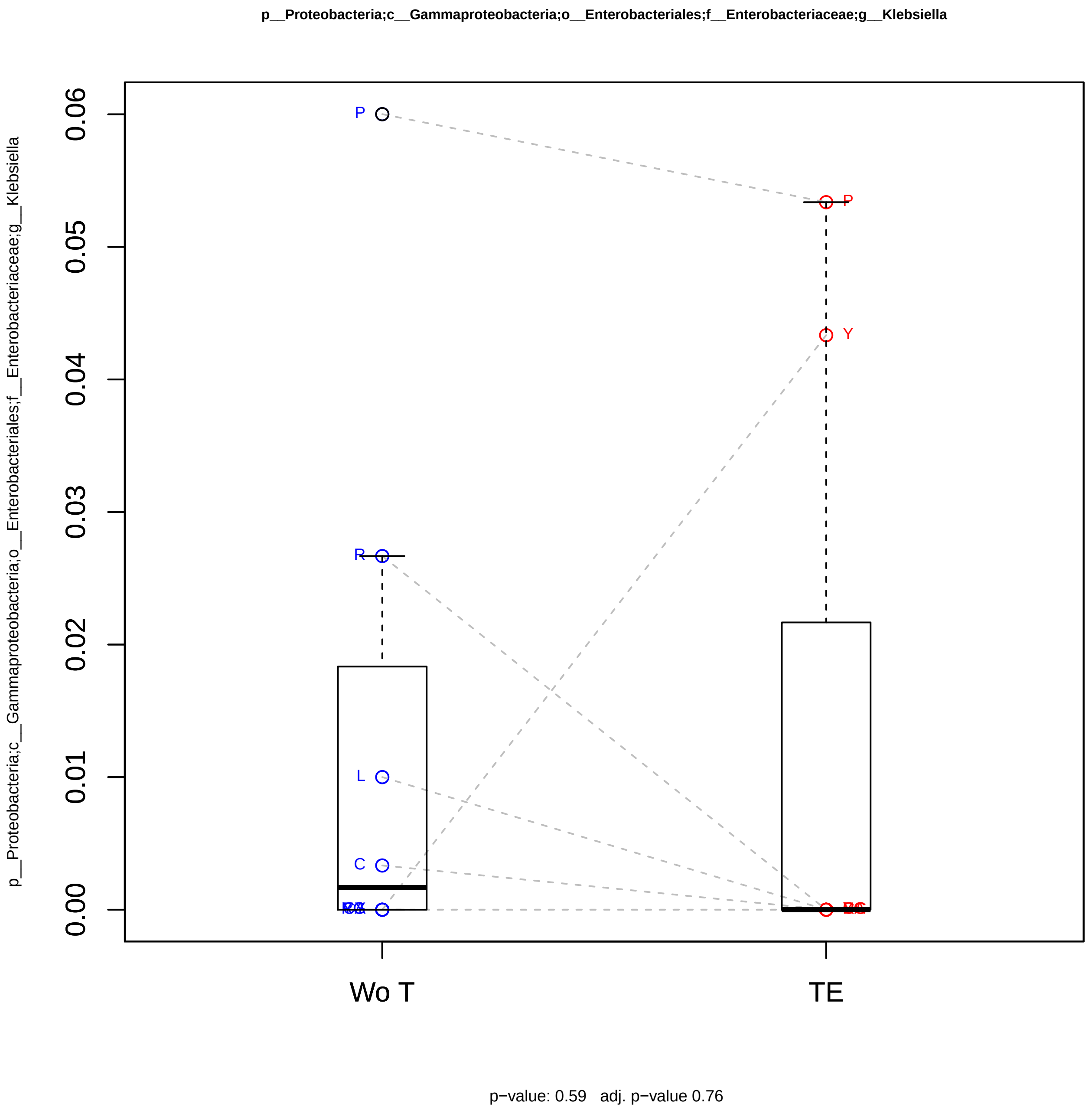
p__Proteobacteria;c__Gammaproteobacteria;o__Enterobacteriales;f__Enterobacteriaceae;g__Klebsiella
p__Proteobacteria;c__Gammaproteobacteria;o__Enterobacteriales;f__Enterobacteriaceae;g__Klebsiella
0.00
0.01
0.02
0.03
0.04
0.05
0.06
P
R
L
C
Y
Wo T
TE
p−value: 0.59 adj. p−value 0.76

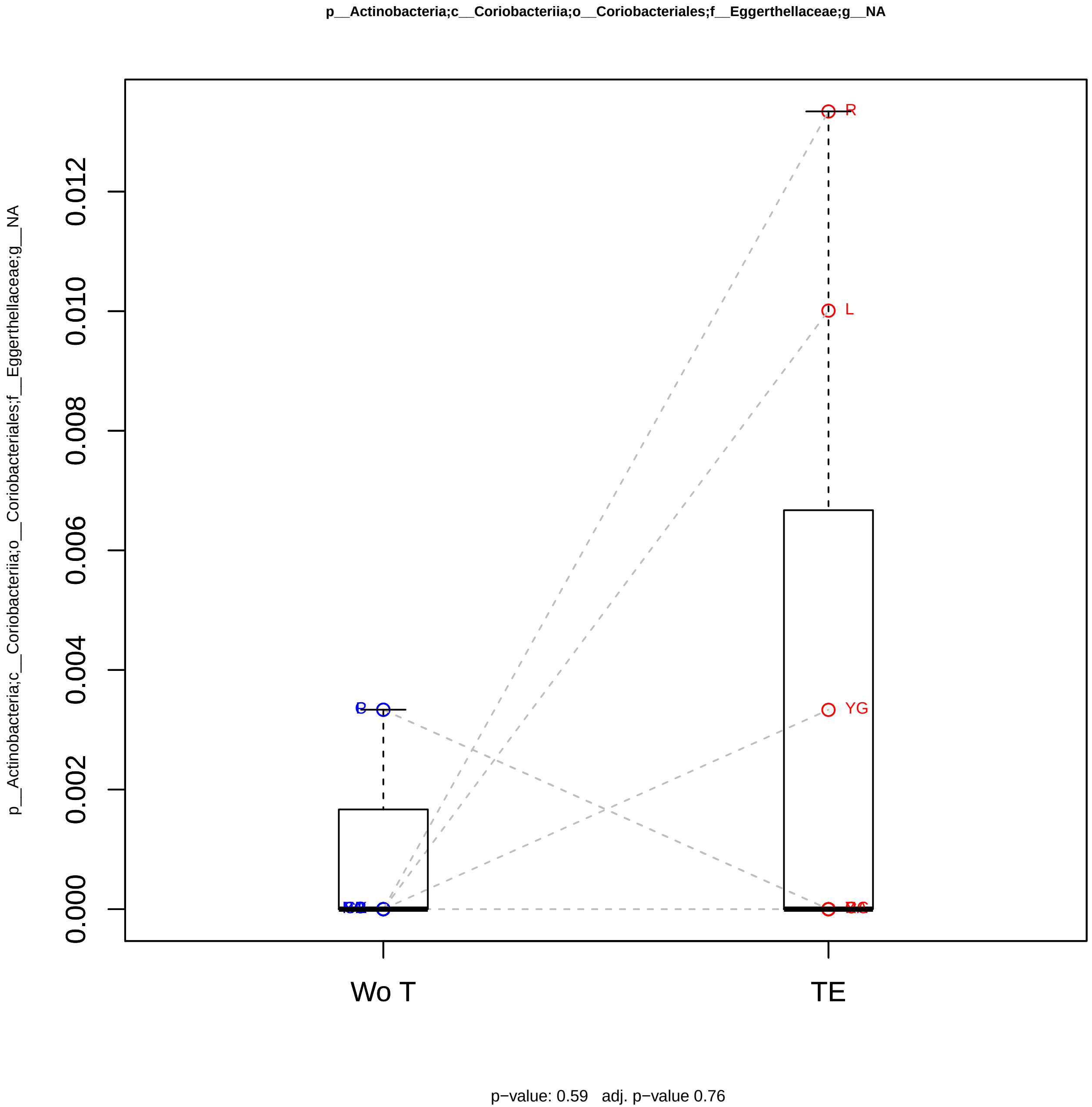

p__Actinobacteria;c__Coriobacteriia;o__Coriobacteriales;f__Eggerthellaceae;g__NA
p__Actinobacteria;c__Coriobacteriia;o__Coriobacteriales;f__Eggerthellaceae;g__NA
0.000
0.002
0.004
0.006
0.008
0.010
0.012
R
L
YG
Wo T
TE
p−value: 0.59 adj. p−value 0.76

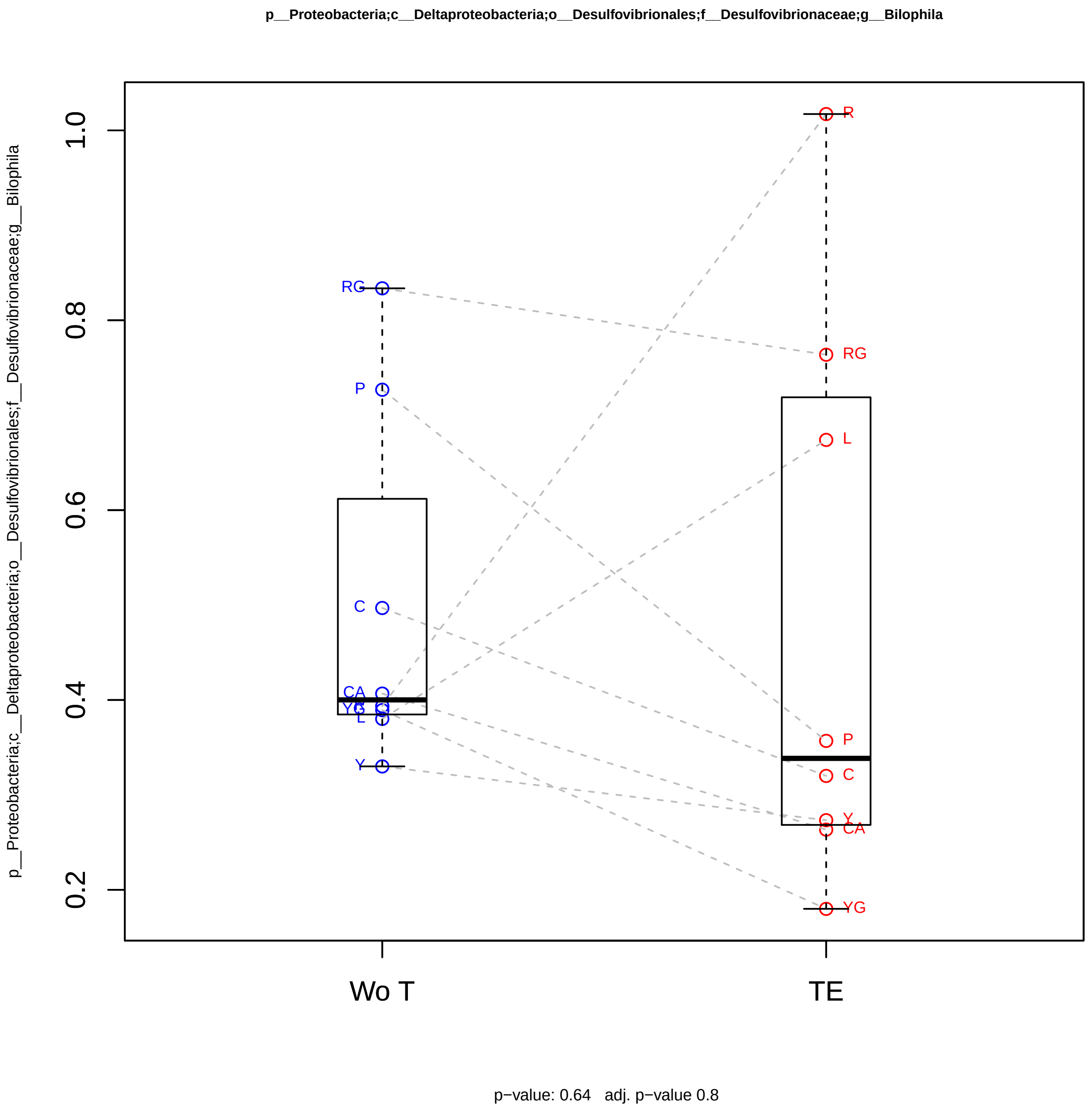
p__Proteobacteria;c__Deltaproteobacteria;o__Desulfovibrionales;f__Desulfovibrionaceae;g__Bilophila
p__Proteobacteria;c__Deltaproteobacteria;o__Desulfovibrionales;f__Desulfovibrionaceae;g__Bilophila
0.2
0.4
0.6
0.8
1.0
Wo T
TE
RG
P
C
CA
YG
L
Y
R
RG
L
P
C
Y
CA
YG
p−value: 0.64 adj. p−value 0.8

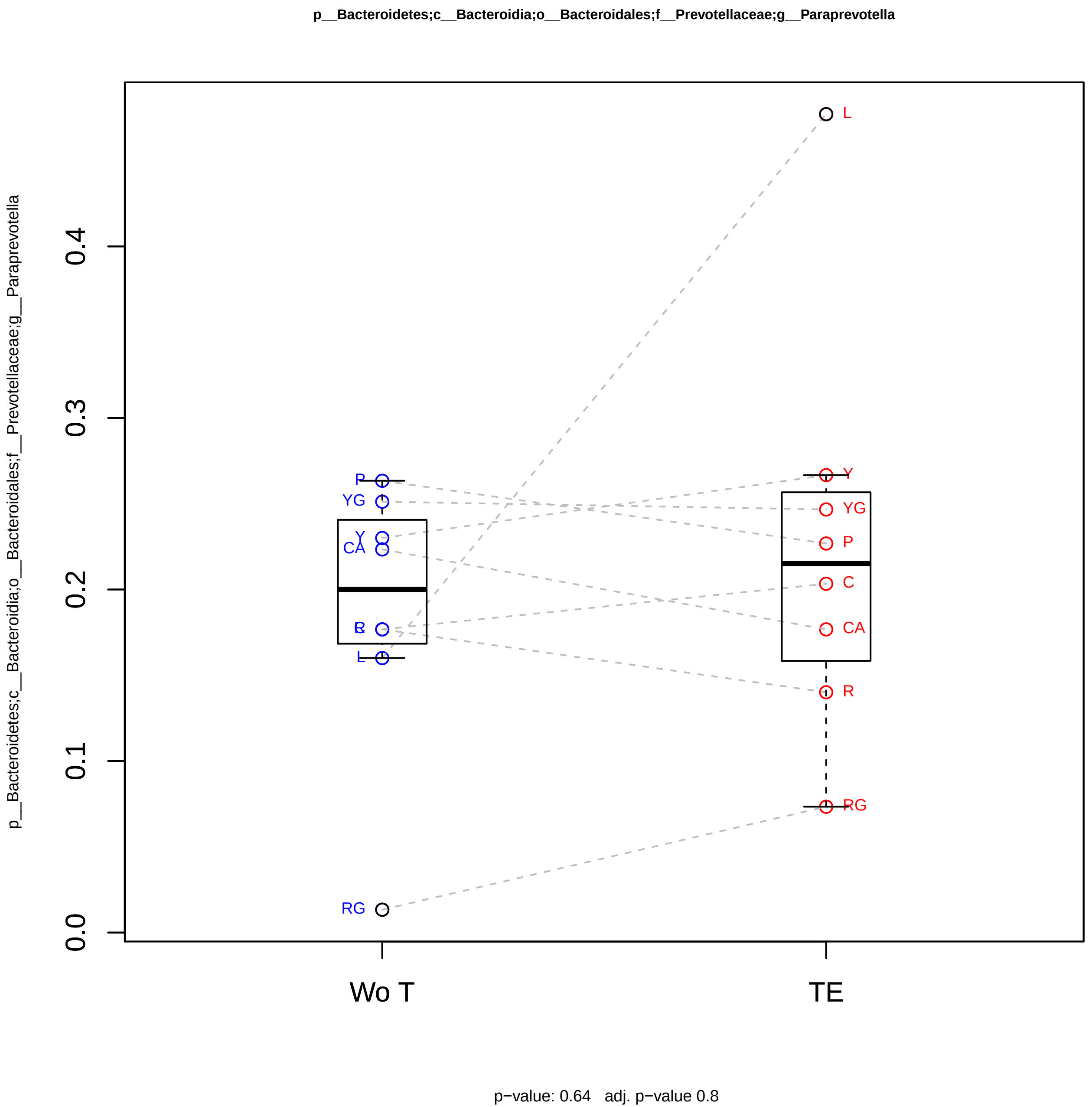
p__Bacteroidetes;c__Bacteroidia;o__Bacteroidales;f__Prevotellaceae;g__Paraprevotella
p__Bacteroidetes;c__Bacteroidia;o__Bacteroidales;f__Prevotellaceae;g__Paraprevotella
0.0
0.1
0.2
0.3
0.4
Wo T
TE
P
YG
Y
CA
R
C
L
RG
p−value: 0.64 adj. p−value 0.8

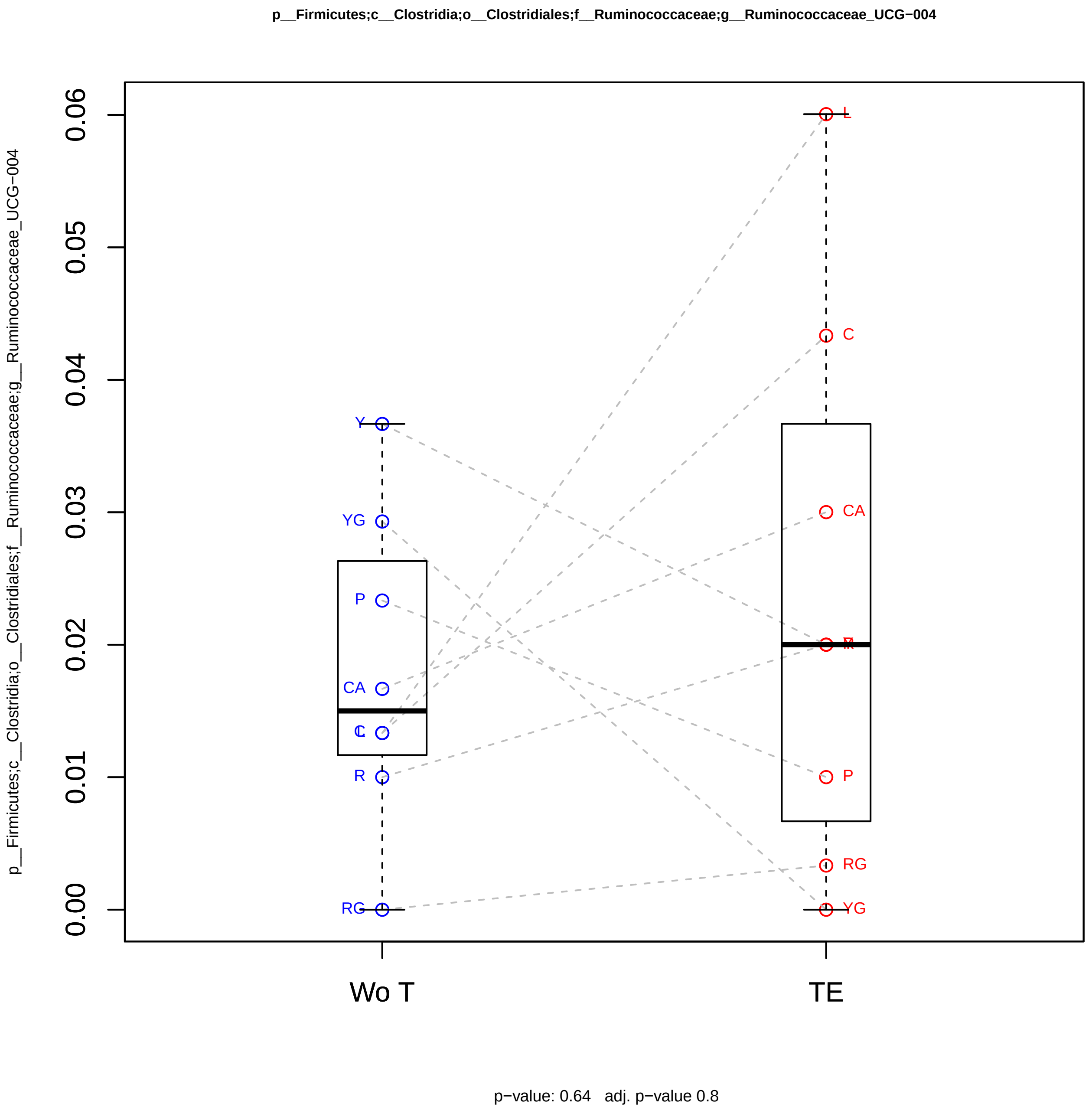

p__Firmicutes;c__Clostridia;o__Clostridiales;f__Ruminococcaceae;g__Ruminococcaceae_UCG−004
p__Firmicutes;c__Clostridia;o__Clostridiales;f__Ruminococcaceae;g__Ruminococcaceae_UCG−004
0.00
0.01
0.02
0.03
0.04
0.05
0.06
Y
YG
P
CA
C
L
R
RG
L
C
CA
P
RG
YG
Wo T
TE
p−value: 0.64 adj. p−value 0.8

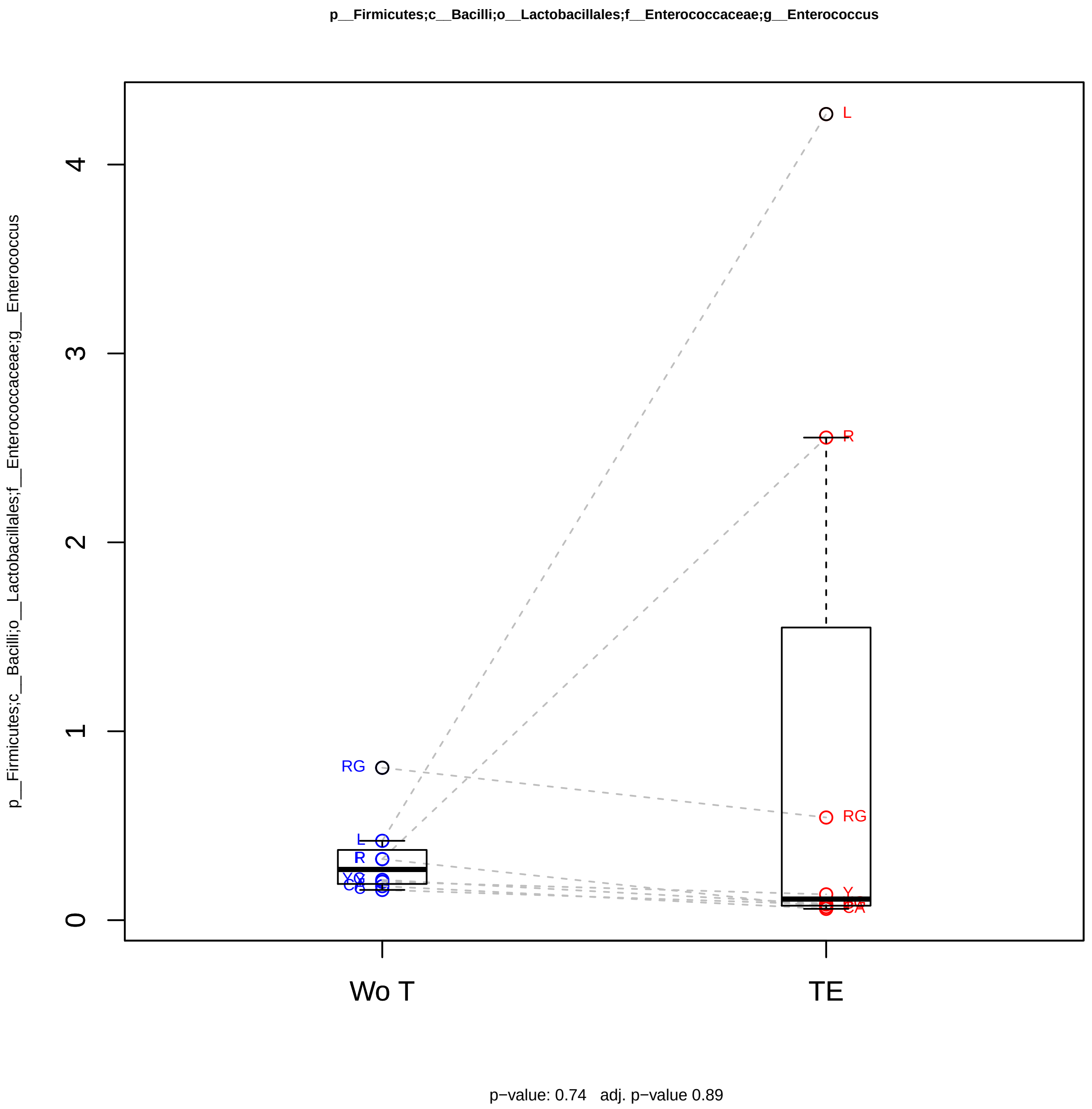
p__Firmicutes;c__Bacilli;o__Lactobacillales;f__Enterococcaceae;g__Enterococcus
p__Firmicutes;c__Bacilli;o__Lactobacillales;f__Enterococcaceae;g__Enterococcus
0
1
2
3
4
Wo T
TE
RG
L
R
L
R
RG
Y
CA
p−value: 0.74 adj. p−value 0.89

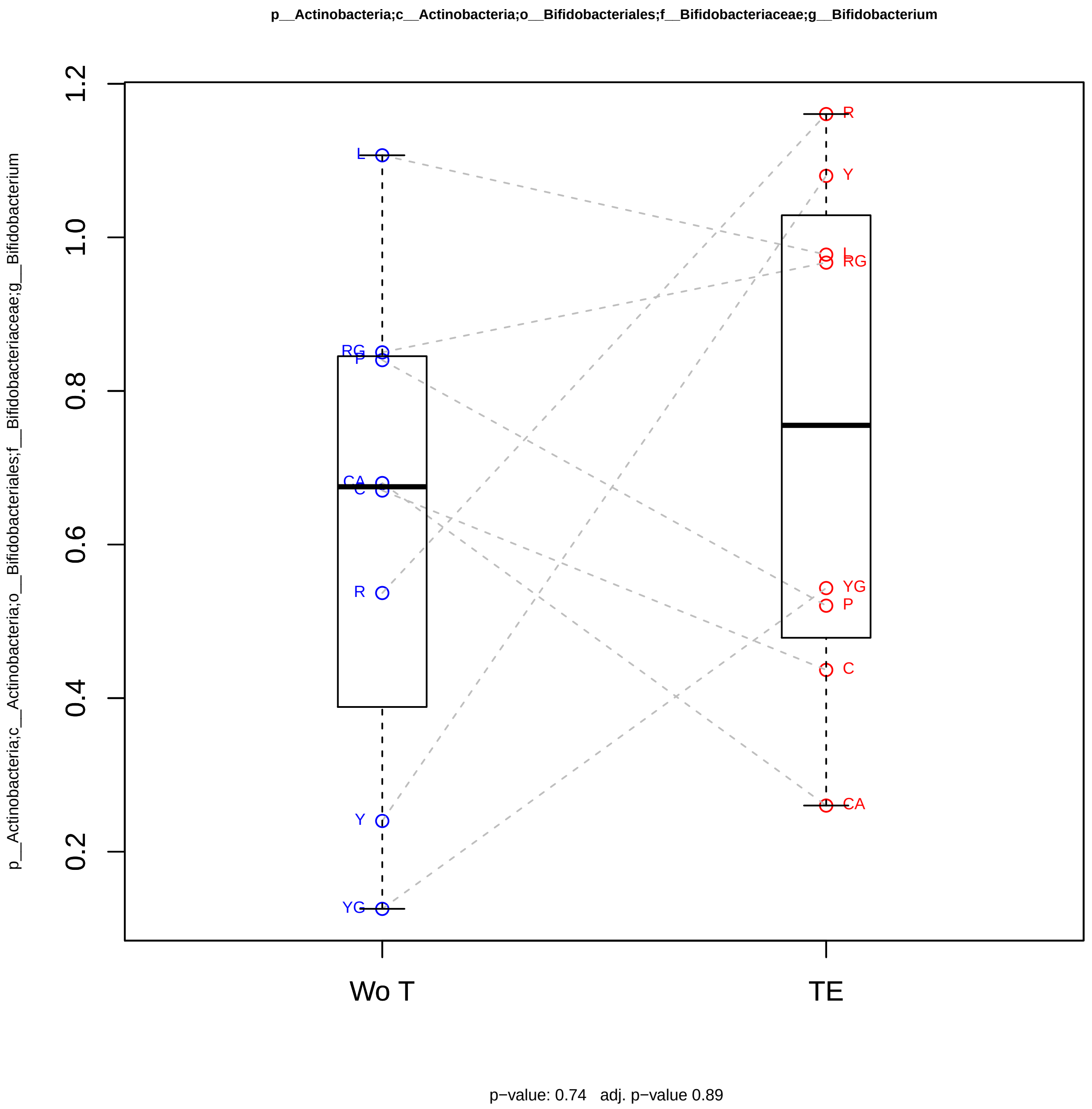

p__Actinobacteria;c__Actinobacteria;o__Bifidobacteriales;f__Bifidobacteriaceae;g__Bifidobacterium
p__Actinobacteria;c__Actinobacteria;o__Bifidobacteriales;f__Bifidobacteriaceae;g__Bifidobacterium
1.2
1.0
0.8
0.6
0.4
0.2
L
RG
P
CA
C
R
Y
YG
R
Y
L
RG
YG
P
C
CA
Wo T
TE
p−value: 0.74 adj. p−value 0.89

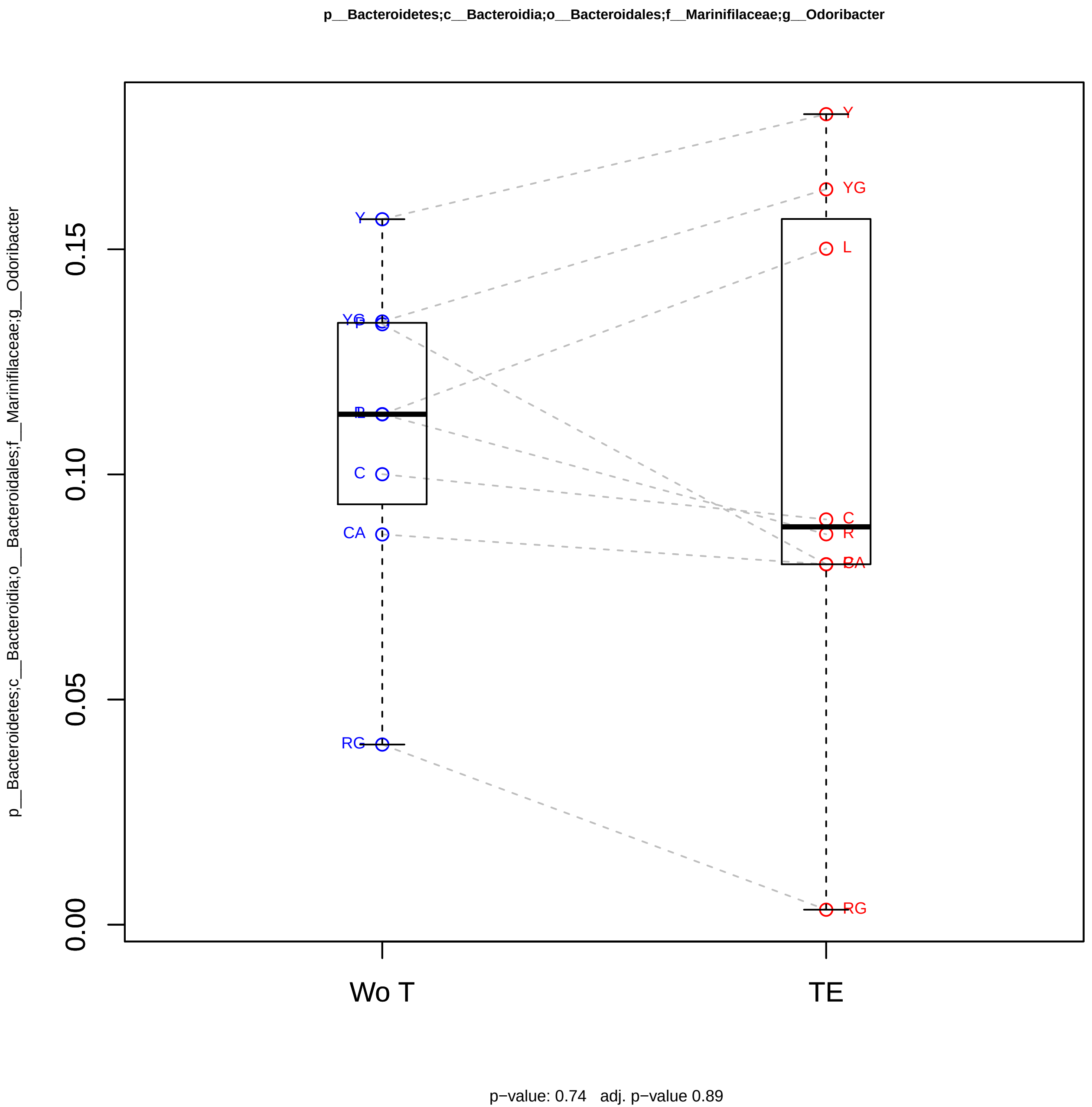
p__Bacteroidetes;c__Bacteroidia;o__Bacteroidales;f__Marinifilaceae;g__Odoribacter
p__Bacteroidetes;c__Bacteroidia;o__Bacteroidales;f__Marinifilaceae;g__Odoribacter
0.00
0.05
0.10
0.15
Wo T
TE
Y
YG
P
L
R
C
CA
RG
p−value: 0.74 adj. p−value 0.89

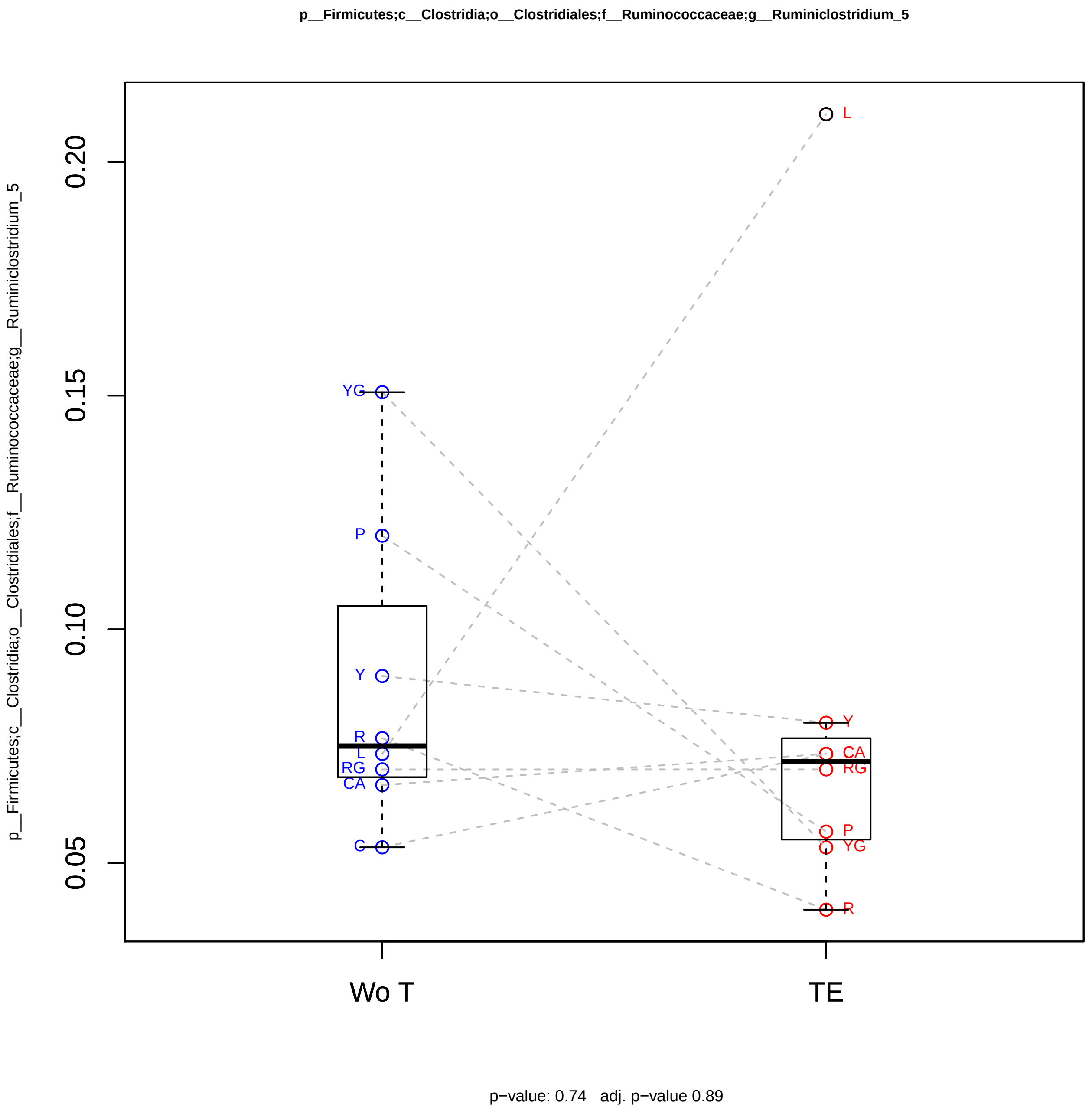
p__Firmicutes;c__Clostridia;o__Clostridiales;f__Ruminococcaceae;g__Ruminiclostridium_5
p__Firmicutes;c__Clostridia;o__Clostridiales;f__Ruminococcaceae;g__Ruminiclostridium_5
0.05
0.10
0.15
0.20
YG
P
Y
R
L
RG
CA
C
L
Y
CA
RG
P
YG
R
Wo T
TE
p−value: 0.74 adj. p−value 0.89

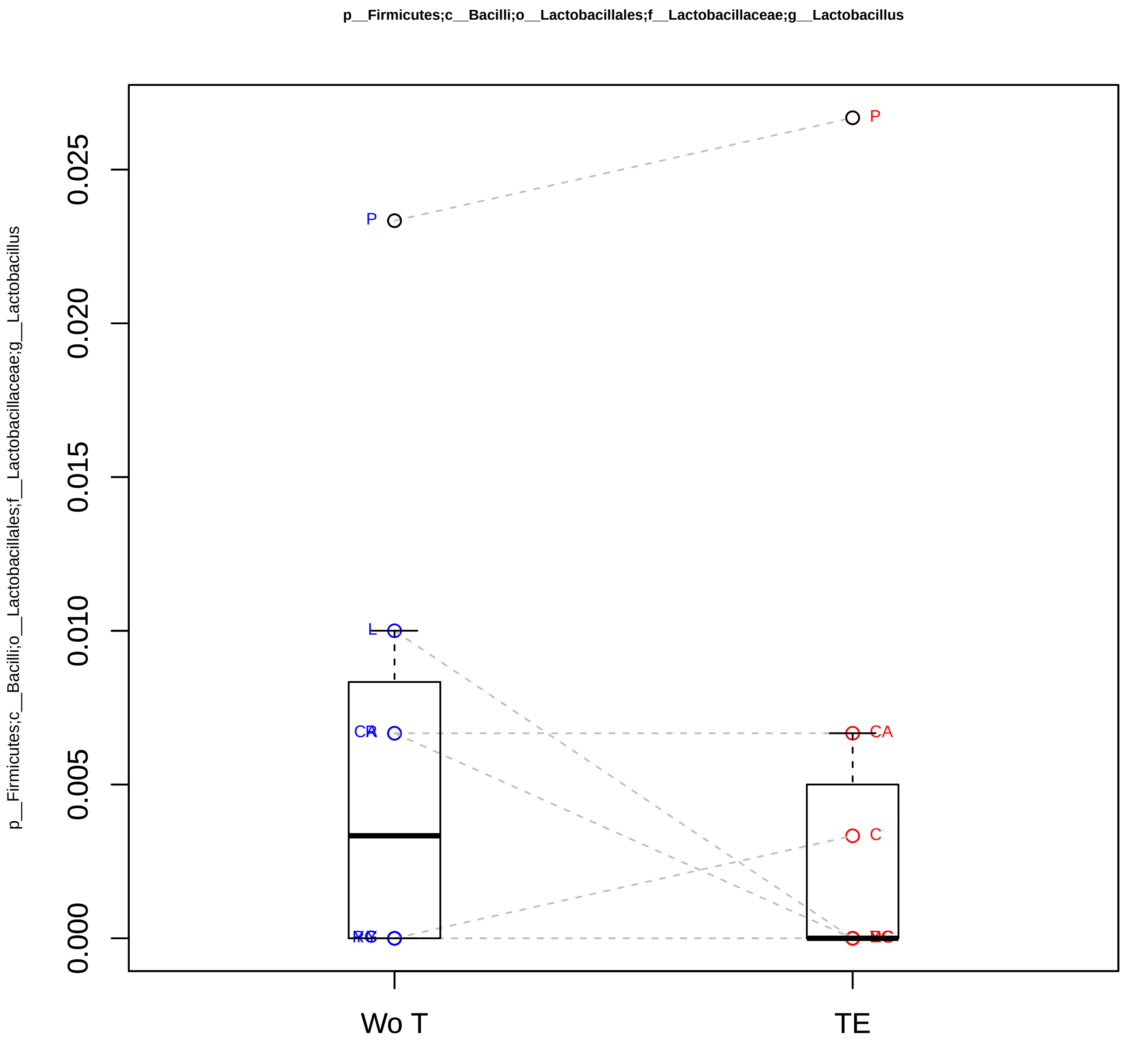

p−value: 0.79 adj. p−value 0.93

p__Firmicutes;c__Clostridia;o__Clostridiales;f__Ruminococcaceae;g__Caproiciproducens

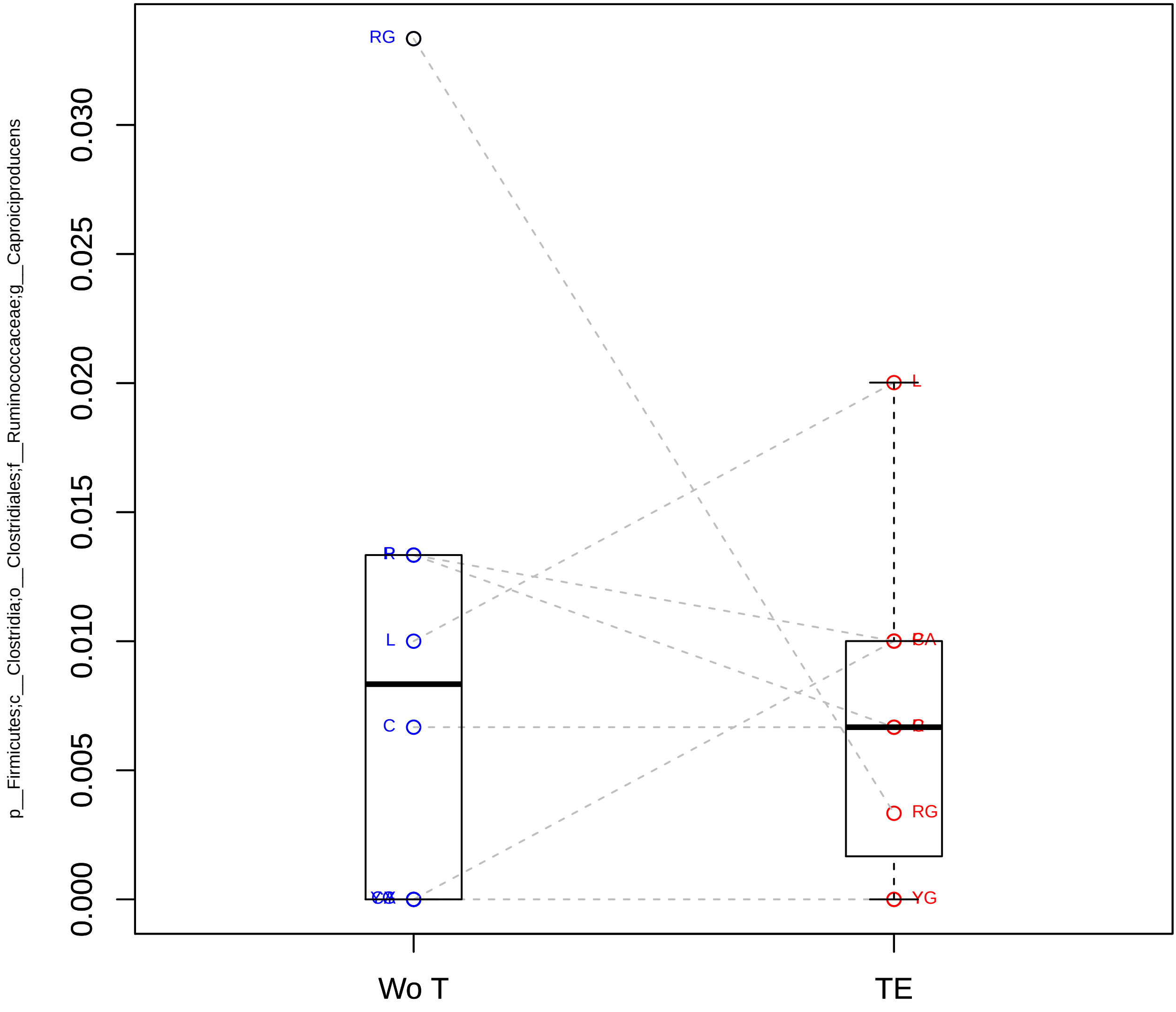

p−value: 0.83   adj. p−value 0.97

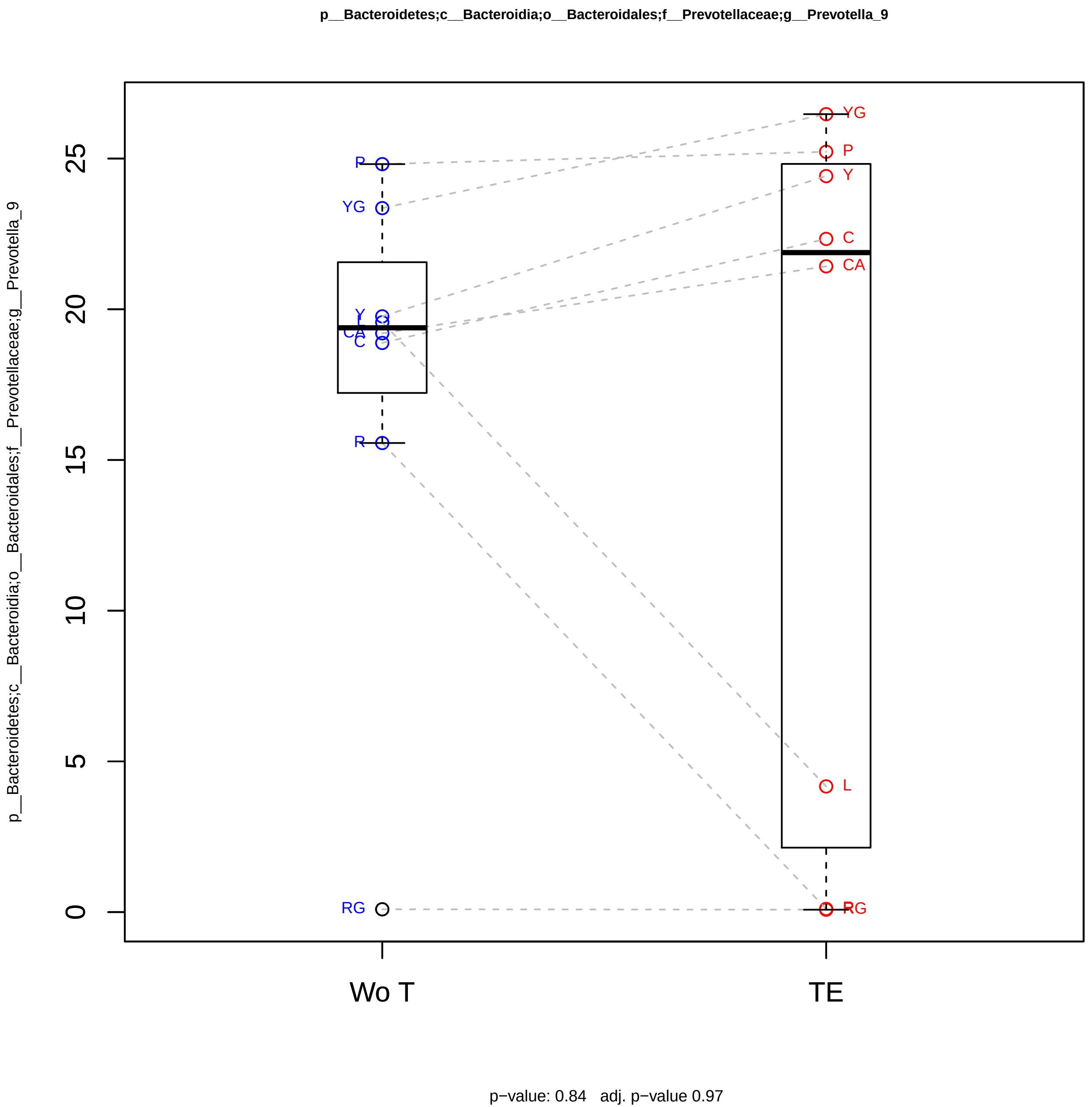
p__Bacteroidetes;c__Bacteroidia;o__Bacteroidales;f__Prevotellaceae;g__Prevotella_9
p__Bacteroidetes;c__Bacteroidia;o__Bacteroidales;f__Prevotellaceae;g__Prevotella_9
0
5
10
15
20
25
P
YG
Y
L
CA
C
R
RG
YG
P
Y
C
CA
L
RG
Wo T
TE
p−value: 0.84 adj. p−value 0.97

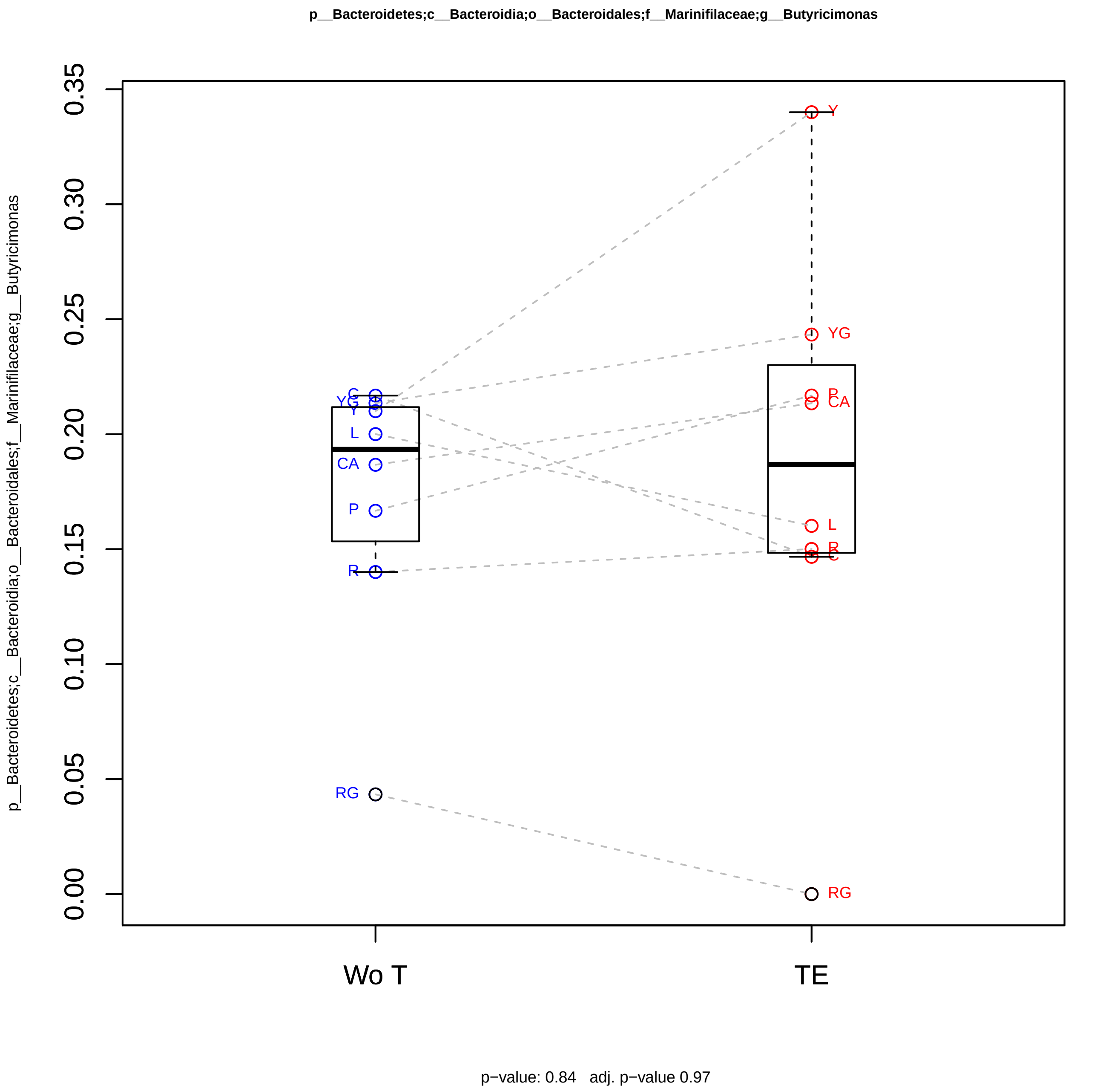
p__Bacteroidetes;c__Bacteroidia;o__Bacteroidales;f__Marinifilaceae;g__Butyricimonas
p__Bacteroidetes;c__Bacteroidia;o__Bacteroidales;f__Marinifilaceae;g__Butyricimonas
0.00
0.05
0.10
0.15
0.20
0.25
0.30
0.35
C
YG
Y
L
CA
P
R
RG
Y
YG
P
CA
L
R
C
RG
Wo T
TE
p−value: 0.84 adj. p−value 0.97

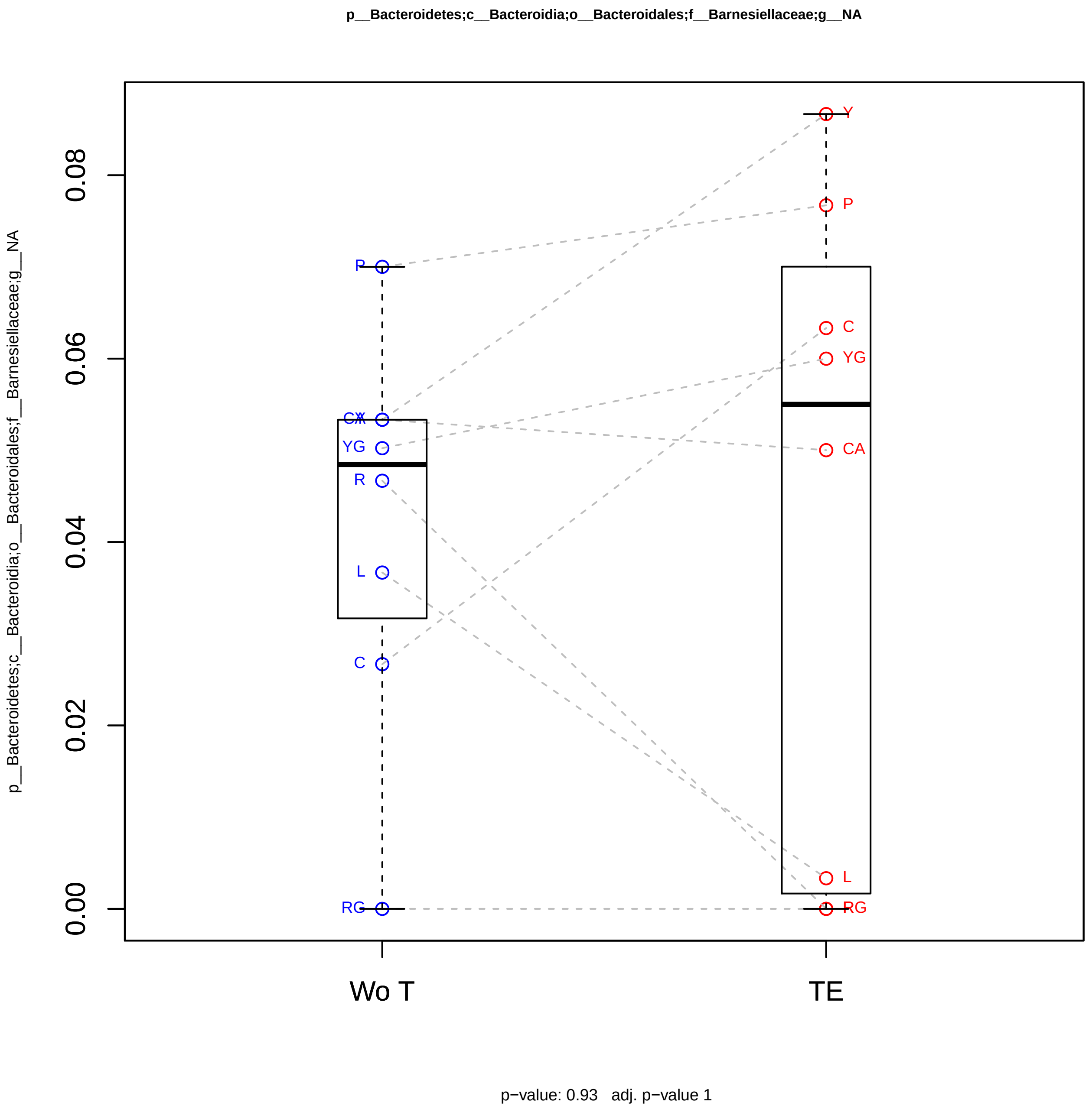
p__Bacteroidetes;c__Bacteroidia;o__Bacteroidales;f__Barnesiellaceae;g__NA
p__Bacteroidetes;c__Bacteroidia;o__Bacteroidales;f__Barnesiellaceae;g__NA
0.00
0.02
0.04
0.06
0.08
P
CA
YG
R
L
C
RG
Y
P
C
YG
CA
L
RG
Wo T
TE
p−value: 0.93   adj. p−value 1

p__Actinobacteria;c__Coriobacteriia;o__Coriobacteriales;f__Eggerthellaceae;g__Eggerthella

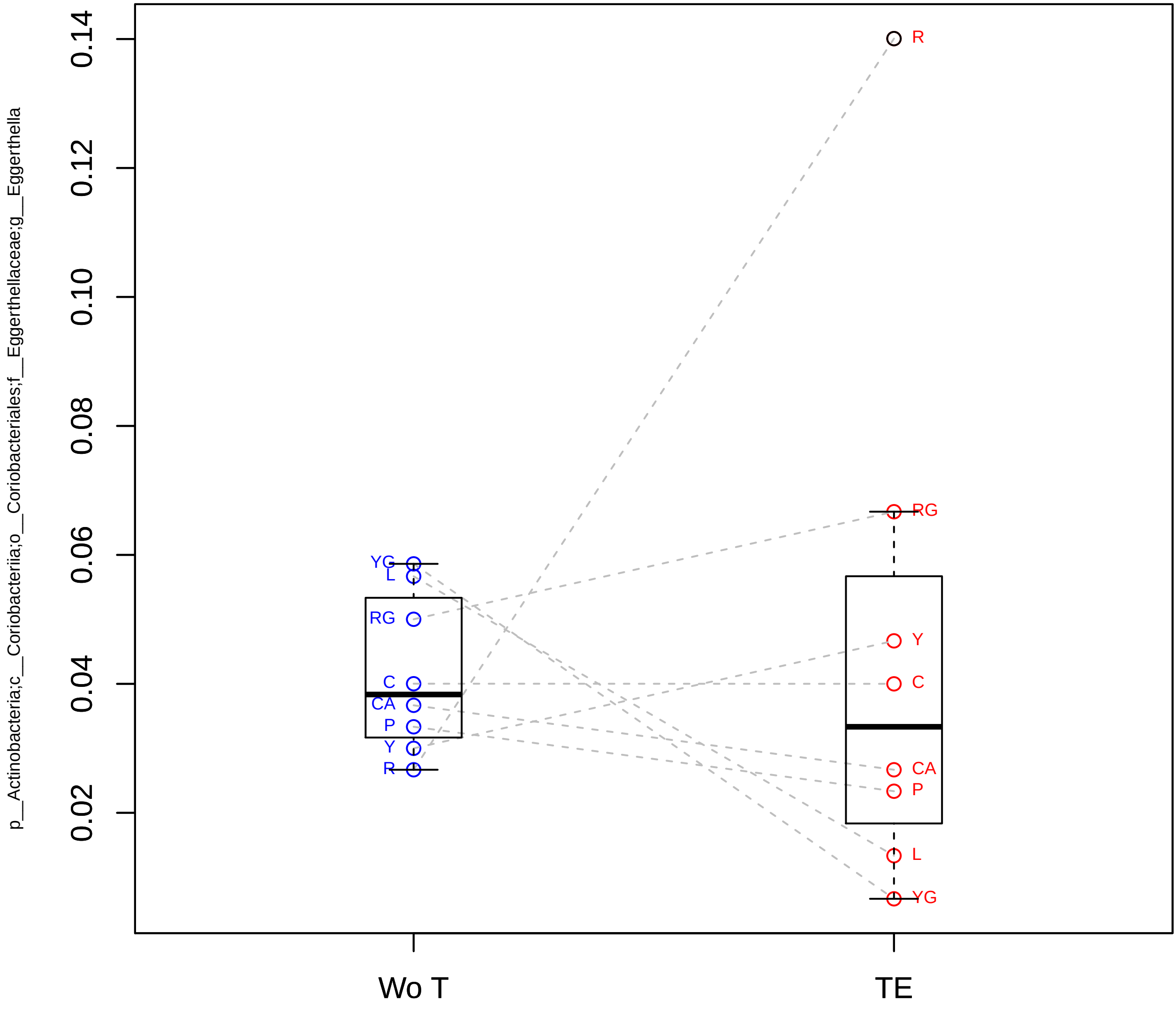

p−value: 0.95 adj. p−value 1

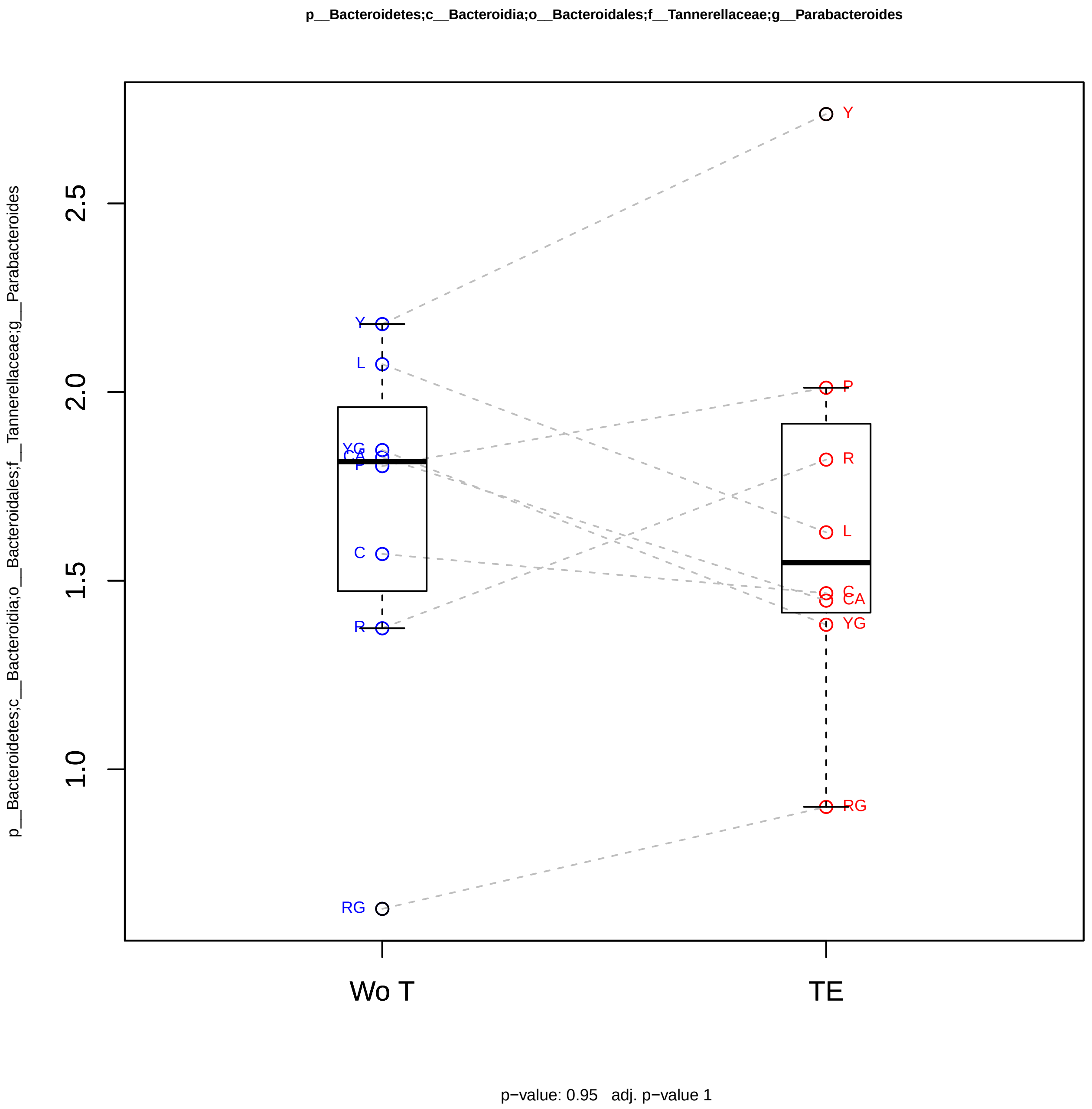
p__Bacteroidetes;c__Bacteroidia;o__Bacteroidales;f__Tannerellaceae;g__Parabacteroides
p__Bacteroidetes;c__Bacteroidia;o__Bacteroidales;f__Tannerellaceae;g__Parabacteroides
2.5
2.0
1.5
1.0
Y
L
YG
CA
P
C
R
RG
Y
P
R
L
C
CA
YG
RG
Wo T
TE
p−value: 0.95 adj. p−value 1

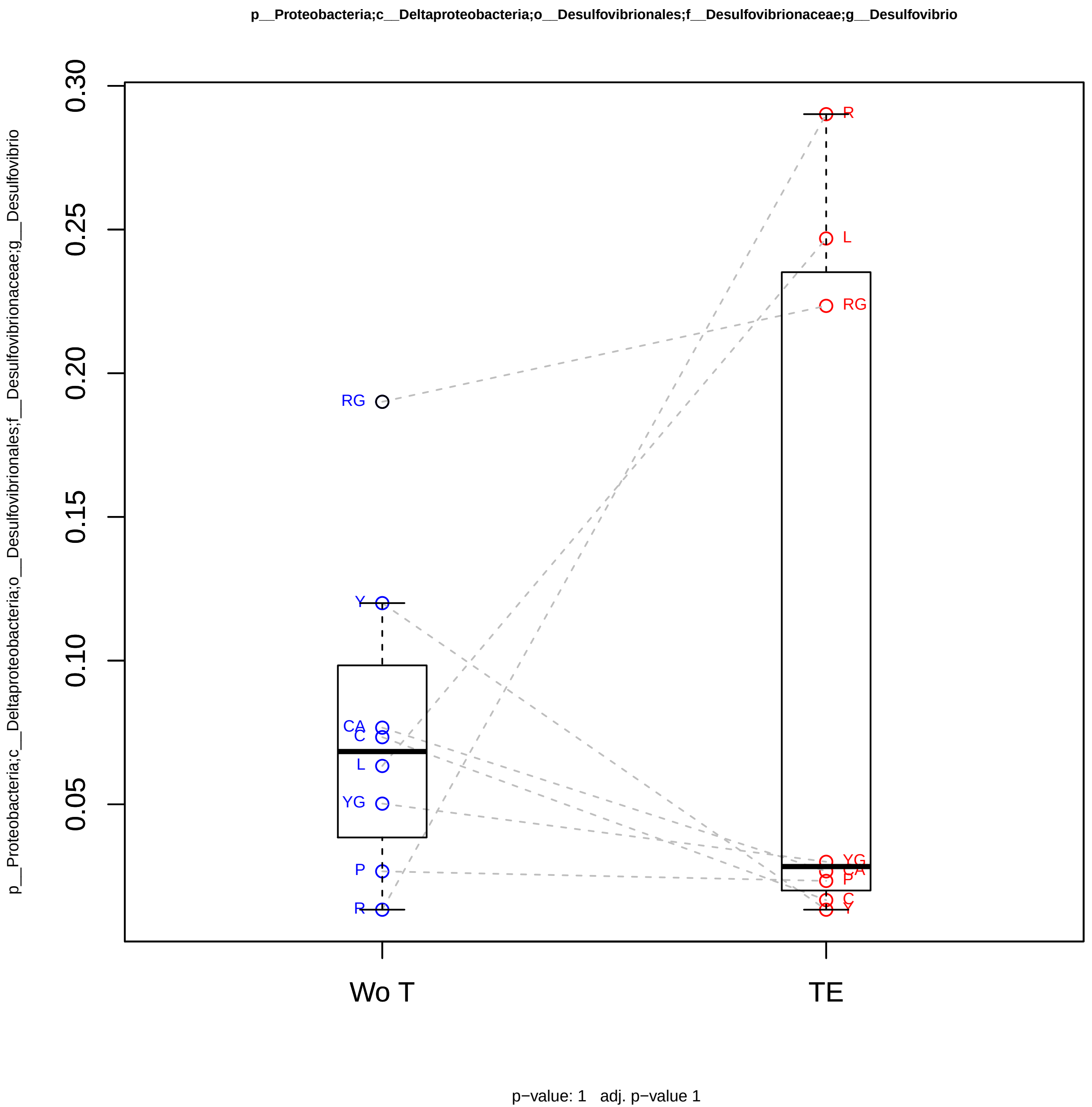
p__Proteobacteria;c__Deltaproteobacteria;o__Desulfovibrionales;f__Desulfovibrionaceae;g__Desulfovibrio
0.30
0.25
0.20
0.15
0.10
0.05
Wo T
TE
RG
Y
CA
C
L
YG
P
R
p−value: 1   adj. p−value 1

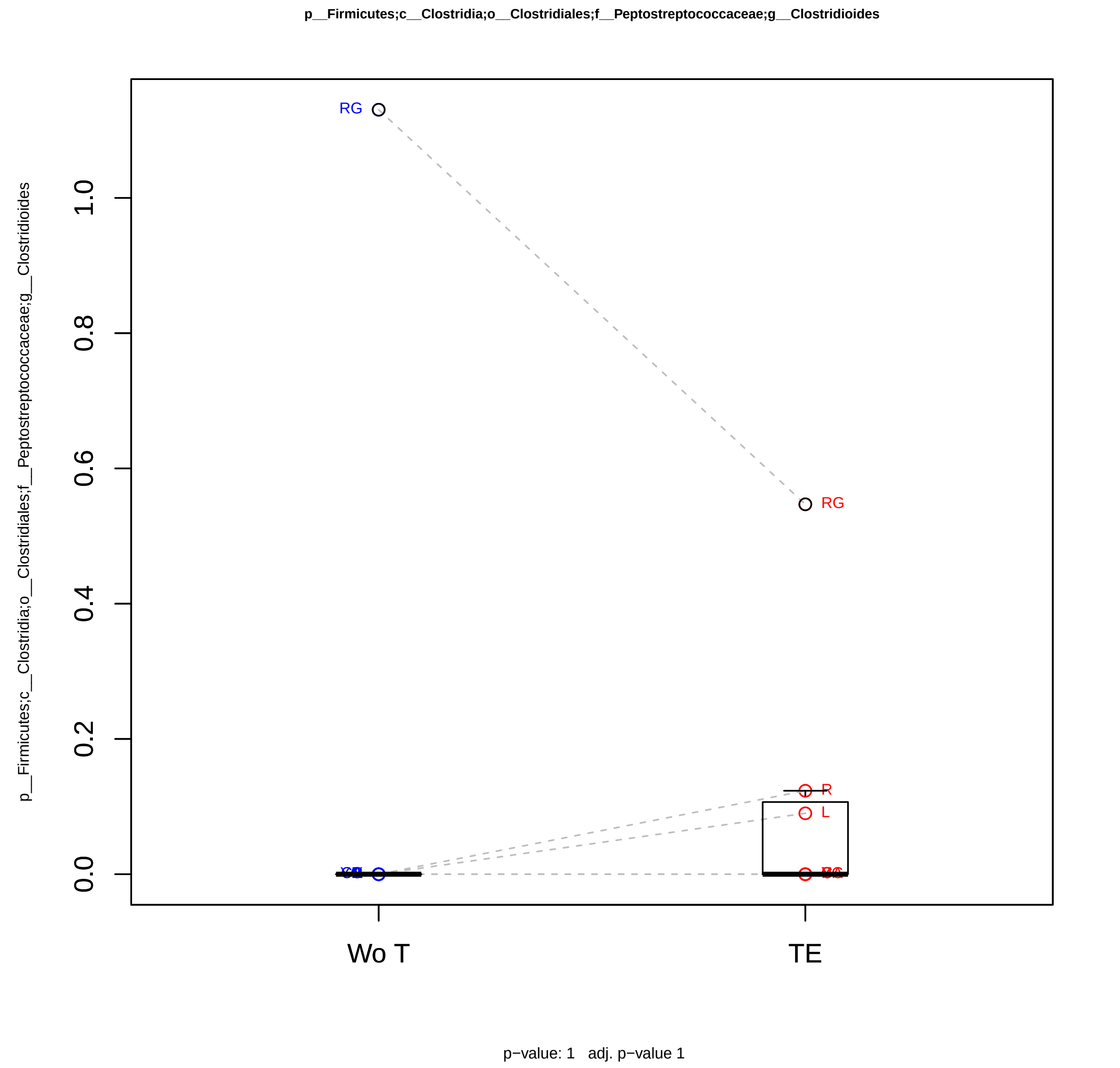
p__Firmicutes;c__Clostridia;o__Clostridiales;f__Peptostreptococcaceae;g__Clostridioides
p__Firmicutes;c__Clostridia;o__Clostridiales;f__Peptostreptococcaceae;g__Clostridioides
0.0
0.2
0.4
0.6
0.8
1.0
RG
RG
R
L
Wo T
TE
p−value: 1   adj. p−value 1

p__Firmicutes;c__Clostridia;o__Clostridiales;f__Lachnospiraceae;g__Tyzzerella_4

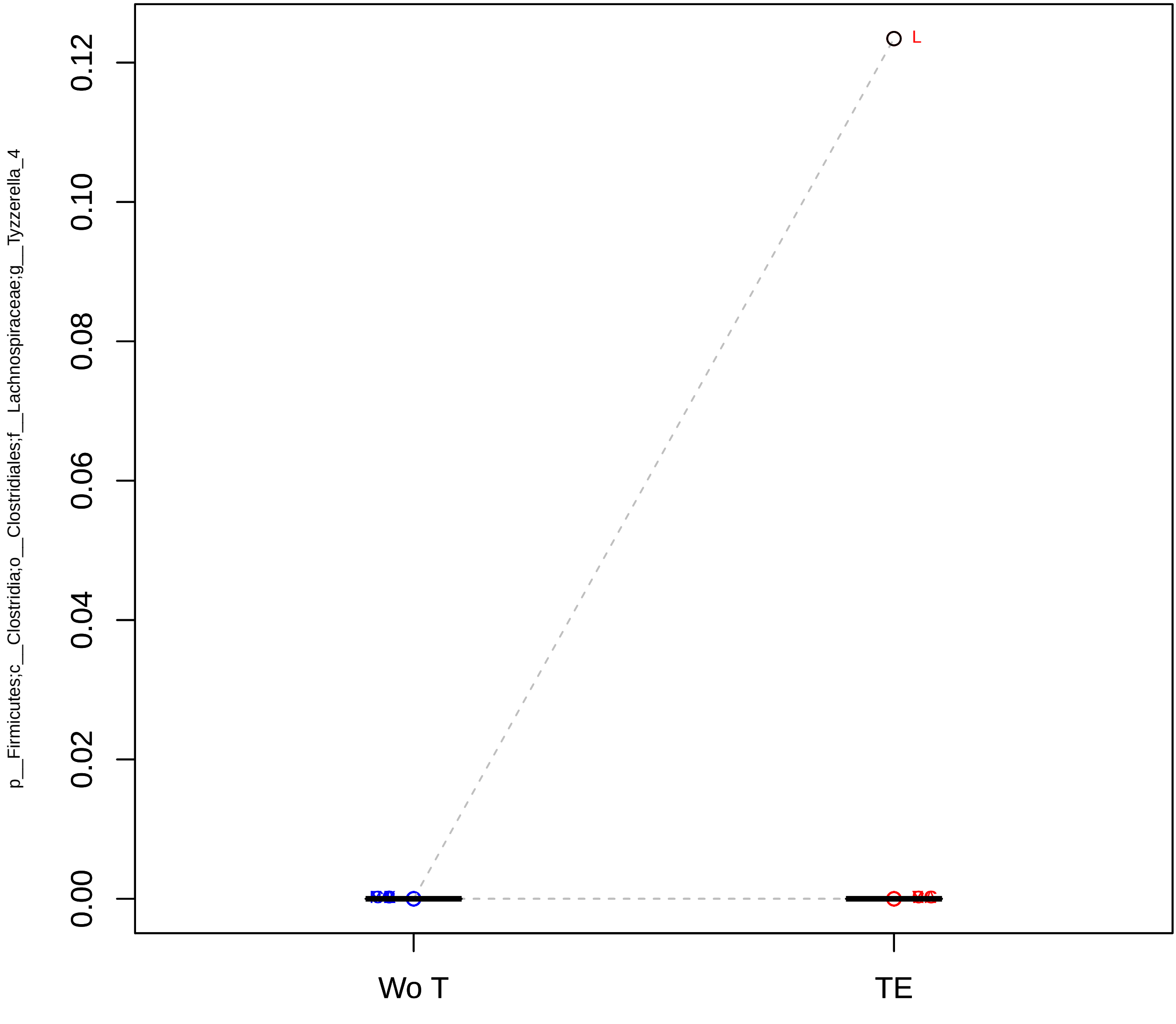

p−value: 1 adj. p−value 1

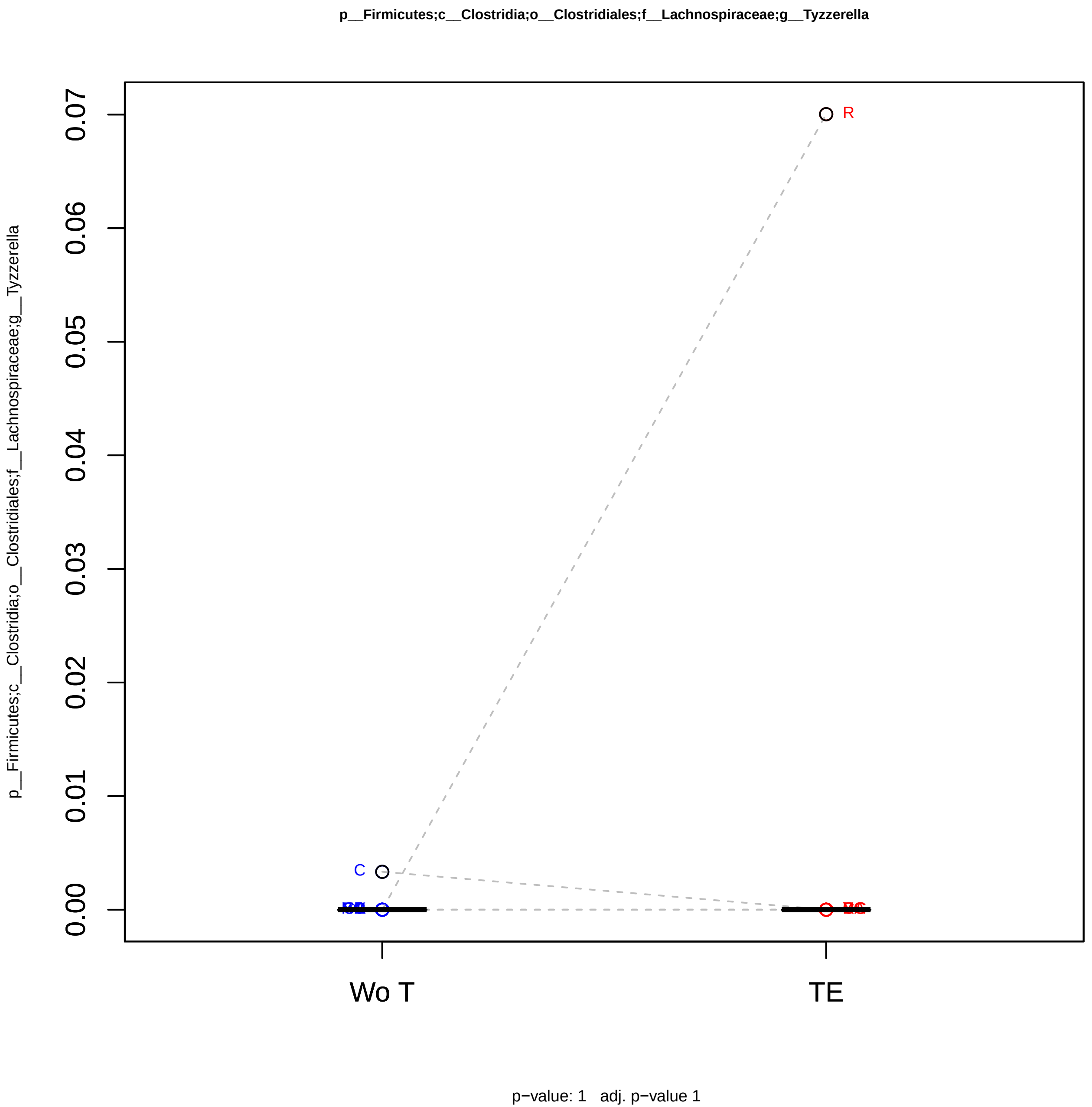
p__Firmicutes;c__Clostridia;o__Clostridiales;f__Lachnospiraceae;g__Tyzzerella
p__Firmicutes;c__Clostridia;o__Clostridiales;f__Lachnospiraceae;g__Tyzzerella
0.00
0.01
0.02
0.03
0.04
0.05
0.06
0.07
R
C
Wo T
TE
p−value: 1 adj. p−value 1

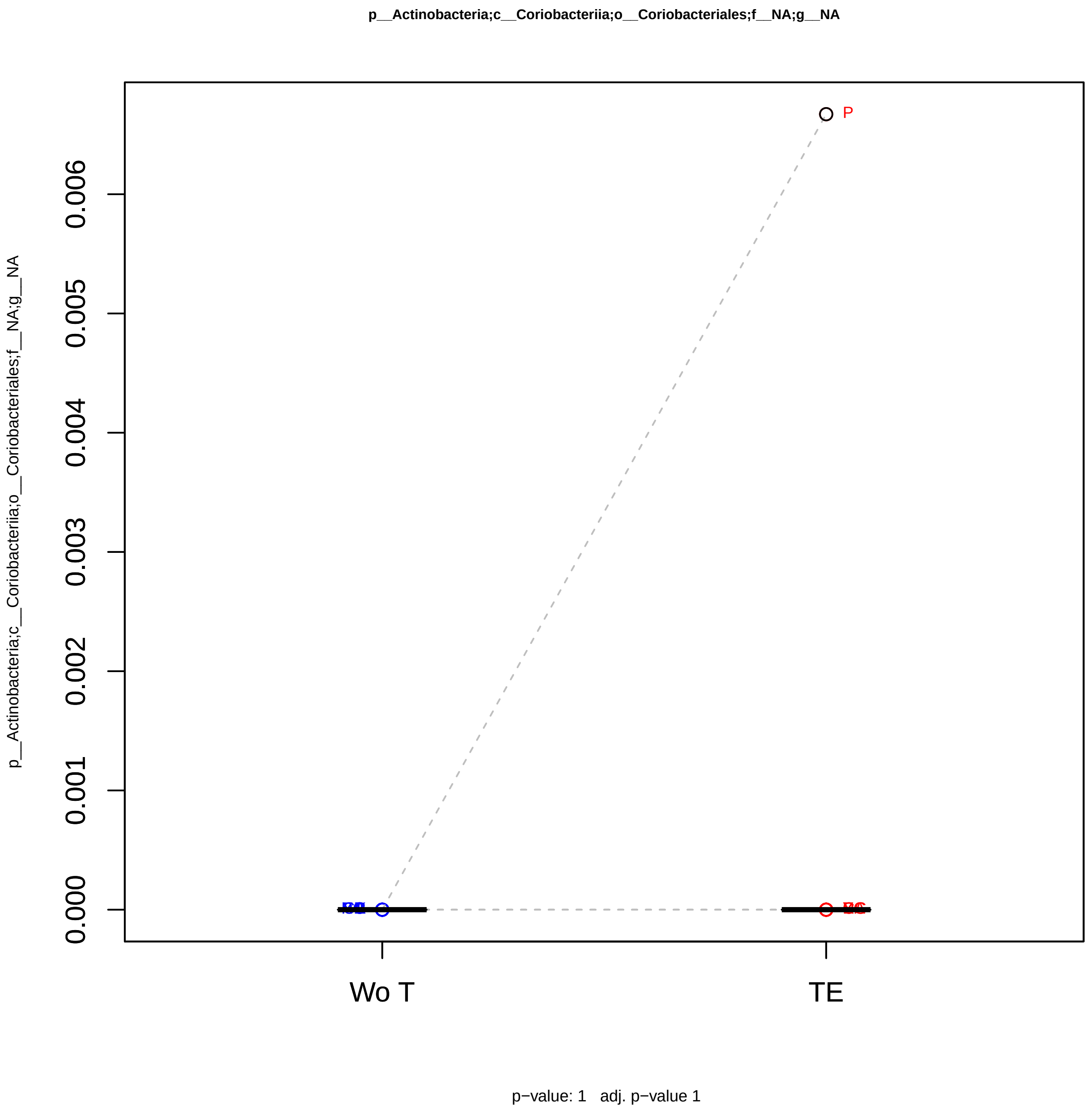
p__Actinobacteria;c__Coriobacteriia;o__Coriobacteriales;f__NA;g__NA
p__Actinobacteria;c__Coriobacteriia;o__Coriobacteriales;f__NA;g__NA
0.000
0.001
0.002
0.003
0.004
0.005
0.006
P
Wo T
TE
p−value: 1 adj. p−value 1

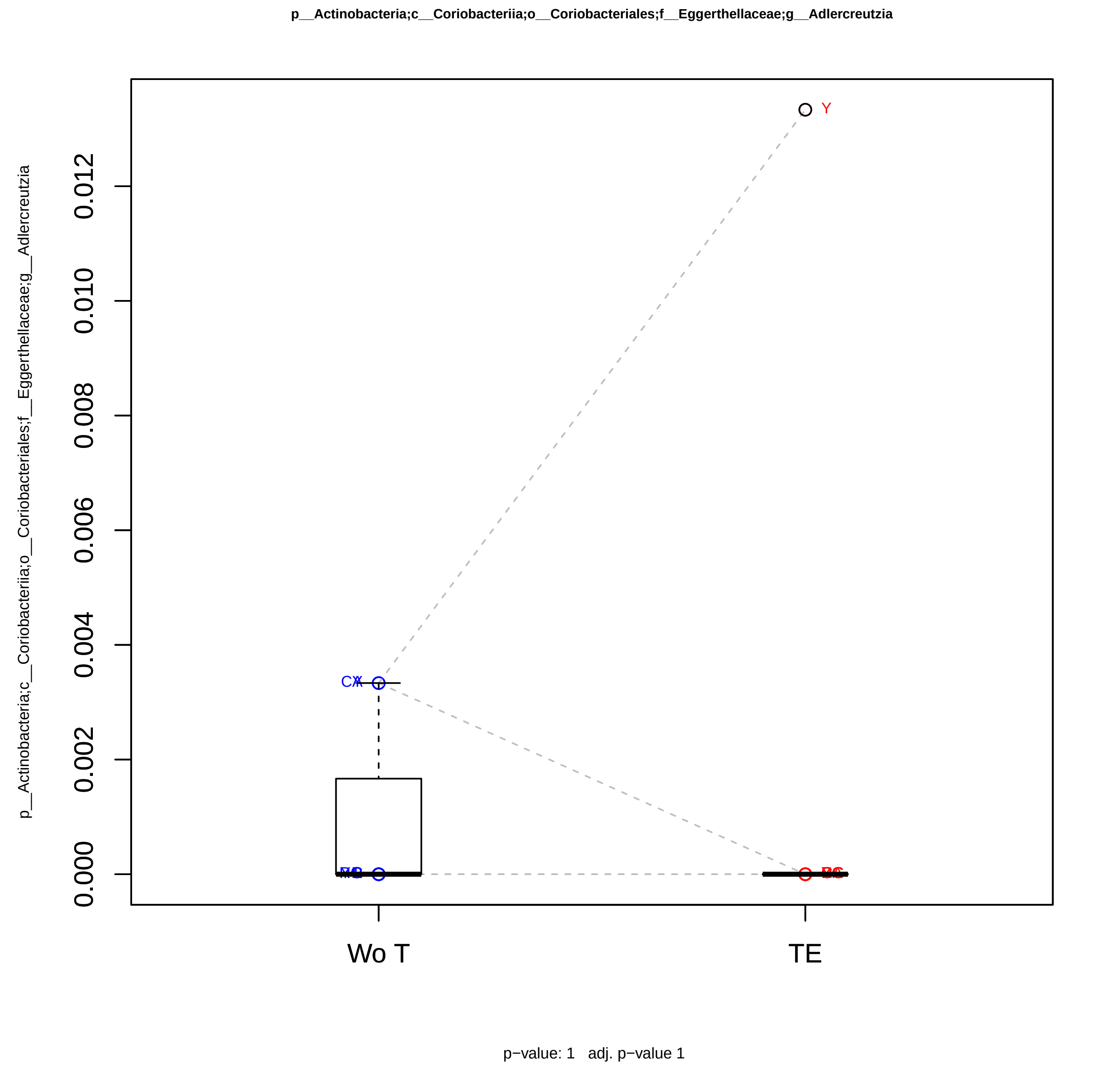

p__Actinobacteria;c__Coriobacteriia;o__Coriobacteriales;f__Eggerthellaceae;g__Adlercreutzia
p__Actinobacteria;c__Coriobacteriia;o__Coriobacteriales;f__Eggerthellaceae;g__Adlercreutzia
0.000
0.002
0.004
0.006
0.008
0.010
0.012
Y
Wo T
TE
p−value: 1   adj. p−value 1

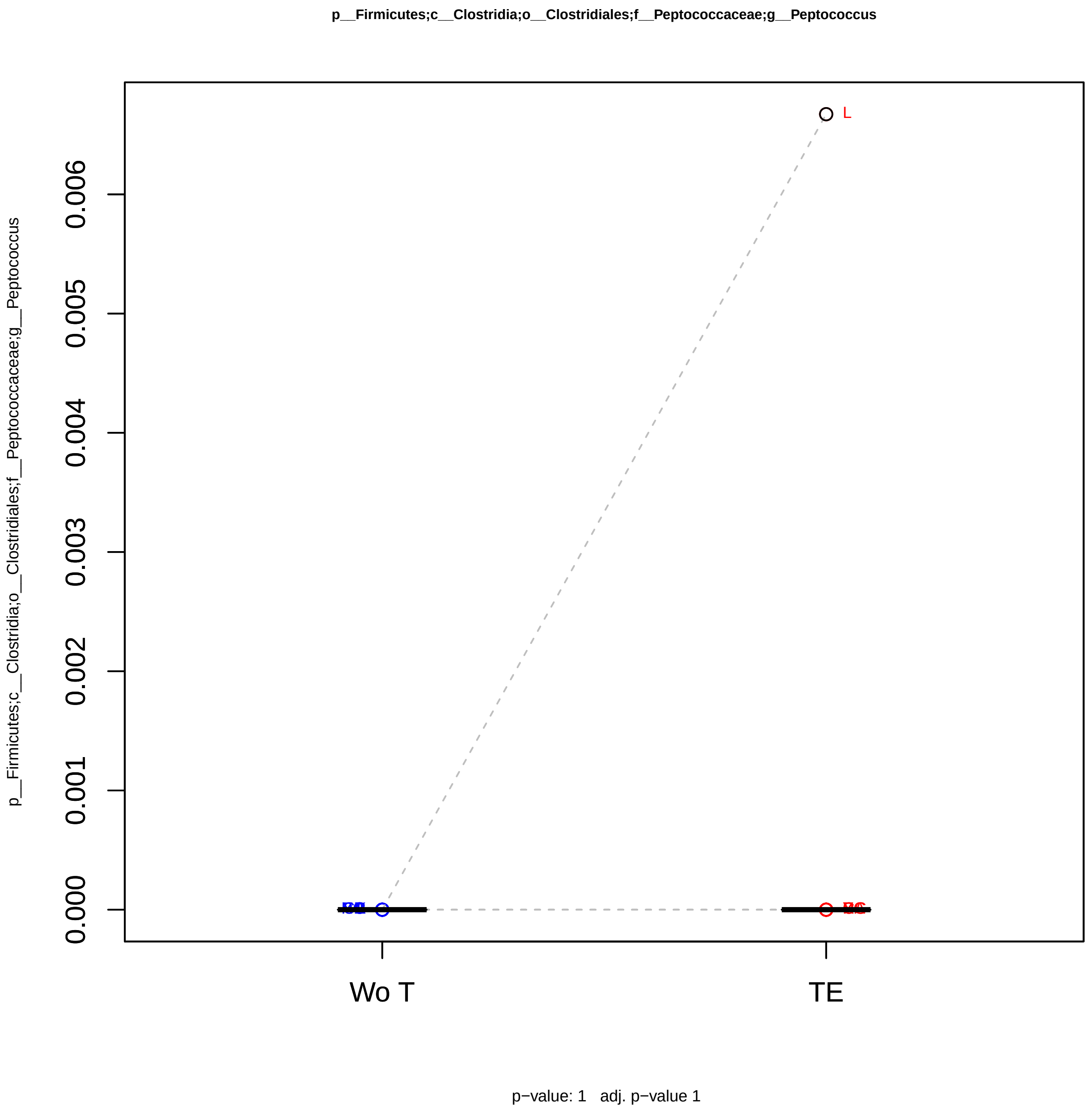
p__Firmicutes;c__Clostridia;o__Clostridiales;f__Peptococcaceae;g__Peptococcus
p__Firmicutes;c__Clostridia;o__Clostridiales;f__Peptococcaceae;g__Peptococcus
0.000
0.001
0.002
0.003
0.004
0.005
0.006
L
Wo T
TE
p−value: 1   adj. p−value 1

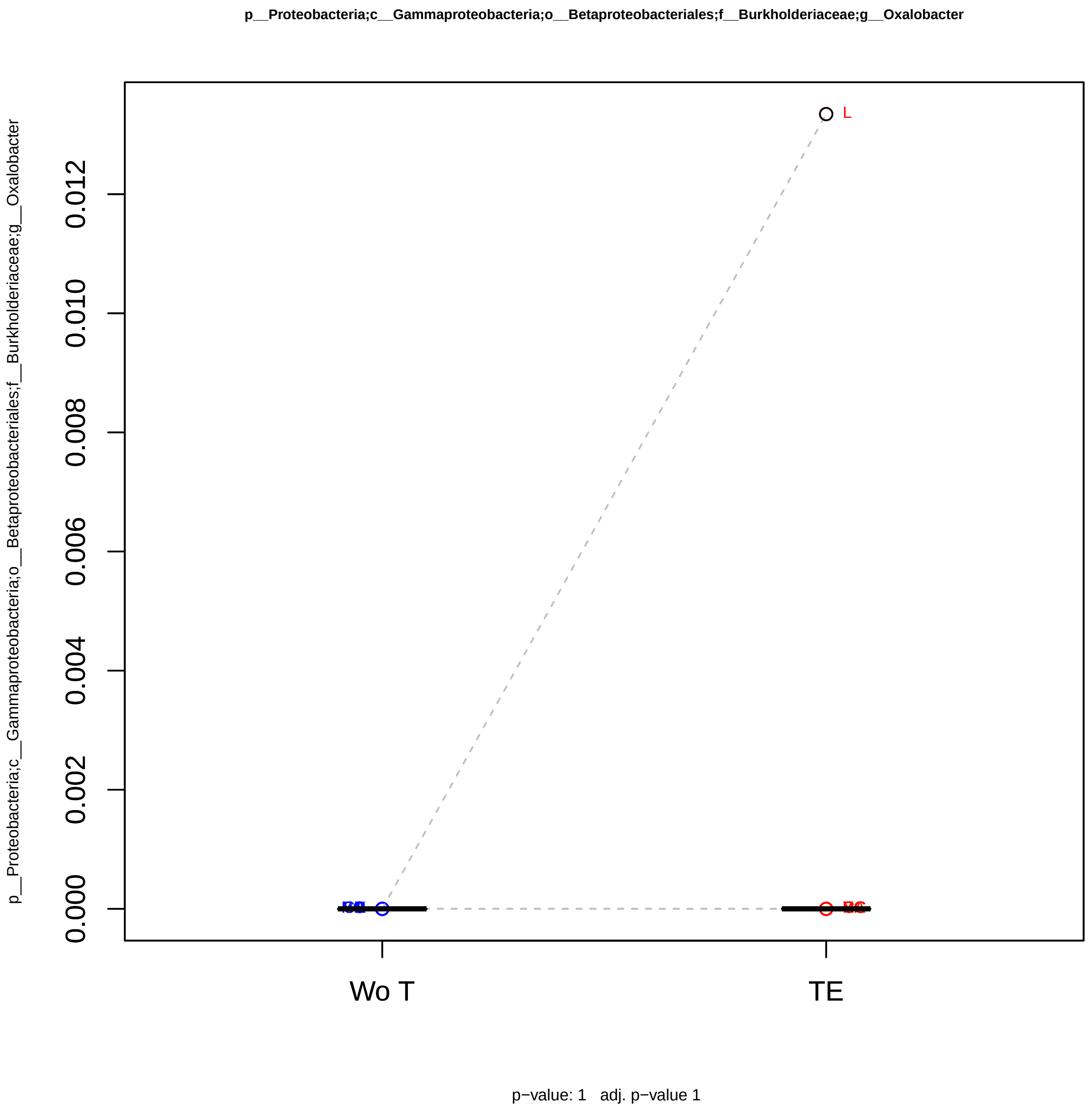
p__Proteobacteria;c__Gammaproteobacteria;o__Betaproteobacteriales;f__Burkholderiaceae;g__Oxalobacter
p__Proteobacteria;c__Gammaproteobacteria;o__Betaproteobacteriales;f__Burkholderiaceae;g__Oxalobacter
0.000
0.002
0.004
0.006
0.008
0.010
0.012
L
Wo T
TE
p−value: 1 adj. p−value 1

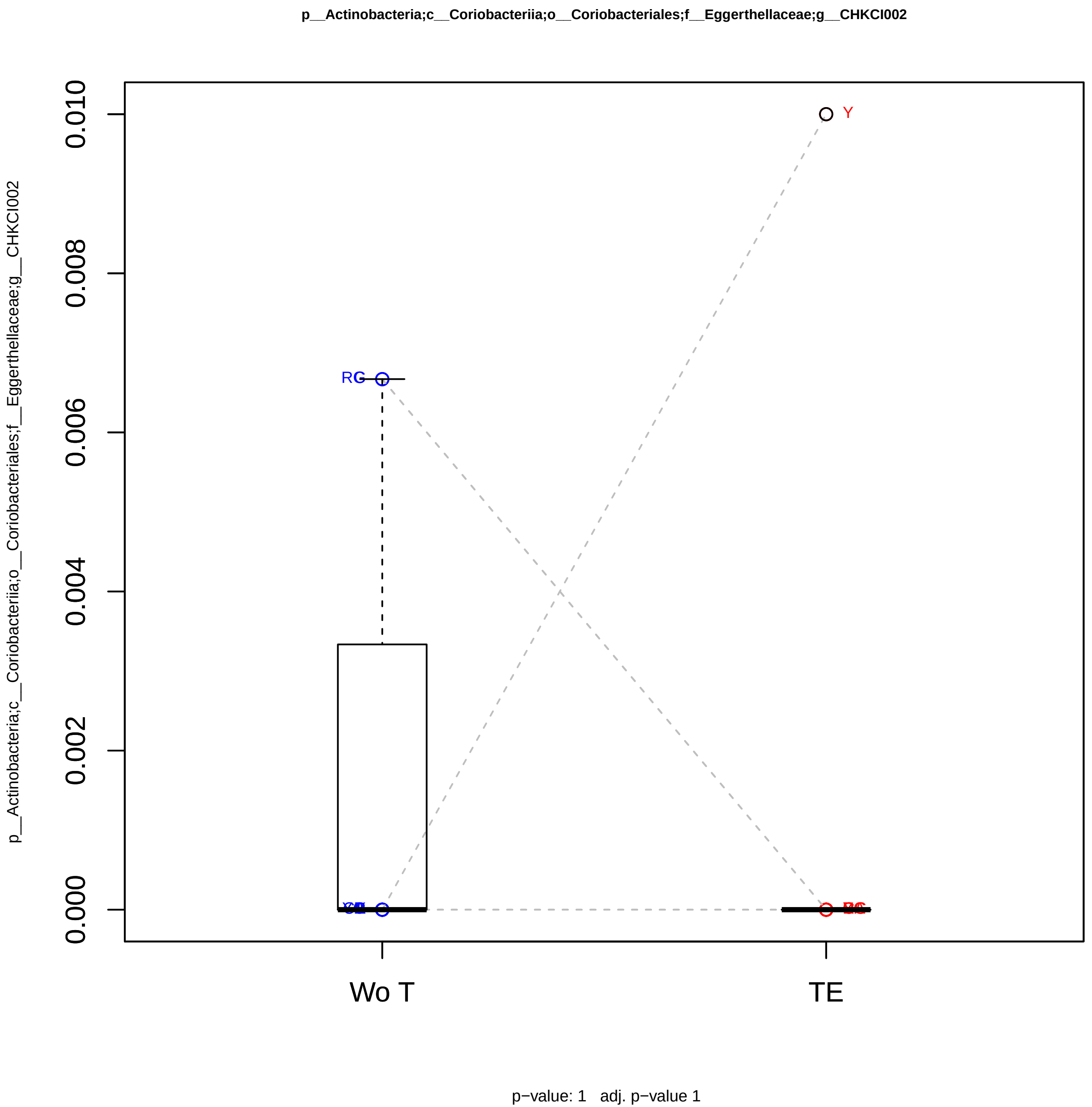

p__Actinobacteria;c__Coriobacteriia;o__Coriobacteriales;f__Eggerthellaceae;g__CHKCI002
p__Actinobacteria;c__Coriobacteriia;o__Coriobacteriales;f__Eggerthellaceae;g__CHKCI002
0.000
0.002
0.004
0.006
0.008
0.010
RG
Y
Wo T
TE
p−value: 1 adj. p−value 1

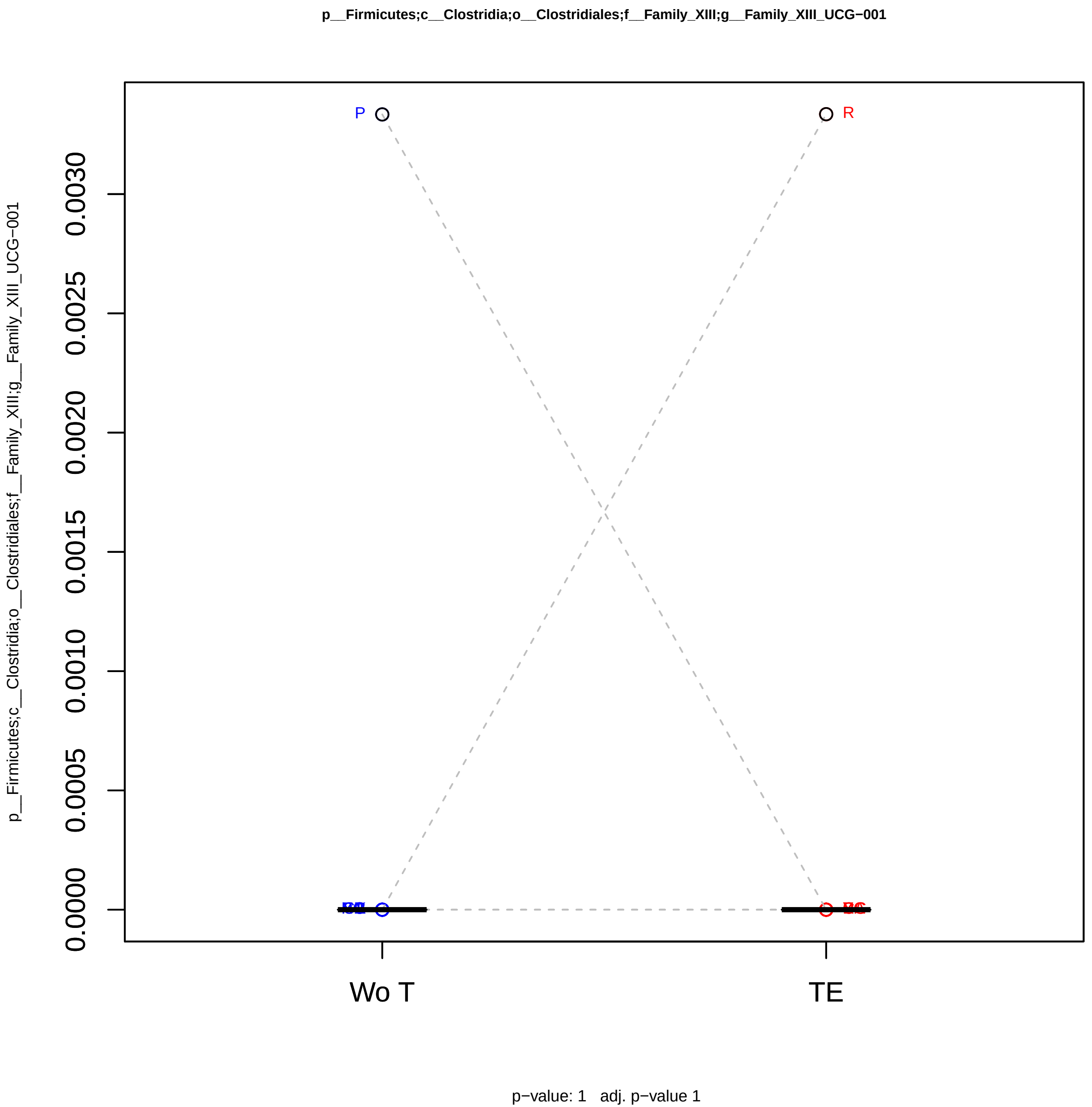
p__Firmicutes;c__Clostridia;o__Clostridiales;f__Family_XIII;g__Family_XIII_UCG−001
p__Firmicutes;c__Clostridia;o__Clostridiales;f__Family_XIII;g__Family_XIII_UCG−001
0.0000
0.0005
0.0010
0.0015
0.0020
0.0025
0.0030
P
R
Wo T
TE
p−value: 1 adj. p−value 1

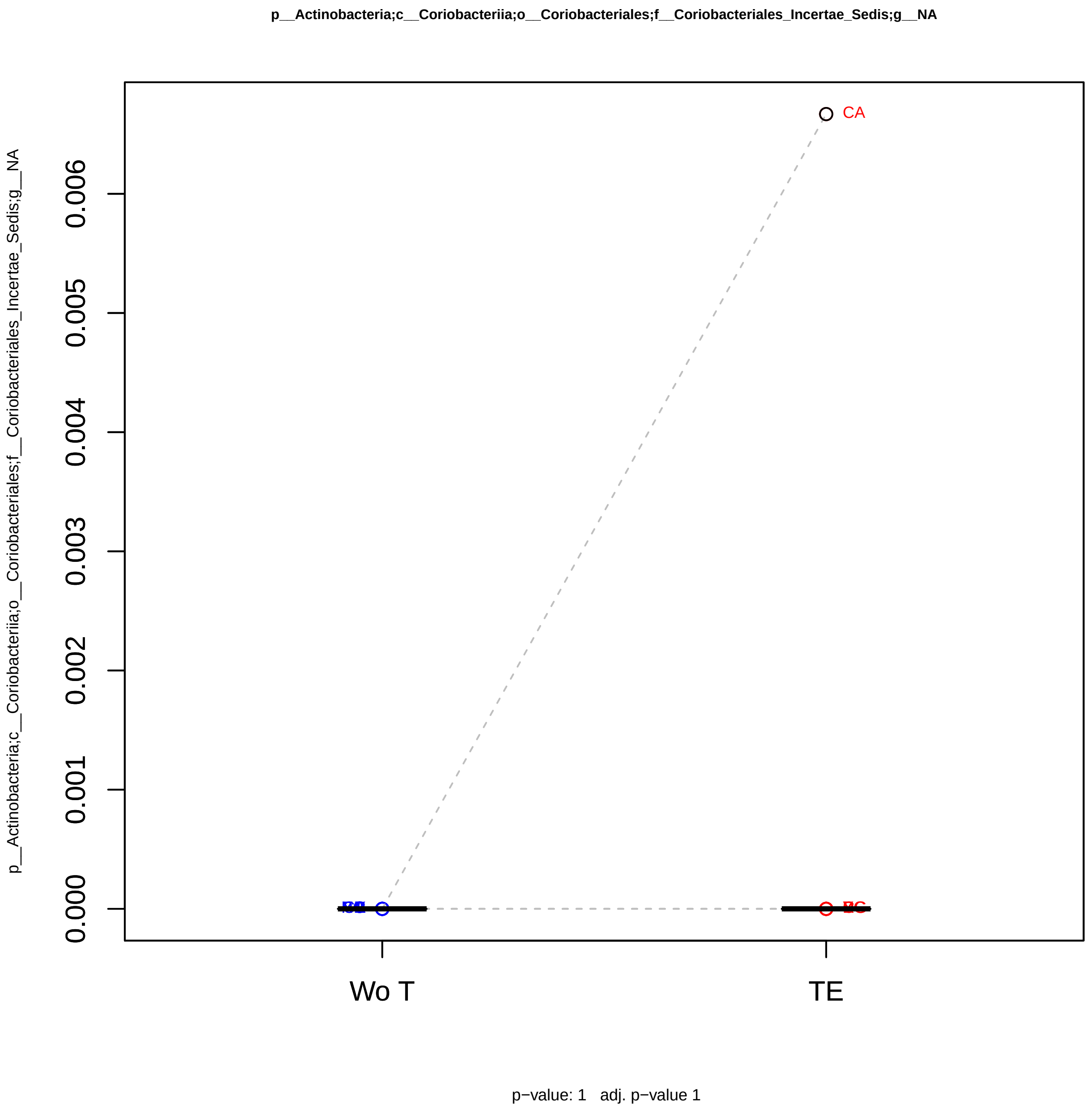
p__Actinobacteria;c__Coriobacteriia;o__Coriobacteriales;f__Coriobacteriales_Incertae_Sedis;g__NA
p__Actinobacteria;c__Coriobacteriia;o__Coriobacteriales;f__Coriobacteriales_Incertae_Sedis;g__NA
0.000
0.001
0.002
0.003
0.004
0.005
0.006
CA
Wo T
TE
p−value: 1 adj. p−value 1

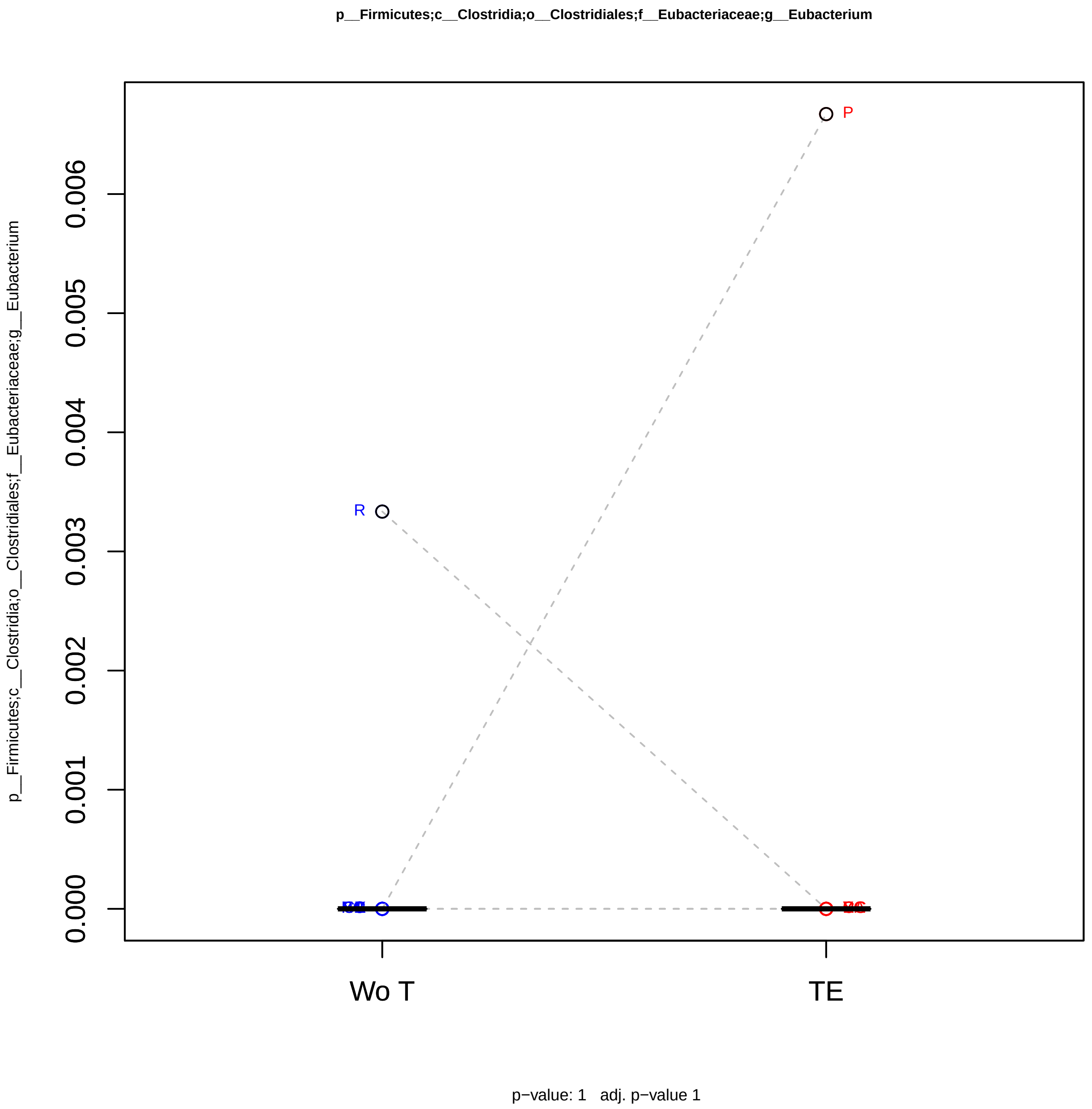
p__Firmicutes;c__Clostridia;o__Clostridiales;f__Eubacteriaceae;g__Eubacterium
p__Firmicutes;c__Clostridia;o__Clostridiales;f__Eubacteriaceae;g__Eubacterium
0.000
0.001
0.002
0.003
0.004
0.005
0.006
R
P
Wo T
TE
p−value: 1 adj. p−value 1

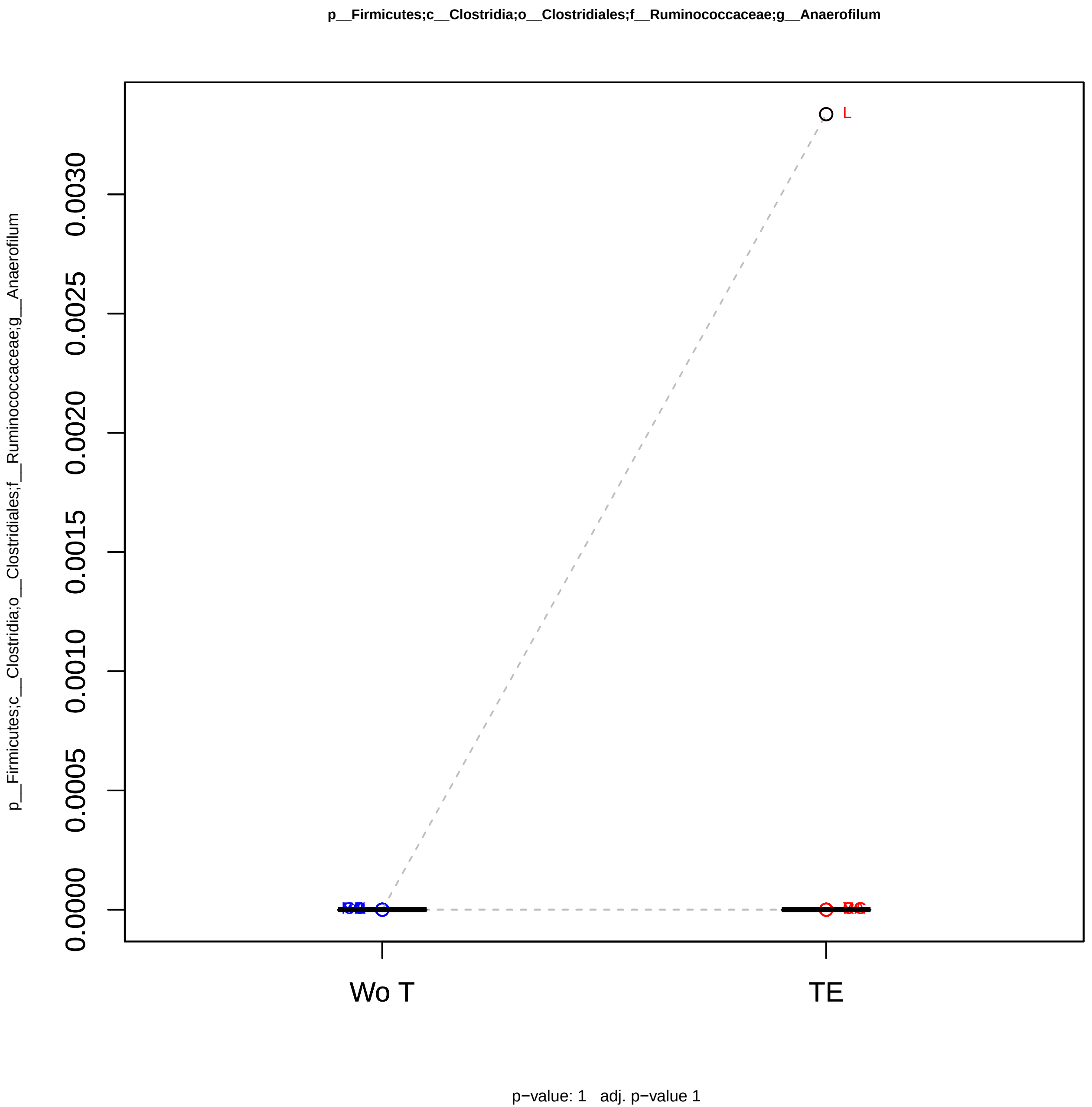
p__Firmicutes;c__Clostridia;o__Clostridiales;f__Ruminococcaceae;g__Anaerofilum
p__Firmicutes;c__Clostridia;o__Clostridiales;f__Ruminococcaceae;g__Anaerofilum
0.0000
0.0005
0.0010
0.0015
0.0020
0.0025
0.0030
L
Wo T
TE
p−value: 1   adj. p−value 1
